# Insights into snoRNA biogenesis and processing from PAR-CLIP of snoRNA core proteins and small RNA sequencing

**DOI:** 10.1186/gb-2013-14-5-r45

**Published:** 2013-05-26

**Authors:** Shivendra Kishore, Andreas R Gruber, Dominik J Jedlinski, Afzal P Syed, Hadi Jorjani, Mihaela Zavolan

**Affiliations:** 1 Computational and Systems Biology, Biozentrum, University of Basel, Klingelbergstrasse 50-70, 4056 Basel, Switzerland

## Abstract

**Background:**

In recent years, a variety of small RNAs derived from other RNAs with well-known functions such as tRNAs and snoRNAs, have been identified. The functional relevance of these RNAs is largely unknown. To gain insight into the complexity of snoRNA processing and the functional relevance of snoRNA-derived small RNAs, we sequence long and short RNAs, small RNAs that co-precipitate with the Argonaute 2 protein and RNA fragments obtained in photoreactive nucleotide-enhanced crosslinking and immunoprecipitation (PAR-CLIP) of core snoRNA-associated proteins.

**Results:**

Analysis of these data sets reveals that many loci in the human genome reproducibly give rise to C/D box-like snoRNAs, whose expression and evolutionary conservation are typically less pronounced relative to the snoRNAs that are currently cataloged. We further find that virtually all C/D box snoRNAs are specifically processed inside the regions of terminal complementarity, retaining in the mature form only 4-5 nucleotides upstream of the C box and 2-5 nucleotides downstream of the D box. Sequencing of the total and Argonaute 2-associated populations of small RNAs reveals that despite their cellular abundance, C/D box-derived small RNAs are not efficiently incorporated into the Ago2 protein.

**Conclusions:**

We conclude that the human genome encodes a large number of snoRNAs that are processed along the canonical pathway and expressed at relatively low levels. Generation of snoRNA-derived processing products with alternative, particularly miRNA-like, functions appears to be uncommon.

## Background

Small nucleolar RNAs (snoRNAs) are a specific class of small non-protein coding RNAs that are best known for their function as guides of modifications (2'-O-methylation and pseudouridylation) of other non-protein coding RNAs such as ribosomal, small nuclear and transfer RNAs (rRNAs, snRNAs and tRNAs, respectively) [1-3]. Based on sequence and structural features, snoRNAs are divided into two classes. C/D box snoRNAs share the consensus C (RUGAUGA, R = A or G) and D (CUGA) box motifs, which are brought into close proximity by short regions of complementarity between the snoRNA 5' and 3' ends [4,5] and are bound by the four core proteins of the small ribonucleoprotein complex (snoRNP), namely 15.5K, NOP56, NOP58 and Fibrillarin (FBL) [6-8] during snoRNA maturation. Fibrillarin is the methyltransferase that catalyzes the 2'-O-methylation of the ribose in target RNAs [9]. Most C/D box snoRNAs also contain additional conserved C' and D' motifs located in the central region of the snoRNA. The other class of snoRNAs is defined by a double-hairpin structure with two single-stranded H (ANANNA, N = A, C, G or U) and ACA box domains [10], and are therefore called H/ACA box snoRNAs. They associate with four conserved proteins, Dyskerin (DKC1), Nhp2, Nop10 and Gar1, to form snoRNPs that are functionally active in pseudouridylation. Although all four H/ACA proteins are necessary for efficient pseudouridylation [10], it is Dyskerin that provides the pseudouridine synthase activity [11]. While H/ACA and C/D box snoRNAs accumulate in the nucleolus, some snoRNAs reside in the nucleoplasmic Cajal bodies (CBs) where they guide modifications of snRNAs [2] and are called small Cajal body-specific RNAs (scaRNAs). In addition to the typical H/ACA snoRNA features, vertebrate H/ACA box scaRNAs carry a CB localization signal called CAB box (UGAG) in the loop of their 5' and/or 3' hairpins [12].

Immediately upstream of the D box and/or the D' box, C/D box snoRNAs contain 10 to 21 nucleotide-long antisense elements that are complementary to sites in their target RNAs [13-15]. The nucleotide in the target RNA that is complementary to the fifth nucleotide upstream from the D/D' box of the snoRNA is targeted for 2'-O-methylation by the snoRNP [14,15]. H/ACA box snoRNAs contain two antisense elements termed pseudouridylation pockets, located in the 5' and 3' hairpin domains of the snoRNA [16,17]. Substrate uridines are selected through base-pairing interactions between the pseudouridylation pocket and target RNA sequences that flank the targeted uridine.

Deep-sequencing studies revealed a surprising diversity of small RNAs derived from non-coding RNAs (ncRNAs) known as small derived RNAs (sdRNAs) with well-established functions such as tRNAs [18,19], Y RNAs [20], vault RNAs [21], ribosomal RNAs [22], spliceosomal RNAs [23] and snoRNAs [24-26]. In fact, the profile of sequenced reads observed for some of these small RNA species are very characteristic and have even been used for ncRNA gene finding based on sequencing data [27,28]. The majority of C/D box and H/ACA snoRNAs seems to be extensively processed, producing stable small RNAs from the termini of the mature snoRNA [29] and the processing pattern is conserved across cell types [30]. Thus, it appears that snoRNAs are versatile molecules that give rise to snoRNA-derived miRNAs [24,31], other small RNAs [25,29] or longer processing fragments [32].

To gain insight into the complexity of snoRNA processing and the functional relevance of the derived sdRNAs, we undertook a comprehensive characterization of products generated from snoRNA loci, combining high-throughput sequencing of long and short RNA fragments with photoactivatable-ribonucleoside-enhanced cross-linking and immunoprecipitation (PAR-CLIP) of core snoRNA-associated proteins and with data from Argonaute 2 (Ago2) immunoprecipitation sequencing (IP-seq) experiments. We found that many loci in the human genome can give rise to C/D box-like snoRNAs. Among the novel snoRNAs that we identified are very short sequences, extending little beyond the C and D boxes, which are essential for the binding of core snoRNA proteins. Compared to the snoRNAs that are already known, the novel snoRNA candidates exhibit a lower level of evolutionary conservation and a lower expression level. These findings indicate that the C/D box snoRNA structure evolves relatively easily and that C/D box snoRNA-like molecules are produced from many more genomic loci than are currently annotated. We further found that C/D box snoRNAs are very specifically processed inside the regions of terminal complementarity, retaining in the mature form only four to five nucleotides upstream of the C box and two to five nucleotides downstream of the D box. Sequencing of the small RNA population as well as of the small RNAs isolated after Ago2 immunoprecipitation revealed that despite their cellular abundance, C/D box-derived small RNAs are not efficiently incorporated into the Ago2 protein. Our extensive data thus indicate that, contrary to previous suggestions [25,33], snoRNA-derived small RNAs that carry out non-canonical, particularly miRNA-like, functions are rare.

## Results

### PAR-CLIP of C/D box and H/ACA box snoRNP core proteins identifies their RNA binding partners

To investigate the RNA population comprehensively that associates with both C/D box and H/ACA box small nucleolar ribonucleoproteins we performed PAR-CLIP as previously described [34] with antibodies against the endogenous Fibrillarin (FBL), NOP58 and Dyskerin (DKC1) proteins, in HEK293 cells (for details see Materials and methods). For NOP56 we used a stable cell line expressing FLAG-tagged NOP56 and anti-FLAG antibodies. Because we recently found that the choice of the ribonuclease and reaction conditions influences the set of binding sites obtained through cross-linking and immunoprecipitation (CLIP) [35], we also generated a Fibrillarin PAR-CLIP library employing partial digestion with micrococcal nuclease (MNase) instead of RNase T1. PAR-CLIP libraries were sequenced on Illumina sequencers, mapped and annotated through the CLIPZ web server [36]. The obtained libraries were comparable to those from previous PAR-CLIP studies in terms of size, rates of mapping to genome and proportion of cross-link-indicative T→C mutations (Table 1). The DKC1 PAR-CLIP library shows a lower frequency of T→C mutations compared to all other libraries, but T→C mutations were still the most frequent in this library as well (data not shown).

**Table 1:** Summary of CLIPZ mapping statistics and annotation categories for PAR-CLIP samples.

Feature	FBL	FBL (MNase)	NOP56	NOP58 rep A	NOP58 rep B	DKC1	Ago2 rep A	HuR rep A
Mapping rate	60.47%	73.3%	26.6%	41.4%	46.6%	47.5%	67.9%	72.4%
Library size	3,755,090	7,396,138	2,789,209	3,678,032	3,798,895	7,727,966	5,899,130	5,491,479
T→C mutations among all observed mutations	64.8%	57.7%	48.6%	67.9%	73.0%	19.7%	55.8%	58.8%
snoRNAs	33.79%	31.55%	29.95%	39.05%	44.10%	13.13%	0.18%	0.01%
snRNAs	20.87%	33.17%	15.45%	22.36%	25.60%	10.18%	0.28%	0.02%
rRNAs	18.64%	13.83%	8.12%	7.42%	7.16%	15.53%	1.07%	0.17%
mRNAs	14.47%	11.61%	22.27%	19.42%	15.14%	17.40%	50.07%	47.87%
Repeats	6.42%	1.60%	15.51%	6.08%	3.36%	18.39%	11.29%	42.08%
tRNAs	1.57%	2.67%	2.44%	0.99%	0.57%	5.10%	0.75%	0.14%
miRNAs	0.07%	0.18%	0.02%	0.01%	0.01%	0.05%	20.41%	00.00%
Other Categories	2.74%	3.66%	3.01%	2.98%	2.78%	2.80%	3.86%	1.99%
No annotation	1.43%	1.74%	3.21%	1.69%	1.27%	17.43%	12.10%	7.71%

Ago2: Argonaute 2; DKC1: Dyskerin; FBL: Fibrillarin; miRNA: micro RNA; MNase: micrococcal nuclease; PAR-CLIP: photoactivatable-ribonucleoside-enhanced cross-linking and immunoprecipitation; rRNA: ribosomal RNA; snoRNA: small nucleolar RNA; snRNA: small nuclear RNA; tRNA: transfer RNA

Compared to the libraries that we previously generated for HuR and Ago2 [35], two proteins whose primary targets are mRNAs, we found that snoRNAs, rRNAs and snRNAs were strongly enriched in PAR-CLIP libraries generated for the snoRNP core proteins (Table 1). The fact that not only snoRNAs but also the primary targets of snoRNAs, namely ribosomal RNAs and small nuclear RNAs, are enriched in these samples suggests that like Ago2 cross-linking, which captures both miRNAs and their targets [34,35], cross-linking of core snoRNPs efficiently captures both snoRNAs and targets. To quantify the specificity of our PAR-CLIP libraries, we intersected the 200 clusters with the highest read density per nucleotide from each library with curated snoRNA gene annotations based on snoRNA-LBME-db [37] (Table 2). Currently, snoRNA-LBME-db lists about 153 human C/D box snoRNA loci and 108 human H/ACA box snoRNA loci that are known to be ubiquitously expressed. For each of the C/D box specific PAR-CLIP libraries, more than 100 of the top 200 clusters could be assigned to C/D box snoRNAs indicating the specificity of our CLIP experiments and the broad coverage of the snoRNA genes by the sequencing reads obtained from HEK293 cells. The Dyskerin PAR-CLIP data set showed a weaker enrichment in snoRNAs compared to the data sets for the core C/D box-specific proteins, with 57% of all known H/ACA box snoRNAs being represented among the 200 top-ranking clusters. scaRNAs were detected in both H/ACA box and C/D box specific libraries, as expected because many scaRNAs have both C/D box and H/ACA box elements. Finally, minor fractions of H/ACA box snoRNAs were also found in PAR-CLIP libraries of the C/D box-specific proteins, and *vice versa*. This could be caused by the close spatial arrangement of snoRNPs on the target molecule, or could indicate that H/ACA box snoRNAs and C/D box snoRNAs guide modifications on each other.

**Table 2:** Annotation summary of the top 200 clusters inferred from PAR-CLIP experiments with snoRNA core proteins.

PAR-CLIP library	C/D box snoRNAs	H/ACA box snoRNAs	scaRNAs	mRNA exons	Other
FBL	123 (61.5%)	9 (4.5%)	10 (5.0%)	5 (2.5%)	53 (26.5%)
FBL (MNase)	106 (53.0%)	16 (8.0%)	10 (5.0%)	26 (13.0%)	42 (21.0%)
NOP56	115 (57.5%)	28 (14.0%)	15 (7.5%)	2 (1.0%)	40 (20.0%)
NOP58 rep A	114 (57.0%)	14 (7.0%)	10 (5.0%)	9 (4.5%)	52 (26.0%)
NOP58 rep B	125 (62.5%)	4 (2.0%)	10 (5.0%)	9 (4.5%)	52 (26.0%)
DKC1	11 (5.5%)	62 (32.0%)	18 (9.0%)	7 (3.5%)	102 (51.0%)
Ago2 rep A	0 (0.0%)	0 (0.0%)	1 (0.5%)	59 (29.5%)	140 (70.0%)
HuR rep A	0 (0.0%)	0 (0.0%)	0 (0.0%)	117 (58.5%)	83 (41.5%)

Ago2: Argonaute 2; DKC1: Dyskerin; FBL: Fibrillarin; MNase: micrococcal nuclease; PAR-CLIP: photoactivatable-ribonucleoside-enhanced cross-linking and immunoprecipitation; scaRNA: small Cajal body-specific RNA; snoRNA: small nucleolar RNA;

### Binding patterns of core proteins on snoRNAs

**Figure 1:**
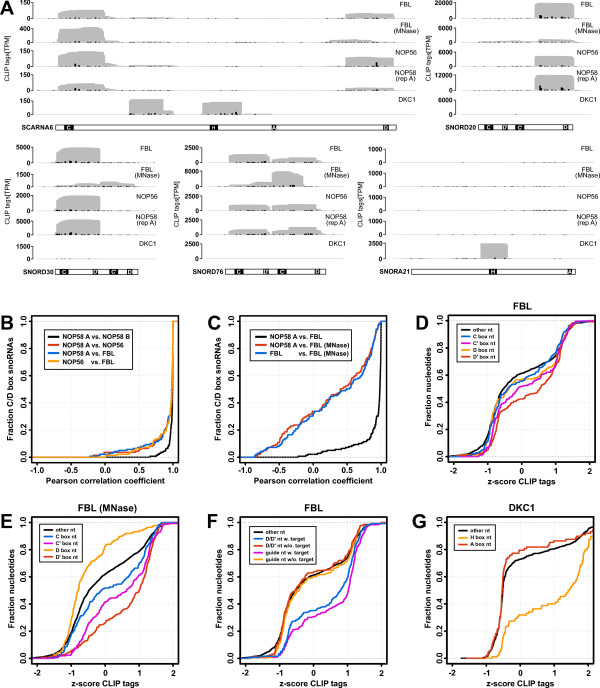
**Summary of PAR-CLIP data of snoRNP core proteins**. **(A) **Profiles of sequencing reads obtained from PAR-CLIP experiments for selected snoRNAs. Black bars in the profiles indicate the number of T→C mutations observed in PAR-CLIP reads at a particular nucleotide. **(B) **Similarity of binding profiles of core proteins that associate with C/D box snoRNAs. **(C) **Comparison of protein binding profiles as inferred from RNase T1-treated and MNase-treated PAR-CLIP samples. **(D, E) **Preferential binding of Fibrillarin to box elements as inferred from PAR-CLIP samples prepared with T1 (D) and MNase ribonucleases (E). **(F) **Comparison of binding preferences at D'/D box elements and guide regions for snoRNAs with and without a known target. **(G) **Analysis of binding preferences of Dyskerin for H/ACA box snoRNA-specific elements. D, E, F and G show the cumulative distributions of CLIP read coverage *z*-scores for nucleotides located in various regions of the snoRNA relative to the overall coverage of the snoRNA. CLIP: cross-linking and immunoprecipitation; MNase: micrococcal nuclease; PAR-CLIP: photoactivatable-ribonucleoside-enhanced cross-linking and immunoprecipitation; snoRNA: small nucleolar RNA; snoRNP: small nucleolar ribonucleoprotein

As mentioned in the introduction, both C/D box and H/ACA box snoRNAs carry very specific functional sequence and structure elements, which are recognized by the snoRNP core proteins. We thus asked whether different C/D box core proteins have distinct preferences in binding different regions of the C/D box snoRNAs. Figure 1A depicts PAR-CLIP read profiles along selected snoRNA genes (profiles for all scaRNA and snoRNA genes are in Additional file 1). Both C/D box core proteins as well as the H/ACA box specific Dyskerin bind to SCARNA6, which has a hybrid structure composed of both C/D box and H/ACA box elements. However, while the CLIP reads from the Fibrillarin, NOP56 and NOP58 samples cover the C and D box motifs, Dyskerin was preferentially cross-linked to the H-box motif and to the 5' end of the first H/ACA box stem. For the C/D box snoRNAs, different snoRNA core proteins gave very similar cross-linking patterns (Figure 1B), which we quantified through the correlation coefficient between read densities obtained along individual snoRNAs in pairs of samples. Comparing NOP58 to Fibrillarin and NOP56 we found that 109 (78%) and 111 (80%) snoRNA genes had a correlation coefficient of at least 0.9. To put this in perspective, between biological replicates of NOP58, 130 out of 139 snoRNAs investigated have a correlation coefficient of at least 0.9. This indicates that Fibrillarin, NOP56 and NOP58 form a tight complex that contacts the snoRNA. As noticed before, however [35], the nuclease treatment has a strong influence on the relative number of tags obtained from different positions along a snoRNA (Figure 1C). Only 19 snoRNA genes (14%) show a correlation ≥ 0.90 in their tag profiles obtained with RNase T1- and MNase-treated Fibrillarin PAR-CLIP samples, reflecting the fact that T1 nuclease is more efficient and generates a more biased position-dependent distribution of reads than MNase (Figure 1A). Figures 1D and Figure 1E summarize these results, showing that nucleotides in D' boxes are most frequently cross-linked, followed by nucleotides in the C' and C boxes, and then by nucleotides in the D box and in the rest of the snoRNA. MNase treatment in particular results in very poor coverage of the D box. On the other hand, we observed gene-specific differences in the binding of the core proteins. For example, SNORD20 only shows a peak of CLIP reads at the D box, SNORD30 only at the C box, while SNORD76 has peaks at both C and D boxes (Figure 1A).

We further asked whether the binding pattern of Fibrillarin reflected in the abundance of CLIP reads differs between guide regions of the snoRNAs that have a target annotated in snoRNA-LBME-db and orphan guide regions. For guide regions, we took the nine nucleotides upstream of the D and D' boxes and as a reference we compared the coverage of the D and D' boxes themselves (Figure 1F). We found that guide regions with a known target and their associated D/D' boxes generally have a higher coverage compared to those that are orphan (70% compared to 40% positive *z*-scores of the average coverage per position in the guide region relative to the entire snoRNA, Figure 1G). This could indicate that the binding to the target renders the snoRNA-core protein complex more accessible to cross-linking.

For H/ACA box snoRNAs we found that Dyskerin strongly prefers the H box nucleotides (Figure 1G), which in 70% of the snoRNAs have a positive *z*-score for coverage compared to the entire snoRNA. This is expected because these snoRNAs are highly structured, with most nucleotides being engaged in base pairs in the two hairpin stems and a few nucleotides are free to interact with the proteins.

### Identification of novel snoRNA genes from PAR-CLIP and small RNA sequencing

We screened the top 500 clusters from each PAR-CLIP library that did not overlap with known ncRNAs, mRNAs or repeat elements for potentially novel snoRNA genes. To identify H/ACA box genes we employed the SnoReport program [38], while for C/D box snoRNA detection we applied a custom approach searching for a C box motif (RUGAUGA, R = A or G; allowing one mismatch) at the 5' end and a D box motif (MUGA, M = A or C) at the 3' end, requiring that a terminal stem of at least four canonical base pairs can be formed by the nucleotides flanking the C and D boxes. We combined these computational screens with isolation and sequencing of the 20 to 200 nucleotide RNA fraction from HEK293 cells, which provides evidence for expression of the predicted snoRNAs. Requiring a minimal average coverage per nucleotide of at least 1 tag per million (TPM) in least one type-specific CLIP library as well as in the small RNA-seq library, we identified 77 and 20 putative C/D and H/ACA box snoRNAs, respectively (Additional files 2 and 3). We additionally screened 14 distinct small RNA sequence libraries from the recently released ENCODE data [39] and found that more than 75% of our putative C/D box snoRNAs were detected in at least one cell type other than HEK293 (see Additional file 4). We further tested the expression of the 20 most abundantly sequenced candidate snoRNAs by Northern blotting (see Additional file 5). Nine of the twenty candidates were also detectable in this assay, while an additional nine C/D box snoRNAs are supported by the ENCODE data (see Additional file 4).

To determine whether the candidates we identified as described are entirely novel snoRNA genes or so far undescribed homologs of known snoRNAs, we performed a BLAST search against the snoRNA genes from snoRNA-LBME-db (requiring an *E*-value ≤ 10^-3^). We further compared the loci of the putative snoRNAs with the snoRNA annotation available in ENSEMBL release 65 [40], which is based on automatic annotation with sequence/structure models available in the Rfam database [41]. Out of the 20 H/ACA box snoRNA candidates, 18 show sequence or structural homology to known snoRNAs, while candidates ZL4 (annotated as nc053 in [42], but not classified as a snoRNA by the authors) and ZL36 appear to be novel H/ACA box snoRNAs without a known homolog. The homology search additionally revealed that ZL4 is conserved until *Xenopus tropicalis*.

Of the 77 C/D box snoRNAs, only seven showed sequence homology to known C/D box snoRNA genes, but in one case (ZL1) the homology consisted solely of a long GU-rich region. The evolutionary conservation of the guide regions of five of these snoRNAs (ZL11, ZL109, ZL126, ZL127 and ZL132) suggests that they target the same nucleotides on ribosomal RNA as their homologs. A sixth snoRNA, ZL142, appears to be a human homolog of the GGN68 snoRNA of chickens [43,44]. An additional comparison with the results of another large snoRNA analysis [45], revealed that ZL2 and ZL107 have been previously described as SNORD41B and Z39, respectively. In order to further characterize the 69 potentially novel C/D box snoRNAs (including ZL1, which only had homology with a known snoRNA in a GU-rich region), we first asked whether their C and D boxes are evolutionarily conserved (Additional file 1). To this end, we computed their average position-wise phastCons scores [46], which we obtained from the UCSC genome browser. Five candidates including ZL1 showed an average phastCons score per nucleotide higher than 0.25 for C and D box nucleotides. A comprehensive homology search of vertebrate genomes allowed us to trace the evolutionary origin of these snoRNAs and to annotate C' and D' boxes as well as putative guide regions based on sequence conservation. ZL1 is highly conserved in vertebrates including *Petromyzon marinus*, while for ZL5, ZL6, ZL8 and ZL24 we were not able to retrieve any homologs outside of mammals.

**Figure 2:**
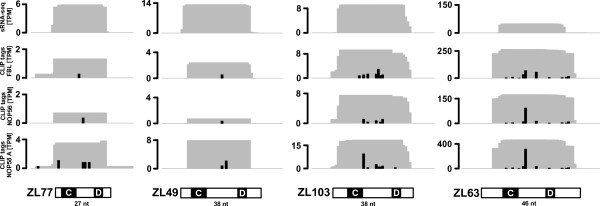
**Small RNA-seq and PAR-CLIP reads mapping to mini-snoRNAs**. Mini-snoRNAs ZL77, ZL49, ZL103 and ZL63 are shown. Black bars in the panels corresponding to PAR-CLIP libraries indicate the number of T→C mutations observed at individual nucleotides. CLIP: cross-linking and immunoprecipitation; PAR-CLIP: photoactivatable-ribonucleoside-enhanced cross-linking and immunoprecipitation; snoRNA: small nucleolar RNA

The remaining C/D box snoRNAs show overall weak conservation in mammals and in primates (Additional file 1). The C' and D' box elements of these snoRNAs, which are typically more variable in sequence, were particularly difficult to annotate without supporting evidence from evolutionary conservation. Because it is not clear that these snoRNAs have a C-D'-C'-D box architecture, we refer to them as C/D box-like. The small RNA sequence data indicates that these C/D box-like snoRNAs are only weakly expressed (Additional file 6). Interestingly, while the shortest C/D box snoRNA that has been characterized so far is SNORD49B, which has 48 nucleotides, 23 of our C/D box-like snoRNAs are even shorter. Figure 2 depicts PAR-CLIP tags and small RNA-seq reads for four of these snoRNAs which we called mini-snoRNAs. ZL77 is among the shortest, with 27 nucleotides in length, and only 7 nucleotides available as a potential guide region between the C and D boxes, while ZL49 and ZL103 are slightly longer (14 and 15 nucleotides between the C and D boxes). Another mini-snoRNA, ZL63, generated a considerable number of reads in all the CLIP libraries as well as in the RNA sequence data.

Our screen could further identify a snoRNA with mixed C/D box and H/ACA box structure. SCARNA21, a computationally predicted H/ACA box snoRNA [47], is surrounded by conserved C and D box elements enclosed by a terminal stem structure (Additional file 7). Northern blot analysis revealed that the prevalent form in the cells is the one that contains the C/D box elements and not the short form, which would be the single H/ACA box snoRNA.

### Target prediction for newly identified snoRNA genes

**Figure 3:**
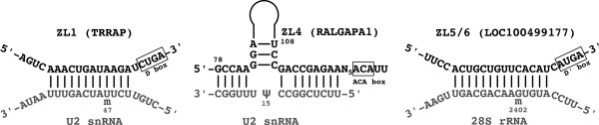
**Predicted structure of hybrids between novel snoRNAs and target RNAs**. The snoRNAs are given at the top of each panel together with the symbol of the host gene in which the snoRNA resides (in parentheses). The targets are indicated at the bottom of the panels. rRNA: ribosomal RNA; snoRNA: small nucleolar RNA; snRNA: small nuclear RNA

To gain insight into the function of the novel snoRNAs that we identified, we sought to determine whether they have canonical targets. We employed the programs RNAsnoop and PLEXY to predict targets of H/ACA box and C/D box snoRNAs, respectively [48,49]. As potential target sequences we considered ribosomal and spliceosomal RNAs obtained from snoRNA-LBME-db. Indeed, for the highly conserved C/D box snoRNAs ZL1, ZL5 and ZL6 (which share the guide region), as well as for the H/ACA box snoRNA ZL4, we could identify canonical targets (Figure 3). ZL1 and ZL4 are both predicted to guide modifications on the U2 snRNA, 2'-O-methylation of U47 and pseudouridylation of U15, respectively. The pseudouridylation of U2 snRNA at U15 has already been described, but the guiding snoRNA was not known [50]. With primer extension assays we could further validate the U47 modification (see Additional File 8). SnRNA modifications are known to occur in Cajal bodies. Consistent with ZL4 H/ACA box snoRNA being a scaRNA that is recruited to Cajal bodies, is the presence of the CAB box motif (UGAG), known to mediate this transport [12], in the hairpin loops. For the C/D box snoRNA ZL1 targeting U2 snRNA we could not identify an H/ACA box-like structural domain with a CAB box. Interestingly, however, this snoRNA candidate contains a long GU repeat, a feature shared by SCARNA9, the only Cajal body-associated snoRNA that lacks H/ACA and CAB boxes. This suggests that the GU element serves as an import signal into Cajal bodies. For ZL5/6, the predicted modification site on the 28S rRNA is in fact a known modification site for which the guide was so far unknown. We could not predict a target for the newly identified C/D box domain of SCARNA21.

We were especially interested to find out whether the non-conserved C/D box-like snoRNAs and in particular the mini-snoRNAs, could guide 2'-O-methylations. To this end, we took a simple approach searching for 8-mer Watson-Crick complementarity between the putative guide regions upstream of the D boxes to ribosomal and spliceosomal RNAs. We did indeed identify seven putative interaction sites, but none of these are known modification sites (Additional file 2). Thus, the targets of these C/D box-like snoRNAs remain to be identified.

### Non-canonical RNA partners of core snoRNA proteins

Although snoRNAs are best known for guiding modifications of rRNAs, snRNAs and tRNAs [1-3], some evidence has emerged for the involvement of full-length mature snoRNAs also in other biological processes such as alternative splicing [51]. To investigate this possibility, we searched our PAR-CLIP data sets for RNAs that were abundantly cross-linked, yet not known to associate with the core snoRNA proteins. In contrast to the HuR PAR-CLIP that we performed before [35], the PAR-CLIP experiments conducted with C/D box snoRNP core proteins repeatedly identified several non-coding RNAs including vault RNA 1-2, 7SK RNA and 7SL RNA as well as H/ACA box snoRNAs. Similarly, in the Dyskerin PAR-CLIP we observed cross-linking of several C/D box snoRNAs.

We performed primer extension experiments to determine potential sites for 2'-O-methyl and pseudouridine modification in prominent ncRNAs such as 7SK RNA, 7SL RNA and vault RNA 1-2 (see Additional file 9 for primer extension assays and Additional file 10 for a catalog of identified modifications sites and target predictions). Indeed, we found that all three of these RNA species carry modifications. Vault RNA 1-2 contains four 2'-O-methyl sites, 7SK RNA carries at least six 2'-O-methyl sites and one pseudouridylation site, and 7SL RNA contains several sites of pseudouridylation. Additionally, we sought to determine whether C/D box and H/ACA box snoRNAs guide modifications on each other. We thus performed 2'-O-methylation primer extension assays on SNORA61 and pseudouridylation assays on SNORD16 and SNORD35A. We found that SNORA61 potentially carries one 2'-O-methylation, while SNORD16 and SNORD35A carry two and six pseudouridylated residues, respectively. To identify C/D box snoRNAs that could guide the observed 2'-O-methylations, we searched for 8-mer complementarity upstream of D and D' boxes of C/D box and C/D box-like snoRNAs, but we did not find sequences complementary to the modification sites. To predict guiding H/ACA box snoRNAs we employed the program RNAsnoop using stringent filtering criteria. We identified potential guiding H/ACA box snoRNAs for 7SK RNA residue Ψ250 and 7SL RNA residue Ψ226.

Previous studies reported that snoRNAs may function in alternative splicing [32,51] and we also repeatedly observed cross-linking of C/D box core proteins to regions that are annotated as exons of protein coding genes. To determine whether these mRNA regions are targeted by snoRNAs, we selected, from the top 1,000 clusters located in mRNA exons in NOP58 libraries, the 157 that were present in both NOP58 replicates and a third CLIP library with at least 10 TPM per nucleotide (Additional file 11). We identified complementarities to the 8-mer guide regions of snoRNAs in 79 of these clusters. In contrast, in shuffled CLIPed regions we only found 60 complementarities to snoRNA guide regions (average of 100 simulations on shuffled sequences). Thus, the mRNA sequences that we isolated in the CLIP experiments are consistent with the possibility that snoRNAs act as guides in some steps of mRNA processing.

### snoRNA processing patterns

**Figure 4:**
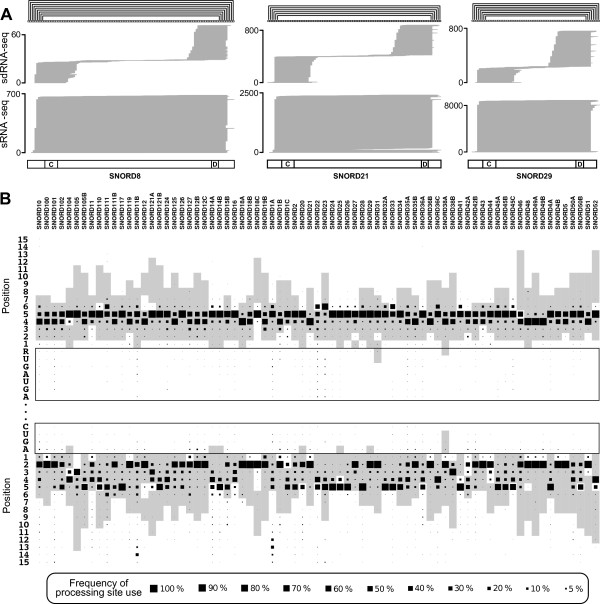
**Terminal processing of C/D box snoRNAs**. **(A) **Profiles of sequencing reads obtained from two small RNA seq libraries for three selected C/D box snoRNAs (SNORD8, SNORD21 and SNORD29). Upper: sdRNA sequencing, 18 to 30 nucleotides. Lower: sRNA sequencing, 20 to 200 nucleotides. Secondary structure annotation of the terminal closing stem is given on the top of the figure, while the locations of C and D motifs are shown on the bottom. **(B) **Detailed analysis of terminal stem processing for C/D box snoRNA expressed in HEK293 cells. The y-axis indicates individual nucleotides, with their specific identity for the nucleotides in C/D boxes and position relative to the boxes for the flanking nucleotides. Each column corresponds to a snoRNA, whose identity is shown at the top of the panel. Grey boxes indicate nucleotides that are predicted to be paired in the terminal stem. The size of black boxes is proportional to the number of sRNA sequencing reads that start (5' end) or end (3' end) at a particular nucleotide. See Additional File 16 for analysis of all C/D box snoRNAs expressed in HEK293 cells. sdRNA: small derived RNA; snoRNA: small nucleolar RNA; sRNA: small RNA

It has become apparent that many ncRNAs such as tRNAs, snRNAs, rRNAs and snoRNAs are extensively processed into small, stable RNA fragments originating mainly from the termini of the mature ncRNA [29], which in some cases are incorporated in the Argonaute proteins to function as microRNAs [24]. To identify snoRNA-derived small RNAs that could potentially act as miRNAs comprehensively, we isolated and sequenced the RNA fraction of 18 to 30 nucleotides from HEK293 cells. Small RNAs derived from C/D box snoRNAs constitute about 1.7% of the small RNA pool in this size range in HEK293 cells (Table 3). Consistent with the results of Li and colleagues [29], we found that most of the 513,339 reads overlapping with C/D box snoRNA genes originate from the 5' or 3' ends (38.7% and 46.0%, respectively). Visual inspection of the alignment of these reads to the snoRNAs revealed, however, that start and end positions of the reads do not generally coincide with the annotated snoRNA termini, which were inferred based on the characteristic C/D box snoRNA terminal stem (Figure 4A). Instead, the reads that we obtained indicate specific trimming that generates sharp 5' ends for 5'-end-derived reads and sharp 3' ends for 3'-end-derived reads. To determine whether this trimming may occur in the process of generating small RNAs from mature C/D box snoRNAs, we isolated small RNAs of length 20 to 200 nucleotides that presumably included the full-length, mature snoRNAs (average C/D box snoRNA length is 70 to 90 nucleotides) and performed a 150-cycle sequencing run. Figure 4A depicts the alignment of reads obtained in the small RNA fraction and the reads obtained in the 150-cycle sequencing run for three selected C/D box snoRNAs. Strikingly, the sharp ends of C/D box snoRNA-derived small RNAs coincide with the 5' and 3' ends of the mature form. More generally, we found that for 84% and 70% of the top 50 expressed C/D box snoRNAs, the most prominent start and end positions, respectively, obtained from long sequencing reads coincided with the most prominent start and end positions obtained from small RNA sequencing. This suggests that the observed trimming of the terminal closing stem occurs during the excision of the snoRNA from the intron and is not specific to the processing of the mature snoRNA form into smaller fragments. Furthermore, we found that it is the distance to the C or D boxes that seems to determine the observed ends of the snoRNAs rather than the length of the terminal closing stem (Figure 4B). The 5' end is sharply defined four to five nucleotides upstream of the C box, while the 3' end is more variably located two to five nucleotides downstream of the D box. In most cases this will leave mature C/D box snoRNAs with a terminal 5' overhang compared to the 3' end. This suggests that, similar to other small RNAs [52,53], snoRNAs are trimmed presumably by exonucleases, to boundaries that are determined by the proteins with which these small RNAs are complexed.

**Table 3:** Functional annotation of sequencing reads obtained in sRNA sequencing and HeLa Ago2 IP sequencing.

RNA class	HEK293 sRNA sequencing (18 to 30 nucleotides)	HeLa Ago2 immunoprecipitation sequencing (asynchronous cells)	HeLa Ago2 immunoprecipitation sequencing (mitotic cells)
microRNAs	18.304%	89.750%	82.237%
tRNAs	9.694%	0.204%	0.298%
snRNAs	5.275%	0.029%	0.071%
C/D box snoRNAs	1.751%	0.005%	0.054%
H/ACA box snoRNAs	0.318%	0.026%	0.046%
No annotation	64.658%	9.985%	17.293%

Ago2: Argonaute 2; IP: immunoprecipitation; snRNA: small nuclear RNA; snoRNA: small nucleolar RNA; sRNA: small RNA; tRNA: transfer RNA

Small RNAs derived from C/D box snoRNA termini appear to be abundant in the cells, and can be incorporated into Argonaute proteins to act as miRNAs [31]. To determine the relative participation of various small RNA classes in the Argonaute-dependent gene silencing, we immunopurified Ago2 from HeLa cells and sequenced the associated small RNA fraction. We found that, as expected, miRNAs constitute the most abundant RNA class that associates with Ago2 (approximately 90%), while C/D box snoRNAs account only for 0.005% of the IP-seq reads (Table 3). Assuming that overall proportions of small RNAs derived from tRNAs and snoRNAs are fairly constant across cell types, we can estimate the efficiency with which small RNAs (from the total small RNA pool) are incorporated in the Argonaute proteins. We found, for example, that although small RNAs derived from tRNAs are 5.6 times more abundant than C/D box derived snoRNAs, tRNA fragments are 40 times more abundant in the Ago2-associated fraction. Thus, tRNA-derived small RNAs appear to be more efficiently incorporated in Ago2 than C/D box snoRNA fragments. This is consistent with observations that tRNAs are cleaved by nucleases such as Angiogenin and even Dicer to generate processing fragments that are active in translation regulation [54,55]. Similarly, small RNAs derived from H/ACA box snoRNAs are 5.5 times less abundant than small RNAs derived from C/D box snoRNAs in the total RNA fraction, but are 5.2 times more efficiently picked up by Ago2. The H/ACA box snoRNA SCARNA15, which has been shown to be processed into smaller fragments that act as microRNAs [24], is represented in this library with 3,636 reads, 29% of all reads mapped to H/ACA box snoRNA loci (see Additional file 12 for a full listing of all snoRNAs). The C/D box snoRNA with the highest number of reads in the Ago2 IP library is SNORD1A with 1,140 reads, but the majority of C/D box snoRNAs are represented by less than 50 reads.

Of all categories of small RNAs, C/D box snoRNA fragments are those that show the strongest nuclear retention, and are found in the cytoplasm with only low frequency [56]. Thus, this physical separation could account for the low frequency of association between C/D box snoRNA-derived RNAs and Ago2. We therefore wondered whether the association of this abundant class of RNA fragments with Ago2 increases in the mitotic phase of the cell cycle, when the nuclear membrane is dissolved. We collected HeLa cells that were in the mitotic phase through mitotic shake off, immunopurified Ago2 and again sequenced the Ago2-associated small RNA fraction. We found that, indeed, the relative abundance of C/D box-derived fragments in Argonaute increased in this condition (Table 3), to 0.054% relative to 0.005%. Nonetheless, these results indicate that C/D box snoRNAs do not generally carry out miRNA-like functions, and that the number of H/ACA box snoRNAs with a dual function is very limited.

## Discussion

To gain insight into the processing of snoRNAs and the functions of snoRNA-derived small RNAs, we performed PAR-CLIP experiments with snoRNP core proteins. Analysis of PAR-CLIP reads showed that C/D box core proteins Fibrillarin, NOP56 and NOP58 have a very similar binding pattern, overlapping with the box elements. Excluding snoRNA families SNORD113 to SNORD116, which are multi-copy families and do not have guide complementarity to rRNAs or snRNAs, snoRNA-LBME-db currently lists 153 C/D box snoRNAs, of which 40 and 78 have a guide region targeting a known modification at the D box and D' box, respectively. Evolutionary conservation profiles of the remaining putative guide regions suggest that most of them are not functional. In support of this concept, our analysis revealed that C/D box core proteins cross-linked more effectively to guide regions that are known to have a target compared to orphan guide regions.

Combining computational prediction with data from small RNA sequencing and PAR-CLIP we identified novel C/D and H/ACA box snoRNAs, and assigned guiding snoRNAs to several modifications on rRNAs and snRNAs that were previously described as orphans. In addition to these *bona fide *snoRNAs, we uncovered a group of C/D box-like snoRNAs that only have a C and a D box as opposed to the common C-D'-C'-D architecture. These C/D box-like snoRNAs are only weakly conserved and most of them are expressed at low levels. The unusual architecture and the weak evolutionary conservation are likely reasons why these RNA species have not been uncovered by computational ncRNA gene finders [57]. Some of the identified C/D box-like snoRNAs are extremely short, one being only 27 nucleotides in length, leaving hardly enough space for a guide region. The requirements for C/D box snoRNA biogenesis appear to be simply the presence of C and D boxes and a short region of complementarity flanking these boxes, leading probably to the production of many snoRNA-like molecules as the C/D box core proteins scan intronic regions of pre-mRNAs. An interesting lead to follow in further investigating the potential function of the C/D box-like snoRNAs originating in the introns of many genes comes from a recent study conducted in *Drosophila*, in which Schubert and colleagues showed that snoRNAs are required for maintenance of higher-order structures of chromatin accessibility [58].

In our PAR-CLIP experiments we also repeatedly cross-linked ncRNAs that are not usual snoRNA targets. We observed H/ACA box snoRNAs in PAR-CLIP experiments targeting the C/D box core proteins. *Vice versa*, we found C/D box snoRNAs in the PAR-CLIP targeting Dyskerin, which is an essential component of H/ACA box snoRNPs. Primer extension assays indicated that these snoRNAs carry modifications that would be expected from the protein complexes to which they were cross-linked, but we were, in general, not able to identify snoRNAs that could guide these modifications. One drawback may be that in the case of the 2'-O-methyl primer extension assays we cannot be sure that it was indeed a 2'-O-methyl modification as opposed to any other nucleoside modification that caused the stoppage of the reverse transcriptase. However, we can be fairly certain that we identified *bona fide *pseudouridylation sites. Particularly, in the case of SNORD35A we were able to identify five putative pseudouridylated residues but no convincing guiding sequence in a known H/ACA box snoRNA. This suggests either that even more snoRNAs remain to be identified or that these pseudouridylations are caused by a protein-only mechanism not requiring guidance by H/ACA box snoRNAs.

The processing patterns of snoRNAs have raised substantial interest and some controversy in recent years [30,32,59]. We strikingly found that snoRNA excision out of the intron follows a well-defined pattern leaving mature snoRNAs with four to five nucleotides upstream of the C box, and two to five nucleotides downstream of the D box, irrespective of the length of the terminal closing stem. Our data support the observations of Darzacq and Kiss [5] that the terminal stem serves to bring the C and D box elements into close proximity so as to be more easily recognized by snoRNP proteins, which then protect the snoRNA from further trimming by the exosome, but may not be needed for the functional, mature snoRNA. This implies that the core proteins actively protect and stabilize the maturing snoRNA.

We further quantified the abundance of snoRNA-derived small RNAs in HEK293 cells, and consistent with other studies [29], we found that small RNAs derived from the ends of C/D box snoRNAs are indeed abundant. However, we did not find evidence that these sdRNAs efficiently associate with Ago2 to act as microRNAs, even in conditions when the accessibility of these sdRNAs to Ago2 should be higher, such as in mitotic cells. We thus conclude that a microRNA-like function of snoRNA-derived small RNAs is an exception rather than a rule. Most of the sdRNAs from C/D box snoRNAs originate from the termini of mature snoRNAs, and hence carry C and D box motifs. It might be that snoRNA core proteins are still attached to these fragments, protect them from total degradation, sequester them in the nucleus and prevent these sdRNAs from being loaded into Ago2.

Deep-sequencing-based studies revealed a very complex landscape of transcription and processing of RNAs. The non-canonical products identified initially in such studies raises the question of additional, yet unknown, functions of molecules that have been studied for many years. What has become apparent more recently, however, is that deep sequencing allows us to construct a very detailed picture of the kinetics of processing various classes of RNAs and of their interactions with proteins that protect them from degradation. Intersection of many data sets such as those generated in our study will eventually reveal kinetic and regulatory aspects of cellular processes at a fine level of detail.

## Materials and methods

### PAR-CLIP experiments

PAR-CLIP was performed with HEK293 Flp-In cells (Invitrogen). Cells were grown in thirty 15-cm cell culture plates per experiment to approximately 80% confluency. At 12 h before harvest, 4-thiouridine (Sigma) was added to the cells to a final concentration of 100 µM. PAR-CLIP was carried out as described previously [34]. For immunoprecipitation, antibodies were coupled to protein-A or protein-G Dynabeads (Invitrogen). Antibodies used against endogenous proteins were α-NOP58 (sc-23705 from Santa Cruz Biotechnology), α-Dyskerin H-300 (sc-48794, Santa Cruz Biotechnology), α-Dyskerin C-15 (sc-26982, Santa Cruz Biotechnology) and α-Fibrillarin AFB01 monoclonal antibody line 72B9, lot 011 (from Cytoskeleton, Inc, AFB01). The α-Ago2 (11A9) monoclonal antibody was a gift from Gunter Meister. For PAR-CLIP with NOP56 we used a HEK293 cell line with a stably integrated FLAG-NOP56 fusion gene and IP was done with monoclonal α-FLAG antibody M2 from Sigma. For one Fibrillarin targeted PAR-CLIP the immunoprecipitated complexes were treated with micrococcal nuclease (MNase, from New England Biolabs) for 5 min at 37°C [35]. After SDS-PAGE, gels were blotted onto nitrocellulose membranes to reduce the background from free RNAs [60]. The PAR-CLIP libraries were prepared as described in Additional file 13 and submitted to deep sequencing on an Illumina HiSeq 2000.

The reads obtained from PAR-CLIP experiments were mapped to the human genome (hg19 assembly from UCSC, February 2009) and annotated with the CLIPZ server [36]. Reads marked with the CLIPZ annotation categories 'fungal', 'bacterial,' or 'vector' were discarded and only reads that mapped uniquely to the genome were used in the analyses. The library size was scaled to 1,000,000 for all samples to obtain a normalized expression value (tags per million).

### Small RNA sequencing

Small RNA sequencing libraries were prepared from size-selected RNAs of 18 to 30 nucleotides (sdRNA sequencing) and 20 to 200 nucleotides (sRNA sequencing). HEK293 total RNA was extracted and treated with DNase. Next, 20 units of T4 polynucleotide kinase and 2 µl of [γ-32P] ATP (10 µCi/µl) were used to radiolabel 10 µg of RNA at the 5'-ends. The RNA was separated together with a radiolabeled 20-nucleotide ladder on a 12% polyacrylamide gel, the bands corresponding to 18 to 30 nucleotides (for sdRNA sequencing libraries) or 20 to 200 nucleotides (for sRNA sequencing libraries) were excised, the RNA was extracted overnight in a 0.4-M NaCl solution and finally precipitated with ethanol. Small RNA libraries were prepared according to a published protocol [61] and sequenced on an Illumina HiSeq 2000 instrument, for 36 (sdRNA sequencing) and 150 cycles (sRNA sequenicng library). Adaptor removal was done with the CLIPZ server, and the mapping to the human genome was then done with the Segemehl software (v. 0.1.3) with parameters '-D 1 -A 90' [62]. The Gene Expression Omnibus (GEO) accession number for the PAR-CLIP and sRNA-seq data is GSE43666.

### Identification of novel C/D snoRNAs and H/ACA snoRNAs from PAR-CLIP and small RNA sequencing data

For each PAR-CLIP library we inferred binding regions of the proteins of interest by clustering reads whose corresponding loci were at most 25 nucleotides apart. To annotate known snoRNA and scaRNA genes we first retrieved sequences from the snoRNA-LBME-db [37], mapped them to the human genome (a list of motif and secondary structure annotated snoRNAs is available in Additional file 13). The 500 binding regions that accumulated the highest number of reads in each individual CLIP library, but did not overlap with known snoRNA or scaRNA genes, ncRNA genes or repeat elements, were screened for novel snoRNA candidates. We used SnoReport [38] to detect H/ACA box snoRNAs, while for detection of C/D box snoRNAs we searched for protein-binding regions that contained motifs corresponding to the C box (RTGATGA; allowing one mismatch) and to the two most common D box motifs (CTGA and ATGA). Sequences that contained both a C box and a D box motif were extended by ten nucleotides in order to search for a terminal closing stem. If a compact closing stem composed of at least four canonical base pairs with at least two G-C/C-G base pairs was found, the sequence was considered a snoRNA candidate. To evaluate the specificity of our C/D box snoRNA gene finding approach, we applied the same procedure to two types of clusters of PAR-CLIP reads from the NOP58 rep A sample both extended by 25 nucleotides on each side. First were the top 100 clusters (defined in terms of the number of reads associated with the cluster) that overlapped with C/D box snoRNA annotation, which served as a positive control. In this set, our program reported 80 sequences as putative snoRNAs. The second type of cluster contained the top 100 clusters that overlap with mRNA exon annotation. These should not contain snoRNAs, and indeed, we only obtained five putative C/D box snoRNAs candidates. Similarly low numbers of snoRNA candidates were obtained from randomized sequences (not shown). Altogether, these tests indicated that our method has very good specificity. In contrast, the number of predictions we obtained from CLIPed clusters without a known annotation was 11 for the top 100 such clusters.

Candidates that showed expression of at least 1 TPM per nucleotide in the 20 to 200 nucleotides small RNA sequencing run (only uniquely mapped reads that covered at least 50% of the candidate snoRNA sequence were considered), and had at least 1 TPM per nucleotide in at least one of the type-specific CLIP libraries were considered putative snoRNAs. They were consecutively numbered, and named as 'ZL#'. To further validate the newly found snoRNAs, we searched for evidence of expression in recently published small RNA-seq libraries from the ENCODE project [39]. Files with the genome coordinates of mapped reads (BAM files) were obtained from the ENCODE data coordination center at UCSC [63] and uniquely mapping reads were used for the analysis. In addition, we selected the 20 candidate C/D box snoRNAs with the highest read count in our data for validation by Northern blotting (see Additional file 13 for details on the experiment). To evaluate the evolutionary conservation of the putative snoRNAs, we carried out a homology search against the vertebrate genomes available in the UCSC genome browser. Once an initial set of homologs was identified, we built sequence/structure models and continued to search for more distant homologs with the Infernal software [64].

### Detection of 2'-O-ribose-methylated and pseudouridylated residues

To identify 2'-O-methylated residues we used a reverse transcriptase-based method coupled with polyacrylamide gel analysis as described in [65]. The method is based on the observation that cDNA synthesis is noticeably impaired in the presence of a 2'-O-methyl when deoxynucleotide triphosphate fragments (dNTPs) are limiting [65,66], giving rise to a characteristic pattern of gel banding immediately preceding the 2'-O-methyls, with strong bands at low dNTP concentrations (0.004 mM) [66], becoming weaker with increasing concentrations of dNTPs.

To map pseudouridines in candidate RNAs we used a method that relies on chemical modification of RNA bases with N-cyclohexyl-N'-β (4-methyl morpholinium) -ethylcarbodiimide (CMC) [67]. The method involves carbodiimide adduct formation with U, G and pseudouridine followed by mild alkali treatment, which removes the adduct from U and G but not from the N-3 of pseudouridine. This modification results in the blockage of reverse transcription one residue 3' of the pseudouridine on the sequencing gel. For a detailed description of assays used to map 2'-O-methyls and pseudouridines see Additional file 13. As a proof of principle, we first applied these assays to the spliceosomal RNA U6, which is known to carry 2'-O-methylated and pseudouridylidated residues. In addition to the well-documented sites, we also observed novel 2'-O-methyl sites that have not been previously reported so far (Additional file 14).

To predict C/D box snoRNAs that could guide 2'-O-methylation, we searched for 8-mer complementarity (only canonical base pairs allowed) to regions immediately or one nucleotide upstream of the D and D' boxes of C/D box and C/D box-like snoRNAs. To predict H/ACA box snoRNAs that could guide pseudouridylations, we used the program RNAsnoop [48]. We first determined for each H/ACA snoRNA stem an energy cutoff value by running simulations on 1,000 random sequences of length 100. Only if an RNAsnoop prediction had an energy value lower than 90% of the random sequences, and at least three canonical base pairs on each side of the binding pocket, did we consider it as a hit.

### Ago2 immunoprecipitation sequencing of asynchronous and mitotic cells

Mitotic cells were collected using mitotic shake-off [68,69], a technique based on the observation that cells become rounded and more easily detachable from the culture vessel as they progress into metaphase during mitosis [70]. Details of the experimental setup are given in Additional file 13. To be able to confirm microscopically that we collected mitotic cells we used HeLa cells with the human histone H2B gene fused to green fluorescent protein (see Additional file 15).

Ago2 was immunoprecipitated from mitotic and asynchronous cells; the Ago2-associated RNAs were extracted and used to prepare cDNA libraries as described above [61], which were then submitted to deep sequencing. Adaptor removal was with the CLIPZ server, and reads were then mapped with Segemehl as described above. In the analysis of small RNA libraries (Ago2-IP and HEK293 sdRNA sequencing (18 to 30 nucleotides)), we considered both uniquely and multi-mapping reads that were annotated based on their mapping to genes in one of the following categories: tRNAs (from the UCSC Table Browser), microRNAs (from mirBase) and snRNAs (from ENSEMBL release 59), C/D box snoRNAs and H/ACA box snoRNAs (curated data set from this work).

## Abbreviations

Ago2: Argonaute 2; CB: Cajal body; CLIP: cross-linking and immunoprecipitation; DKC1: Dyskerin; dNTP: deoxynucleotide triphosphate; FBL: Fibrillarin; IP: immunoprecipitation; IP-seq: immunoprecipitation sequencing; miRNA: micro RNA; MNase: micrococcal nuclease; ncRNA: non-coding RNA; PAR-CLIP: photoactivatable-ribonucleoside-enhanced cross-linking and immunoprecipitation; rRNA: ribosomal RNA; scaRNA: small Cajal body-specific RNA; sdRNA: small derived RNA; snoRNA: small nucleolar RNA; snoRNP: small nucleolar ribonucleoprotein; snRNA: small nuclear RNA; sRNA: small RNA; TPM: tags per million; tRNA: transfer RNA.

## Authors' contributions

SK and MZ conceived the project. SK performed the experiments, with help from DJJ (Ago2 IP and primer extensions) and APS (novel snoRNA validation). ARG performed the computational analysis of the sequencing data, with help from HJ (computational prediction of snoRNA targets). ARG, DJJ, SK and MZ wrote the manuscript. All authors read and approved the final manuscript.

## Supplementary Material

Additional file 1 **Profiles of PAR-CLIPs reads obtained with various core snoRNP proteins for snoRNAs and scaRNAs**. The proteins and normalized read counts are shown on the y-axis. The snoRNA and location of boxes are shown at the bottom. Red bars in the profiles indicate the number of T→C mutations observed at individual nucleotides in the PAR-CLIP reads. Click here for file

Additional file 2 **List of novel C/D box, C/D box-like snoRNAs and mini-snoRNAs obtained in this study**. Click here for file

Additional file 3 **List of novel H/ACA snoRNAs or homologs of known snoRNAs (indicated in the 'BLAST hits' column) that were obtained in this study**. Click here for file

Additional file 4 **RNA-seq read profiles from selected ENCODE small RNA-seq samples along the novel C/D box and H/ACA box snoRNA loci identified in our study**. Click here for file

Additional file 5 **Northern blots for selected novel C/D box snoRNAs**. Among the 20 most abundantly expressed (in the small RNA-seq data) novel C/D box snoRNAs we could confirm the presence of ZL1, ZL2, ZL8, ZL11, ZL63, ZL107, ZL116, ZL126 and ZL127 by Northern blotting. Click here for file

Additional file 6 **Expression of C/D box and C/D box-like snoRNAs in our small RNA-seq run (20 to 200 nucleotides; sequenced 150 cycles)**. Only reads that cover at least 50% of the snoRNA locus were considered. Click here for file

Additional file 7 **SCARNA21 has a C/D box H/ACA box hybrid structure. (A) **Screenshot from the UCSC genome browser showing conserved C and D box elements. **(B) **Northern blot probing for H/ACA box structure only (left) and for the hybrid structure (right). Click here for file

Additional file 8 **Primer extension assays for U2 snRNA**. Primer extension assay reveals a 2'-O-methyl modification site for nucleotide U47. Click here for file

Additional file 9 **Primer extension assays for non-canonical snoRNA targets**. Primer extension runs reveal 2'-O-methyl (A-C) and pseudouridine (D-G) modification sites in several non-canonical RNAs. **(A) **SNORA61: G50. **(B) **VTRNA1-2: G30, U31, C33, A34. **(C) **7SK RNA: C137, G139, C141, G148, C150, G151. **(D) **SNORD16: U52, U55. **(E) **SNORD35A: U26, U31, U37, U43, U45, U51. **(F) **7SK RNA: U250. **(G) **7SL RNA: U226, U233, U236, U266, U273. Click here for file

Additional file 10 **Summary of nucleotide modifications detected by primer extension assays and predicted guide snoRNA-target interactions**. Click here for file

Additional file 11 **Analysis of PAR-CLIP clusters overlapping with mRNA exon annotation**. Shown are genome coordinates, host transcript and exon identifier, the number of C and D boxes predicted within the genomic region, snoRNAs to whose guide regions these mRNA fragments are complementary and the number of (normalized) reads obtained from the regions in various PAR-CLIP libraries. Click here for file

Additional file 12 **Detailed list of reads mapping to snoRNA loci in Ago2 IP-seq libraries**. Click here for file

Additional file 13 **Supplementary materials and methods**. Detailed information about the experimental methods (PAR-CLIP library preparation, Northern blotting, primer extension assays, mitotic shake-off and Ago2 immunoprecipitation and sequencing). In addition, the annotated C/D and H/ACA snoRNAs used in this study are listed. Click here for file

Additional file 14 **Primer extension assays on spliceosomal RNA U6. (A) **Primer extension assay on spliceosomal RNA U6 detected documented 2'-O-methylation as well as potentially novel 2'-O-methylation sites. **(B) **Primer extension assay detected documented pseudouridine sites in U6. CTRL indicates the untreated sample, +CMC the sample treated with 1-cyclohexyl-3-(2-morpholinoethyl)carbodiimide metho-p-toluenesulfonate (CMC). Click here for file

Additional file 15 **Asynchronous and mitotic GFP-tagged HeLa cells**. Green fluorescent protein appears in green and cell boundaries in orange. **(A) **In an asynchronous cell culture only a few cells are in the mitotic phase, which can be seen from the condensed chromatin and the rounded cell morphology. **(B) **Cell obtained with mitotic shake-off. The procedure enriches for round cells containing condensed chromatin. Click here for file

Additional file 16 **Extended version of Figure 4B showing all snoRNA genes expressed in HEK293 cells**. Click here for file

## References

[B1] Decatur W Fournier M rRNA modifications and ribosome function. Trends Biochem Sci 2002 14 344 51 10.1016/S0968-0004(02)02109-6 12114023

[B2] Darzacq X Jády B Verheggen C Kiss A Bertrand E Kiss T Cajal body-specific small nuclear RNAs: a novel class of 2'-O-methylation and pseudouridylation guide RNAs. EMBO J 2002 14 2746 56 10.1093/emboj/21.11.2746 12032087 PMC126017

[B3] Clouet d'Orval B Bortolin ML Gaspin C Bachellerie JP Box C/D RNA guides for the ribose methylation of archaeal tRNAs. The tRNATrp intron guides the formation of two ribose-methylated nucleosides in the mature tRNATrp. Nucleic Acids Res 2001 14 4518 29 10.1093/nar/29.22.4518 11713301 PMC92551

[B4] Tollervey D Kiss T Function and synthesis of small nucleolar RNAs. Curr Opin Cell Biol 1997 14 337 42 10.1016/S0955-0674(97)80005-1 9159079

[B5] Darzacq X Kiss T Processing of intron-encoded box C/D small nucleolar RNAs lacking a 5',3'-terminal stem structure. Mol Cell Biol 2000 14 4522 31 10.1128/MCB.20.13.4522-4531.2000 10848579 PMC85834

[B6] Brown JW Echeverria M Qu LH Plant snoRNAs: functional evolution and new modes of gene expression. Trends Plant Sci 2003 14 42 9 12523999 10.1016/s1360-1385(02)00007-9

[B7] Kiss T Small nucleolar RNA-guided post-transcriptional modification of cellular RNAs. EMBO J 2001 14 3617 22 10.1093/emboj/20.14.3617 11447102 PMC125535

[B8] McKeegan KS Debieux CM Boulon S Bertrand E Watkins NJ A dynamic scaffold of pre-snoRNP factors facilitates human box C/D snoRNP assembly. Mol Cell Biol 2007 14 6782 93 10.1128/MCB.01097-07 17636026 PMC2099223

[B9] Tollervey D Lehtonen H Jansen R Kern H Hurt EC Temperature-sensitive mutations demonstrate roles for yeast fibrillarin in pre-rRNA processing, pre-rRNA methylation, and ribosome assembly. Cell 1993 14 443 57 10.1016/0092-8674(93)90120-F 8431947

[B10] Kiss T Fayet-Lebaron E Jády BE Box H/ACA small ribonucleoproteins. Mol Cell 2010 14 597 606 10.1016/j.molcel.2010.01.032 20227365

[B11] Lafontaine DL Bousquet-Antonelli C Henry Y Caizergues-Ferrer M Tollervey D The box H + ACA snoRNAs carry Cbf5p, the putative rRNA pseudouridine synthase. Genes Dev 1998 14 527 37 10.1101/gad.12.4.527 9472021 PMC316522

[B12] Richard P Darzacq X Bertrand E Jády BE Verheggen C Kiss T A common sequence motif determines the Cajal body-specific localization of box H/ACA scaRNAs. EMBO J 2003 14 4283 93 10.1093/emboj/cdg394 12912925 PMC175784

[B13] Nicoloso M Qu LH Michot B Bachellerie JP Intron-encoded, antisense small nucleolar RNAs: the characterization of nine novel species points to their direct role as guides for the 2'-O-ribose methylation of rRNAs. J Mol Biol 1996 14 178 95 10.1006/jmbi.1996.0391 8764399

[B14] Kiss-László Z Henry Y Bachellerie JP Caizergues-Ferrer M Kiss T Site-specific ribose methylation of preribosomal RNA: a novel function for small nucleolar RNAs. Cell 1996 14 1077 88 10.1016/S0092-8674(00)81308-2 8674114

[B15] Cavaillé J Nicoloso M Bachellerie JP Targeted ribose methylation of RNA in vivo directed by tailored antisense RNA guides. Nature 1996 14 732 5 10.1038/383732a0 8878486

[B16] Ganot P Bortolin ML Kiss T Site-specific pseudouridine formation in preribosomal RNA is guided by small nucleolar RNAs. Cell 1997 14 799 809 10.1016/S0092-8674(00)80263-9 9182768

[B17] Bortolin ML Ganot P Kiss T Elements essential for accumulation and function of small nucleolar RNAs directing site-specific pseudouridylation of ribosomal RNAs. EMBO J 1999 14 457 69 10.1093/emboj/18.2.457 9889201 PMC1171139

[B18] Lee Y Shibata Y Malhotra A Dutta A A novel class of small RNAs: tRNA-derived RNA fragments (tRFs). Genes Dev 2009 14 2639 49 10.1101/gad.1837609 19933153 PMC2779758

[B19] Haussecker D Huang Y Lau A Parameswaran P Fire A Kay M Human tRNA-derived small RNAs in the global regulation of RNA silencing. RNA 2010 14 673 95 10.1261/rna.2000810 20181738 PMC2844617

[B20] Nicolas F Hall A Csorba T Turnbull C Dalmay T Biogenesis of Y RNA-derived small RNAs is independent of the microRNA pathway. FEBS Lett 2012 14 1226 30 10.1016/j.febslet.2012.03.026 22575660

[B21] Persson H Kvist A Vallon-Christersson J Medstrand P Borg A Rovira C The non-coding RNA of the multidrug resistance-linked vault particle encodes multiple regulatory small RNAs. Nat Cell Biol 2009 14 1268 71 10.1038/ncb1972 19749744

[B22] Zywicki M Bakowska-Zywicka K Polacek N Revealing stable processing products from ribosome-associated small RNAs by deep-sequencing data analysis. Nucleic Acids Res 2012 14 4013 24 10.1093/nar/gks020 22266655 PMC3351166

[B23] Kawaji H Nakamura M Takahashi Y Sandelin A Katayama S Fukuda S Daub C Kai C Kawai J Yasuda J Carninci P Hayashizaki Y Hidden layers of human small RNAs. BMC Genomics 2008 14 157 10.1186/1471-2164-9-157 18402656 PMC2359750

[B24] Ender C Krek A Friedlander M Beitzinger M Weinmann L Chen W Pfeffer S Rajewsky N Meister G A human snoRNA with microRNA-like functions. Mol Cell 2008 14 519 28 10.1016/j.molcel.2008.10.017 19026782

[B25] Taft R Glazov E Lassmann T Hayashizaki Y Carninci P Mattick J Small RNAs derived from snoRNAs. RNA 2009 14 1233 40 10.1261/rna.1528909 19474147 PMC2704076

[B26] Shen M Eyras E Wu J Khanna A Josiah S Rederstorff M Zhang M Stamm S Direct cloning of double-stranded RNAs from RNase protection analysis reveals processing patterns of C/D box snoRNAs and provides evidence for widespread antisense transcript expression. Nucleic Acids Res 2011 14 9720 30 10.1093/nar/gkr684 21880592 PMC3239178

[B27] Jung C Hansen M Makunin I Korbie D Mattick J Identification of novel non-coding RNAs using profiles of short sequence reads from next generation sequencing data. BMC Genomics 2010 14 77 10.1186/1471-2164-11-77 20113528 PMC2825236

[B28] Langenberger D Pundhir S Ekstrøm C Stadler P Hoffmann S Gorodkin J deepBlockAlign: a tool for aligning RNA-seq profiles of read block patterns. Bioinformatics 2012 14 17 24 10.1093/bioinformatics/btr598 22053076 PMC3244762

[B29] Li Z Ender C Meister G Moore P Chang Y John B Extensive terminal and asymmetric processing of small RNAs from rRNAs, snoRNAs, snRNAs, and tRNAs. Nucleic Acids Res 2012 14 6787 99 10.1093/nar/gks307 22492706 PMC3413118

[B30] Scott M Ono M Yamada K Endo A Barton G Lamond A Human box C/D snoRNA processing conservation across multiple cell types. Nucleic Acids Res 2012 14 3676 88 10.1093/nar/gkr1233 22199253 PMC3333852

[B31] Li W Saraiya A Wang C The profile of snoRNA-derived microRNAs that regulate expression of variant surface proteins in *Giardia lamblia*. Cell Microbiol 2012 14 1455 73 10.1111/j.1462-5822.2012.01811.x 22568619 PMC3422372

[B32] Kishore S Khanna A Zhang Z Hui J Balwierz P Stefan M Beach C Nicholls R Zavolan M Stamm S The snoRNA MBII-52 (SNORD 115) is processed into smaller RNAs and regulates alternative splicing. Hum Mol Genet 2010 14 1153 64 10.1093/hmg/ddp585 20053671 PMC2838533

[B33] Brameier M Herwig A Reinhardt R Walter L Gruber J Human box C/D snoRNAs with miRNA like functions: expanding the range of regulatory RNAs. Nucleic Acids Res 2011 14 675 86 10.1093/nar/gkq776 20846955 PMC3025573

[B34] Hafner M Landthaler M Burger L Khorshid M Hausser J Berninger P Rothballer A Ascano M Jr Jungkamp A Munschauer M Ulrich A Wardle G Dewell S Zavolan M Tuschl T Transcriptome-wide identification of RNA-binding protein and microRNA target sites by PAR-CLIP. Cell 2010 14 129 41 10.1016/j.cell.2010.03.009 20371350 PMC2861495

[B35] Kishore S Jaskiewicz L Burger L Hausser J Khorshid M Zavolan M A quantitative analysis of CLIP methods for identifying binding sites of RNA-binding proteins. Nat Methods 2011 14 559 64 10.1038/nmeth.1608 21572407

[B36] Khorshid M Rodak C Zavolan M CLIPZ: a database and analysis environment for experimentally determined binding sites of RNA-binding proteins. Nucleic Acids Res 2011 14 D245 52 10.1093/nar/gkq940 21087992 PMC3013791

[B37] Lestrade L Weber M snoRNA-LBME-db, a comprehensive database of human H/ACA and C/D box snoRNAs. Nucleic Acids Res 2006 14 D158 62 10.1093/nar/gkj002 16381836 PMC1347365

[B38] Hertel J Hofacker I Stadler P SnoReport: computational identification of snoRNAs with unknown targets. Bioinformatics 2008 14 158 64 10.1093/bioinformatics/btm464 17895272

[B39] Djebali S Davis CA Merkel A Dobin A Lassmann T Mortazavi A Tanzer A Lagarde J Lin W Schlesinger F Xue C Marinov GK Khatun J Williams BA Zaleski C Rozowsky J Roder M Kokocinski F Abdelhamid RF Alioto T Antoshechkin I Baer MT Bar NS Batut P Bell K Bell I Chakrabortty S Chen X Chrast J Curado J Landscape of transcription in human cells. Nature 2012 14 101 8 10.1038/nature11233 22955620 PMC3684276

[B40] ENSEMBL release 65. http://www.ensembl.org

[B41] Burge S Daub J Eberhardt R Tate J Barquist L Nawrocki E Eddy S Gardner P Bateman A Rfam 11.0: 10 years of RNA families. Nucleic Acids Res 2013 14 D226 32 10.1093/nar/gks1005 23125362 PMC3531072

[B42] Yan D He D He S Chen X Fan Z Chen R Identification and analysis of intermediate size noncoding RNAs in the human fetal brain. PLoS One 2011 14 e21652 10.1371/journal.pone.0021652 21789175 PMC3138756

[B43] Zhang Y Wang J Huang S Zhu X Liu J Yang N Song D Wu R Deng W Skogerbo G Wang XJ Chen R Zhu D Systematic identification and characterization of chicken (*Gallus gallus*) ncRNAs. Nucleic Acids Res 2009 14 6562 74 10.1093/nar/gkp704 19720738 PMC2770669

[B44] Marz M Gruber AR Höner Zu Siederdissen C Amman F Badelt S Bartschat S Bernhart SH Beyer W Kehr S Lorenz R Tanzer A Yusuf D Tafer H Hofacker IL Stadler PF Animal snoRNAs and scaRNAs with exceptional structures. RNA Biol 2011 14 938 46 10.4161/rna.8.6.16603 21955586 PMC3256416

[B45] Yang J Zhang X Huang Z Zhou H Huang M Zhang S Chen Y Qu L snoSeeker: an advanced computational package for screening of guide and orphan snoRNA genes in the human genome. Nucleic Acids Res 2006 14 5112 23 10.1093/nar/gkl672 16990247 PMC1636440

[B46] Siepel A Bejerano G Pedersen JS Hinrichs AS Hou M Rosenbloom K Clawson H Spieth J Hillier LW Richards S Weinstock GM Wilson RK Gibbs RA Kent WJ Miller W Haussler D Evolutionarily conserved elements in vertebrate, insect, worm, and yeast genomes. Genome Res 2005 14 1034 50 10.1101/gr.3715005 16024819 PMC1182216

[B47] Schattner P Barberan-Soler S Lowe T A computational screen for mammalian pseudouridylation guide H/ACA RNAs. RNA 2006 14 15 25 10.1261/rna.2210406 16373490 PMC1370881

[B48] Tafer H Kehr S Hertel J Hofacker IL Stadler PF RNAsnoop: efficient target prediction for H/ACA snoRNAs. Bioinformatics 2010 14 610 16 10.1093/bioinformatics/btp680 20015949

[B49] Kehr S Bartschat S Stadler PF Tafer H PLEXY: efficient target prediction for box C/D snoRNAs. Bioinformatics 2011 14 279 80 10.1093/bioinformatics/btq642 21076148

[B50] Karijolich J Yu Y Spliceosomal snRNA modifications and their function. RNA Biol 2010 14 192 204 10.4161/rna.7.2.11207 20215871 PMC4154345

[B51] Kishore S Stamm S The snoRNA HBII-52 regulates alternative splicing of the serotonin receptor 2C. Science 2006 14 230 2 10.1126/science.1118265 16357227

[B52] Berninger P Jaskiewicz L Khorshid M Zavolan M Conserved generation of short products at piRNA loci. BMC Genomics 2011 14 46 10.1186/1471-2164-12-46 21247452 PMC3037900

[B53] Valen E Preker P Andersen PR Zhao X Chen Y Ender C Dueck A Meister G Sandelin A Jensen TH Biogenic mechanisms and utilization of small RNAs derived from human protein-coding genes. Nat Struct Mol Biol 2011 14 1075 82 10.1038/nsmb.2091 21822281

[B54] Cole C Sobala A Lu C Thatcher SR Bowman A Brown JW Green PJ Barton GJ Hutvagner G Filtering of deep sequencing data reveals the existence of abundant Dicer-dependent small RNAs derived from tRNAs. RNA 2009 14 2147 60 10.1261/rna.1738409 19850906 PMC2779667

[B55] Yamasaki S Ivanov P Hu GF Anderson P Angiogenin cleaves tRNA and promotes stress-induced translational repression. J Cell Biol 2009 14 35 42 10.1083/jcb.200811106 19332886 PMC2700517

[B56] Liao J Ma L Guo Y Zhang Y Zhou H Shao P Chen Y Qu L Deep sequencing of human nuclear and cytoplasmic small RNAs reveals an unexpectedly complex subcellular distribution of miRNAs and tRNA 3' trailers. PLoS One 2010 14 e10563 10.1371/journal.pone.0010563 20498841 PMC2871053

[B57] Bernhart SH Hofacker IL From consensus structure prediction to RNA gene finding. Brief Funct Genomic Proteomic 2009 14 461 71 10.1093/bfgp/elp043 19833701

[B58] Schubert T Pusch M Diermeier S Benes V Kremmer E Imhof A Längst G Df31 protein and snoRNAs maintain accessible higher-order structures of chromatin. Mol Cell 2012 14 434 44 10.1016/j.molcel.2012.08.021 23022379

[B59] Scott MS Ono M From snoRNA to miRNA: dual function regulatory non-coding RNAs. Biochimie 2011 14 1987 92 10.1016/j.biochi.2011.05.026 21664409 PMC3476530

[B60] Ule J Ule A Spencer J Williams A Hu J Cline M Wang H Clark T Fraser C Ruggiu M Zeeberg B Kane D Weinstein J Blume J Darnell R Nova regulates brain-specific splicing to shape the synapse. Nat Genet 2005 14 844 52 10.1038/ng1610 16041372

[B61] Hafner M Landgraf P Ludwig J Rice A Ojo T Lin C Holoch D Lim C Tuschl T Identification of microRNAs and other small regulatory RNAs using cDNA library sequencing. Methods 2008 14 3 12 10.1016/j.ymeth.2007.09.009 18158127 PMC2847350

[B62] Hoffmann S Otto C Kurtz S Sharma C Khaitovich P Vogel J Stadler P Hackermüller J Fast mapping of short sequences with mismatches, insertions and deletions using index structures. PLoS Comput Biol 2009 14 e1000502 10.1371/journal.pcbi.1000502 19750212 PMC2730575

[B63] ENCODE data coordination center at UCSC. http://genome.ucsc.edu/ENCODE/downloads.html 10.1093/database/baw001 PMC4792520 26980513

[B64] Nawrocki EP Kolbe DL Eddy SR Infernal 1.0: inference of RNA alignments. Bioinformatics 2009 14 1335 7 10.1093/bioinformatics/btp157 19307242 PMC2732312

[B65] Maden BE Corbett ME Heeney PA Pugh K Ajuh PM Classical and novel approaches to the detection and localization of the numerous modified nucleotides in eukaryotic ribosomal RNA. Biochimie 1995 14 22 9 10.1016/0300-9084(96)88100-4 7599273

[B66] Maden BE Mapping 2'-O-methyl groups in ribosomal RNA. Methods 2001 14 374 82 10.1006/meth.2001.1250 11860292

[B67] Ofengand J Del Campo M Kaya Y Mapping pseudouridines in RNA molecules. Methods 2001 14 365 73 10.1006/meth.2001.1249 11860291

[B68] Morla AO Draetta G Beach D Wang JY Reversible tyrosine phosphorylation of cdc2: dephosphorylation accompanies activation during entry into mitosis. Cell 1989 14 193 203 10.1016/0092-8674(89)90415-7 2473839

[B69] Pines J Hunter T Isolation of a human cyclin cDNA: evidence for cyclin mRNA and protein regulation in the cell cycle and for interaction with p34cdc2. Cell 1989 14 833 46 10.1016/0092-8674(89)90936-7 2570636

[B70] Elvin P Evans CW Cell adhesiveness and the cell cycle: correlation in synchronized Balb/c 3T3 cells. Biol Cell 1983 14 1 9 6673784 10.1111/j.1768-322x.1984.tb00196.x

