# Supplementary material for: Insights into snoRNA biogenesis and processing from PAR-CLIP of snoRNA core proteins and small RNA sequencing
Source: Genome Biol. 2013 May 26;14(5):R45. doi: 10.1186/gb-2013-14-5-r45 (PMC4053766; doi:10.1186/gb-2013-14-5-r45)
Supplement: Additional file 1 — Profiles of PAR-CLIPs reads obtained with various core snoRNP proteins for snoRNAs and scaRNAs. The proteins and normalized read counts are shown on the y-axis. The snoRNA and location of boxes are shown at the bottom. Red bars in the profiles indicate the number of T→C mutations observed at individual nucleotides in the PAR-CLIP reads. [file gb-2013-14-5-r45-S1.PDF]

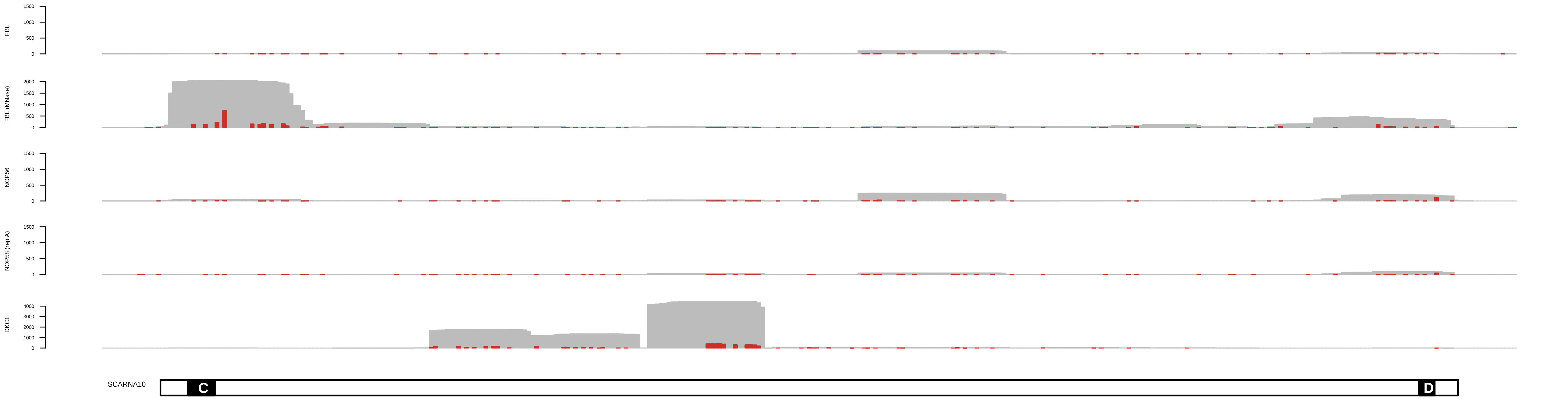
FBL
FBL (MNase)
NOP56
NOP58 (rep A)
DKC1
SCARNA10
C
D

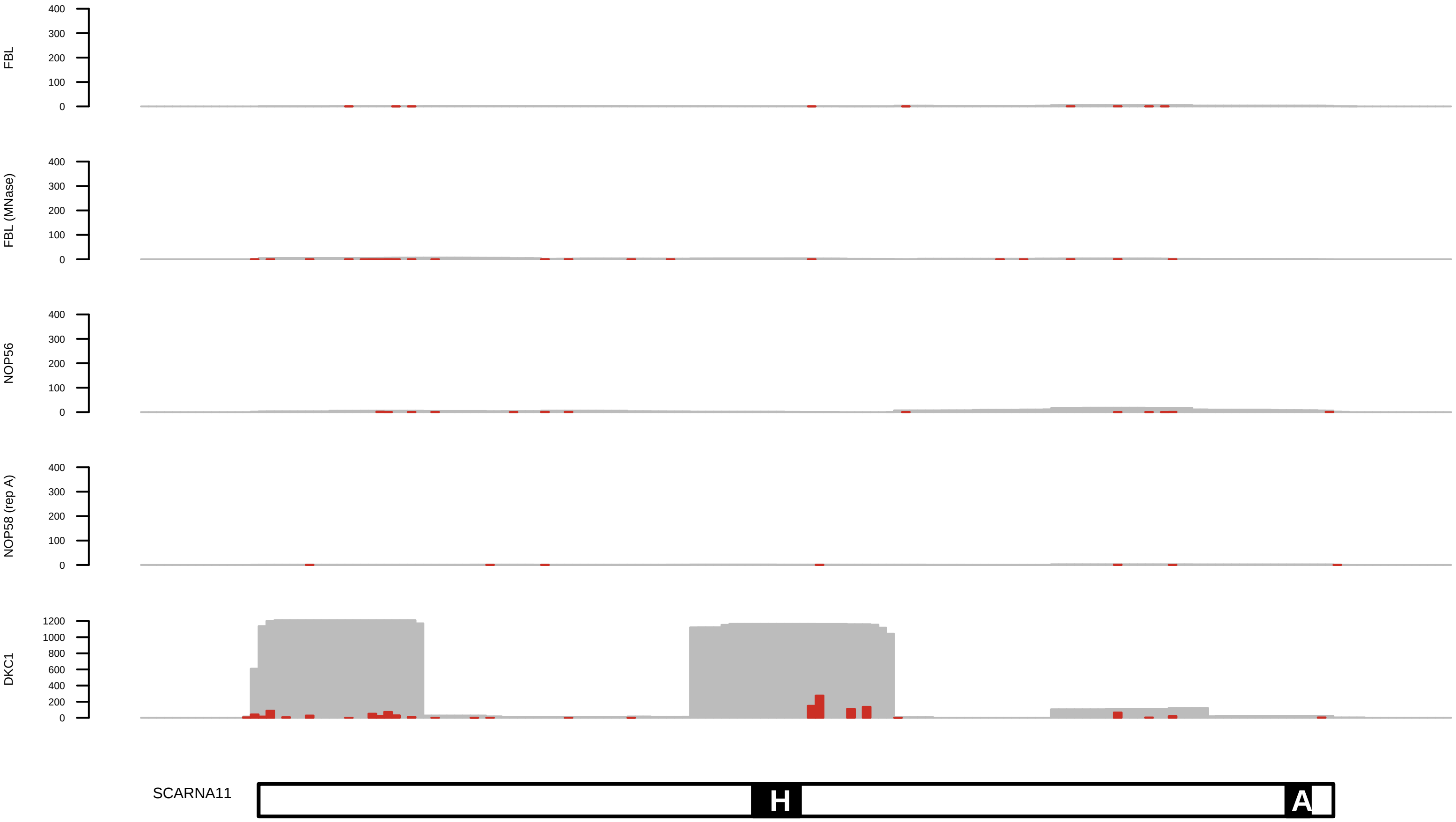

FBL
FBL (MNase)
NOP56
NOP58 (rep A)
DKC1
400
300
200
100
0
1200
1000
800
600
400
200
0
SCARNA11
H
A

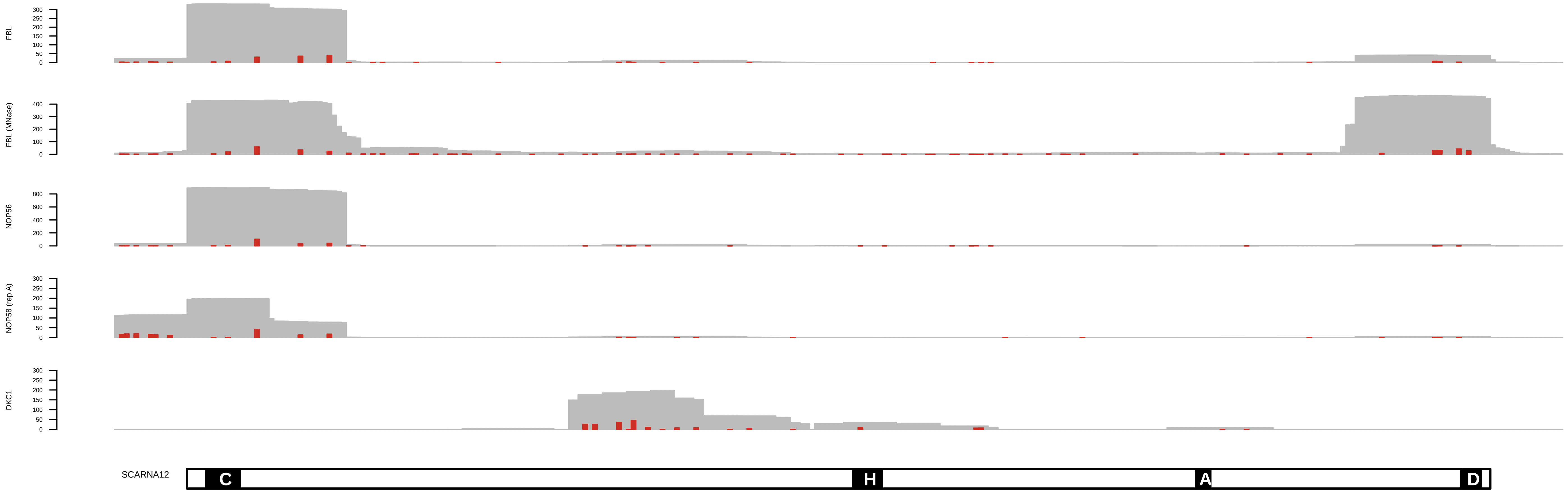
FBL
300
250
200
150
100
50
0
FBL (MNase)
400
300
200
100
0
NOP56
800
600
400
200
0
NOP58 (rep A)
300
250
200
150
100
50
0
DKC1
300
250
200
150
100
50
0
SCARNA12
C
H
A
D

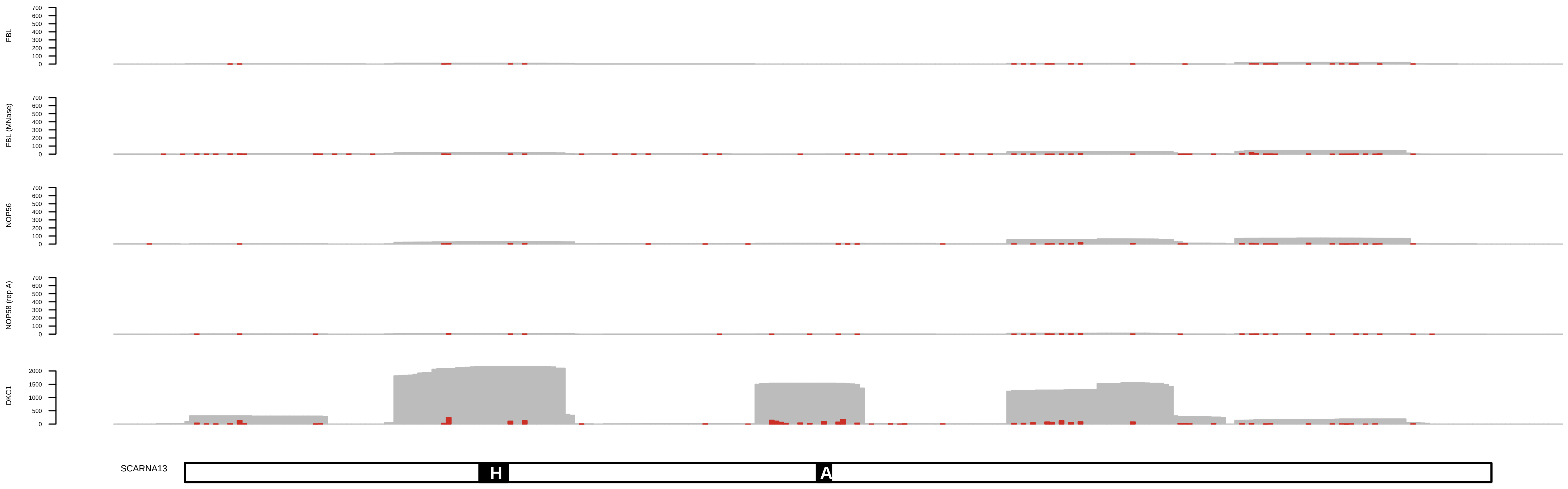
FBL
700
600
500
400
300
200
100
0
FBL (MNase)
NOP56
NOP58 (rep A)
DKC1
2000
1500
1000
500
0
SCARNA13
H
A

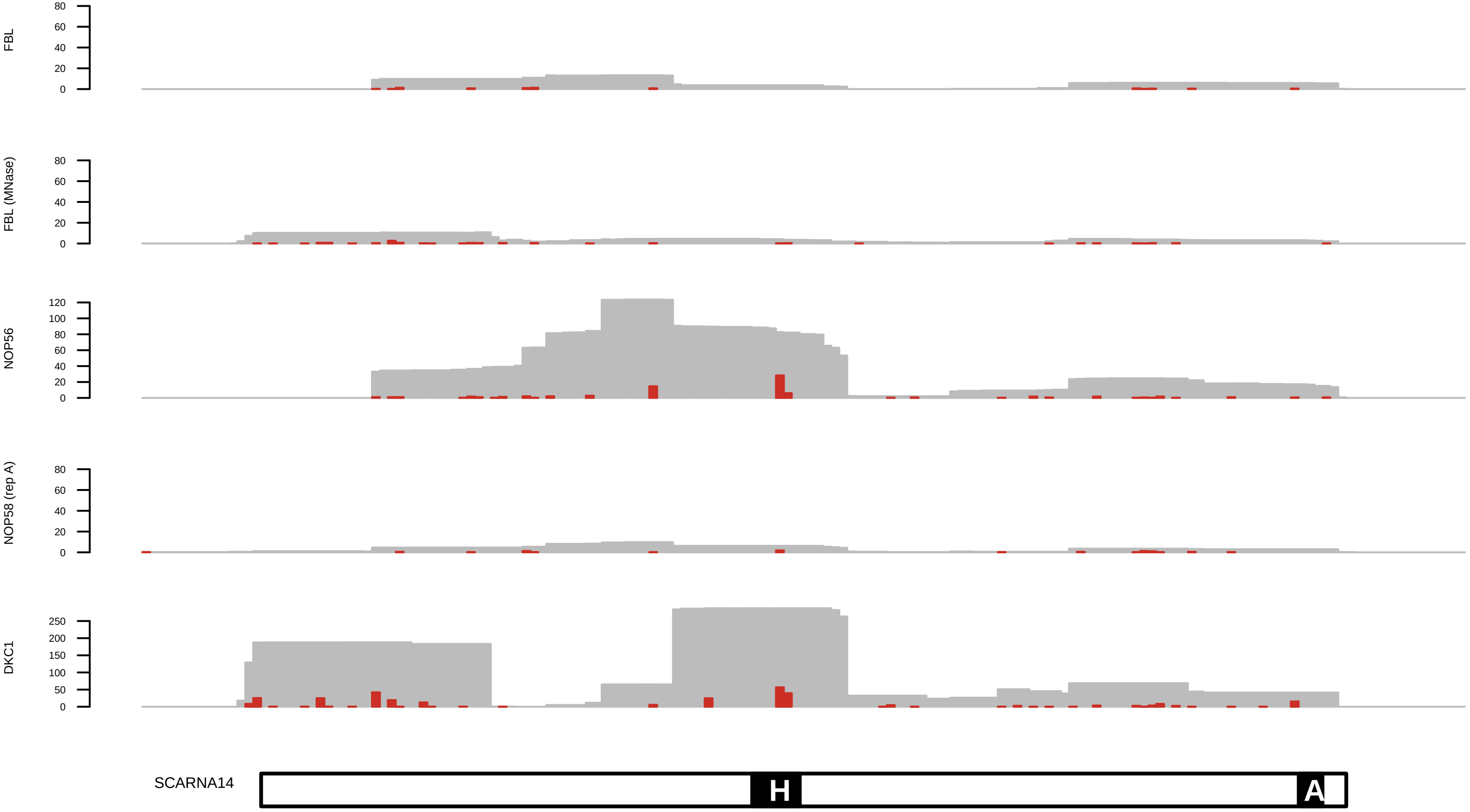
FBL
FBL (MNase)
NOP56
NOP58 (rep A)
DKC1
SCARNA14
H
A

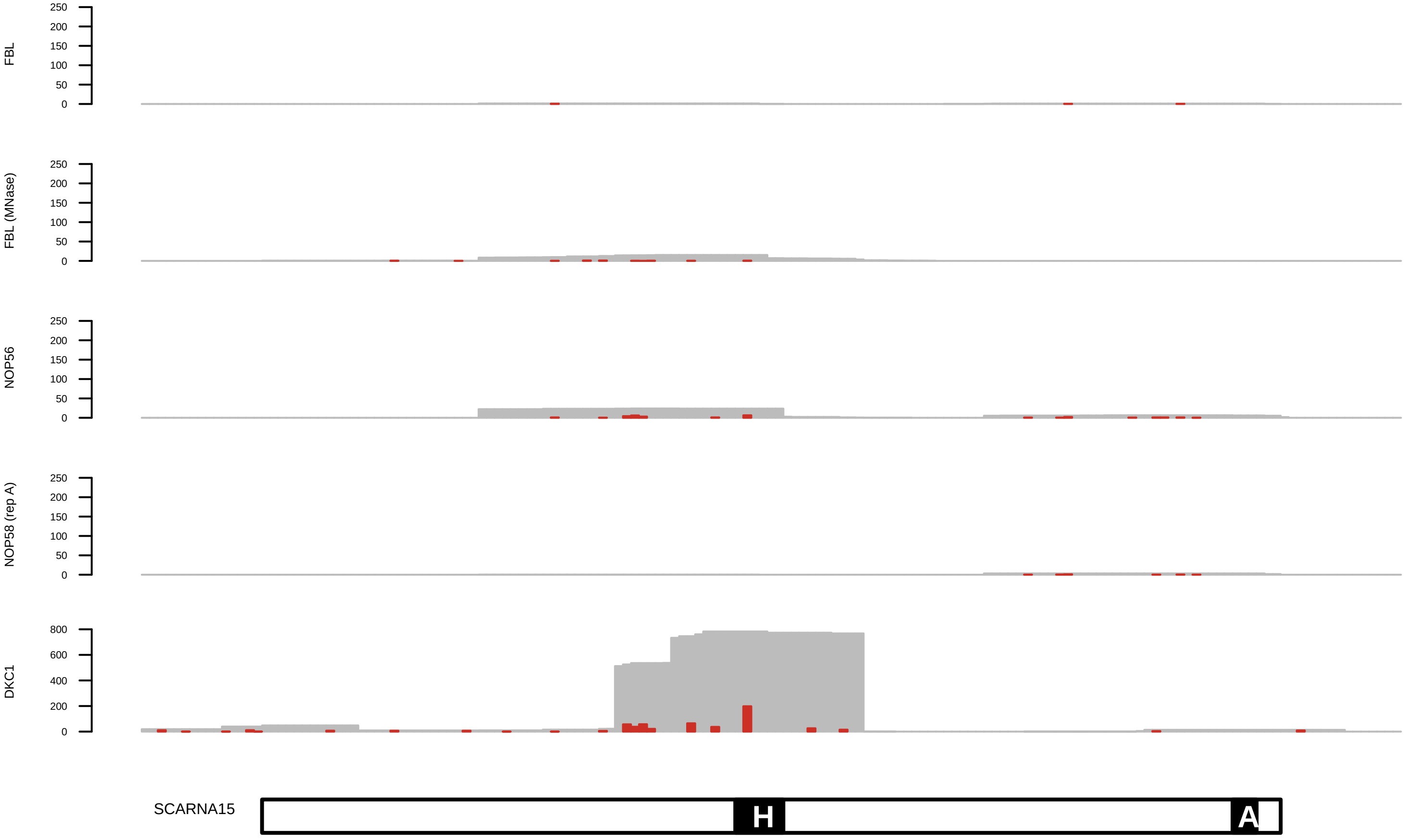
FBL
FBL (MNase)
NOP56
NOP58 (rep A)
DKC1
0
50
100
150
200
250
400
600
800
SCARNA15
H
A

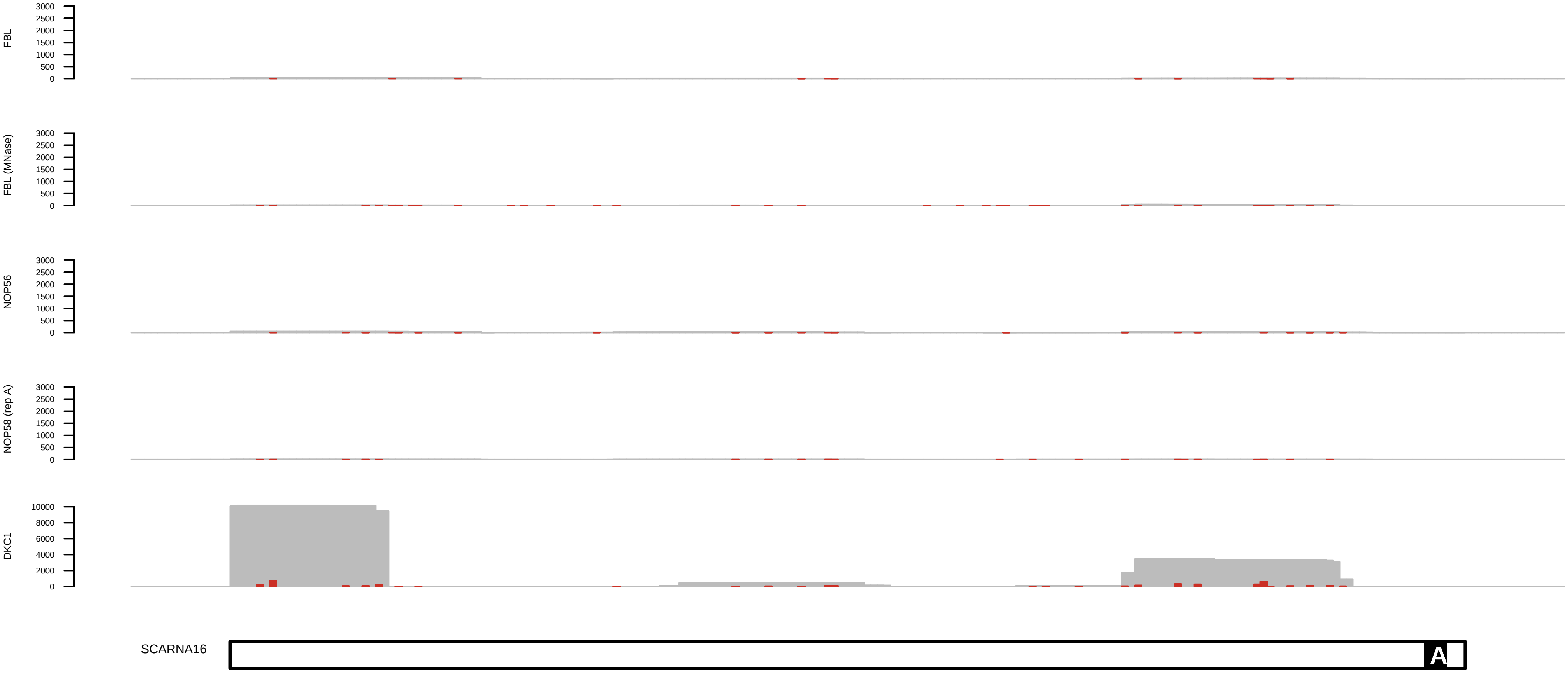

FBL
FBL (MNase)
NOP56
NOP58 (rep A)
DKC1
3000
2500
2000
1500
1000
500
0
10000
8000
6000
4000
2000
SCARNA16
A

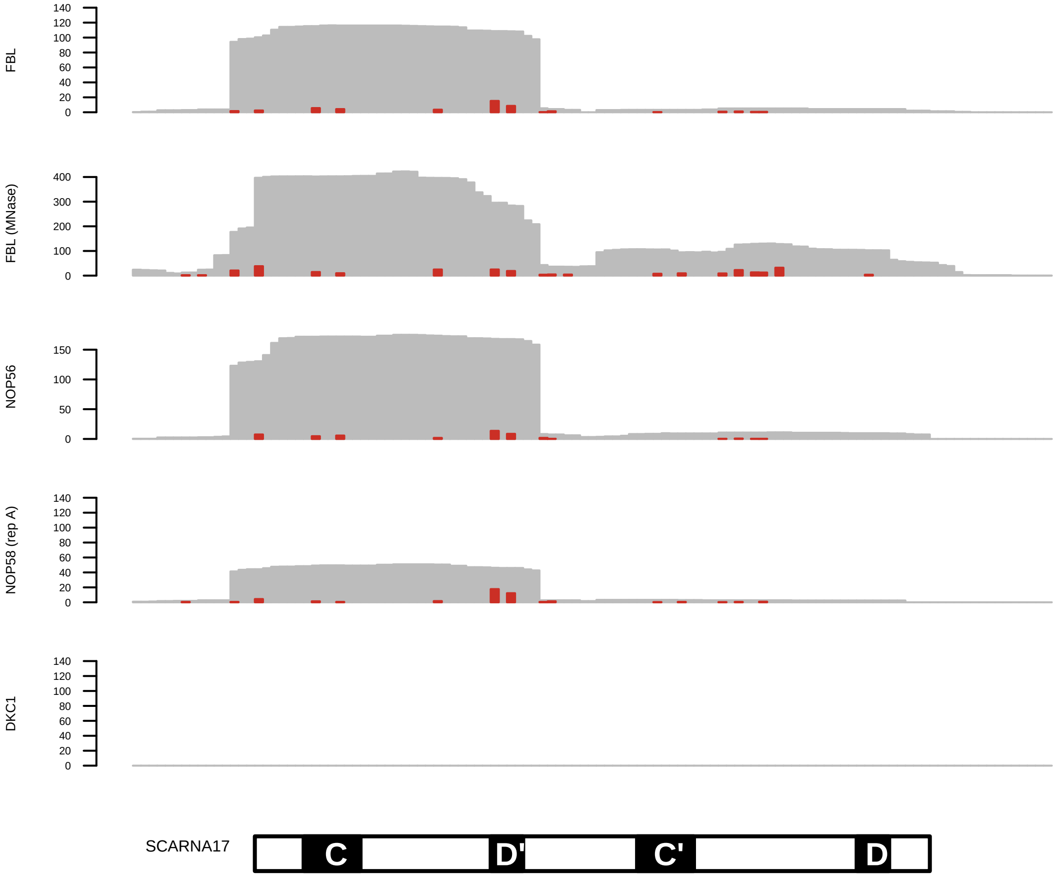

FBL
0
20
40
60
80
100
120
140
FBL (MNase)
0
100
200
300
400
NOP56
0
50
100
150
NOP58 (rep A)
0
20
40
60
80
100
120
140
DKC1
0
20
40
60
80
100
120
140
SCARNA17
C
D'
C'
D

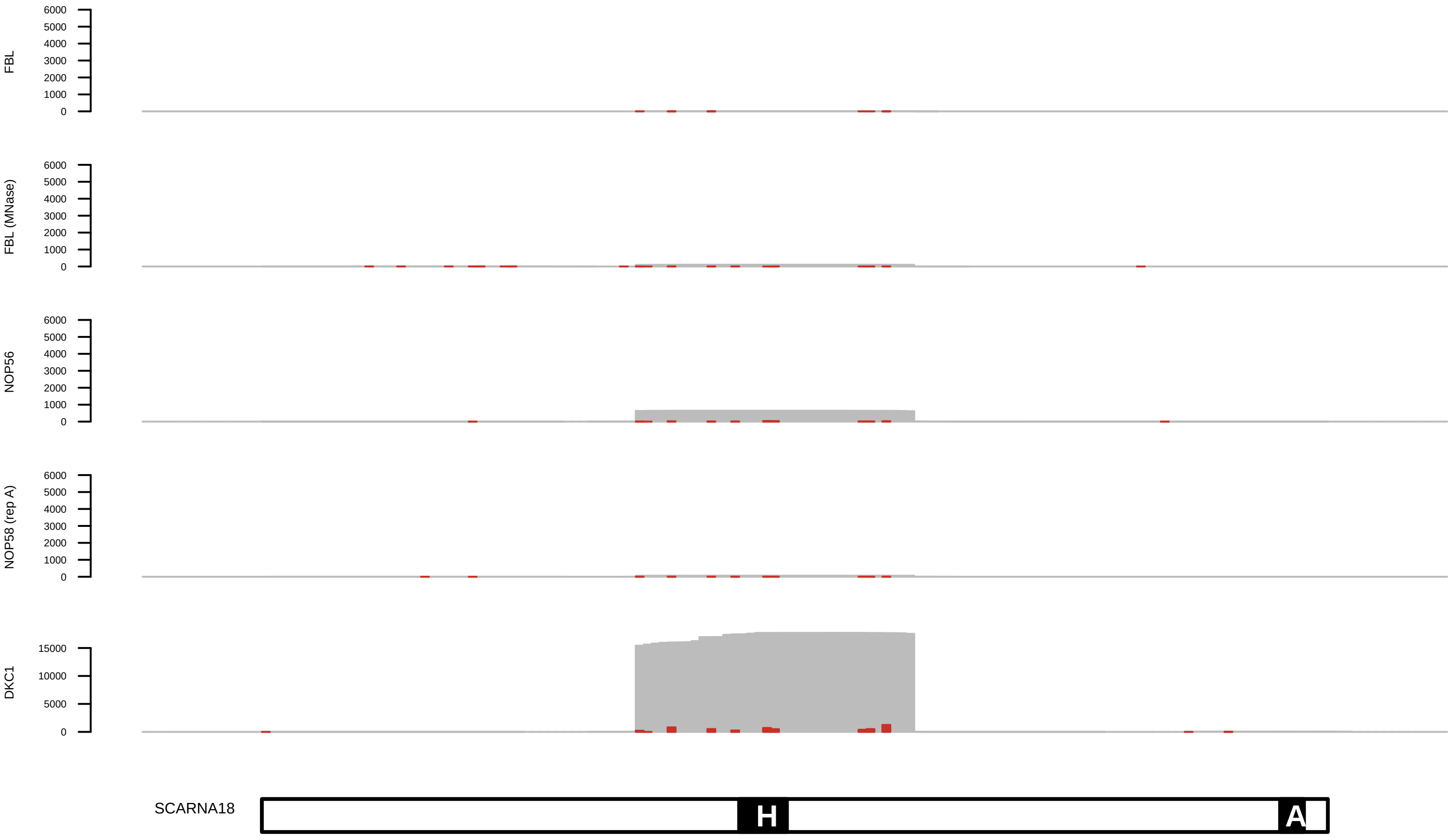
FBL
6000
5000
4000
3000
2000
1000
0
FBL (MNase)
6000
5000
4000
3000
2000
1000
0
NOP56
6000
5000
4000
3000
2000
1000
0
NOP58 (rep A)
6000
5000
4000
3000
2000
1000
0
DKC1
15000
10000
5000
0
SCARNA18
H
A

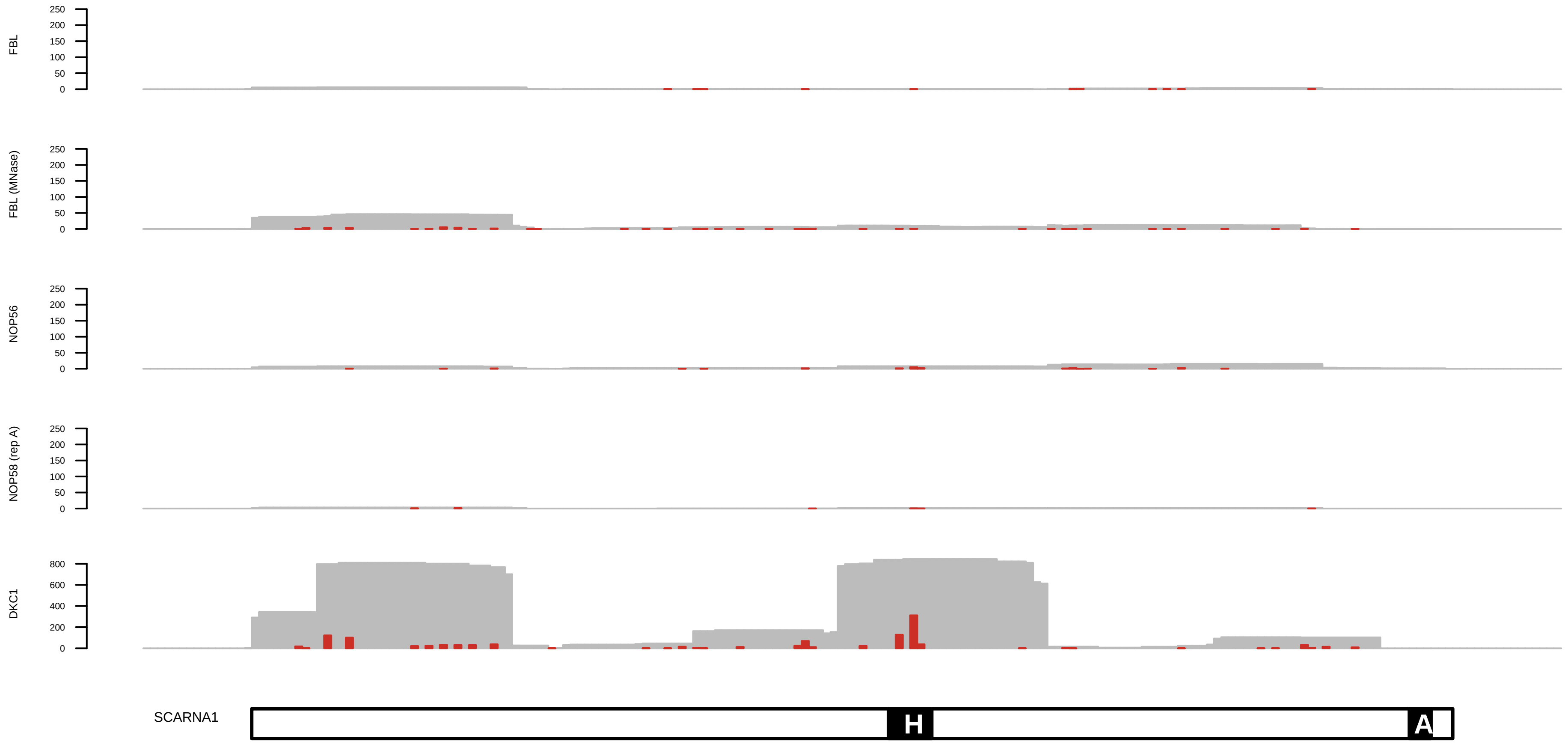

FBL
FBL (MNase)
NOP56
NOP58 (rep A)
DKC1
250
200
150
100
50
0
800
600
400
200
SCARNA1
H
A

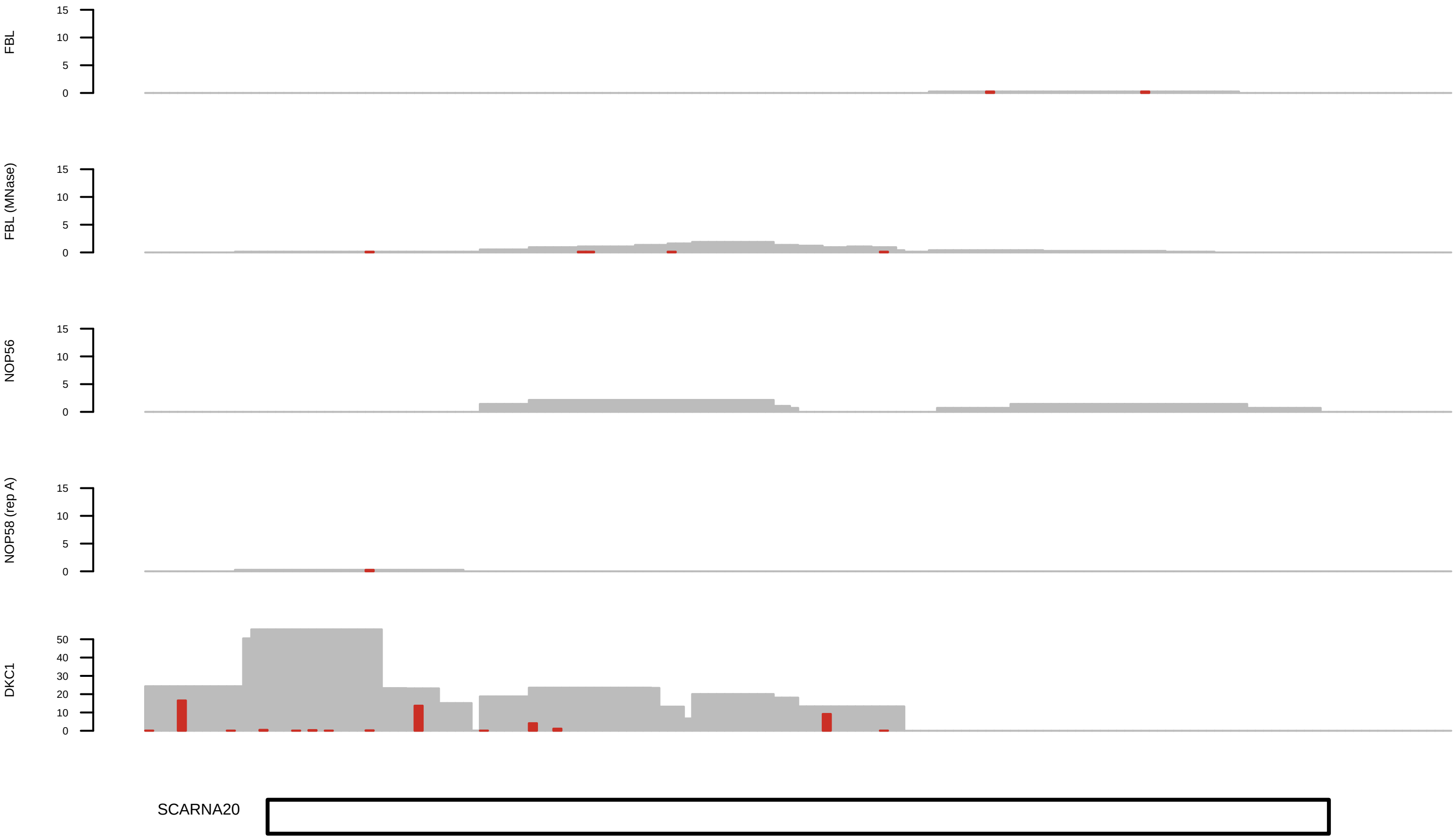
FBL
FBL (MNase)
NOP56
NOP58 (rep A)
DKC1
SCARNA20
0
5
10
15
0
10
20
30
40
50

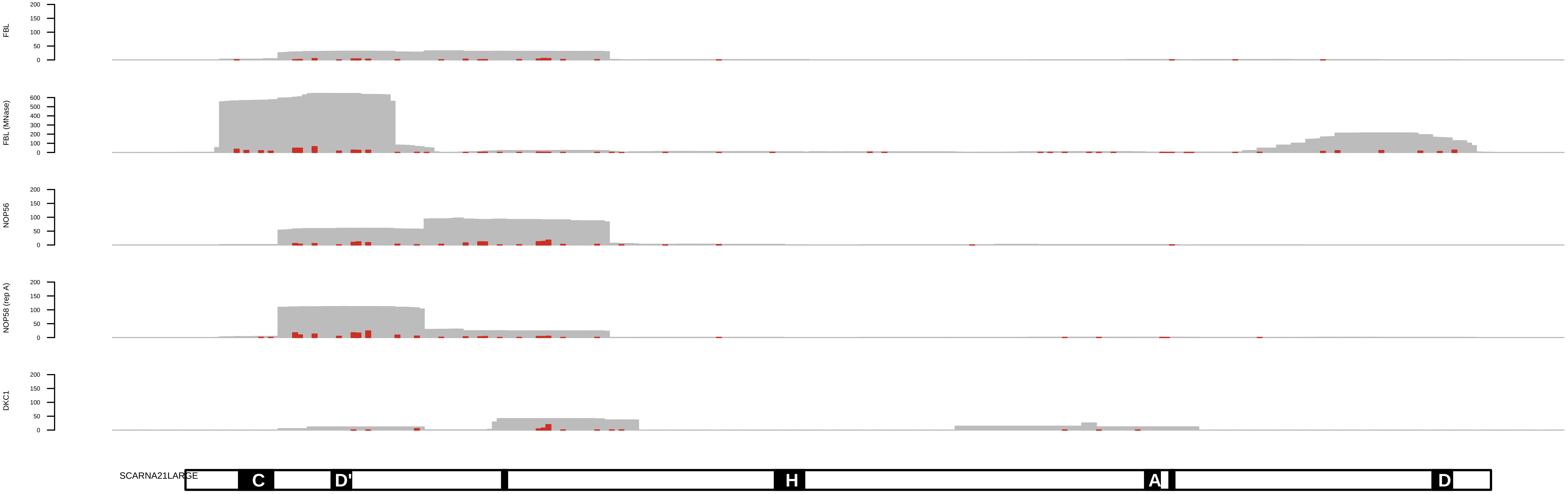

FBL
FBL (MNase)
NOP56
NOP58 (rep A)
DKC1
SCARNA21LARGE
C
D'
H
A
D

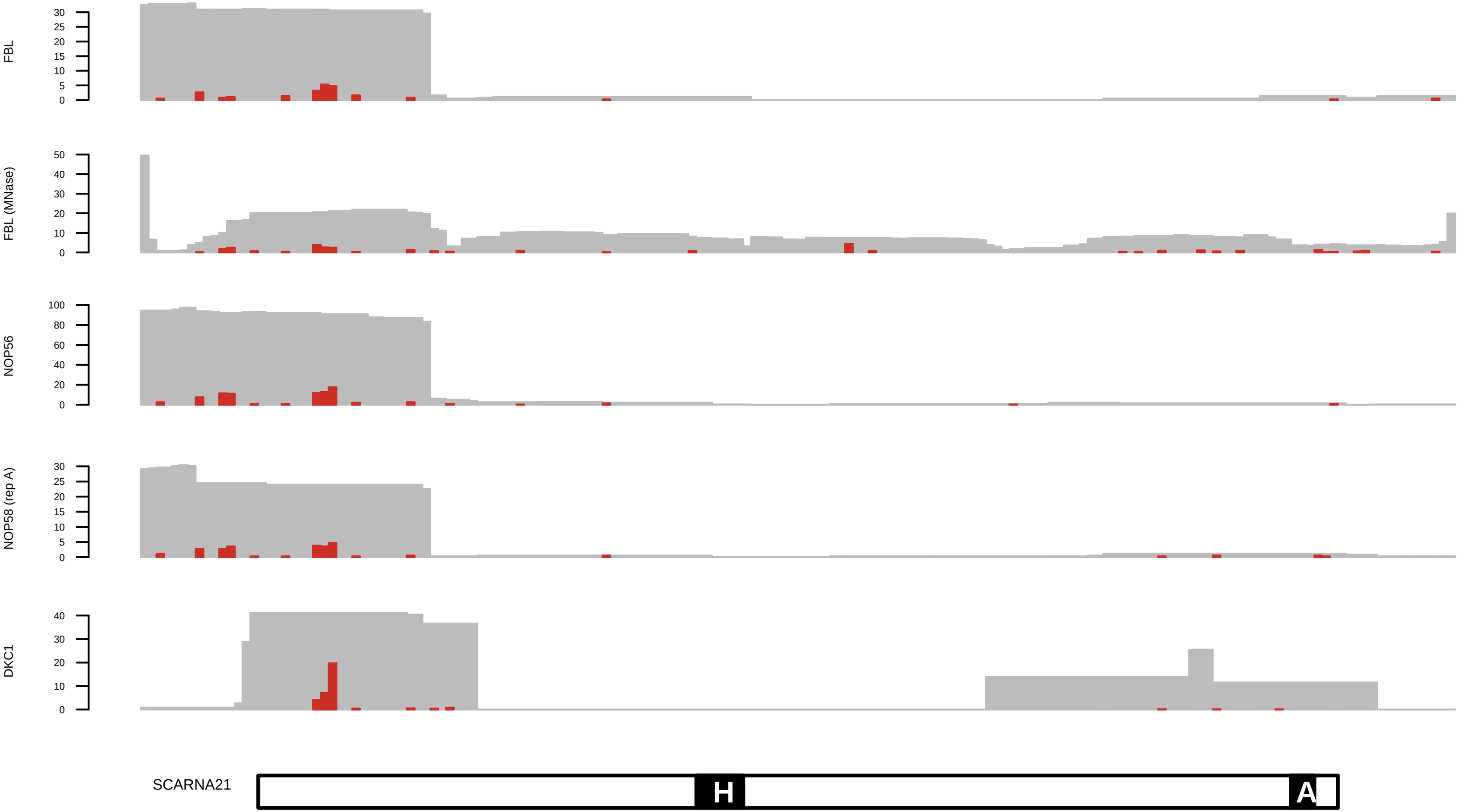

FBL
30
25
20
15
10
5
0
FBL (MNase)
50
40
30
20
10
0
NOP56
100
80
60
40
20
0
NOP58 (rep A)
30
25
20
15
10
5
0
DKC1
40
30
20
10
0
SCARNA21
H
A

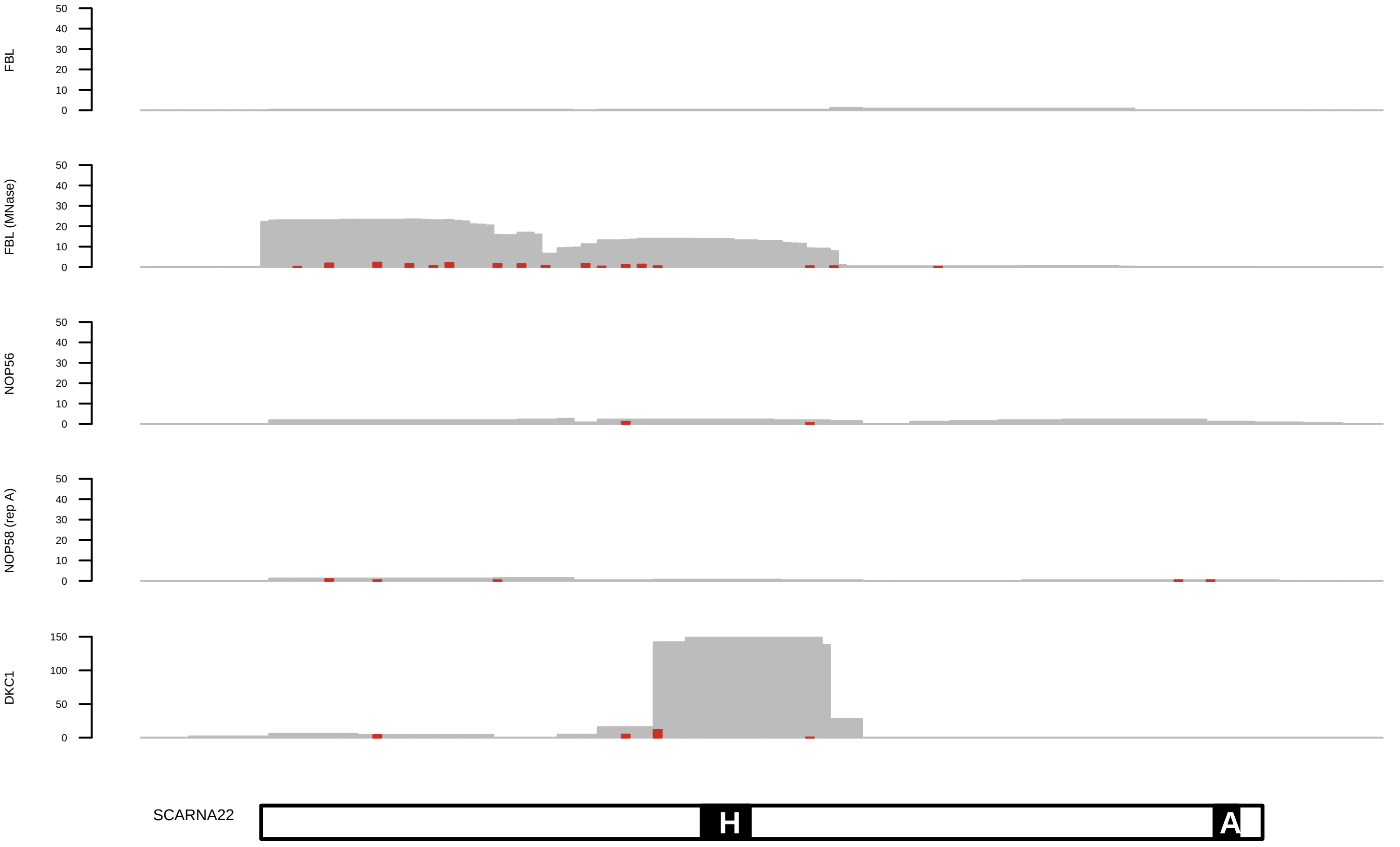
FBL
FBL (MNase)
NOP56
NOP58 (rep A)
DKC1
0
10
20
30
40
50
100
150
SCARNA22
H
A

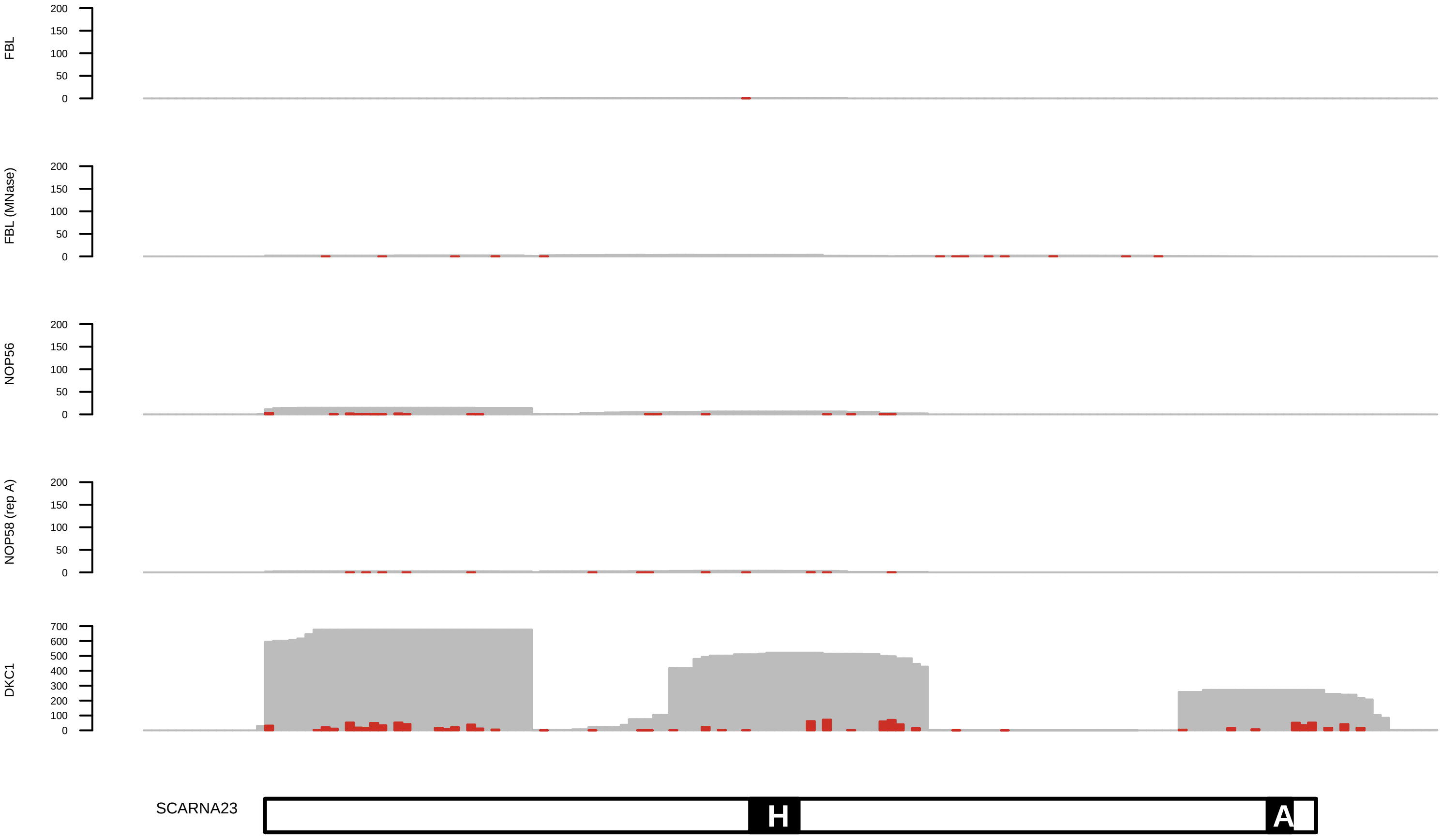

FBL
FBL (MNase)
NOP56
NOP58 (rep A)
DKC1
200
150
100
50
0
700
600
500
400
300
200
100
0
SCARNA23
H
A

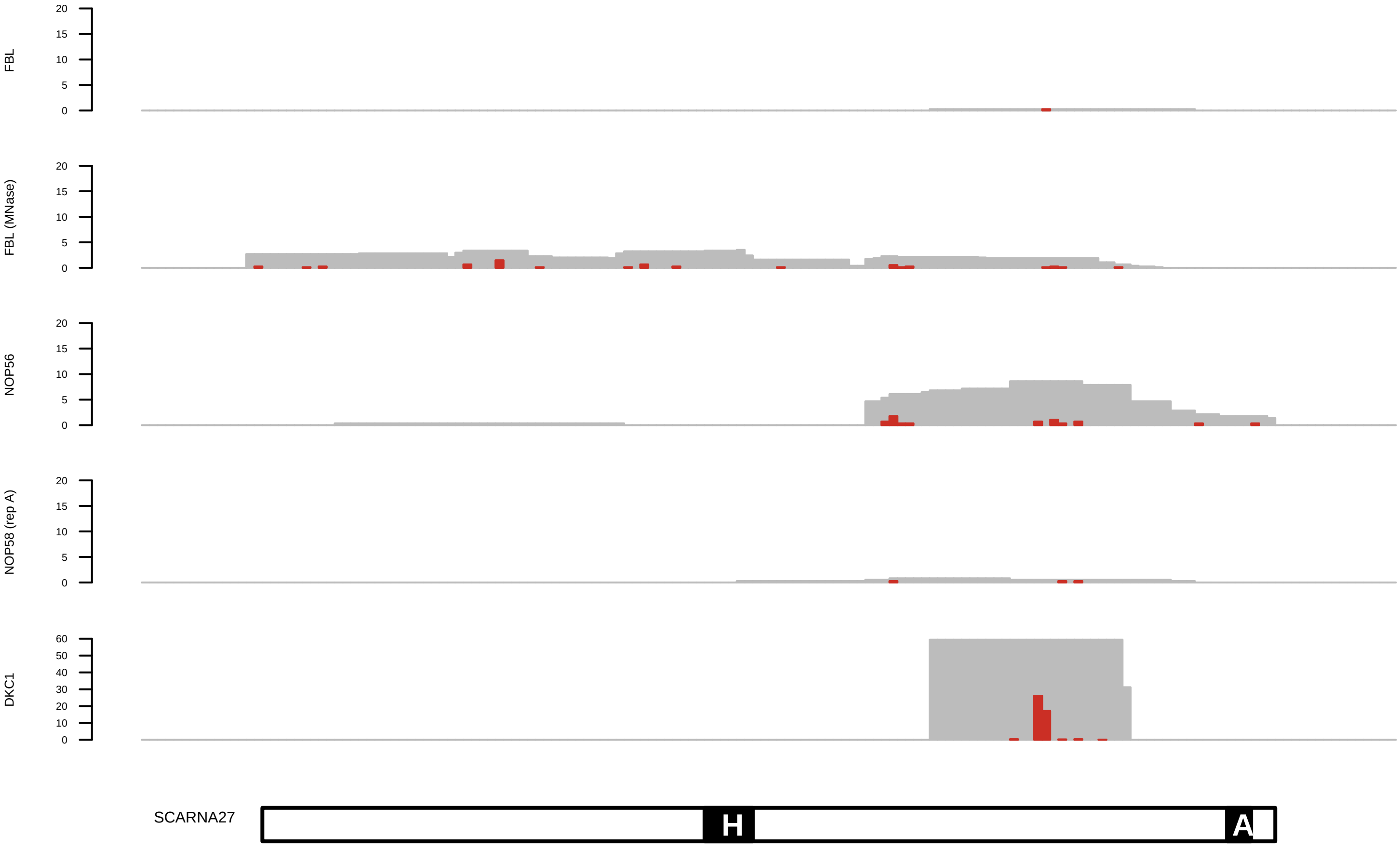
FBL
FBL (MNase)
NOP56
NOP58 (rep A)
DKC1
0
5
10
15
20
30
40
50
60
SCARNA27
H
A

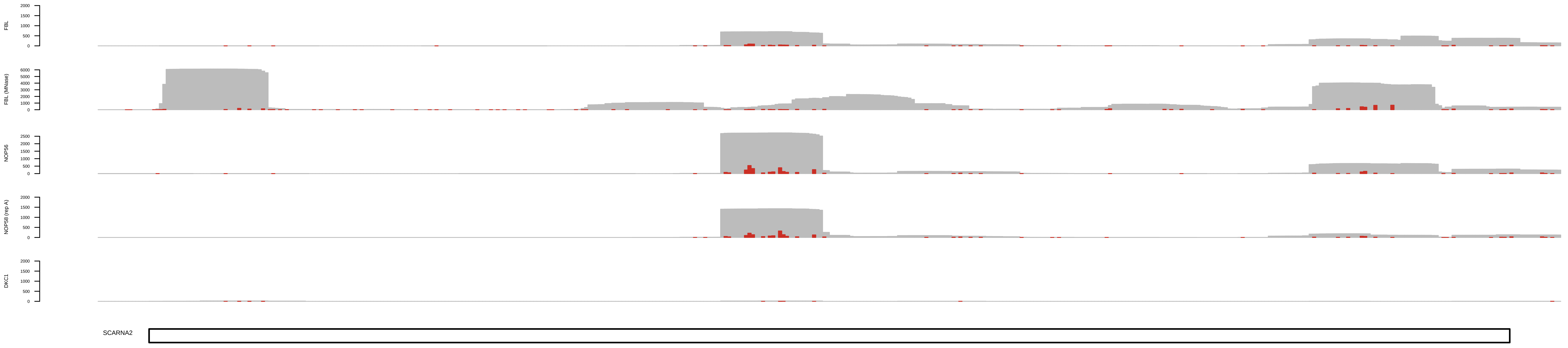
FBL
FBL (MNase)
NOP56
NOP58 (rep A)
DKC1
SCARNA2

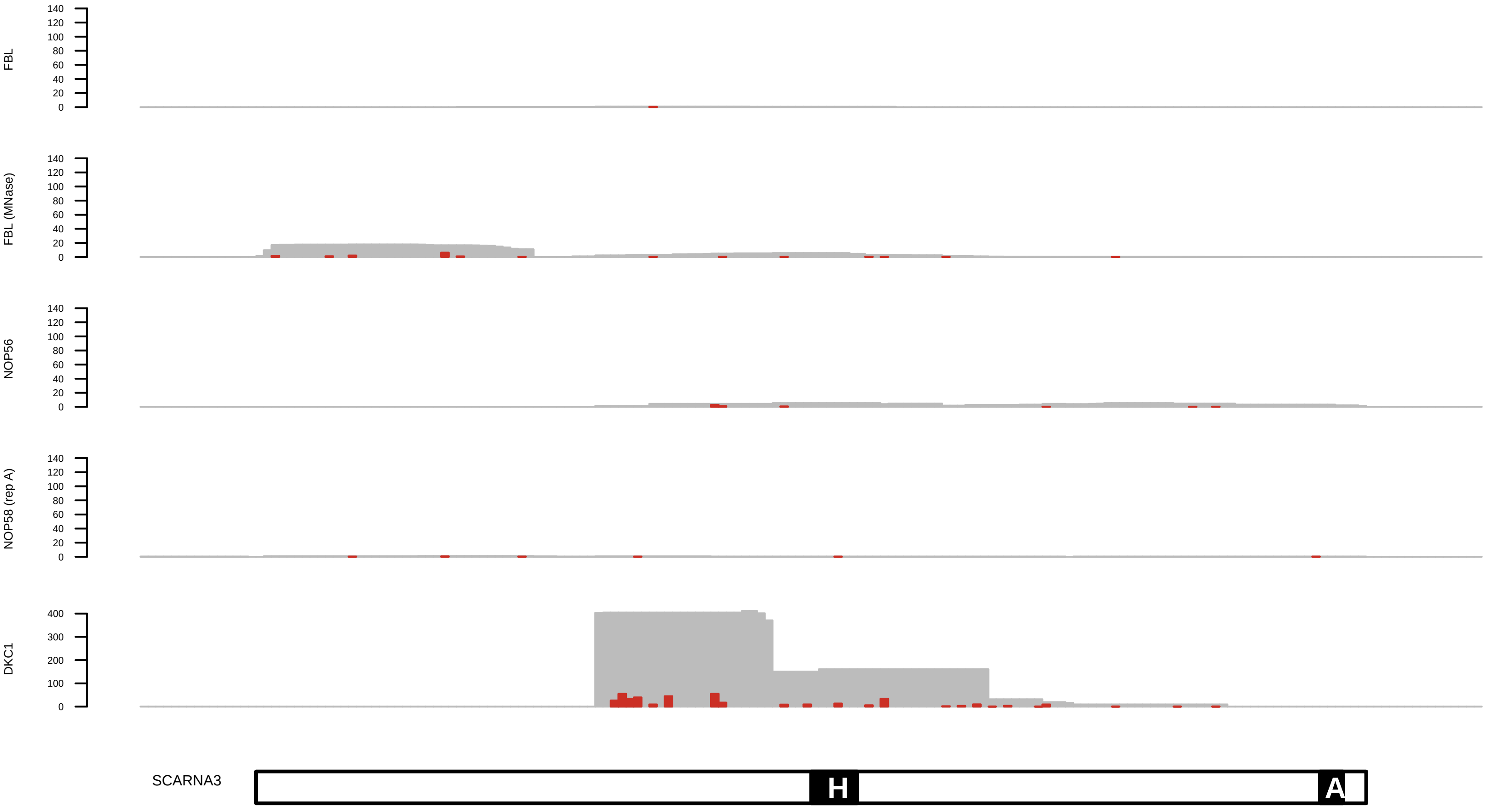
FBL
FBL (MNase)
NOP56
NOP58 (rep A)
DKC1
140
120
100
80
60
40
20
0
400
300
200
100
SCARNA3
H
A

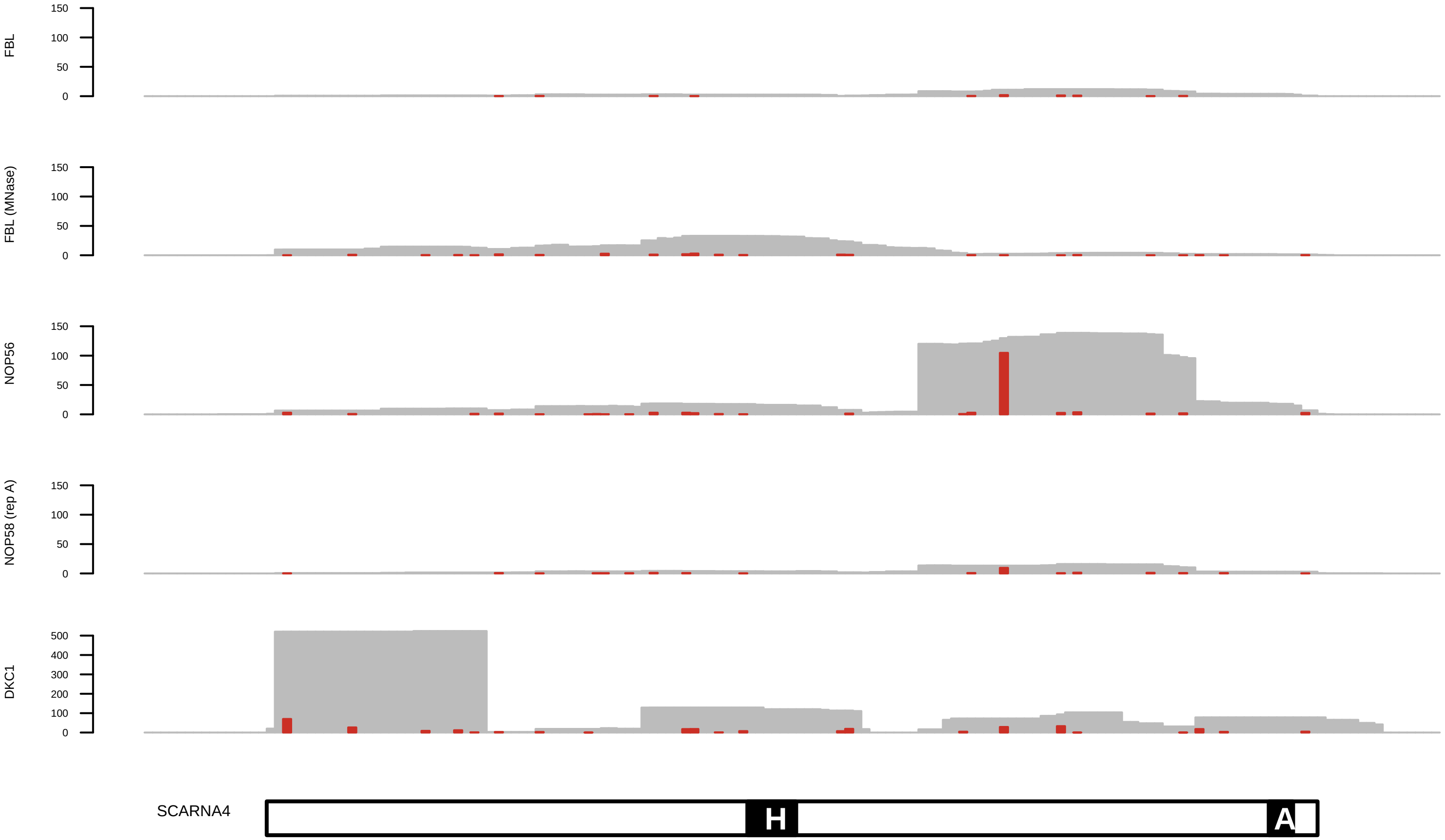

FBL
150
100
50
0
FBL (MNase)
150
100
50
0
NOP56
150
100
50
0
NOP58 (rep A)
150
100
50
0
DKC1
500
400
300
200
100
0
SCARNA4
H
A

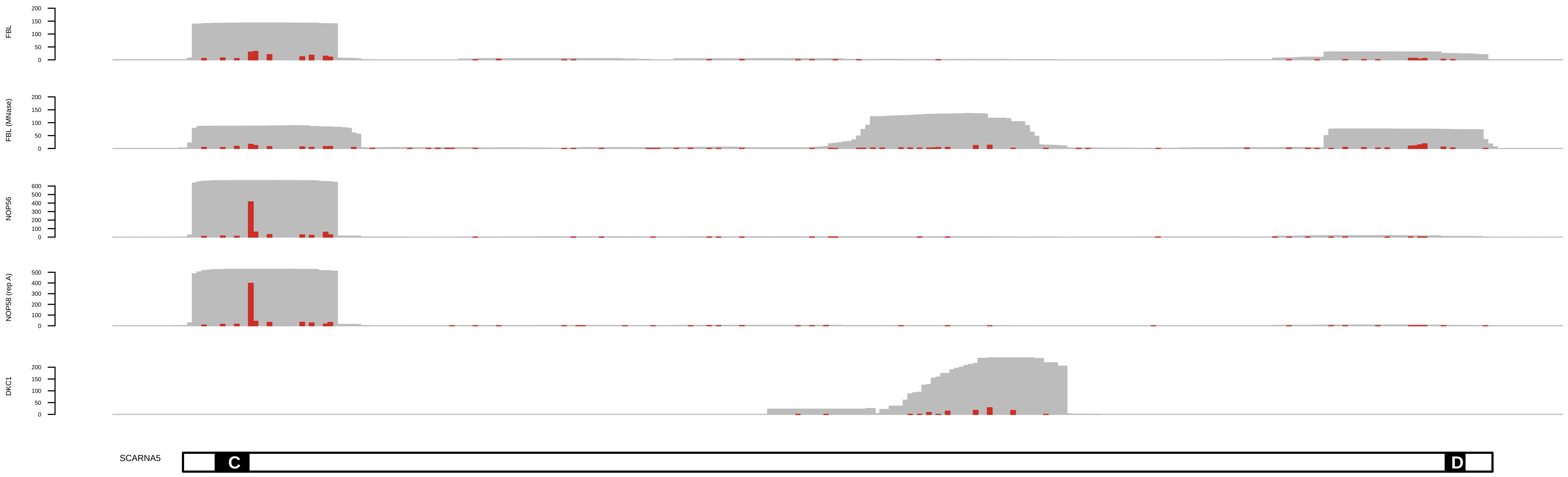

FBL
FBL (MNase)
NOP56
NOP58 (rep A)
DKC1
SCARNA5
C
D

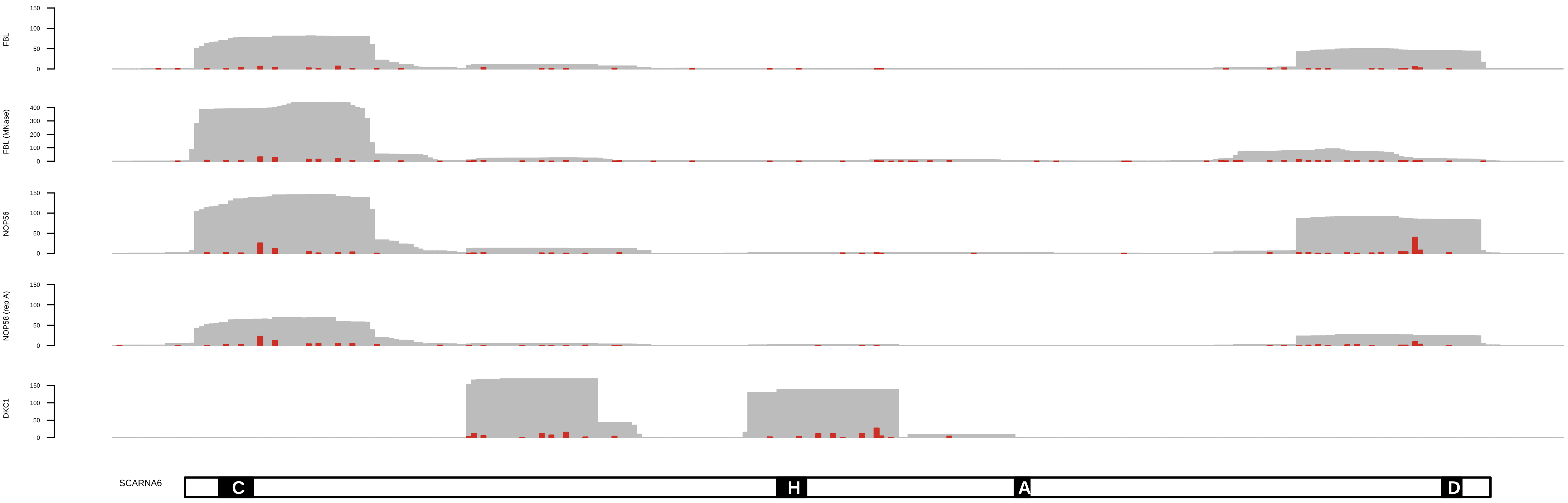
FBL
150
100
50
0
FBL (MNase)
400
300
200
100
0
NOP56
150
100
50
0
NOP58 (rep A)
150
100
50
0
DKC1
150
100
50
0
SCARNA6
C
H
A
D

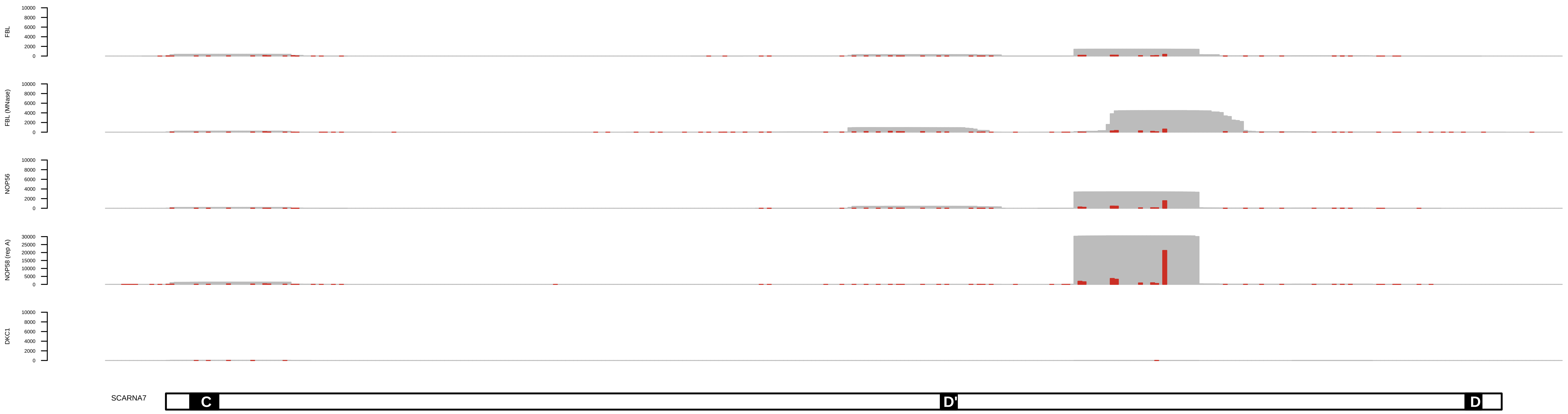
FBL
FBL (MNase)
NOP56
NOP58 (rep A)
DKC1
SCARNA7
C
D'
D

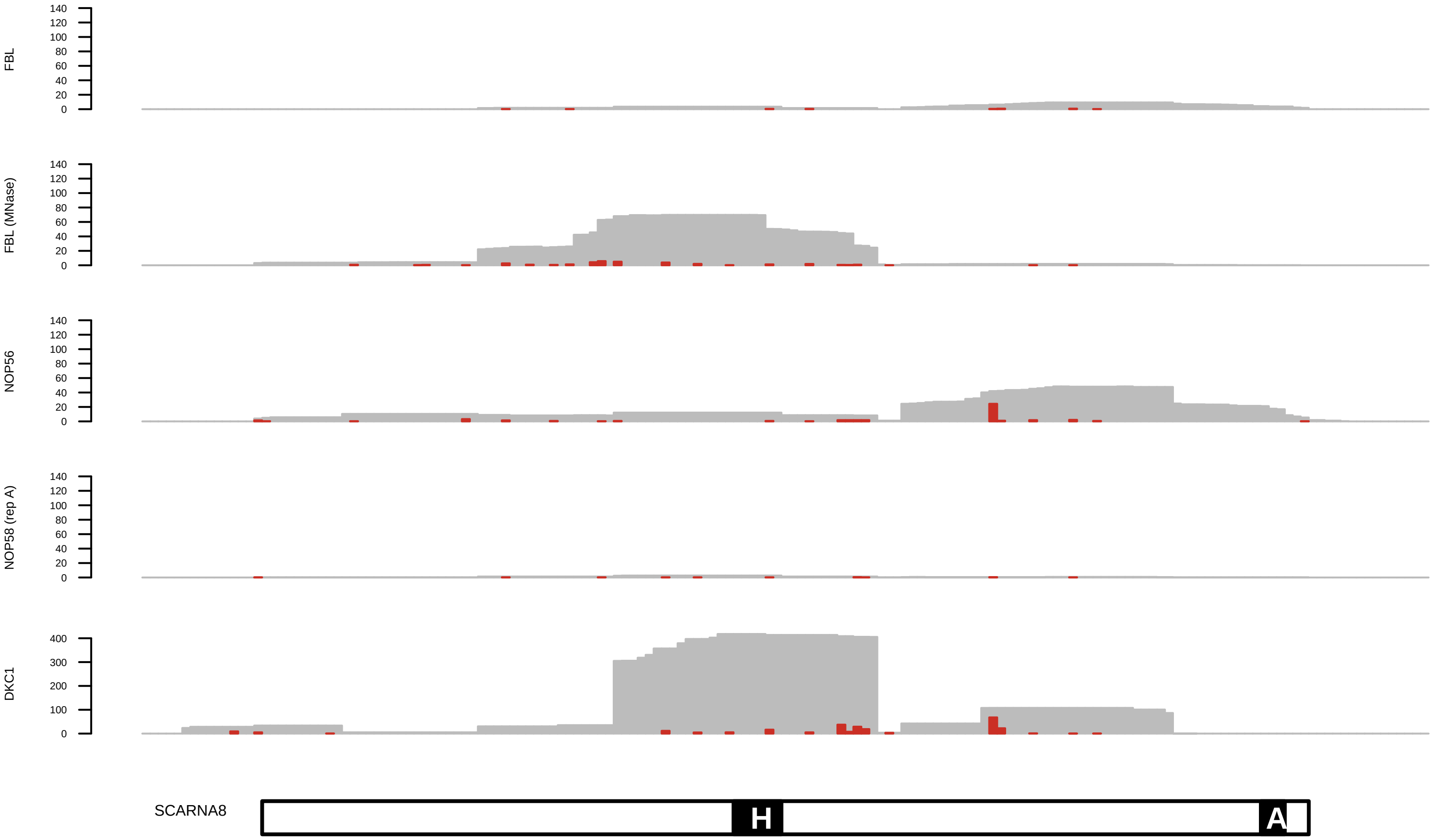

FBL
FBL (MNase)
NOP56
NOP58 (rep A)
DKC1
0
20
40
60
80
100
120
140
0
100
200
300
400
SCARNA8
H
A

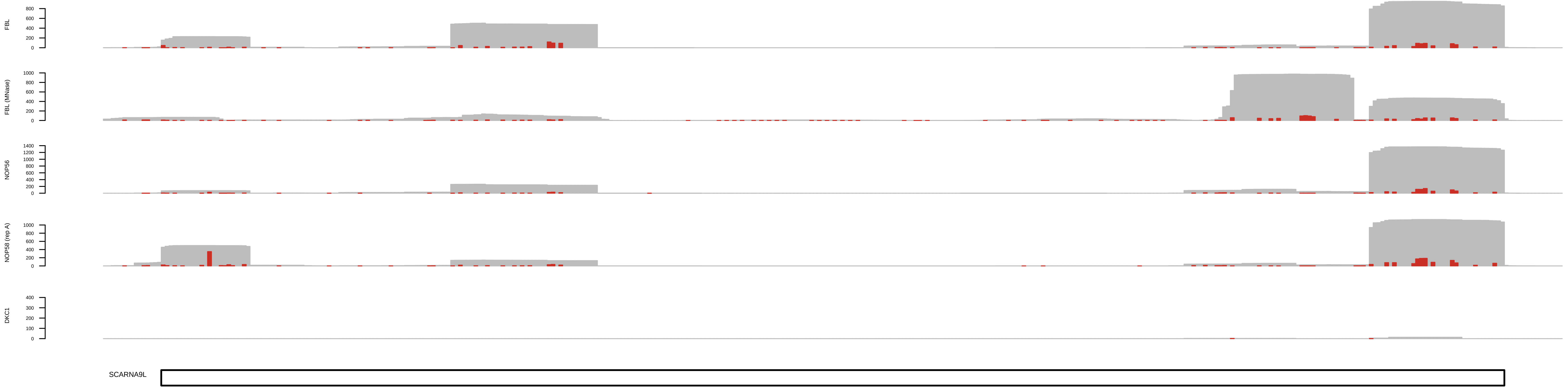
FBL
800
600
400
200
0
FBL (MNase)
1000
800
600
400
200
0
NOP56
1400
1200
1000
800
600
400
200
0
NOP58 (rep A)
1000
800
600
400
200
0
DKC1
400
300
200
100
0
SCARNA9L

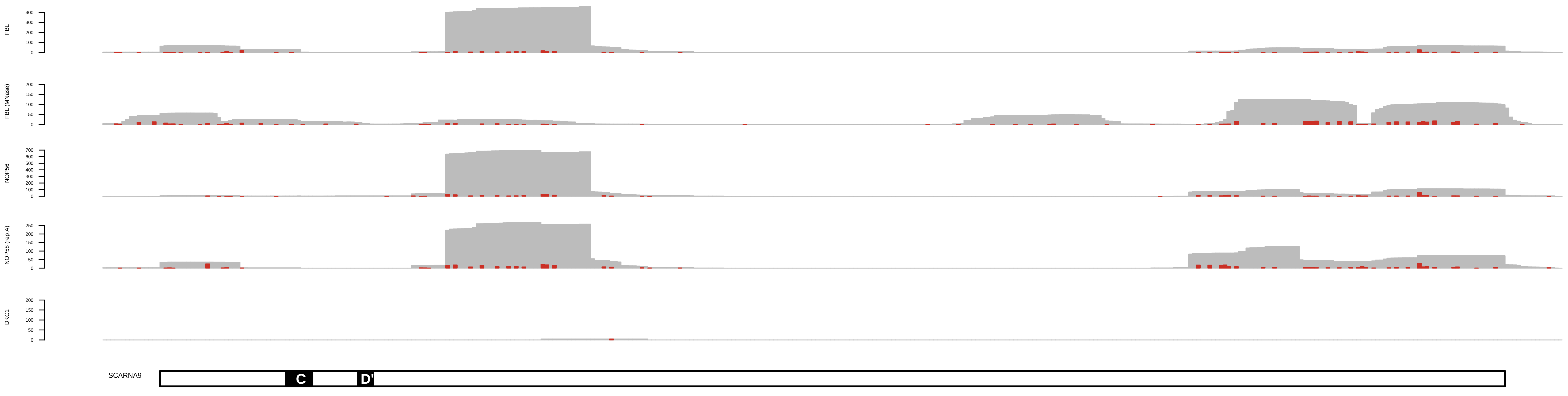
FBL
FBL (MNase)
NOP56
NOP58 (rep A)
DKC1
SCARNA9
C
D'

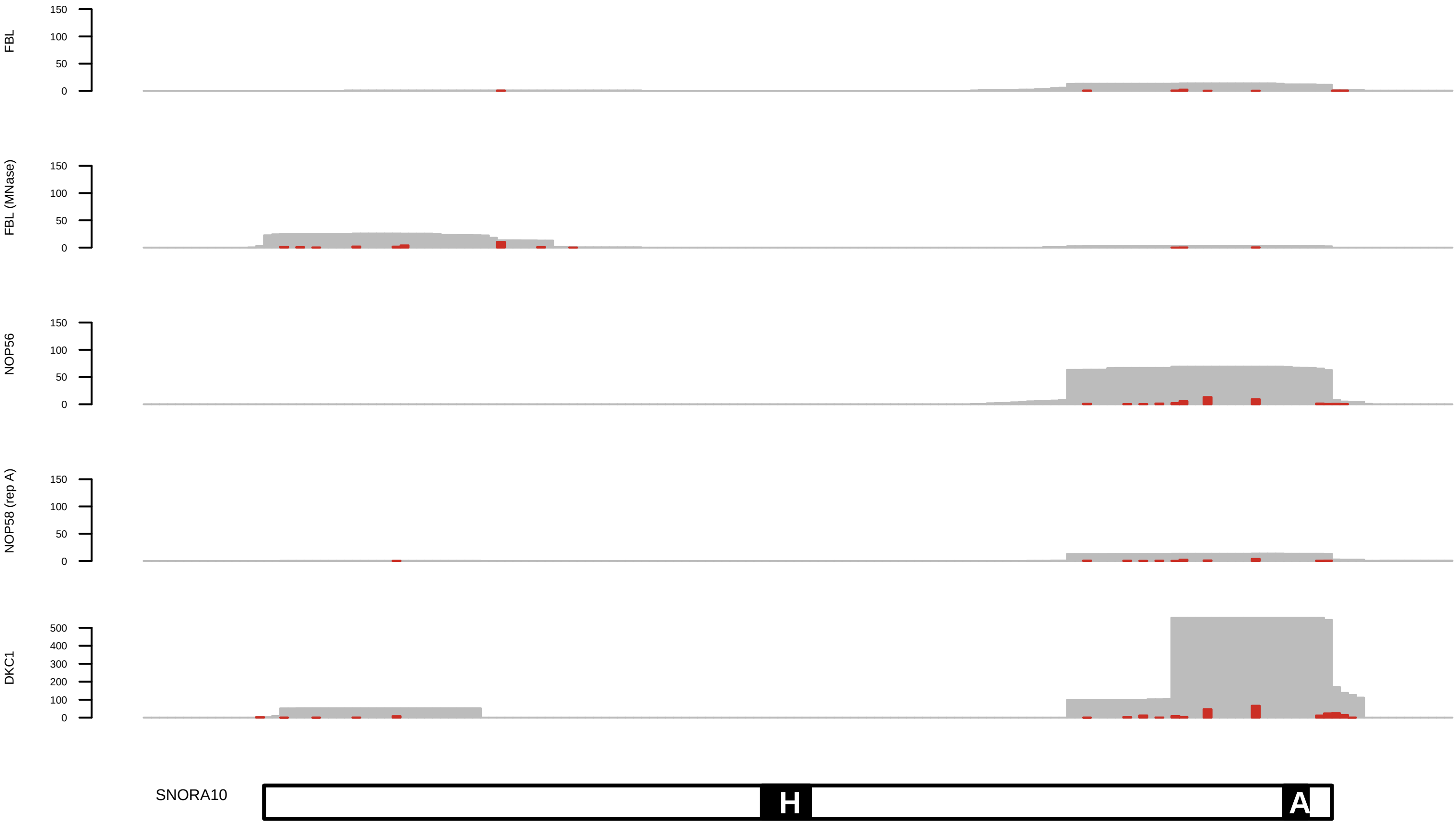
FBL
150
100
50
0
FBL (MNase)
150
100
50
0
NOP56
150
100
50
0
NOP58 (rep A)
150
100
50
0
DKC1
500
400
300
200
100
0
SNORA10
H
A

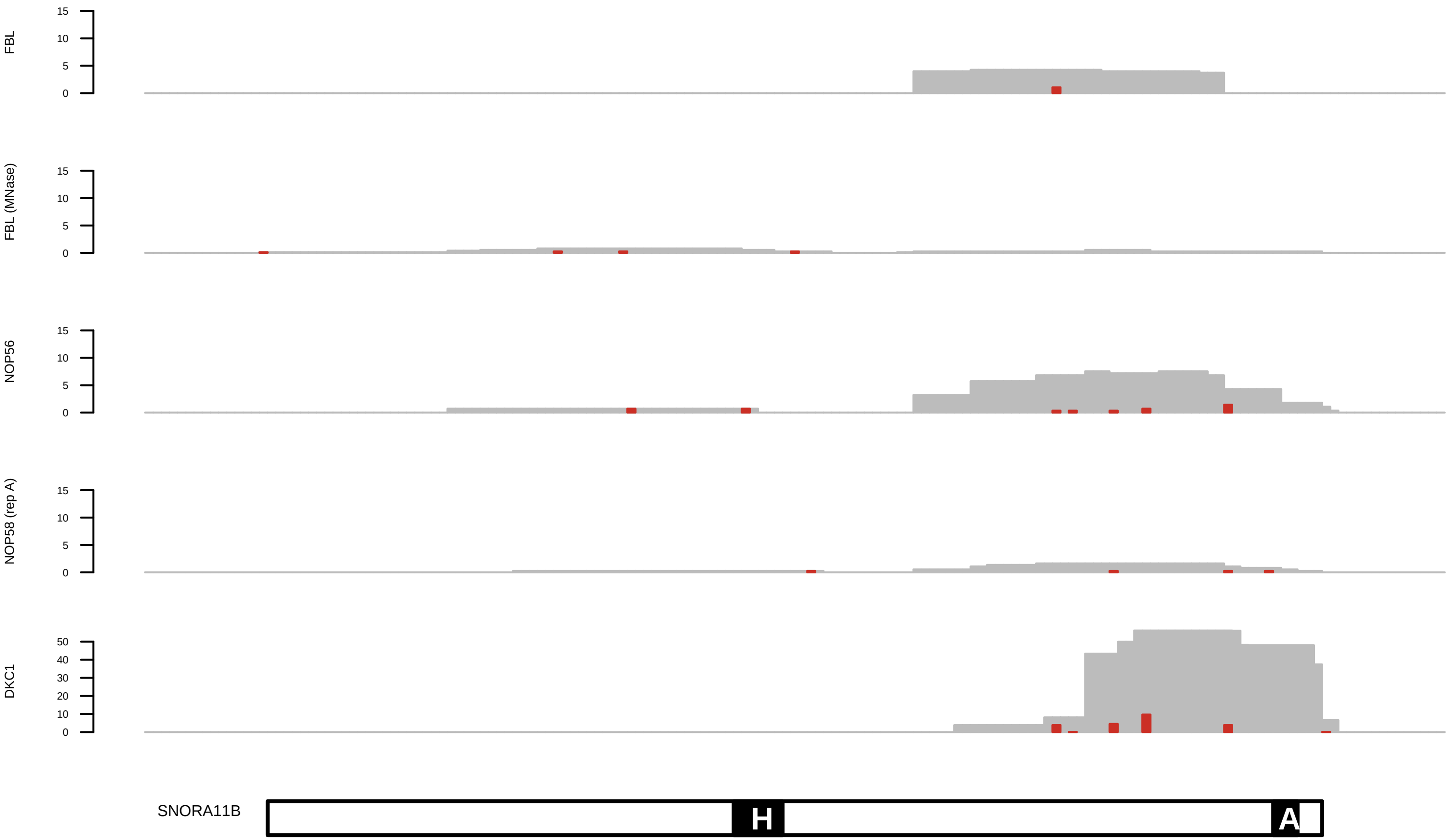

FBL
15
10
5
0
FBL (MNase)
15
10
5
0
NOP56
15
10
5
0
NOP58 (rep A)
15
10
5
0
DKC1
50
40
30
20
10
0
SNORA11B
H
A

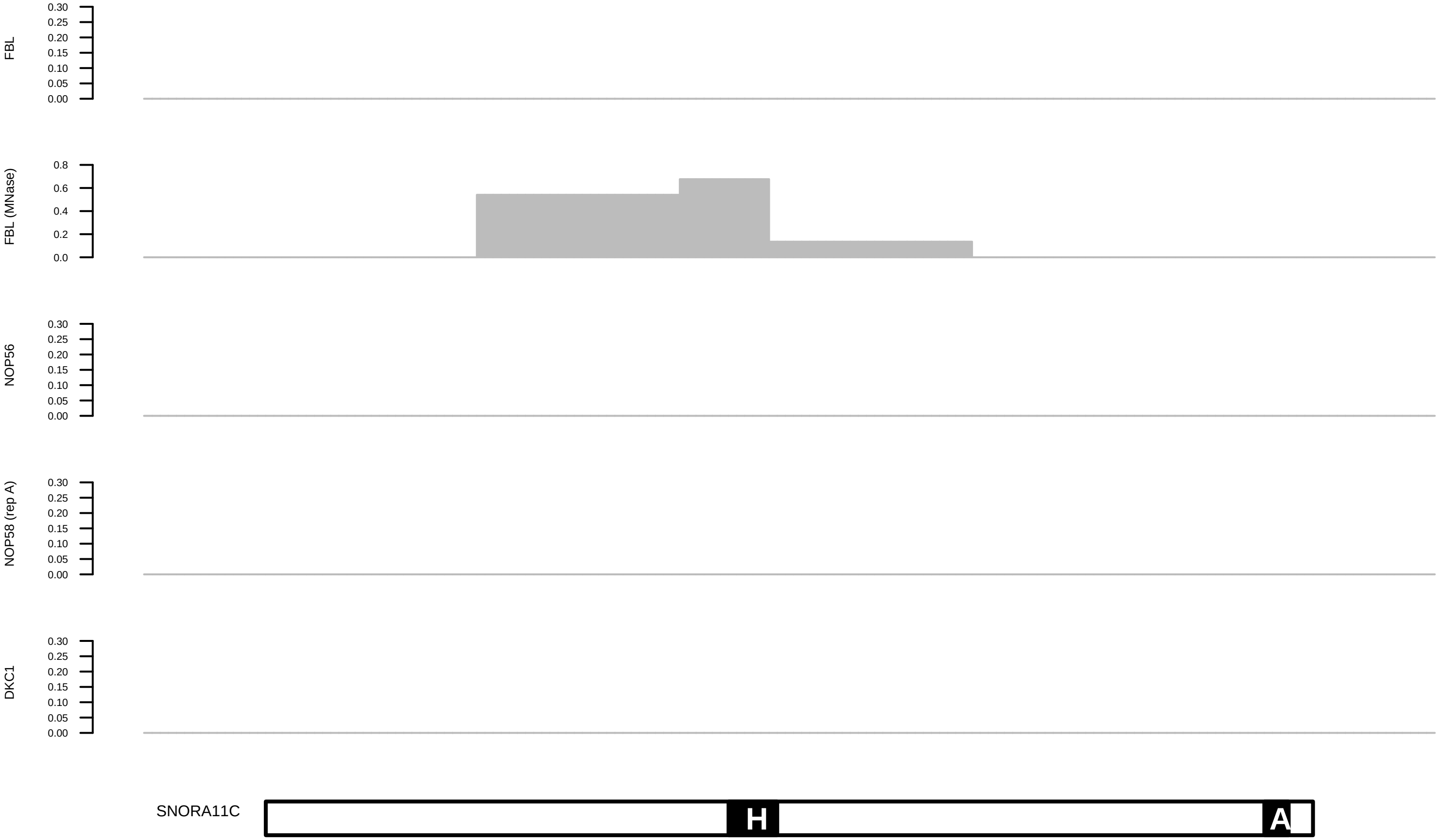
FBL
0.30
0.25
0.20
0.15
0.10
0.05
0.00
FBL (MNase)
0.8
0.6
0.4
0.2
0.0
NOP56
0.30
0.25
0.20
0.15
0.10
0.05
0.00
NOP58 (rep A)
0.30
0.25
0.20
0.15
0.10
0.05
0.00
DKC1
0.30
0.25
0.20
0.15
0.10
0.05
0.00
SNORA11C
H
A

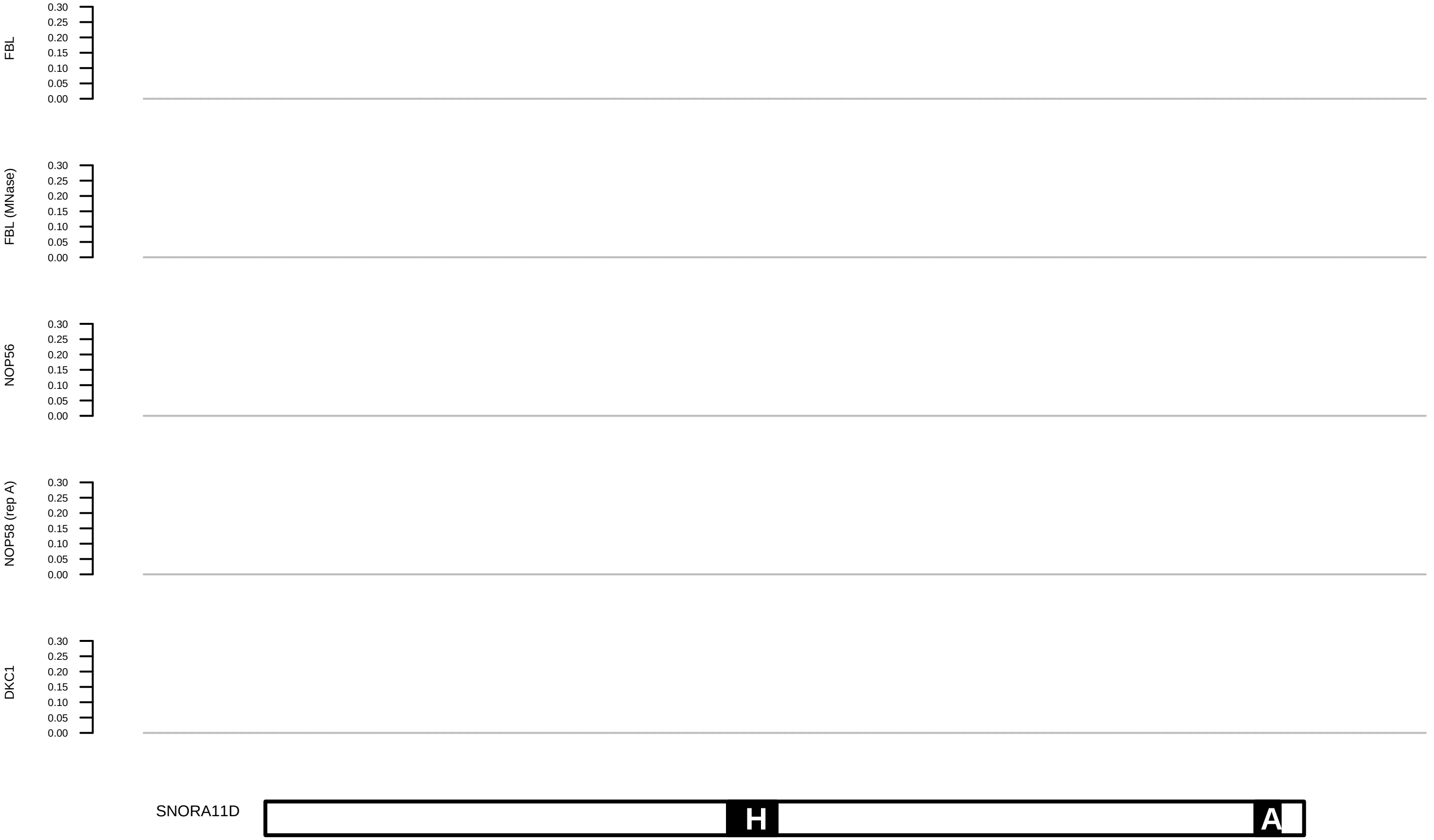
FBL
FBL (MNase)
NOP56
NOP58 (rep A)
DKC1
0.30
0.25
0.20
0.15
0.10
0.05
0.00
SNORA11D
H
A

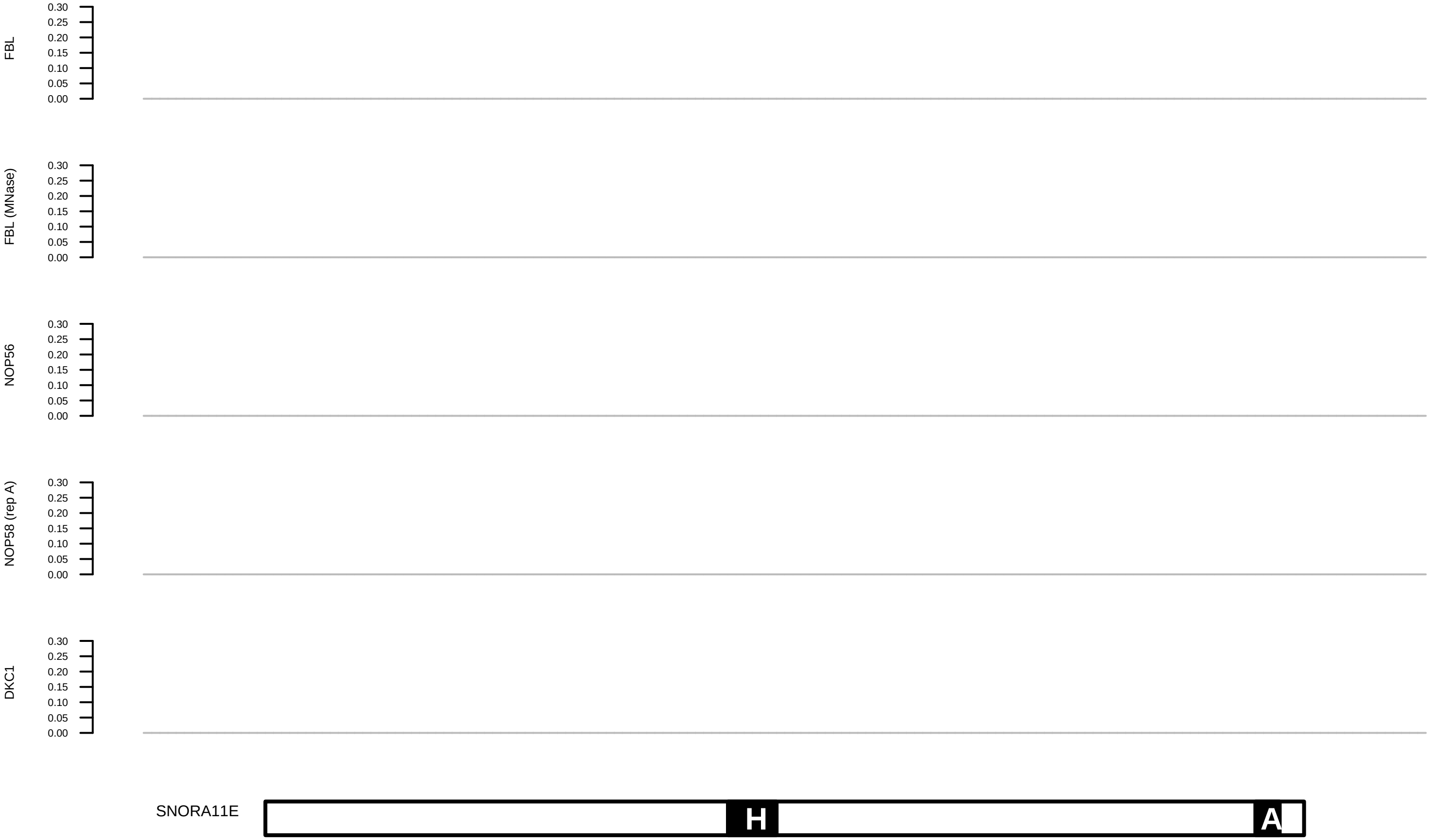
FBL
FBL (MNase)
NOP56
NOP58 (rep A)
DKC1
0.30
0.25
0.20
0.15
0.10
0.05
0.00
SNORA11E
H
A

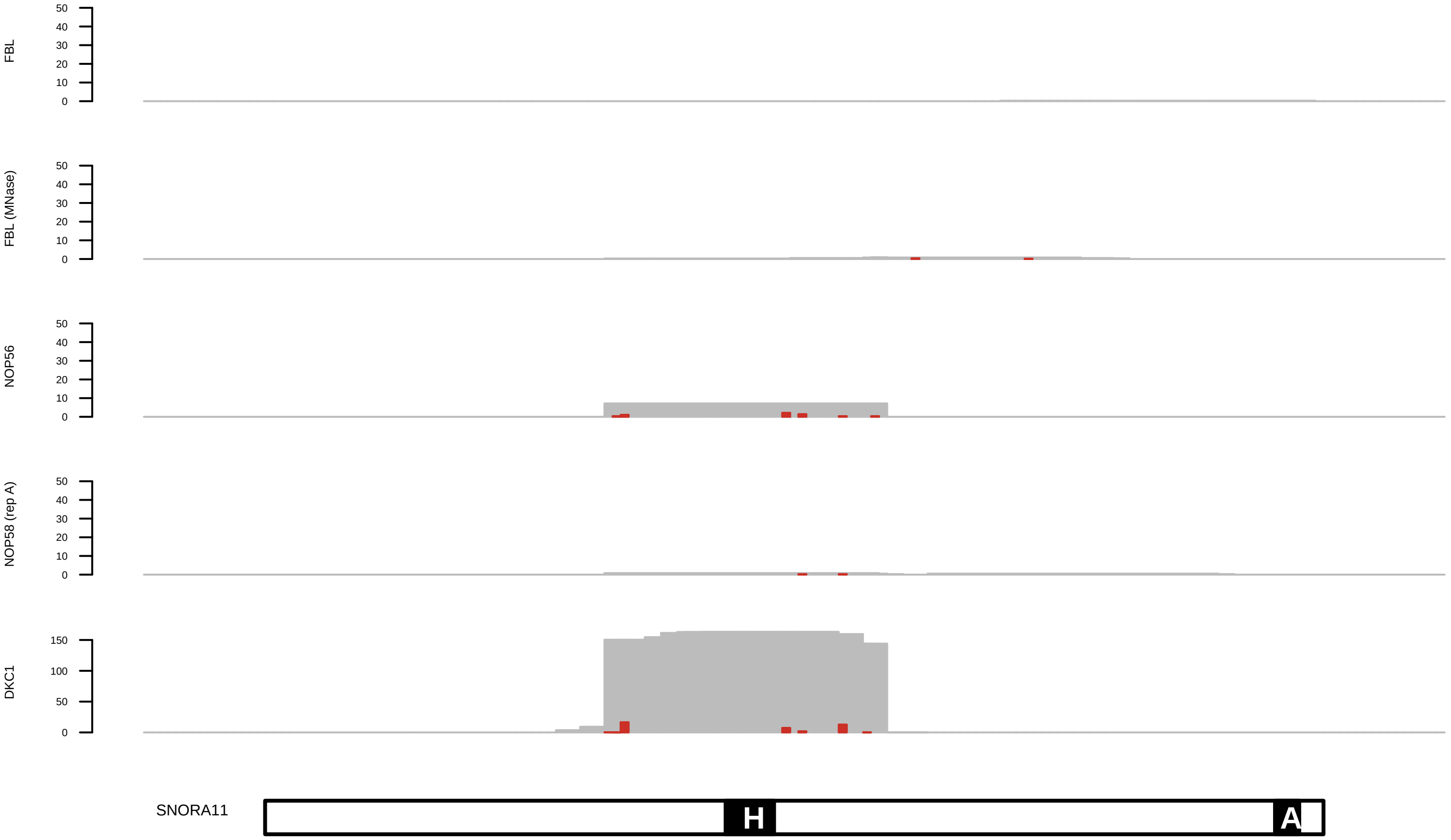
FBL
FBL (MNase)
NOP56
NOP58 (rep A)
DKC1
0
10
20
30
40
50
100
150
SNORA11
H
A

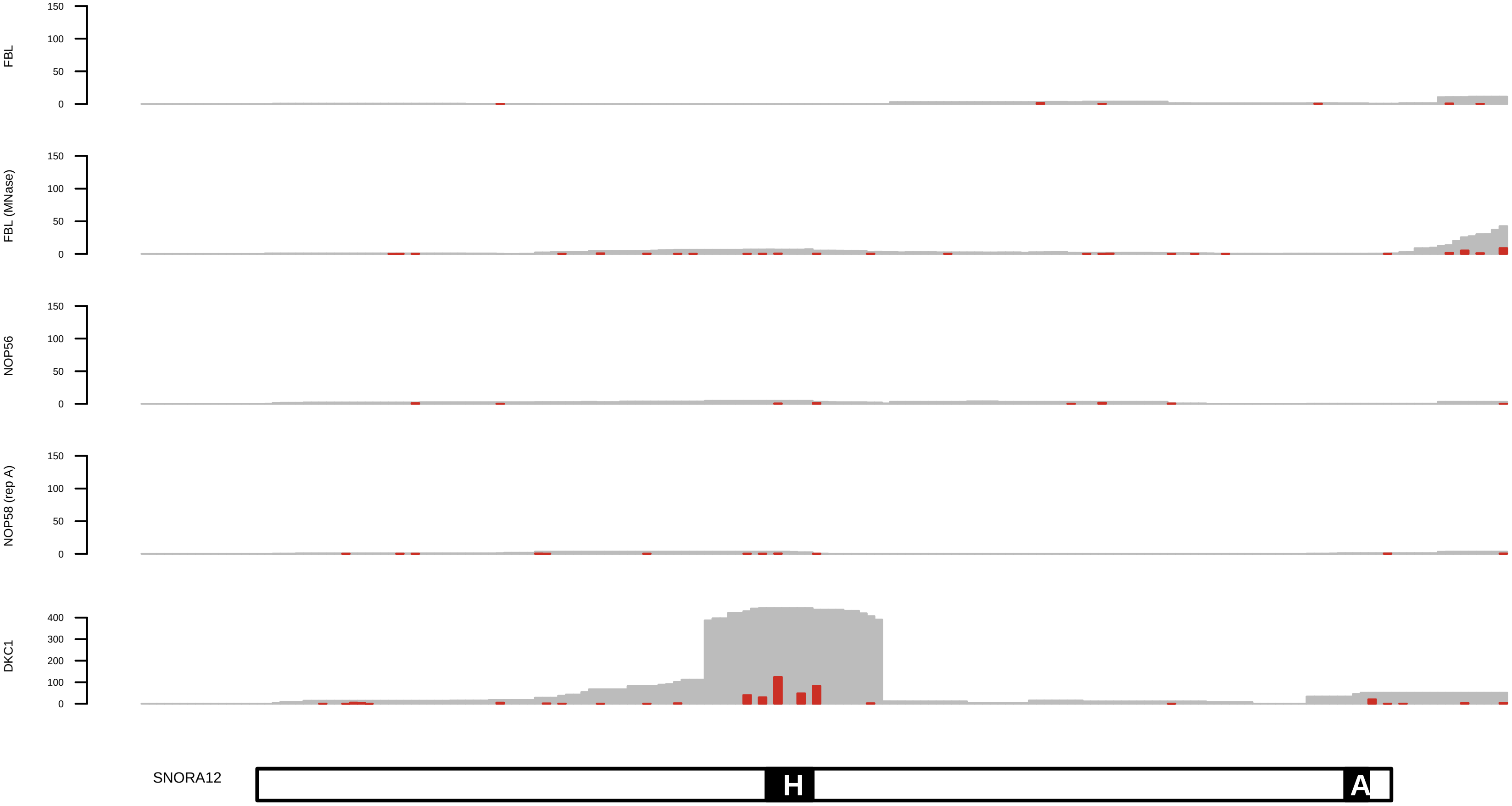

FBL
FBL (MNase)
NOP56
NOP58 (rep A)
DKC1
150
100
50
0
400
300
200
100
SNORA12
H
A

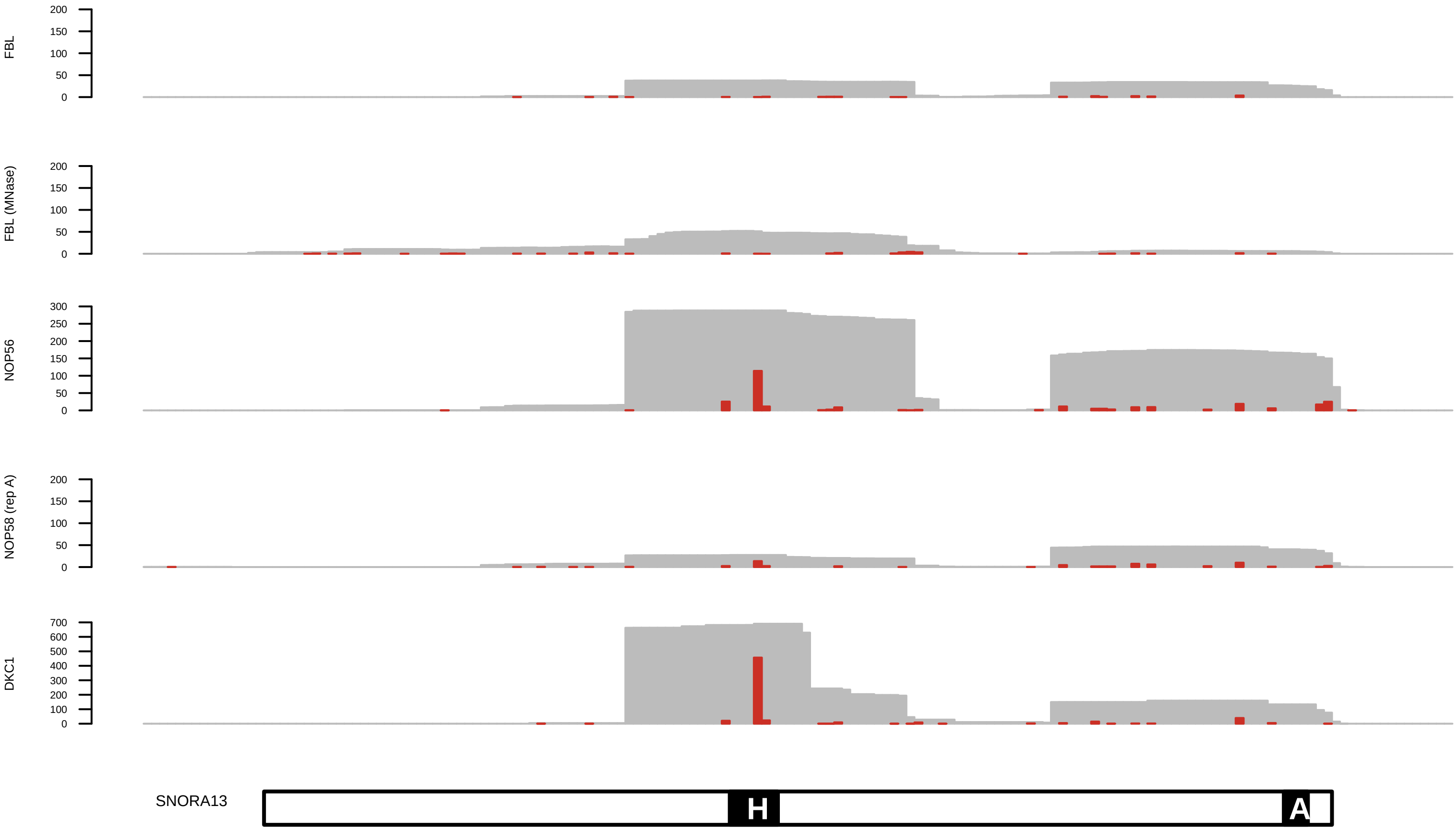

FBL
FBL (MNase)
NOP56
NOP58 (rep A)
DKC1
SNORA13
H
A

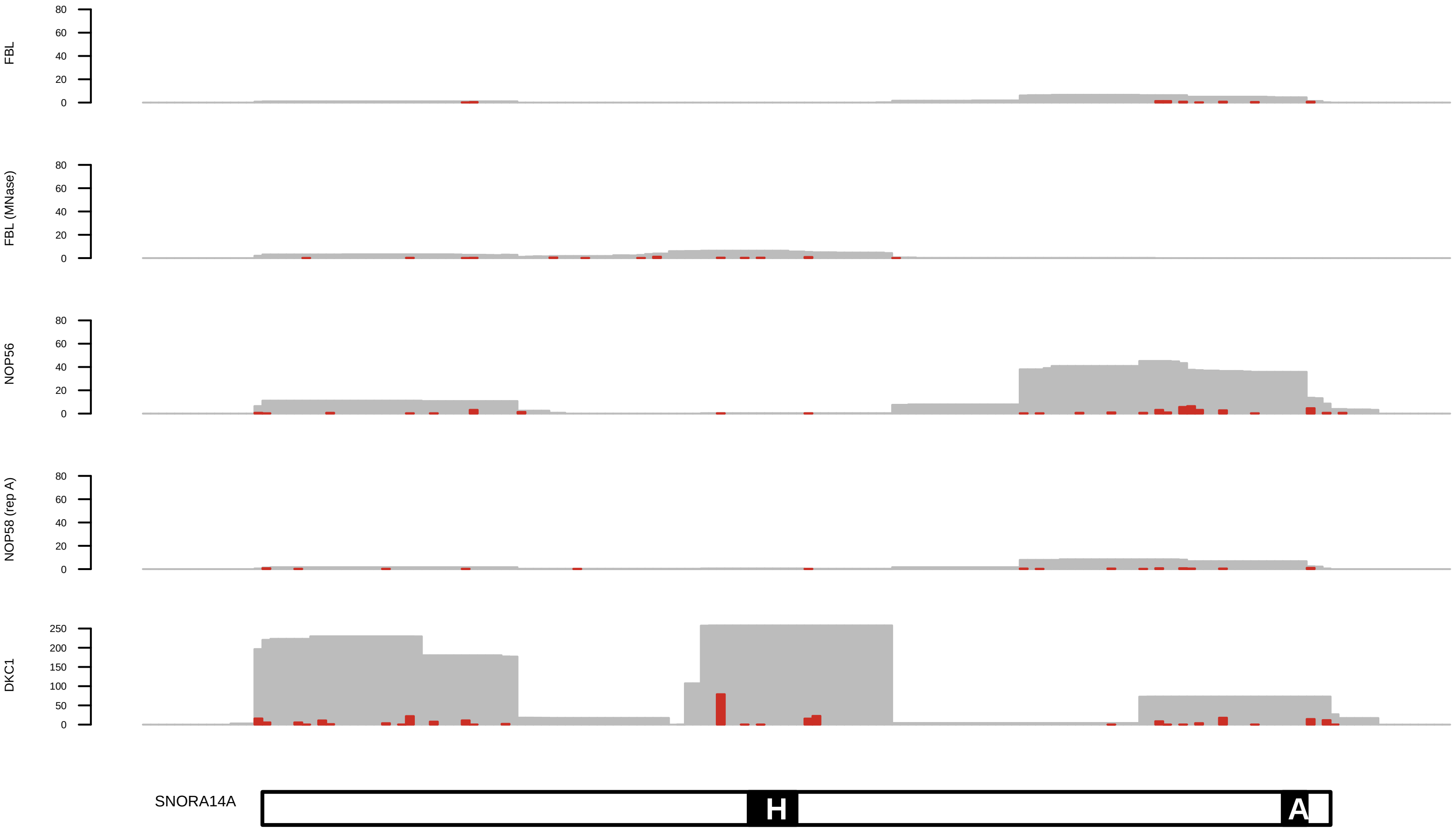
FBL
FBL (MNase)
NOP56
NOP58 (rep A)
DKC1
80
60
40
20
0
250
200
150
100
50
0
SNORA14A
H
A

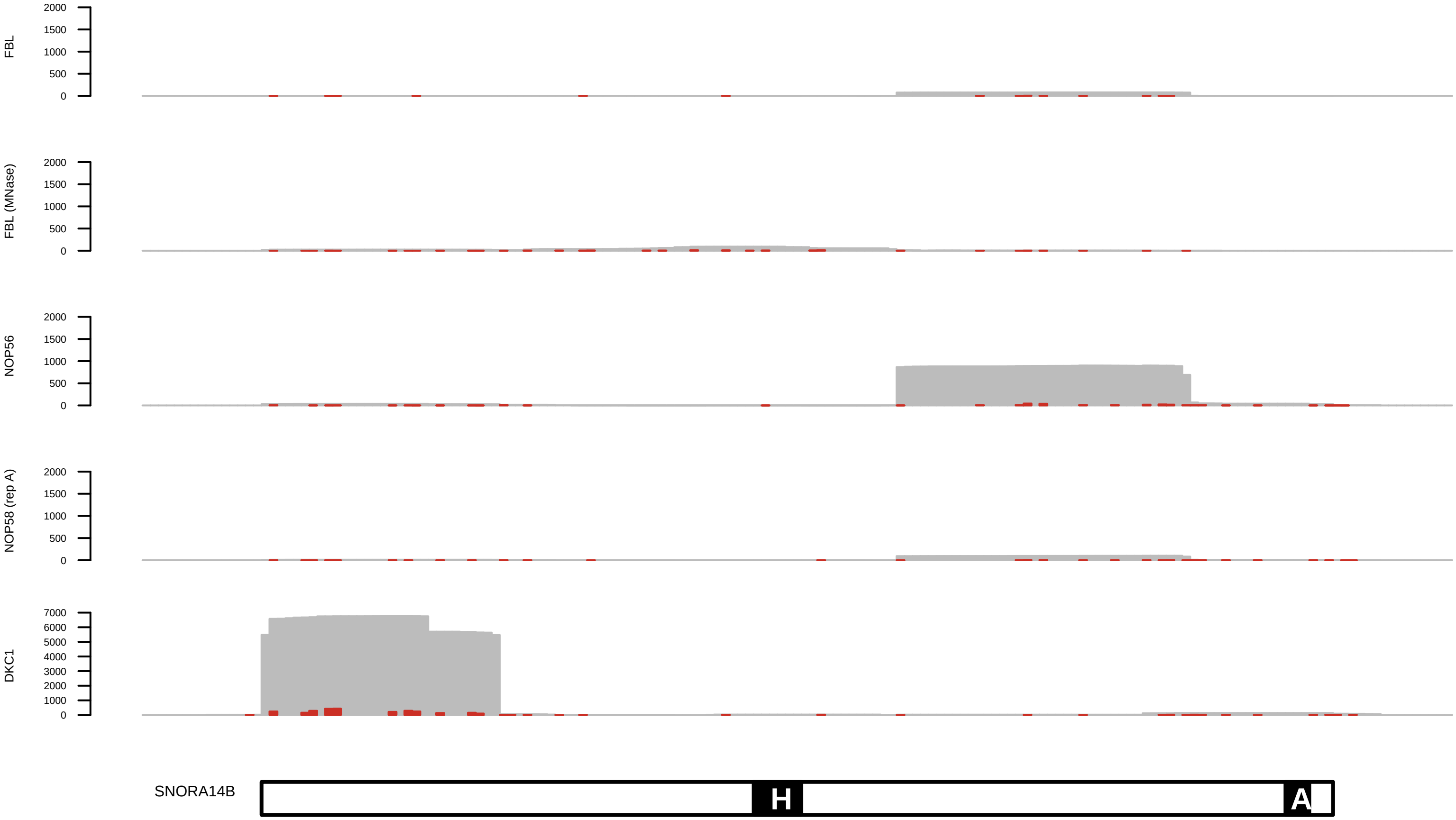
FBL
2000
1500
1000
500
0
FBL (MNase)
2000
1500
1000
500
0
NOP56
2000
1500
1000
500
0
NOP58 (rep A)
2000
1500
1000
500
0
DKC1
7000
6000
5000
4000
3000
2000
1000
0
SNORA14B
H
A

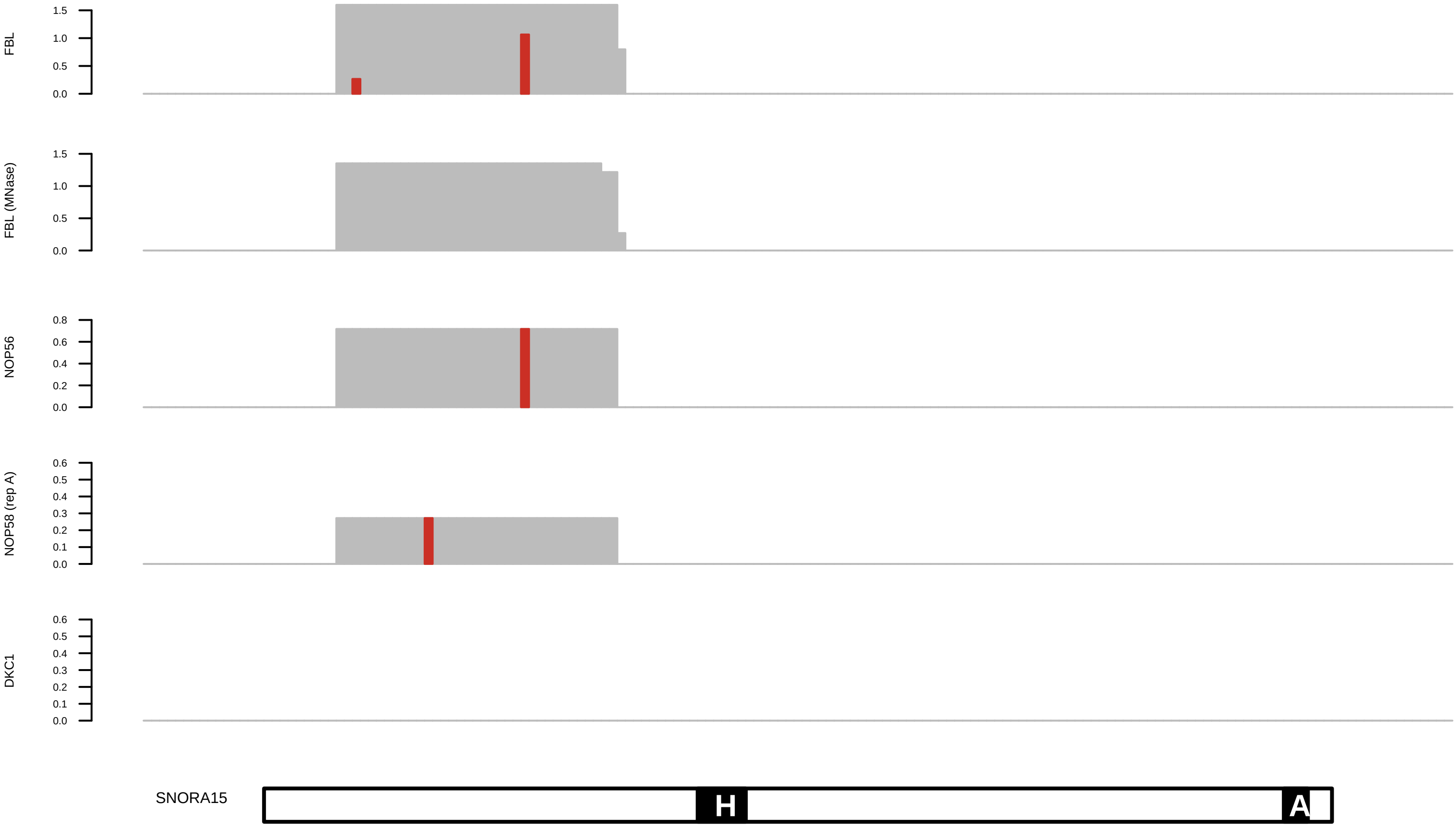
FBL
1.5
1.0
0.5
0.0
FBL (MNase)
1.5
1.0
0.5
0.0
NOP56
0.8
0.6
0.4
0.2
0.0
NOP58 (rep A)
0.6
0.5
0.4
0.3
0.2
0.1
0.0
DKC1
0.6
0.5
0.4
0.3
0.2
0.1
0.0
SNORA15
H
A

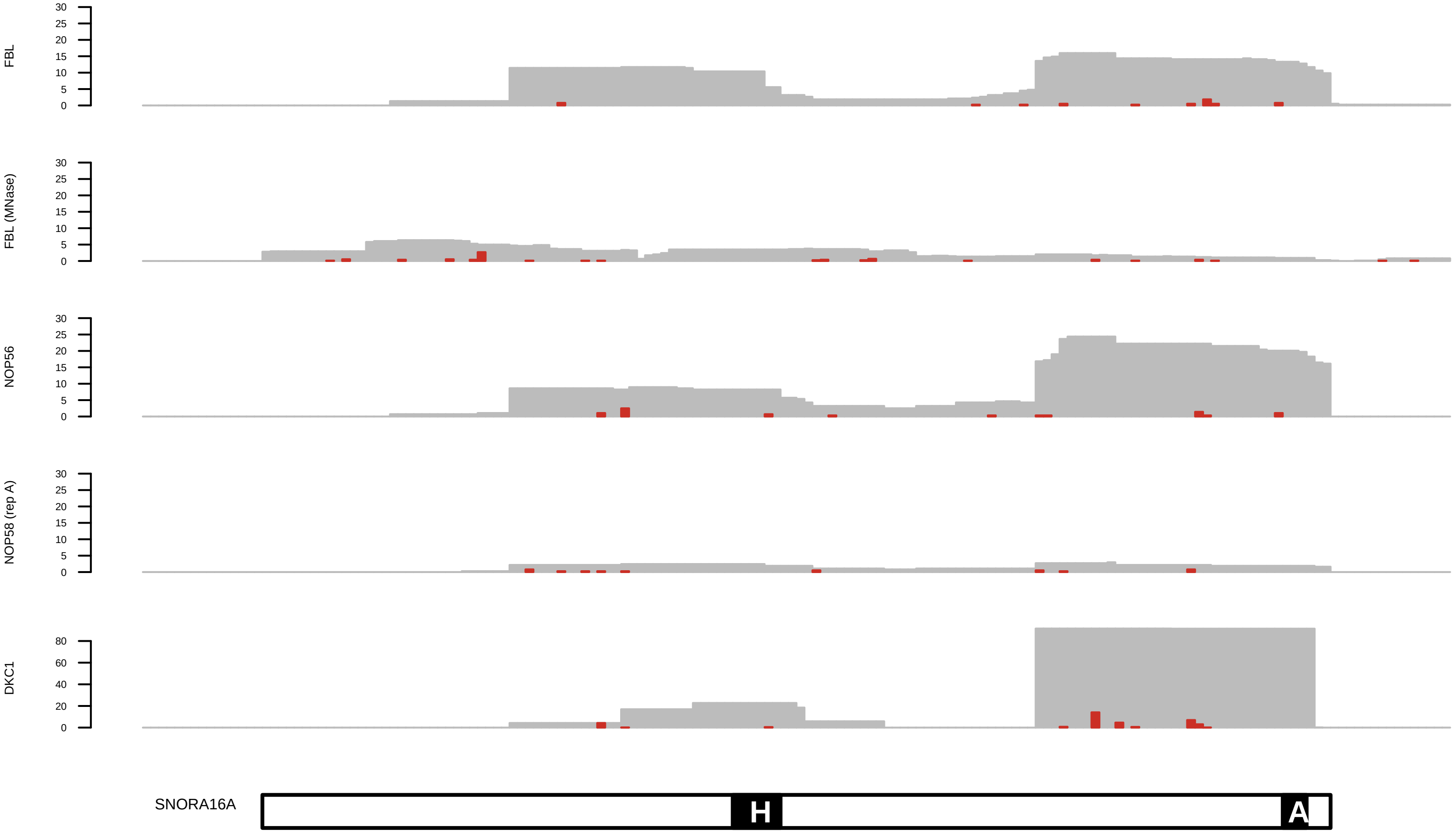
FBL
FBL (MNase)
NOP56
NOP58 (rep A)
DKC1
0
5
10
15
20
25
30
0
20
40
60
80
SNORA16A
H
A

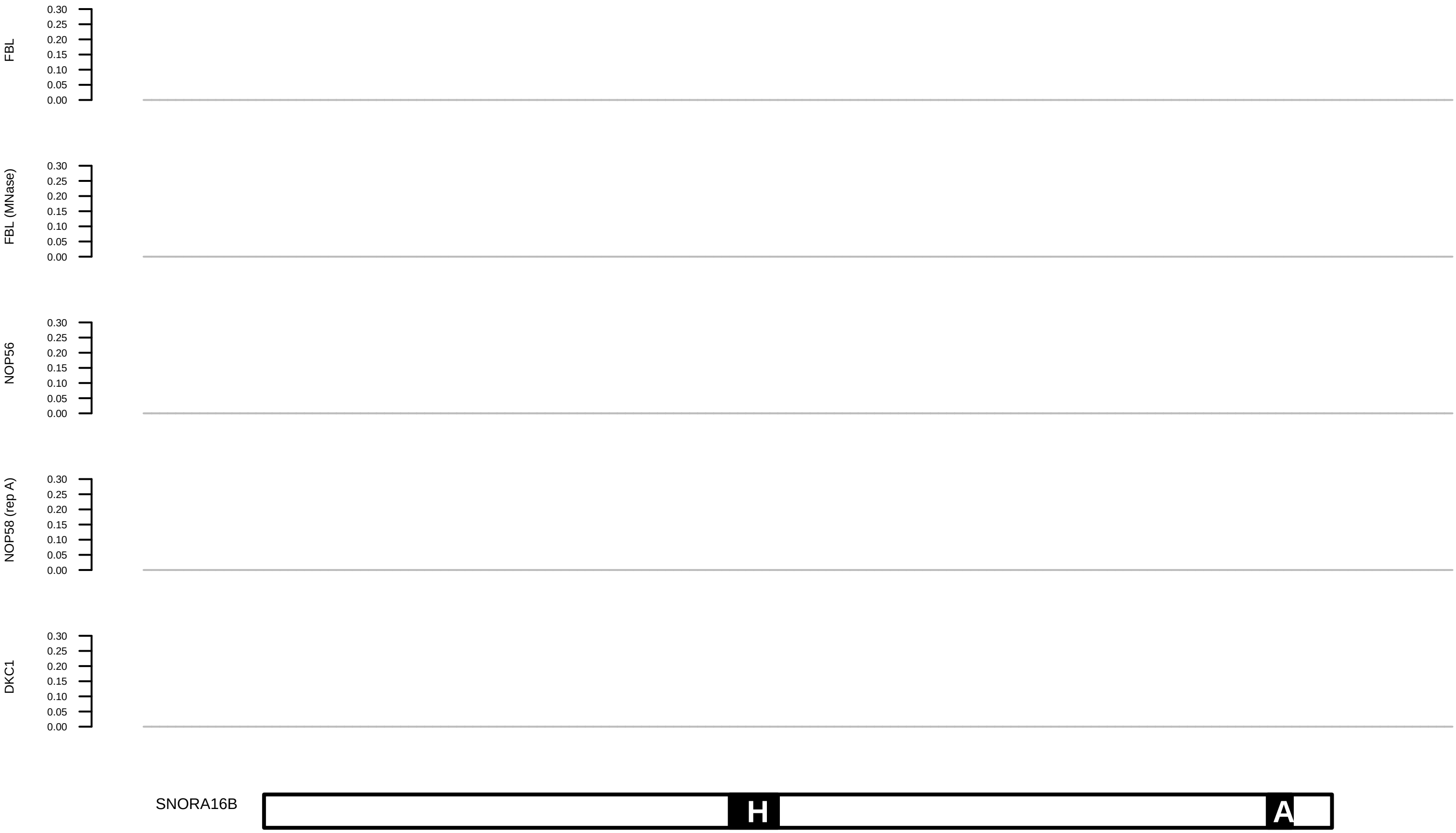
FBL
FBL (MNase)
NOP56
NOP58 (rep A)
DKC1
0.30
0.25
0.20
0.15
0.10
0.05
0.00
SNORA16B
H
A

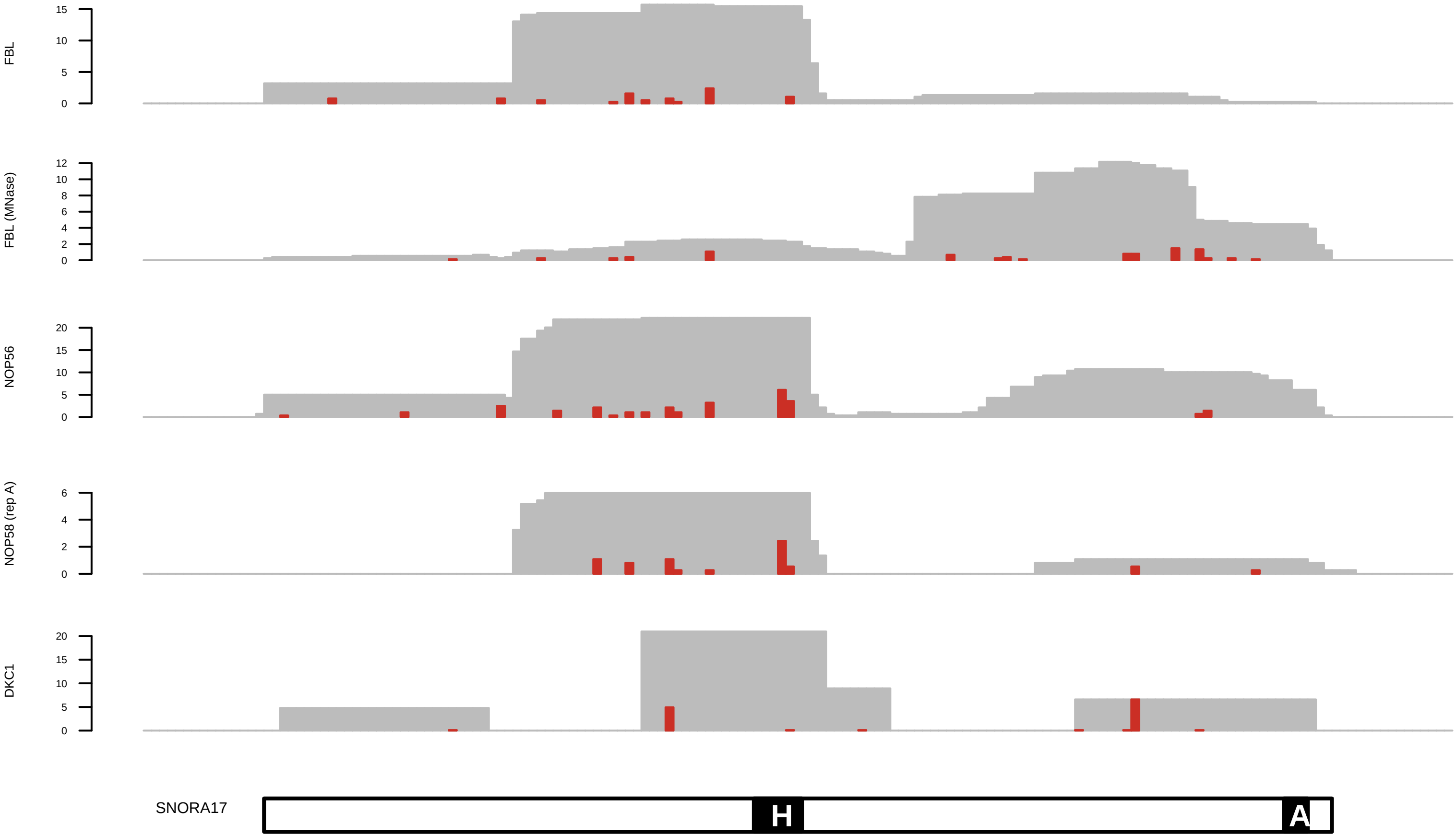
FBL
15
10
5
0
FBL (MNase)
12
10
8
6
4
2
0
NOP56
20
15
10
5
0
NOP58 (rep A)
6
4
2
0
DKC1
20
15
10
5
0
SNORA17
H
A

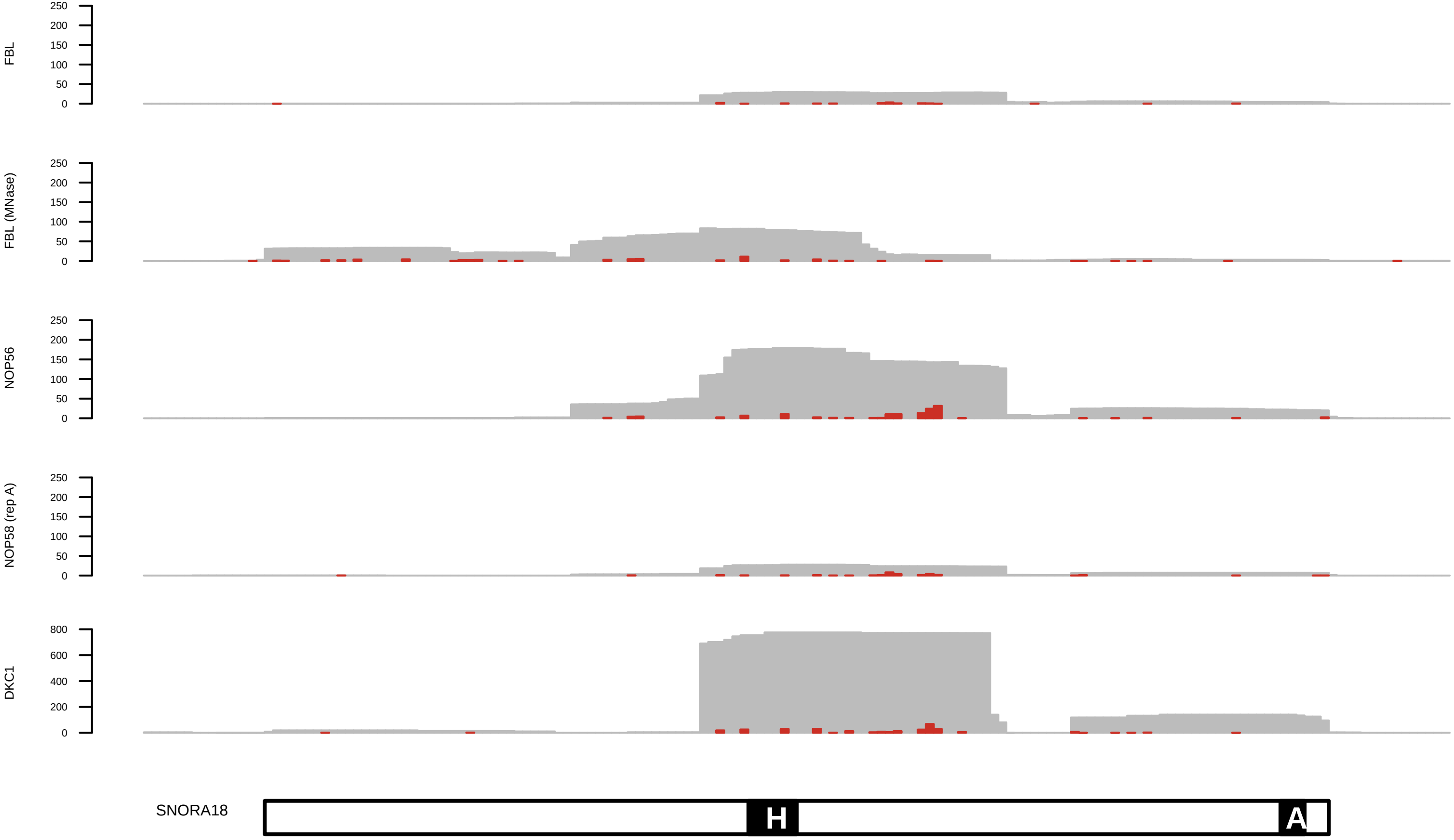
FBL
250
200
150
100
50
0
FBL (MNase)
250
200
150
100
50
0
NOP56
250
200
150
100
50
0
NOP58 (rep A)
250
200
150
100
50
0
DKC1
800
600
400
200
0
SNORA18
H
A

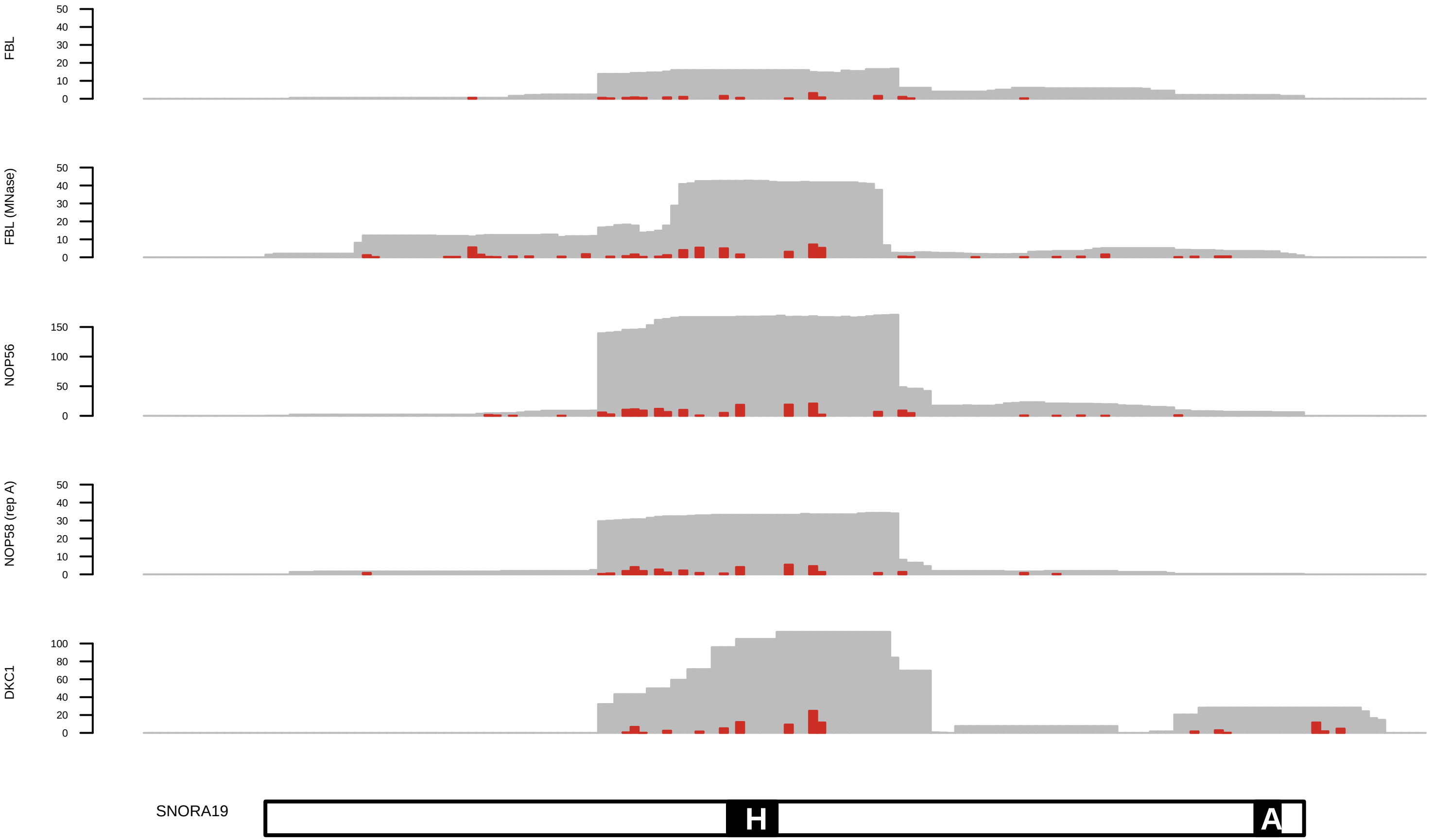

FBL
50
40
30
20
10
0
FBL (MNase)
50
40
30
20
10
0
NOP56
150
100
50
0
NOP58 (rep A)
50
40
30
20
10
0
DKC1
100
80
60
40
20
0
SNORA19
H
A

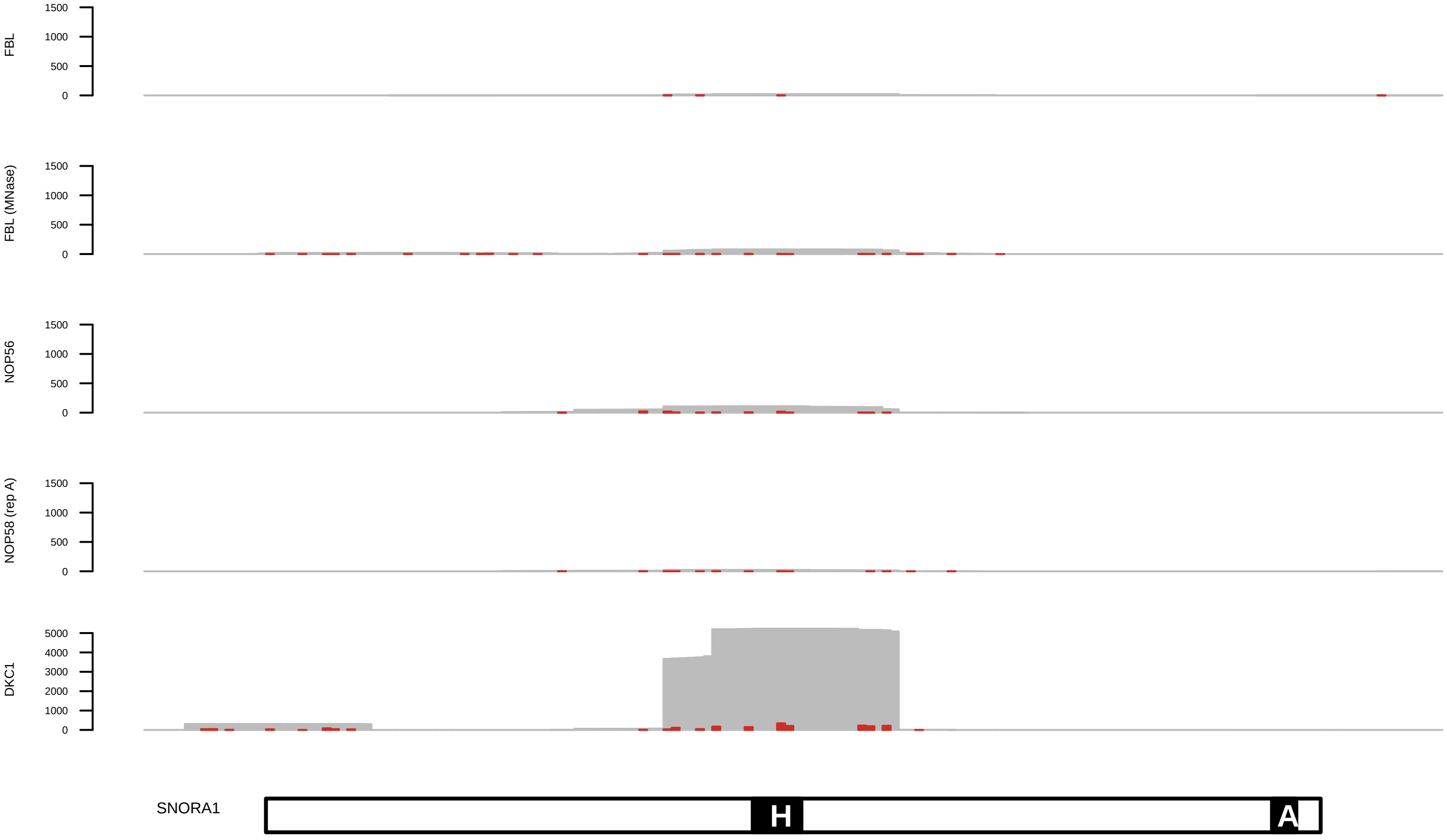
FBL
1500
1000
500
0
FBL (MNase)
1500
1000
500
0
NOP56
1500
1000
500
0
NOP58 (rep A)
1500
1000
500
0
DKC1
5000
4000
3000
2000
1000
0
SNORA1
H
A

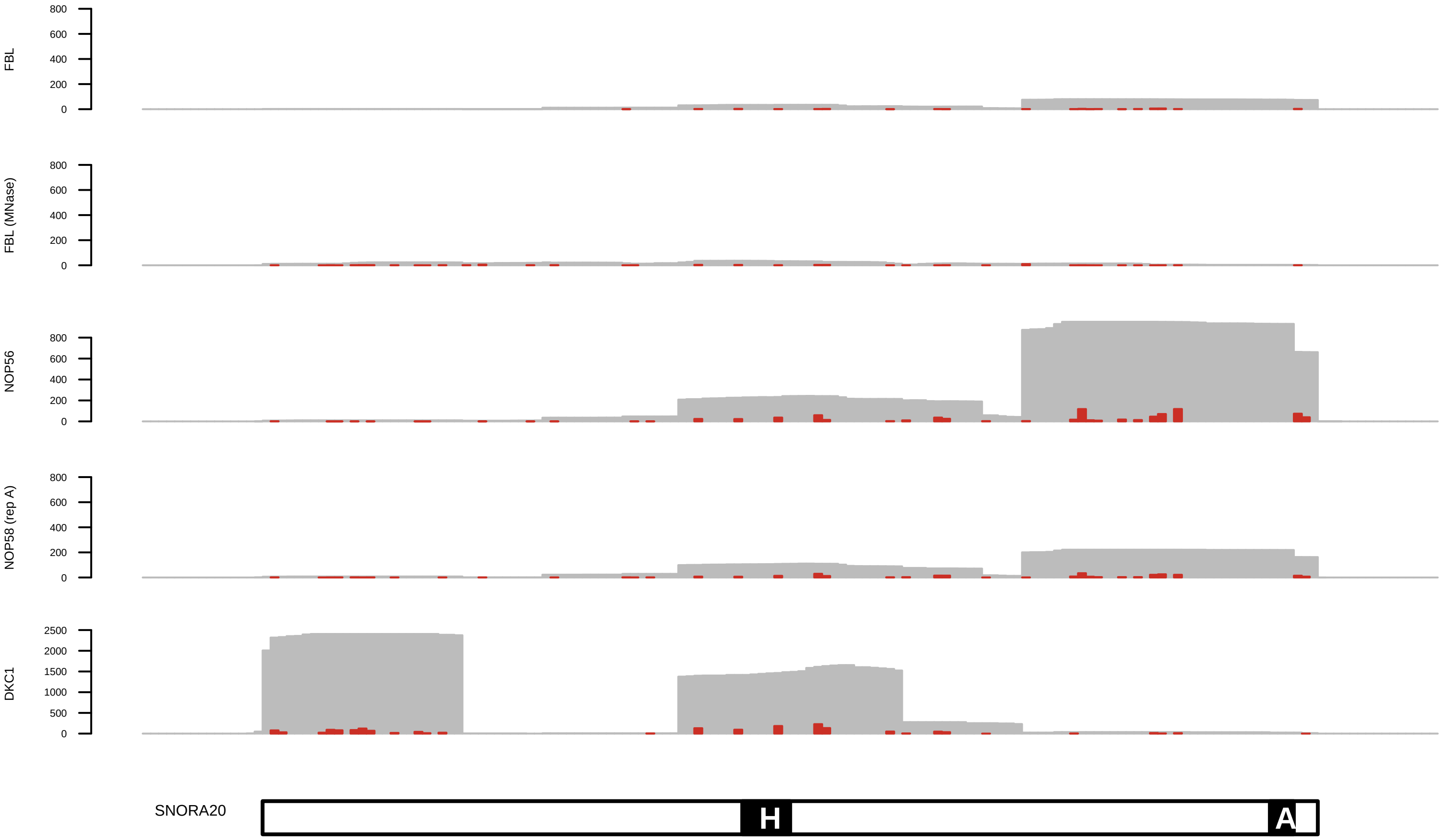
FBL
800
600
400
200
0
FBL (MNase)
800
600
400
200
0
NOP56
800
600
400
200
0
NOP58 (rep A)
800
600
400
200
0
DKC1
2500
2000
1500
1000
500
0
SNORA20
H
A

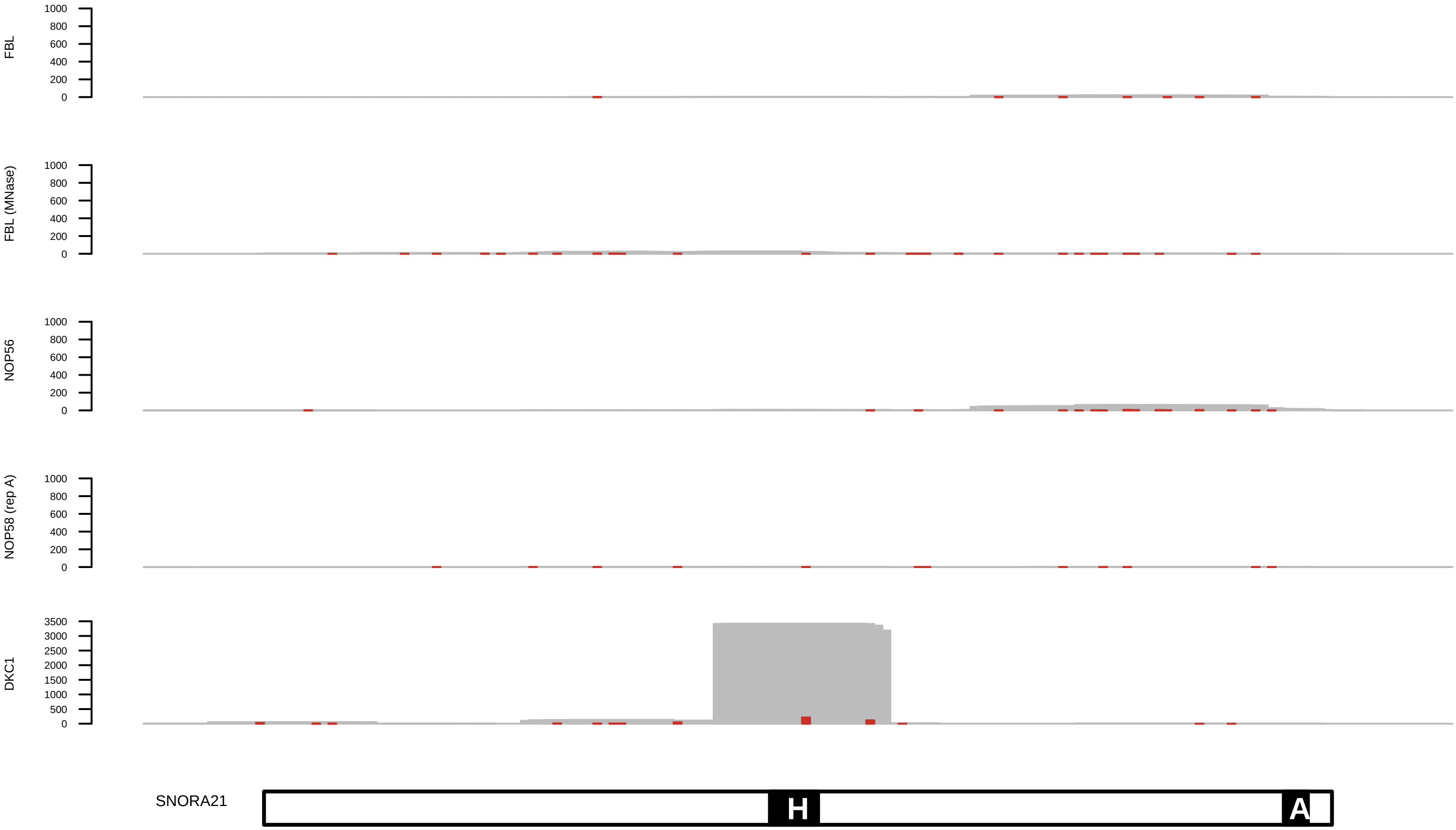
FBL
FBL (MNase)
NOP56
NOP58 (rep A)
DKC1
1000
800
600
400
200
0
3500
3000
2500
2000
1500
1000
500
SNORA21
H
A

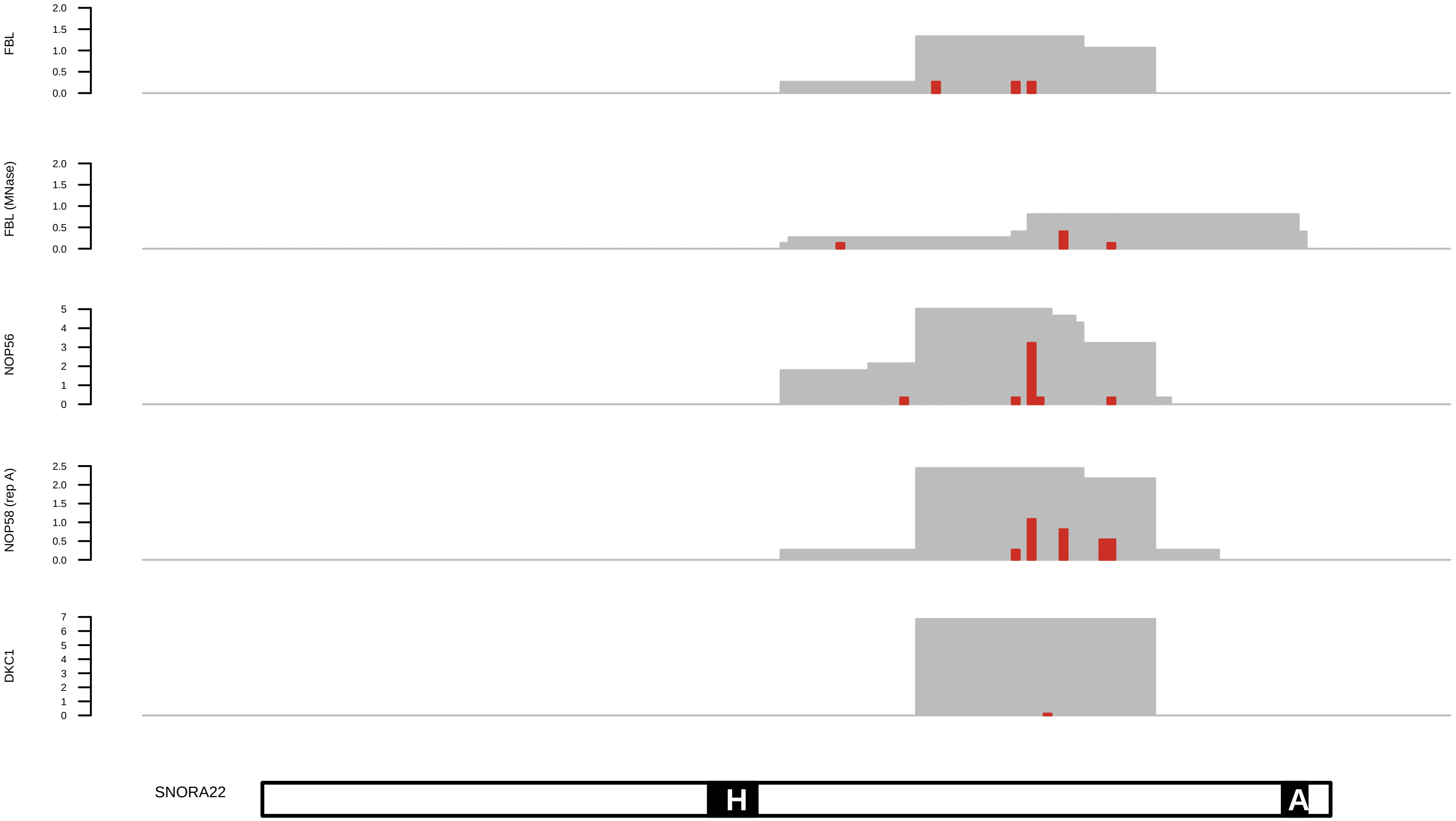

FBL
FBL (MNase)
NOP56
NOP58 (rep A)
DKC1
SNORA22
H
A

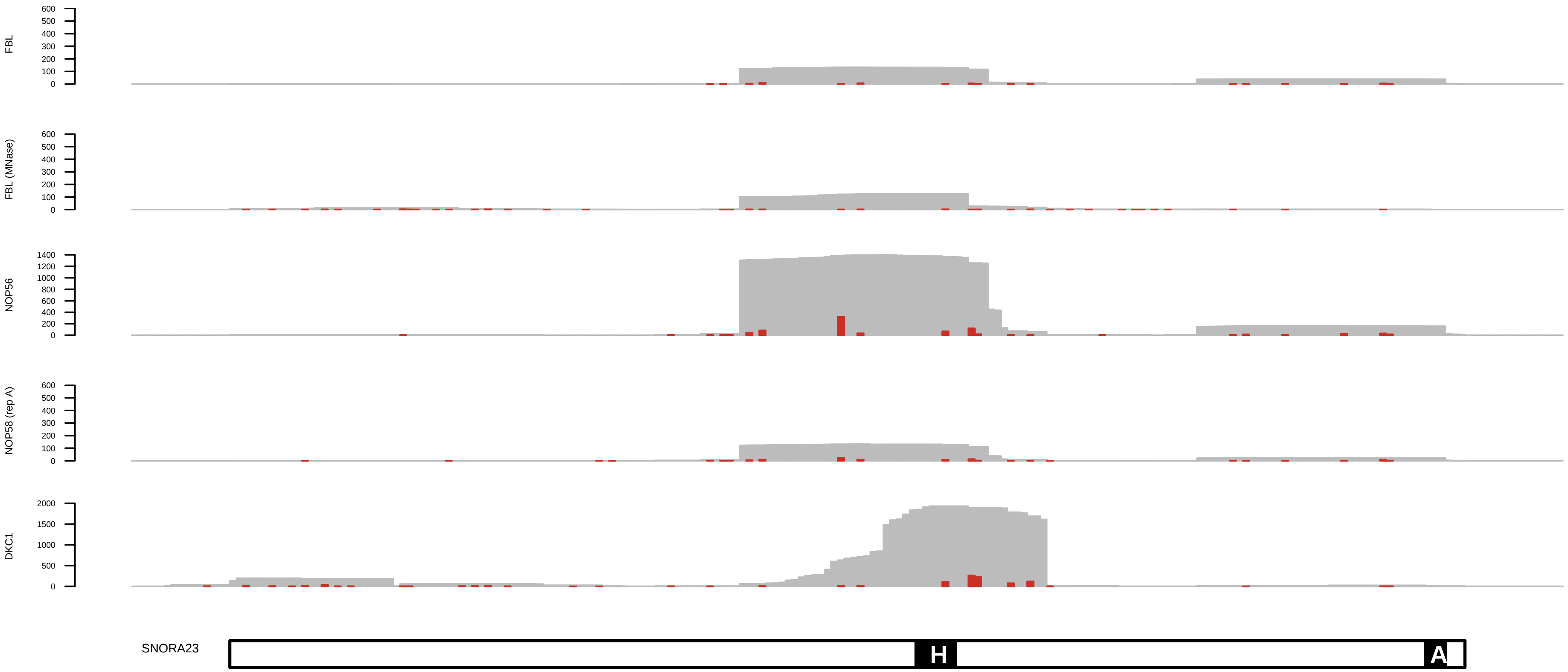

FBL
FBL (MNase)
NOP56
NOP58 (rep A)
DKC1
SNORA23
H
A

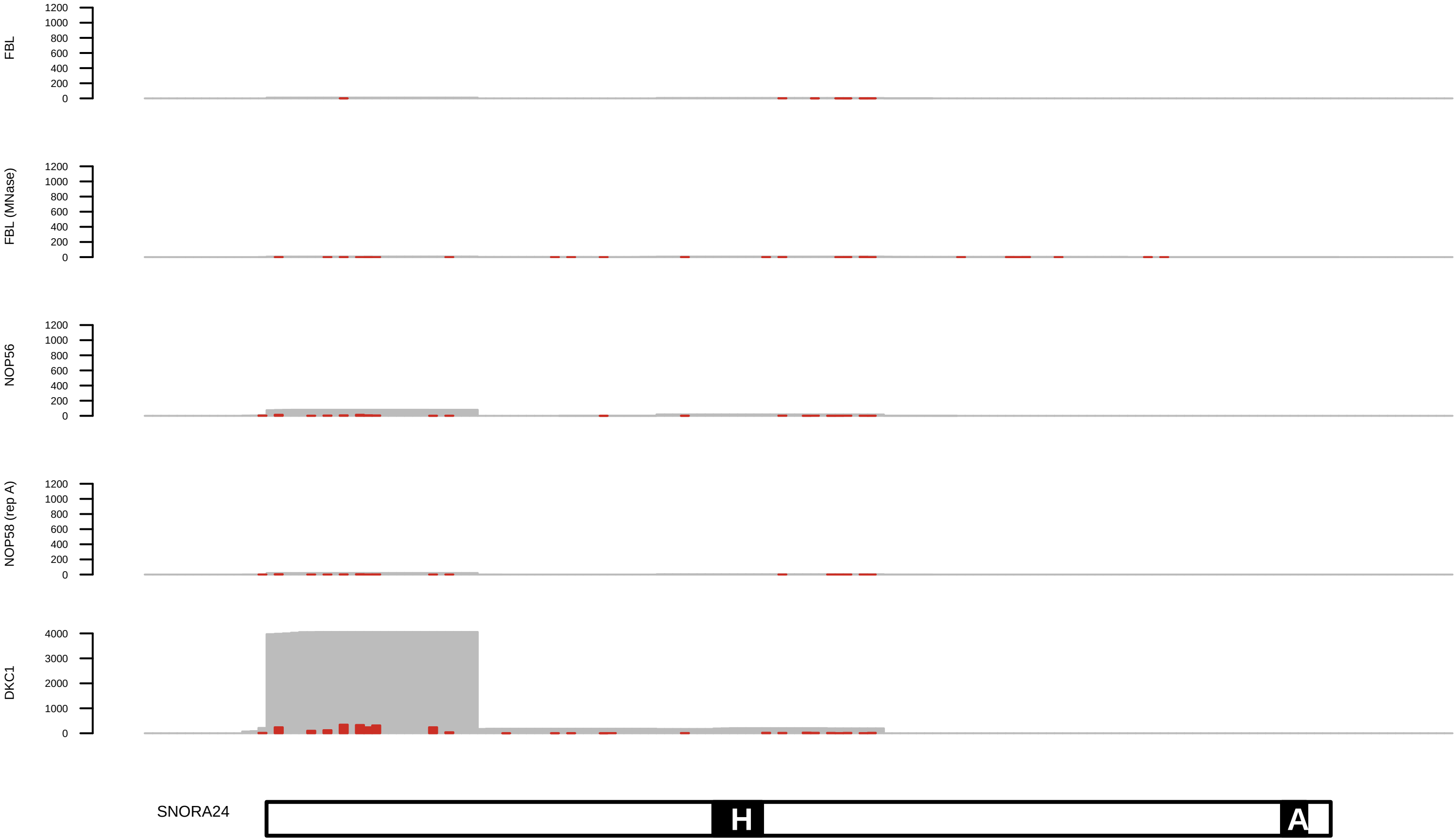

FBL
1200
1000
800
600
400
200
0
FBL (MNase)
NOP56
NOP58 (rep A)
DKC1
4000
3000
2000
1000
SNORA24
H
A

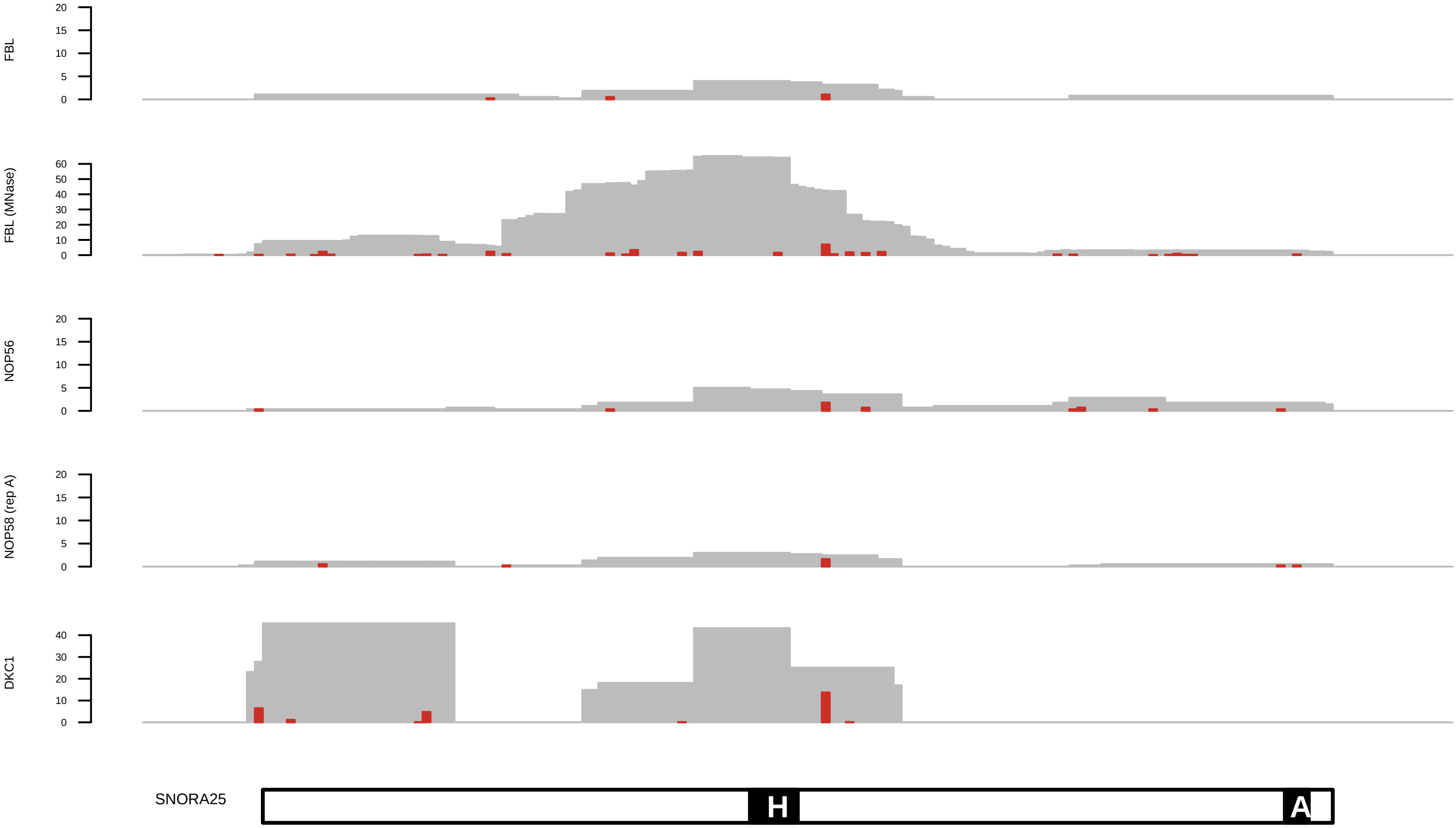
FBL
0
5
10
15
20
FBL (MNase)
0
10
20
30
40
50
60
NOP56
0
5
10
15
20
NOP58 (rep A)
0
5
10
15
20
DKC1
0
10
20
30
40
SNORA25
H
A

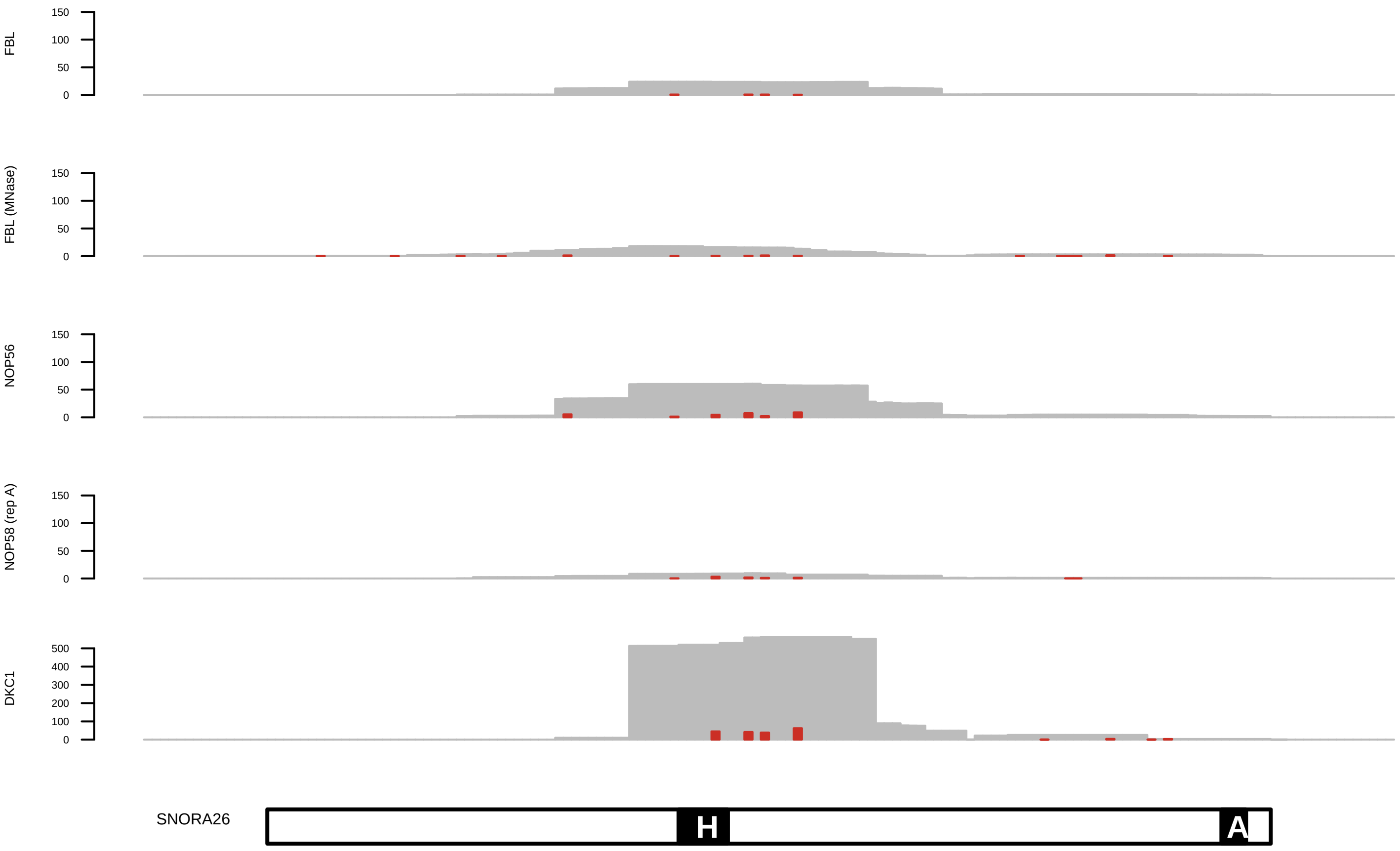

FBL
150
100
50
0
FBL (MNase)
150
100
50
0
NOP56
150
100
50
0
NOP58 (rep A)
150
100
50
0
DKC1
500
400
300
200
100
0
SNORA26
H
A

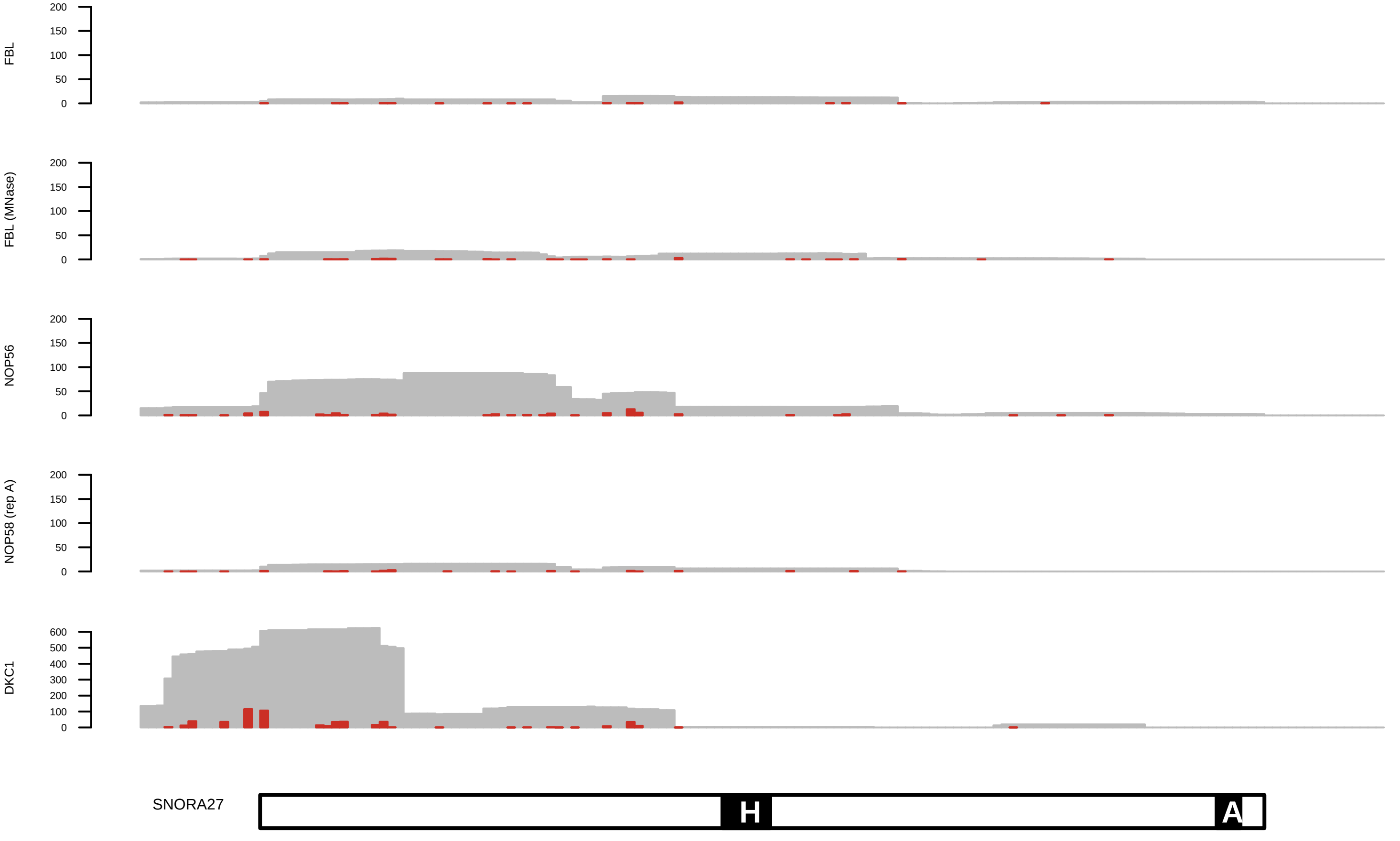
FBL
FBL (MNase)
NOP56
NOP58 (rep A)
DKC1
200
150
100
50
0
600
500
400
300
200
100
SNORA27
H
A

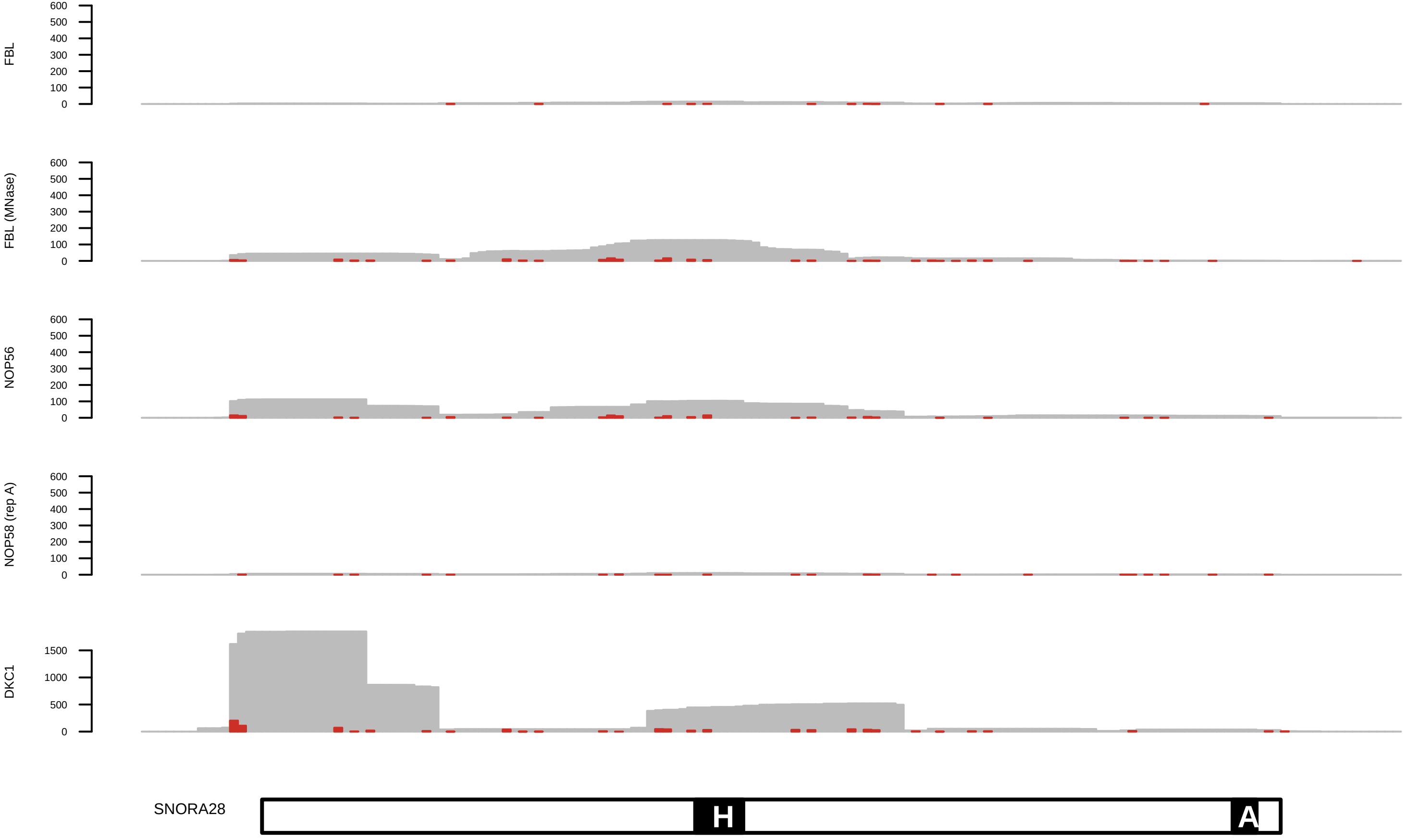
FBL
FBL (MNase)
NOP56
NOP58 (rep A)
DKC1
0
100
200
300
400
500
600
500
1000
1500
SNORA28
H
A

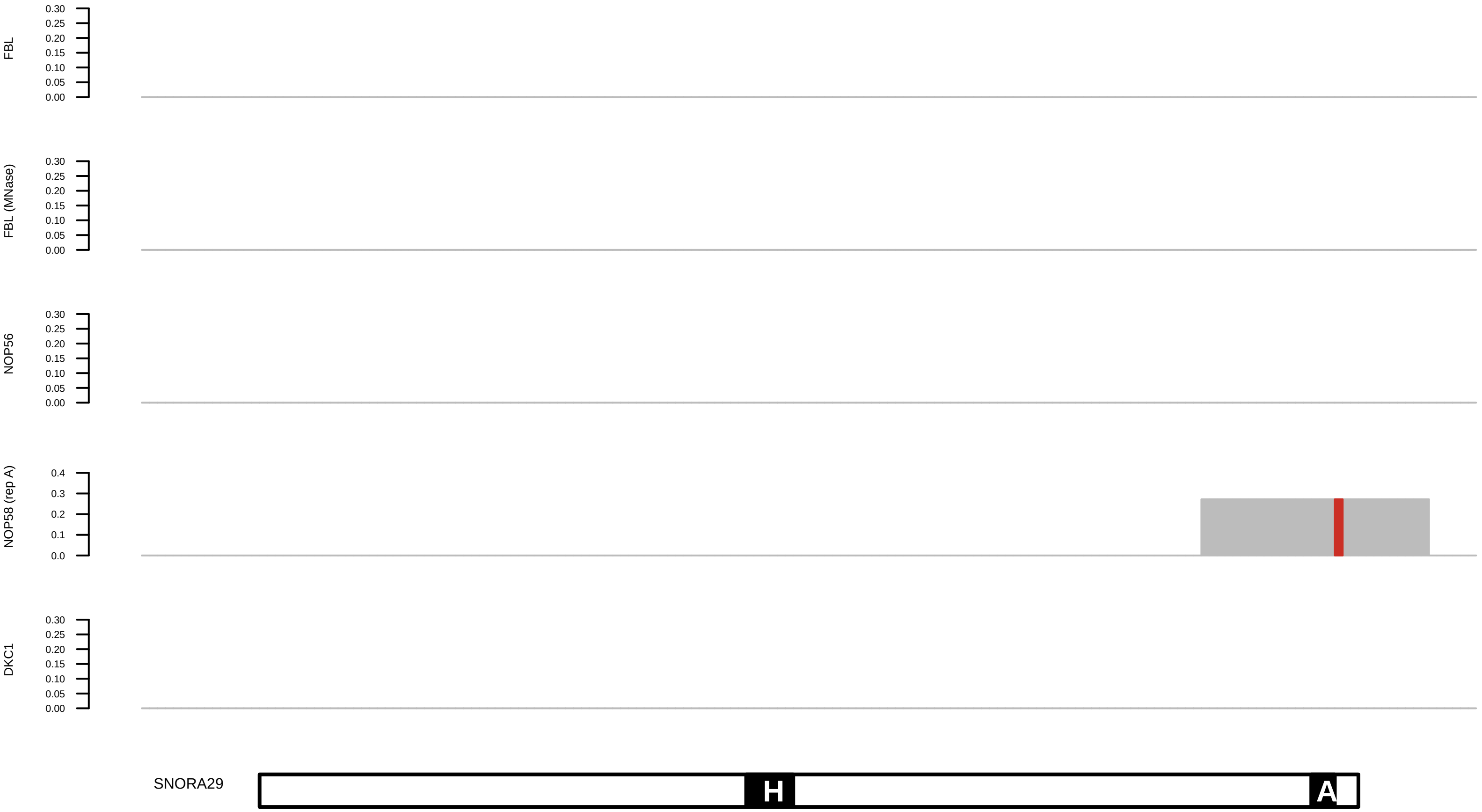

FBL
0.30
0.25
0.20
0.15
0.10
0.05
0.00
FBL (MNase)
0.30
0.25
0.20
0.15
0.10
0.05
0.00
NOP56
0.30
0.25
0.20
0.15
0.10
0.05
0.00
NOP58 (rep A)
0.4
0.3
0.2
0.1
0.0
DKC1
0.30
0.25
0.20
0.15
0.10
0.05
0.00
SNORA29
H
A

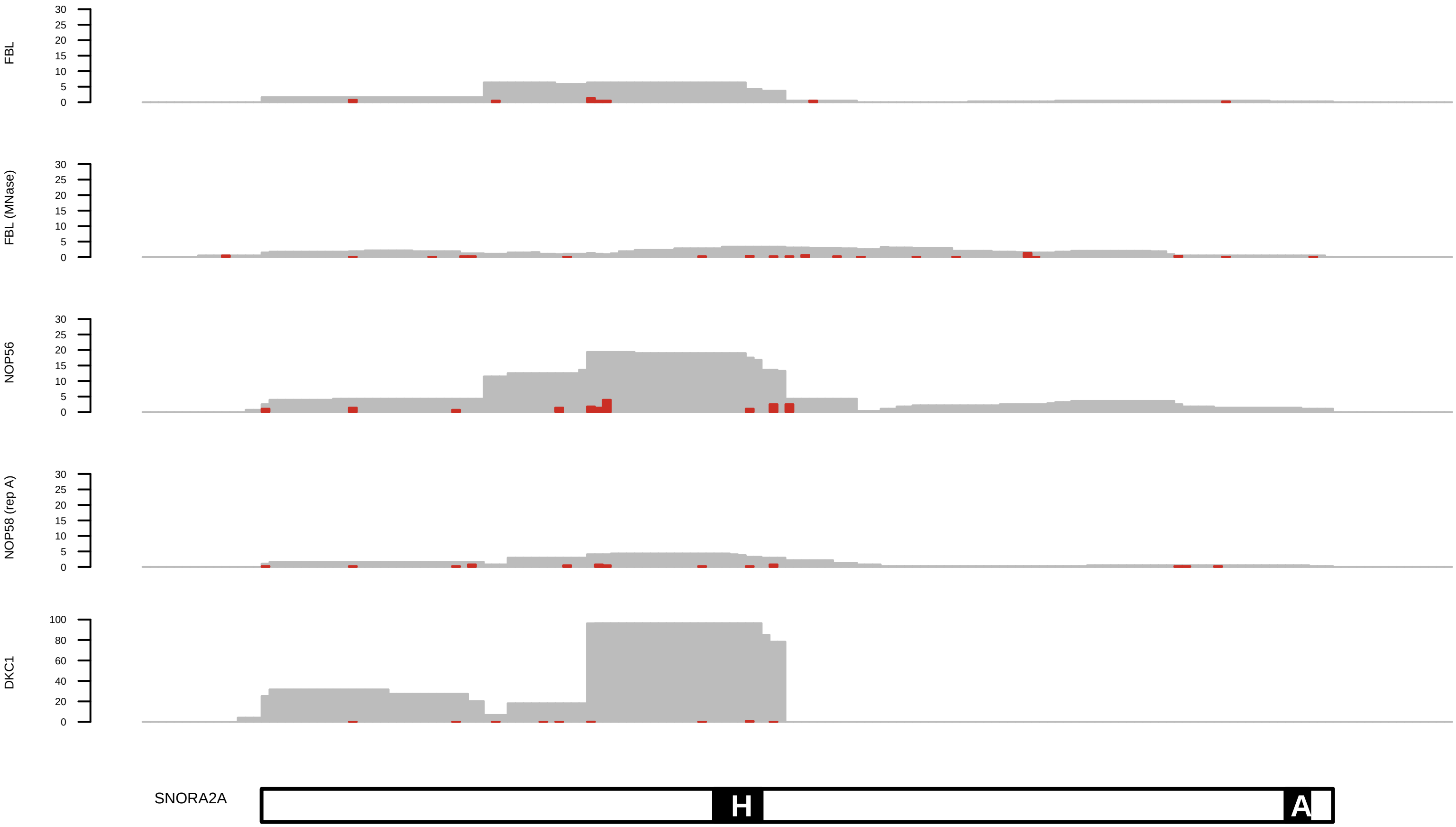
FBL
FBL (MNase)
NOP56
NOP58 (rep A)
DKC1
30
25
20
15
10
5
0
100
80
60
40
20
SNORA2A
H
A

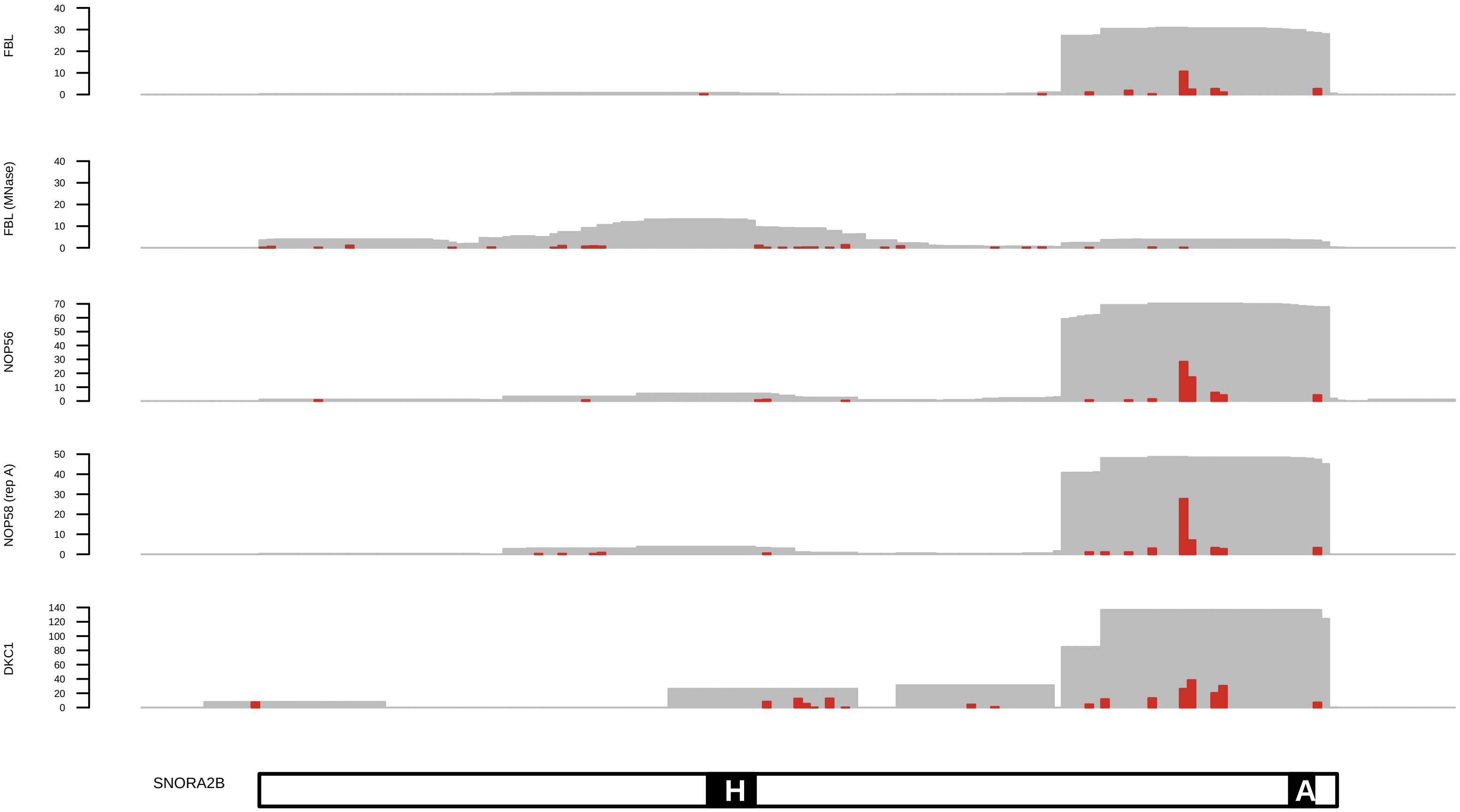

FBL
FBL (MNase)
NOP56
NOP58 (rep A)
DKC1
SNORA2B
H
A

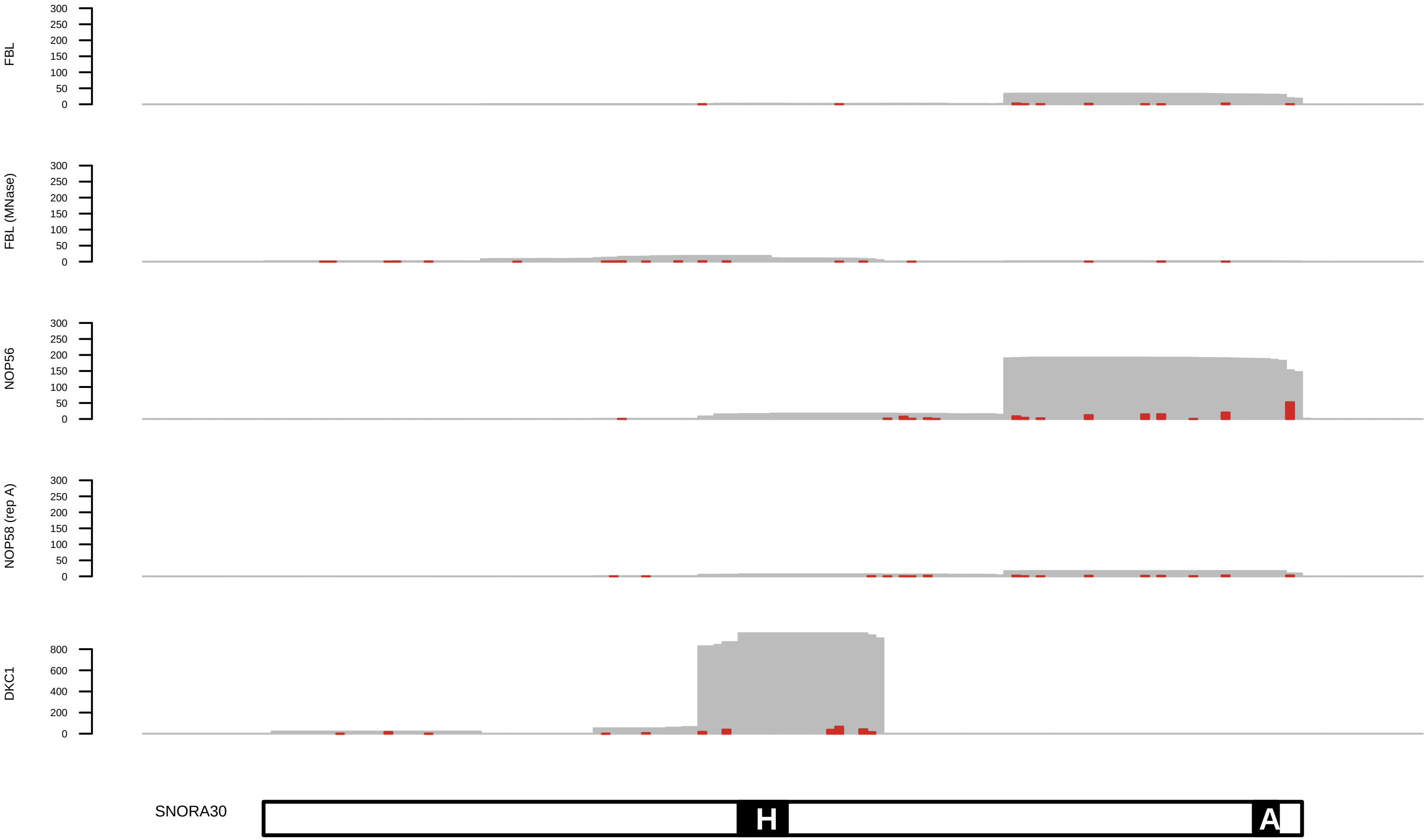
FBL
FBL (MNase)
NOP56
NOP58 (rep A)
DKC1
0
50
100
150
200
250
300
200
400
600
800
SNORA30
H
A

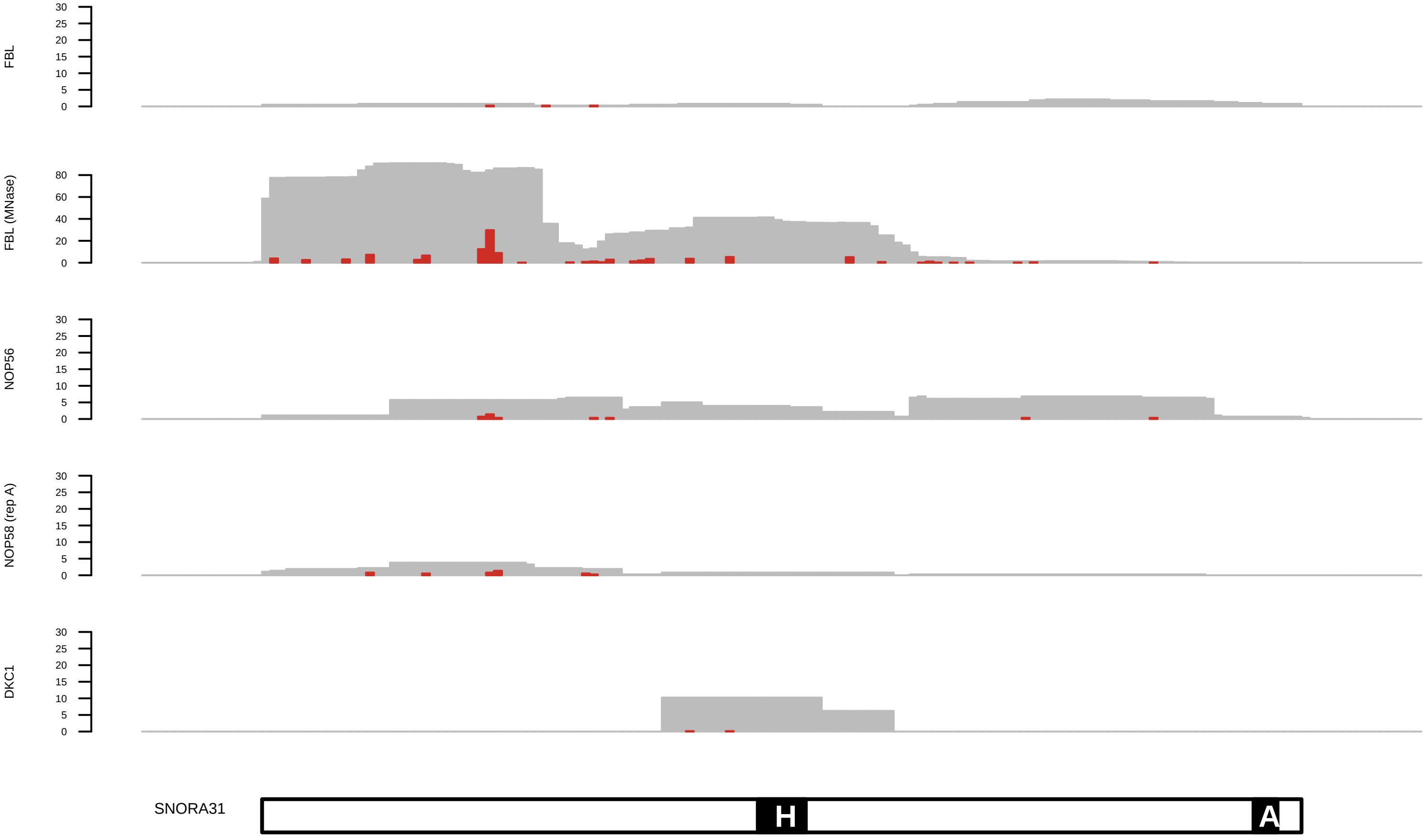
FBL
FBL (MNase)
NOP56
NOP58 (rep A)
DKC1
SNORA31
H
A

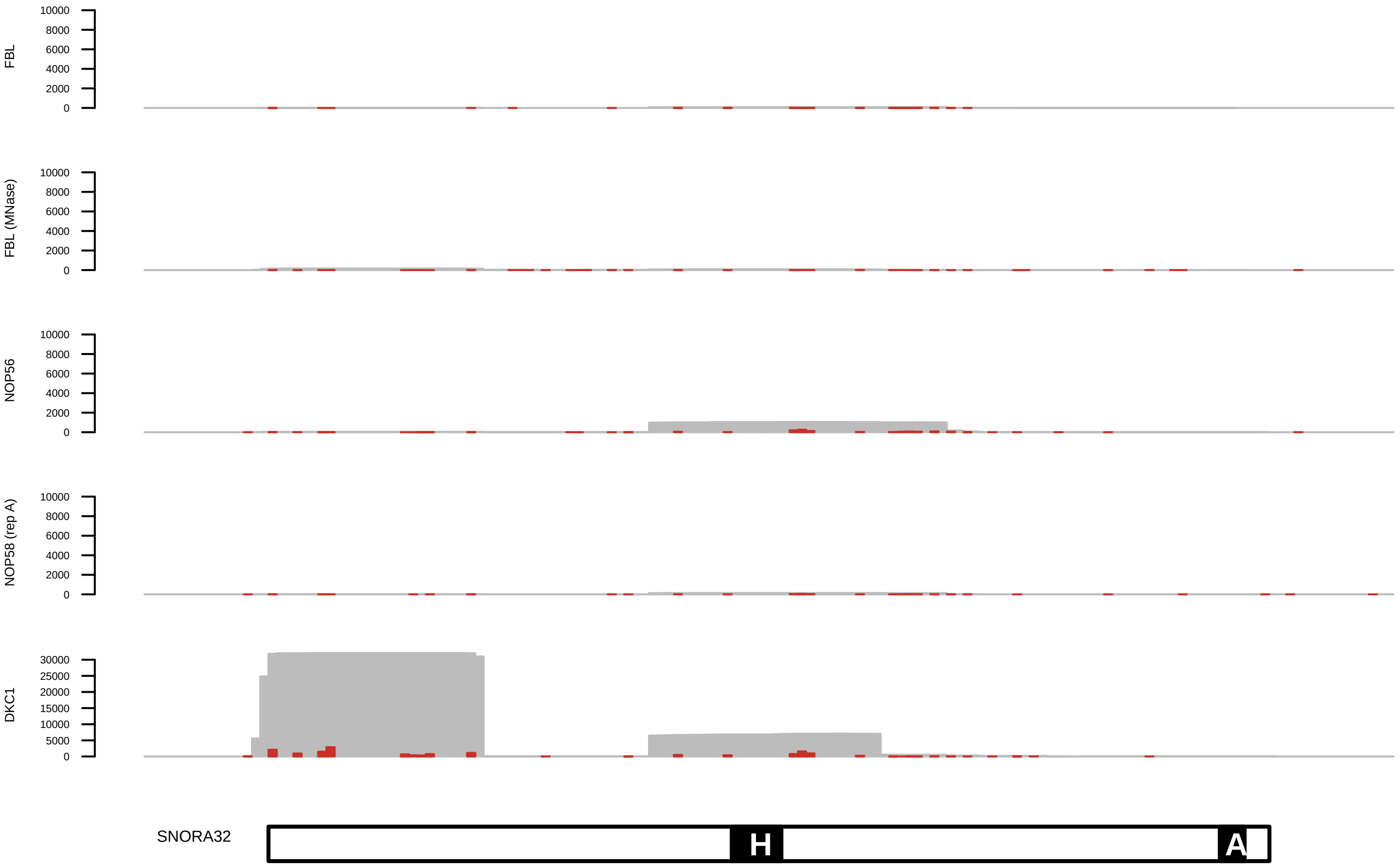
FBL
FBL (MNase)
NOP56
NOP58 (rep A)
DKC1
10000
8000
6000
4000
2000
0
30000
25000
20000
15000
10000
5000
SNORA32
H
A

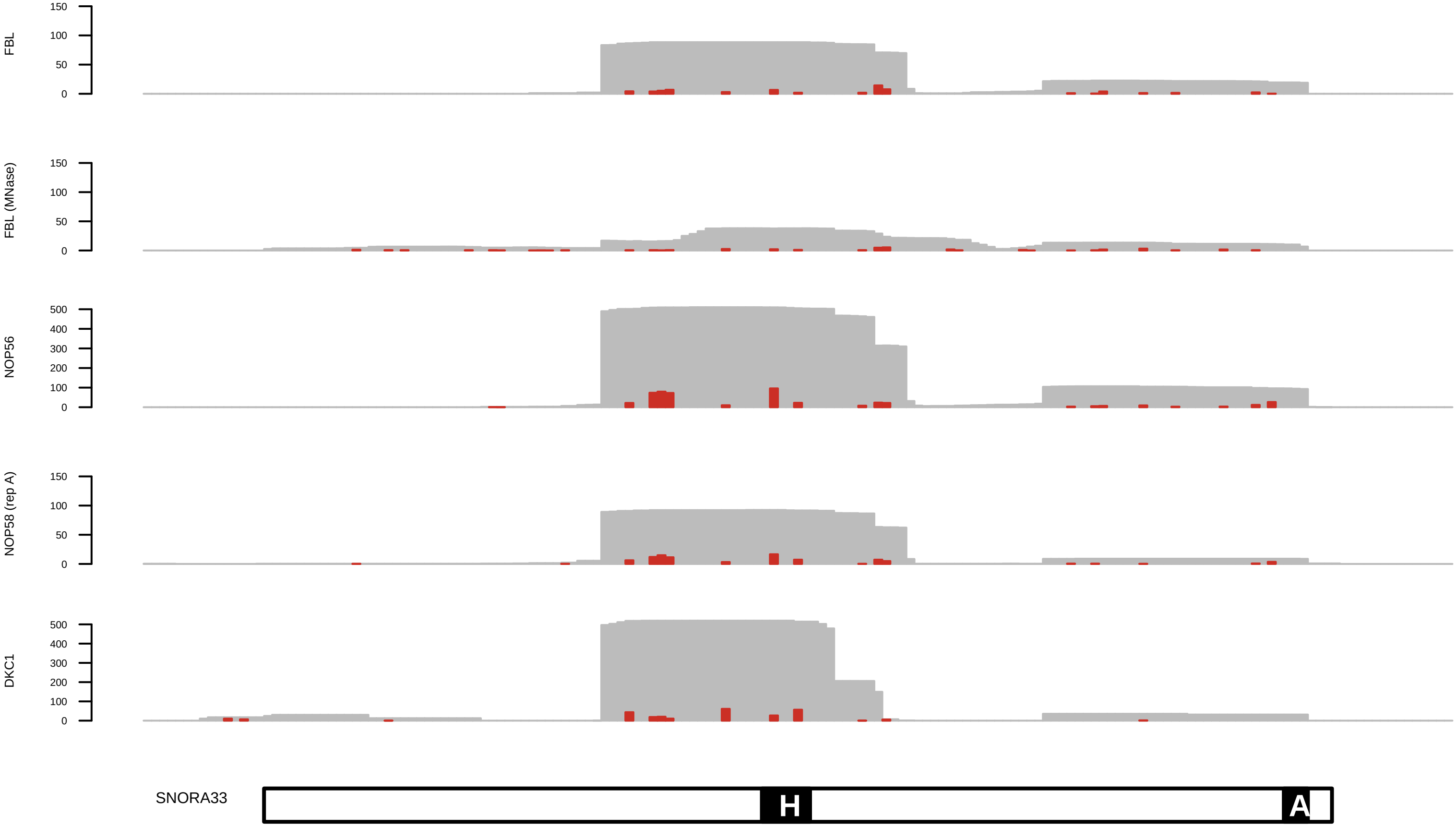
FBL
FBL (MNase)
NOP56
NOP58 (rep A)
DKC1
SNORA33
H
A

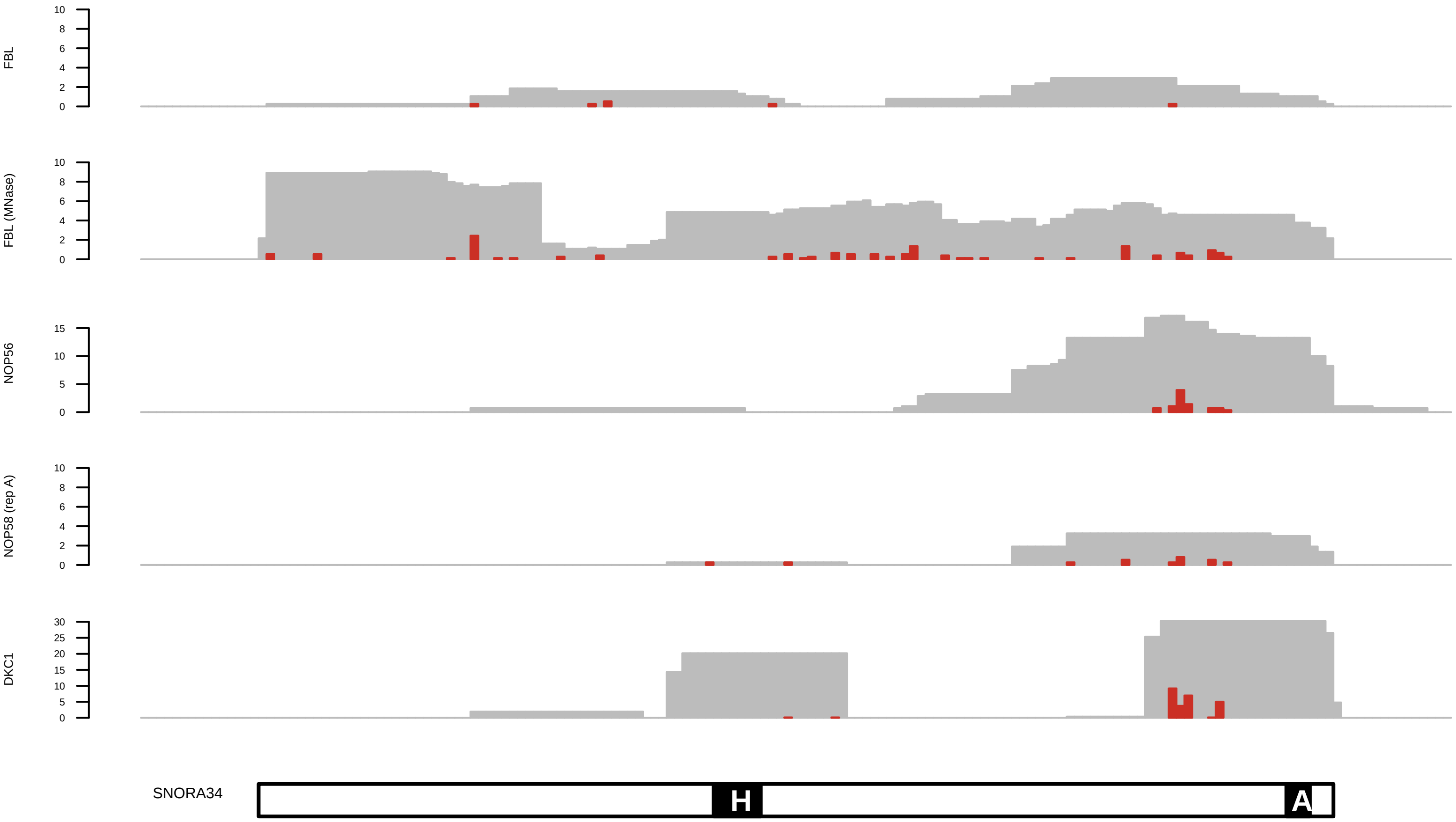

FBL
FBL (MNase)
NOP56
NOP58 (rep A)
DKC1
SNORA34
H
A

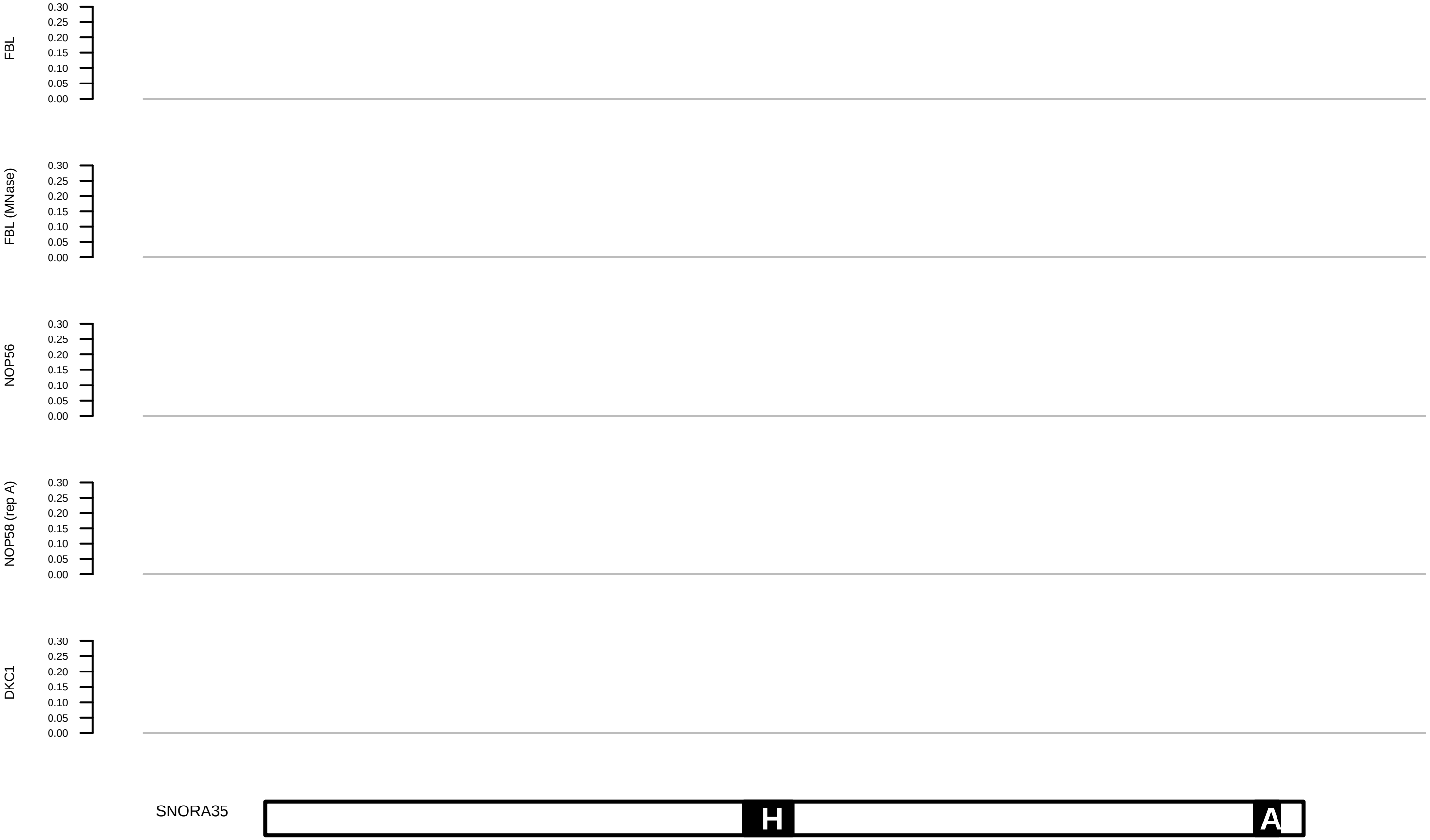
FBL
0.30
0.25
0.20
0.15
0.10
0.05
0.00
FBL (MNase)
NOP56
NOP58 (rep A)
DKC1
SNORA35
H
A

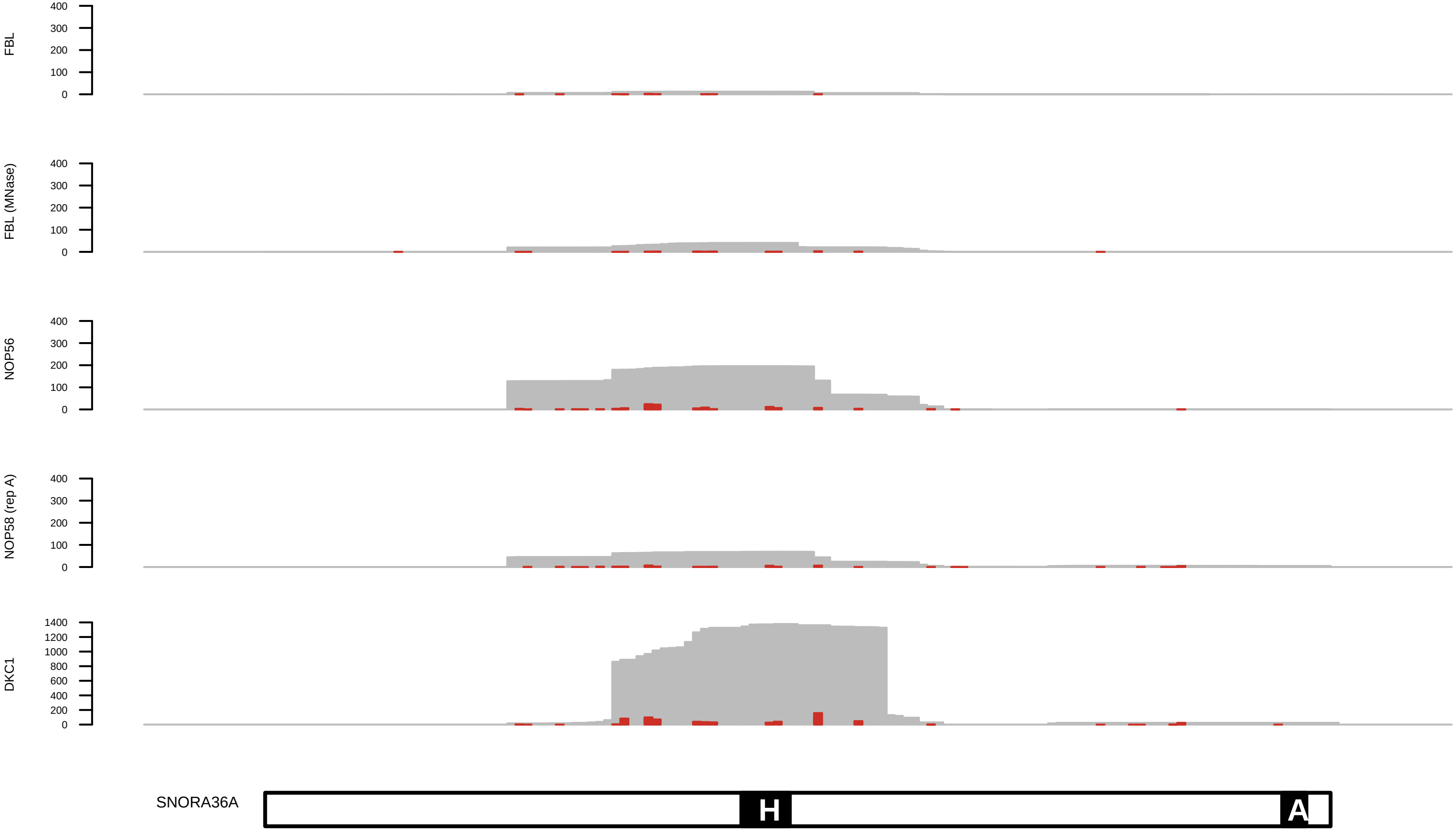
FBL
400
300
200
100
0
FBL (MNase)
400
300
200
100
0
NOP56
400
300
200
100
0
NOP58 (rep A)
400
300
200
100
0
DKC1
1400
1200
1000
800
600
400
200
0
SNORA36A
H
A

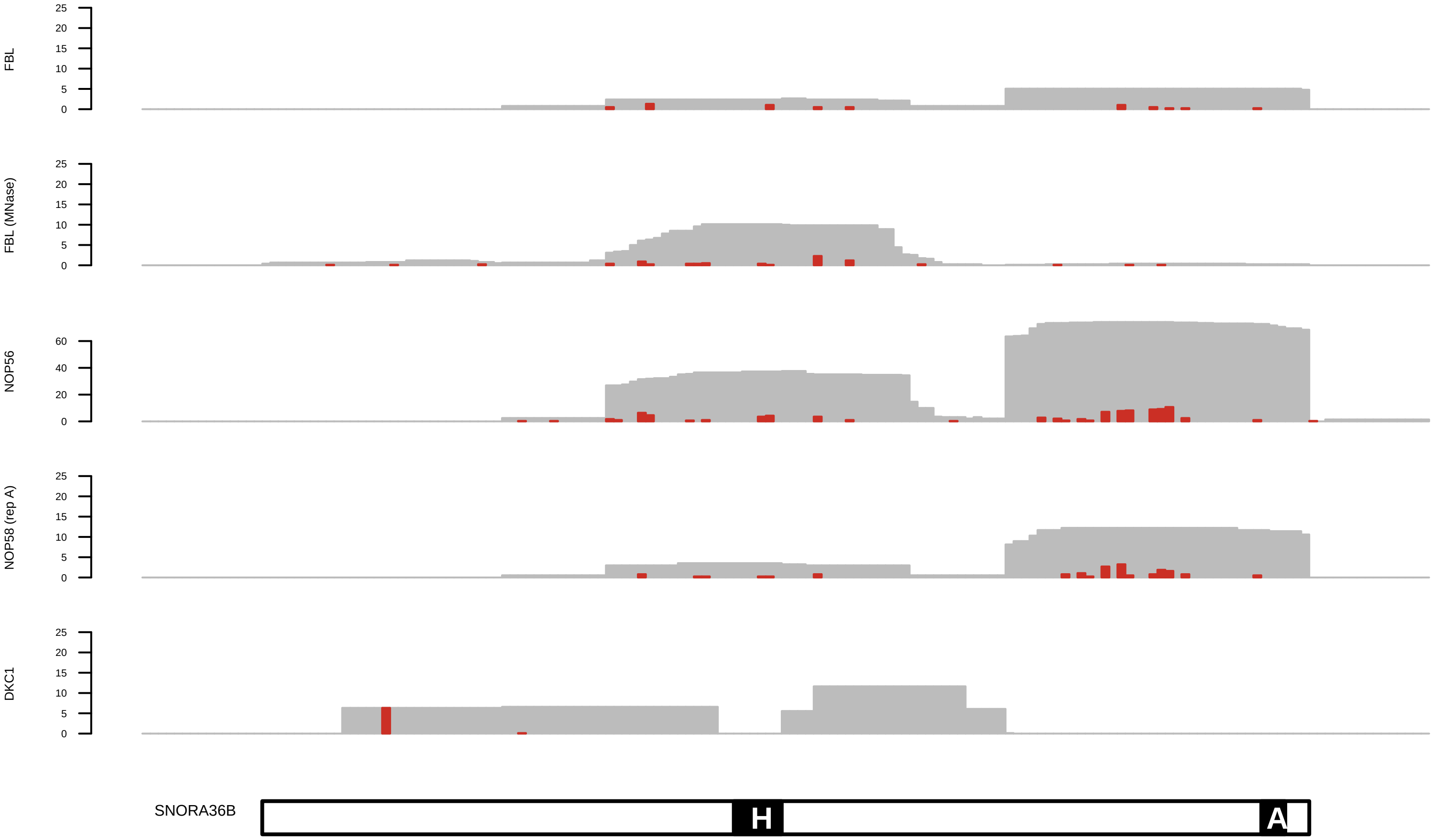

FBL
FBL (MNase)
NOP56
NOP58 (rep A)
DKC1
0
5
10
15
20
25
40
60
SNORA36B
H
A

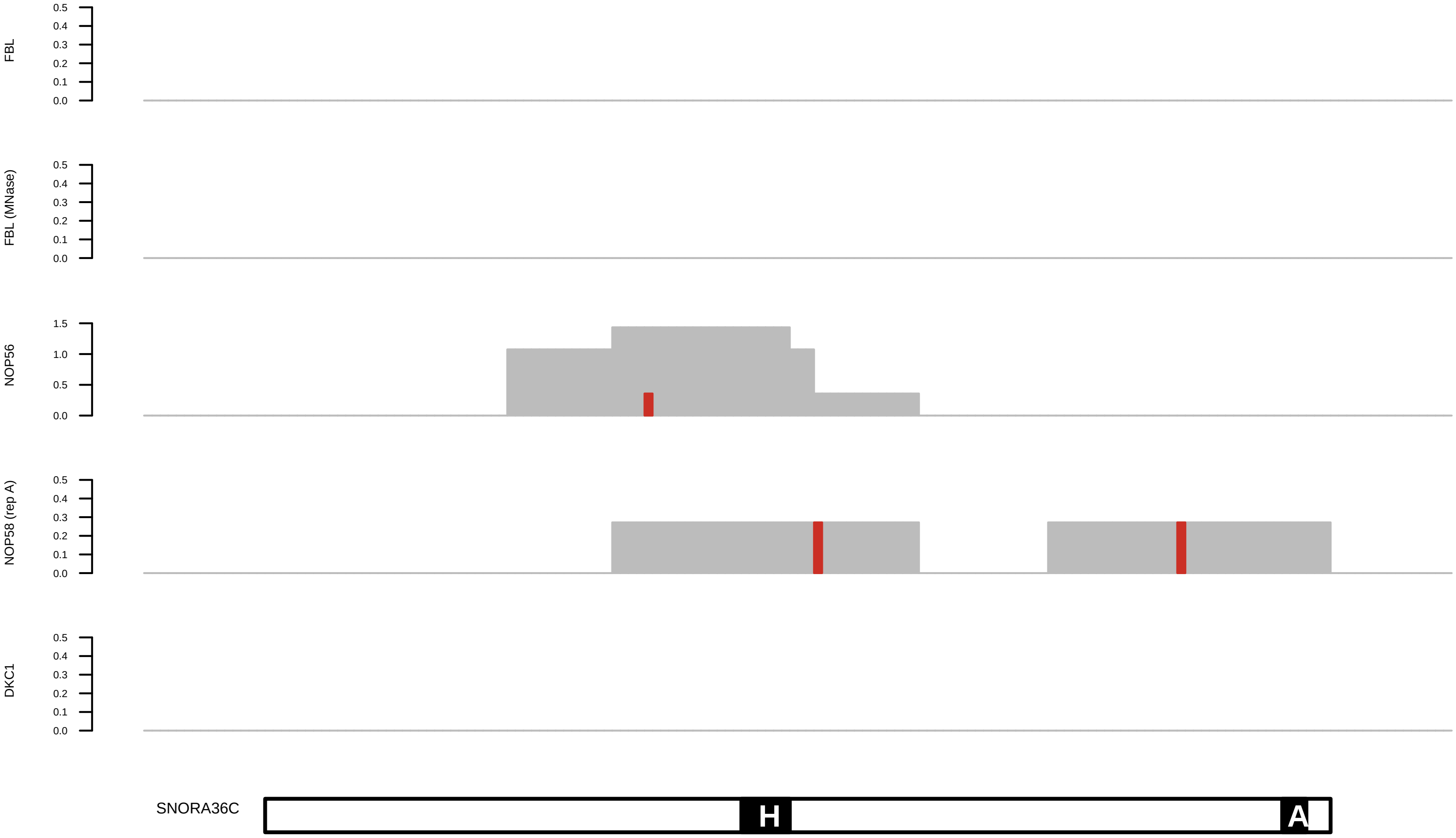
FBL
FBL (MNase)
NOP56
NOP58 (rep A)
DKC1
0.0
0.1
0.2
0.3
0.4
0.5
1.0
1.5
SNORA36C
H
A

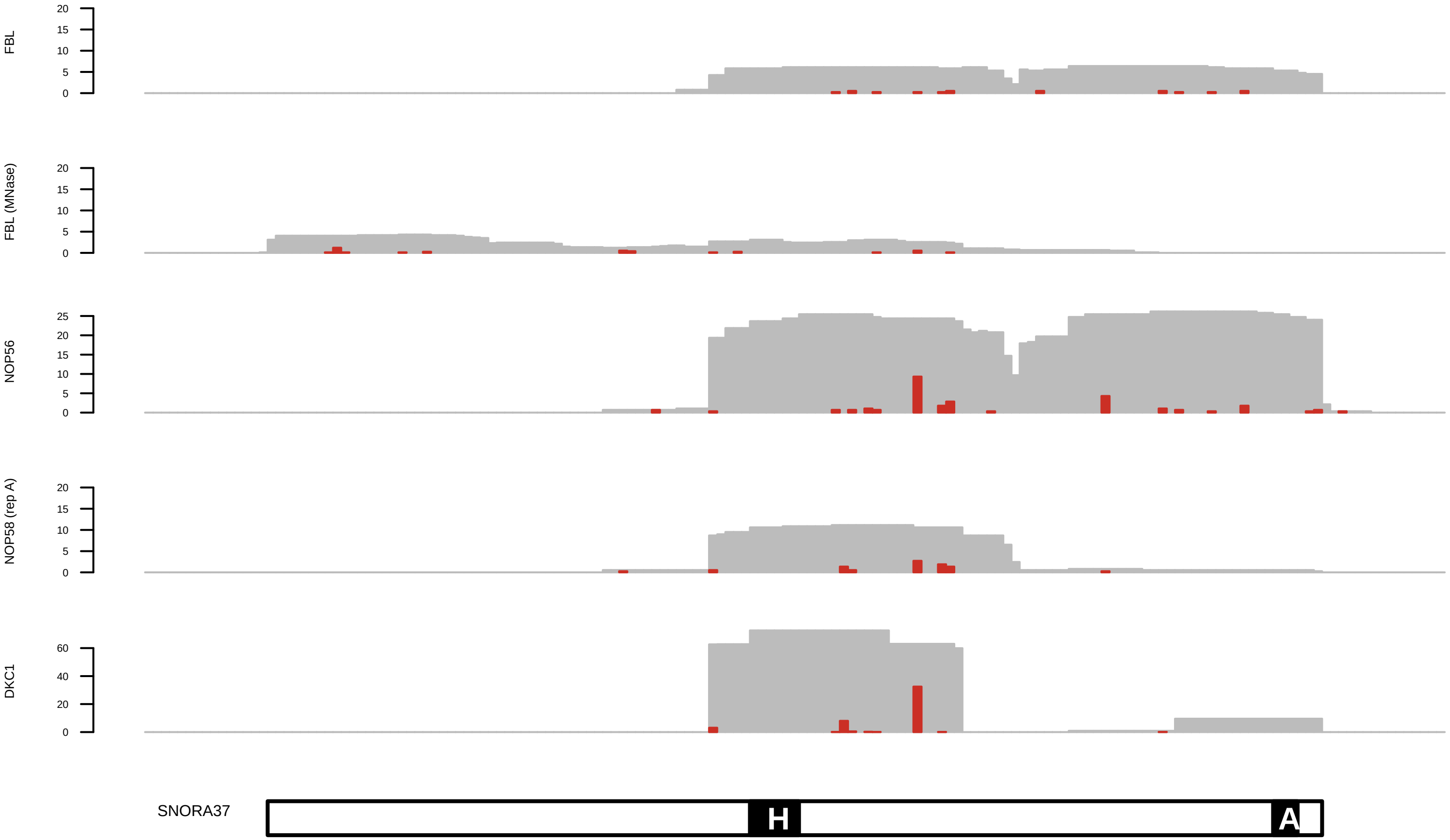
FBL
FBL (MNase)
NOP56
NOP58 (rep A)
DKC1
SNORA37
H
A

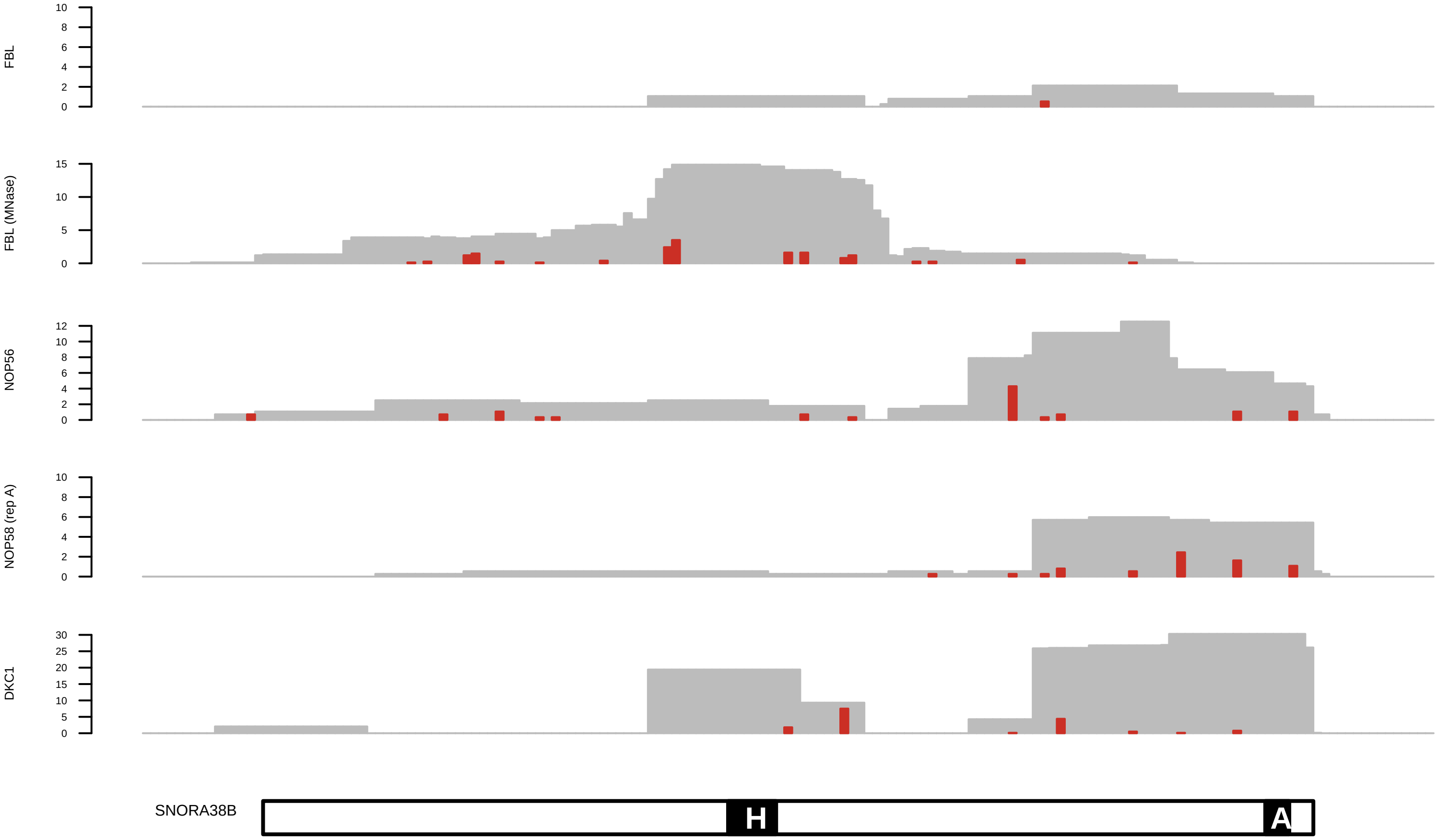
FBL
FBL (MNase)
NOP56
NOP58 (rep A)
DKC1
SNORA38B
H
A

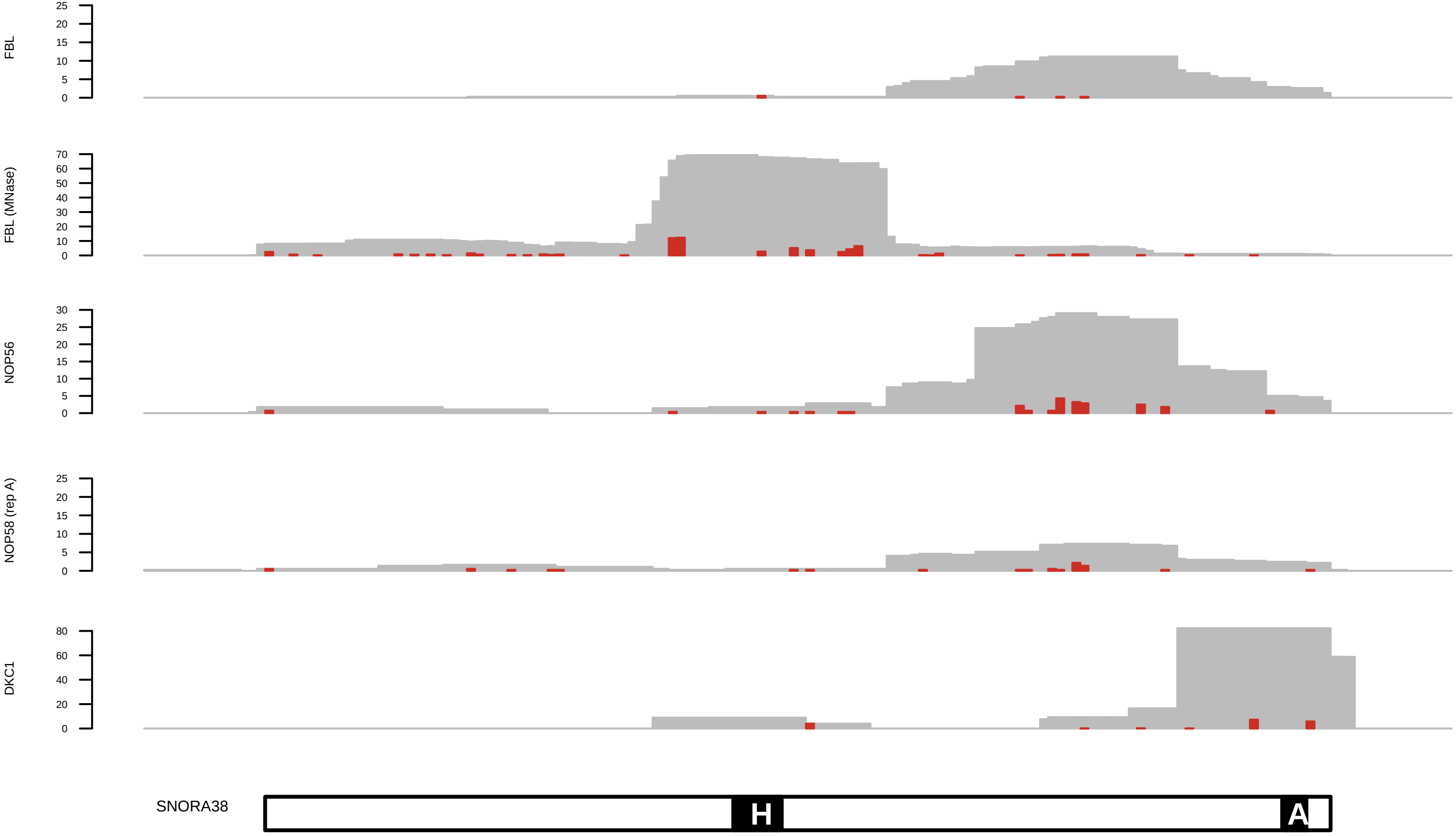
FBL
FBL (MNase)
NOP56
NOP58 (rep A)
DKC1
SNORA38
H
A

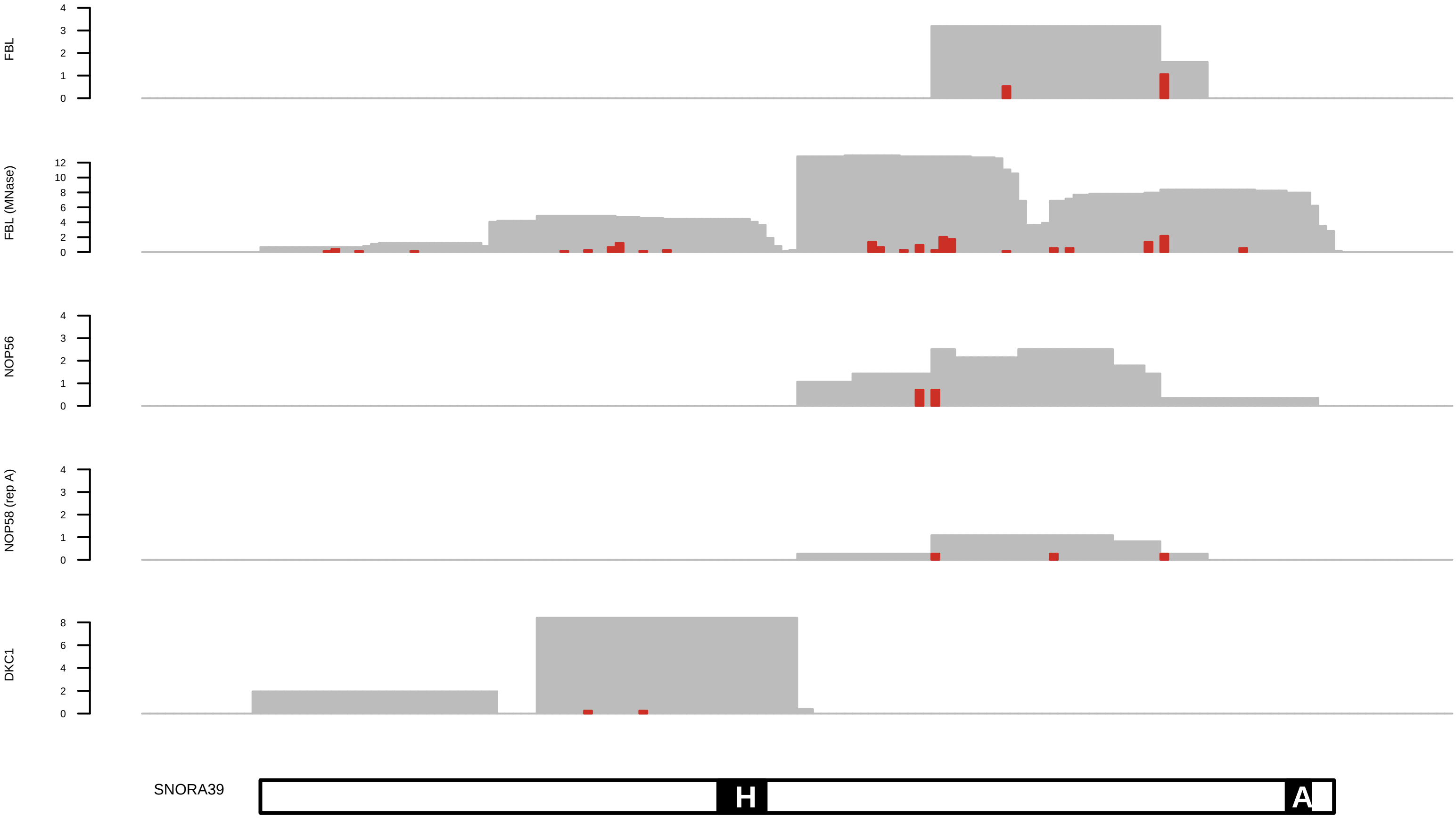
FBL
FBL (MNase)
NOP56
NOP58 (rep A)
DKC1
SNORA39
H
A

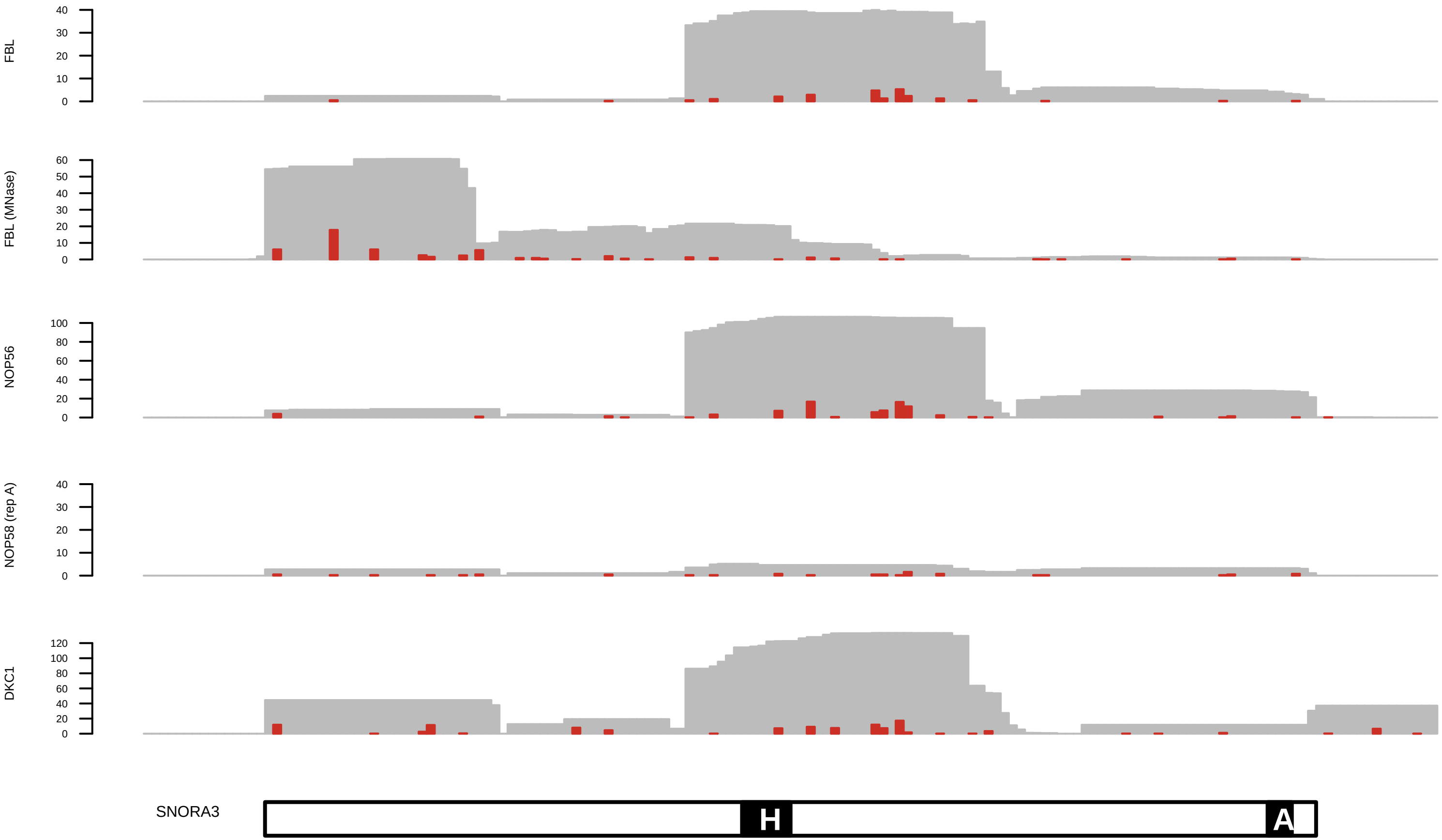

FBL
40
30
20
10
0
FBL (MNase)
60
50
40
30
20
10
0
NOP56
100
80
60
40
20
0
NOP58 (rep A)
40
30
20
10
0
DKC1
120
100
80
60
40
20
0
SNORA3
H
A

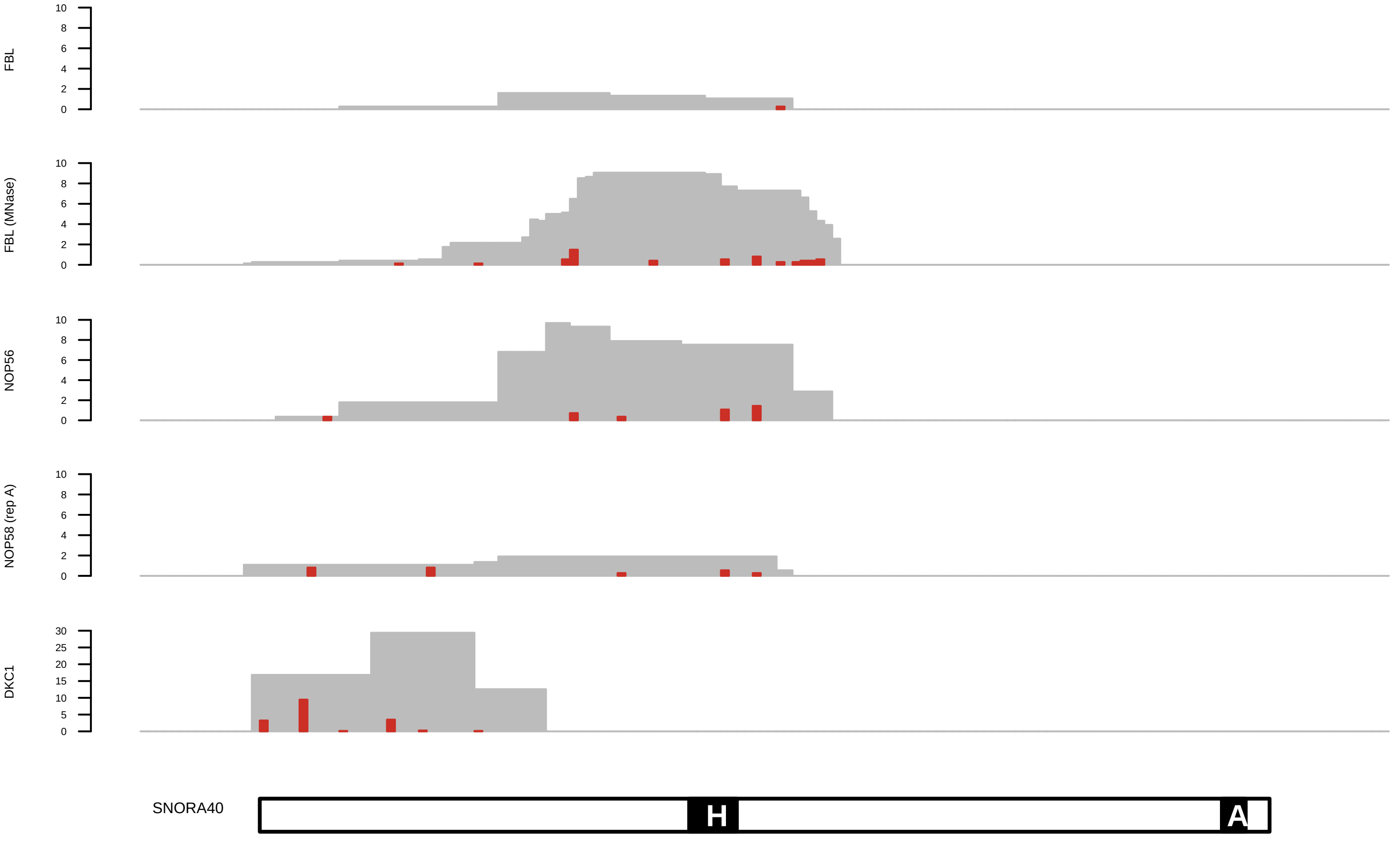

FBL
FBL (MNase)
NOP56
NOP58 (rep A)
DKC1
0
2
4
6
8
10
0
5
10
15
20
25
30
SNORA40
H
A

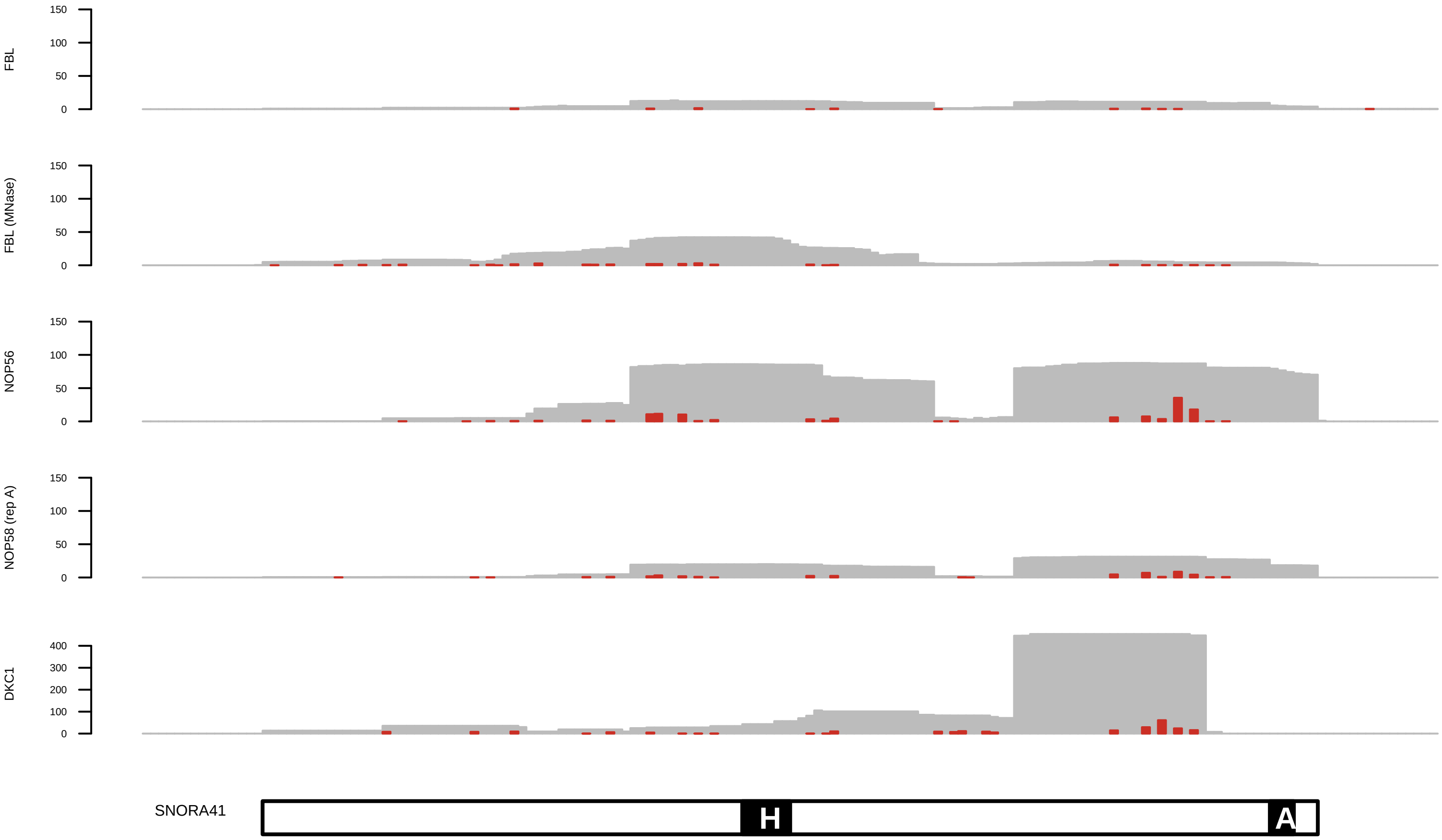

FBL
150
100
50
0
FBL (MNase)
150
100
50
0
NOP56
150
100
50
0
NOP58 (rep A)
150
100
50
0
DKC1
400
300
200
100
0
SNORA41
H
A

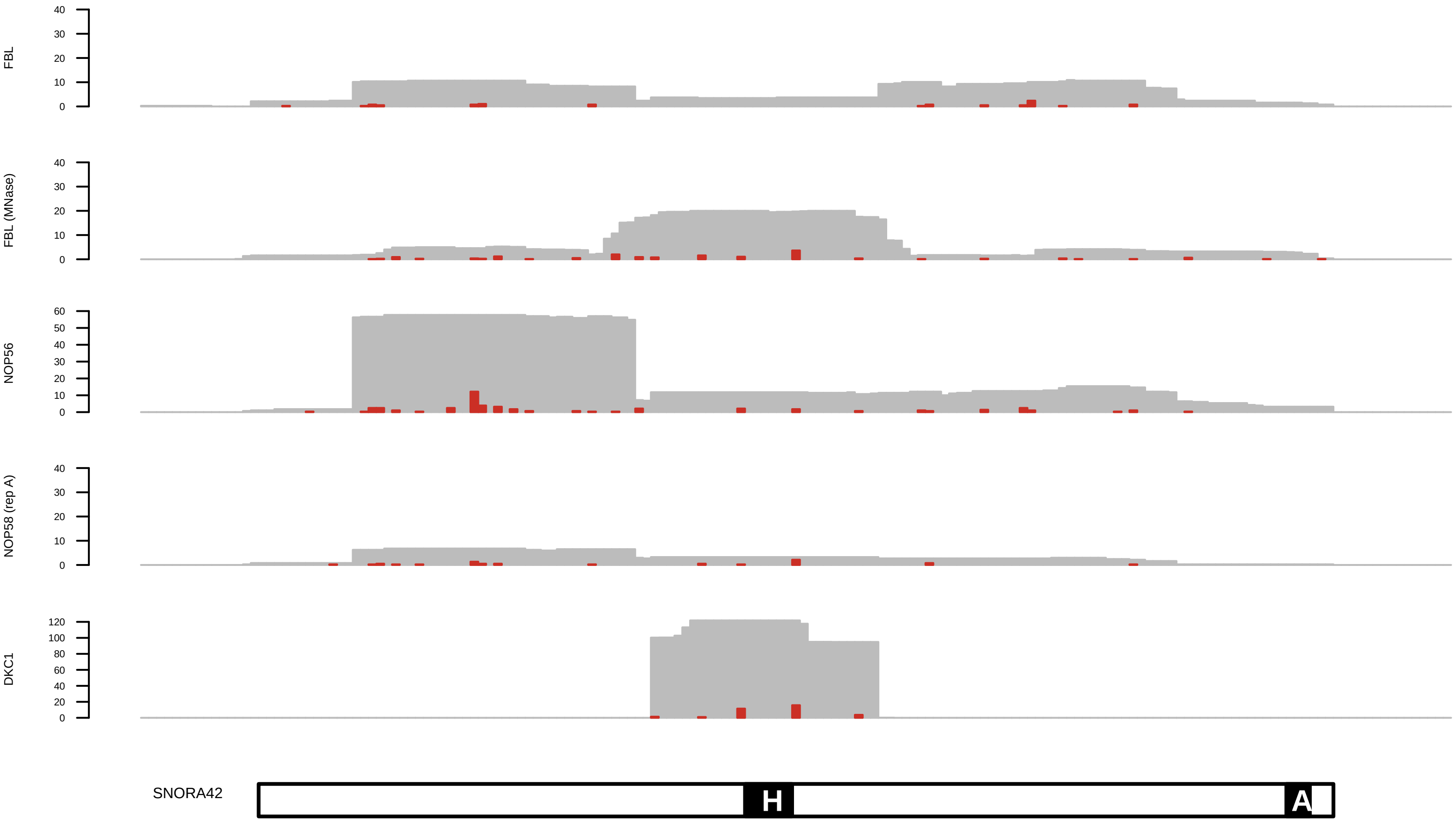
FBL
FBL (MNase)
NOP56
NOP58 (rep A)
DKC1
SNORA42
H
A

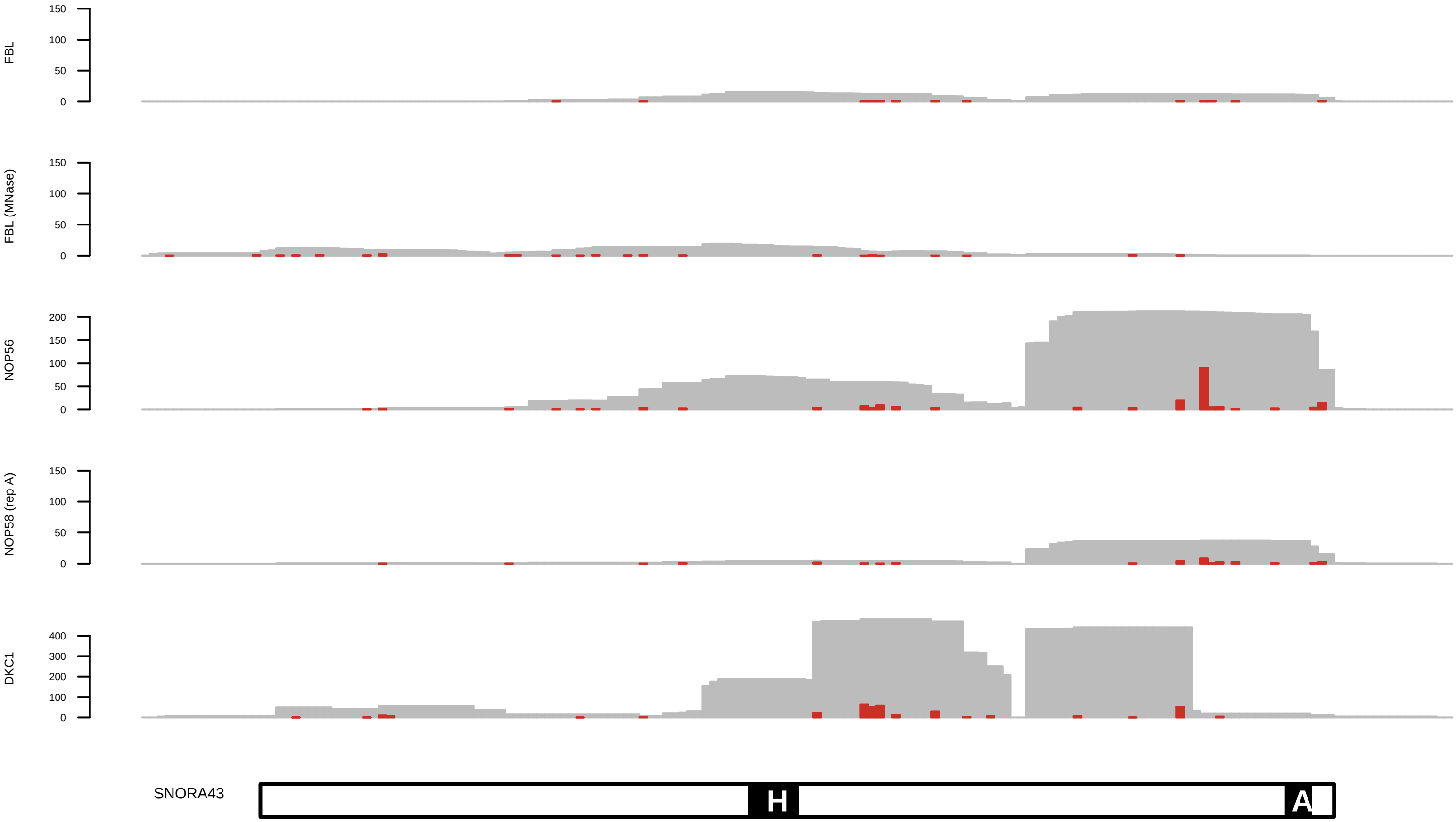

FBL
FBL (MNase)
NOP56
NOP58 (rep A)
DKC1
SNORA43
H
A

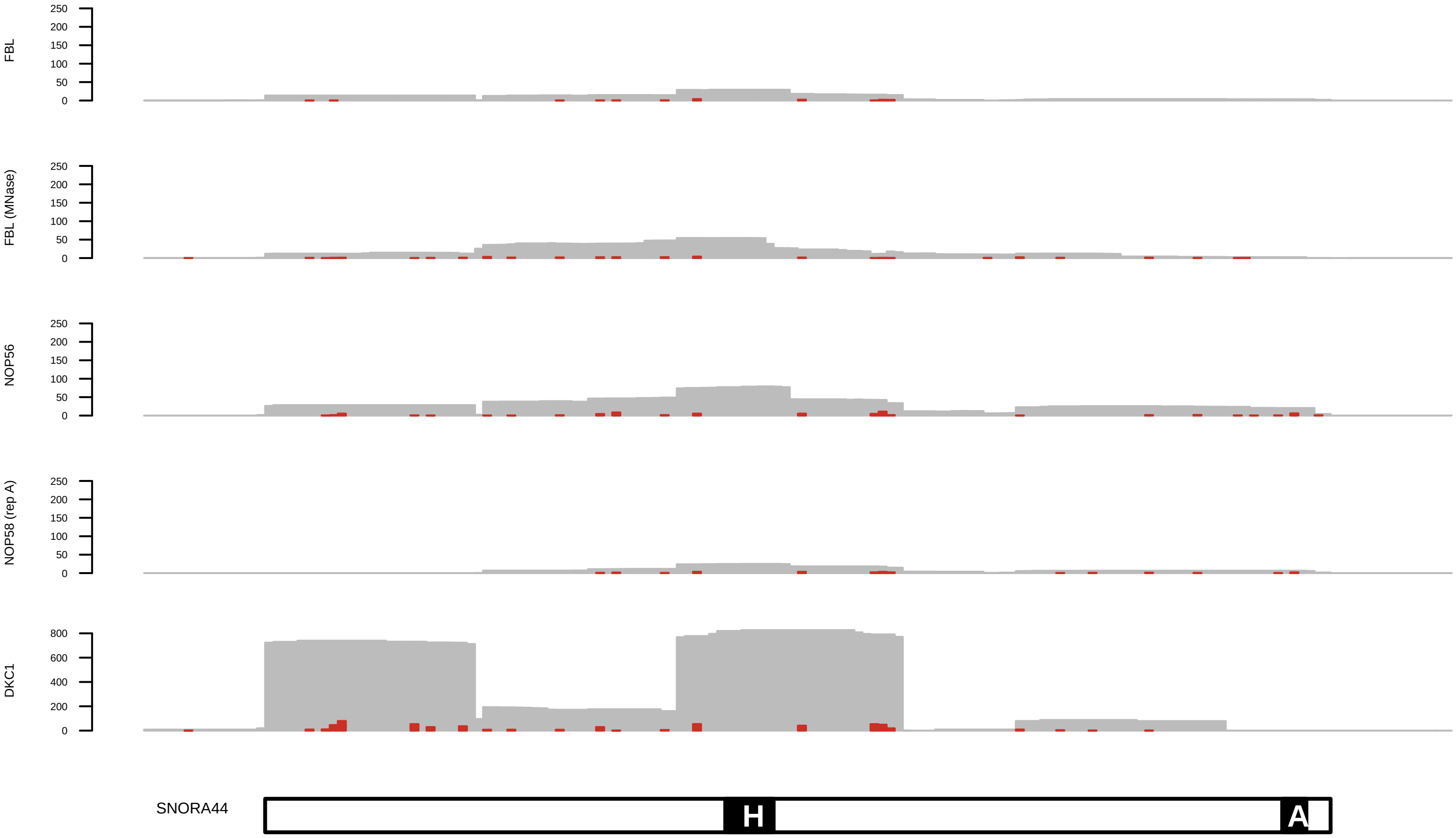

FBL
250
200
150
100
50
0
FBL (MNase)
250
200
150
100
50
0
NOP56
250
200
150
100
50
0
NOP58 (rep A)
250
200
150
100
50
0
DKC1
800
600
400
200
0
SNORA44
H
A

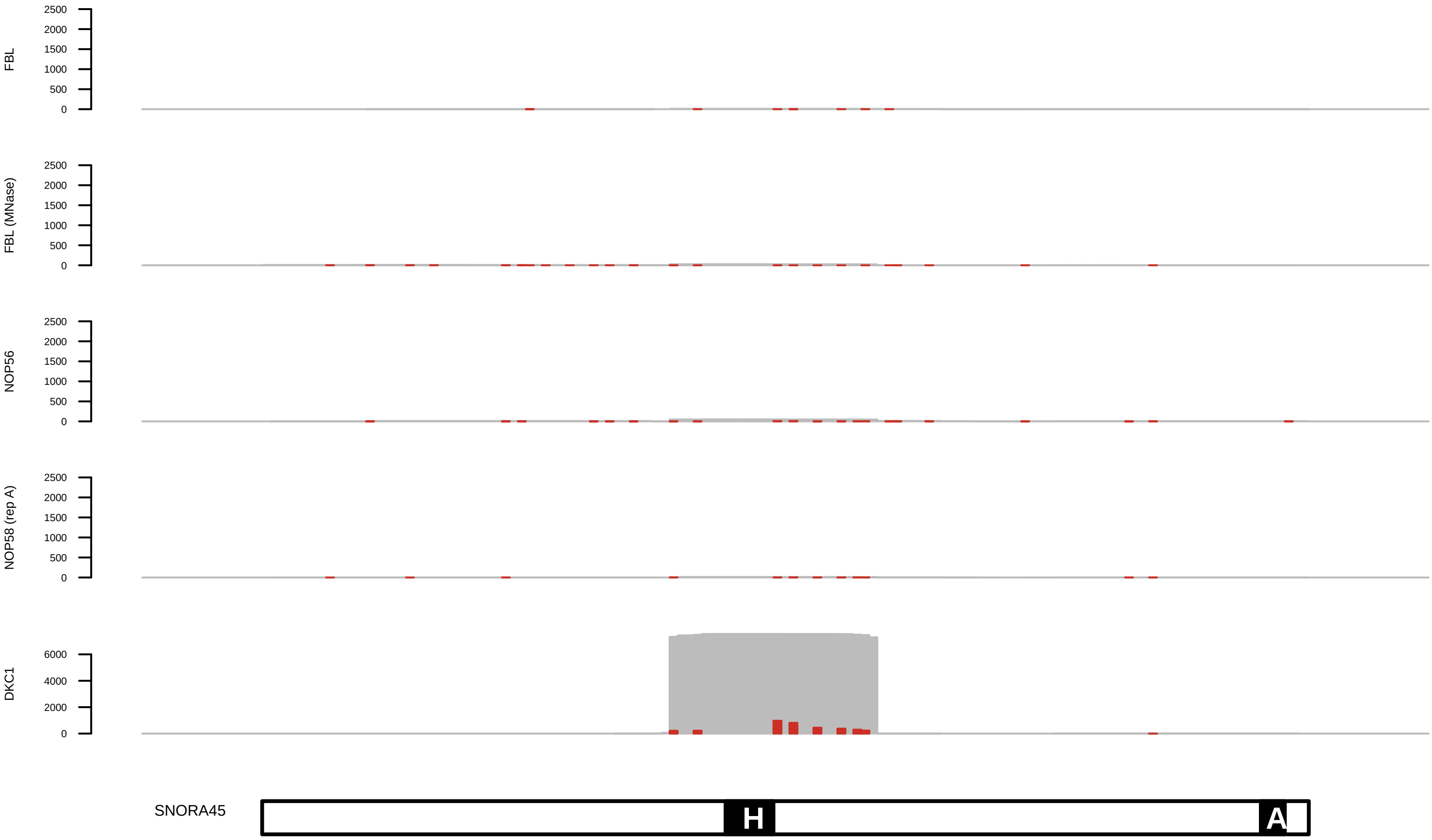

FBL
2500
2000
1500
1000
500
0
FBL (MNase)
2500
2000
1500
1000
500
0
NOP56
2500
2000
1500
1000
500
0
NOP58 (rep A)
2500
2000
1500
1000
500
0
DKC1
6000
4000
2000
0
SNORA45
H
A

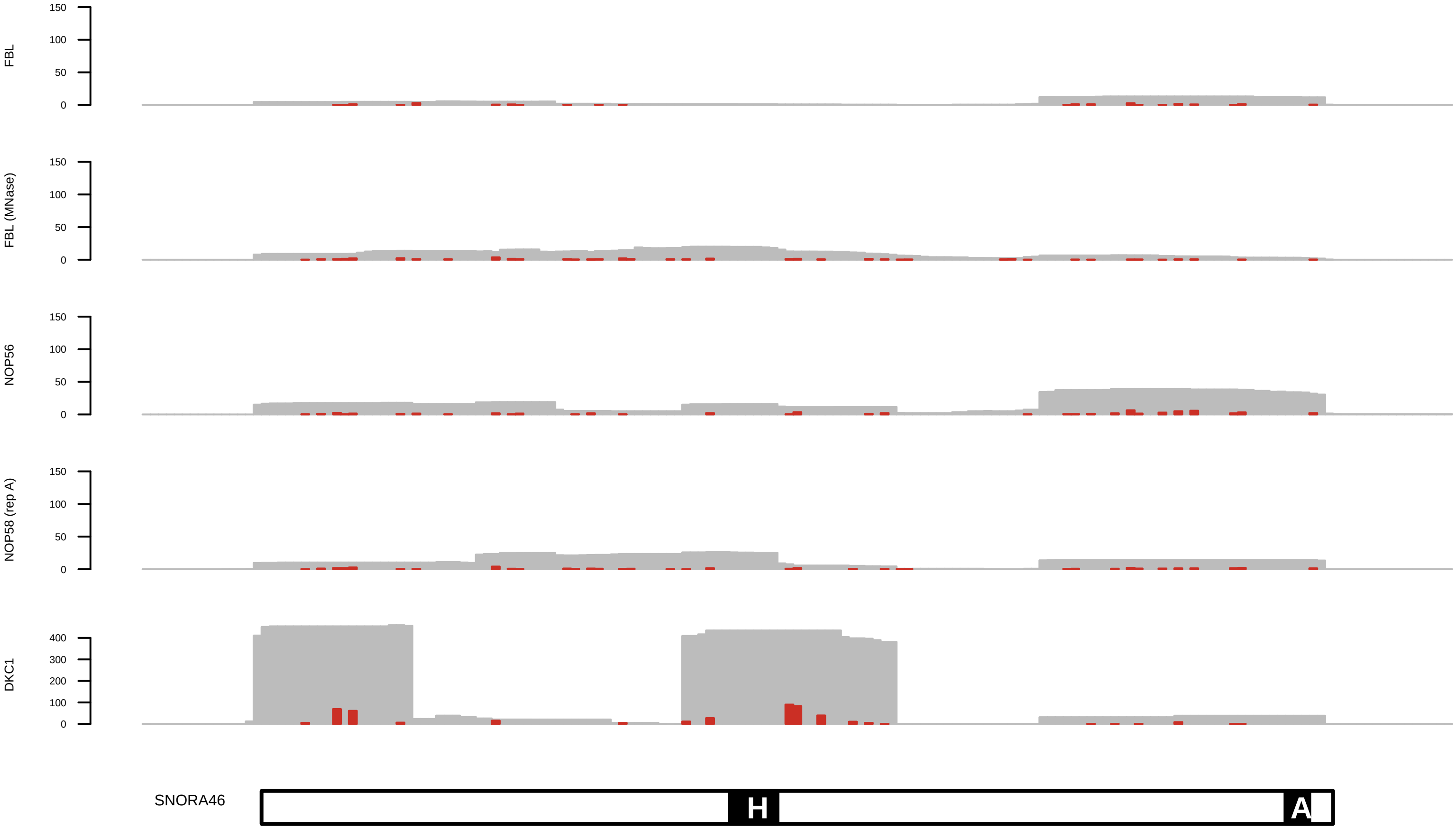
FBL
FBL (MNase)
NOP56
NOP58 (rep A)
DKC1
150
100
50
0
400
300
200
SNORA46
H
A

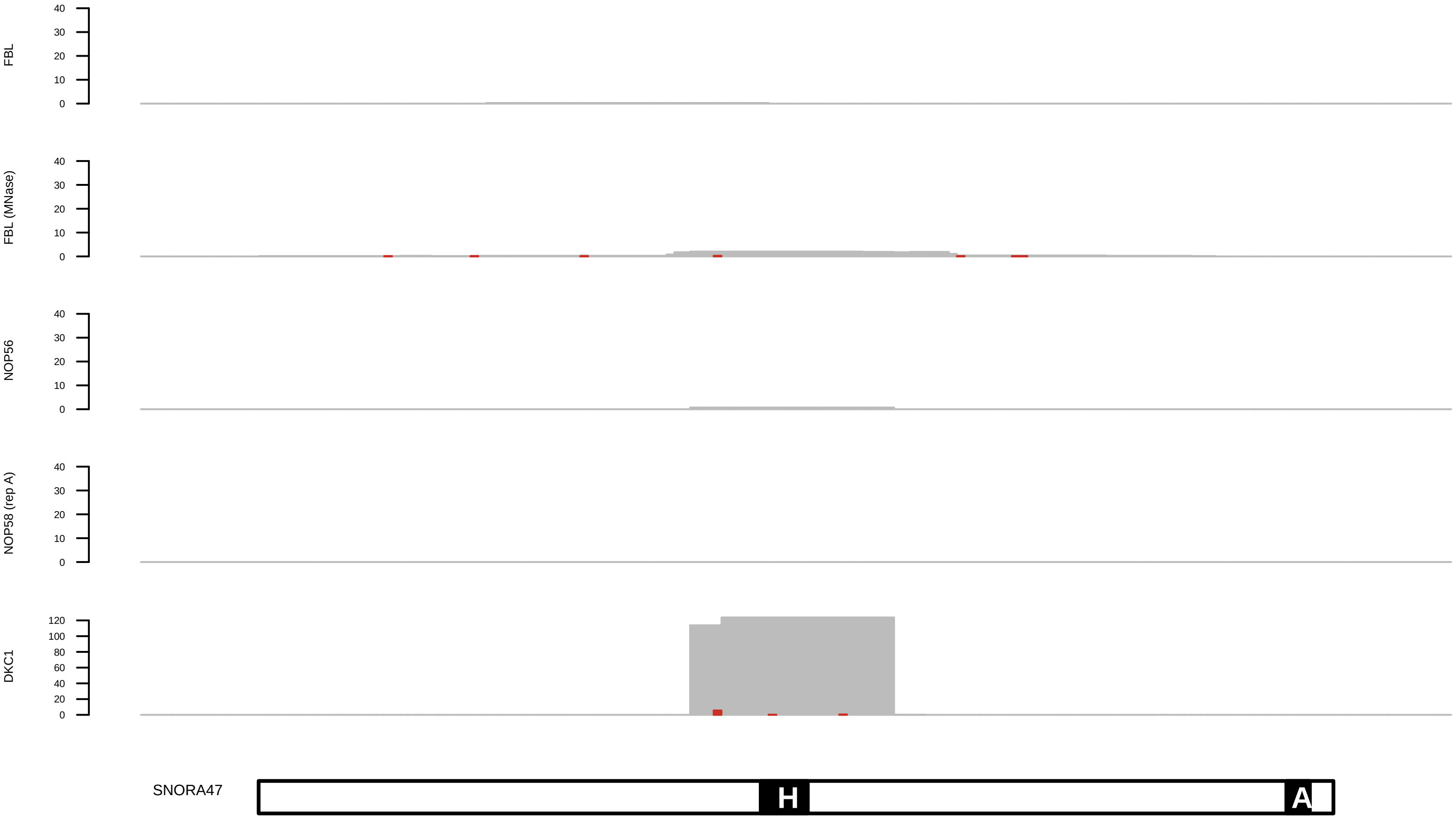
FBL
FBL (MNase)
NOP56
NOP58 (rep A)
DKC1
40
30
20
10
0
120
100
80
60
40
20
SNORA47
H
A

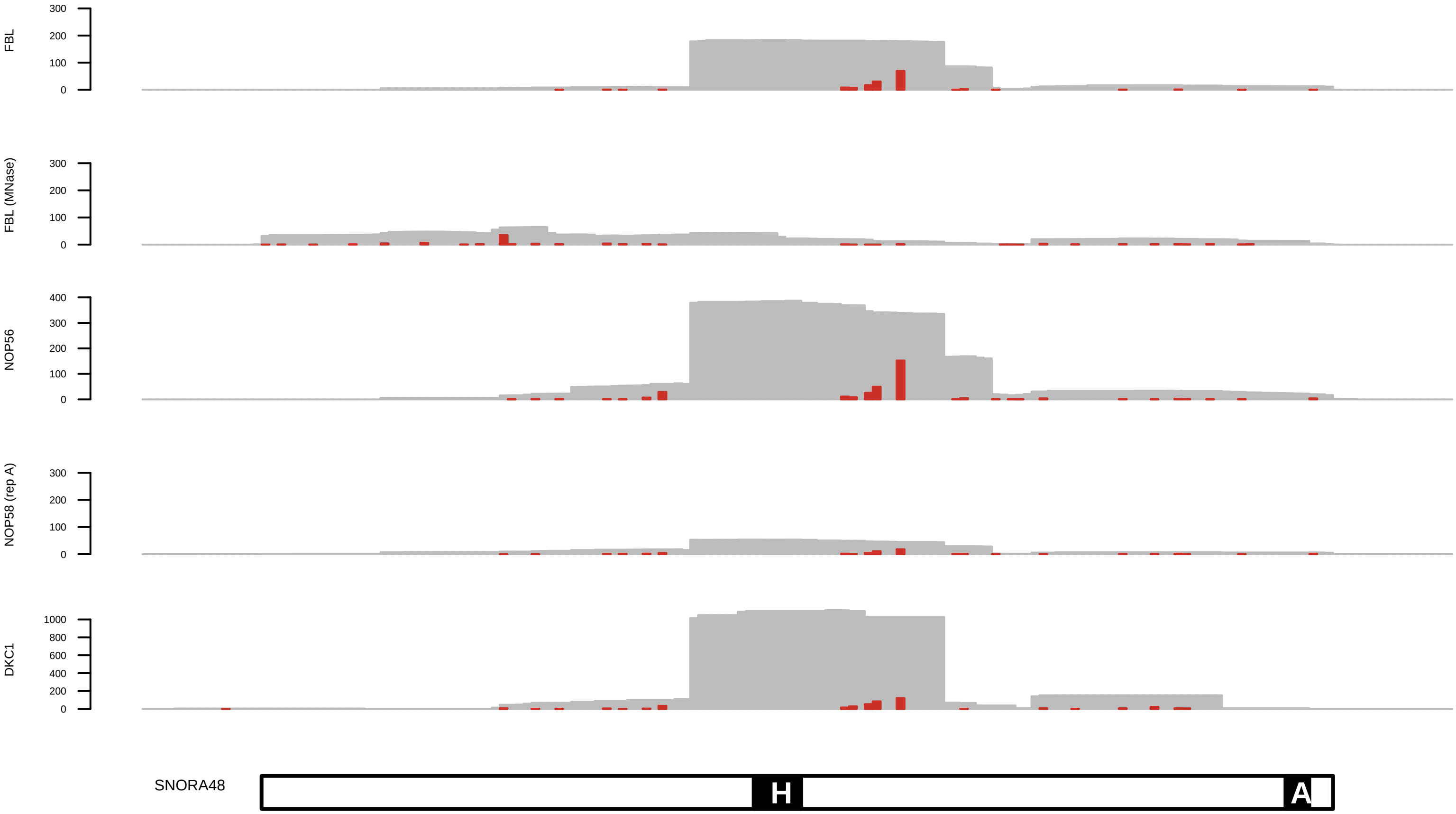
FBL
0
100
200
300
FBL (MNase)
0
100
200
300
NOP56
0
100
200
300
400
NOP58 (rep A)
0
100
200
300
DKC1
0
200
400
600
800
1000
SNORA48
H
A

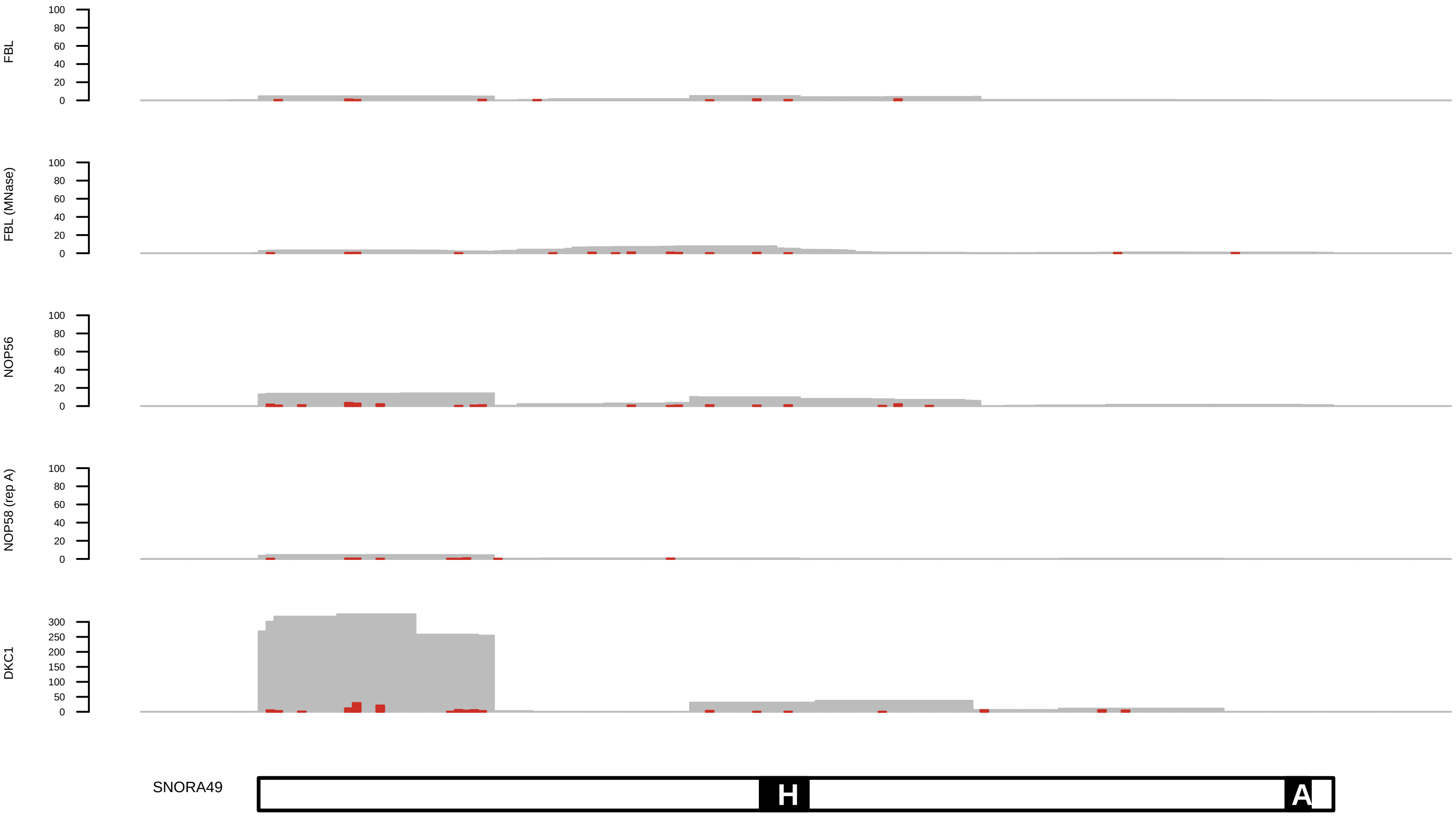
FBL
FBL (MNase)
NOP56
NOP58 (rep A)
DKC1
0
20
40
60
80
100
50
150
200
250
300
SNORA49
H
A

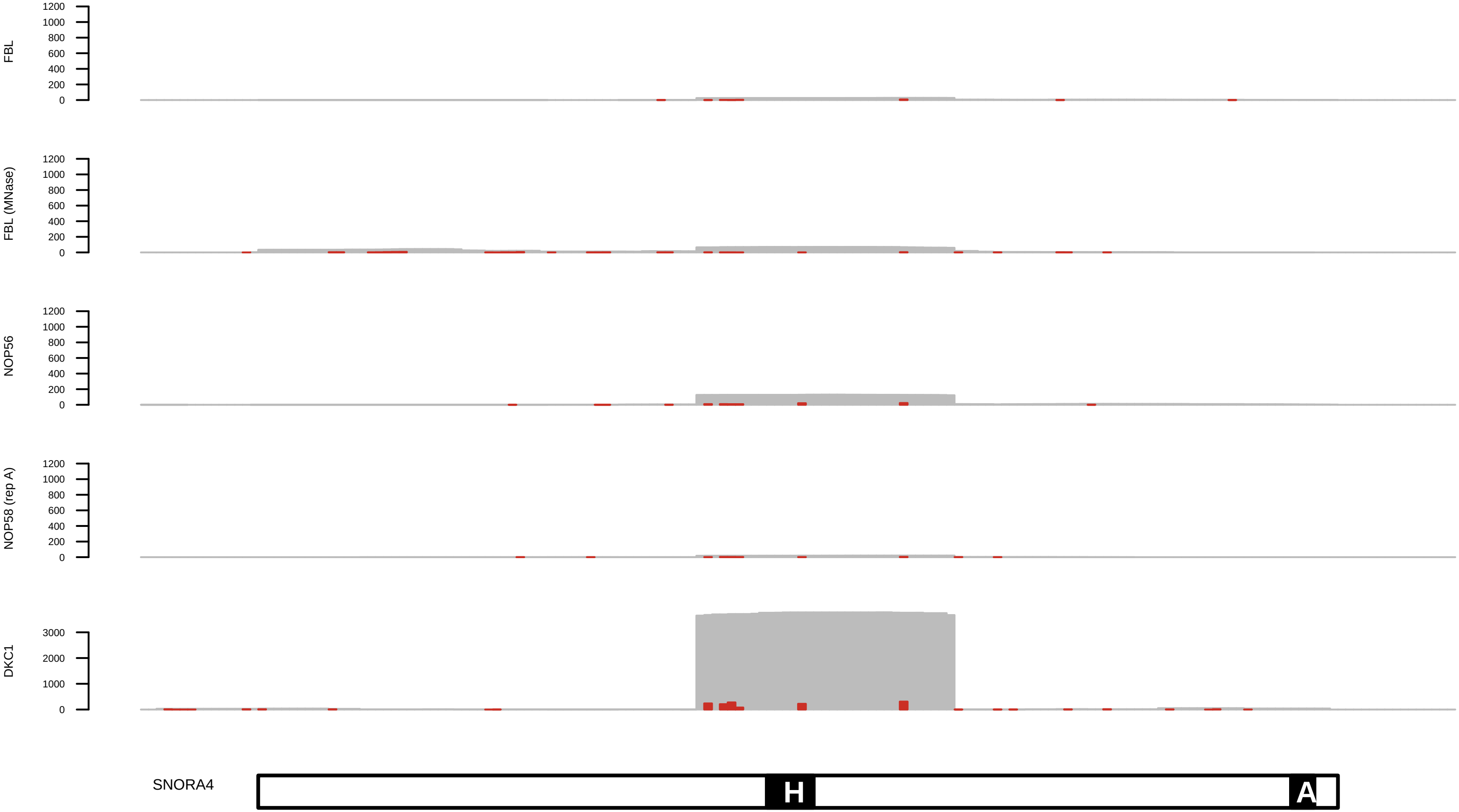

FBL
FBL (MNase)
NOP56
NOP58 (rep A)
DKC1
1200
1000
800
600
400
200
0
3000
2000
1000
SNORA4
H
A

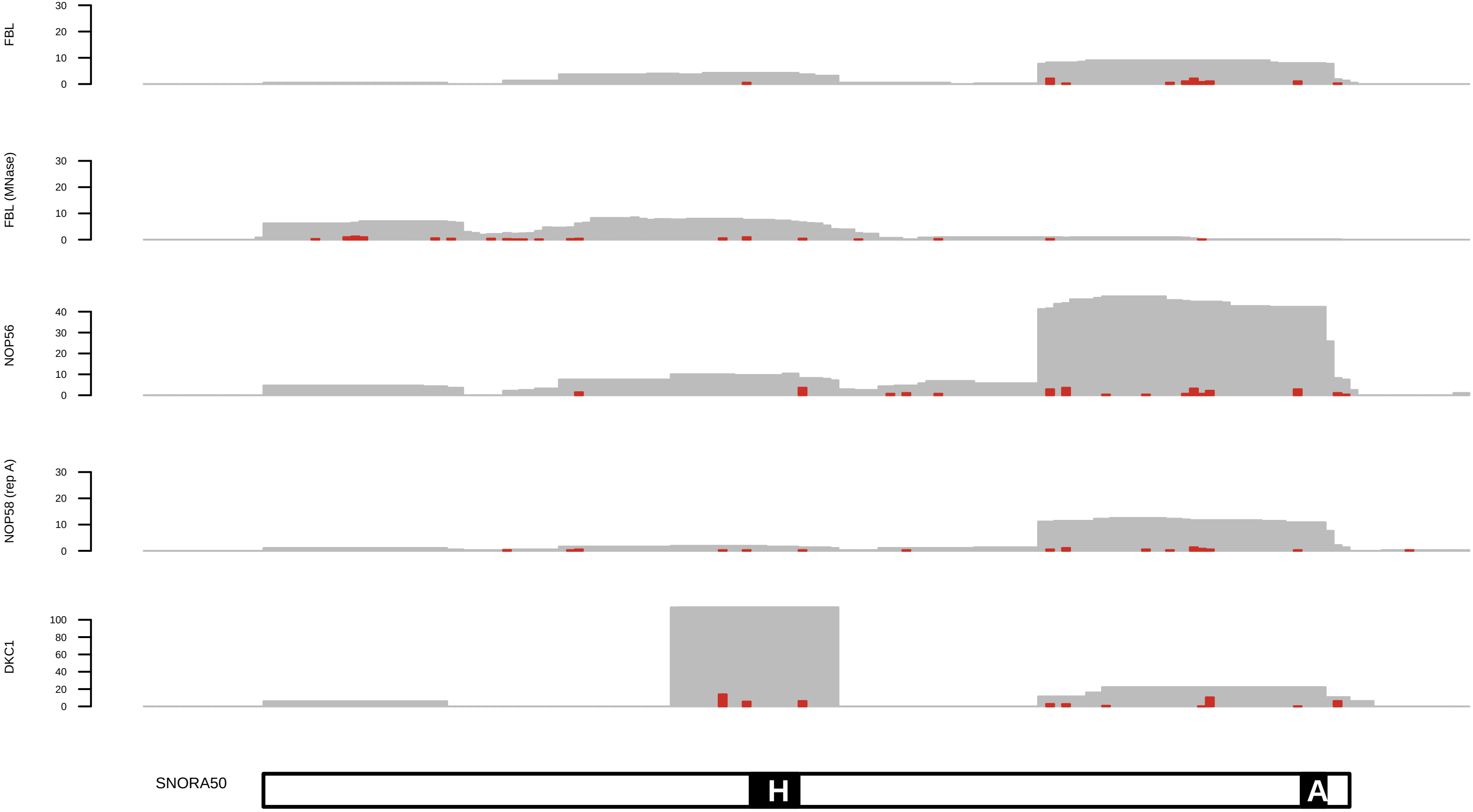
FBL
30
20
10
0
FBL (MNase)
30
20
10
0
NOP56
40
30
20
10
0
NOP58 (rep A)
30
20
10
0
DKC1
100
80
60
40
20
0
SNORA50
H
A

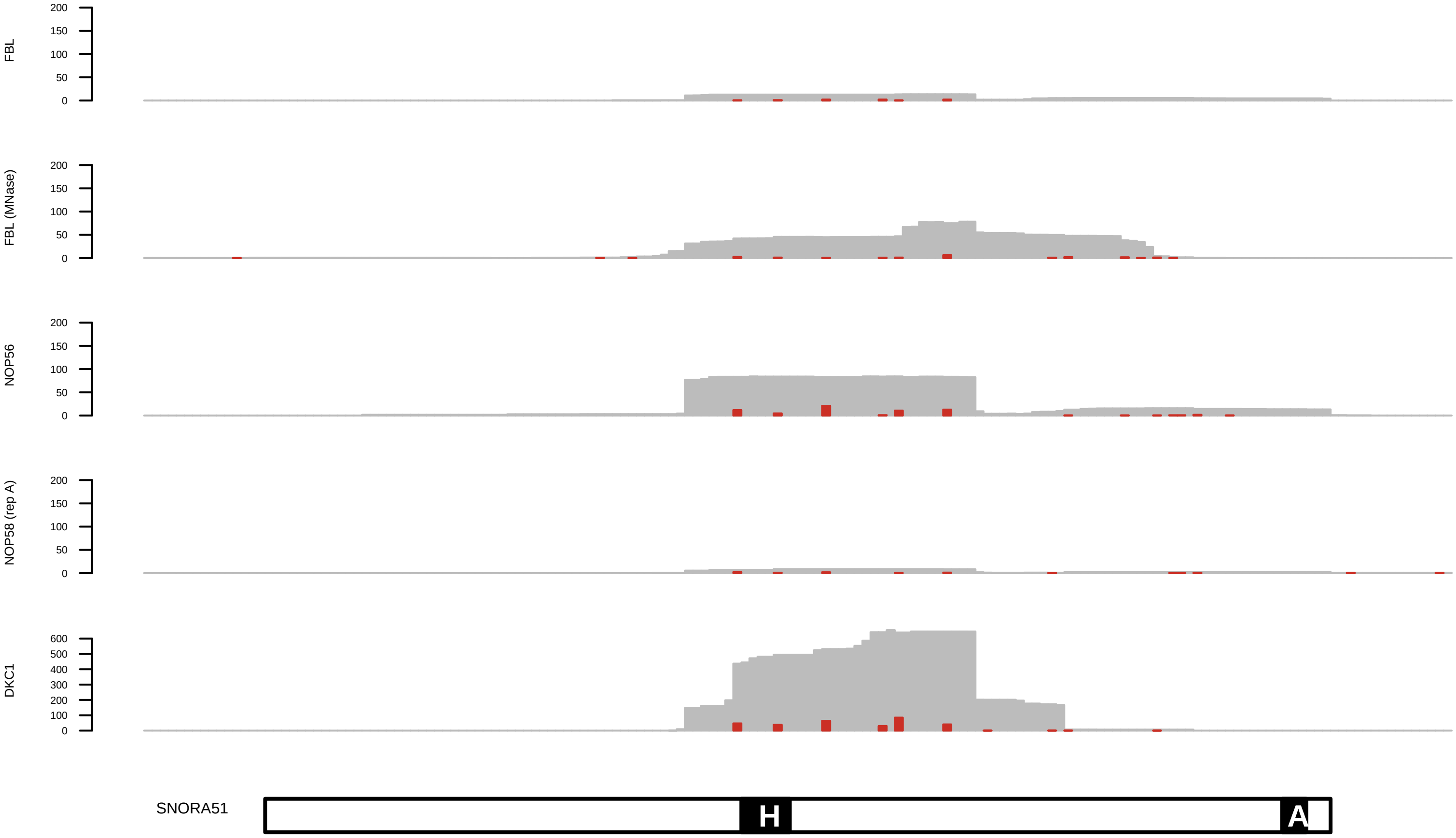

FBL
FBL (MNase)
NOP56
NOP58 (rep A)
DKC1
0
50
100
150
200
300
400
500
600
SNORA51
H
A

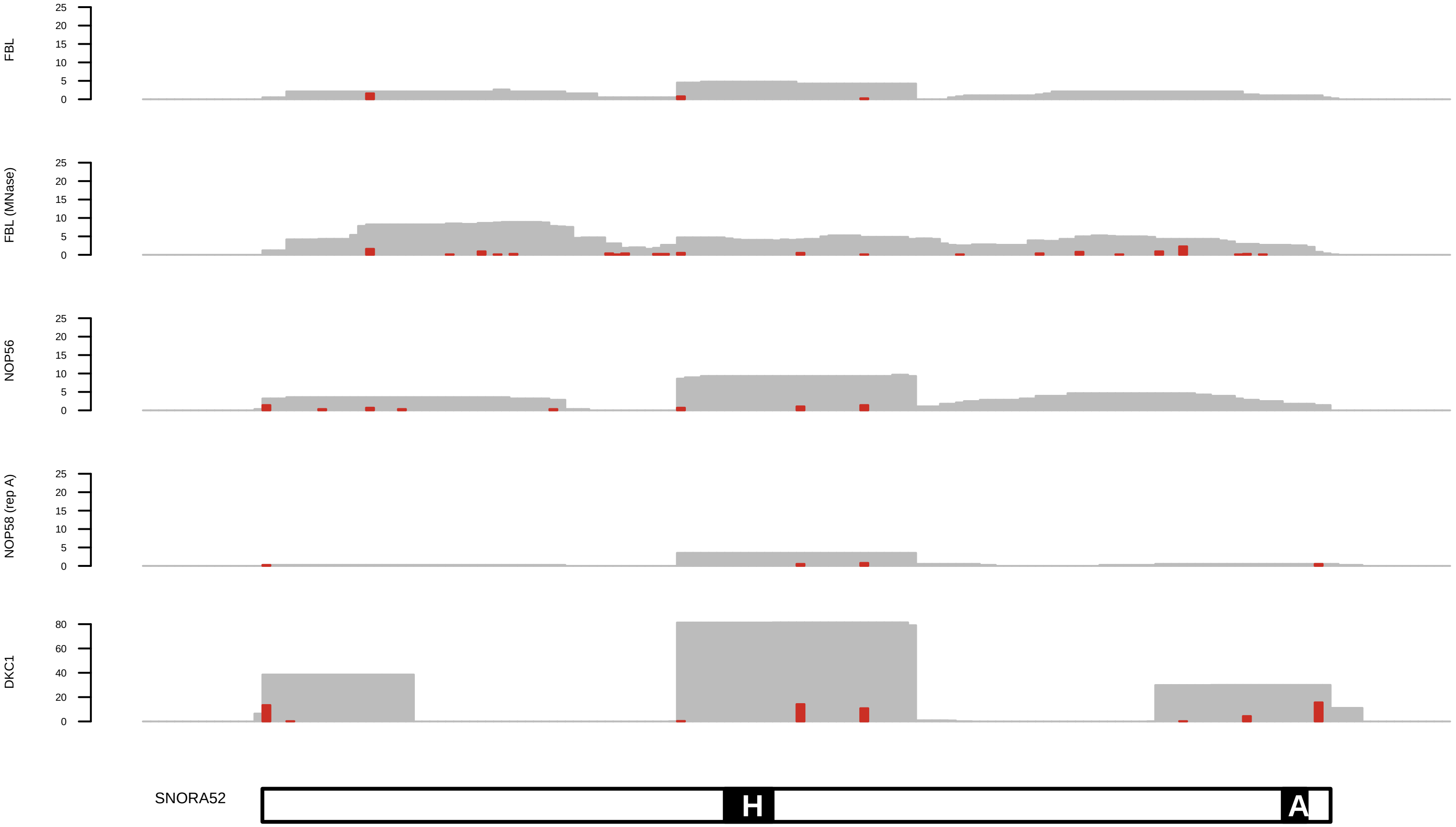

FBL
FBL (MNase)
NOP56
NOP58 (rep A)
DKC1
0
5
10
15
20
25
0
20
40
60
80
SNORA52
H
A

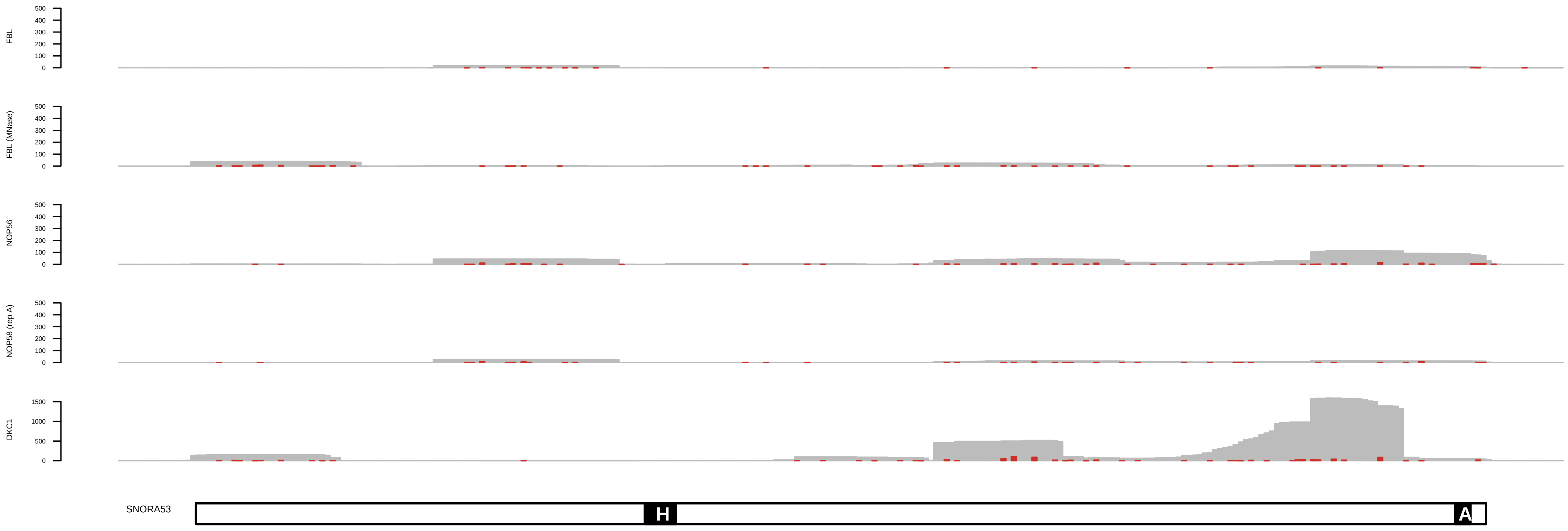
FBL
FBL (MNase)
NOP56
NOP58 (rep A)
DKC1
SNORA53
H
A

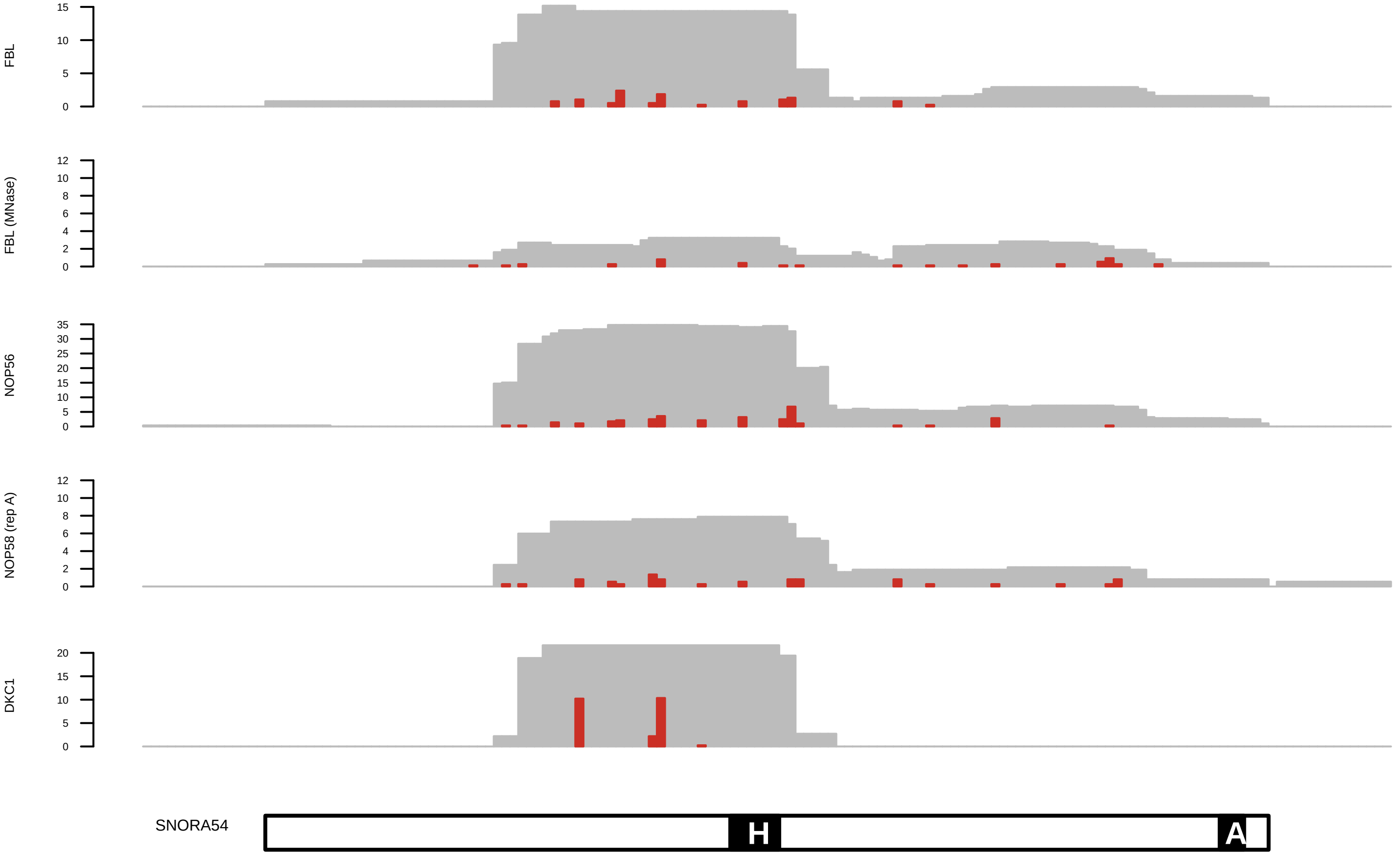
FBL
15
10
5
0
FBL (MNase)
12
10
8
6
4
2
0
NOP56
35
30
25
20
15
10
5
0
NOP58 (rep A)
12
10
8
6
4
2
0
DKC1
20
15
10
5
0
SNORA54
H
A

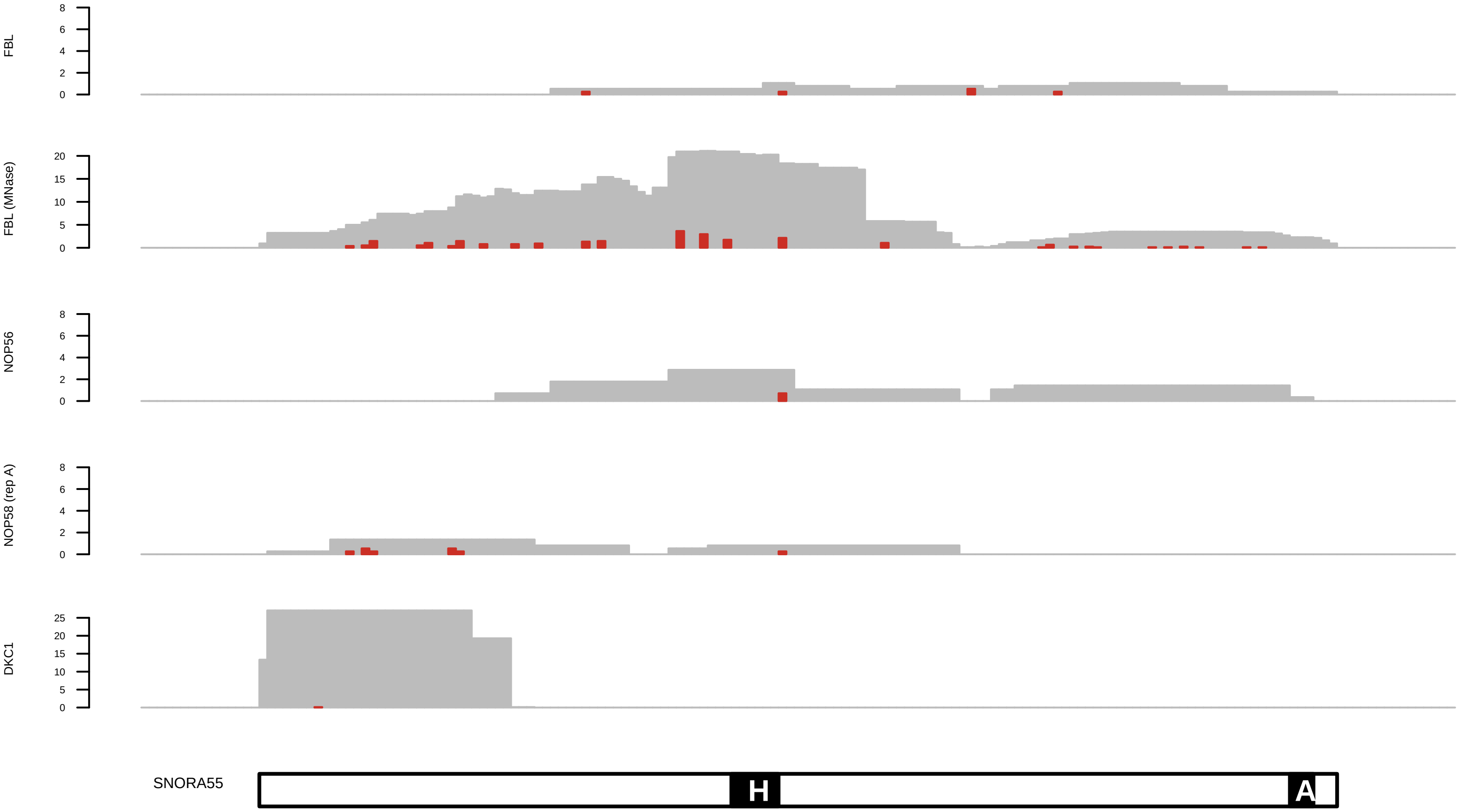
FBL
8
6
4
2
0
FBL (MNase)
20
15
10
5
0
NOP56
8
6
4
2
0
NOP58 (rep A)
8
6
4
2
0
DKC1
25
20
15
10
5
0
SNORA55
H
A

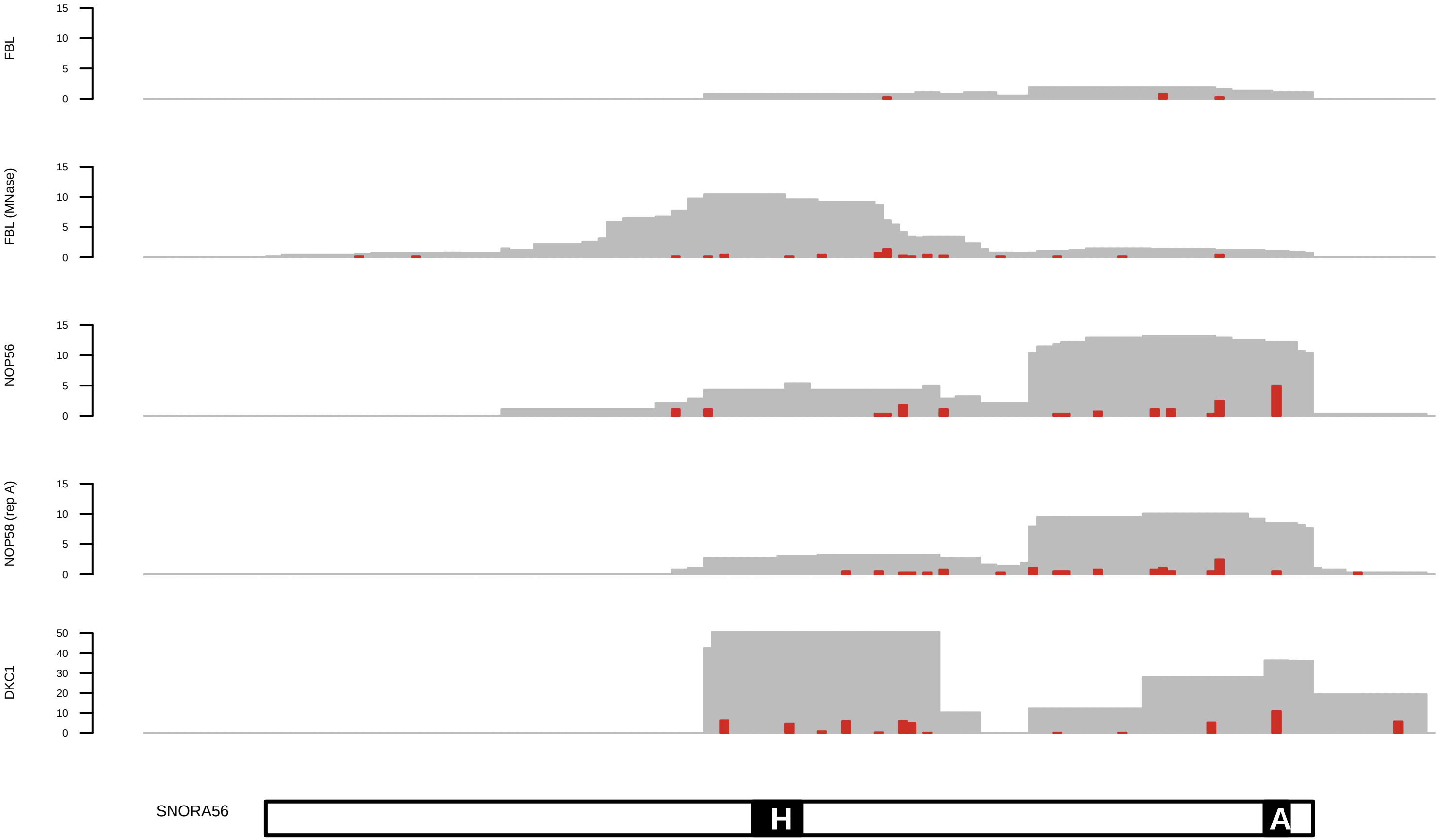
FBL
FBL (MNase)
NOP56
NOP58 (rep A)
DKC1
0
5
10
15
20
30
40
50
SNORA56
H
A

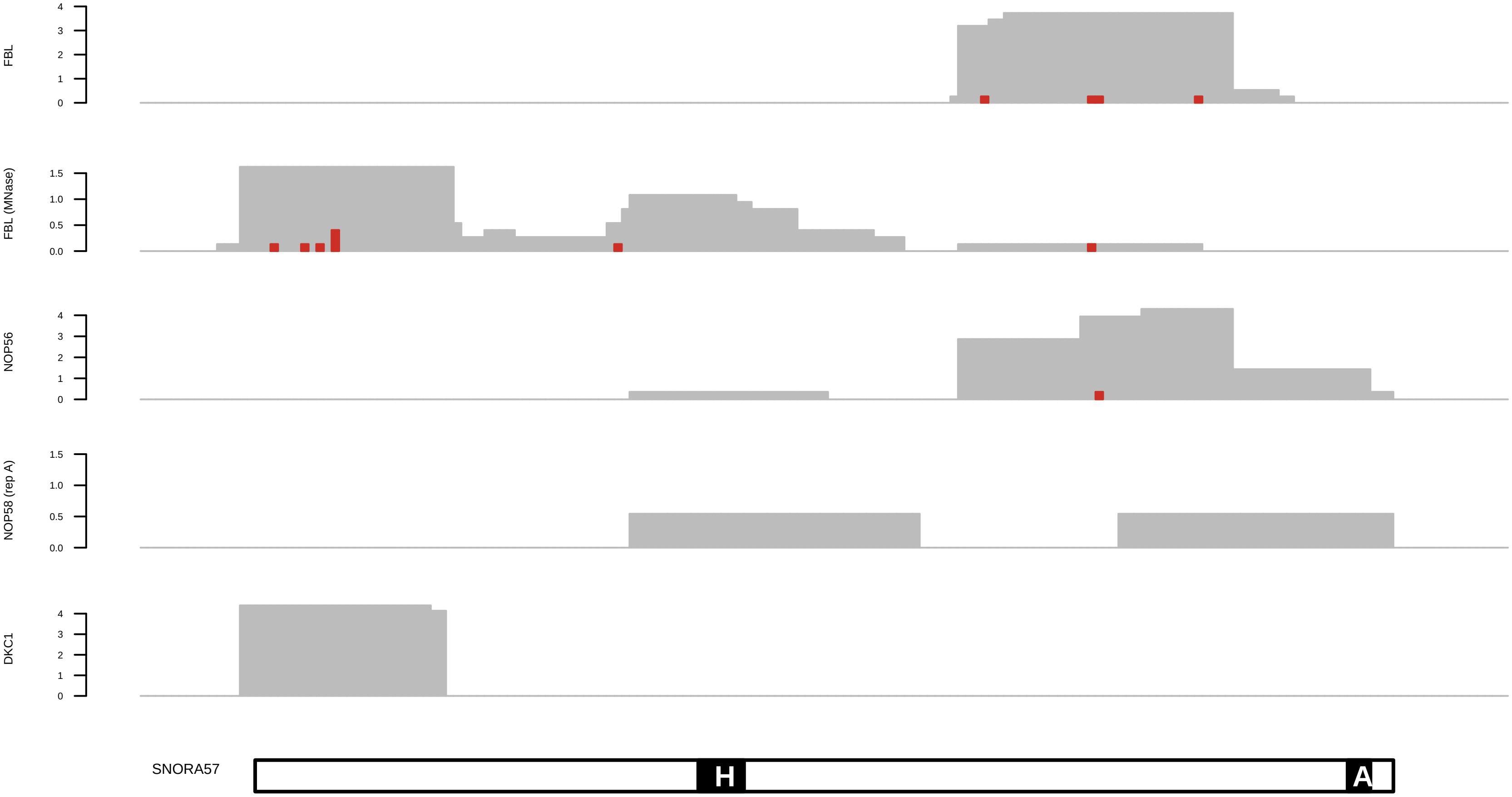
FBL
4
3
2
1
0
FBL (MNase)
1.5
1.0
0.5
0.0
NOP56
4
3
2
1
0
NOP58 (rep A)
1.5
1.0
0.5
0.0
DKC1
4
3
2
1
0
SNORA57
H
A

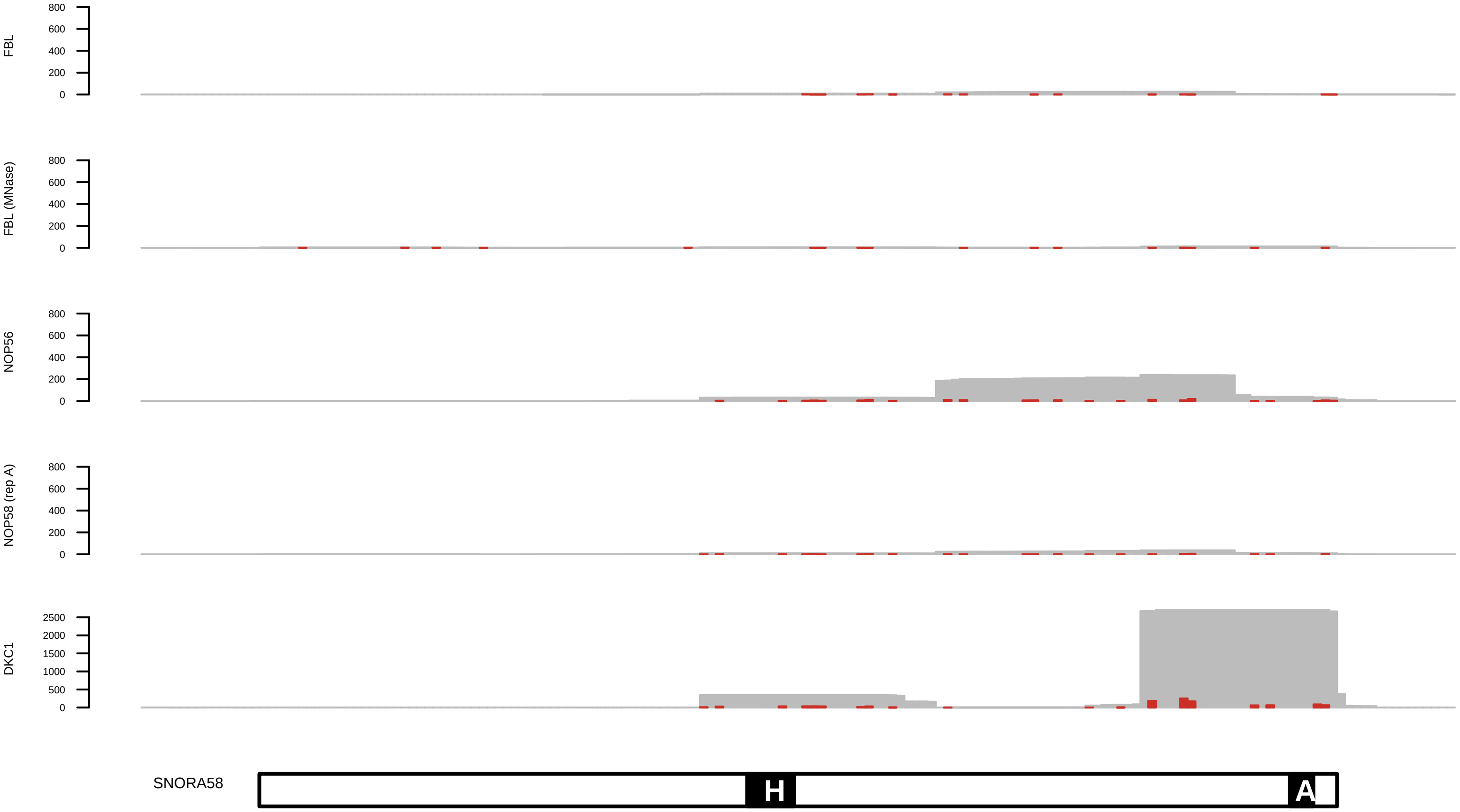
FBL
800
600
400
200
0
FBL (MNase)
800
600
400
200
0
NOP56
800
600
400
200
0
NOP58 (rep A)
800
600
400
200
0
DKC1
2500
2000
1500
1000
500
0
SNORA58
H
A

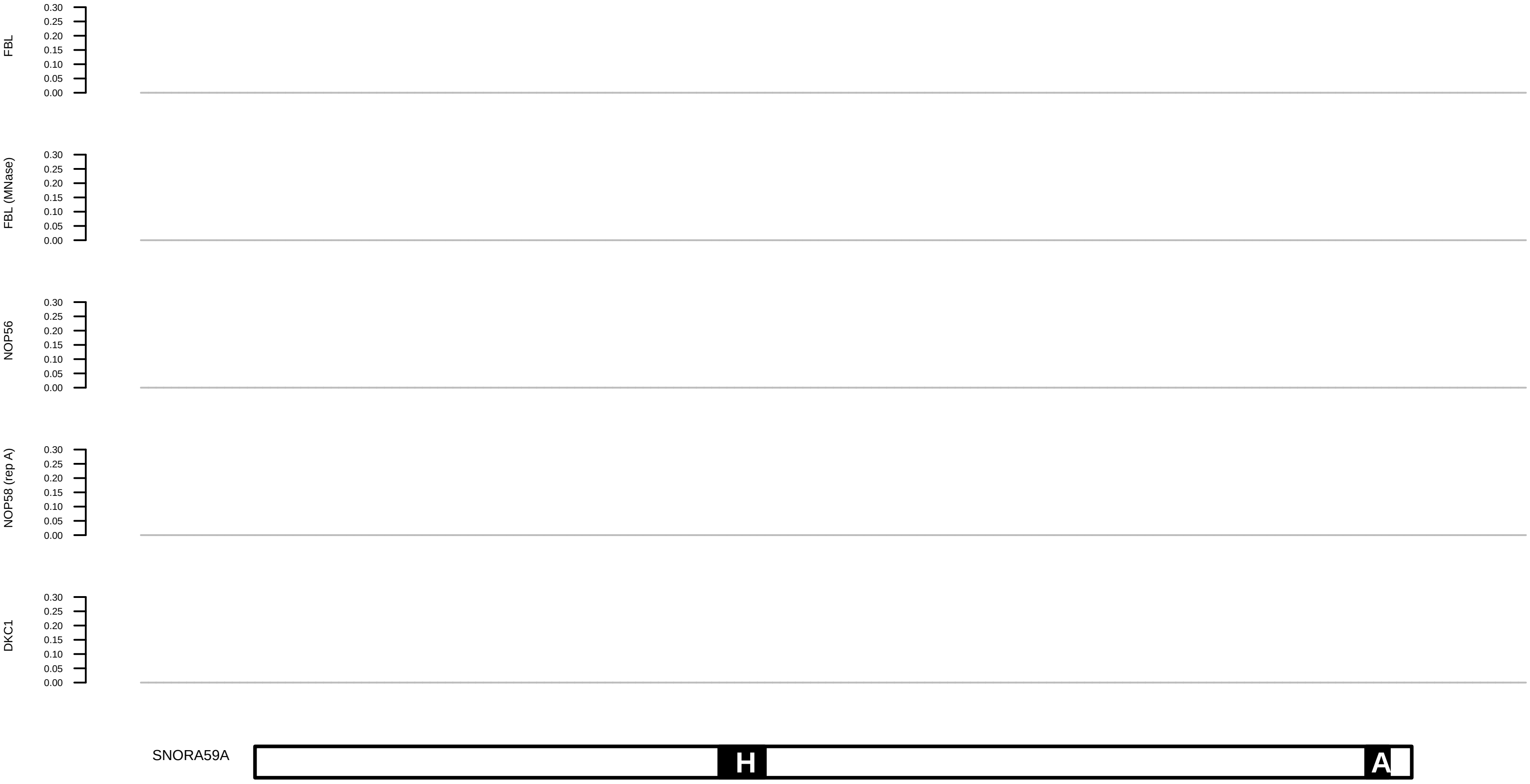
FBL
FBL (MNase)
NOP56
NOP58 (rep A)
DKC1
0.30
0.25
0.20
0.15
0.10
0.05
0.00
SNORA59A
H
A

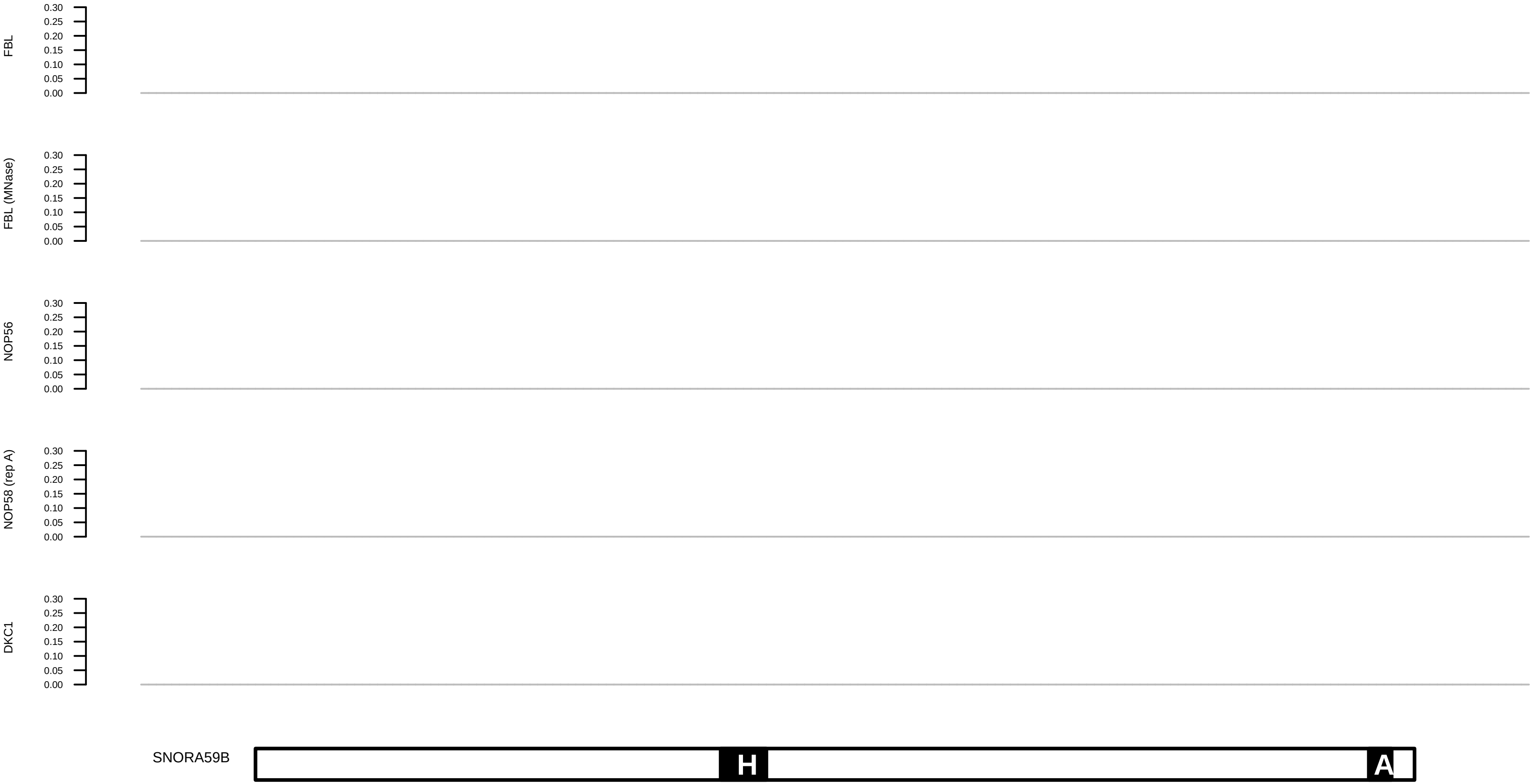
FBL
FBL (MNase)
NOP56
NOP58 (rep A)
DKC1
0.30
0.25
0.20
0.15
0.10
0.05
0.00
SNORA59B
H
A

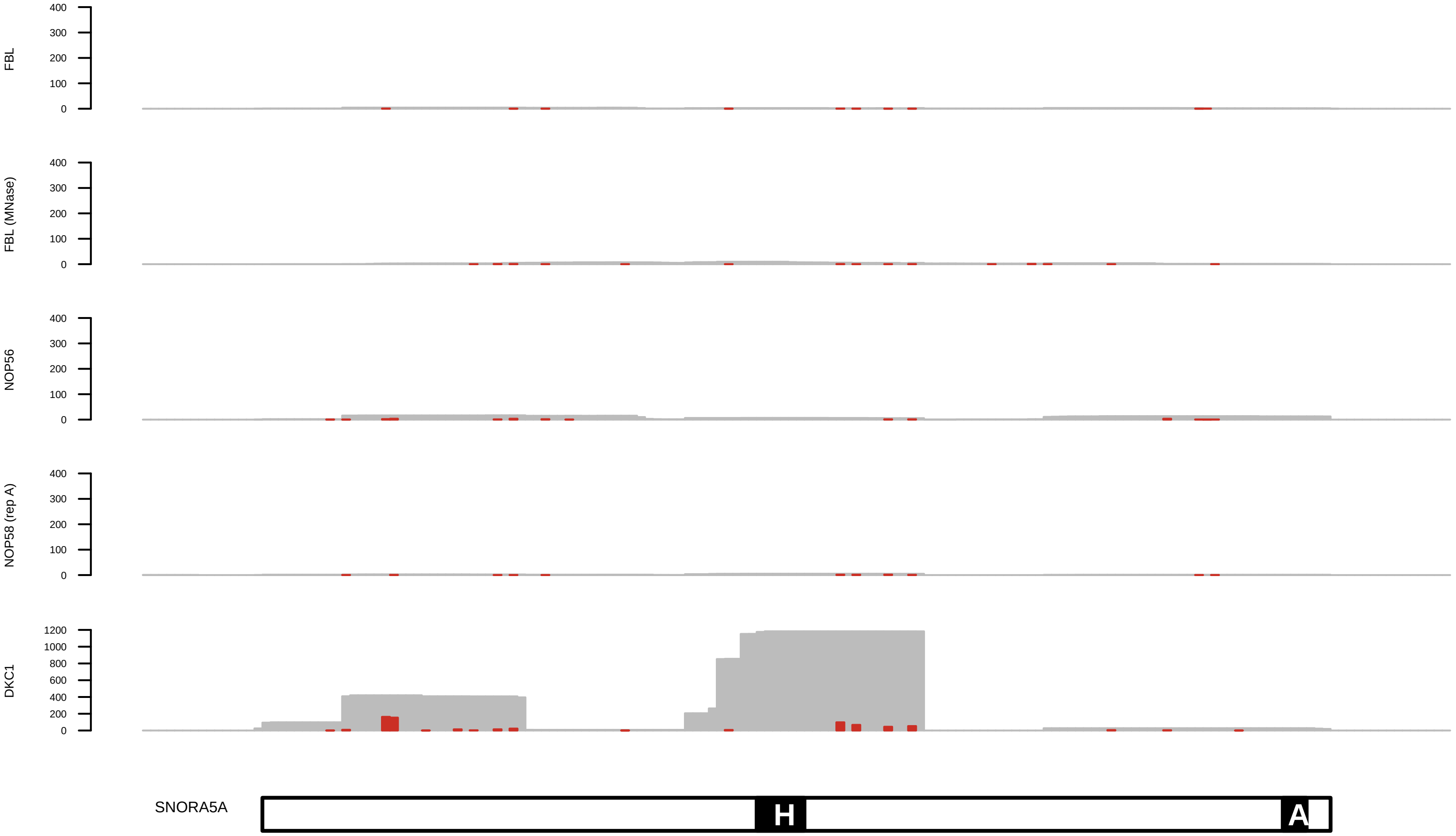

FBL
400
300
200
100
0
FBL (MNase)
400
300
200
100
0
NOP56
400
300
200
100
0
NOP58 (rep A)
400
300
200
100
0
DKC1
1200
1000
800
600
400
200
0
SNORA5A
H
A

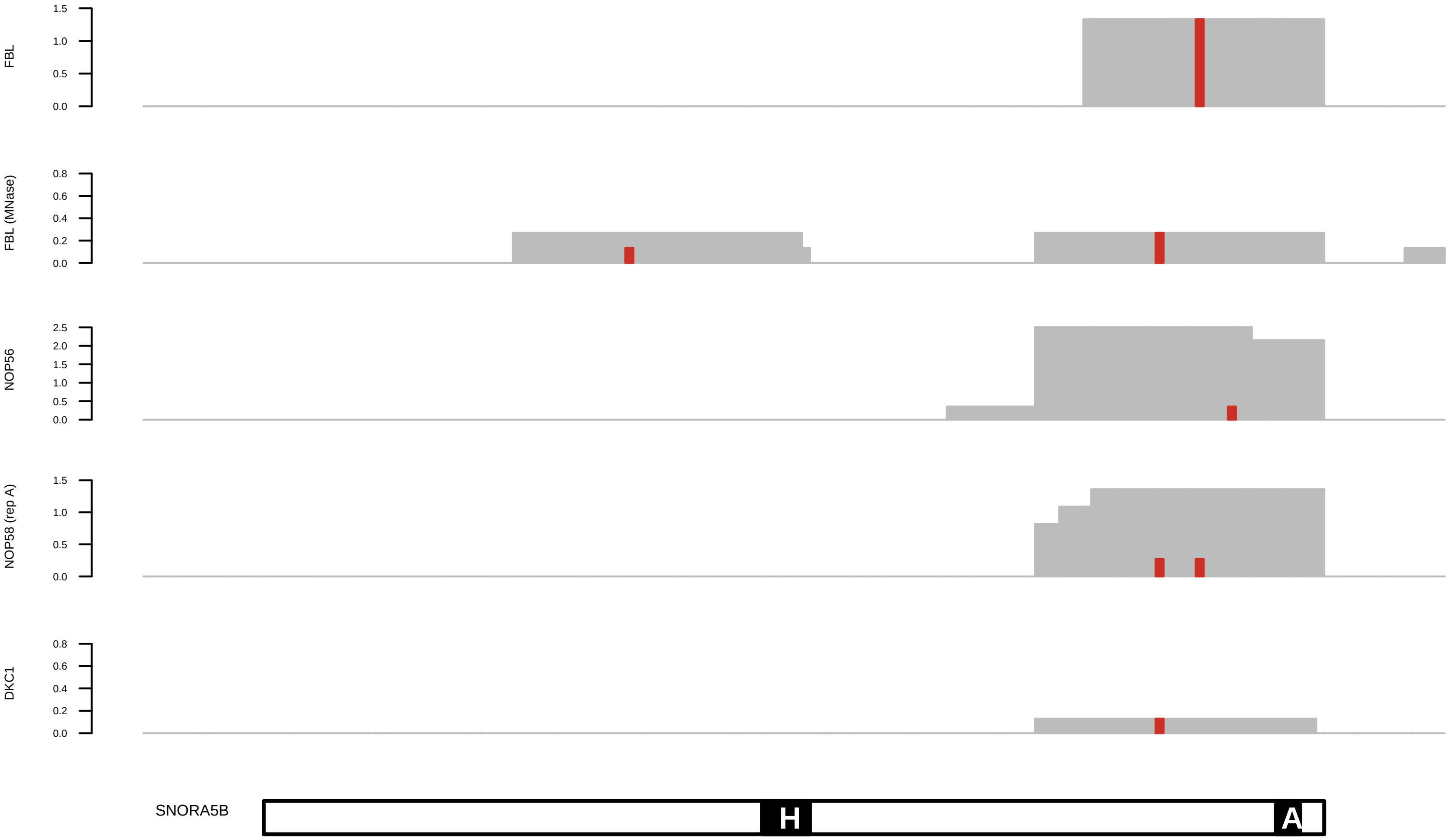

FBL
1.5
1.0
0.5
0.0
FBL (MNase)
0.8
0.6
0.4
0.2
0.0
NOP56
2.5
2.0
1.5
1.0
0.5
0.0
NOP58 (rep A)
1.5
1.0
0.5
0.0
DKC1
0.8
0.6
0.4
0.2
0.0
SNORA5B
H
A

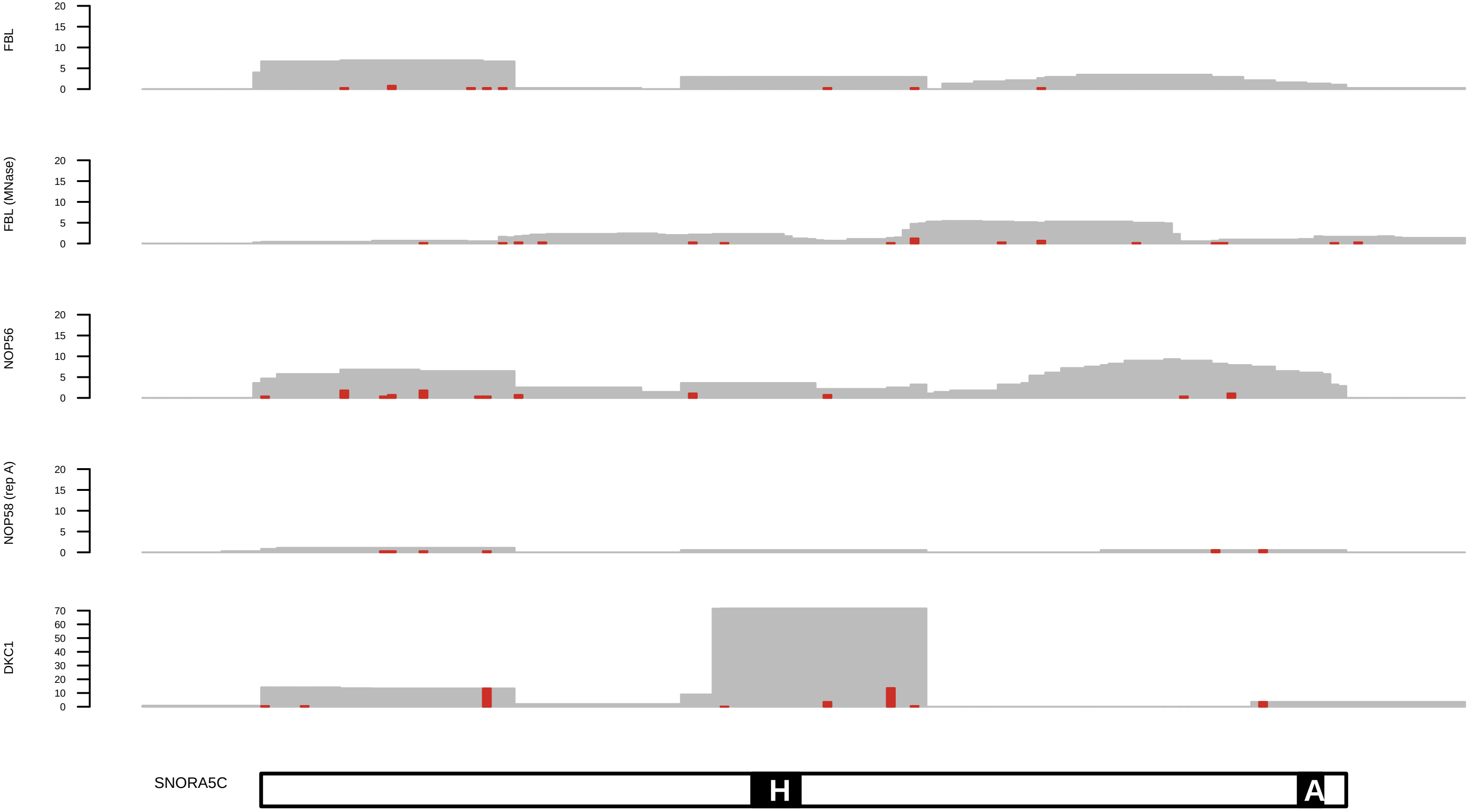
FBL
FBL (MNase)
NOP56
NOP58 (rep A)
DKC1
SNORA5C
H
A

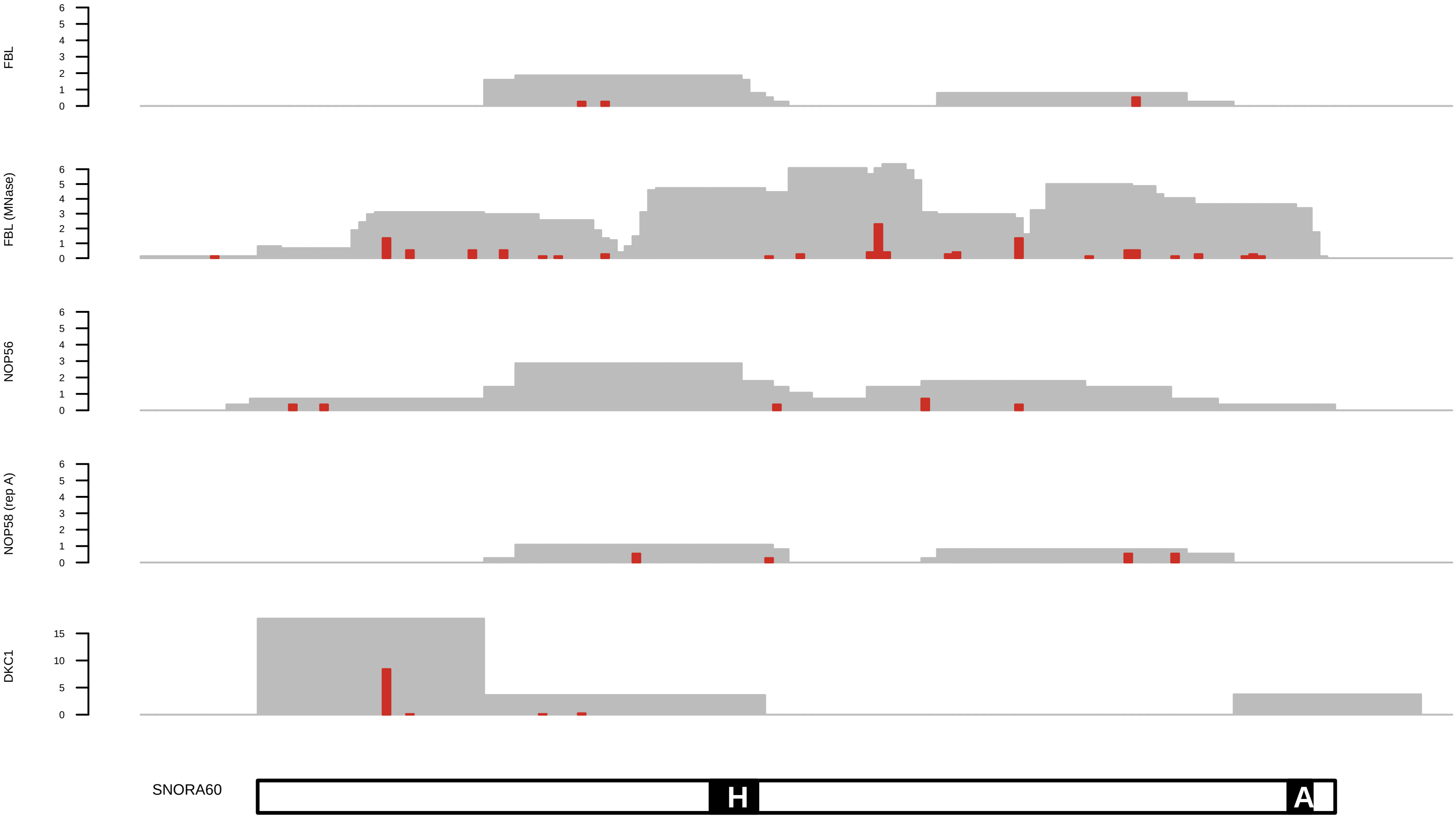
FBL
FBL (MNase)
NOP56
NOP58 (rep A)
DKC1
SNORA60
H
A

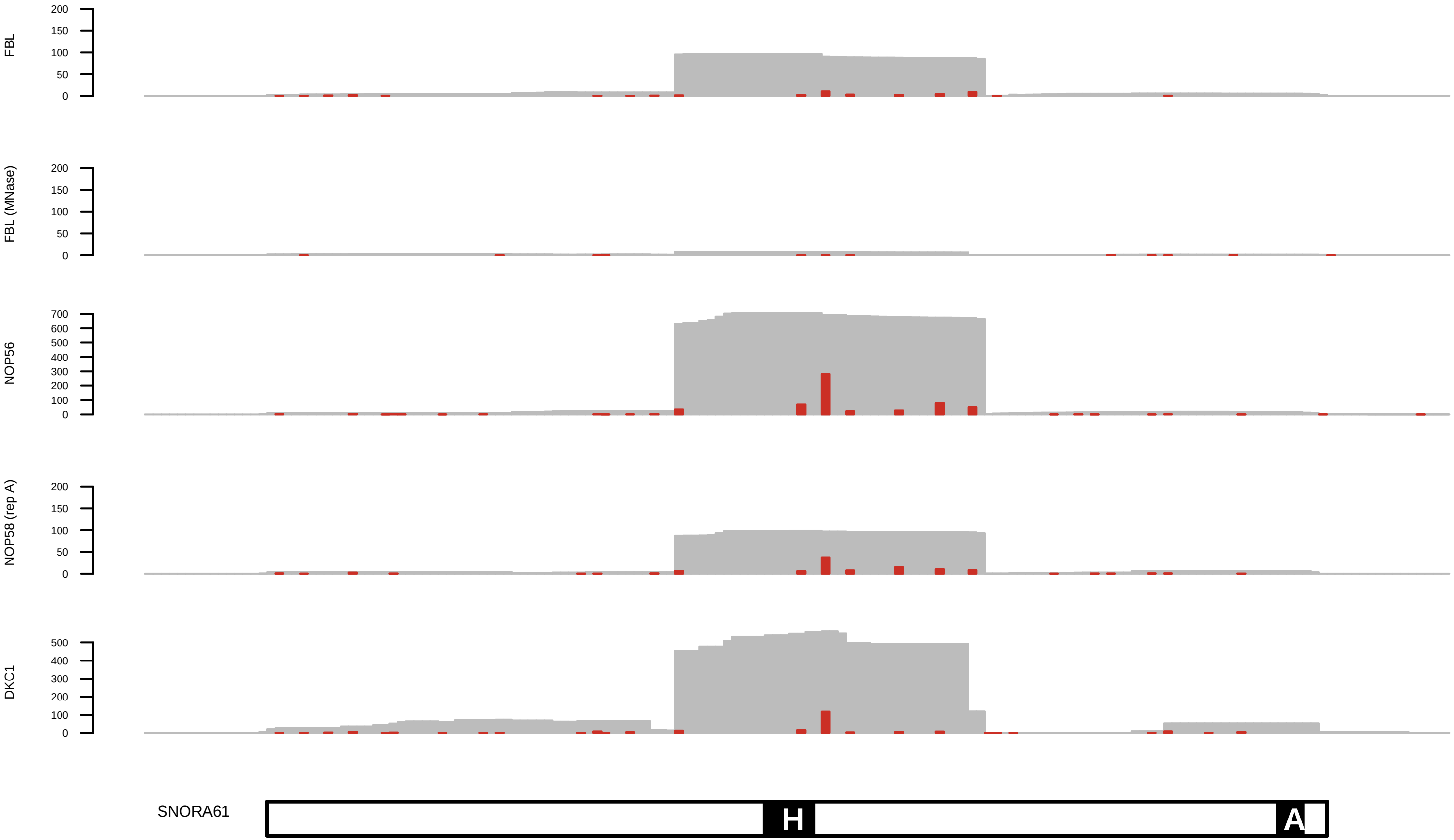

FBL
200
150
100
50
0
FBL (MNase)
200
150
100
50
0
NOP56
700
600
500
400
300
200
100
0
NOP58 (rep A)
200
150
100
50
0
DKC1
500
400
300
200
100
0
SNORA61
H
A

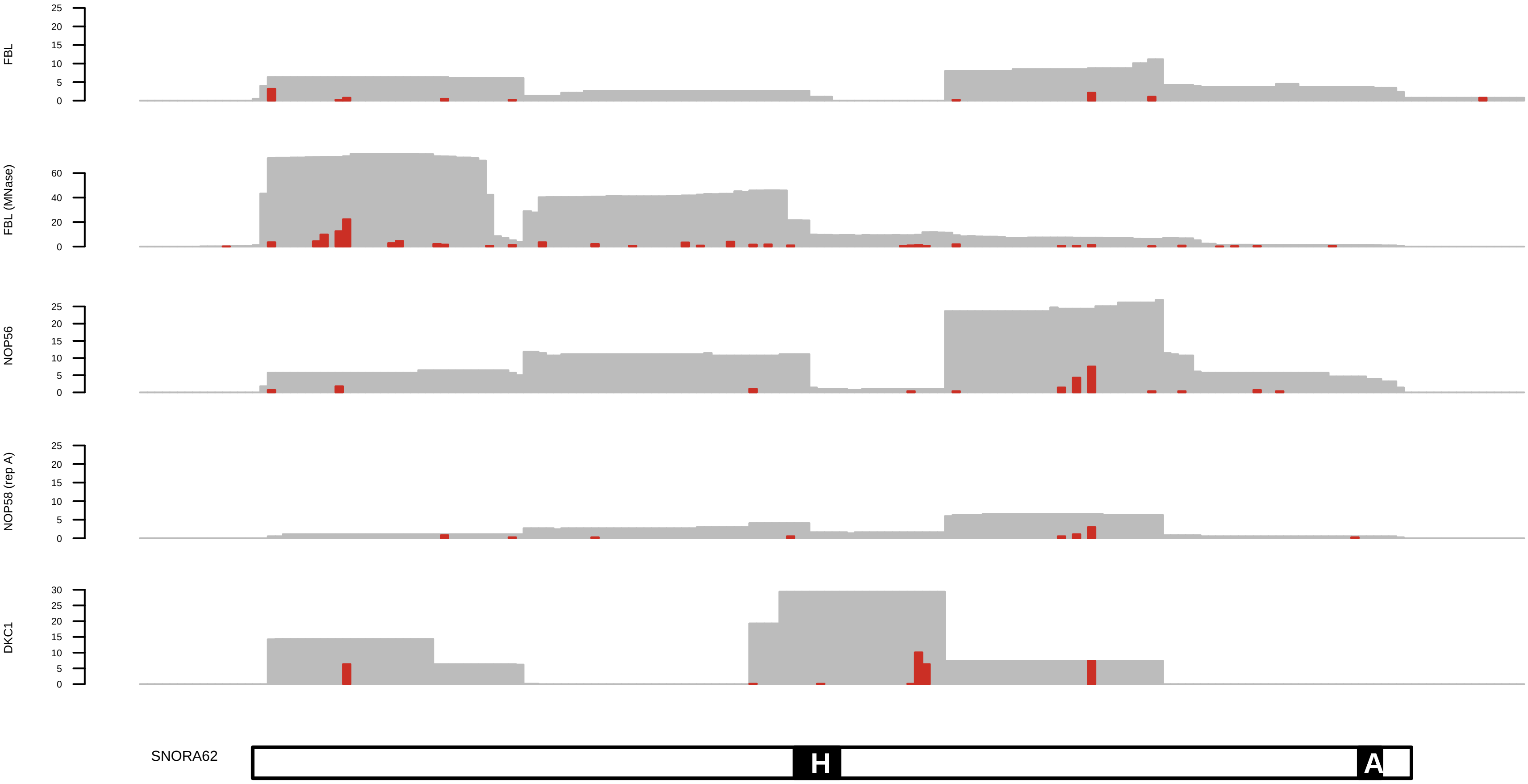

FBL
FBL (MNase)
NOP56
NOP58 (rep A)
DKC1
SNORA62
H
A

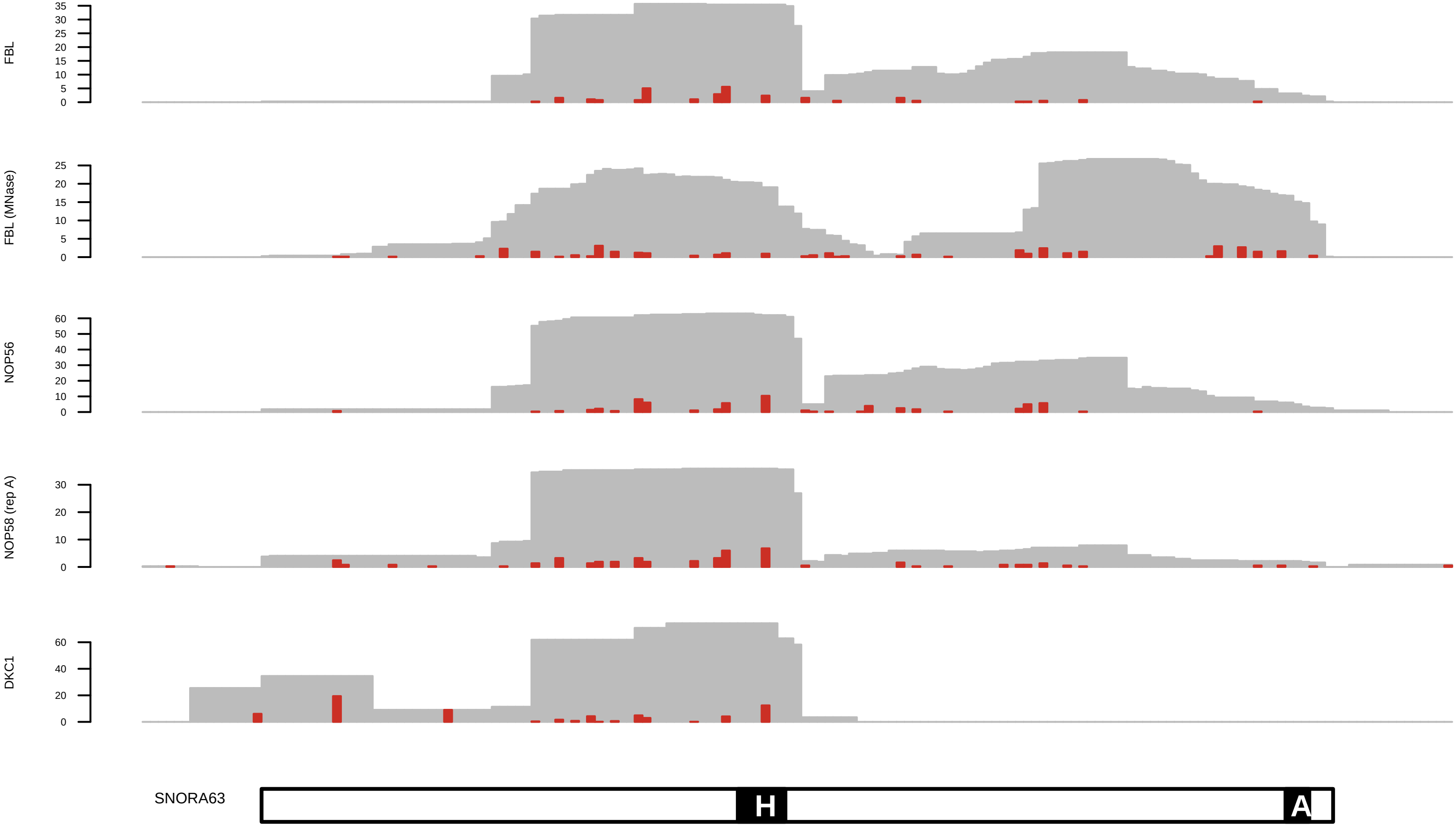

FBL
35
30
25
20
15
10
5
0
FBL (MNase)
25
20
15
10
5
0
NOP56
60
50
40
30
20
10
0
NOP58 (rep A)
30
20
10
0
DKC1
60
40
20
0
SNORA63
H
A

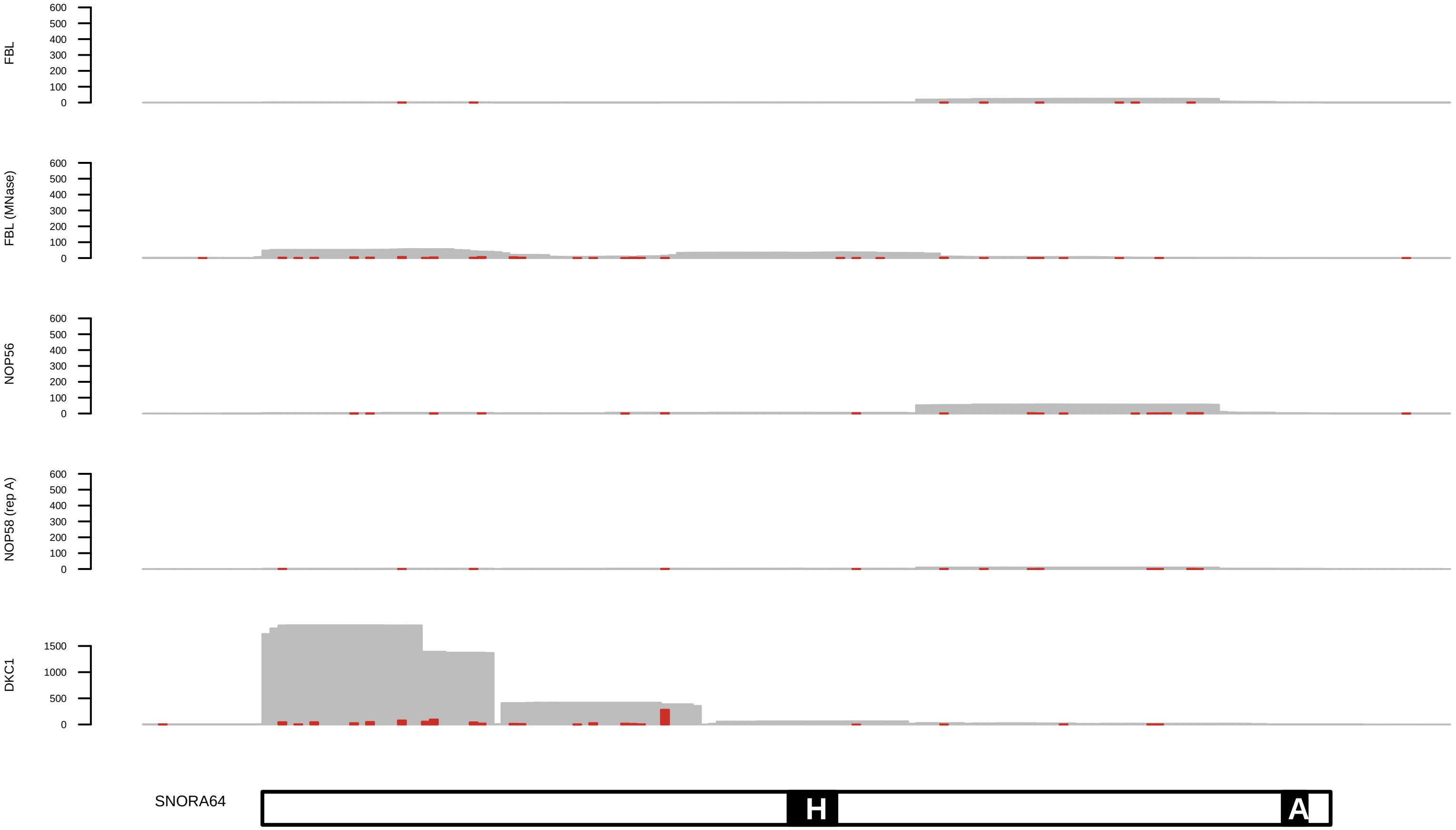

FBL
FBL (MNase)
NOP56
NOP58 (rep A)
DKC1
600
500
400
300
200
100
0
1500
1000
500
SNORA64
H
A

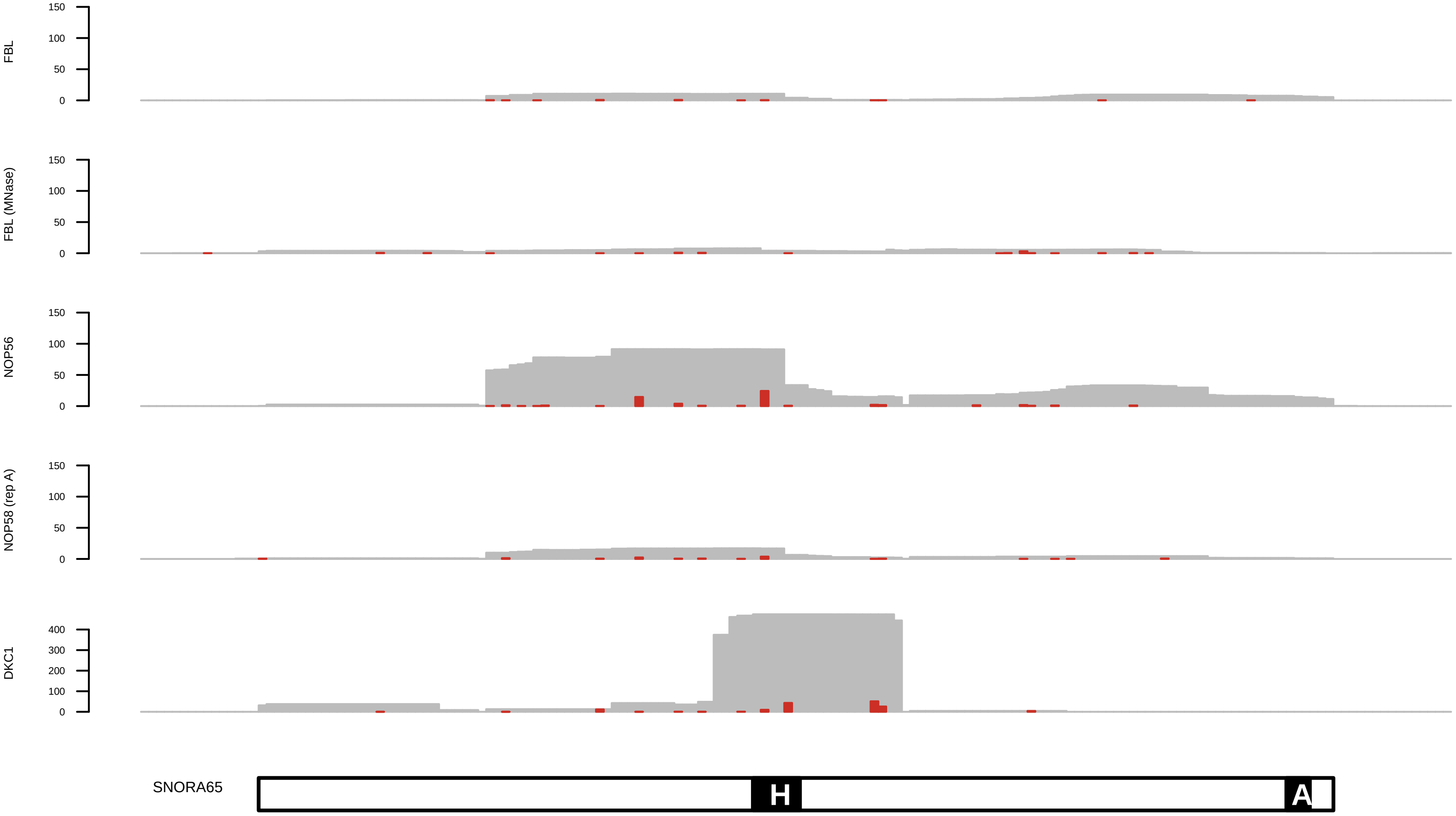

FBL
FBL (MNase)
NOP56
NOP58 (rep A)
DKC1
150
100
50
0
400
300
200
100
0
SNORA65
H
A

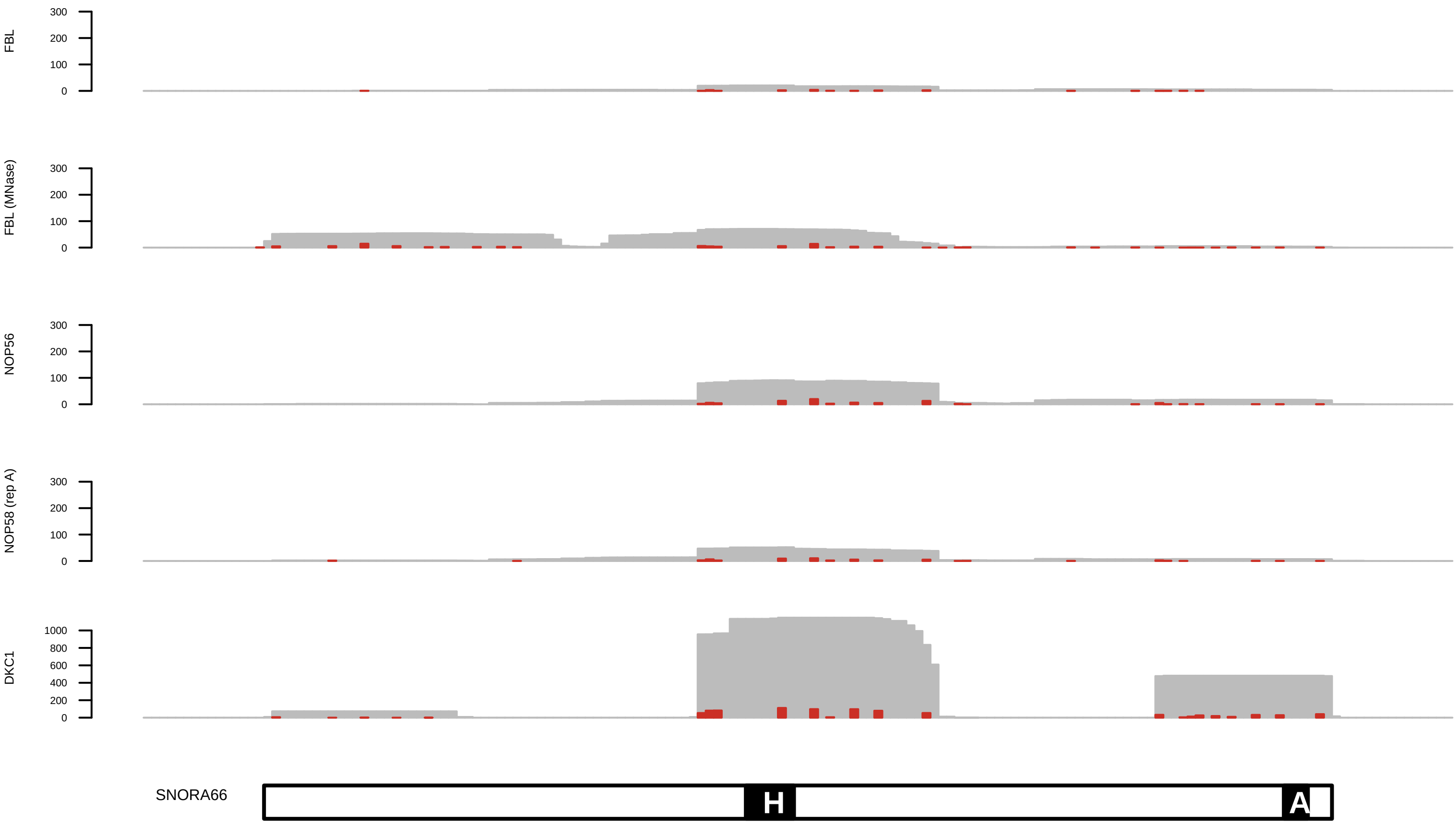
FBL
300
200
100
0
FBL (MNase)
300
200
100
0
NOP56
300
200
100
0
NOP58 (rep A)
300
200
100
0
DKC1
1000
800
600
400
200
0
SNORA66
H
A

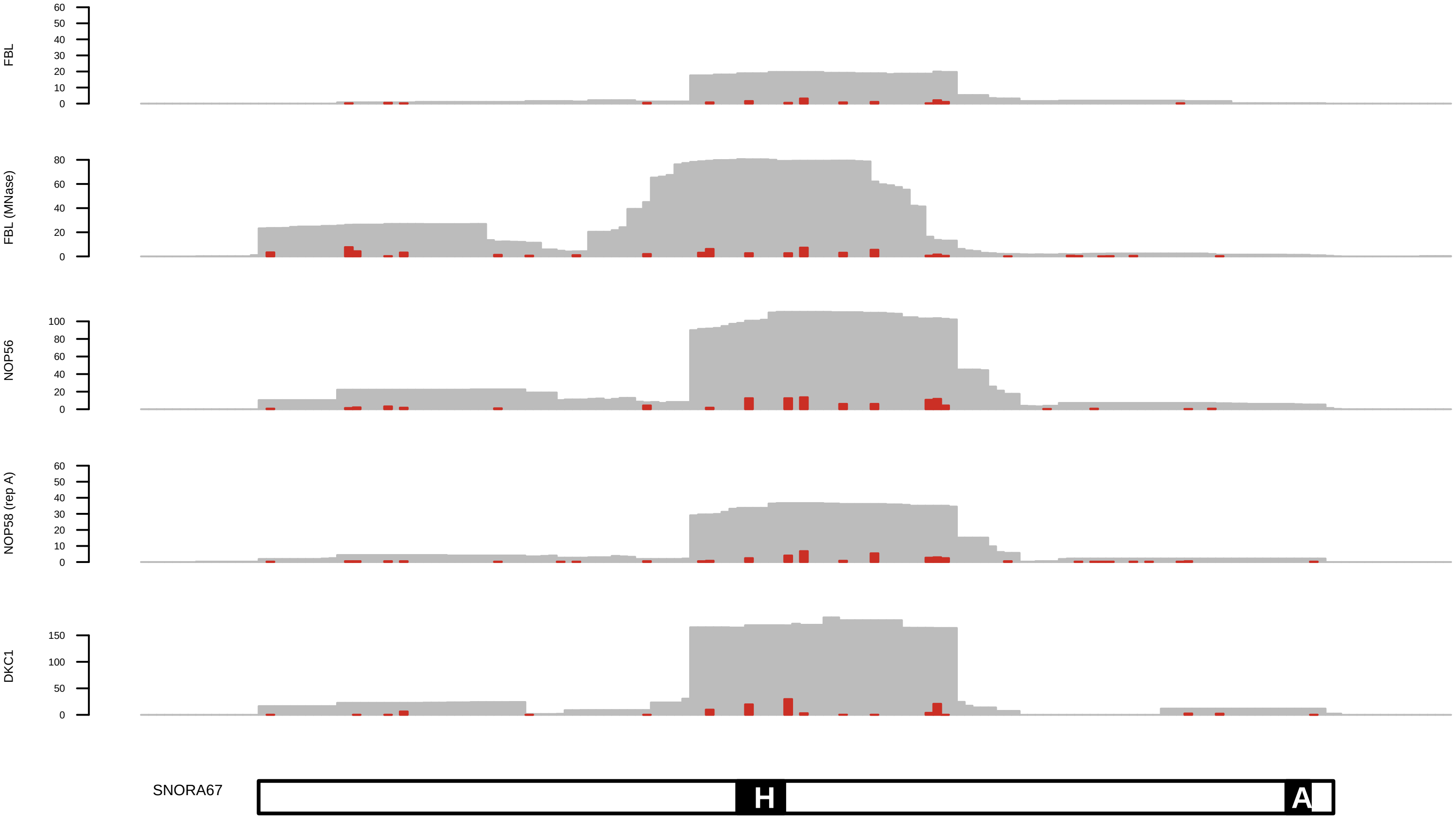
FBL
FBL (MNase)
NOP56
NOP58 (rep A)
DKC1
SNORA67
H
A

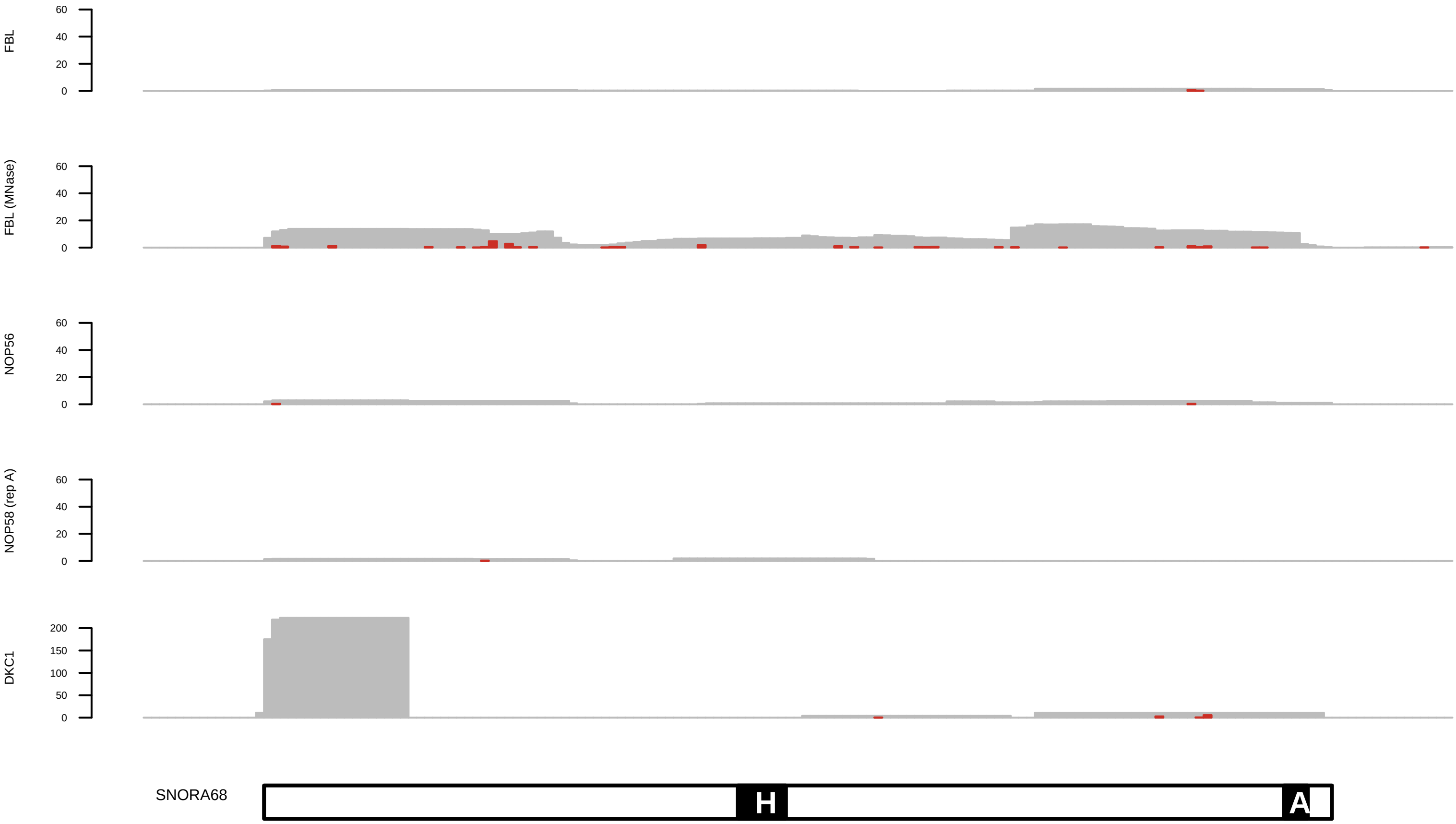
FBL
60
40
20
0
FBL (MNase)
60
40
20
0
NOP56
60
40
20
0
NOP58 (rep A)
60
40
20
0
DKC1
200
150
100
50
0
SNORA68
H
A

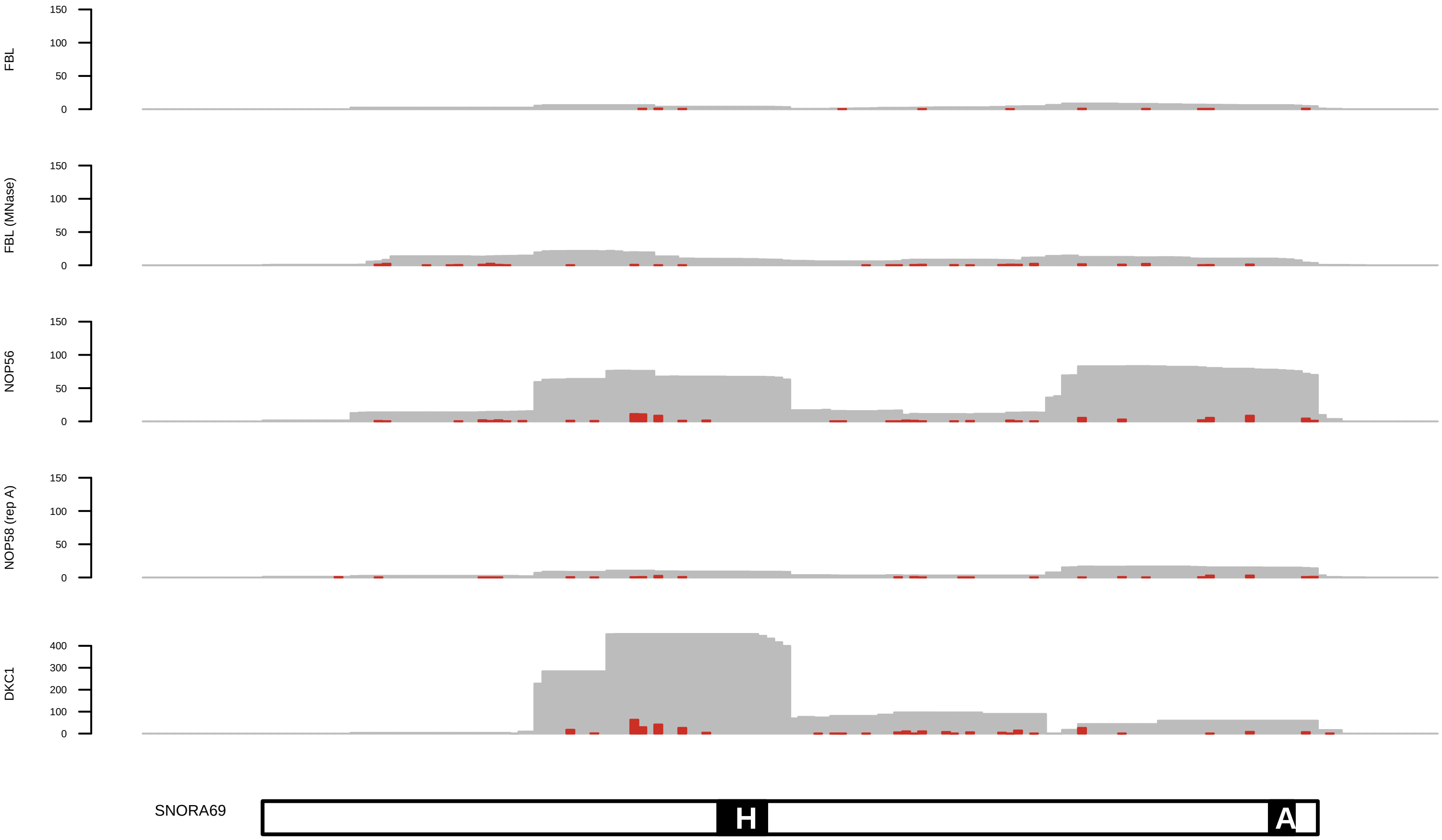
FBL
FBL (MNase)
NOP56
NOP58 (rep A)
DKC1
150
100
50
0
400
300
200
100
SNORA69
H
A

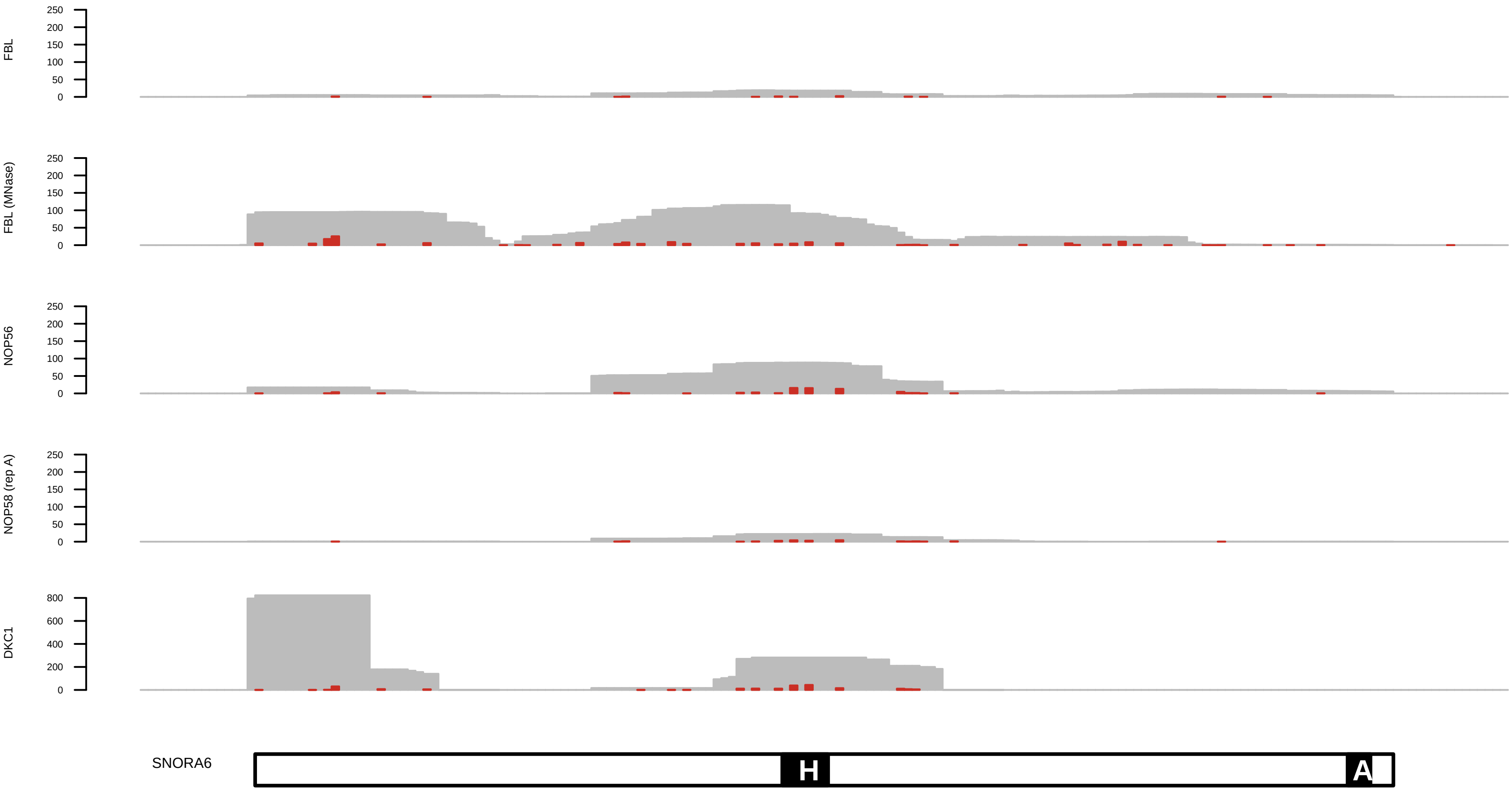
FBL
FBL (MNase)
NOP56
NOP58 (rep A)
DKC1
250
200
150
100
50
0
800
600
400
200
SNORA6
H
A

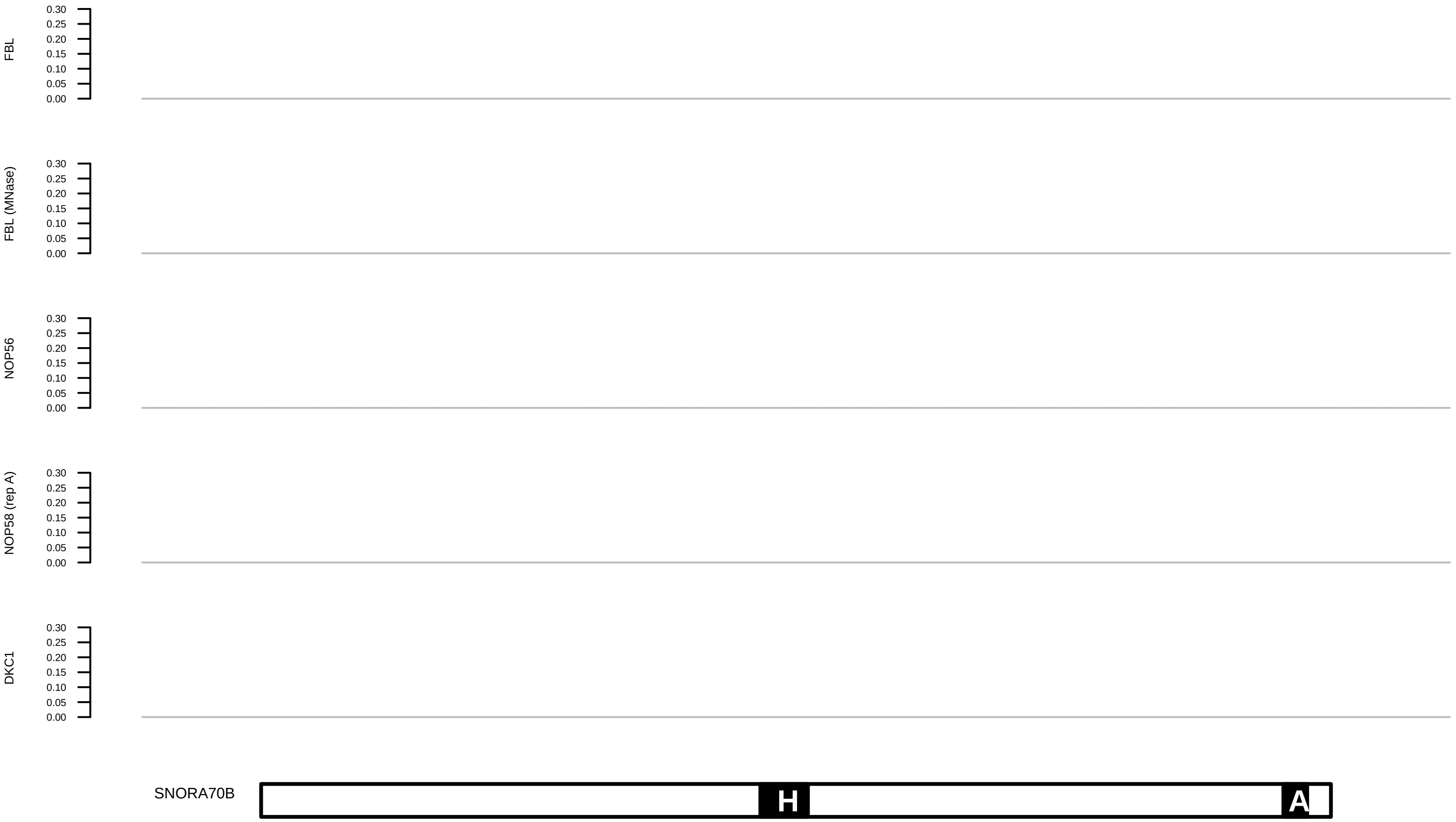
FBL
FBL (MNase)
NOP56
NOP58 (rep A)
DKC1
0.30
0.25
0.20
0.15
0.10
0.05
0.00
SNORA70B
H
A

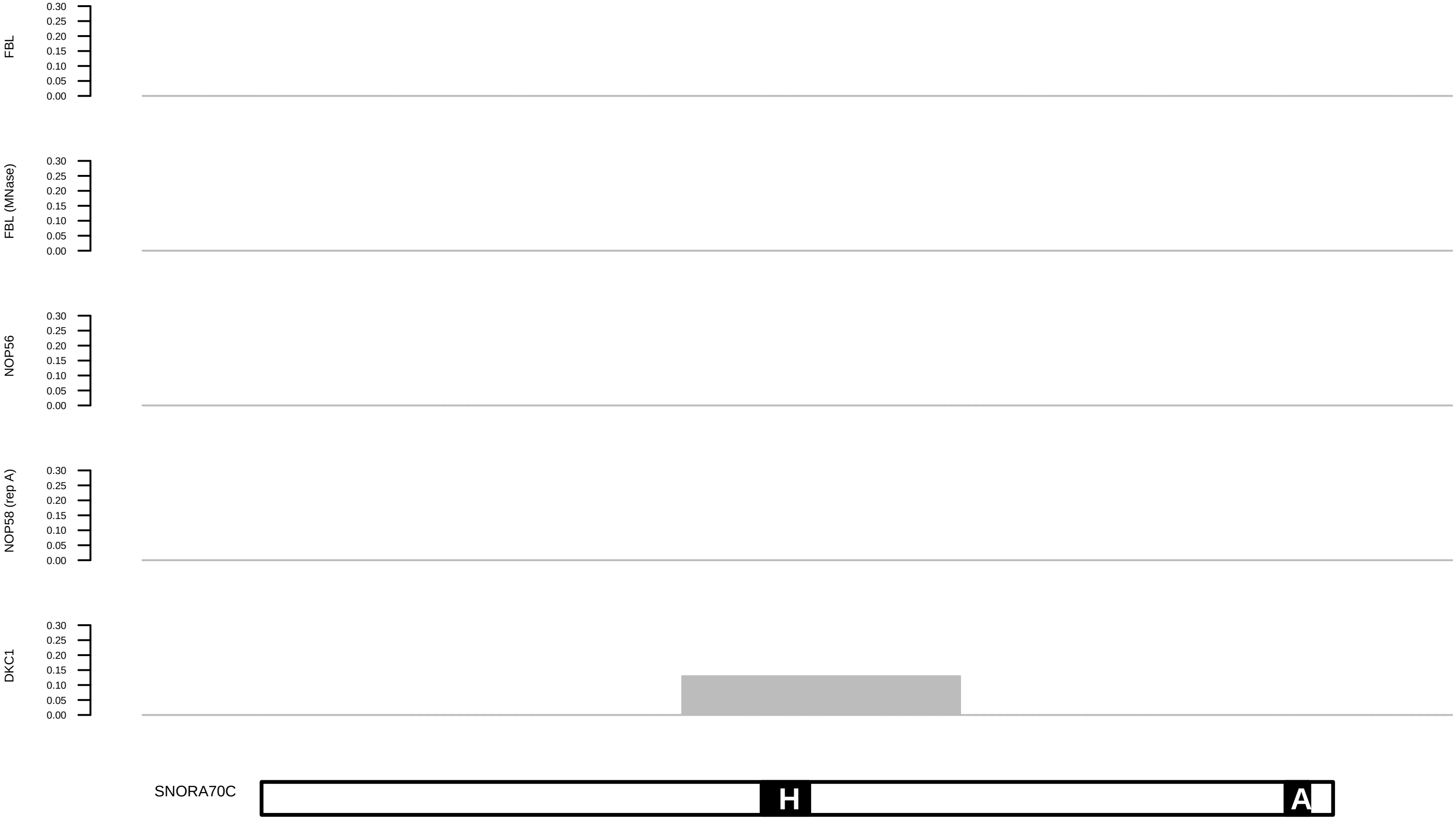
FBL
FBL (MNase)
NOP56
NOP58 (rep A)
DKC1
0.30
0.25
0.20
0.15
0.10
0.05
0.00
SNORA70C
H
A

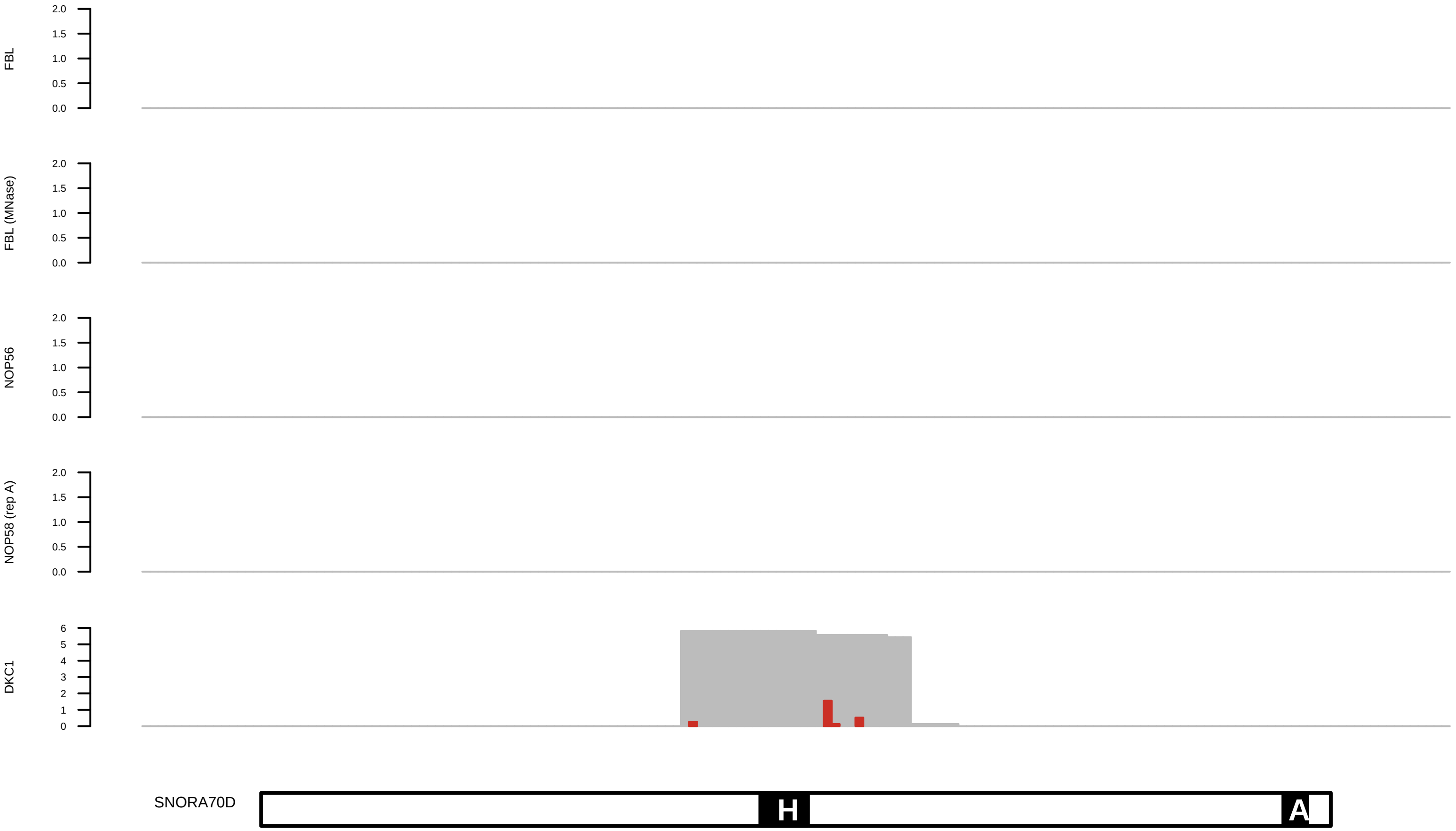
FBL
FBL (MNase)
NOP56
NOP58 (rep A)
DKC1
2.0
1.5
1.0
0.5
0.0
6
5
4
3
2
1
0
SNORA70D
H
A

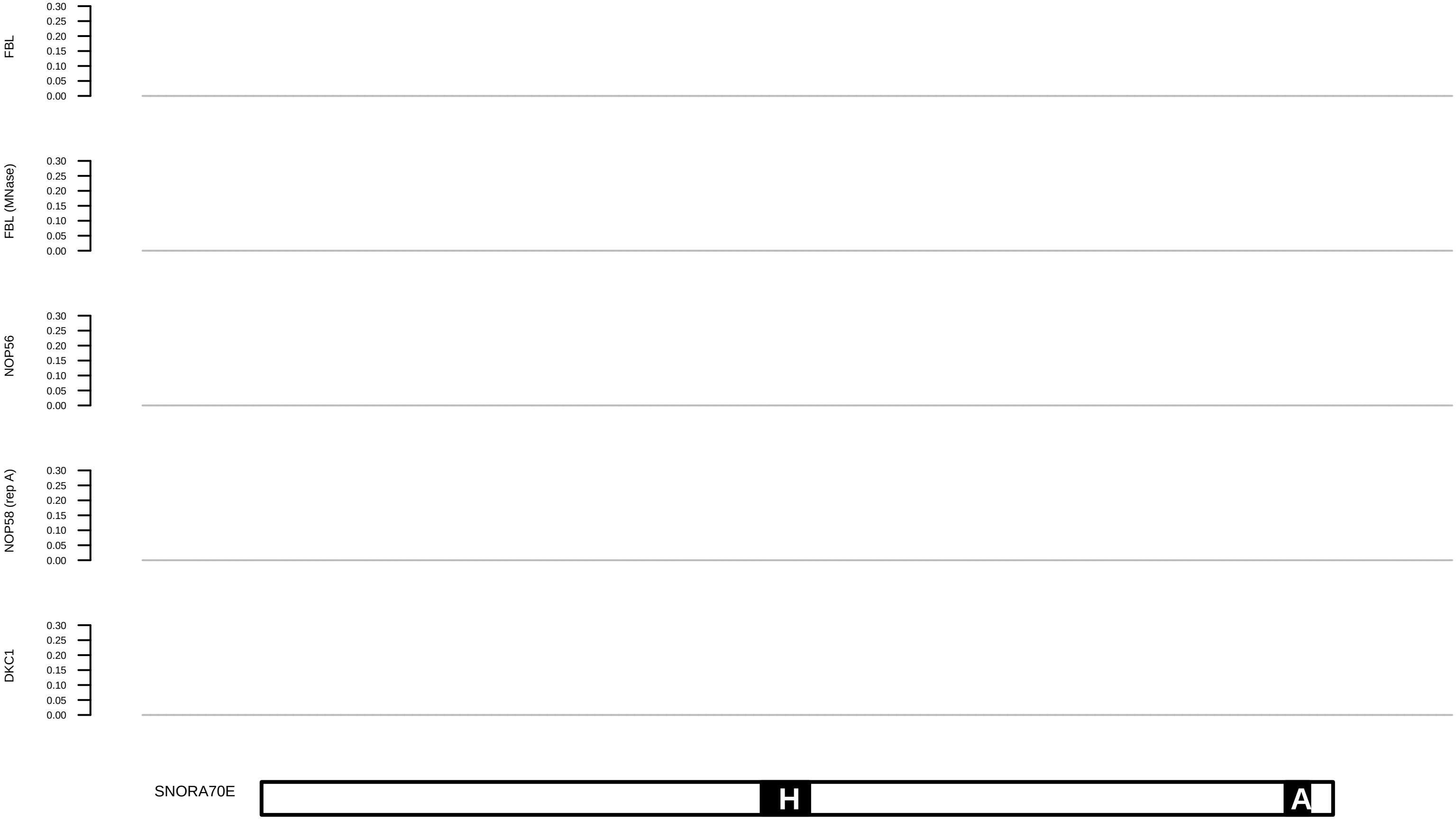
FBL
FBL (MNase)
NOP56
NOP58 (rep A)
DKC1
0.30
0.25
0.20
0.15
0.10
0.05
0.00
SNORA70E
H
A

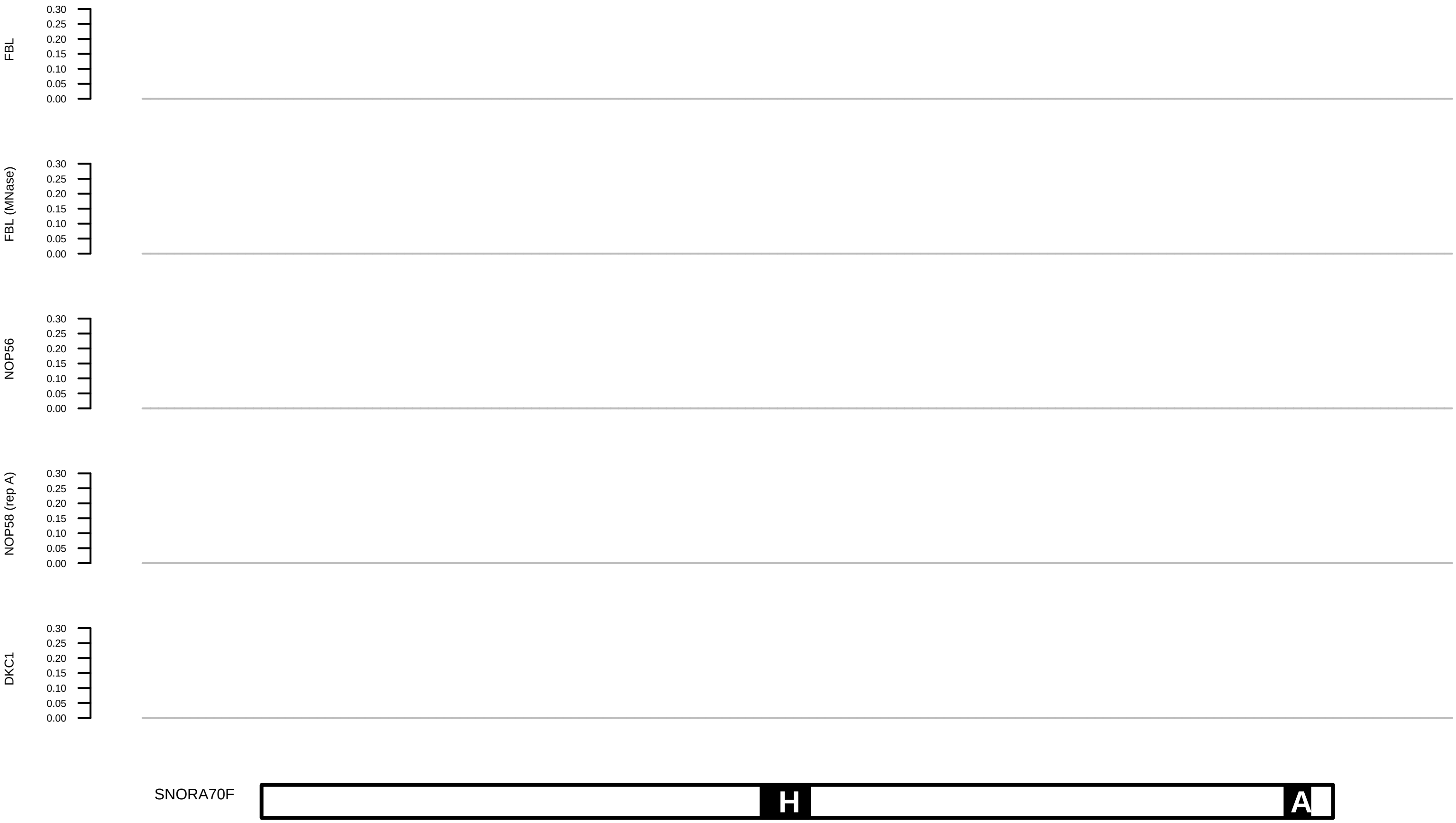
FBL
FBL (MNase)
NOP56
NOP58 (rep A)
DKC1
0.30
0.25
0.20
0.15
0.10
0.05
0.00
SNORA70F
H
A

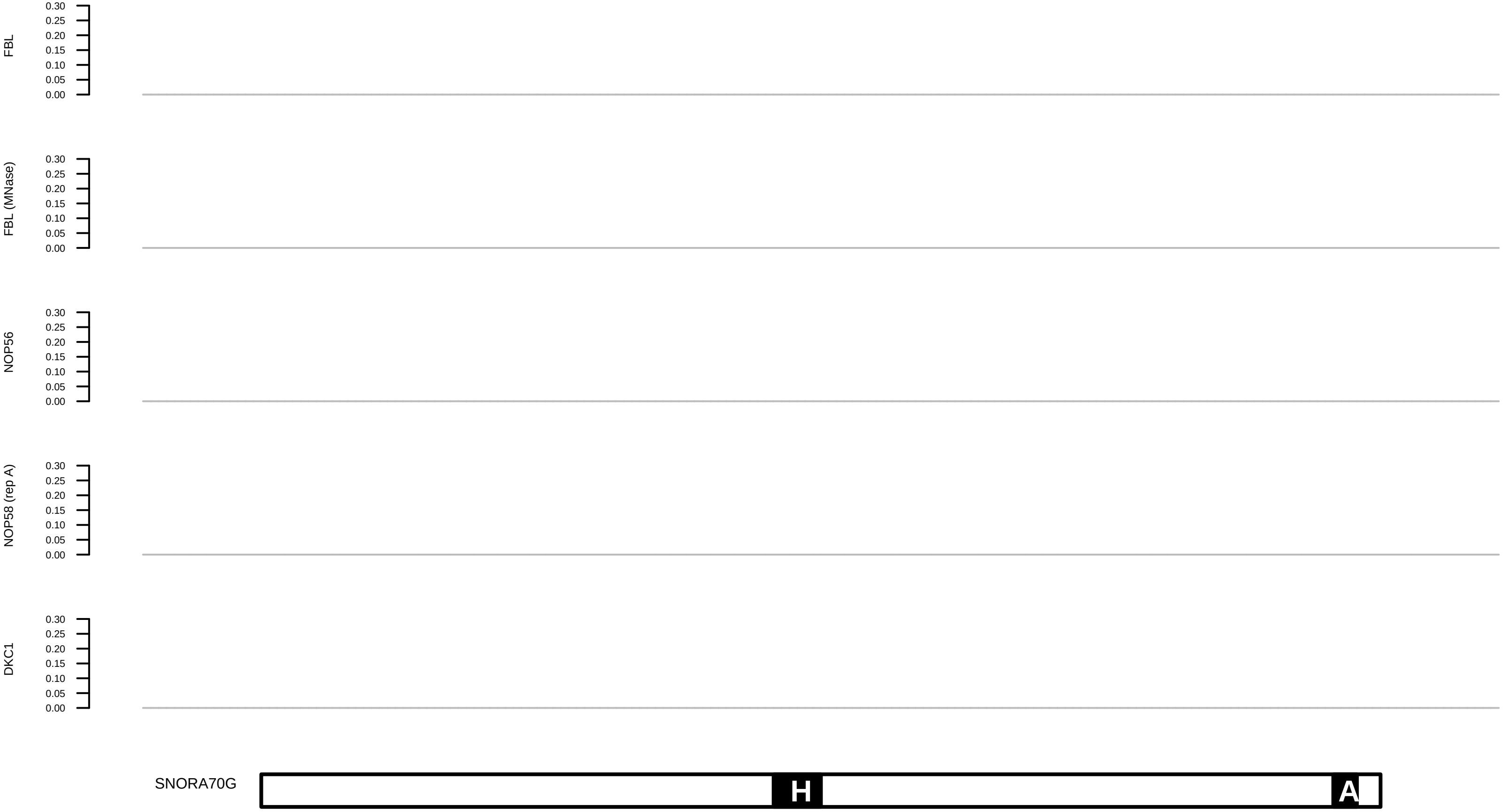
FBL
FBL (MNase)
NOP56
NOP58 (rep A)
DKC1
0.30
0.25
0.20
0.15
0.10
0.05
0.00
SNORA70G
H
A

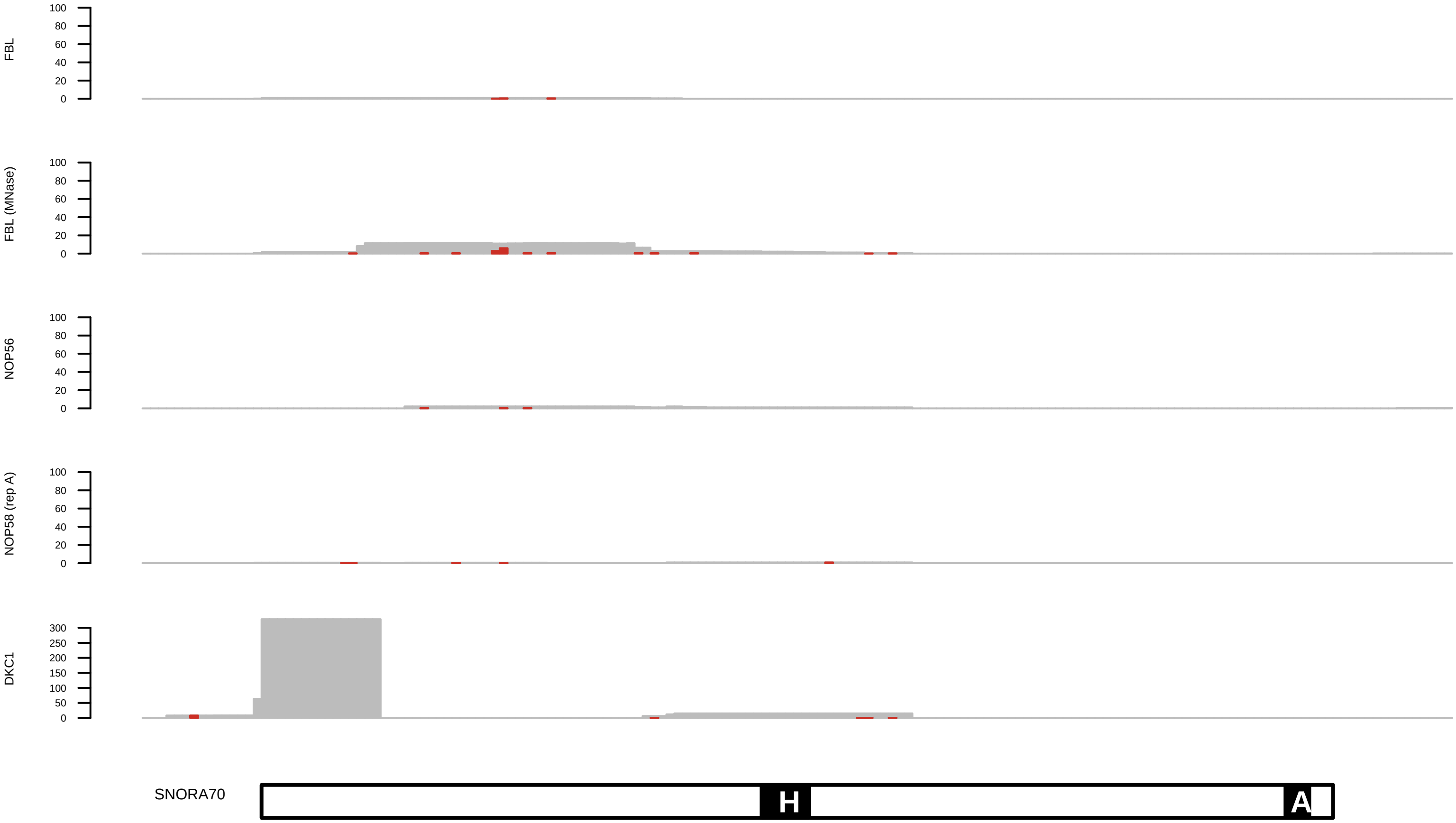

FBL
100
80
60
40
20
0
FBL (MNase)
100
80
60
40
20
0
NOP56
100
80
60
40
20
0
NOP58 (rep A)
100
80
60
40
20
0
DKC1
300
250
200
150
100
50
0
SNORA70
H
A

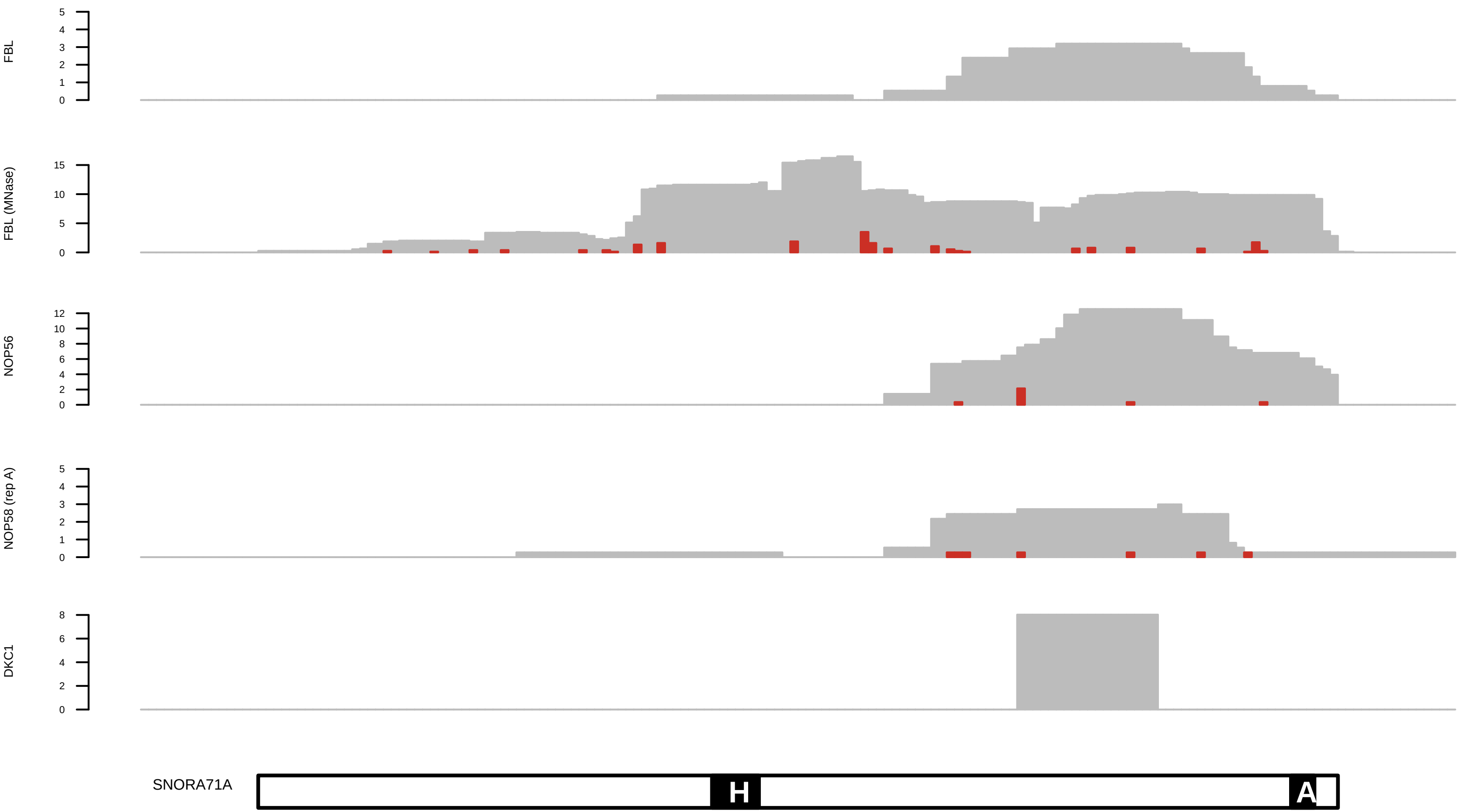
FBL
FBL (MNase)
NOP56
NOP58 (rep A)
DKC1
SNORA71A
H
A

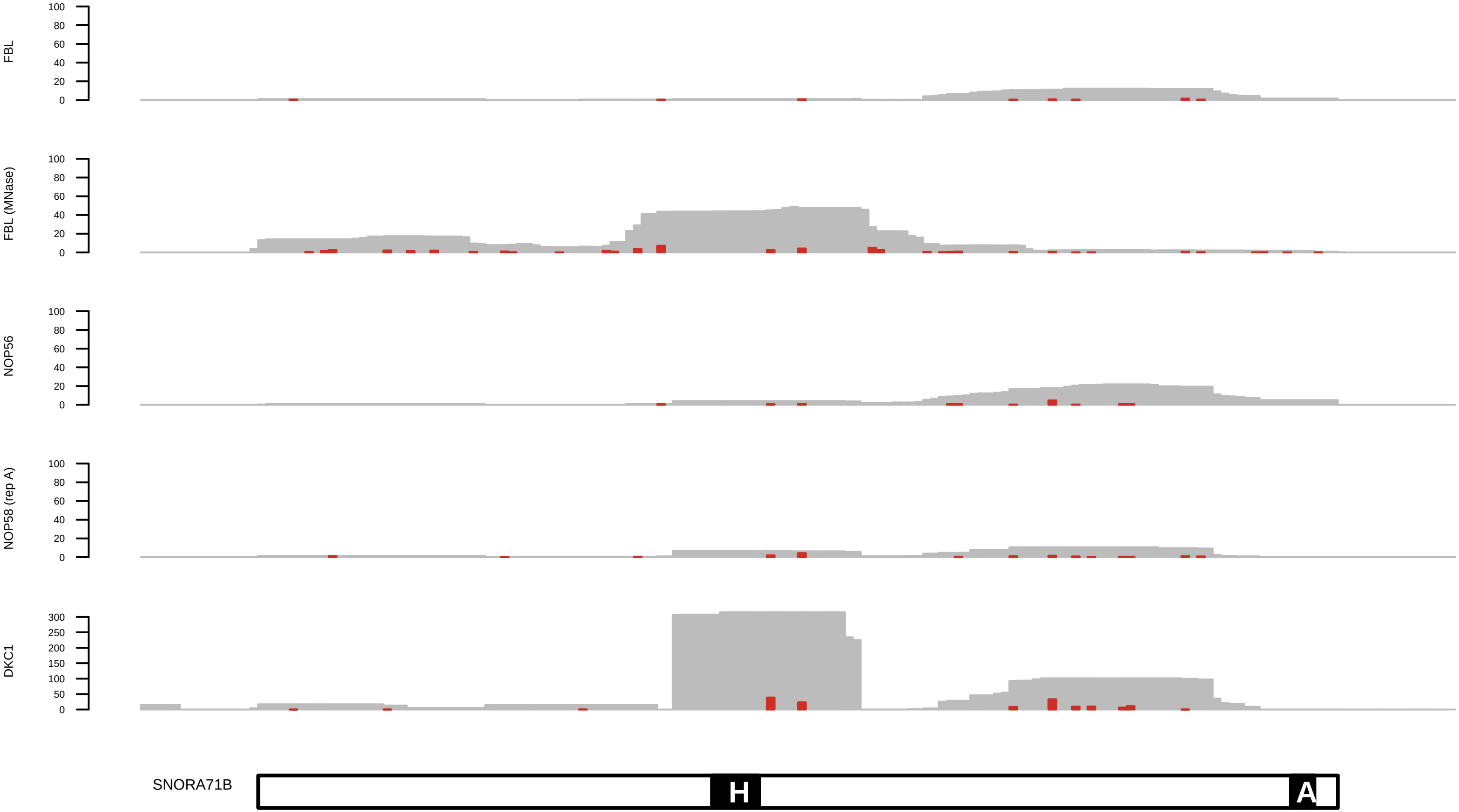
FBL
FBL (MNase)
NOP56
NOP58 (rep A)
DKC1
0
20
40
60
80
100
0
50
100
150
200
250
300
SNORA71B
H
A

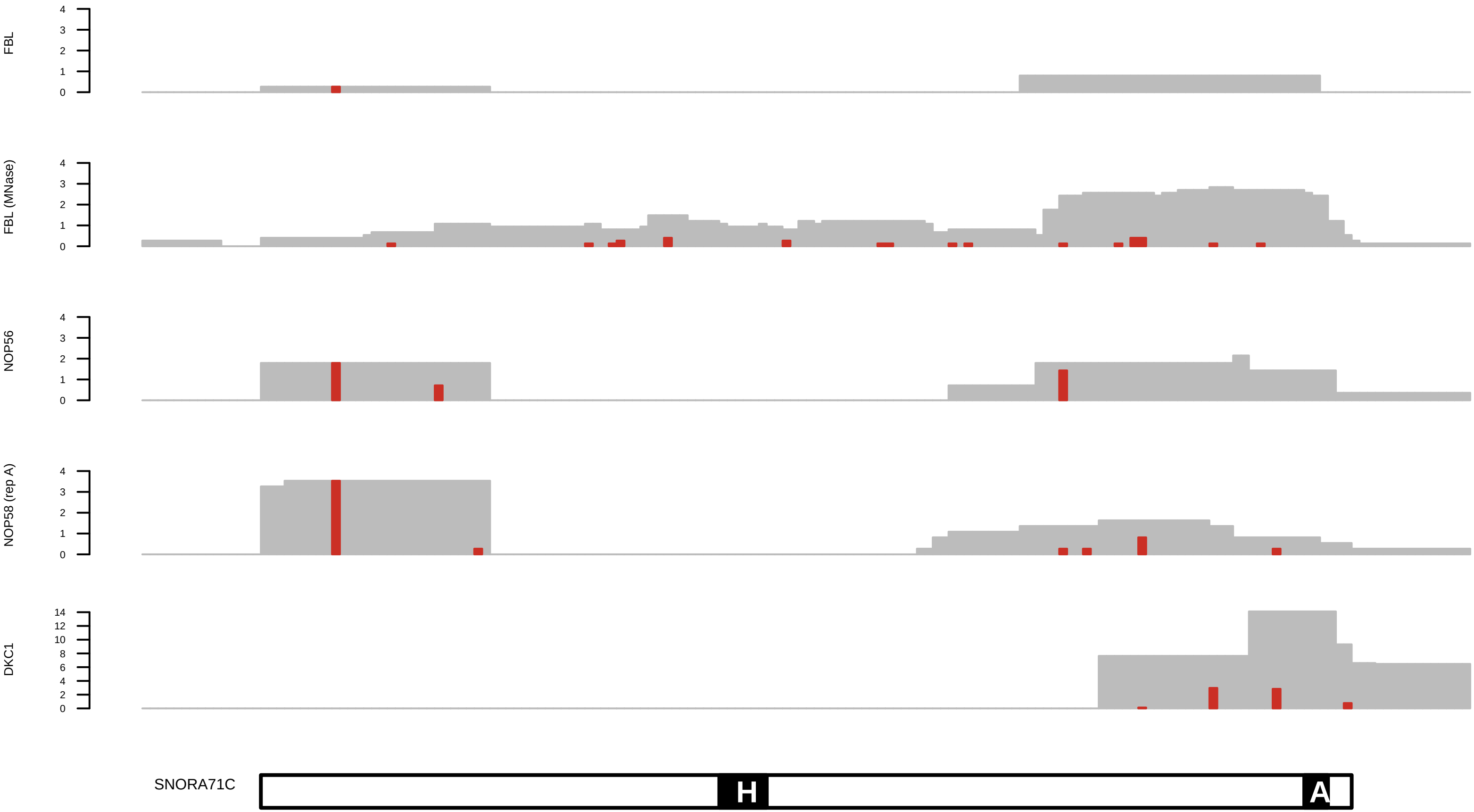
FBL
FBL (MNase)
NOP56
NOP58 (rep A)
DKC1
SNORA71C
H
A

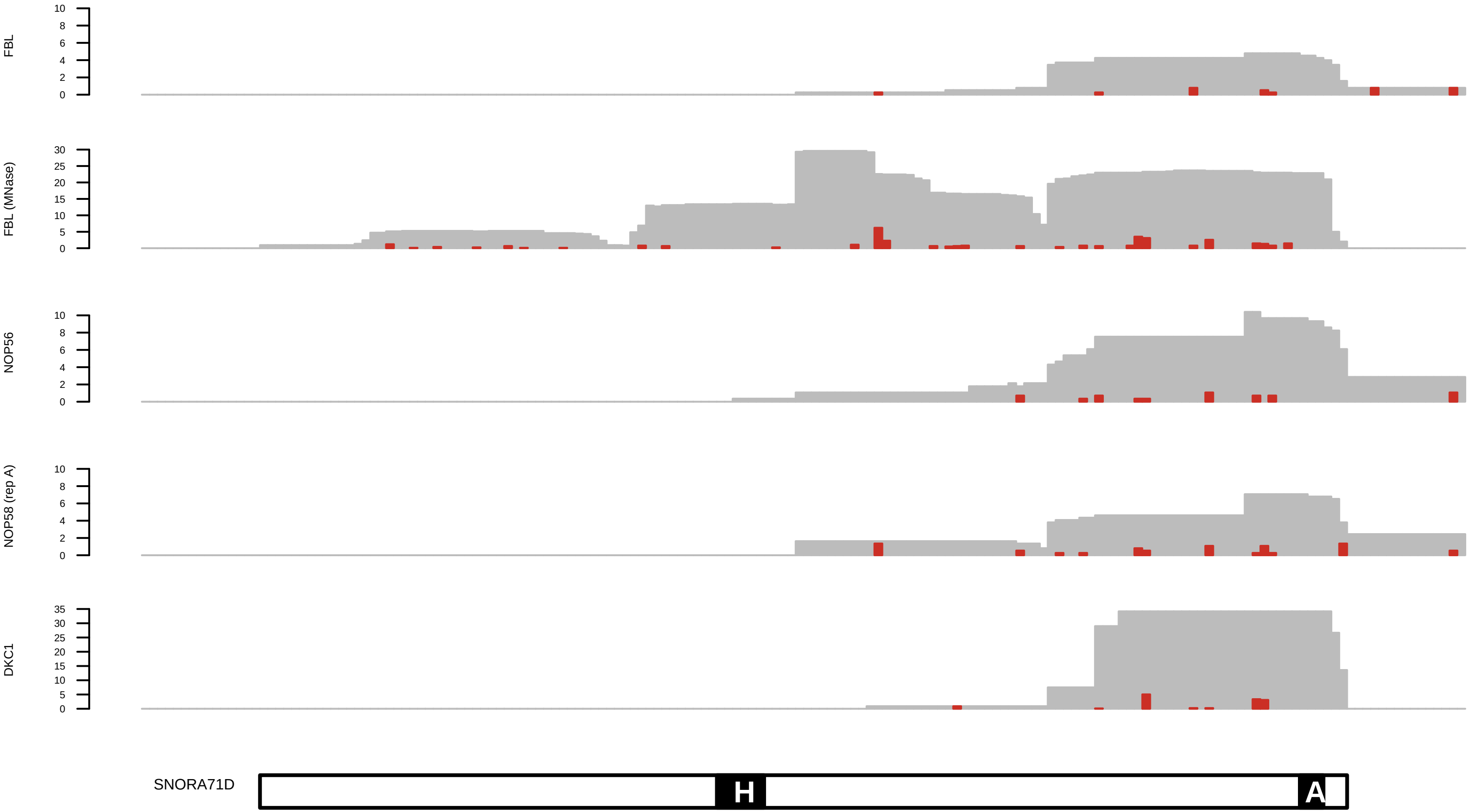

FBL
FBL (MNase)
NOP56
NOP58 (rep A)
DKC1
SNORA71D
H
A

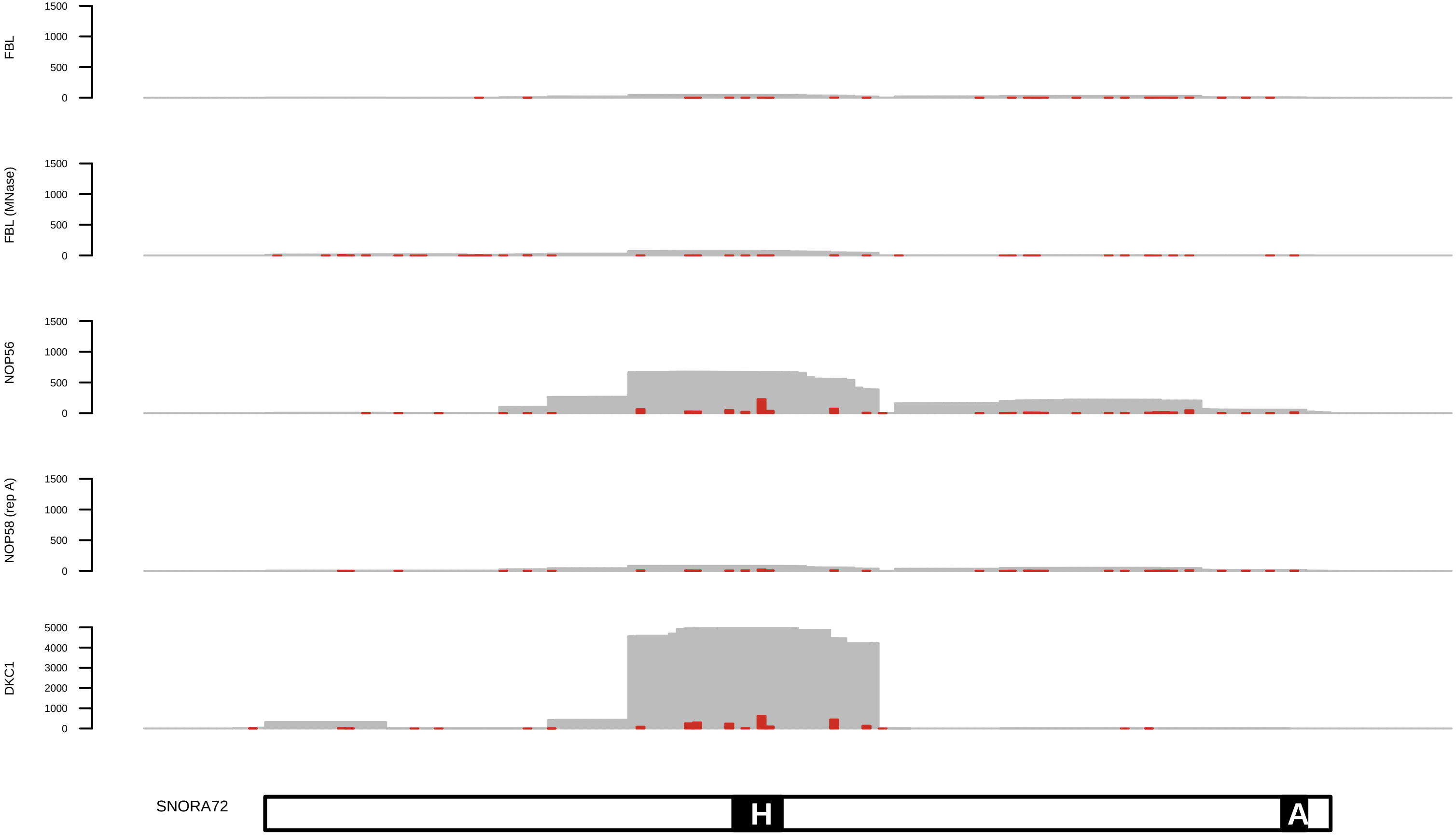
FBL
1500
1000
500
0
FBL (MNase)
NOP56
NOP58 (rep A)
DKC1
5000
4000
3000
2000
1000
SNORA72
H
A

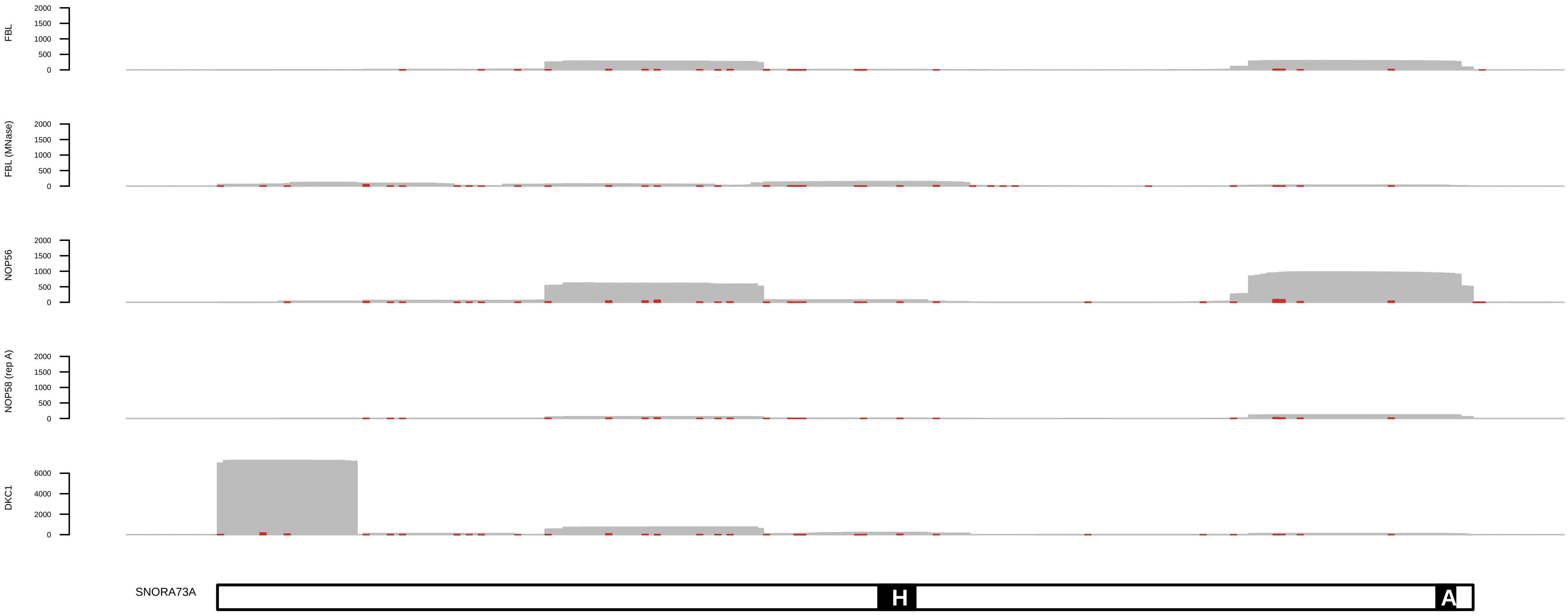
FBL
FBL (MNase)
NOP56
NOP58 (rep A)
DKC1
2000
1500
1000
500
0
6000
4000
2000
SNORA73A
H
A

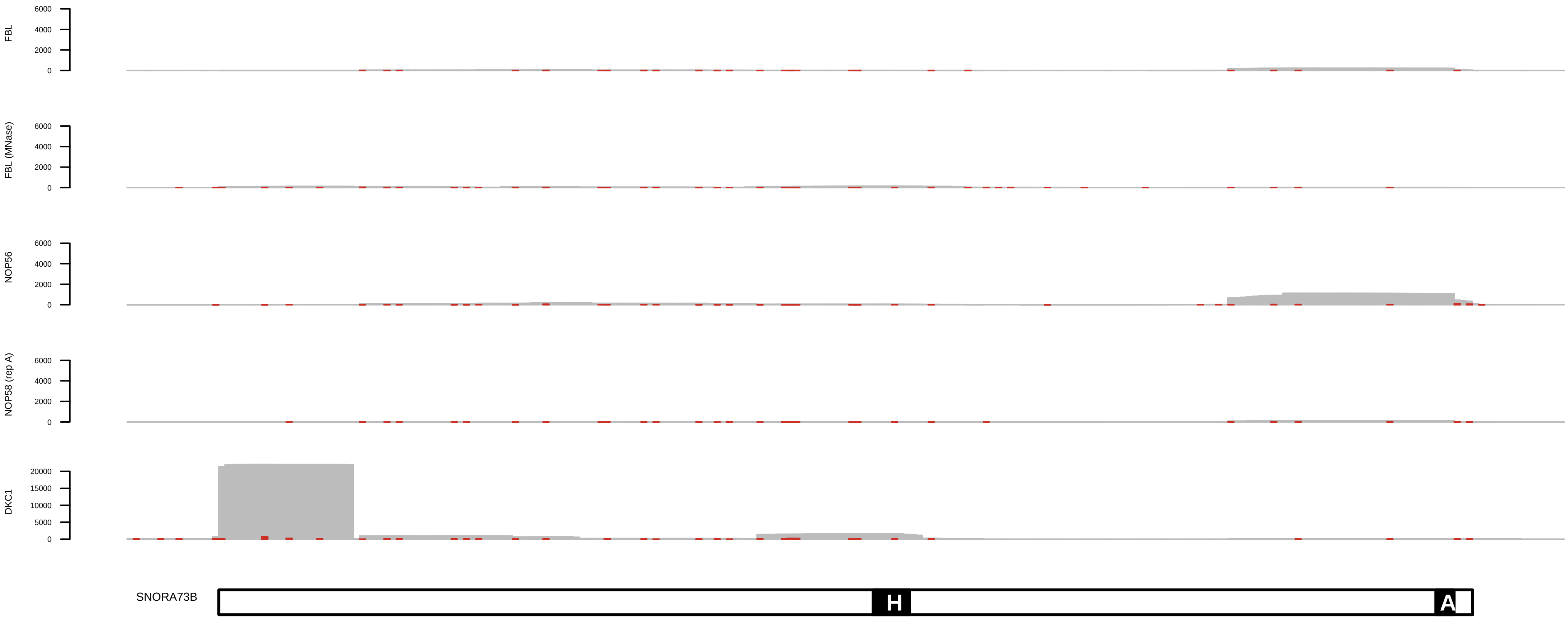
FBL
6000
4000
2000
0
FBL (MNase)
6000
4000
2000
0
NOP56
6000
4000
2000
0
NOP58 (rep A)
6000
4000
2000
0
DKC1
20000
15000
10000
5000
0
SNORA73B
H
A

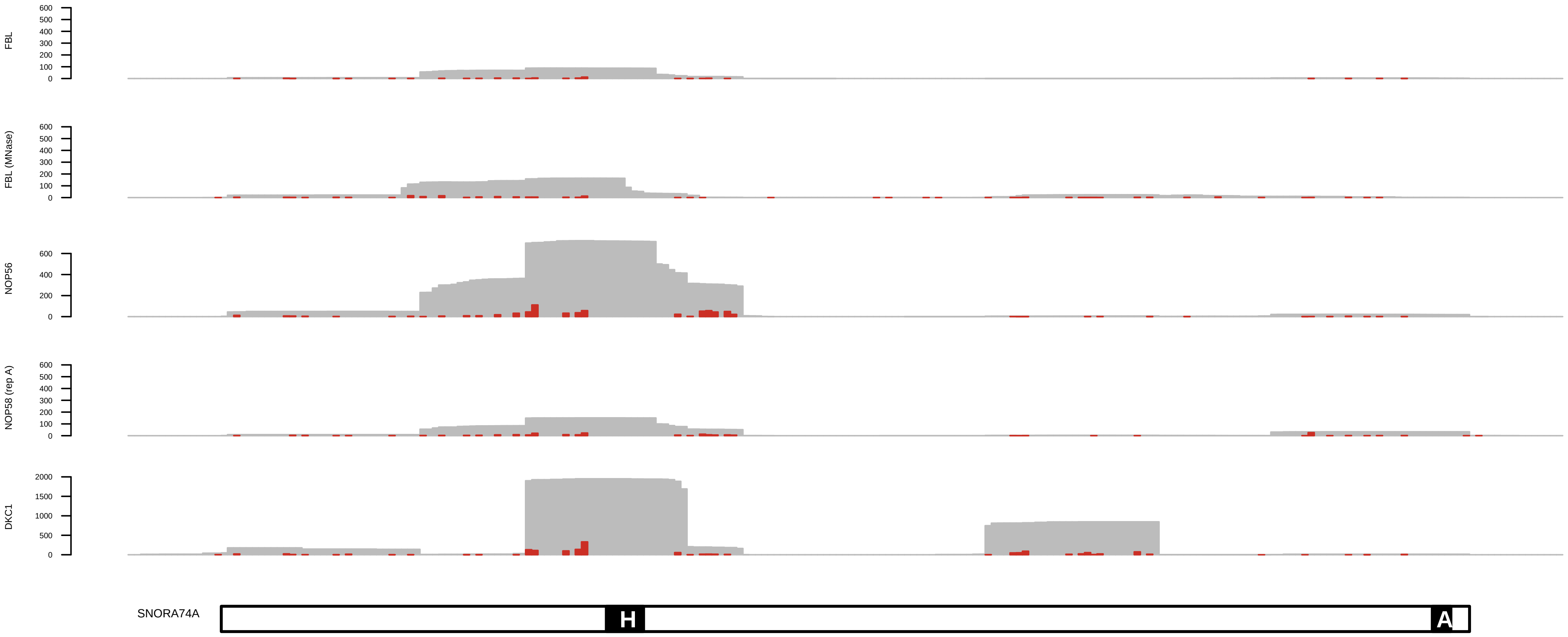

FBL
600
500
400
300
200
100
0
FBL (MNase)
NOP56
600
400
200
0
NOP58 (rep A)
DKC1
2000
1500
1000
500
SNORA74A
H
A

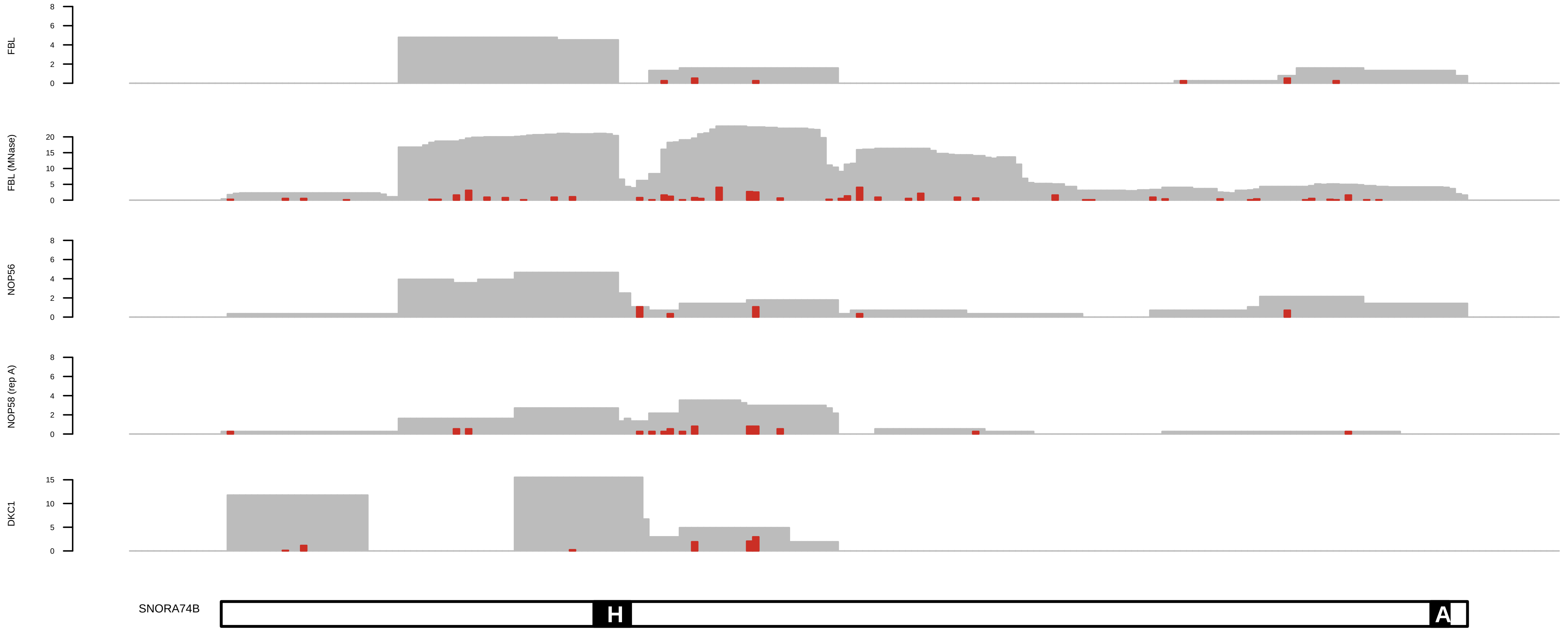

FBL
FBL (MNase)
NOP56
NOP58 (rep A)
DKC1
SNORA74B
H
A

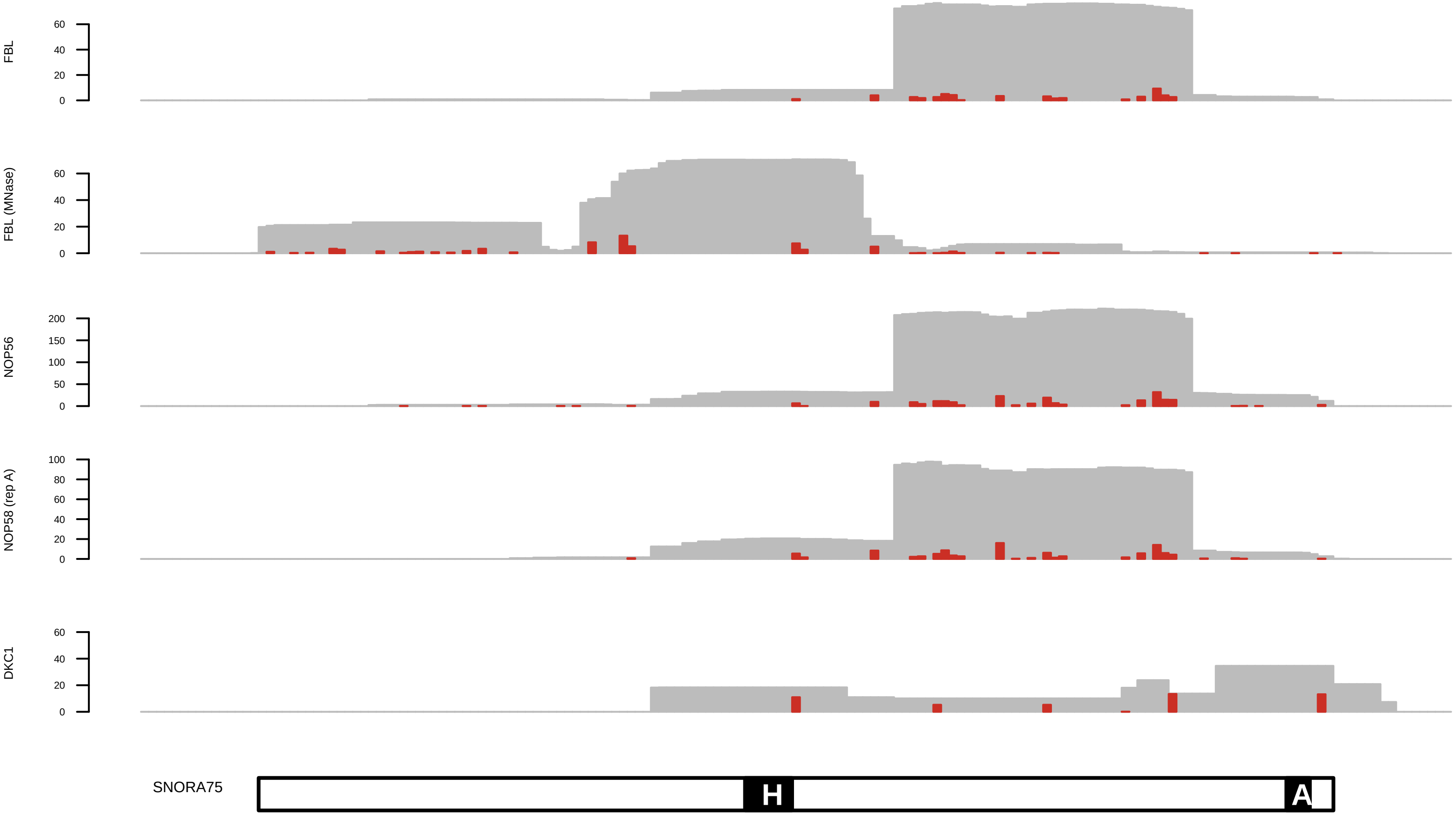

FBL
FBL (MNase)
NOP56
NOP58 (rep A)
DKC1
0
20
40
60
50
100
150
200
80
SNORA75
H
A

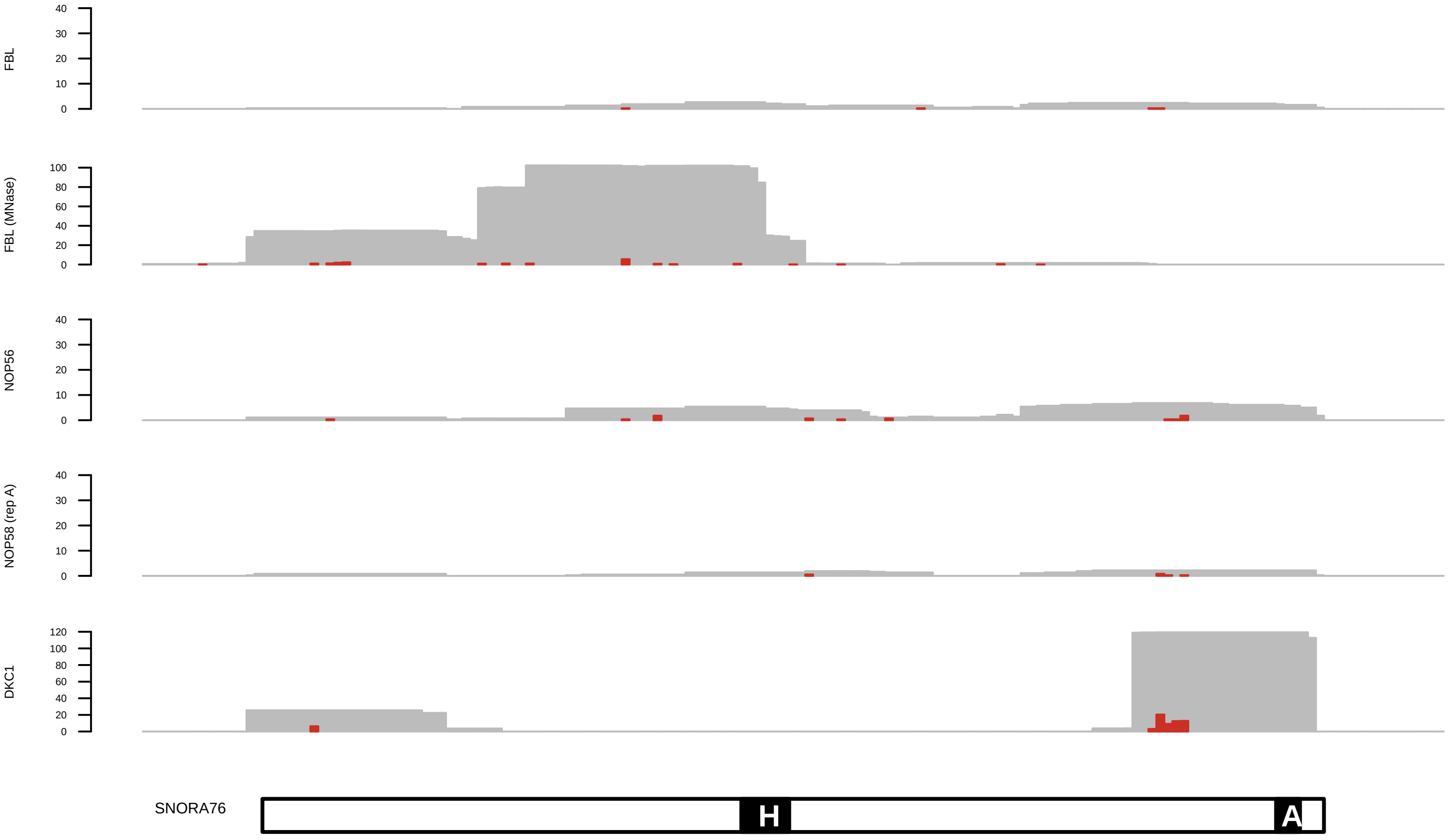
FBL
FBL (MNase)
NOP56
NOP58 (rep A)
DKC1
SNORA76
H
A

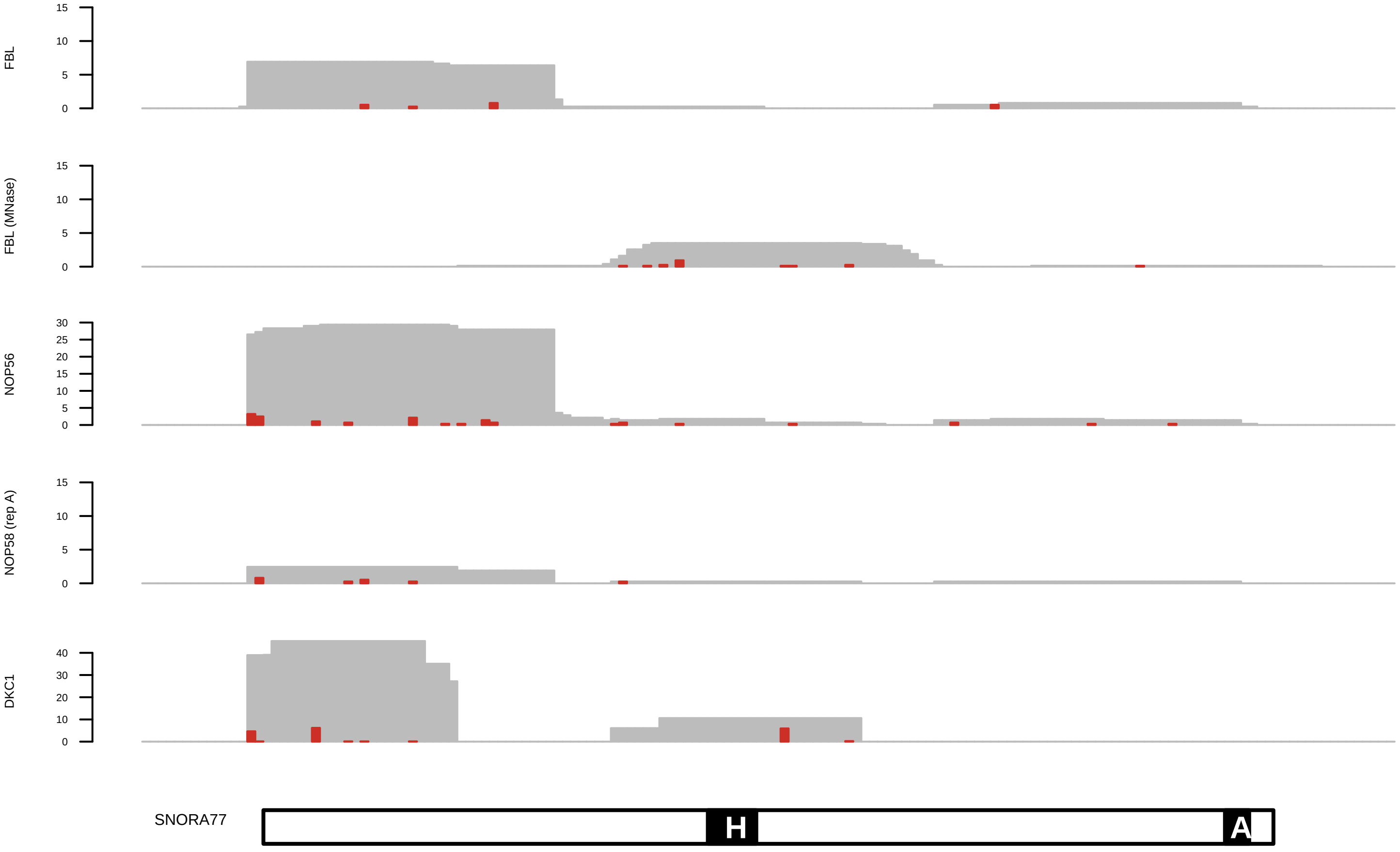

FBL
FBL (MNase)
NOP56
NOP58 (rep A)
DKC1
SNORA77
H
A

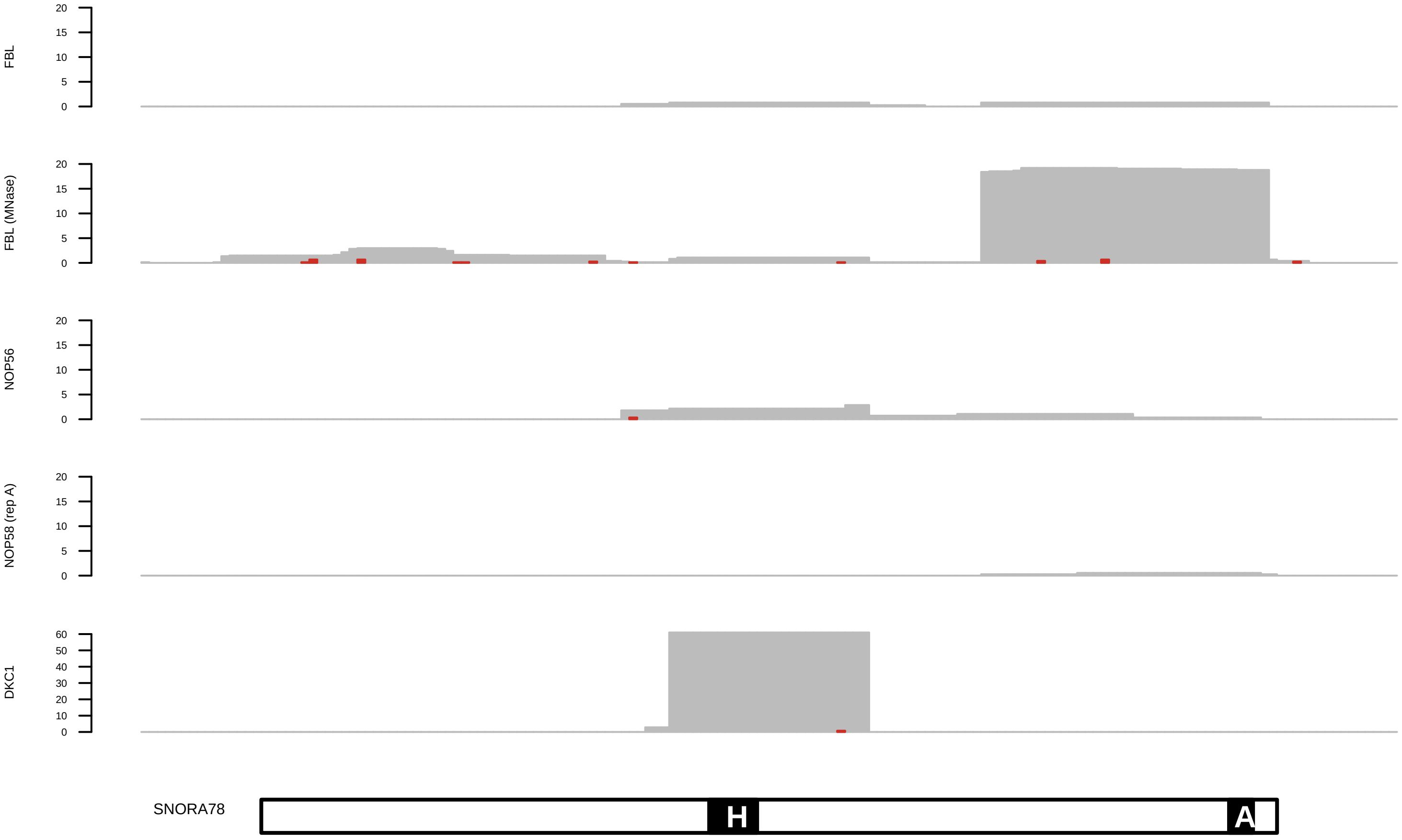

FBL
FBL (MNase)
NOP56
NOP58 (rep A)
DKC1
20
15
10
5
0
60
50
40
30
20
10
0
SNORA78
H
A

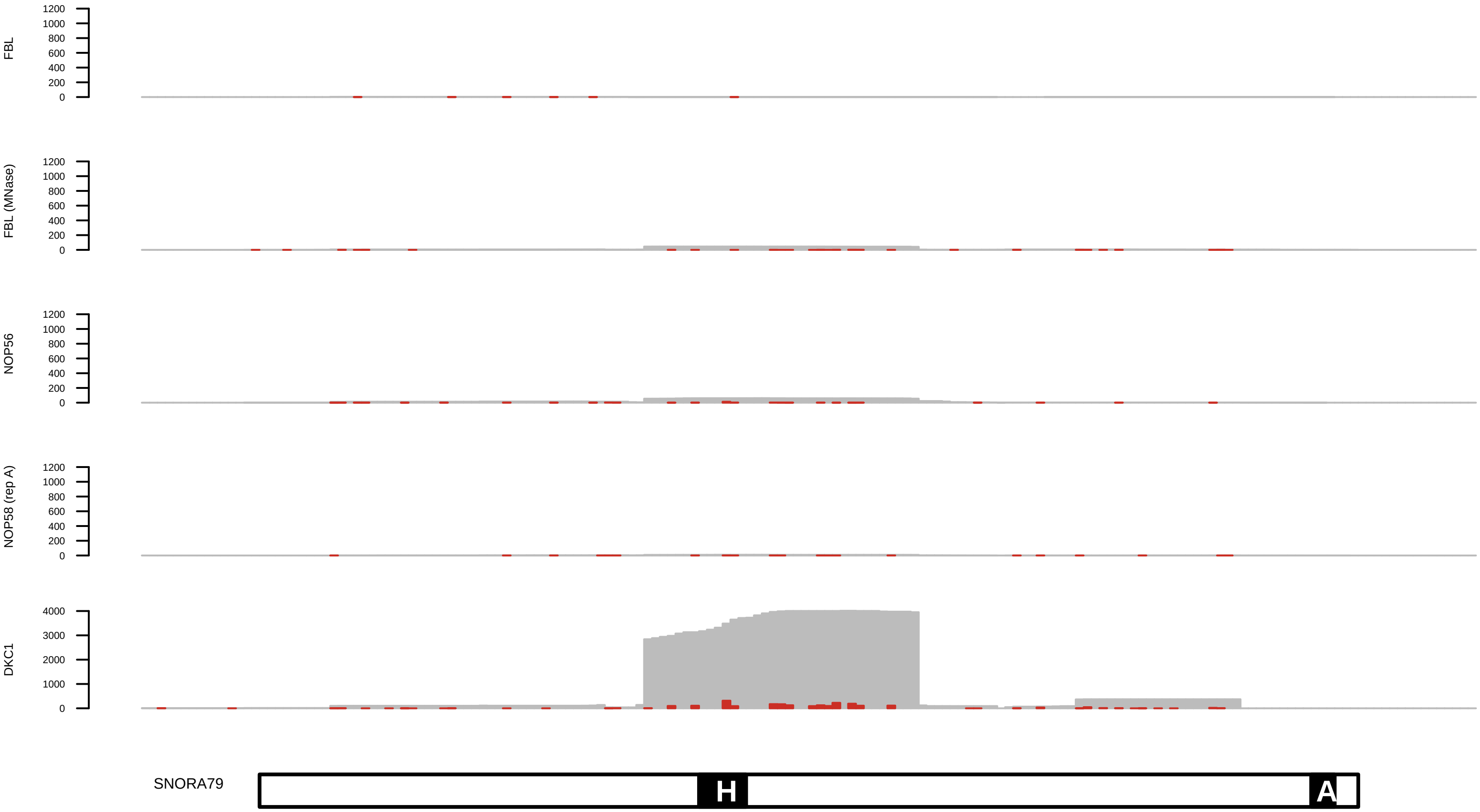
FBL
FBL (MNase)
NOP56
NOP58 (rep A)
DKC1
SNORA79
H
A

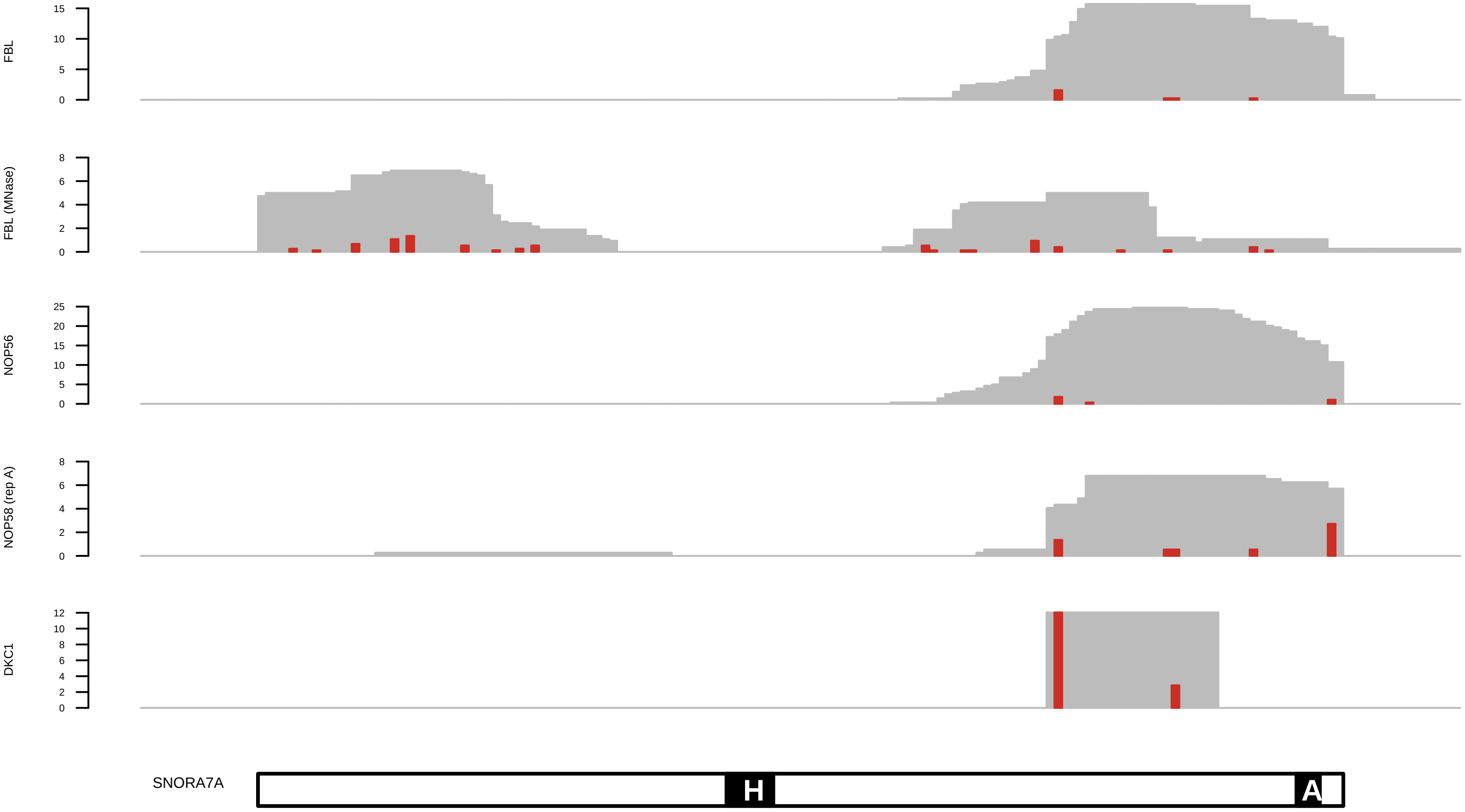
FBL
15
10
5
0
FBL (MNase)
8
6
4
2
0
NOP56
25
20
15
10
5
0
NOP58 (rep A)
8
6
4
2
0
DKC1
12
10
8
6
4
2
0
SNORA7A
H
A

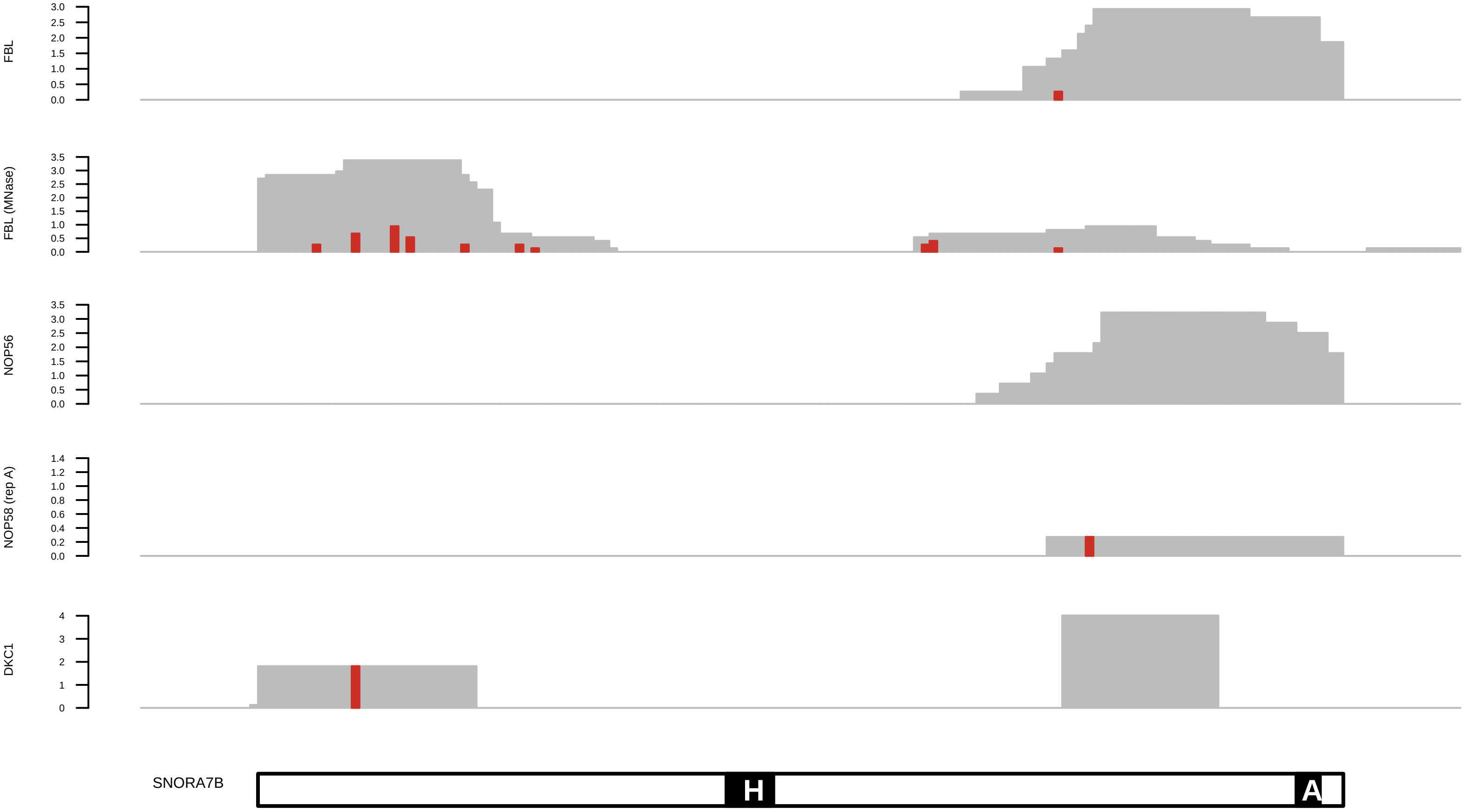
FBL
FBL (MNase)
NOP56
NOP58 (rep A)
DKC1
SNORA7B
H
A

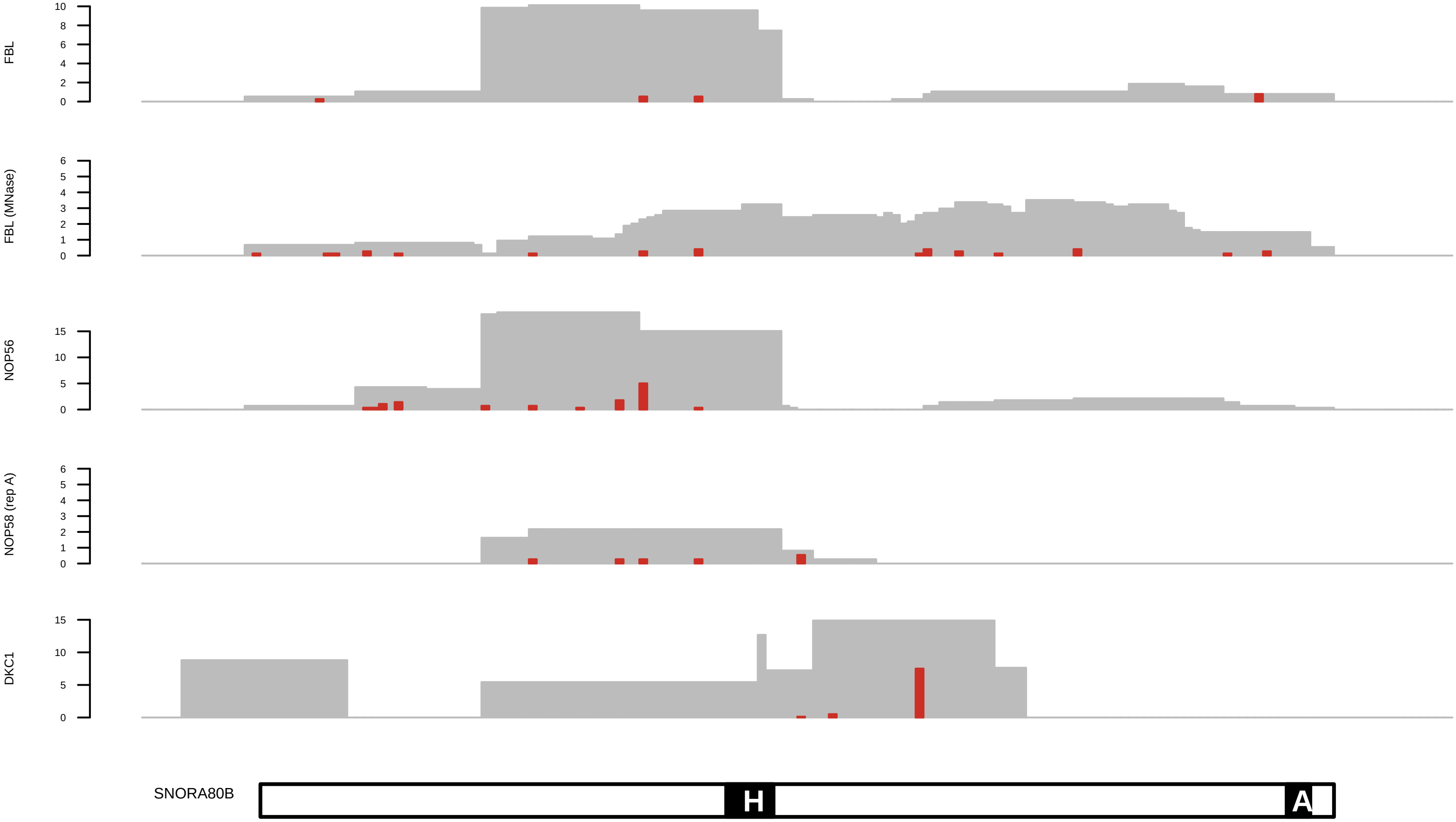
FBL
FBL (MNase)
NOP56
NOP58 (rep A)
DKC1
SNORA80B
H
A

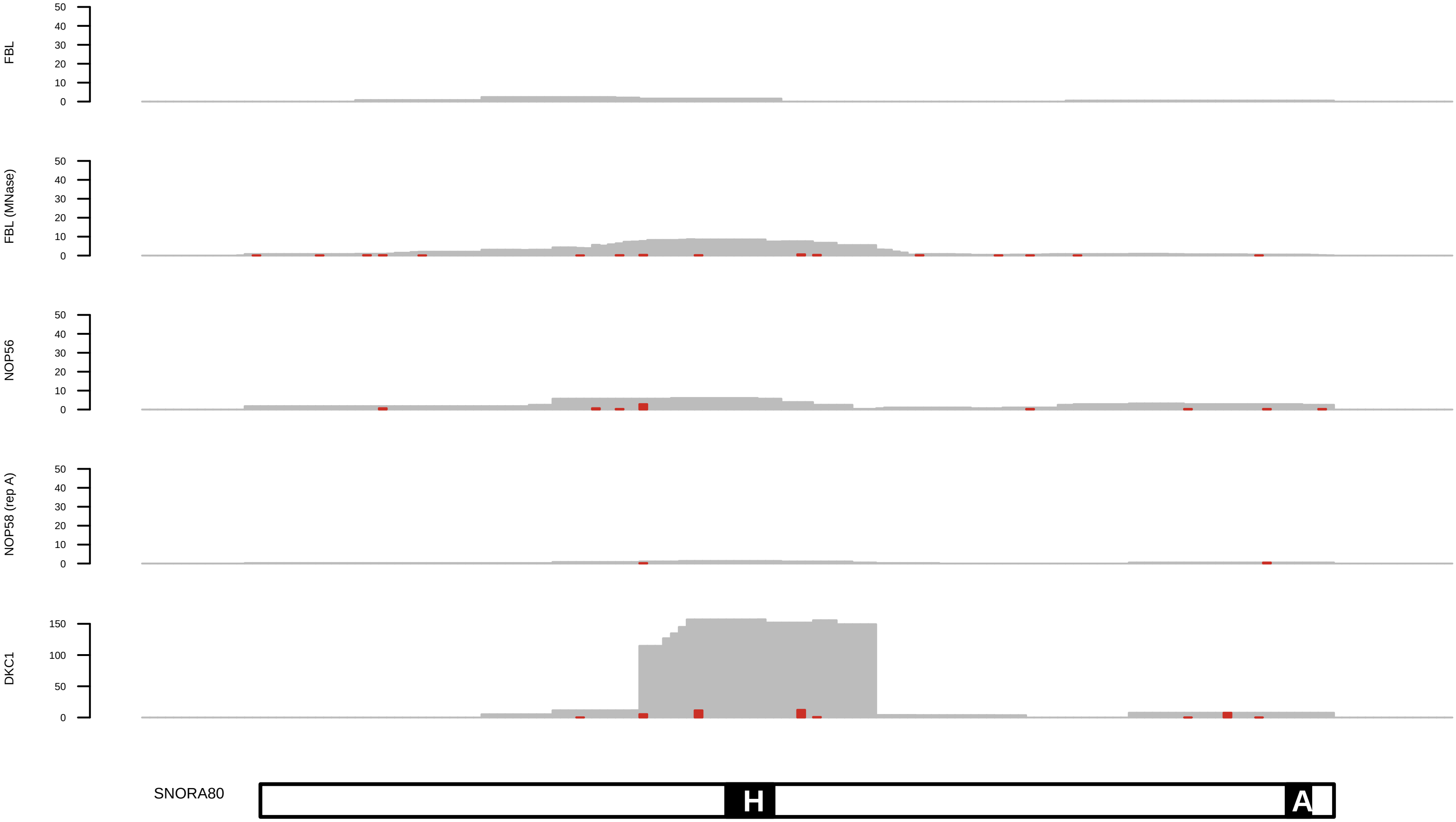
FBL
FBL (MNase)
NOP56
NOP58 (rep A)
DKC1
0
10
20
30
40
50
100
150
SNORA80
H
A

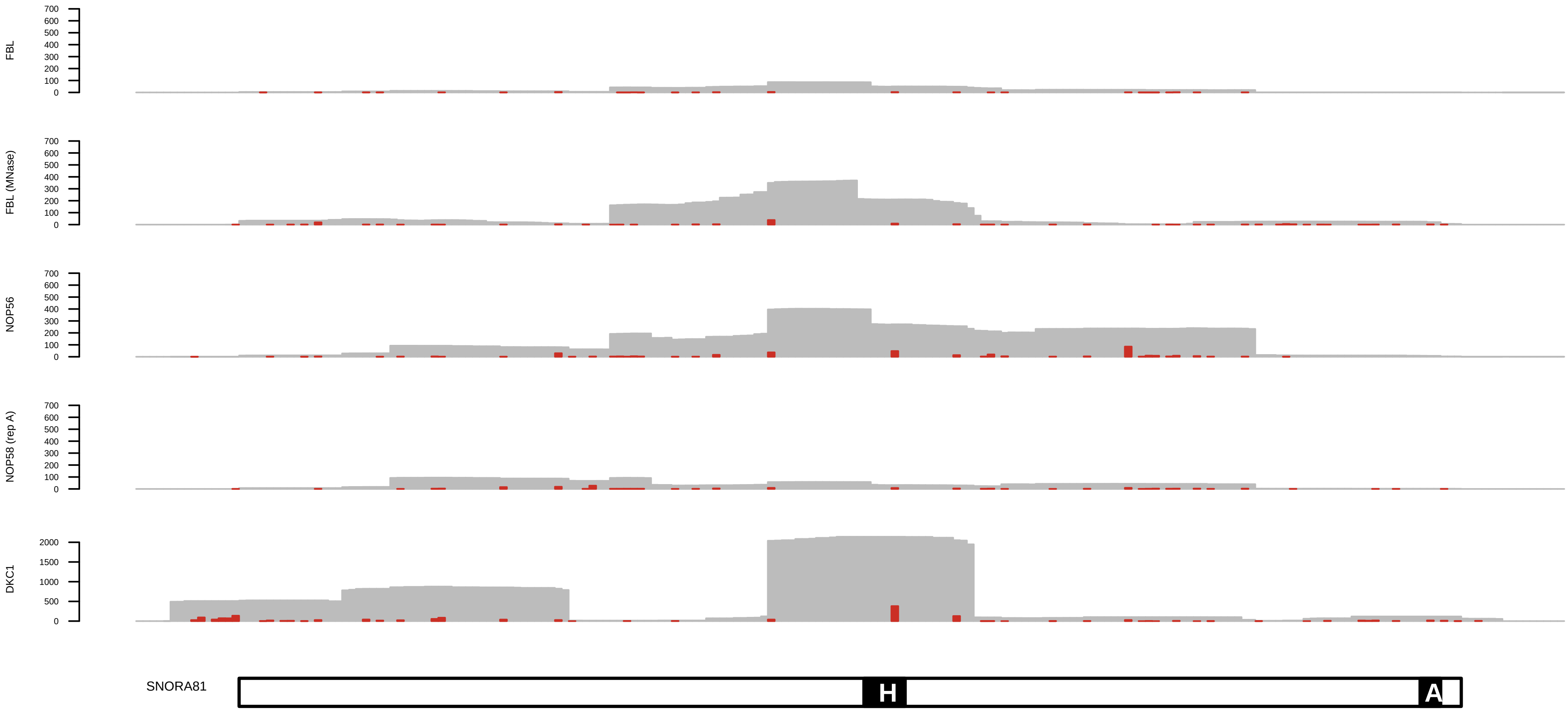
FBL
FBL (MNase)
NOP56
NOP58 (rep A)
DKC1
SNORA81
H
A

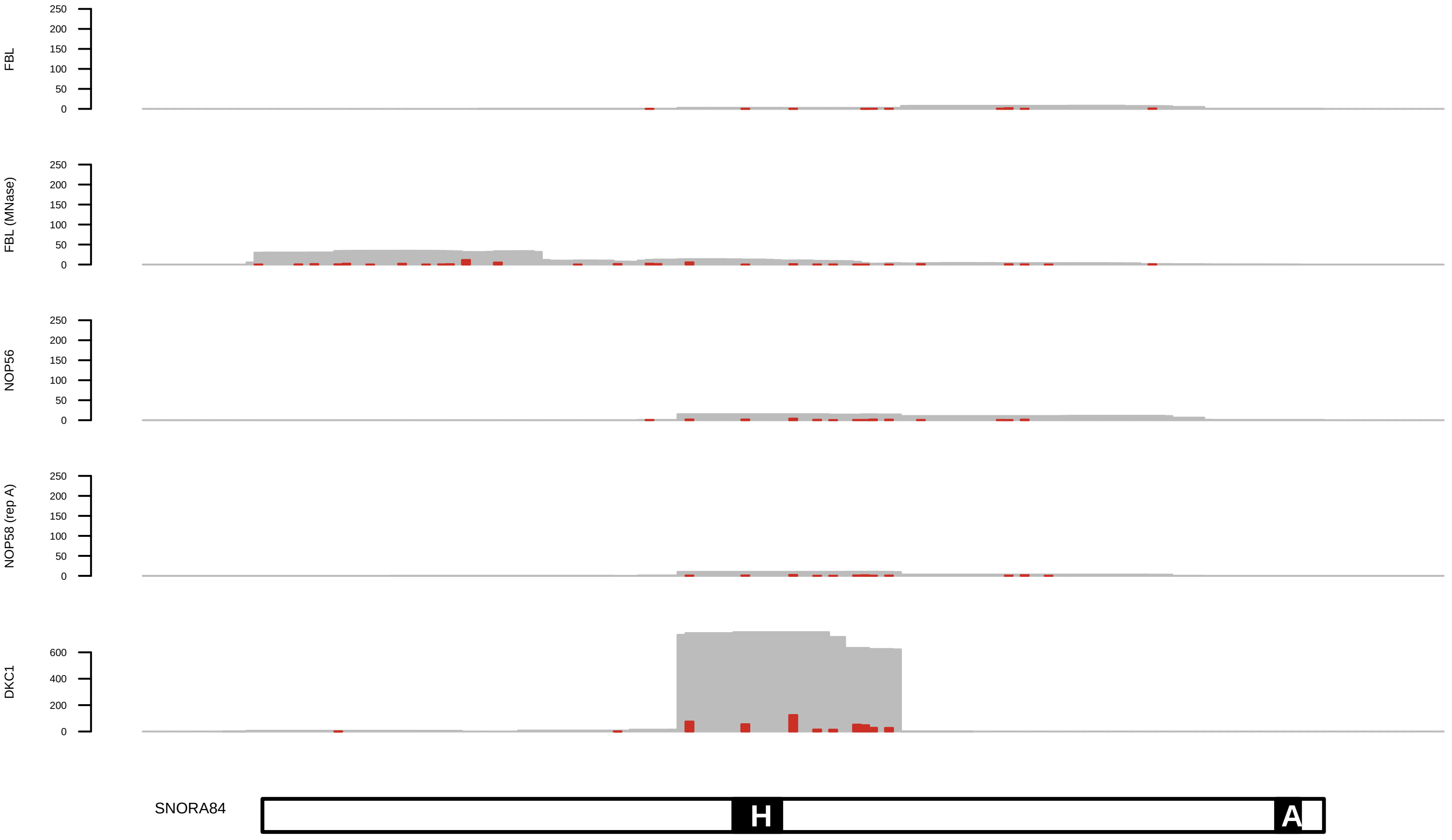
FBL
FBL (MNase)
NOP56
NOP58 (rep A)
DKC1
250
200
150
100
50
0
600
400
200
SNORA84
H
A

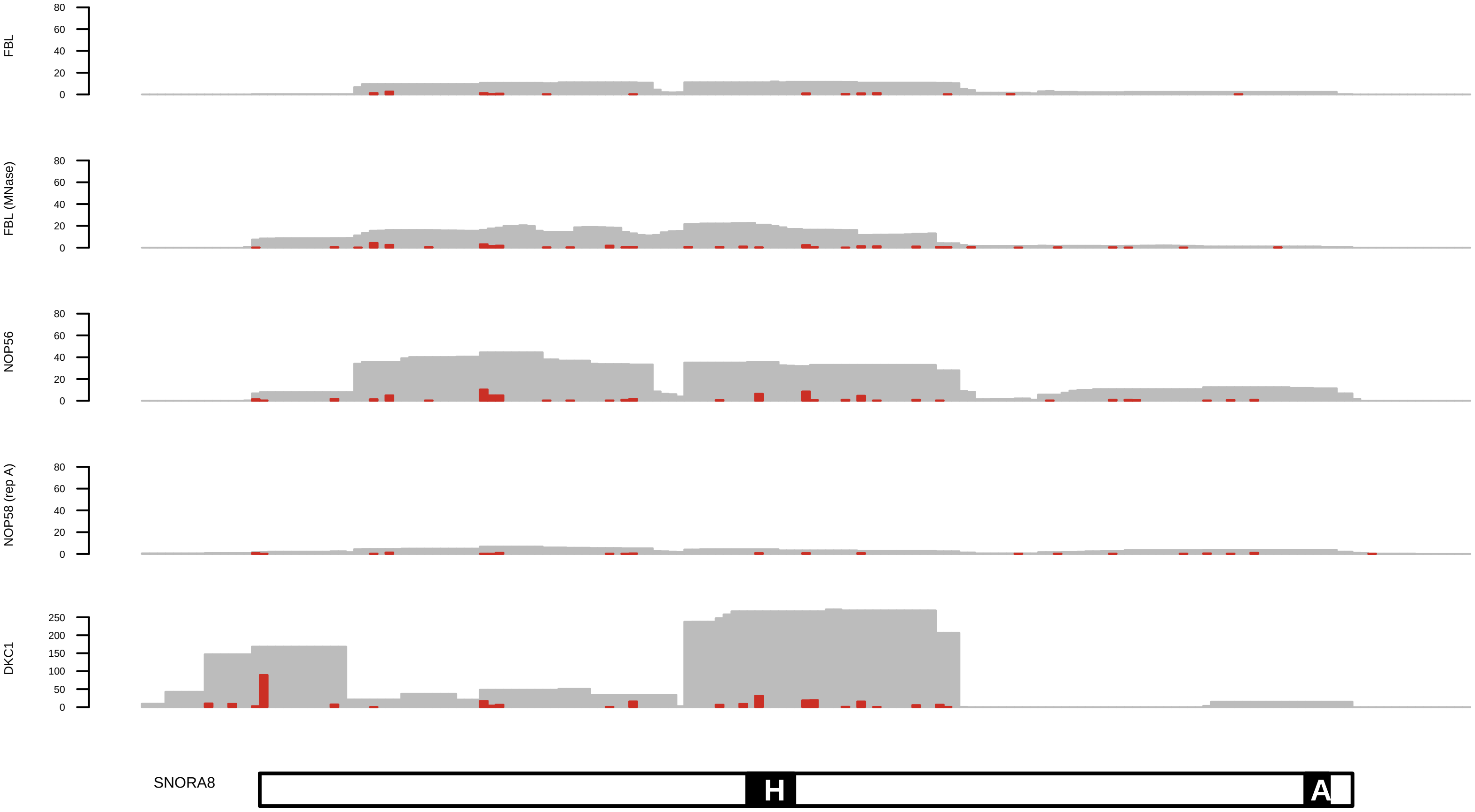
FBL
80
60
40
20
0
FBL (MNase)
80
60
40
20
0
NOP56
80
60
40
20
0
NOP58 (rep A)
80
60
40
20
0
DKC1
250
200
150
100
50
0
SNORA8
H
A

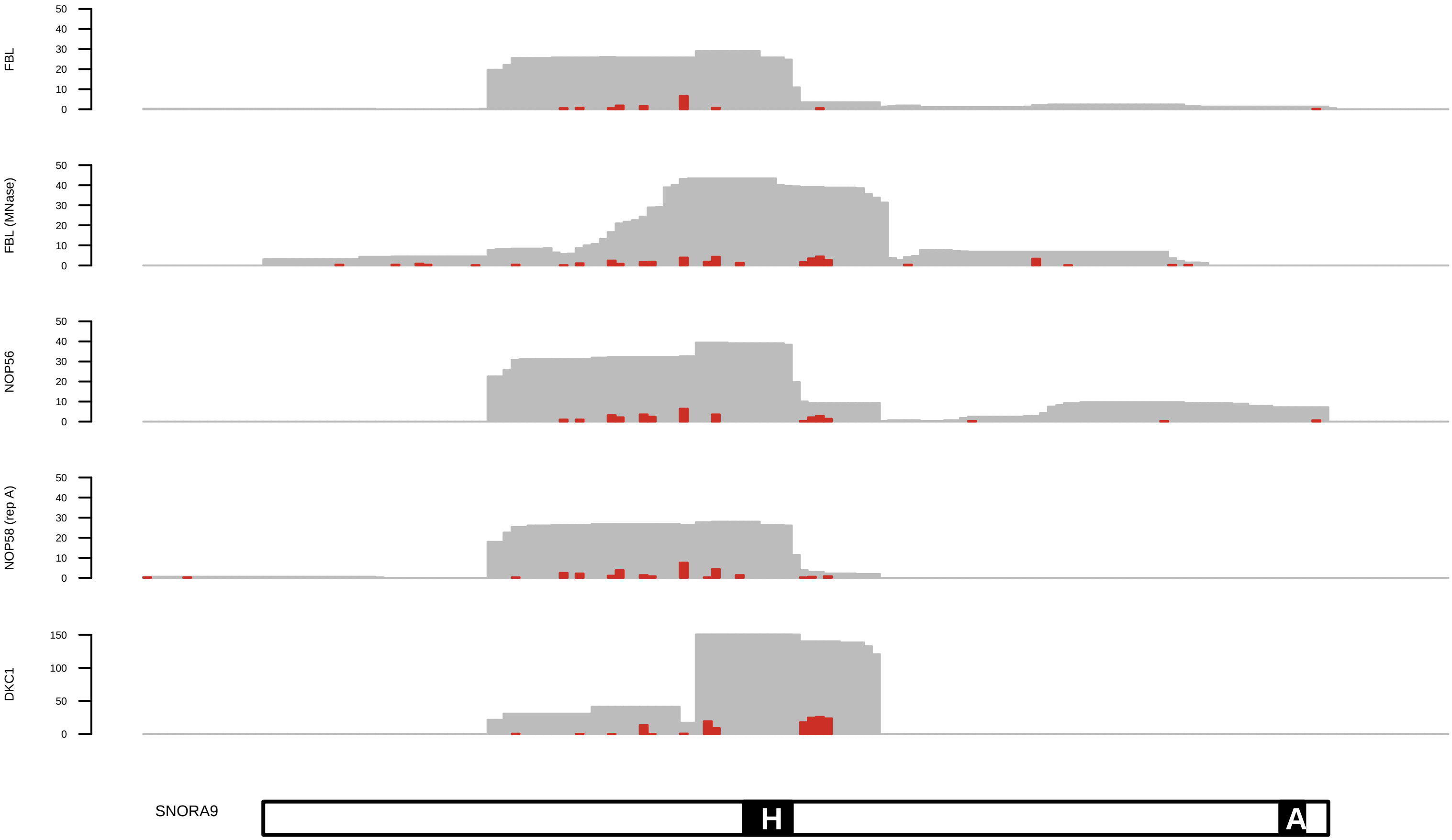
FBL
FBL (MNase)
NOP56
NOP58 (rep A)
DKC1
0
10
20
30
40
50
100
150
SNORA9
H
A

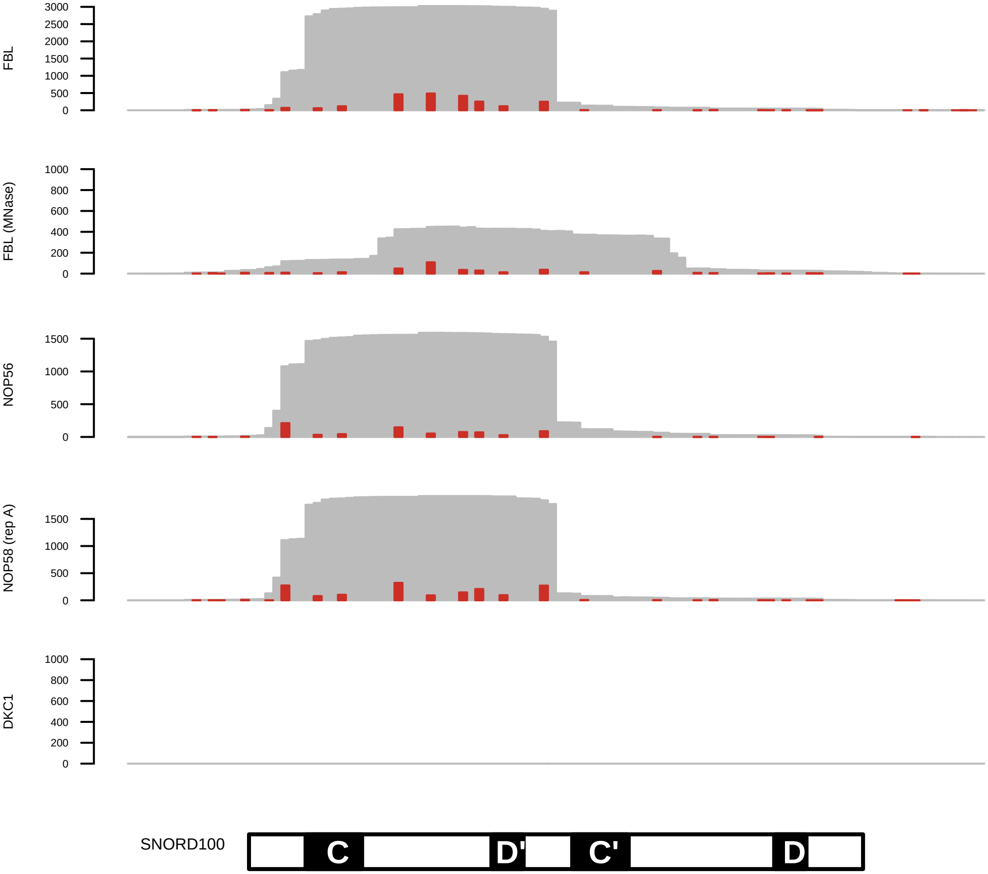
FBL
3000
2500
2000
1500
1000
500
0
FBL (MNase)
1000
800
600
400
200
0
NOP56
1500
1000
500
0
NOP58 (rep A)
1500
1000
500
0
DKC1
1000
800
600
400
200
0
SNORD100
C
D'
C'
D

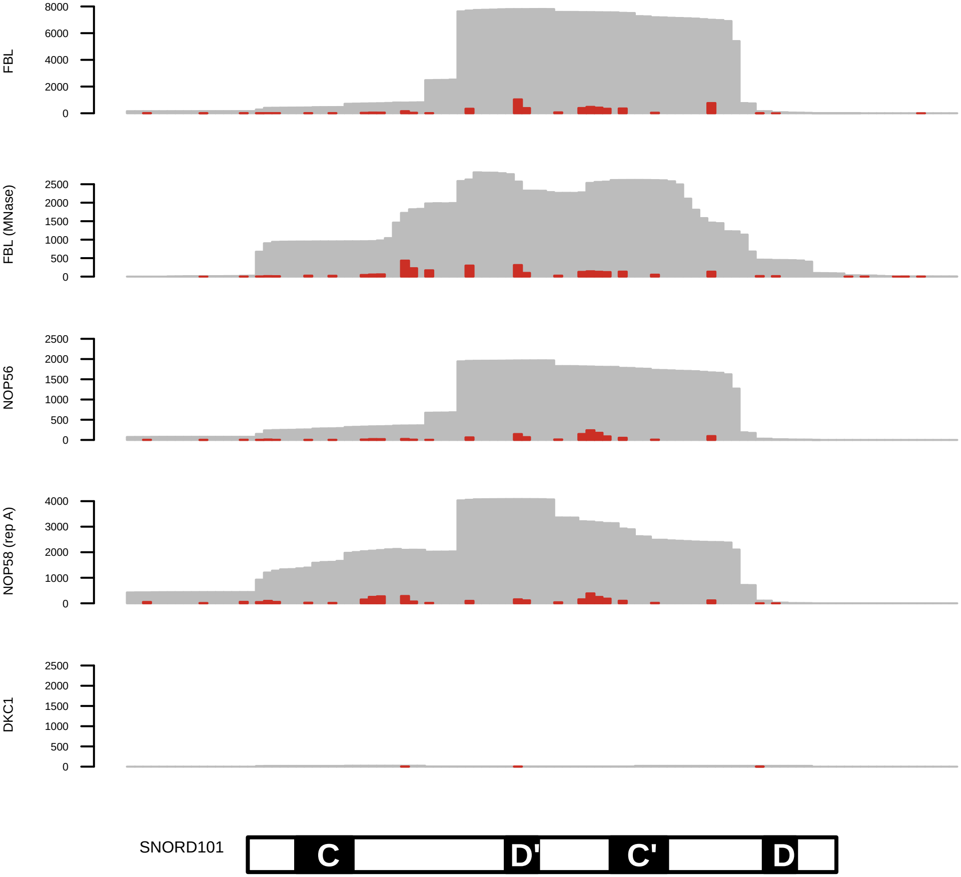
FBL
8000
6000
4000
2000
0
FBL (MNase)
2500
2000
1500
1000
500
0
NOP56
2500
2000
1500
1000
500
0
NOP58 (rep A)
4000
3000
2000
1000
0
DKC1
2500
2000
1500
1000
500
0
SNORD101
C
D'
C'
D

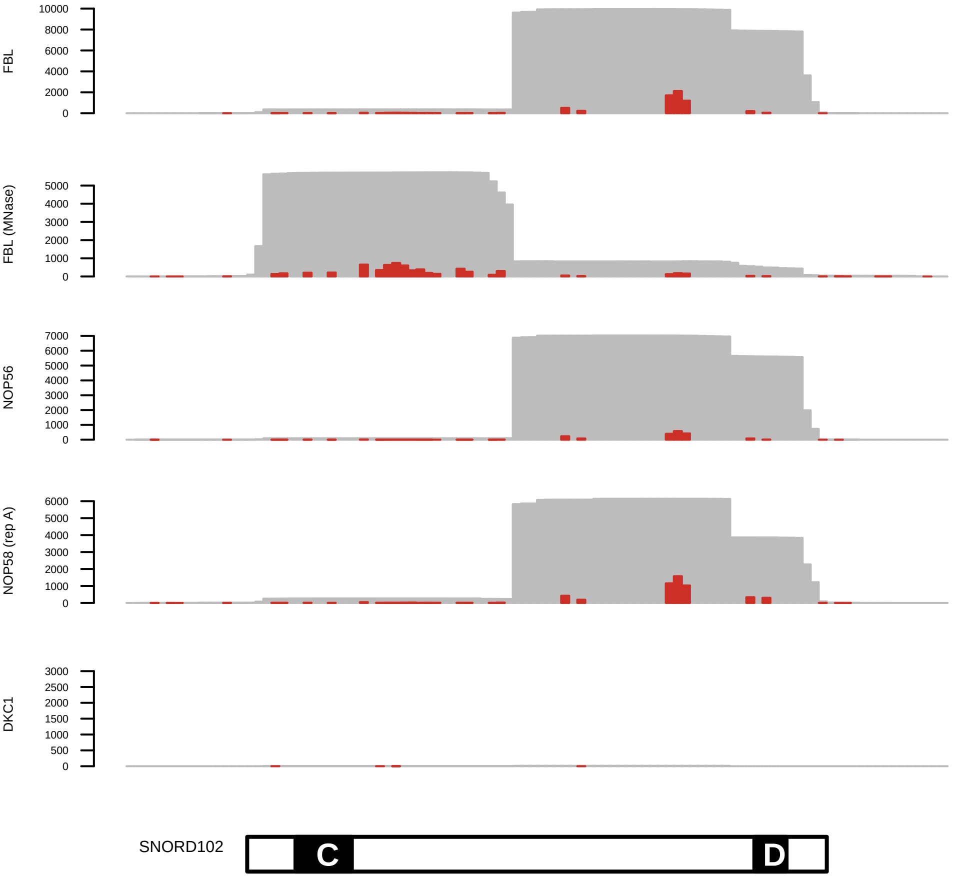

FBL
10000
8000
6000
4000
2000
0
FBL (MNase)
5000
4000
3000
2000
1000
0
NOP56
7000
6000
5000
4000
3000
2000
1000
0
NOP58 (rep A)
6000
5000
4000
3000
2000
1000
0
DKC1
3000
2500
2000
1500
1000
500
0
SNORD102
C
D

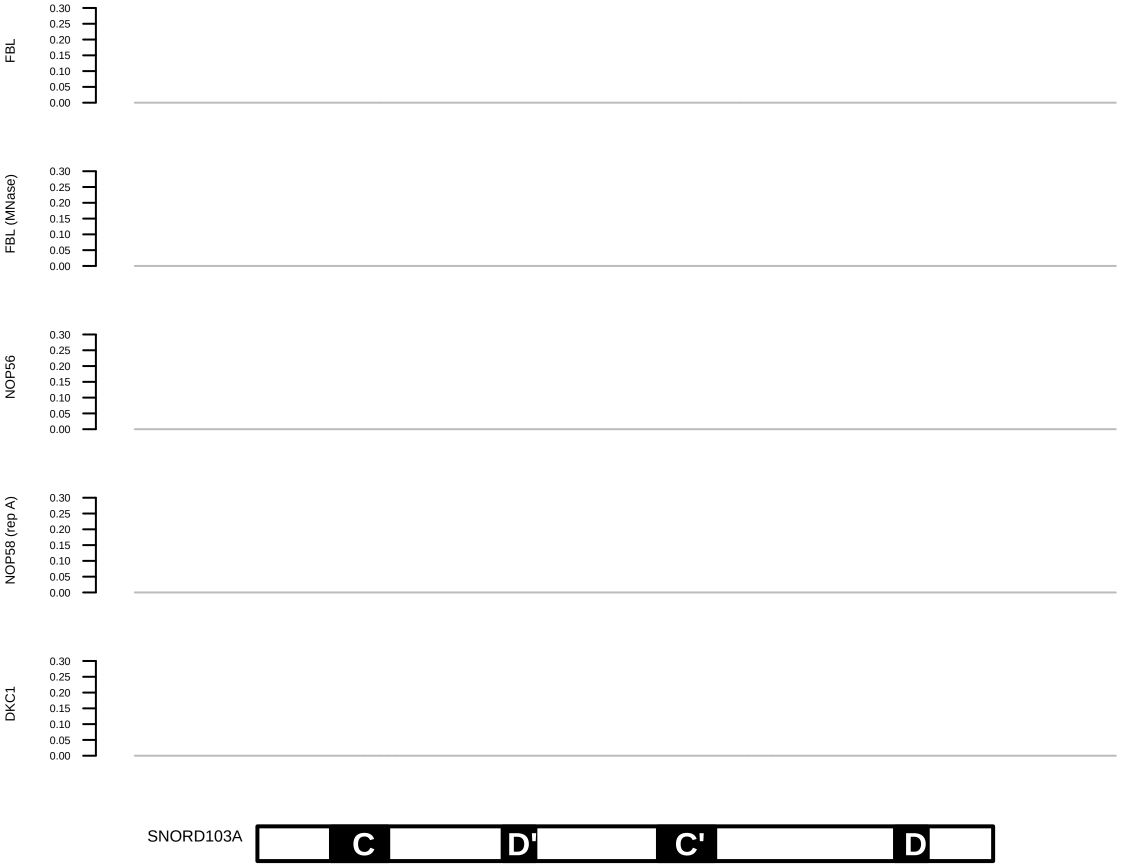
FBL
FBL (MNase)
NOP56
NOP58 (rep A)
DKC1
0.30
0.25
0.20
0.15
0.10
0.05
0.00
SNORD103A
C
D'
C'
D

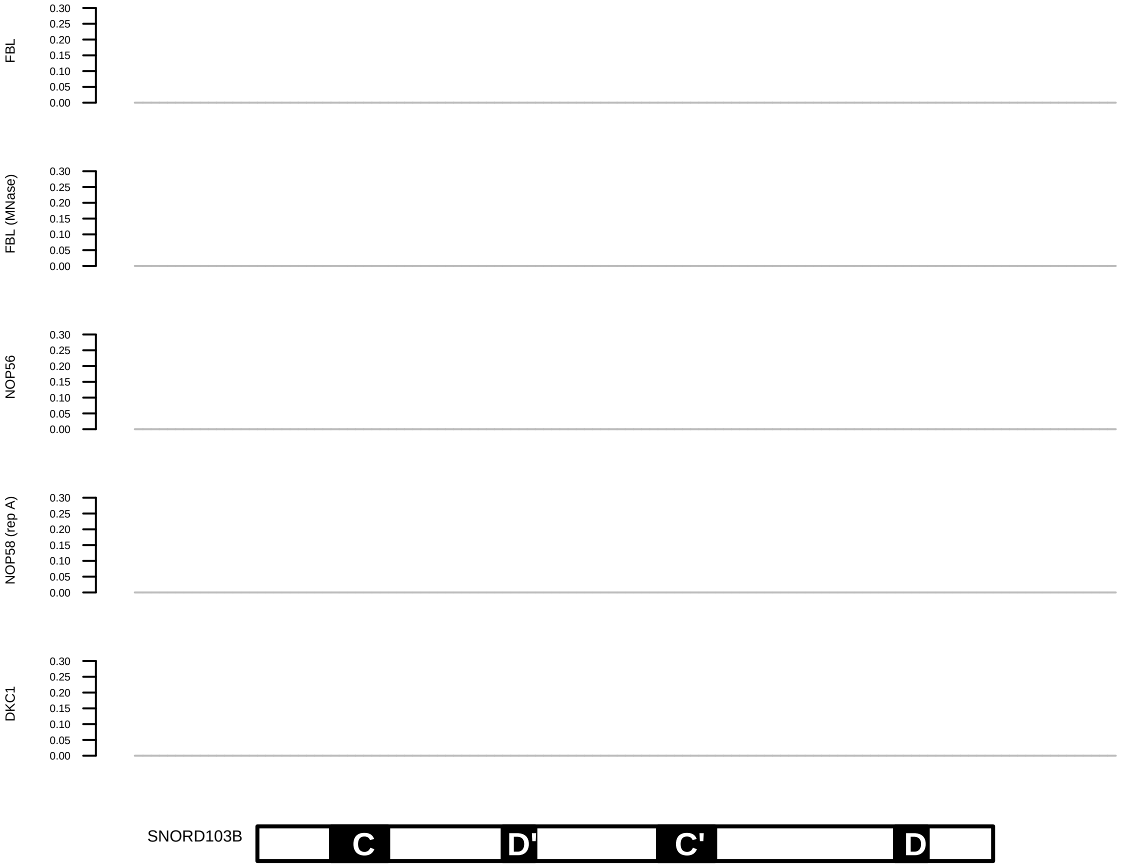
FBL
FBL (MNase)
NOP56
NOP58 (rep A)
DKC1
0.30
0.25
0.20
0.15
0.10
0.05
0.00
SNORD103B
C
D'
C'
D

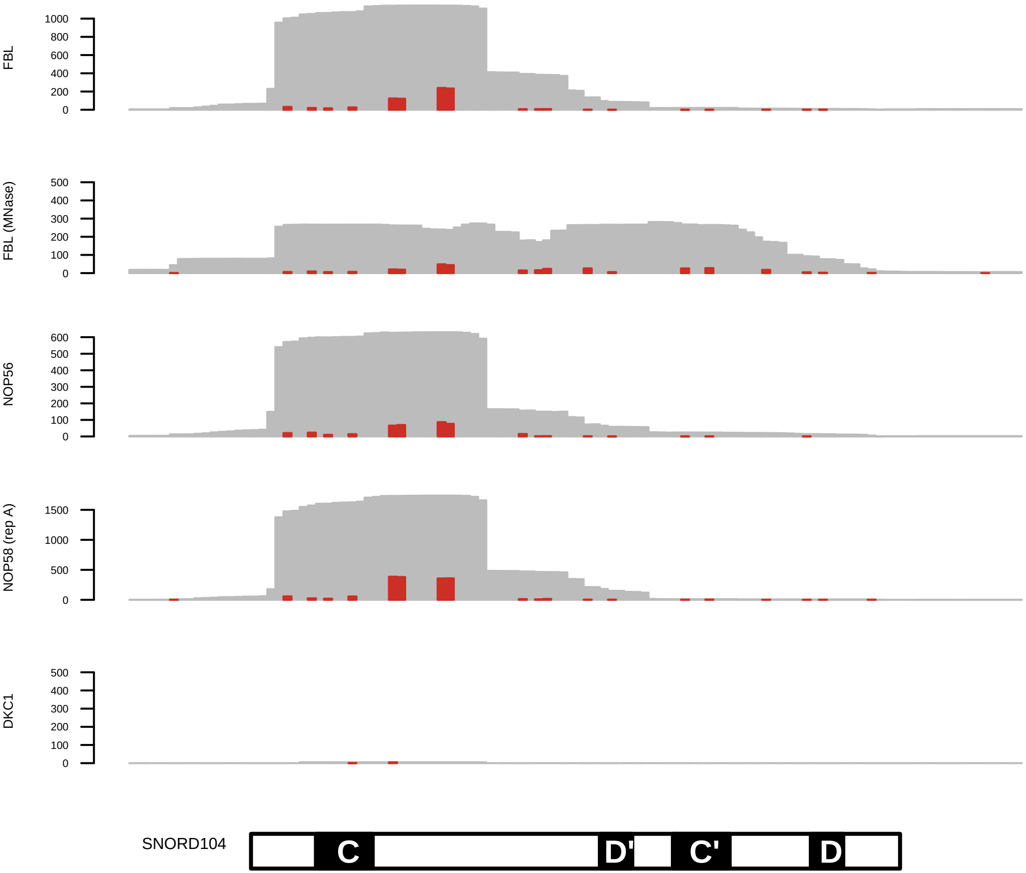
FBL
1000
800
600
400
200
0
FBL (MNase)
500
400
300
200
100
0
NOP56
600
500
400
300
200
100
0
NOP58 (rep A)
1500
1000
500
0
DKC1
500
400
300
200
100
0
SNORD104
C
D'
C'
D

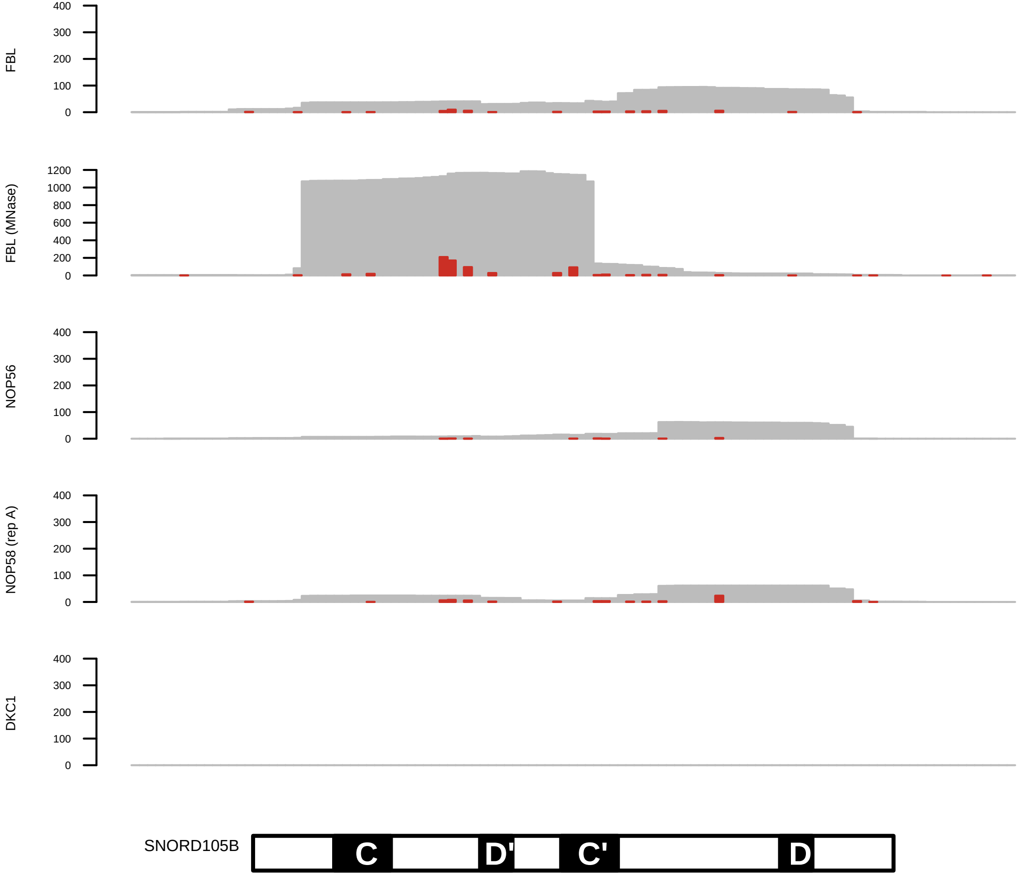

FBL
400
300
200
100
0
FBL (MNase)
1200
1000
800
600
400
200
0
NOP56
400
300
200
100
0
NOP58 (rep A)
400
300
200
100
0
DKC1
400
300
200
100
0
SNORD105B
C
D'
C'
D

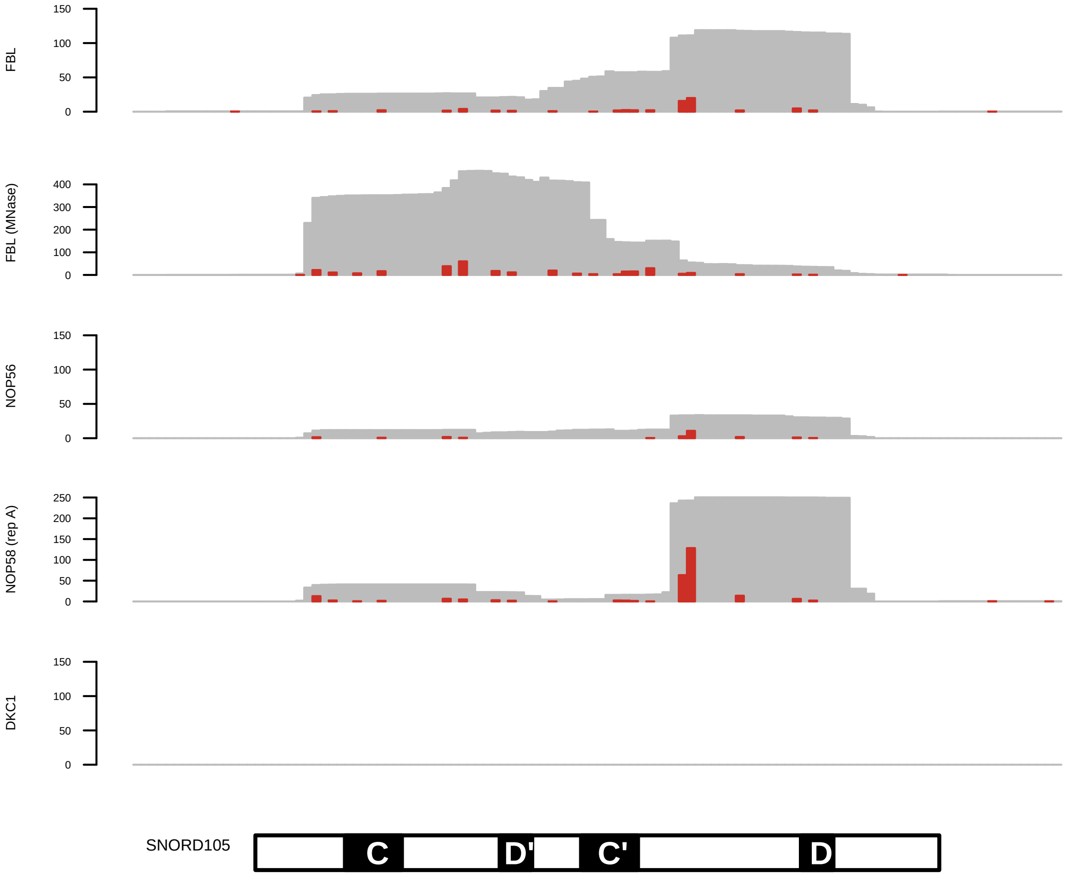

FBL
150
100
50
0
FBL (MNase)
400
300
200
100
0
NOP56
150
100
50
0
NOP58 (rep A)
250
200
150
100
50
0
DKC1
150
100
50
0
SNORD105
C
D'
C'
D

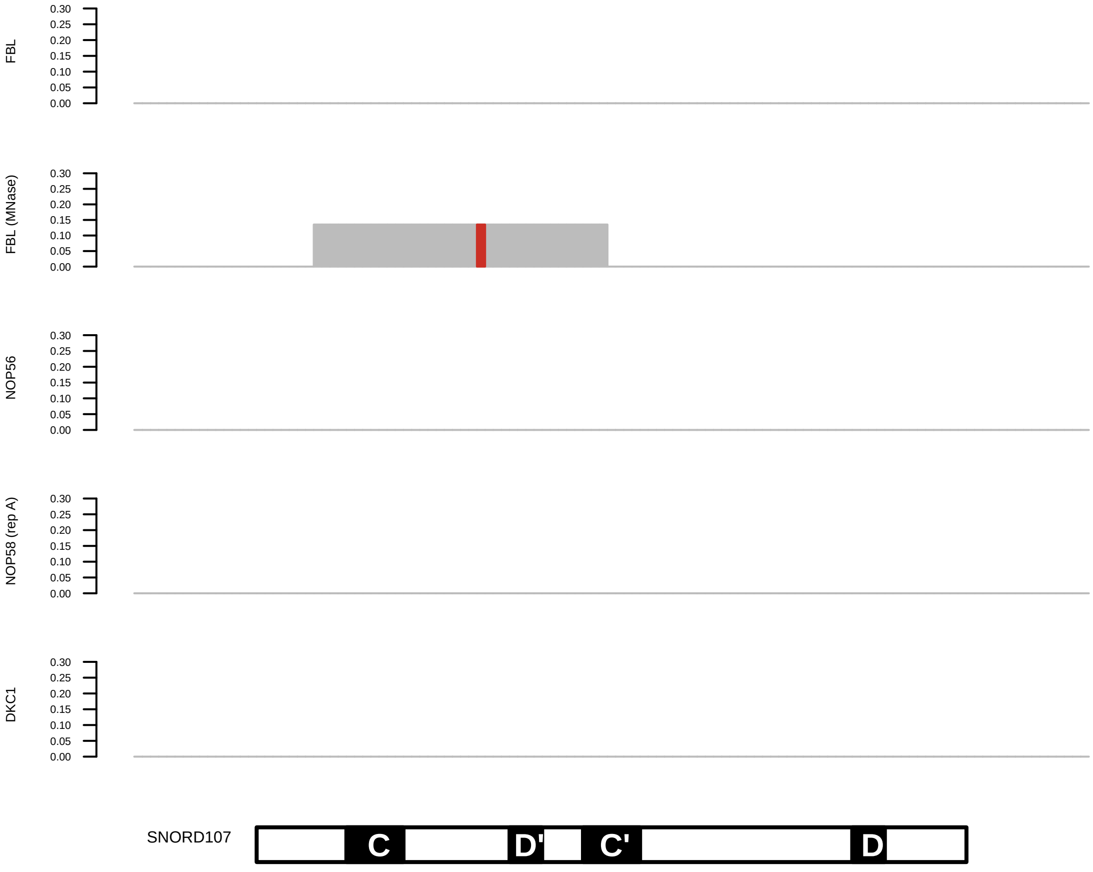
FBL
0.30
0.25
0.20
0.15
0.10
0.05
0.00
FBL (MNase)
NOP56
NOP58 (rep A)
DKC1
SNORD107
C
D'
C'
D

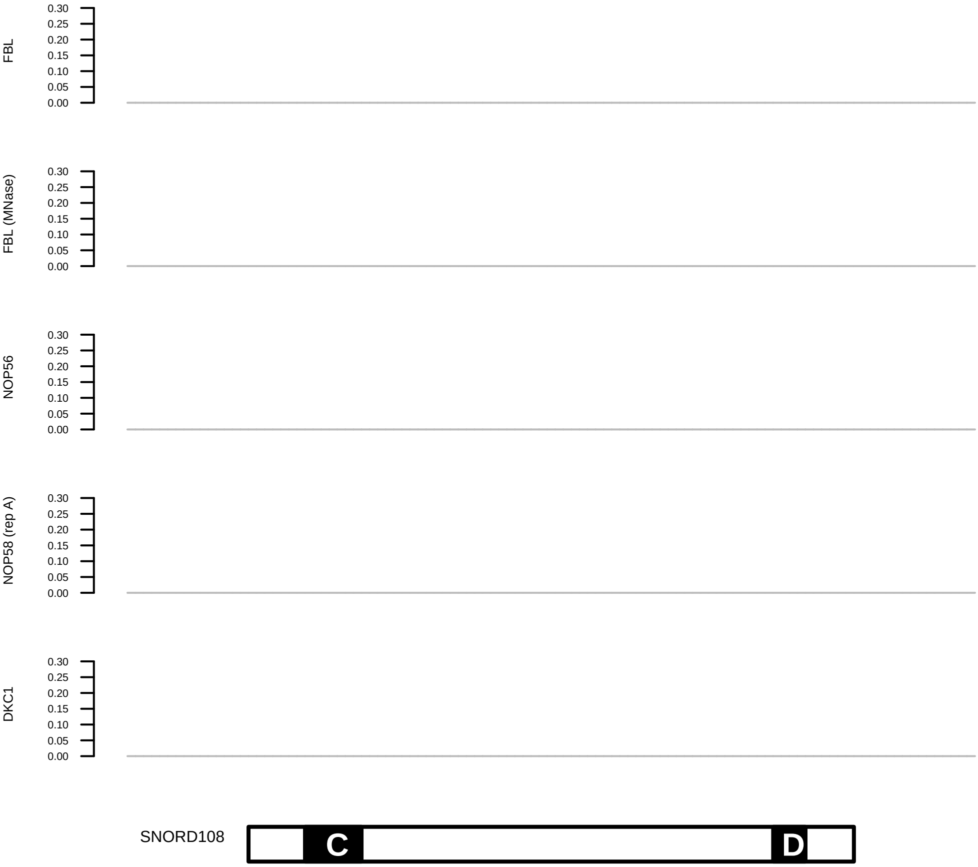
FBL
FBL (MNase)
NOP56
NOP58 (rep A)
DKC1
0.30
0.25
0.20
0.15
0.10
0.05
0.00
SNORD108
C
D

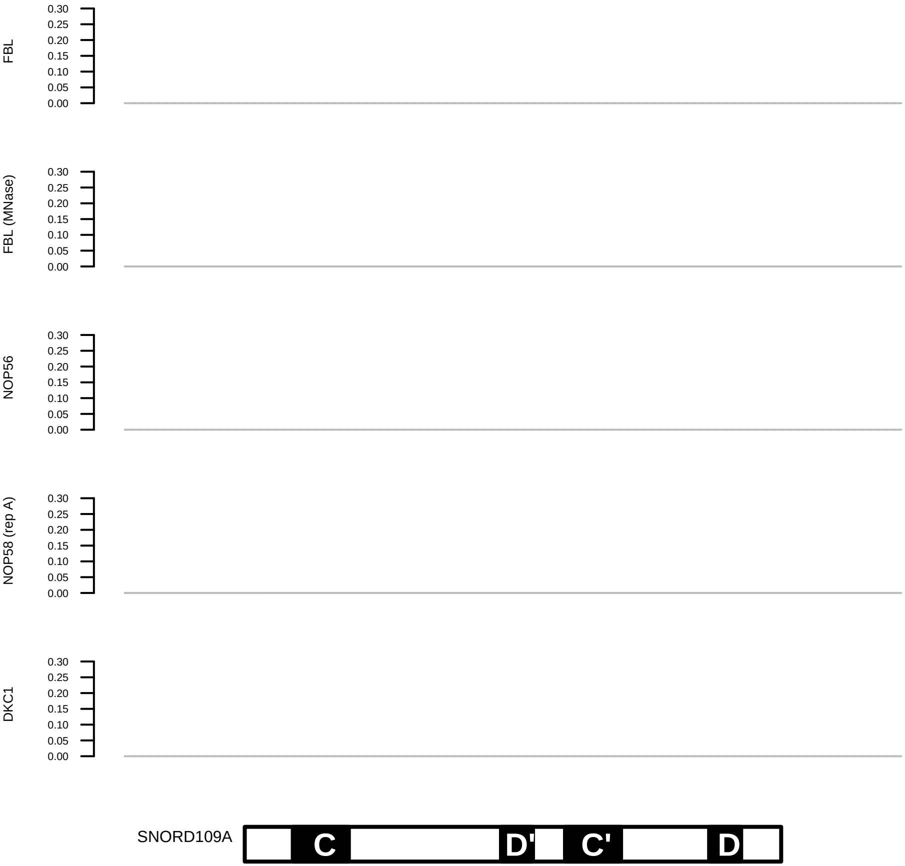
FBL
FBL (MNase)
NOP56
NOP58 (rep A)
DKC1
0.30
0.25
0.20
0.15
0.10
0.05
0.00
SNORD109A
C
D'
C'
D

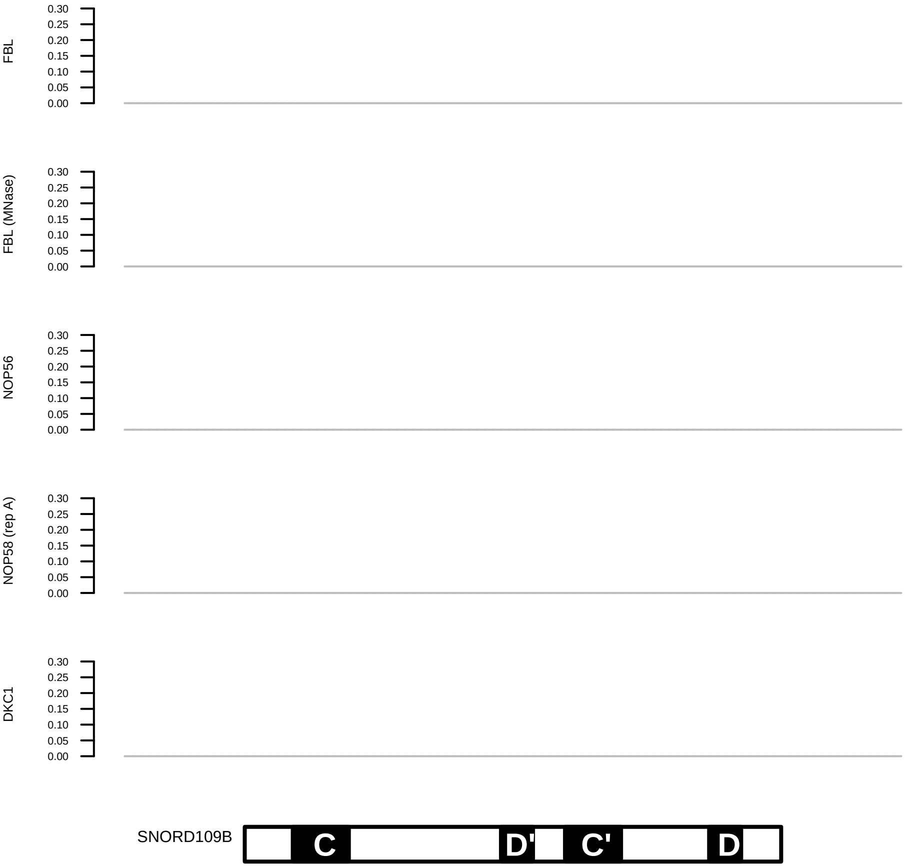
FBL
FBL (MNase)
NOP56
NOP58 (rep A)
DKC1
0.30
0.25
0.20
0.15
0.10
0.05
0.00
SNORD109B
C
D'
C'
D

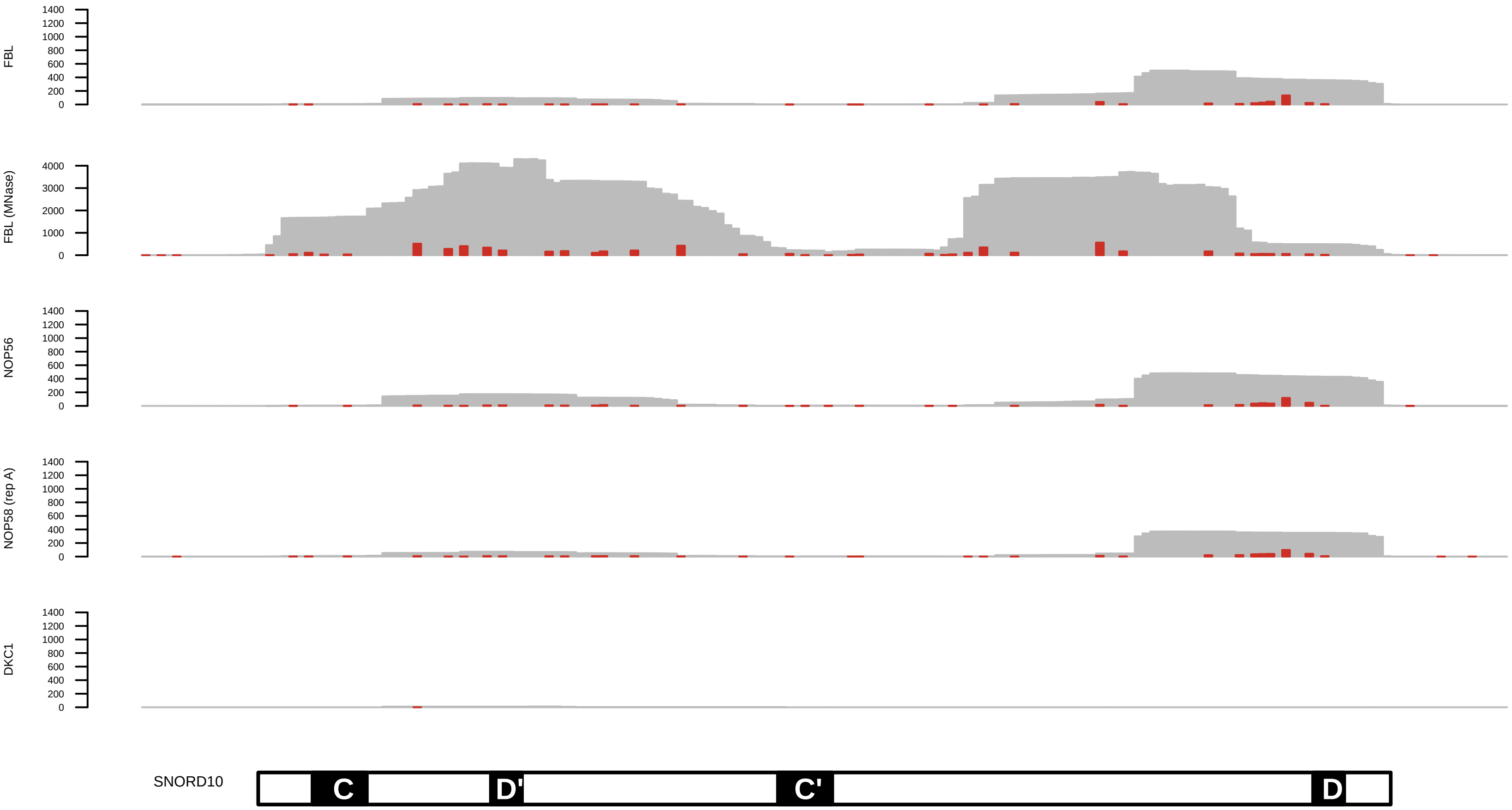

FBL
FBL (MNase)
NOP56
NOP58 (rep A)
DKC1
0
200
400
600
800
1000
1200
1400
1000
2000
3000
4000
SNORD10
C
D'
C'
D

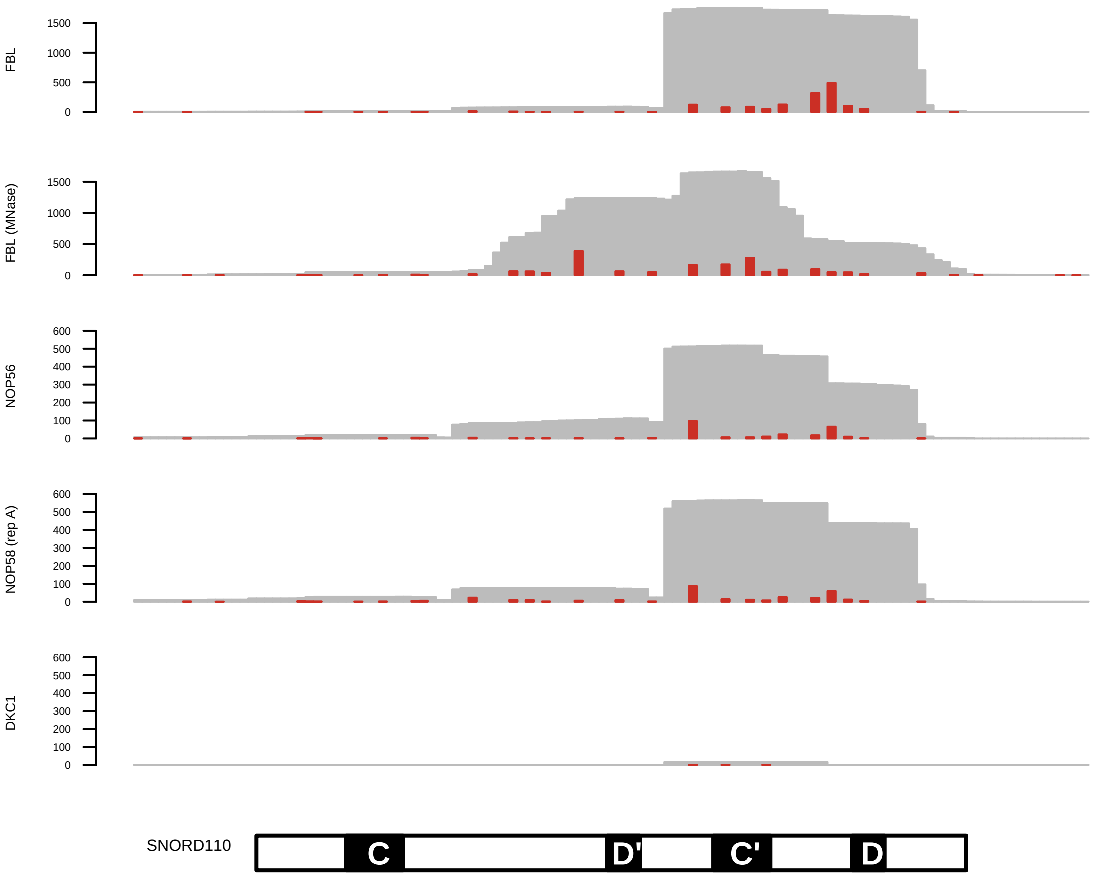
FBL
1500
1000
500
0
FBL (MNase)
1500
1000
500
0
NOP56
600
500
400
300
200
100
0
NOP58 (rep A)
600
500
400
300
200
100
0
DKC1
600
500
400
300
200
100
0
SNORD110
C
D'
C'
D

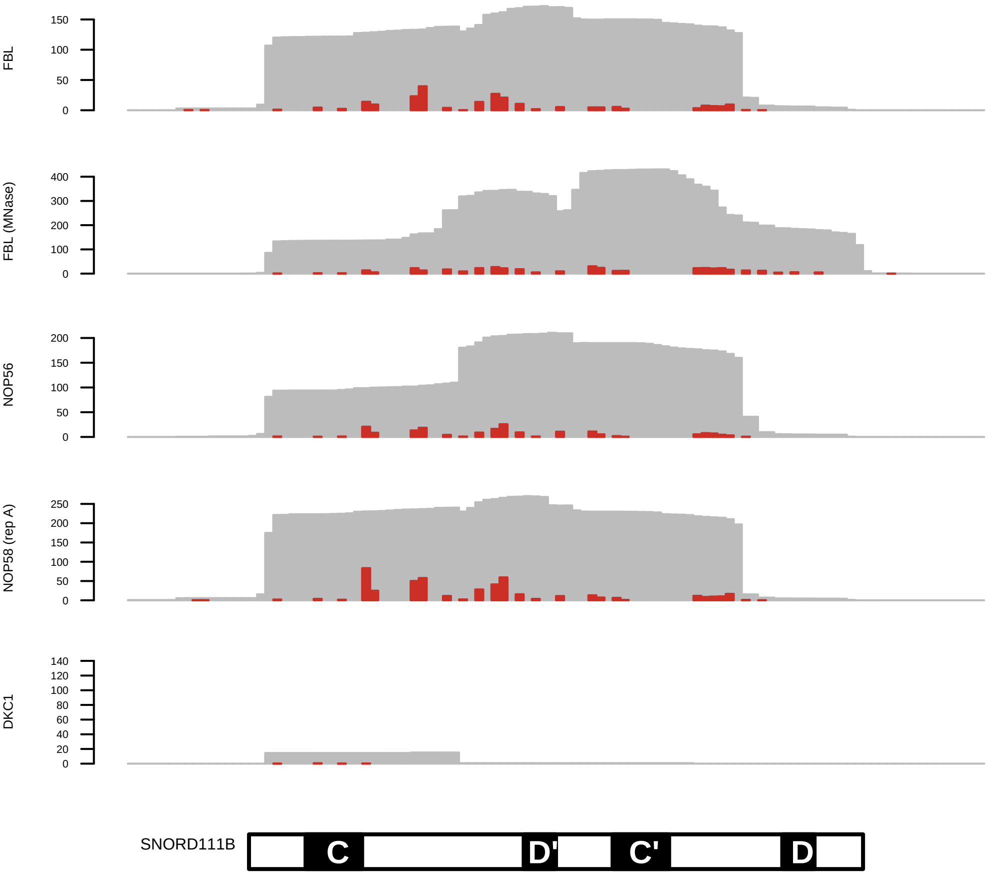
FBL
150
100
50
0
FBL (MNase)
400
300
200
100
0
NOP56
200
150
100
50
0
NOP58 (rep A)
250
200
150
100
50
0
DKC1
140
120
100
80
60
40
20
0
SNORD111B
C
D'
C'
D

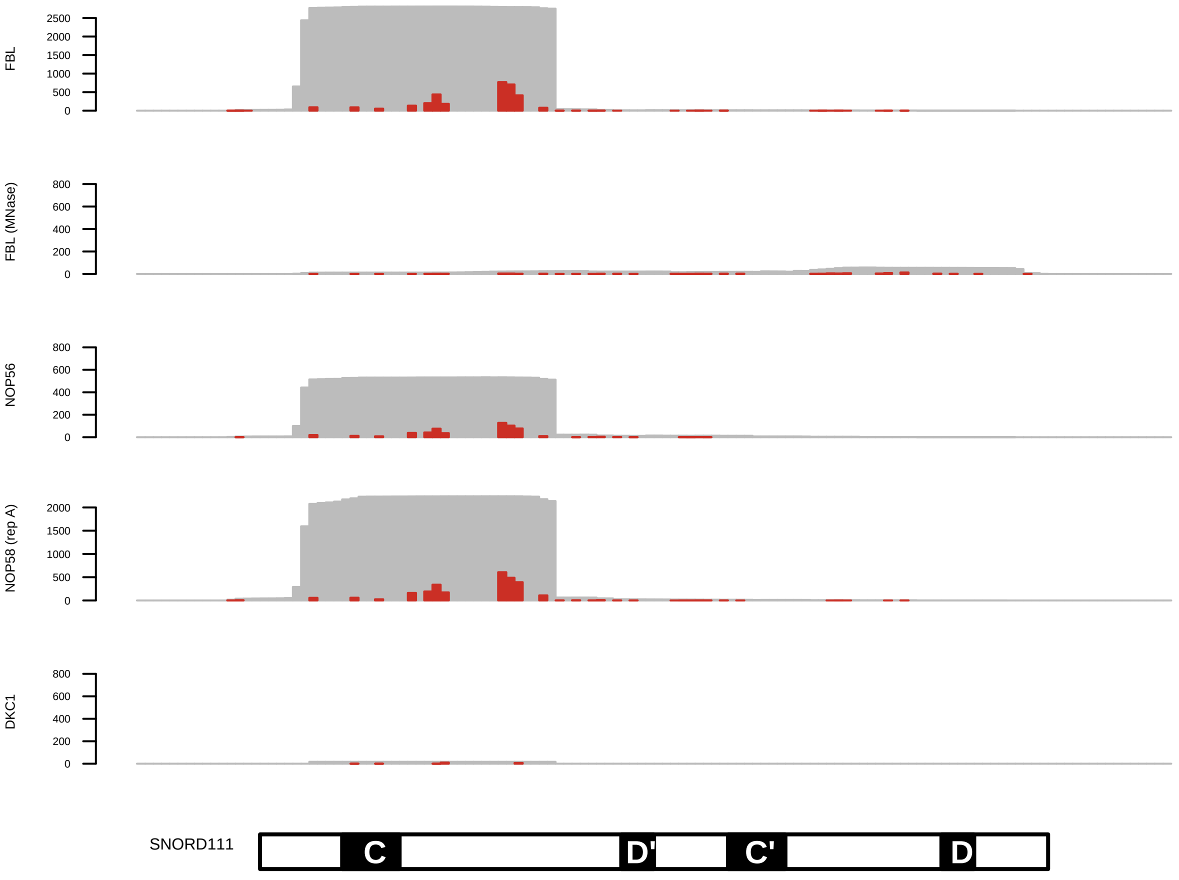

FBL
2500
2000
1500
1000
500
0
FBL (MNase)
800
600
400
200
0
NOP56
800
600
400
200
0
NOP58 (rep A)
2000
1500
1000
500
0
DKC1
800
600
400
200
0
SNORD111
C
D'
C'
D

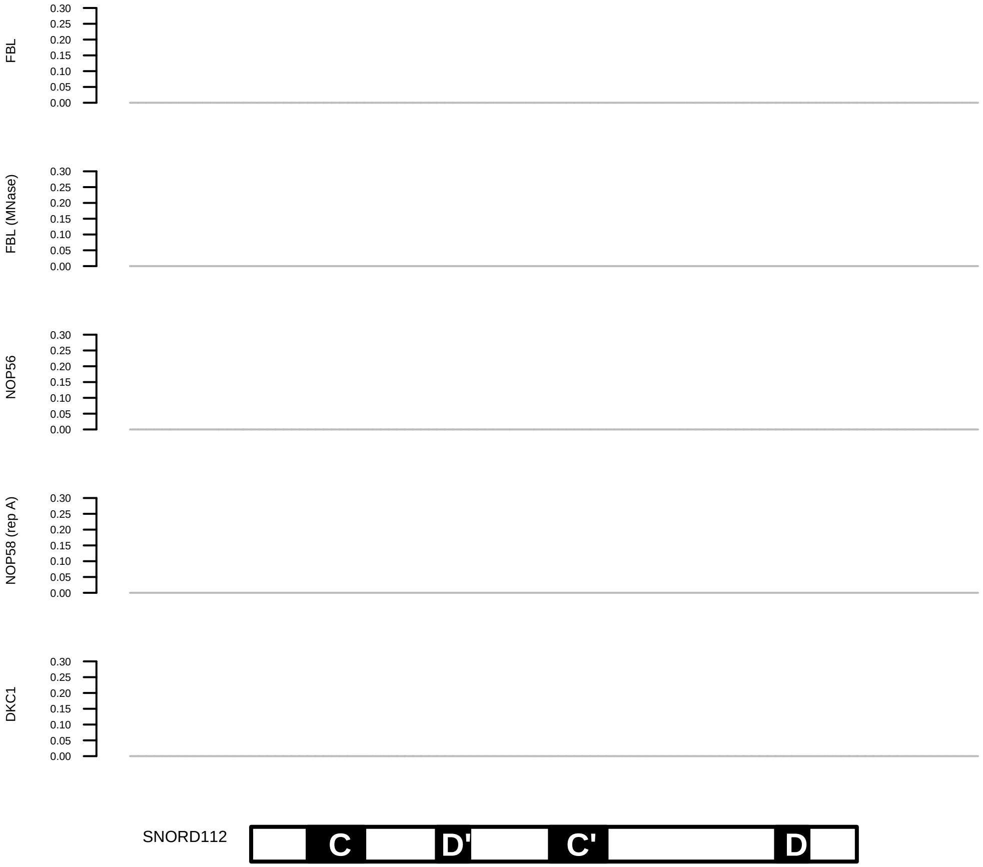

FBL
FBL (MNase)
NOP56
NOP58 (rep A)
DKC1
0.30
0.25
0.20
0.15
0.10
0.05
0.00
SNORD112
C
D'
C'
D

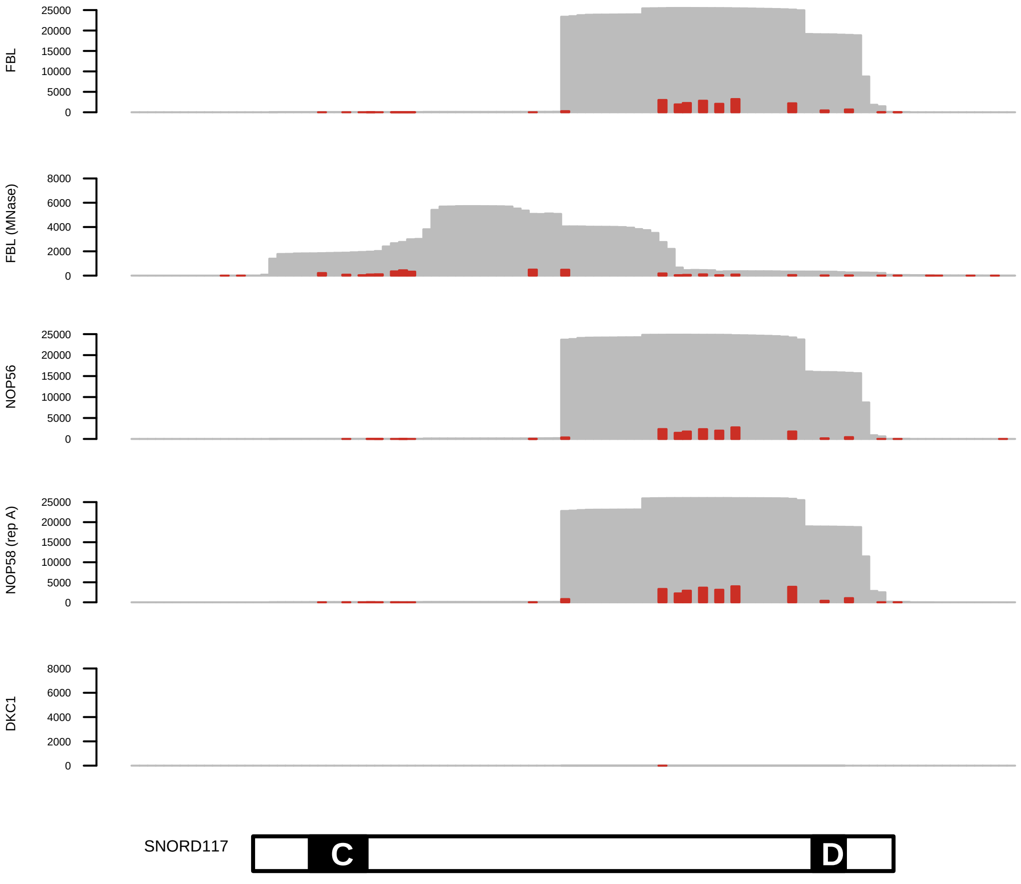

FBL
25000
20000
15000
10000
5000
0
FBL (MNase)
8000
6000
4000
2000
0
NOP56
25000
20000
15000
10000
5000
0
NOP58 (rep A)
25000
20000
15000
10000
5000
0
DKC1
8000
6000
4000
2000
0
SNORD117
C
D

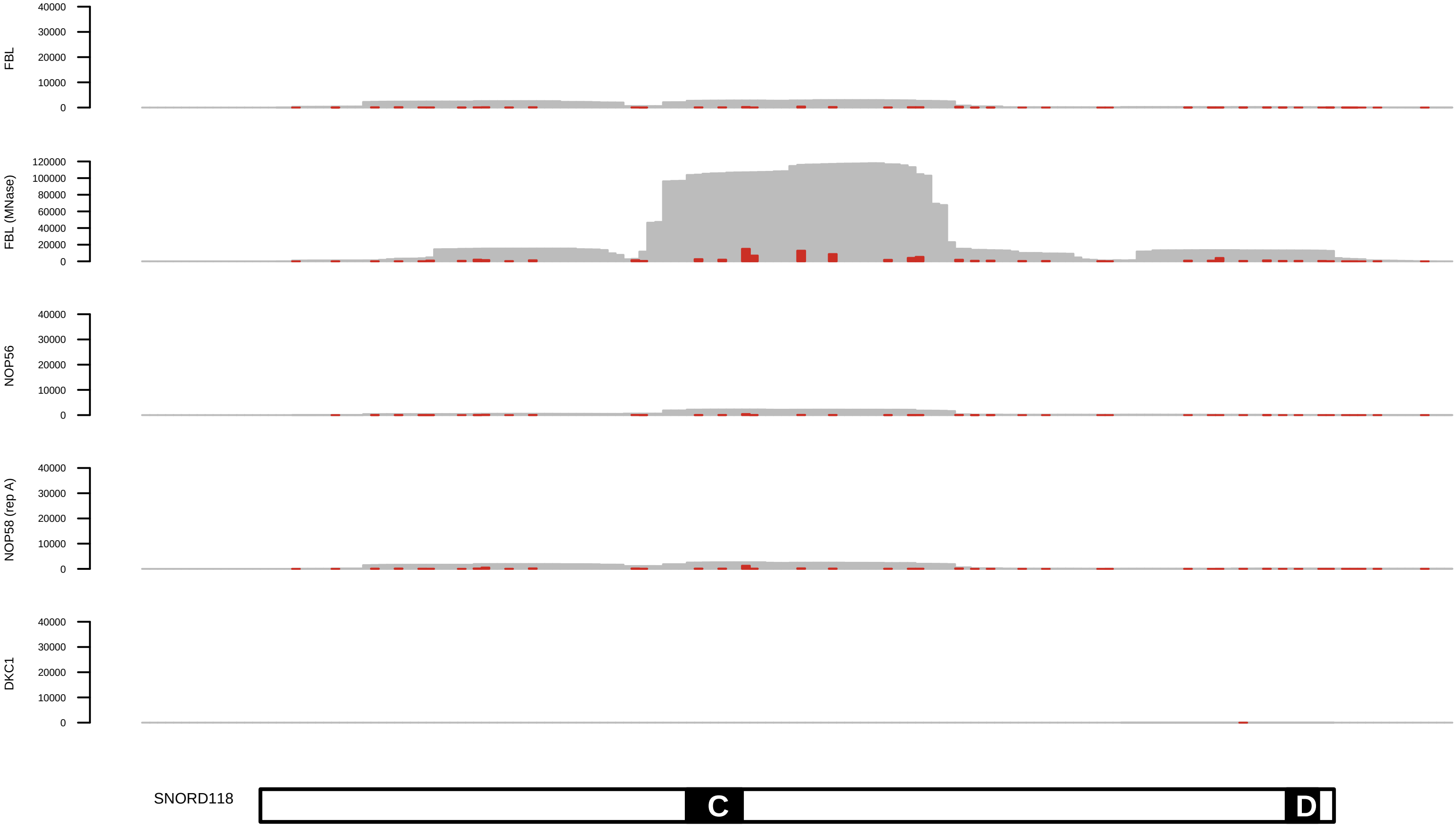
FBL
40000
30000
20000
10000
0
FBL (MNase)
120000
100000
80000
60000
40000
20000
0
NOP56
40000
30000
20000
10000
0
NOP58 (rep A)
40000
30000
20000
10000
0
DKC1
40000
30000
20000
10000
0
SNORD118
C
D

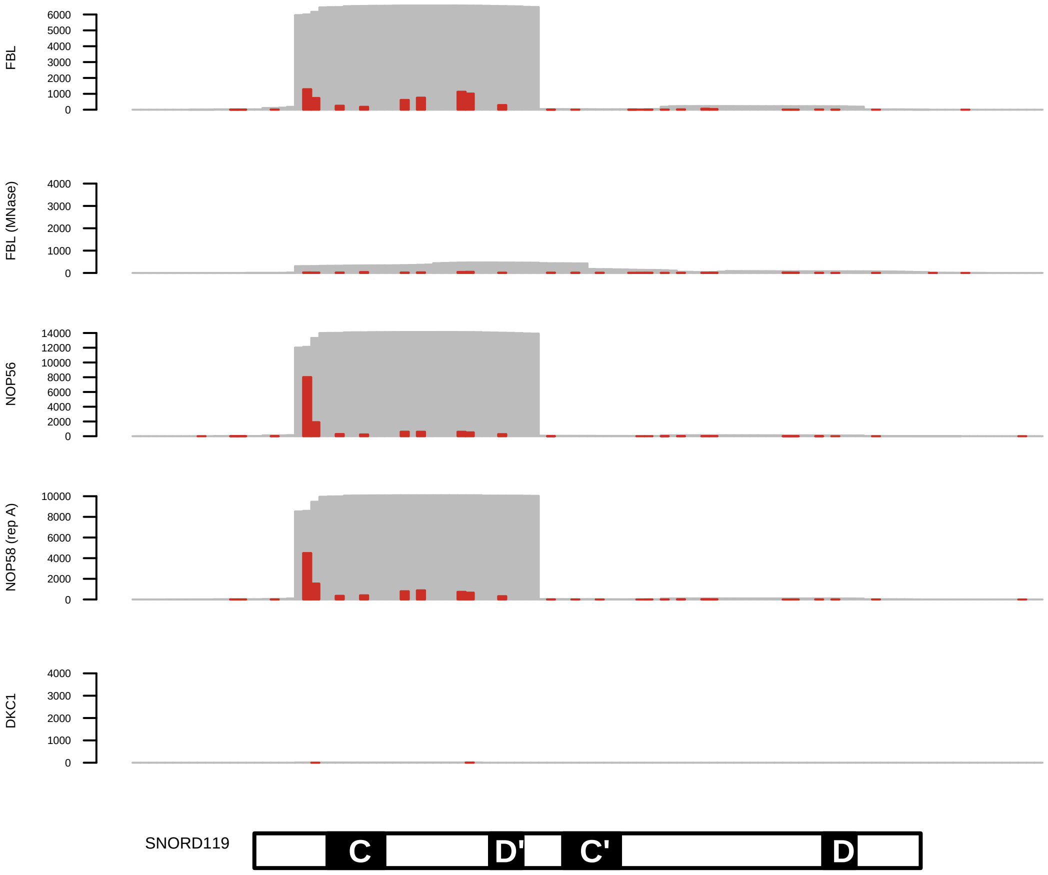

FBL
6000
5000
4000
3000
2000
1000
0
FBL (MNase)
4000
3000
2000
1000
0
NOP56
14000
12000
10000
8000
6000
4000
2000
0
NOP58 (rep A)
10000
8000
6000
4000
2000
0
DKC1
4000
3000
2000
1000
0
SNORD119
C
D'
C'
D

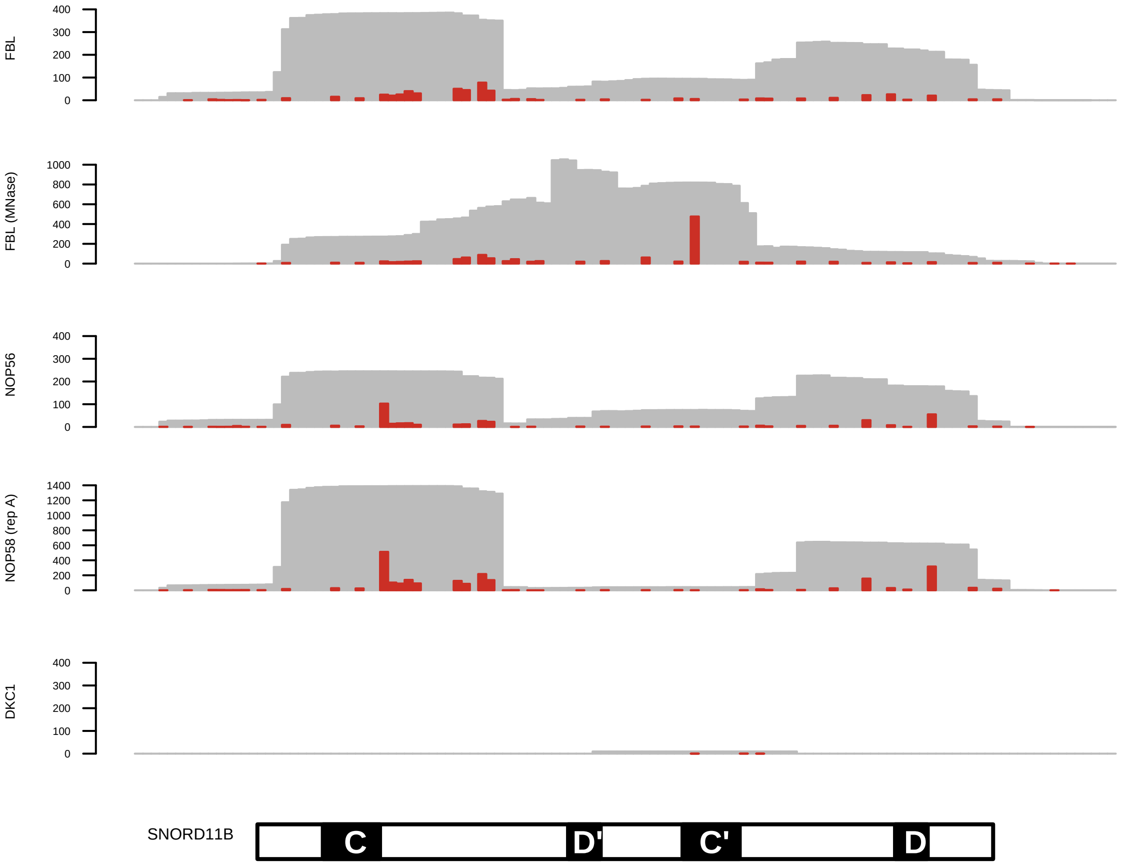
FBL
FBL (MNase)
NOP56
NOP58 (rep A)
DKC1
SNORD11B
C
D'
C'
D

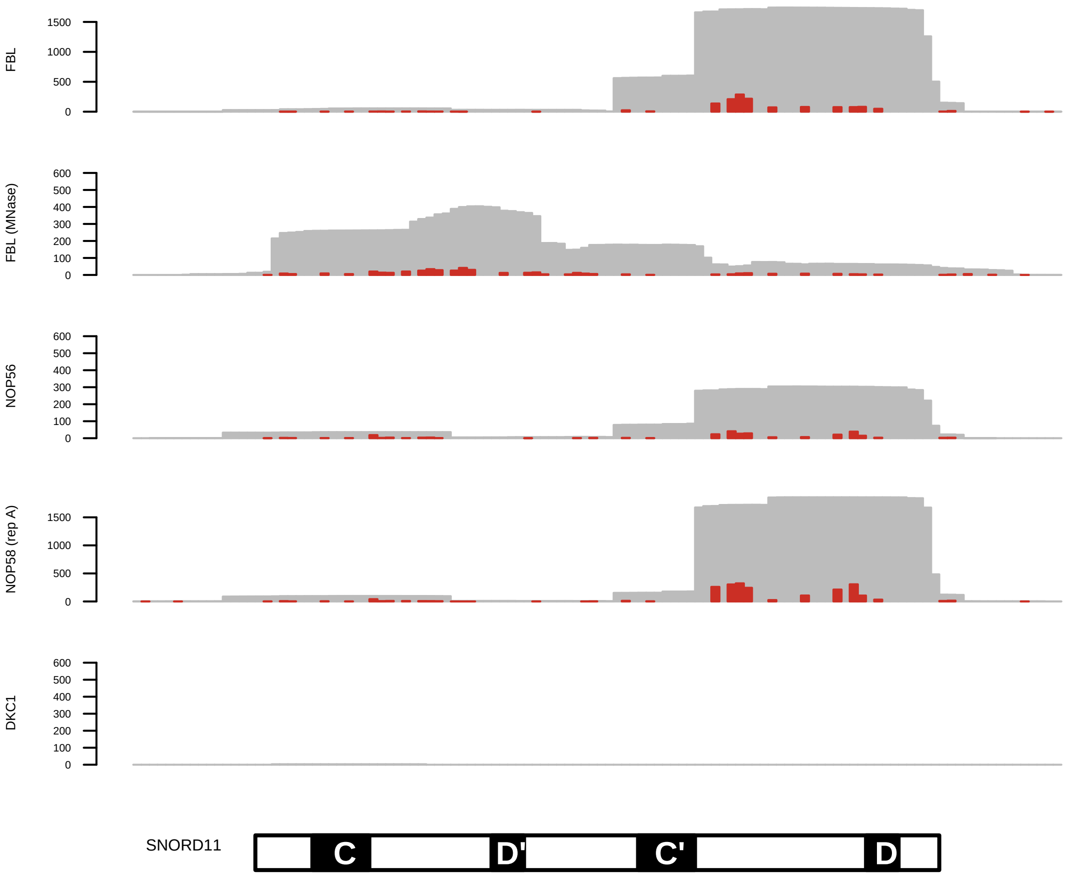

FBL
1500
1000
500
0
FBL (MNase)
600
500
400
300
200
100
0
NOP56
600
500
400
300
200
100
0
NOP58 (rep A)
1500
1000
500
0
DKC1
600
500
400
300
200
100
0
SNORD11
C
D'
C'
D

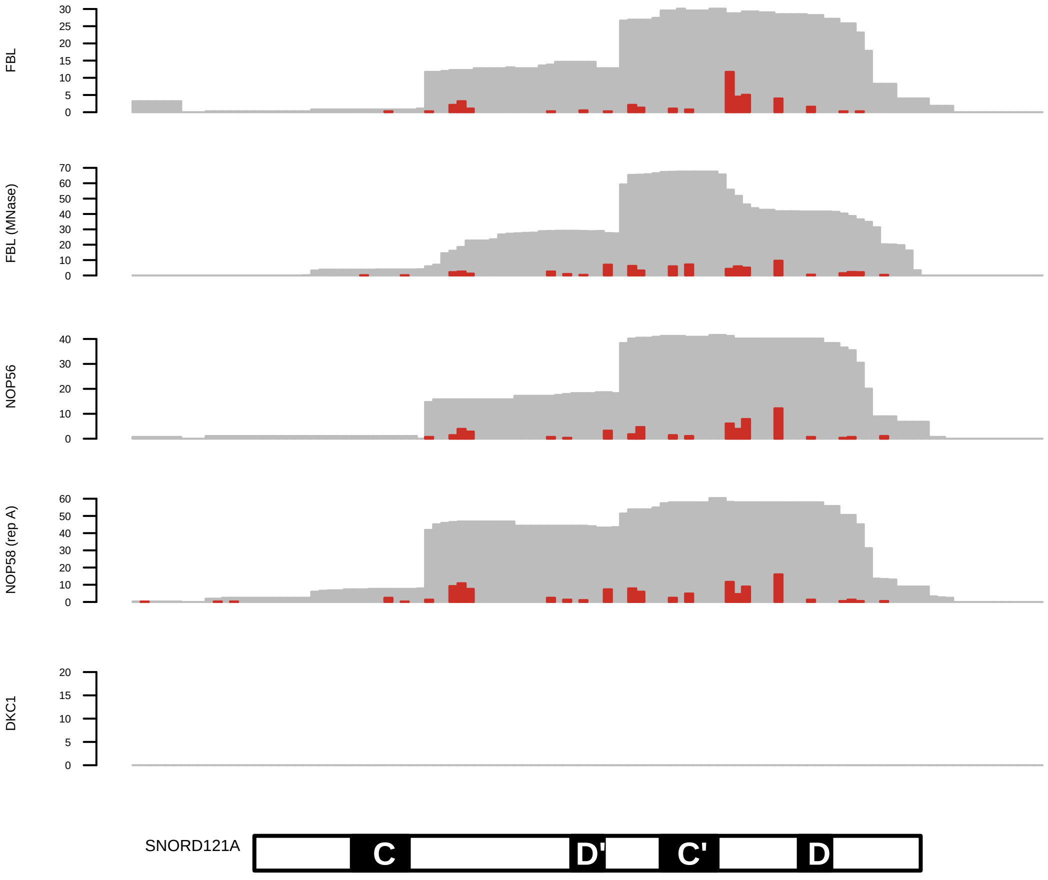
FBL
30
25
20
15
10
5
0
FBL (MNase)
70
60
50
40
30
20
10
0
NOP56
40
30
20
10
0
NOP58 (rep A)
60
50
40
30
20
10
0
DKC1
20
15
10
5
0
SNORD121A
C
D'
C'
D

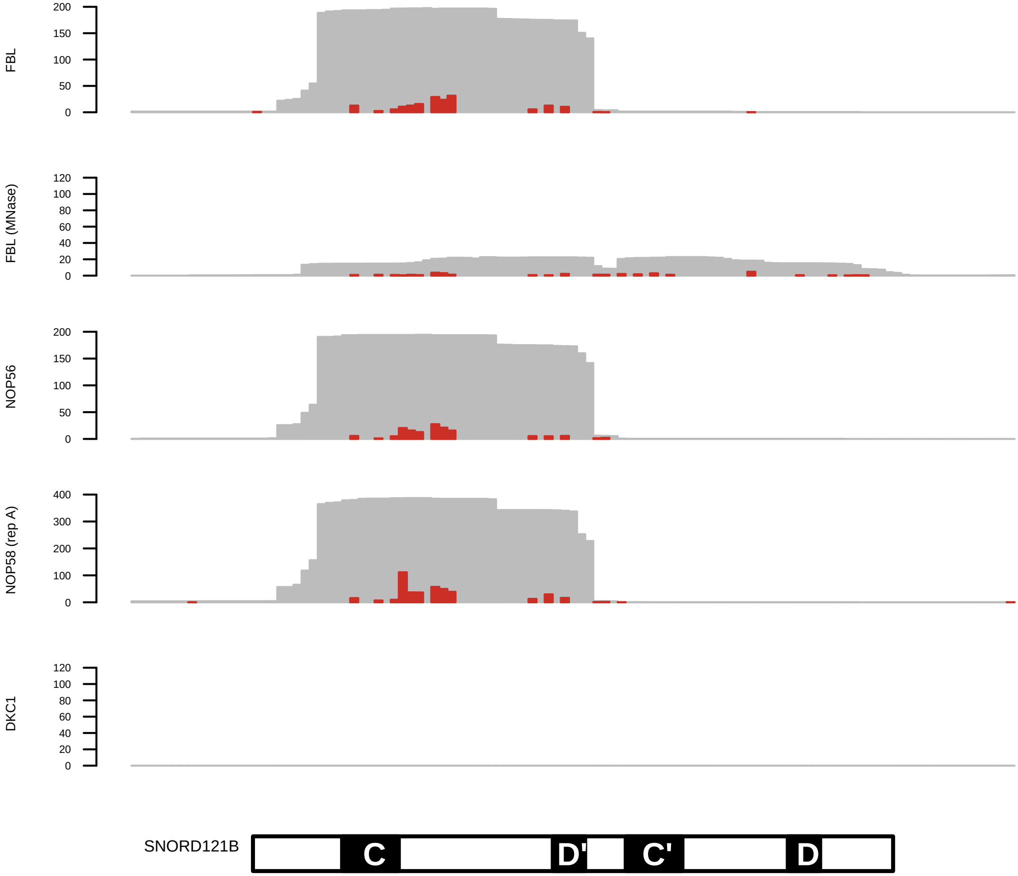

FBL
FBL (MNase)
NOP56
NOP58 (rep A)
DKC1
0
50
100
150
200
0
20
40
60
80
100
120
300
400
SNORD121B
C
D'
C'
D

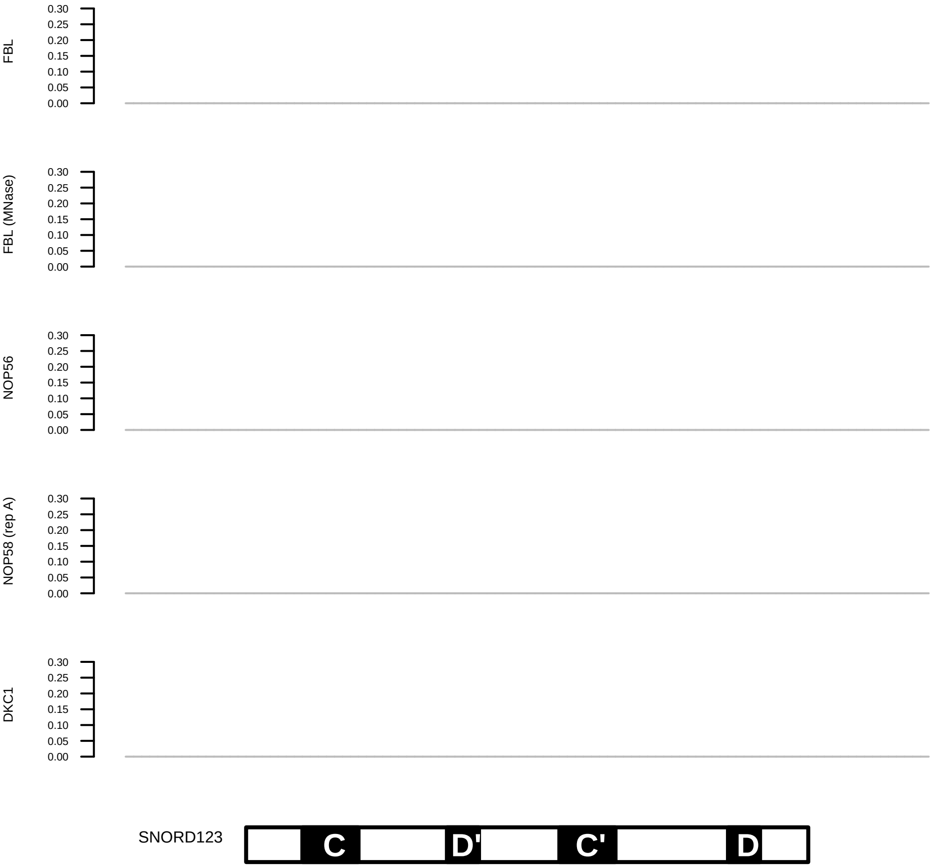
FBL
0.30
0.25
0.20
0.15
0.10
0.05
0.00
FBL (MNase)
NOP56
NOP58 (rep A)
DKC1
SNORD123
C
D'
C'
D

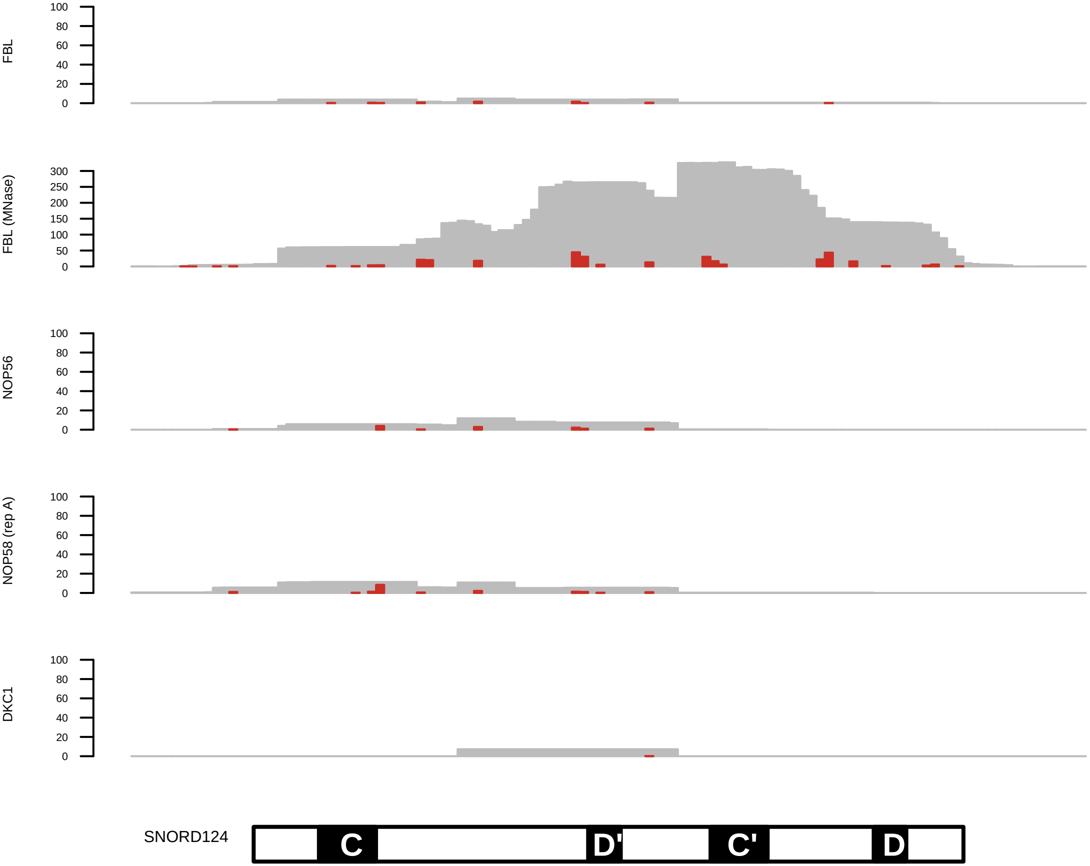

FBL
FBL (MNase)
NOP56
NOP58 (rep A)
DKC1
0
20
40
60
80
100
50
150
200
250
300
SNORD124
C
D'
C'
D

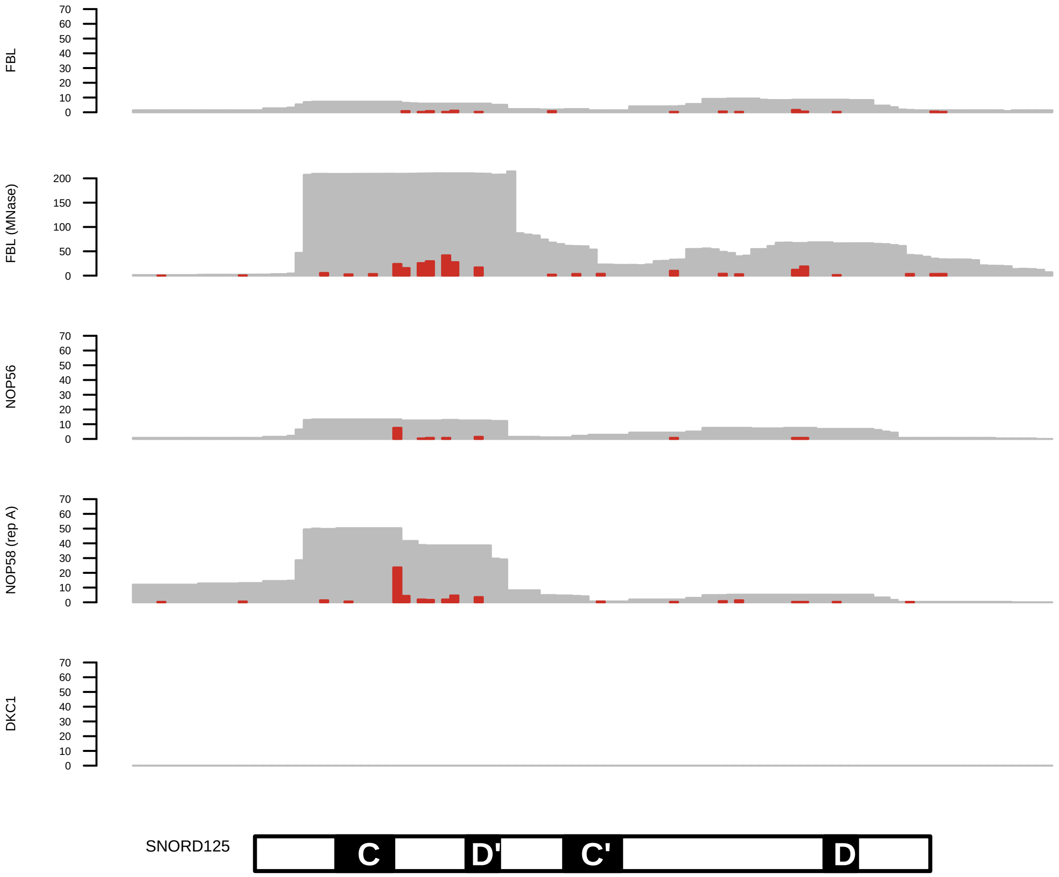

FBL
FBL (MNase)
NOP56
NOP58 (rep A)
DKC1
0
10
20
30
40
50
60
70
0
50
100
150
200
SNORD125
C
D'
C'
D

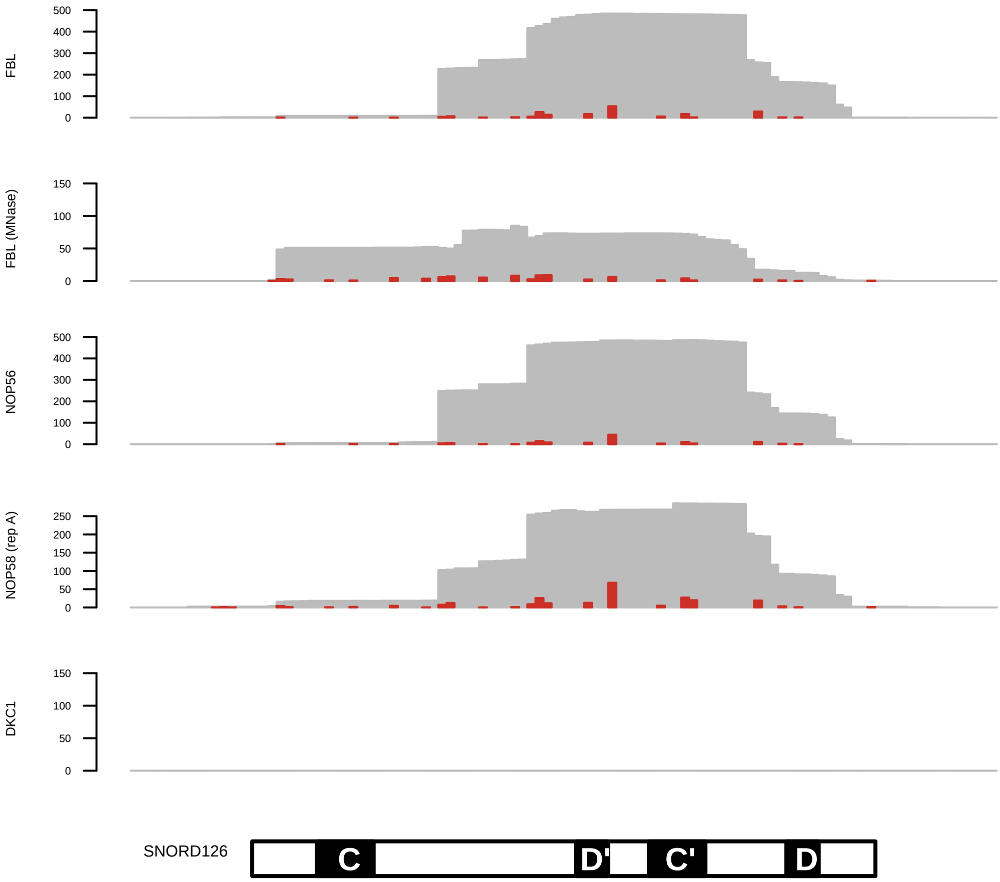
FBL
500
400
300
200
100
0
FBL (MNase)
150
100
50
0
NOP56
500
400
300
200
100
0
NOP58 (rep A)
250
200
150
100
50
0
DKC1
150
100
50
0
SNORD126
C
D'
C'
D

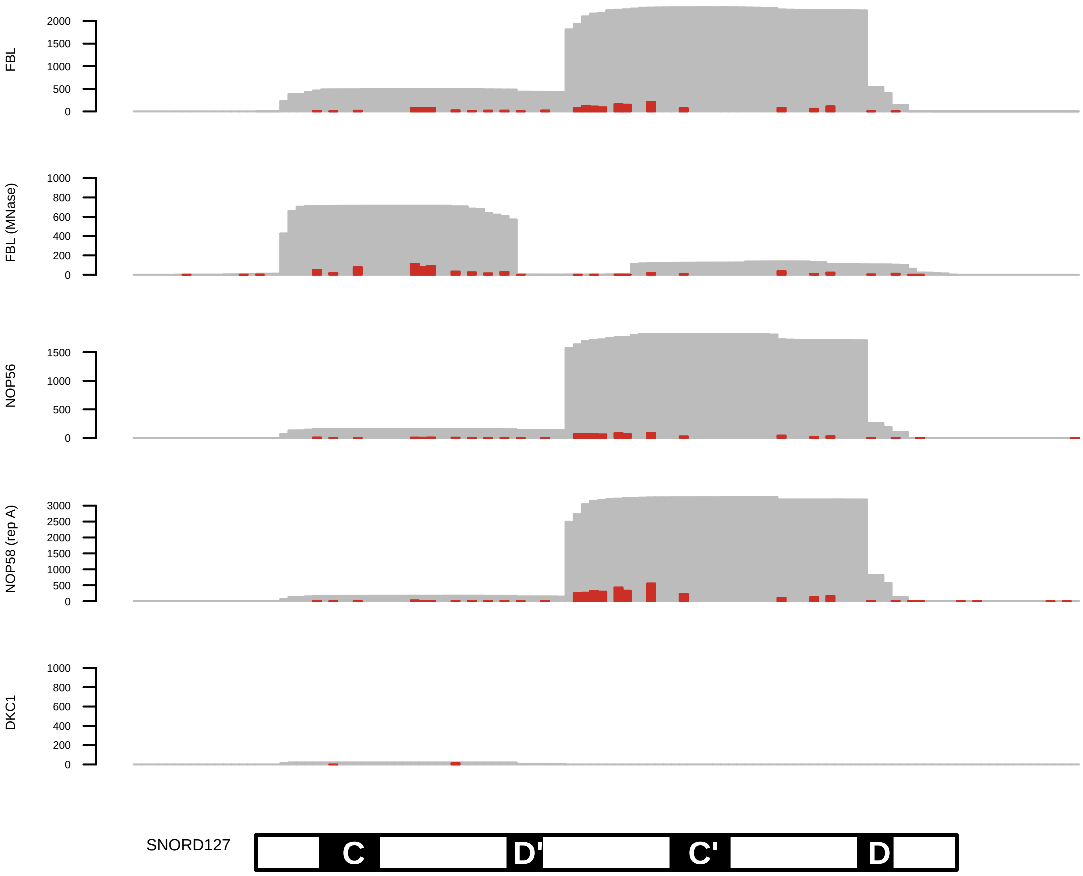

FBL
2000
1500
1000
500
0
FBL (MNase)
1000
800
600
400
200
0
NOP56
1500
1000
500
0
NOP58 (rep A)
3000
2500
2000
1500
1000
500
0
DKC1
1000
800
600
400
200
0
SNORD127
C
D'
C'
D

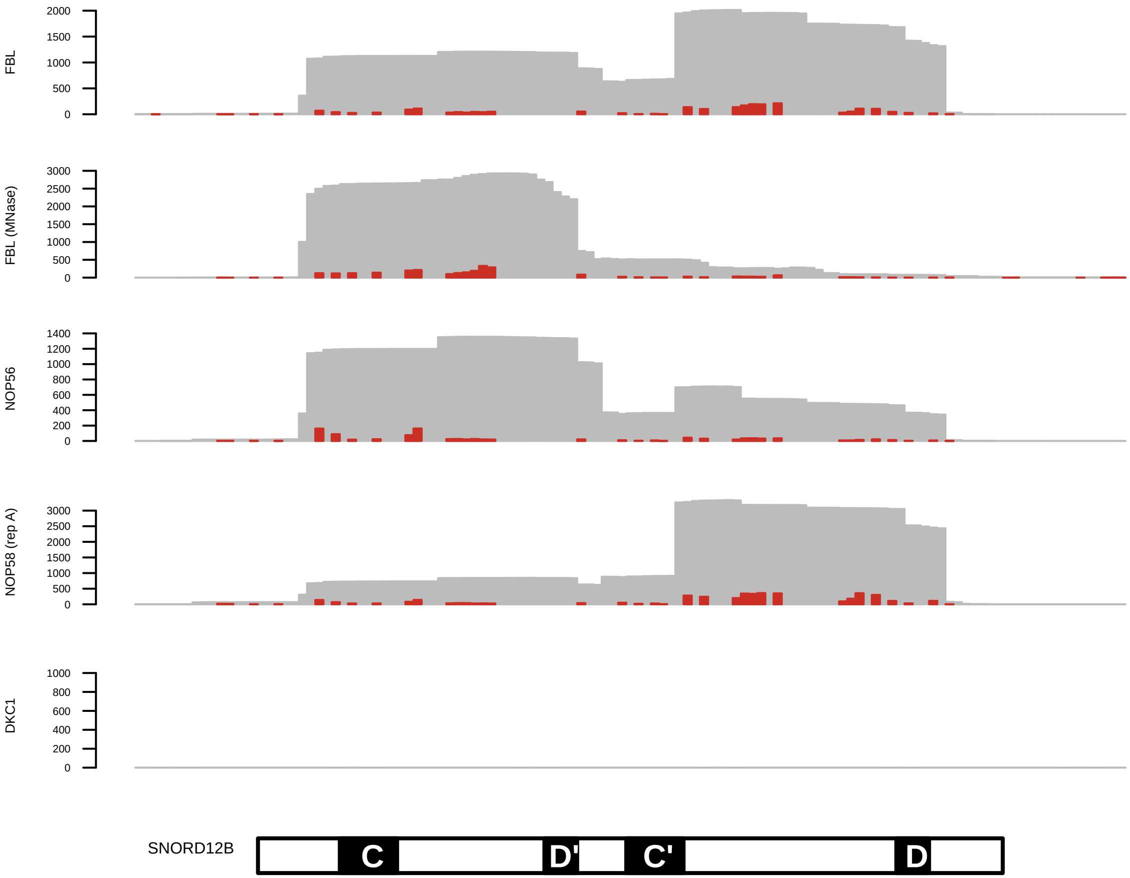

FBL
2000
1500
1000
500
0
FBL (MNase)
3000
2500
2000
1500
1000
500
0
NOP56
1400
1200
1000
800
600
400
200
0
NOP58 (rep A)
3000
2500
2000
1500
1000
500
0
DKC1
1000
800
600
400
200
0
SNORD12B
C
D'
C'
D

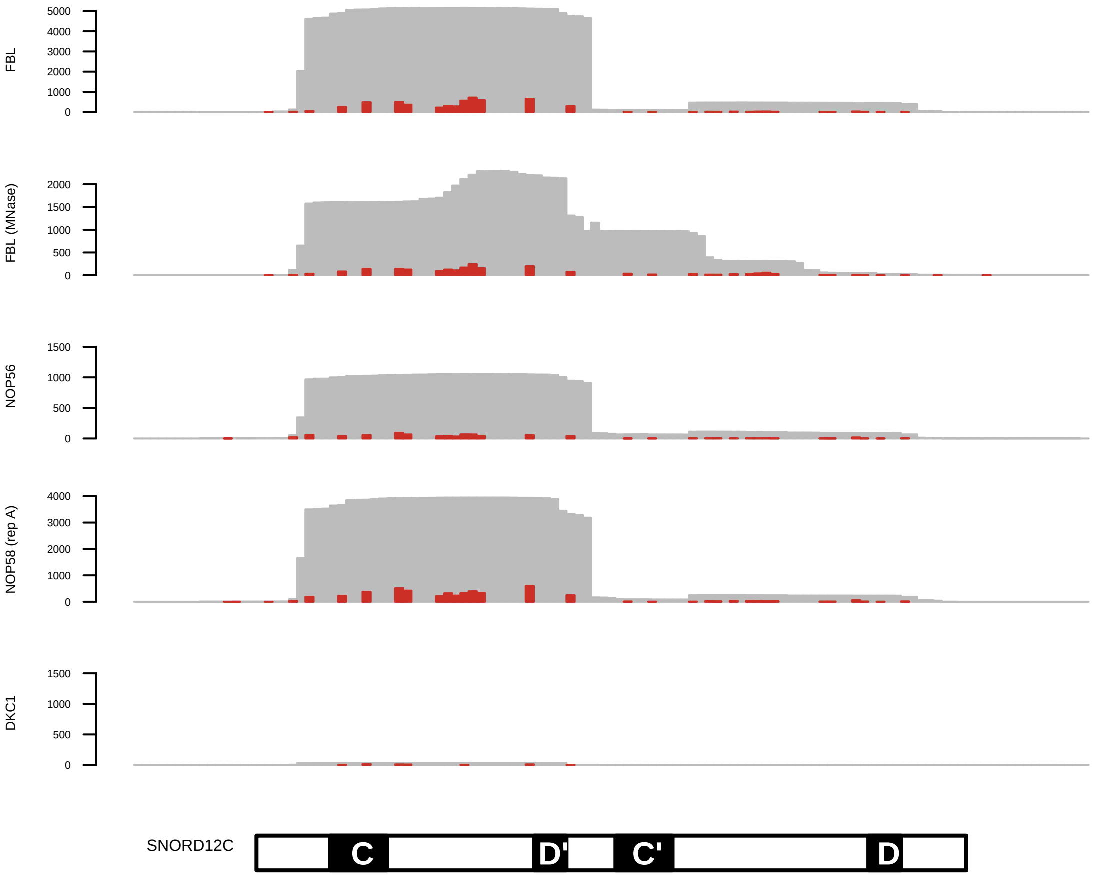
FBL
5000
4000
3000
2000
1000
0
FBL (MNase)
2000
1500
1000
500
0
NOP56
1500
1000
500
0
NOP58 (rep A)
4000
3000
2000
1000
0
DKC1
1500
1000
500
0
SNORD12C
C
D'
C'
D

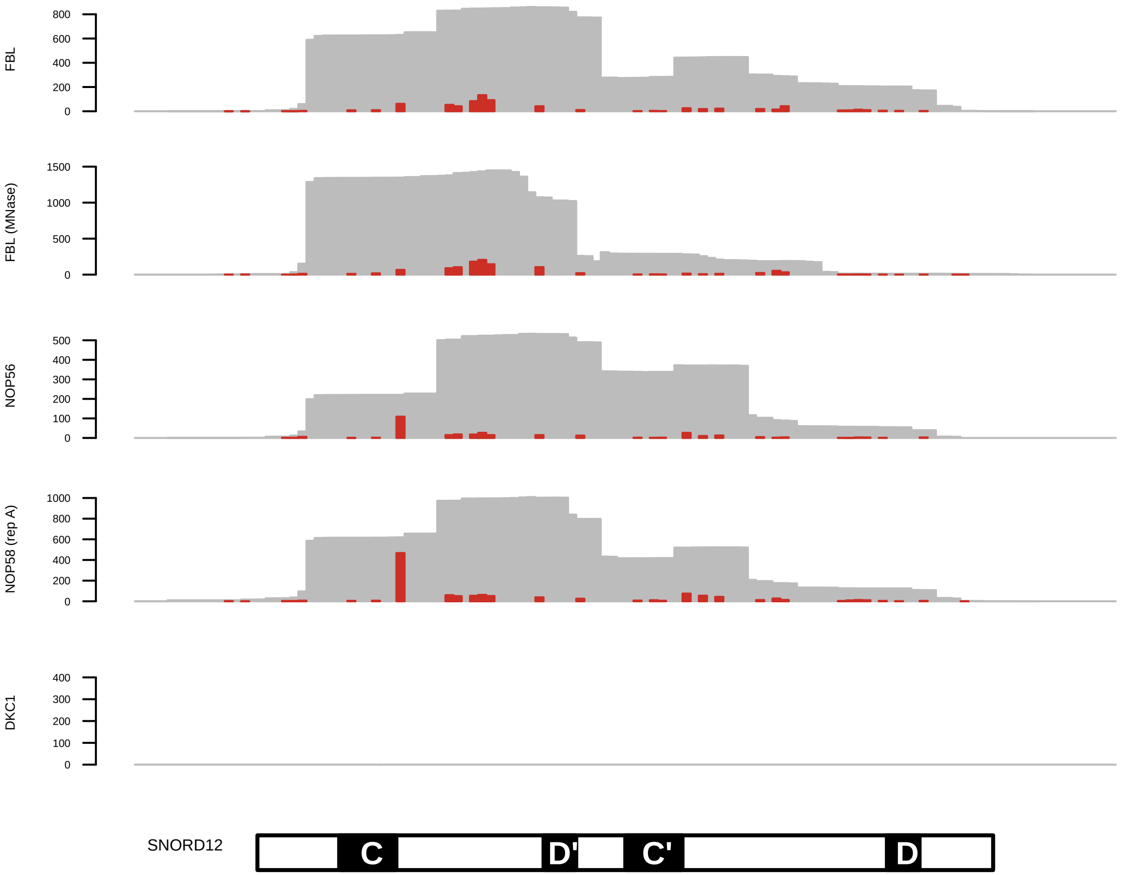
FBL
800
600
400
200
0
FBL (MNase)
1500
1000
500
0
NOP56
500
400
300
200
100
0
NOP58 (rep A)
1000
800
600
400
200
0
DKC1
400
300
200
100
0
SNORD12
C
D'
C'
D

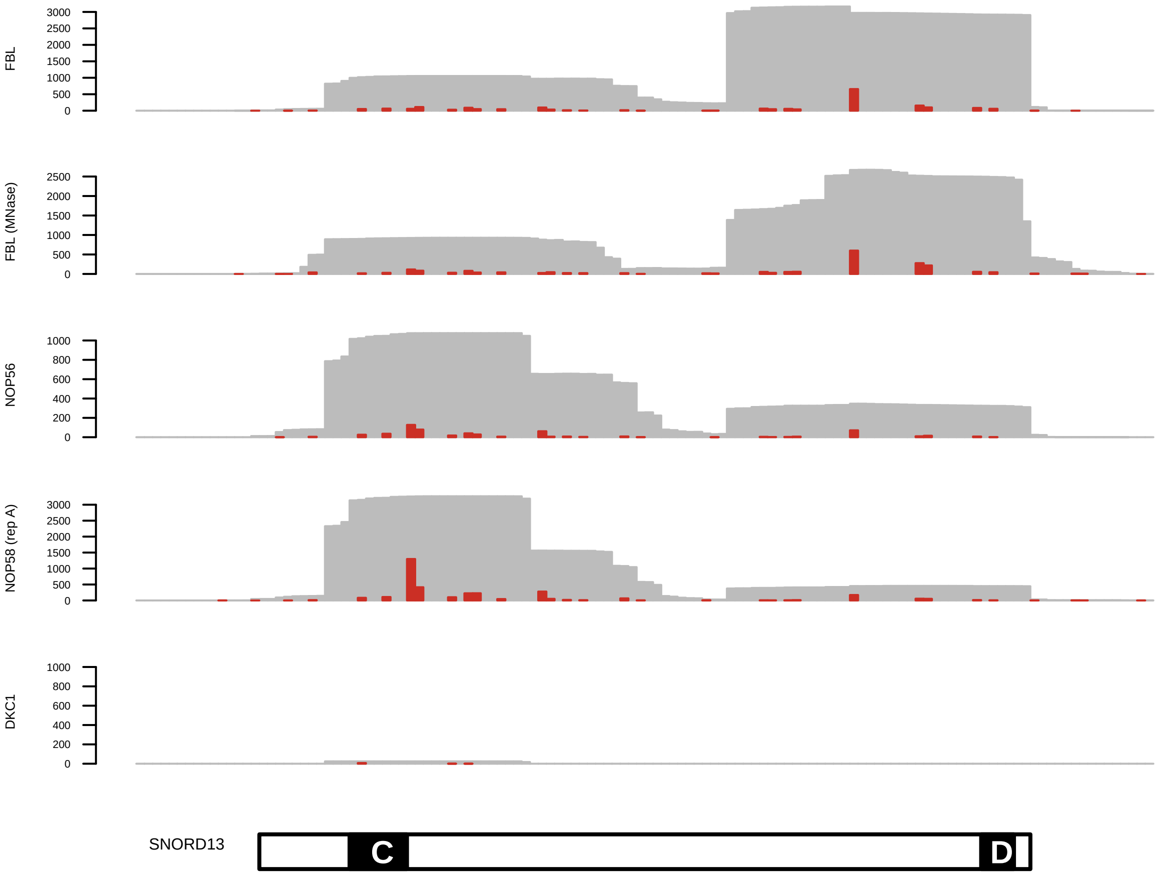

FBL
3000
2500
2000
1500
1000
500
0
FBL (MNase)
2500
2000
1500
1000
500
0
NOP56
1000
800
600
400
200
0
NOP58 (rep A)
3000
2500
2000
1500
1000
500
0
DKC1
1000
800
600
400
200
0
SNORD13
C
D

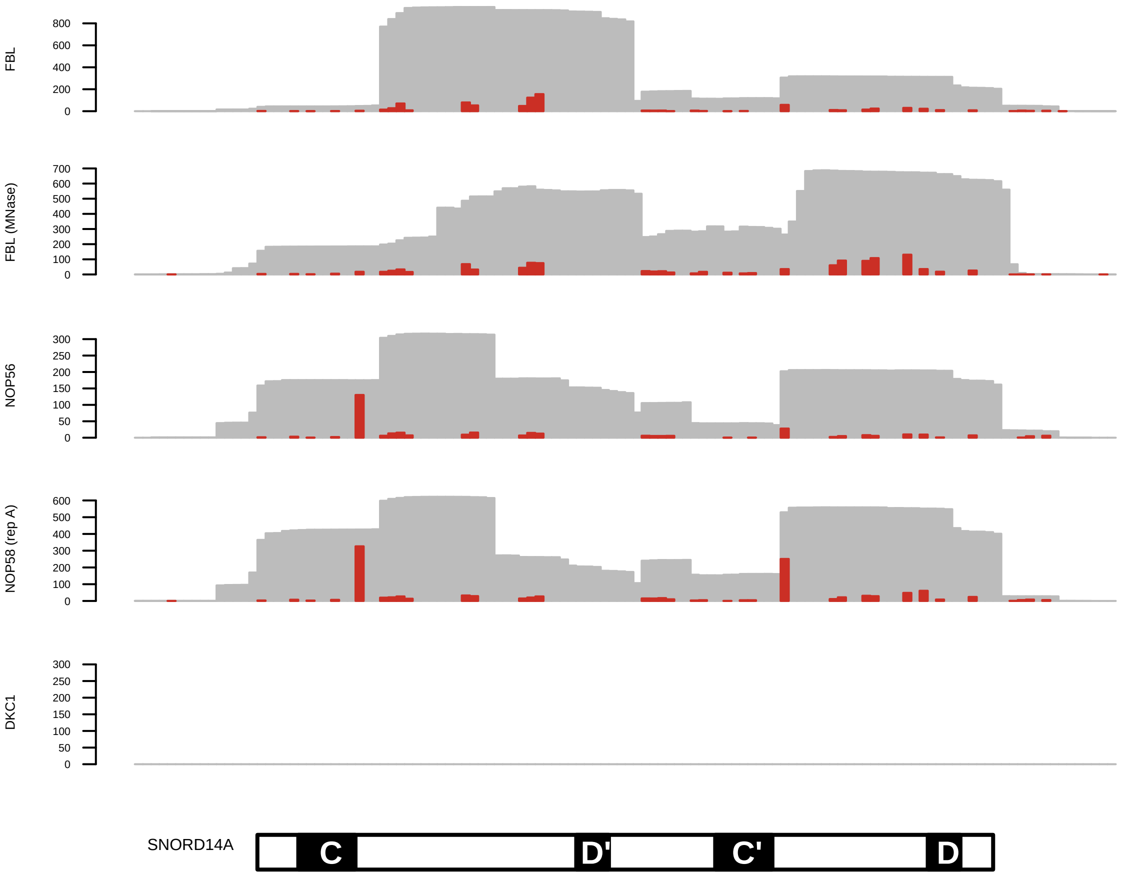
FBL
0
200
400
600
800
FBL (MNase)
0
100
200
300
400
500
600
700
NOP56
0
50
100
150
200
250
300
NOP58 (rep A)
0
100
200
300
400
500
600
DKC1
0
50
100
150
200
250
300
SNORD14A
C
D'
C'
D

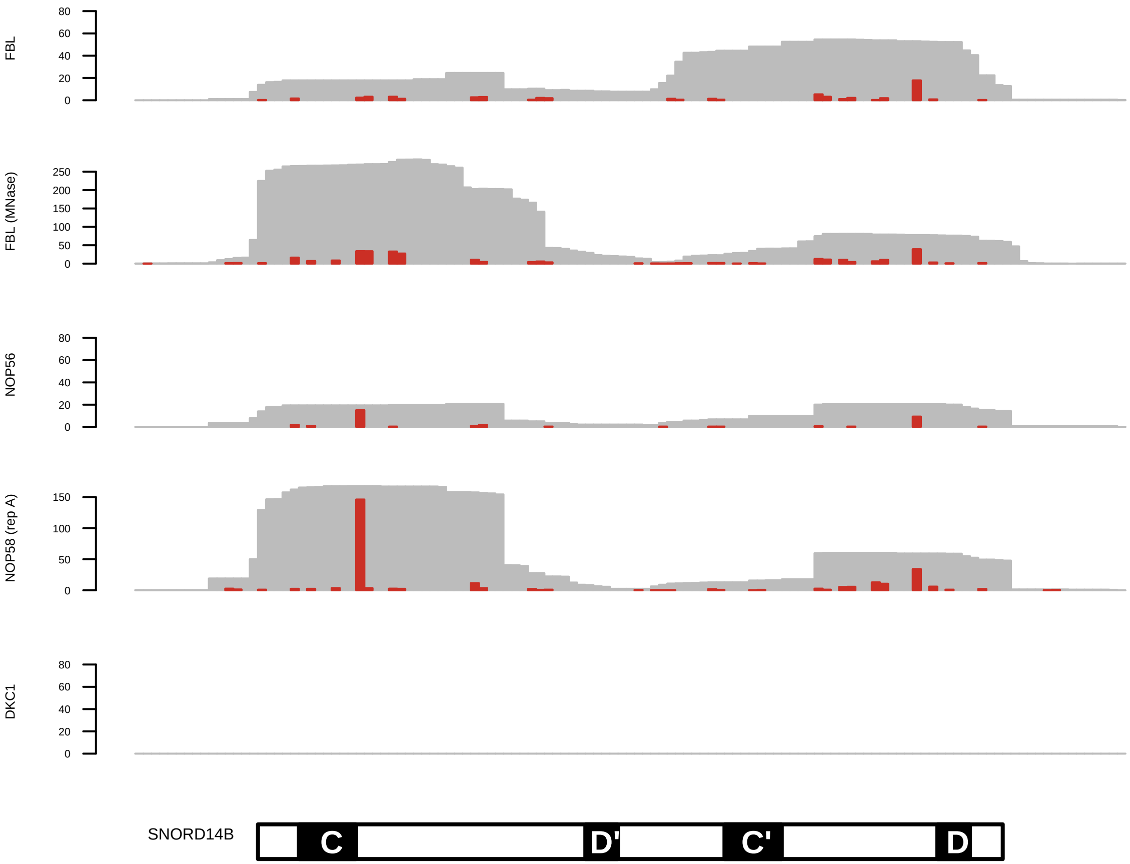
FBL
80
60
40
20
0
FBL (MNase)
250
200
150
100
50
0
NOP56
80
60
40
20
0
NOP58 (rep A)
150
100
50
0
DKC1
80
60
40
20
0
SNORD14B
C
D'
C'
D

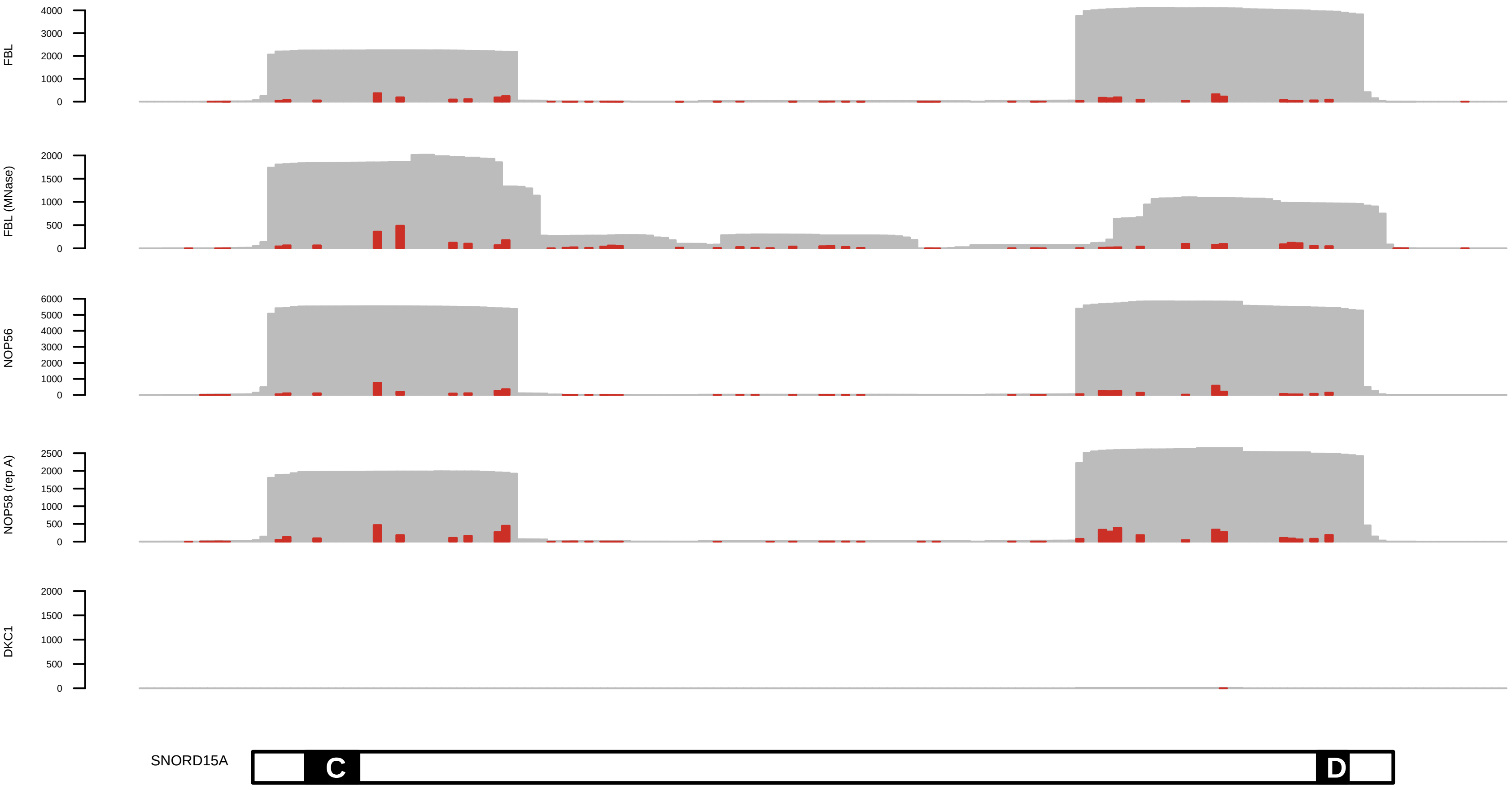
FBL
4000
3000
2000
1000
0
FBL (MNase)
2000
1500
1000
500
0
NOP56
6000
5000
4000
3000
2000
1000
0
NOP58 (rep A)
2500
2000
1500
1000
500
0
DKC1
2000
1500
1000
500
0
SNORD15A
C
D

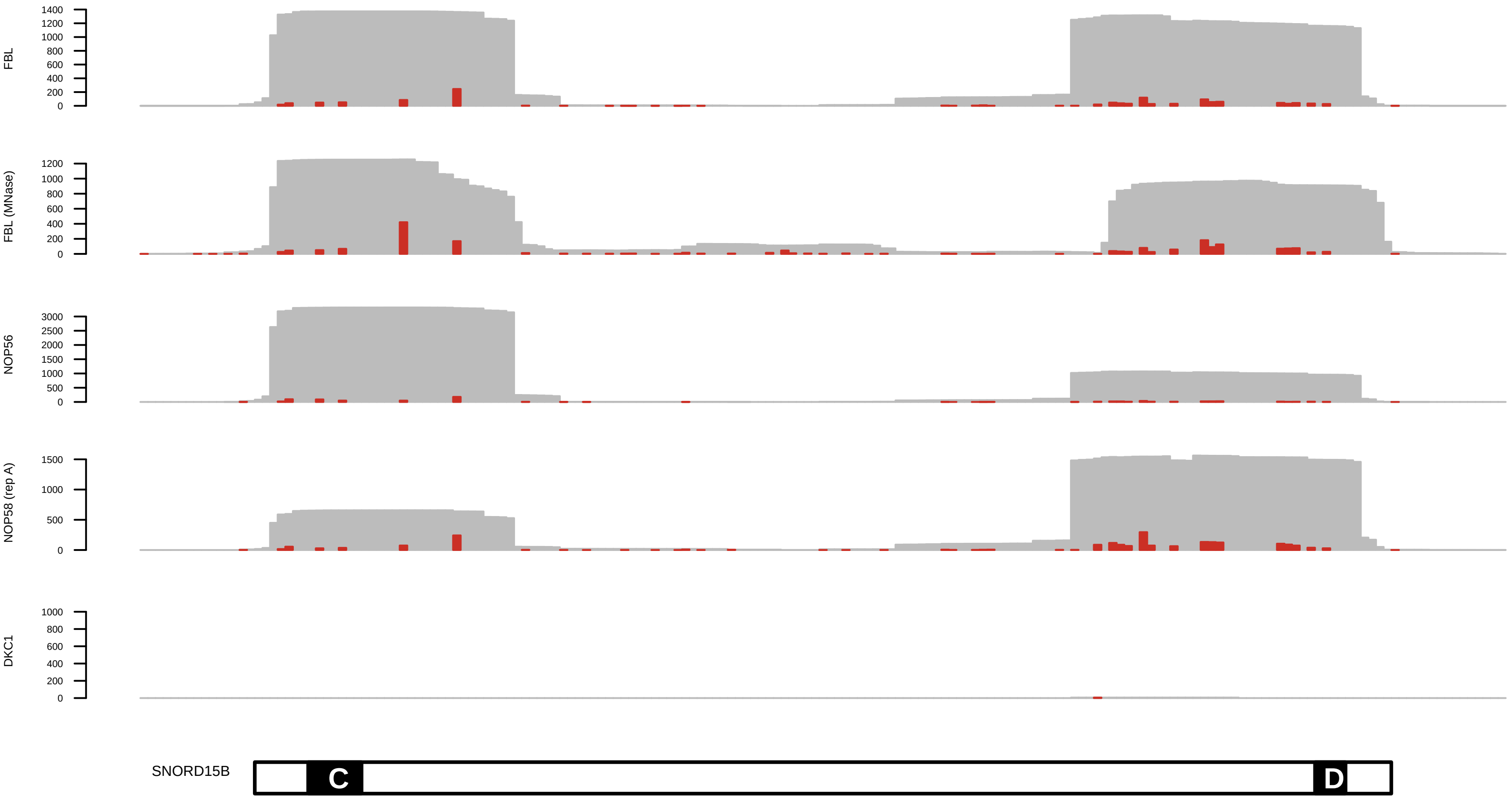

FBL
1400
1200
1000
800
600
400
200
0
FBL (MNase)
1200
1000
800
600
400
200
0
NOP56
3000
2500
2000
1500
1000
500
0
NOP58 (rep A)
1500
1000
500
0
DKC1
1000
800
600
400
200
0
SNORD15B
C
D

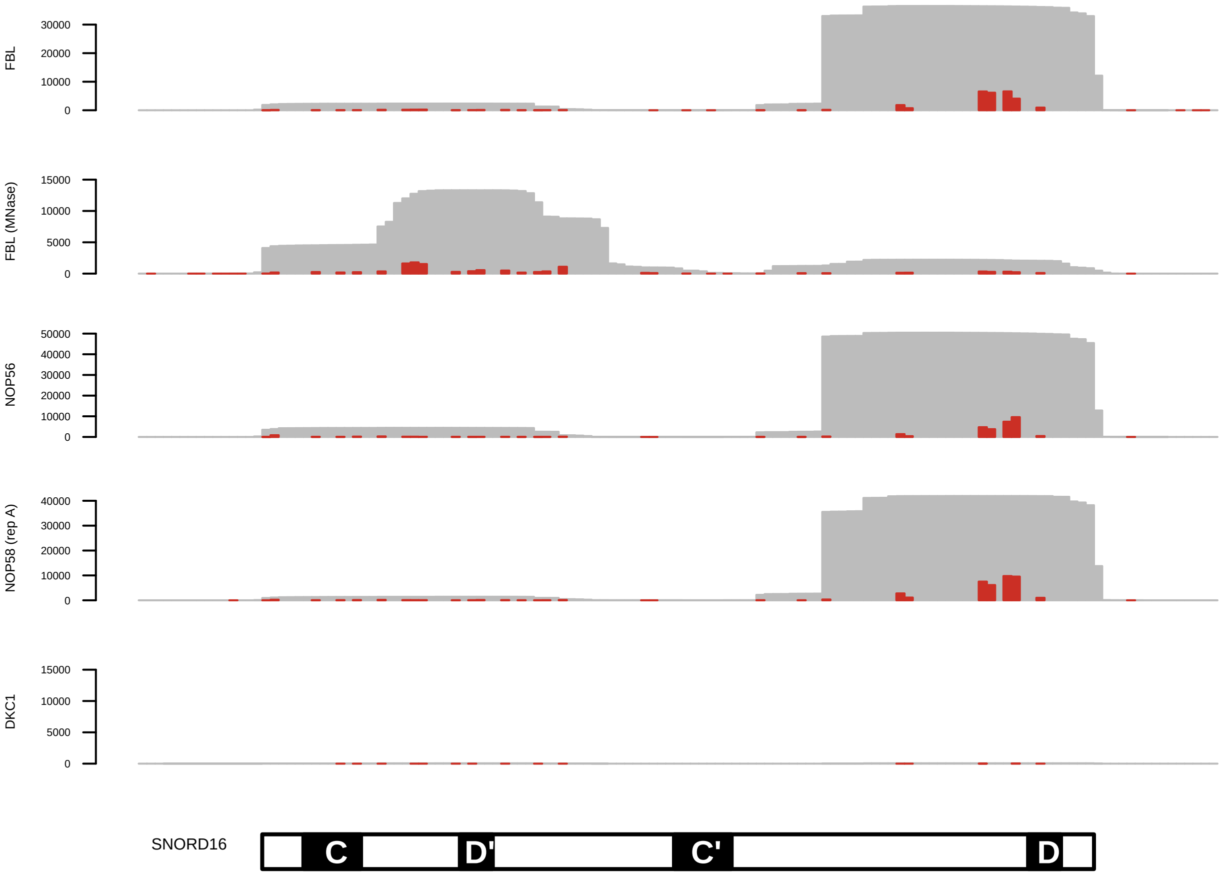

FBL
30000
20000
10000
0
FBL (MNase)
15000
10000
5000
0
NOP56
50000
40000
30000
20000
10000
0
NOP58 (rep A)
40000
30000
20000
10000
0
DKC1
15000
10000
5000
0
SNORD16
C
D'
C'
D

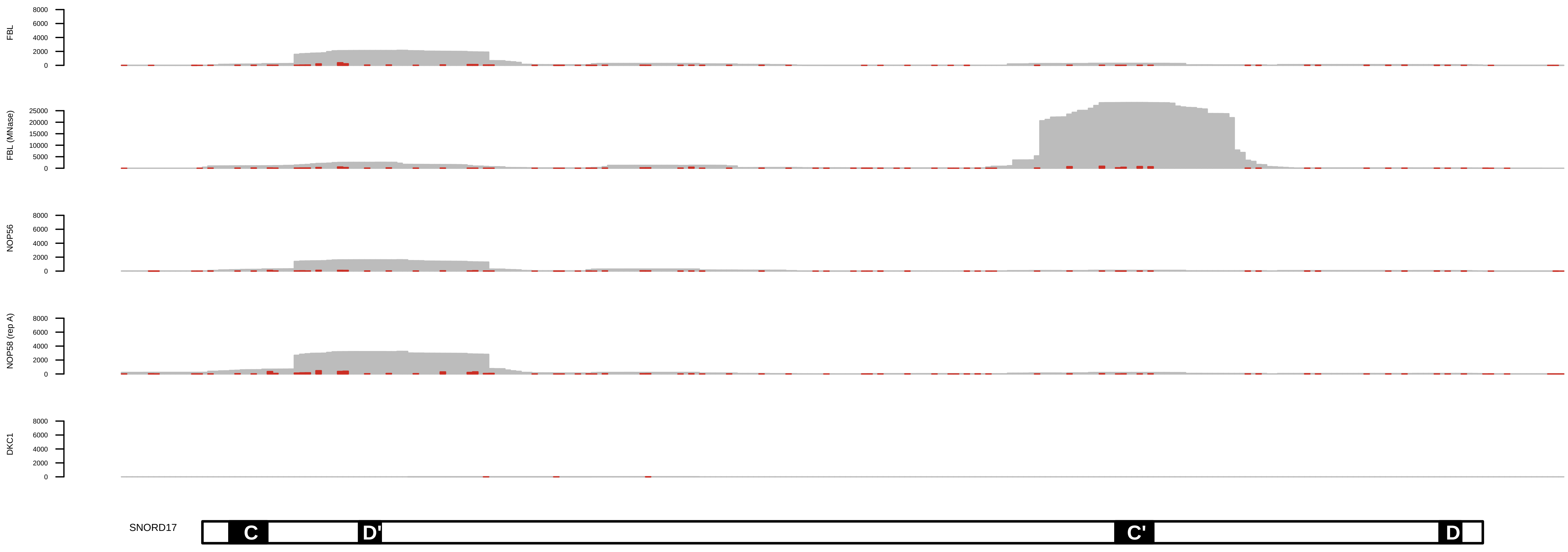
FBL
8000
6000
4000
2000
0
FBL (MNase)
25000
20000
15000
10000
5000
0
NOP56
8000
6000
4000
2000
0
NOP58 (rep A)
8000
6000
4000
2000
0
DKC1
8000
6000
4000
2000
0
SNORD17
C
D'
C'
D

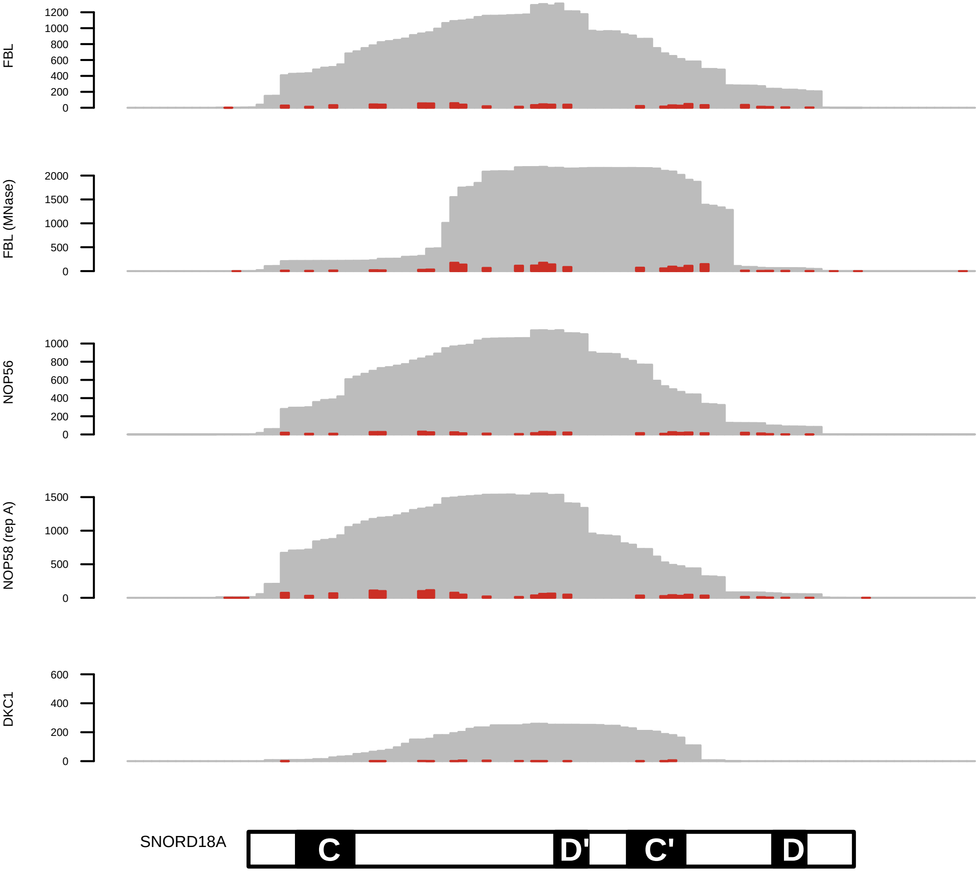
FBL
1200
1000
800
600
400
200
0
FBL (MNase)
2000
1500
1000
500
0
NOP56
1000
800
600
400
200
0
NOP58 (rep A)
1500
1000
500
0
DKC1
600
400
200
0
SNORD18A
C
D'
C'
D

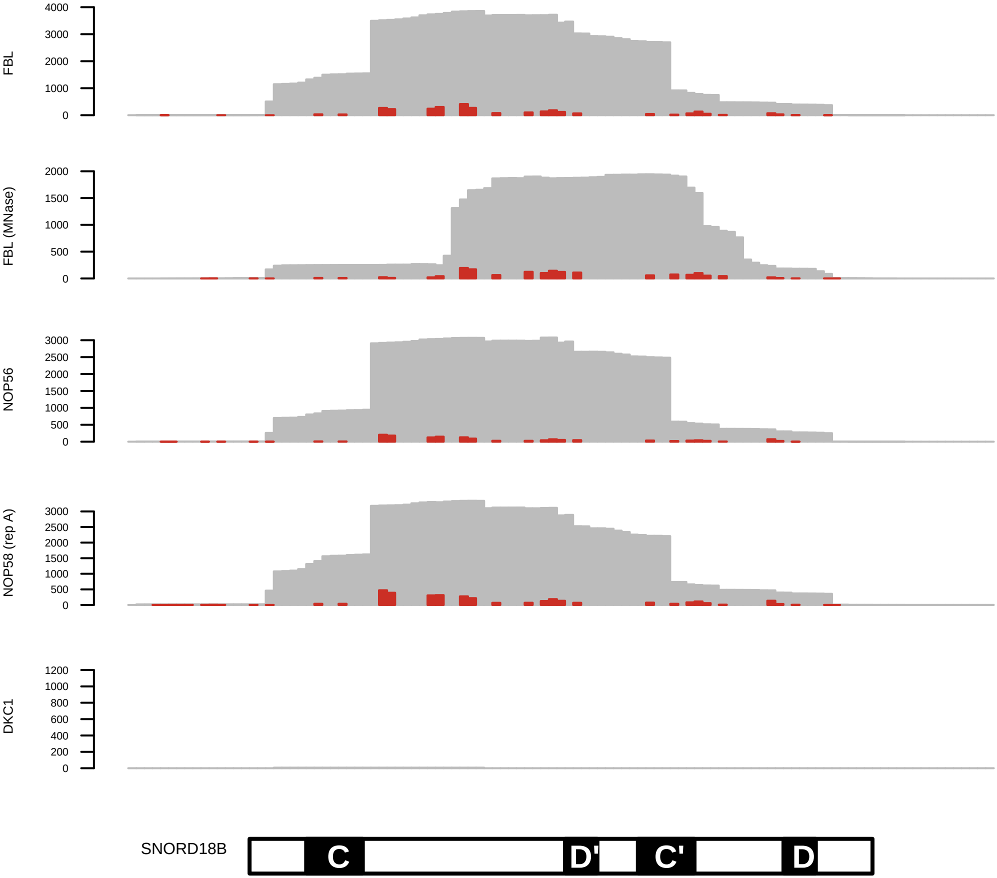

FBL
4000
3000
2000
1000
0
FBL (MNase)
2000
1500
1000
500
0
NOP56
3000
2500
2000
1500
1000
500
0
NOP58 (rep A)
3000
2500
2000
1500
1000
500
0
DKC1
1200
1000
800
600
400
200
0
SNORD18B
C
D'
C'
D

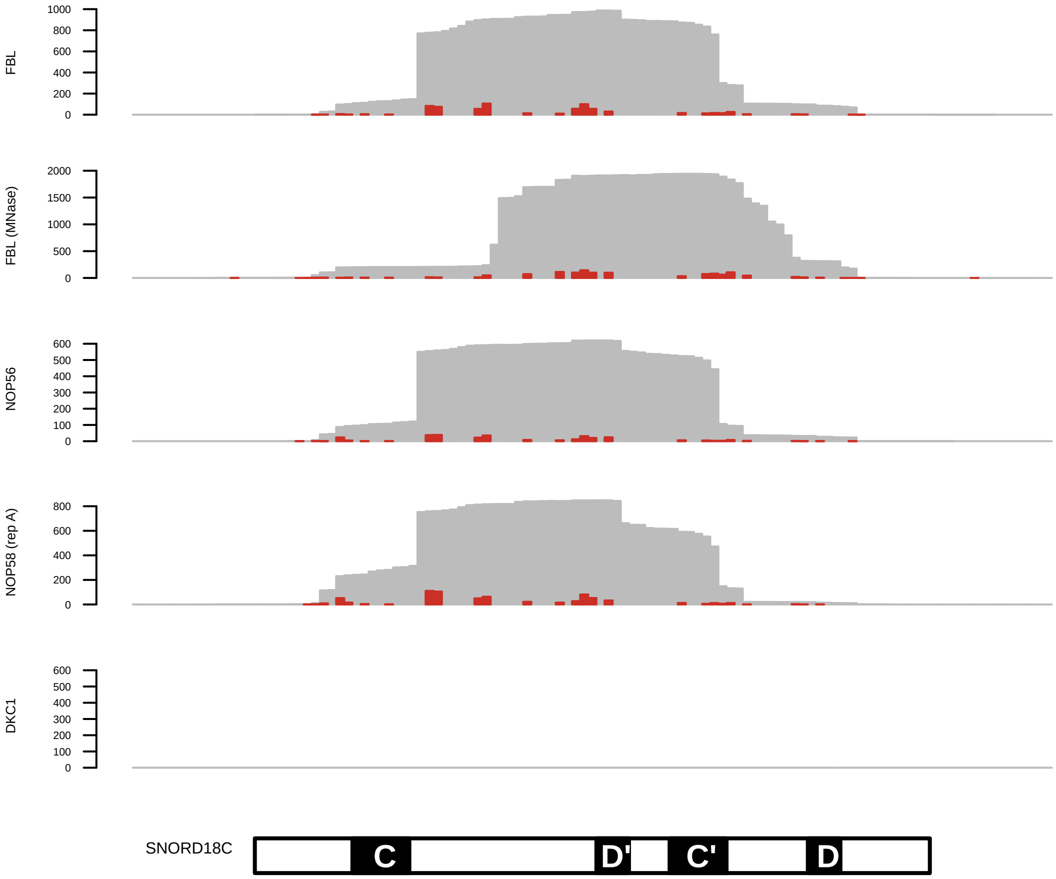

FBL
1000
800
600
400
200
0
FBL (MNase)
2000
1500
1000
500
0
NOP56
600
500
400
300
200
100
0
NOP58 (rep A)
800
600
400
200
0
DKC1
600
500
400
300
200
100
0
SNORD18C
C
D'
C'
D

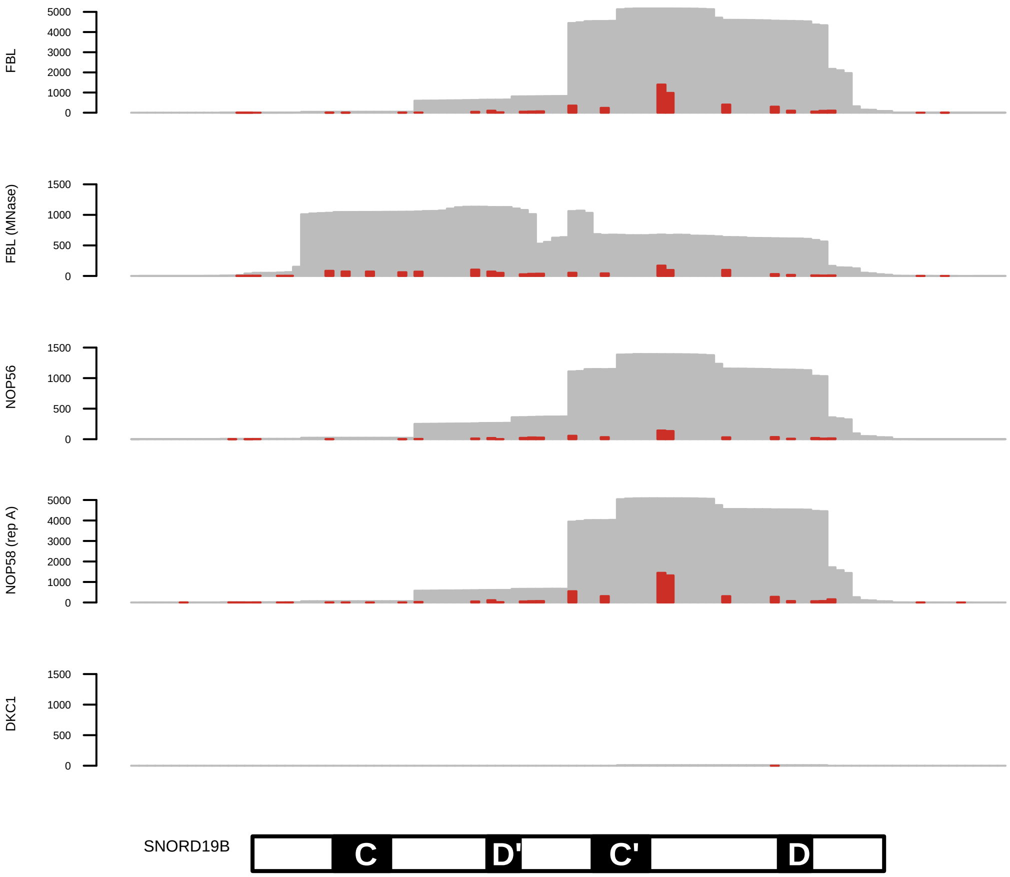

FBL
5000
4000
3000
2000
1000
0
FBL (MNase)
1500
1000
500
0
NOP56
1500
1000
500
0
NOP58 (rep A)
5000
4000
3000
2000
1000
0
DKC1
1500
1000
500
0
SNORD19B
C
D'
C'
D

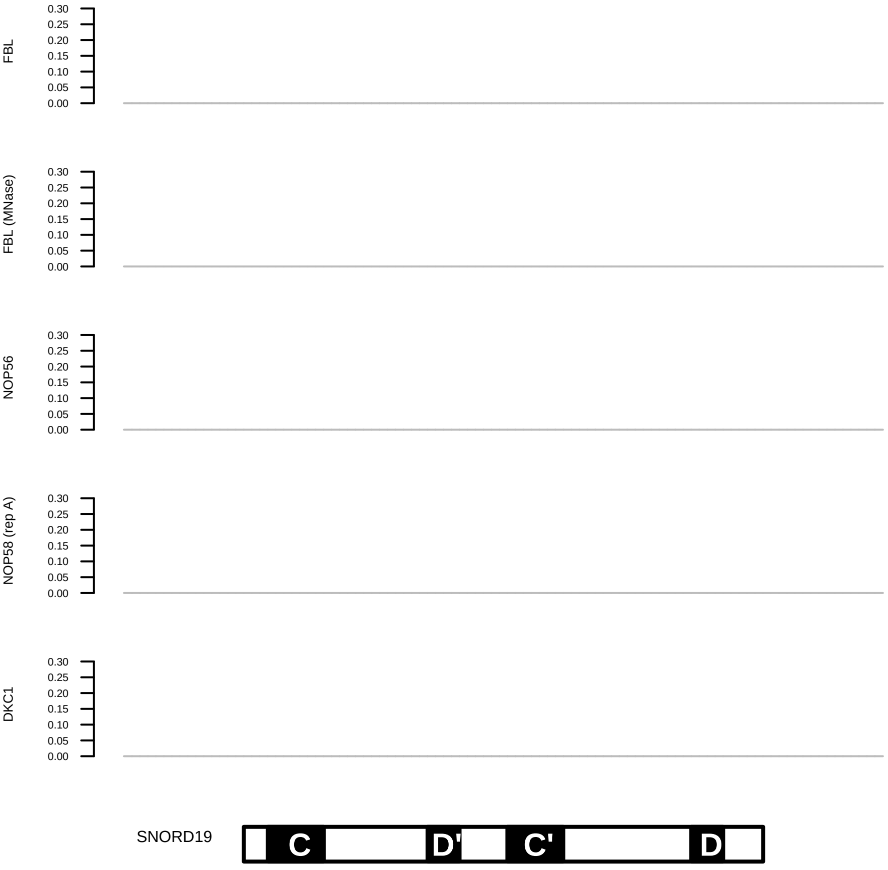

FBL
FBL (MNase)
NOP56
NOP58 (rep A)
DKC1
0.30
0.25
0.20
0.15
0.10
0.05
0.00
SNORD19
C
D'
C'
D

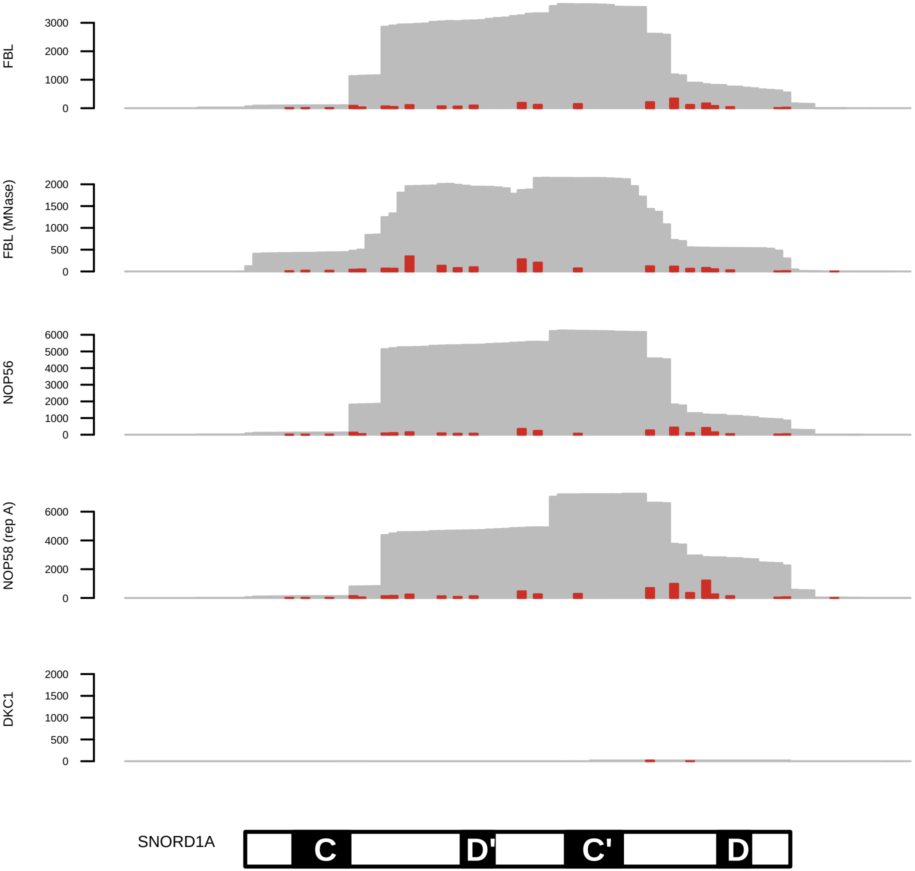
FBL
3000
2000
1000
0
FBL (MNase)
2000
1500
1000
500
0
NOP56
6000
5000
4000
3000
2000
1000
0
NOP58 (rep A)
6000
4000
2000
0
DKC1
2000
1500
1000
500
0
SNORD1A
C
D'
C'
D

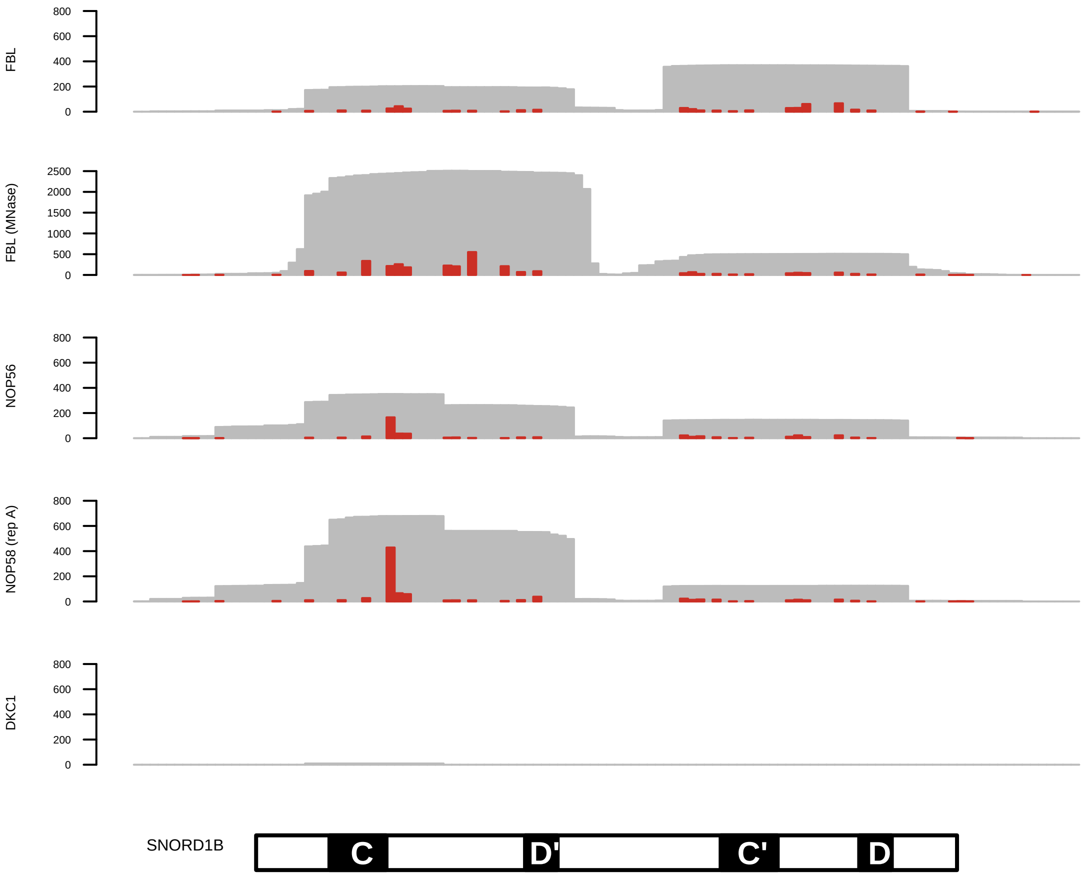

FBL
800
600
400
200
0
FBL (MNase)
2500
2000
1500
1000
500
0
NOP56
800
600
400
200
0
NOP58 (rep A)
800
600
400
200
0
DKC1
800
600
400
200
0
SNORD1B
C
D'
C'
D

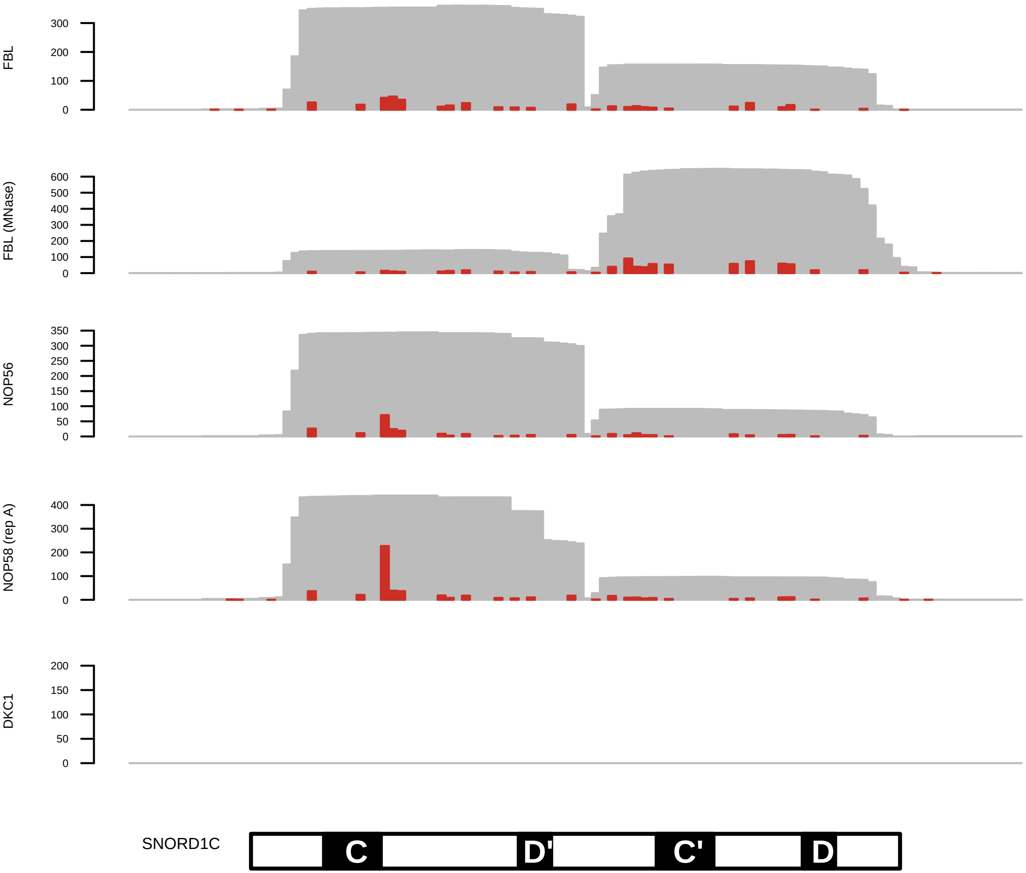
FBL
300
200
100
0
FBL (MNase)
600
500
400
300
200
100
0
NOP56
350
300
250
200
150
100
50
0
NOP58 (rep A)
400
300
200
100
0
DKC1
200
150
100
50
0
SNORD1C
C
D'
C'
D

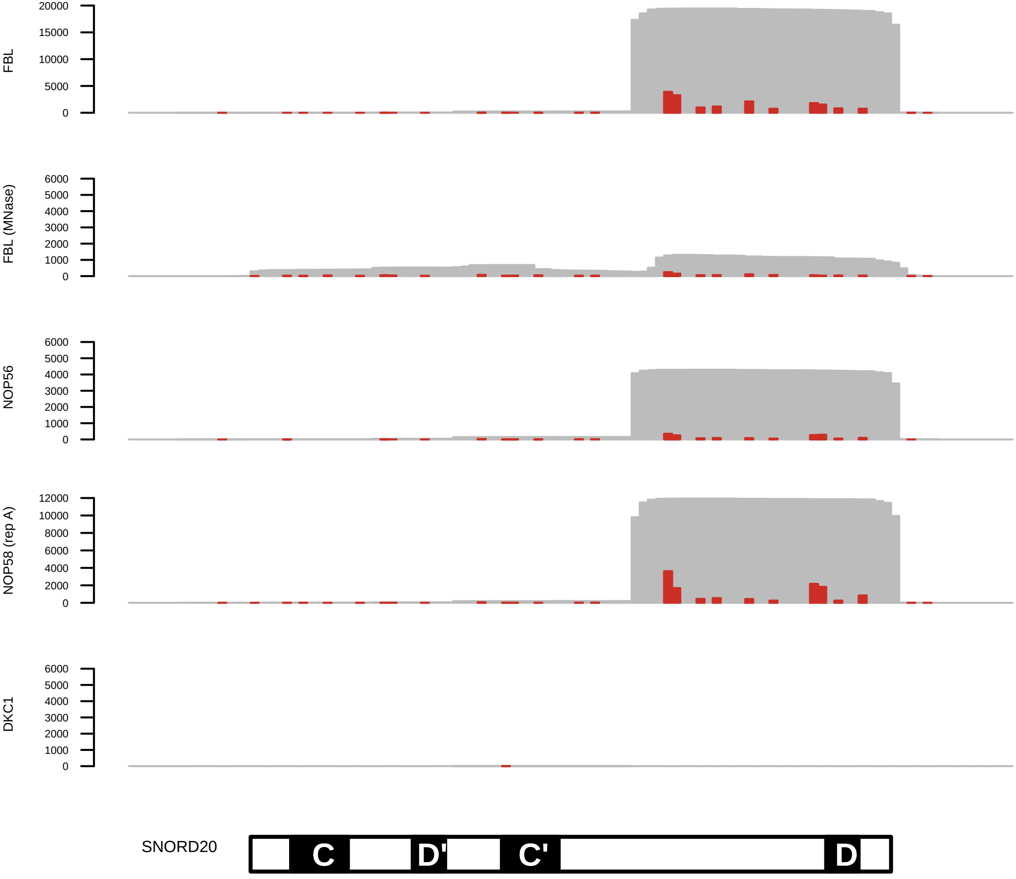

FBL
20000
15000
10000
5000
0
FBL (MNase)
6000
5000
4000
3000
2000
1000
0
NOP56
6000
5000
4000
3000
2000
1000
0
NOP58 (rep A)
12000
10000
8000
6000
4000
2000
0
DKC1
6000
5000
4000
3000
2000
1000
0
SNORD20
C
D'
C'
D

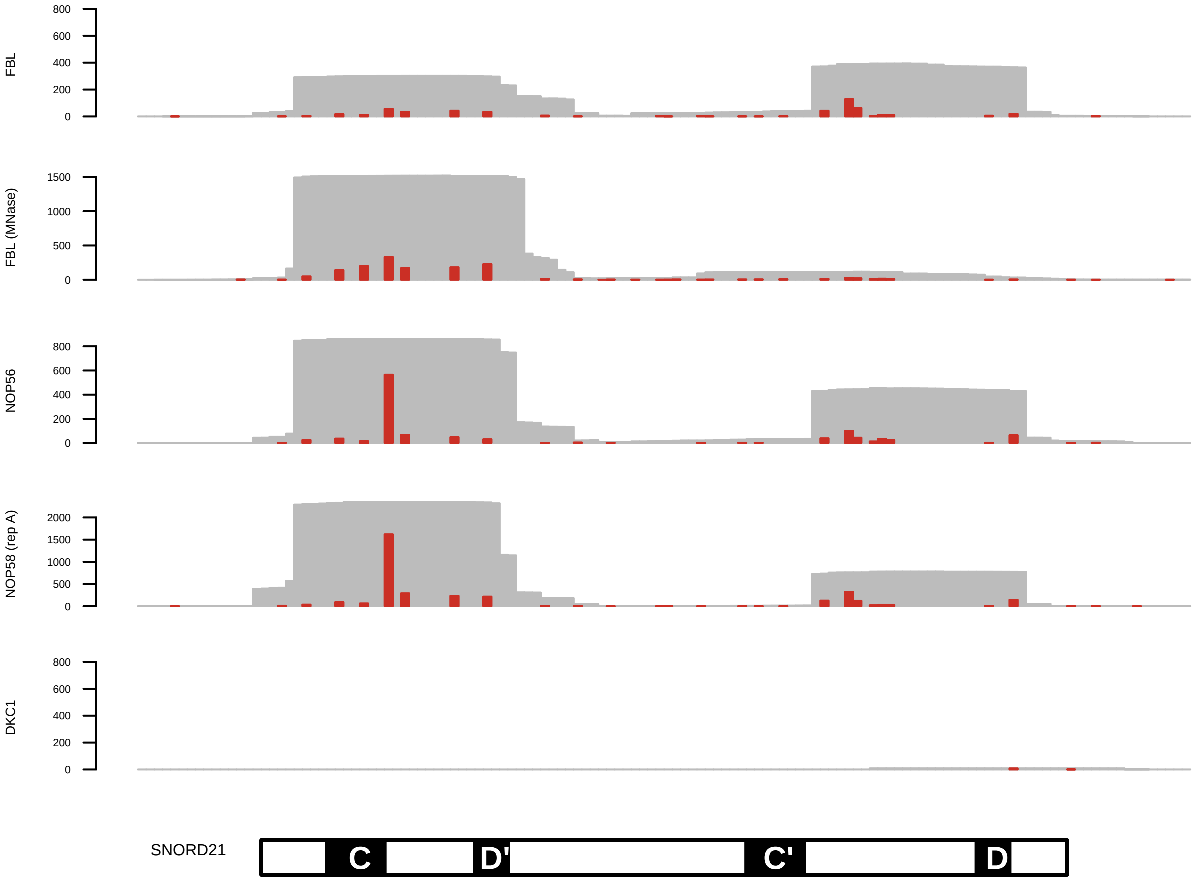

FBL
800
600
400
200
0
FBL (MNase)
1500
1000
500
0
NOP56
800
600
400
200
0
NOP58 (rep A)
2000
1500
1000
500
0
DKC1
800
600
400
200
0
SNORD21
C
D'
C'
D

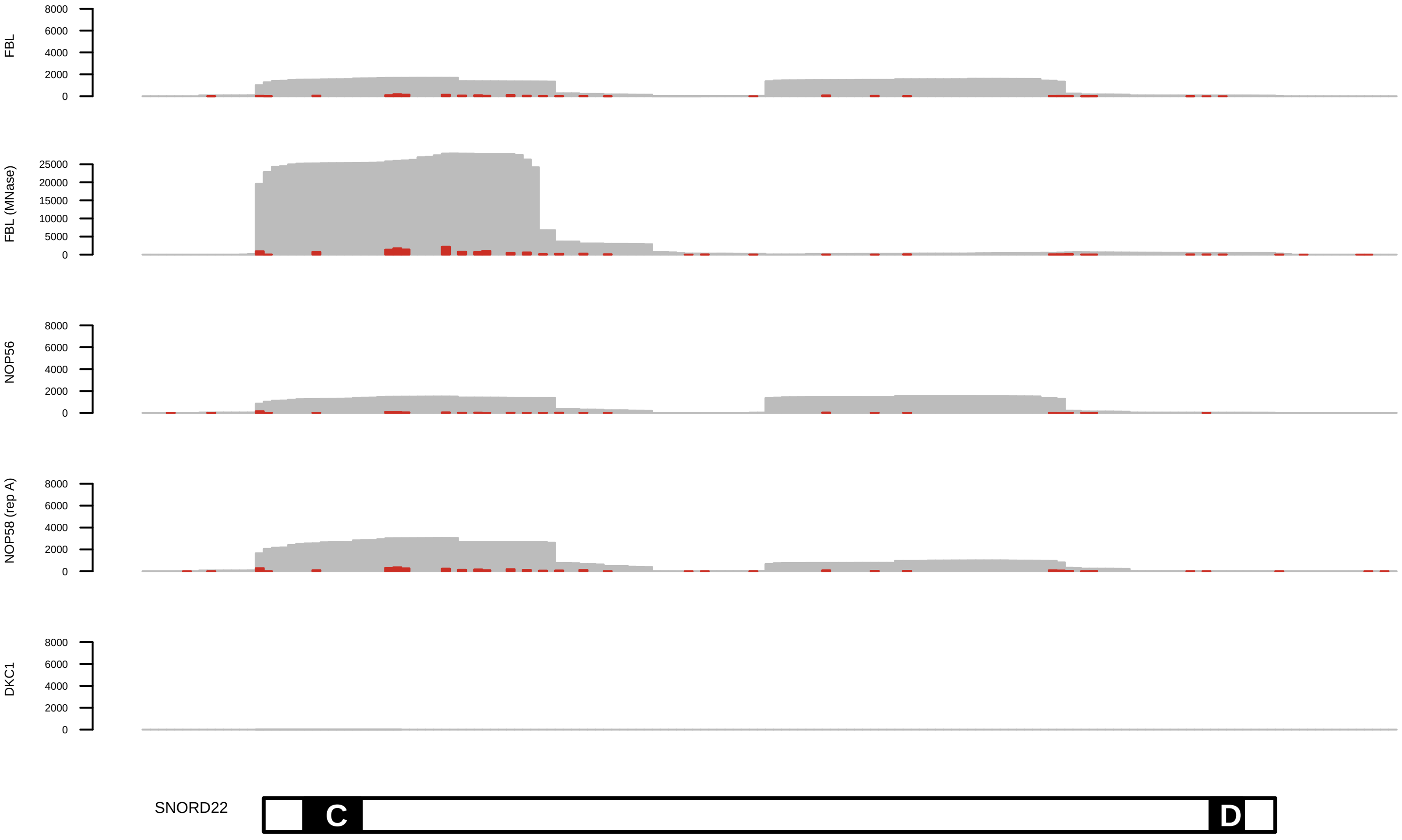
FBL
8000
6000
4000
2000
0
FBL (MNase)
25000
20000
15000
10000
5000
0
NOP56
8000
6000
4000
2000
0
NOP58 (rep A)
8000
6000
4000
2000
0
DKC1
8000
6000
4000
2000
0
SNORD22
C
D

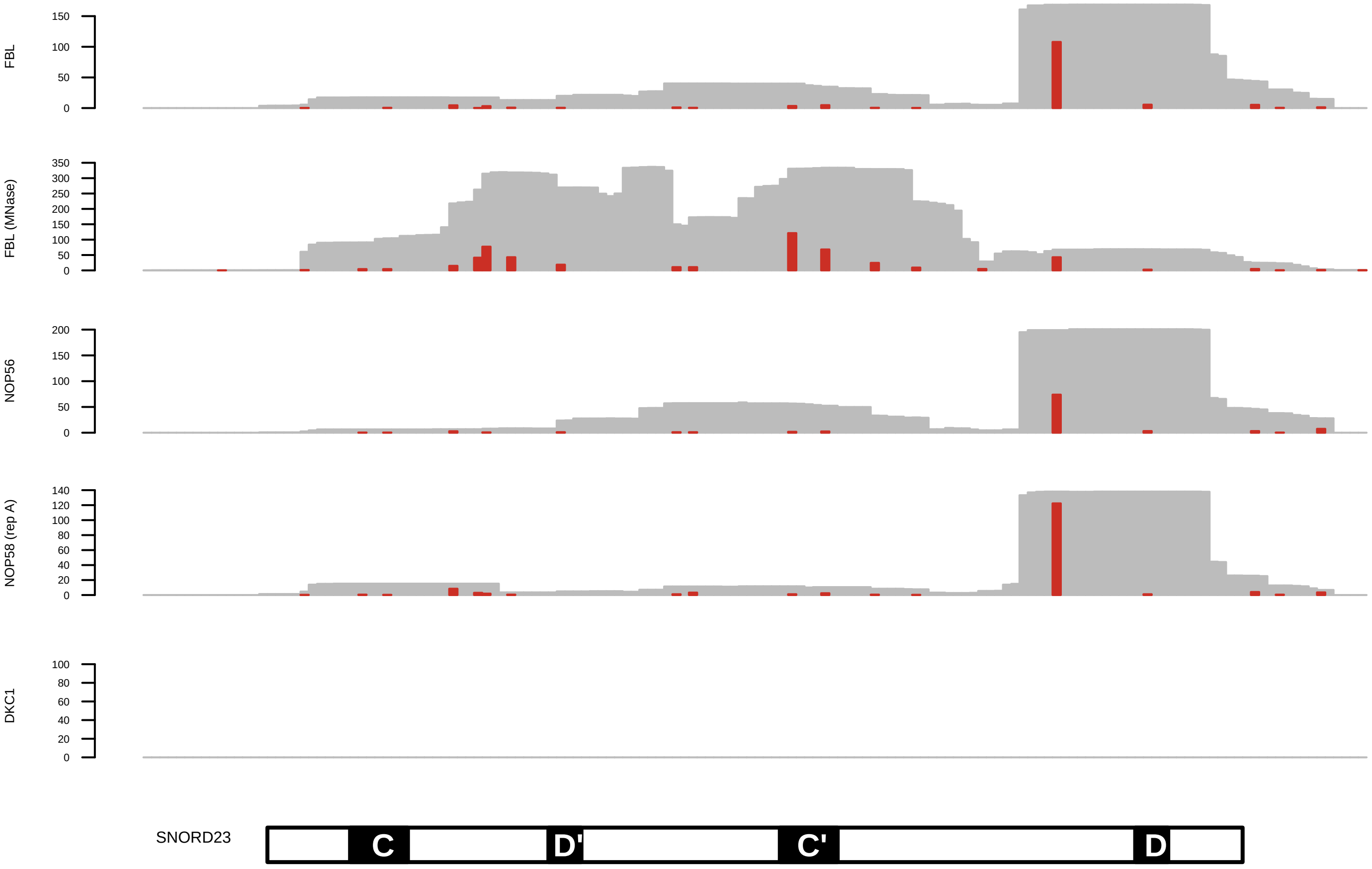
FBL
150
100
50
0
FBL (MNase)
350
300
250
200
150
100
50
0
NOP56
200
150
100
50
0
NOP58 (rep A)
140
120
100
80
60
40
20
0
DKC1
100
80
60
40
20
0
SNORD23
C
D'
C'
D

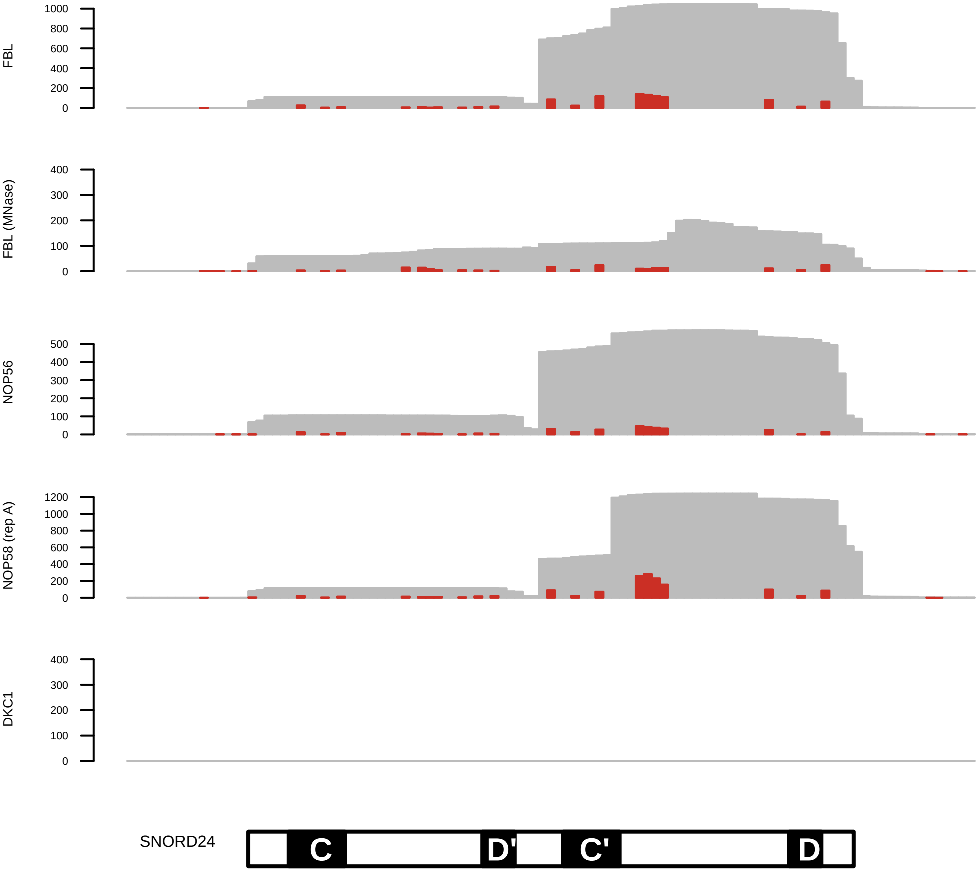
FBL
1000
800
600
400
200
0
FBL (MNase)
400
300
200
100
0
NOP56
500
400
300
200
100
0
NOP58 (rep A)
1200
1000
800
600
400
200
0
DKC1
400
300
200
100
0
SNORD24
C
D'
C'
D

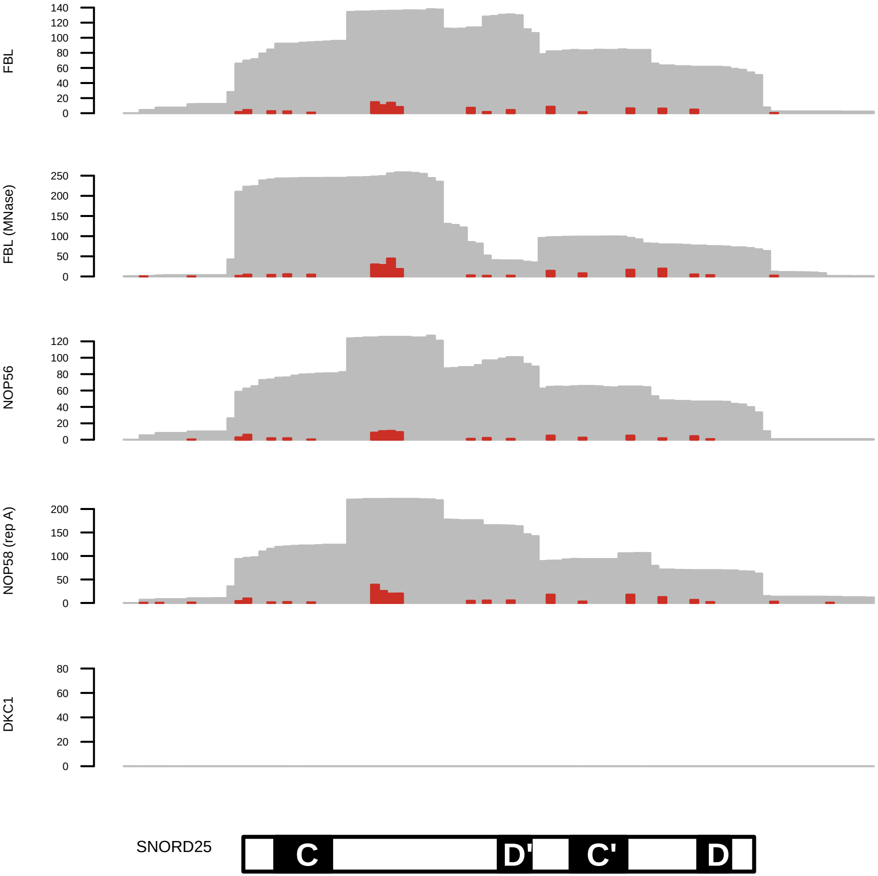
FBL
FBL (MNase)
NOP56
NOP58 (rep A)
DKC1
0
20
40
60
80
100
120
140
50
150
200
250
SNORD25
C
D'
C'
D

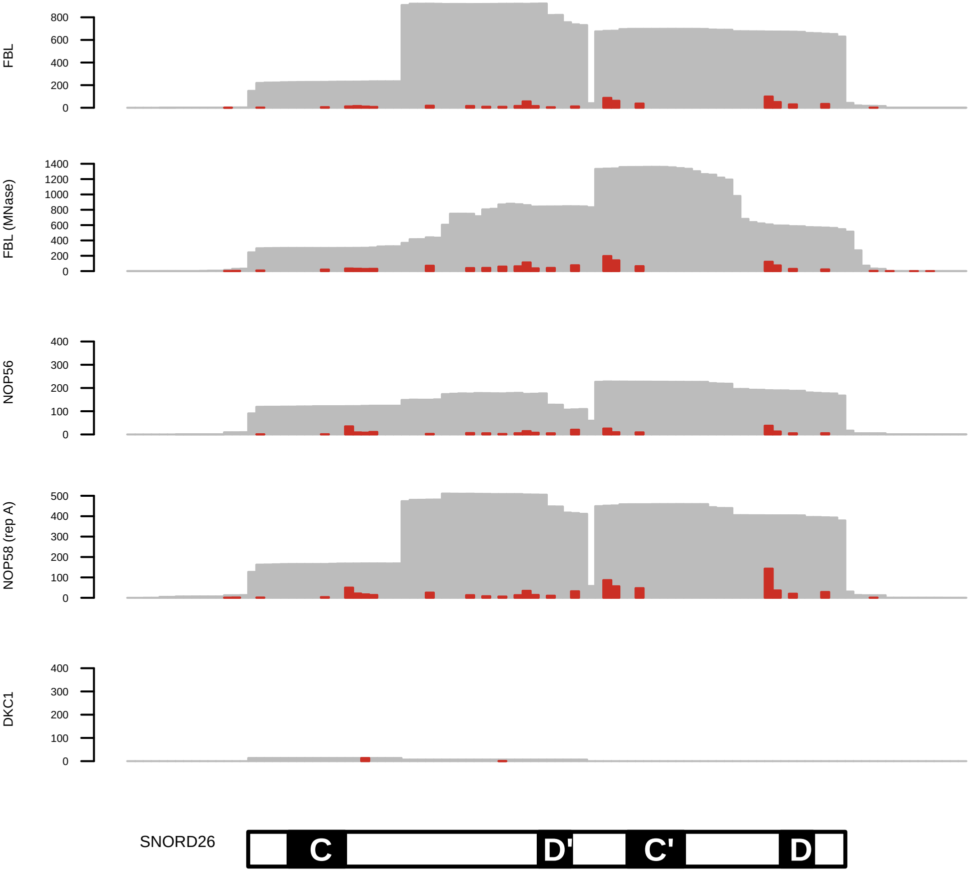

FBL
800
600
400
200
0
FBL (MNase)
1400
1200
1000
800
600
400
200
0
NOP56
400
300
200
100
0
NOP58 (rep A)
500
400
300
200
100
0
DKC1
400
300
200
100
0
SNORD26
C
D'
C'
D

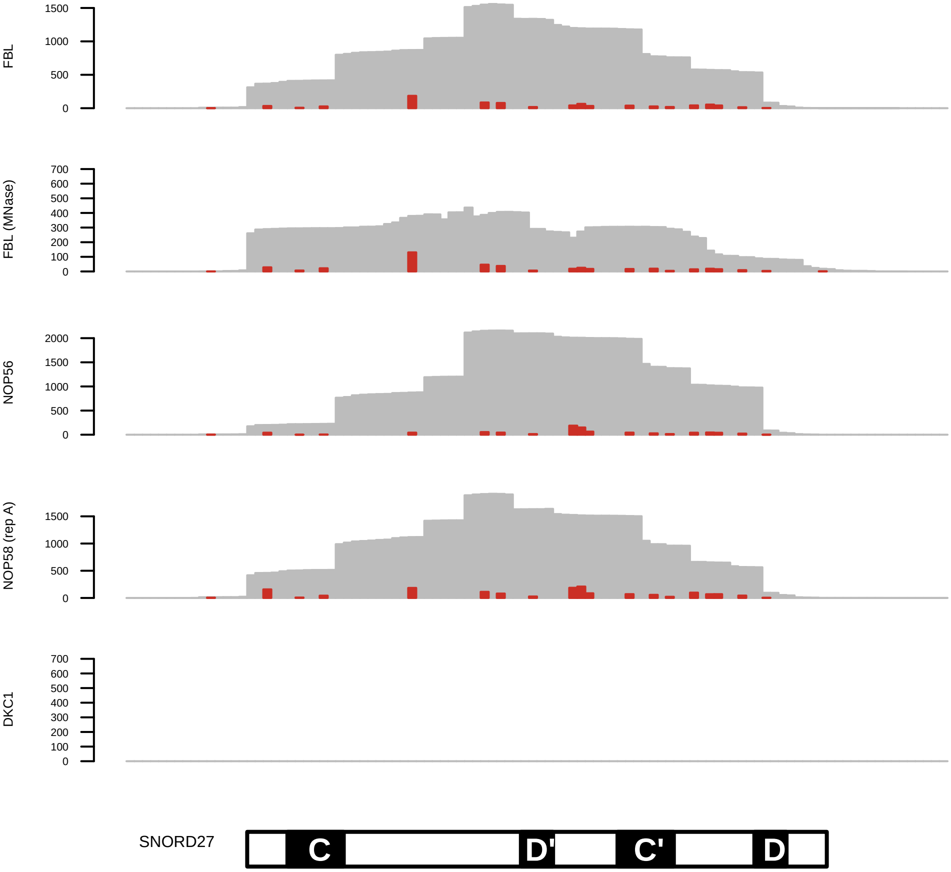
FBL
1500
1000
500
0
FBL (MNase)
700
600
500
400
300
200
100
0
NOP56
2000
1500
1000
500
0
NOP58 (rep A)
1500
1000
500
0
DKC1
700
600
500
400
300
200
100
0
SNORD27
C
D'
C'
D

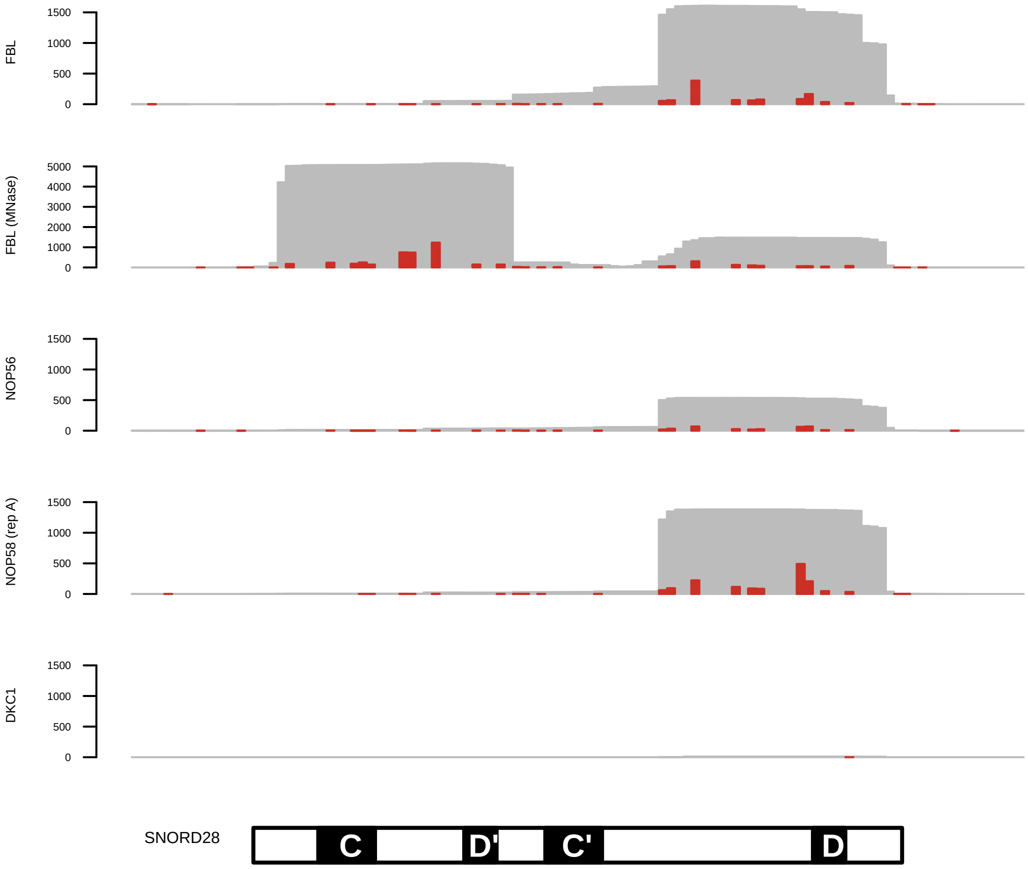
FBL
FBL (MNase)
NOP56
NOP58 (rep A)
DKC1
0
500
1000
1500
2000
3000
4000
5000
SNORD28
C
D'
C'
D

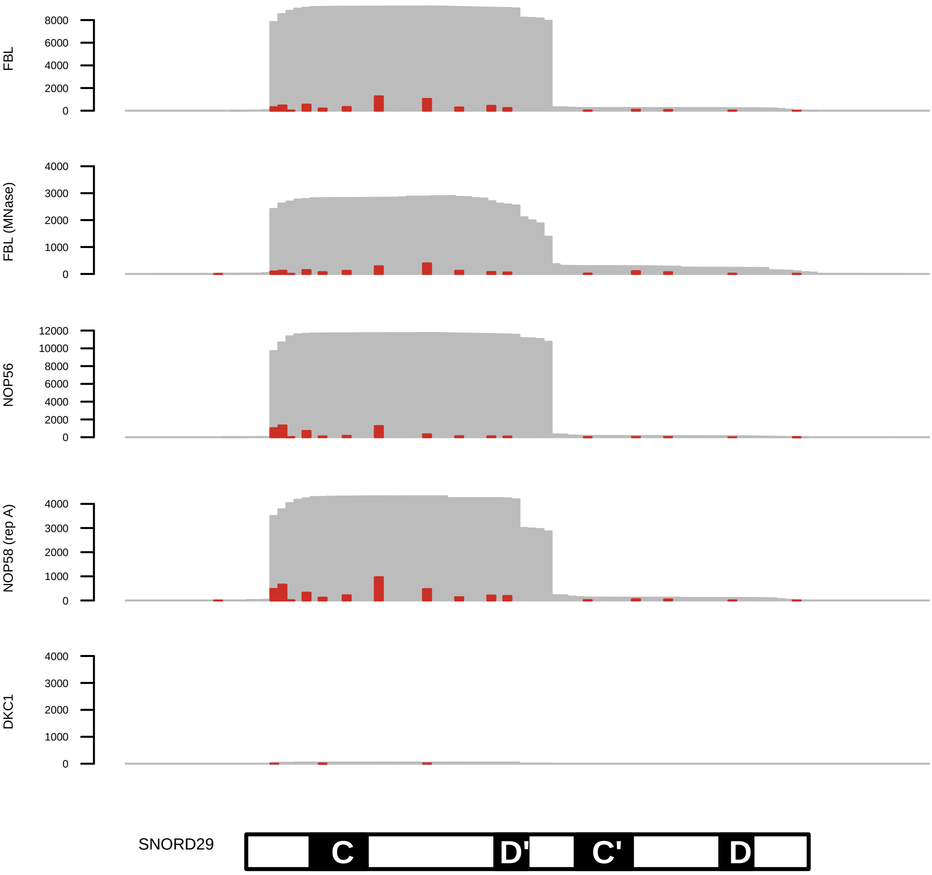
FBL
8000
6000
4000
2000
0
FBL (MNase)
4000
3000
2000
1000
0
NOP56
12000
10000
8000
6000
4000
2000
0
NOP58 (rep A)
4000
3000
2000
1000
0
DKC1
4000
3000
2000
1000
0
SNORD29
C
D'
C'
D

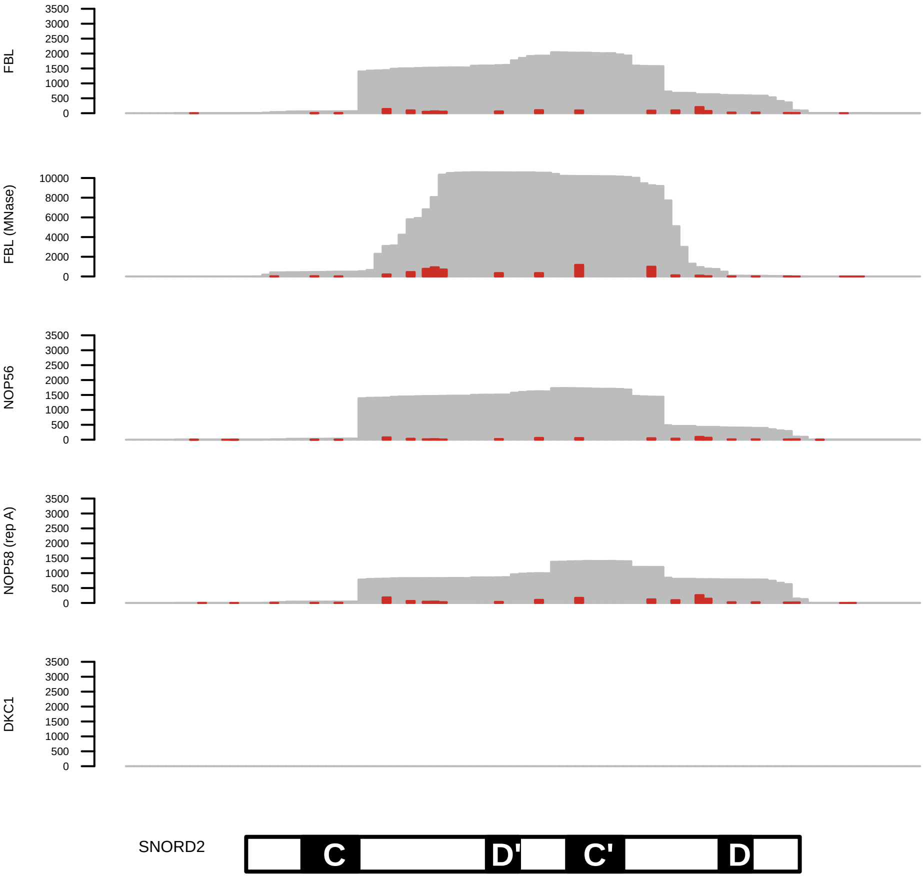
FBL
3500
3000
2500
2000
1500
1000
500
0
FBL (MNase)
10000
8000
6000
4000
2000
0
NOP56
3500
3000
2500
2000
1500
1000
500
0
NOP58 (rep A)
3500
3000
2500
2000
1500
1000
500
0
DKC1
3500
3000
2500
2000
1500
1000
500
0
SNORD2
C
D'
C'
D

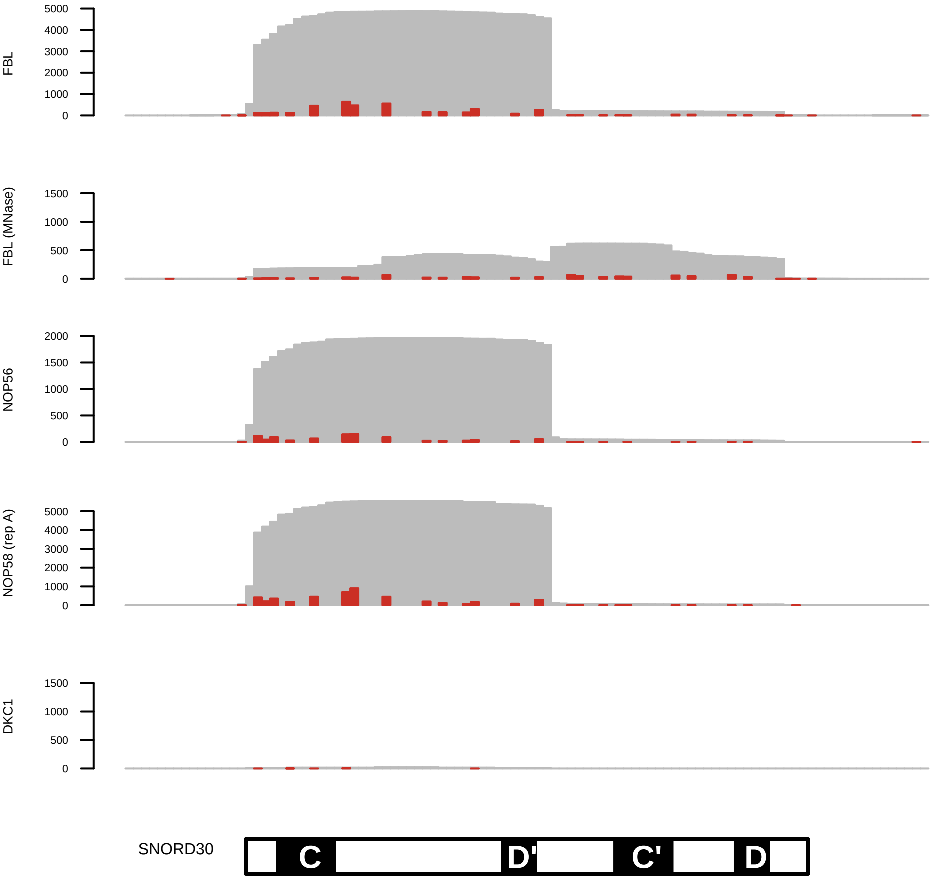
FBL
5000
4000
3000
2000
1000
0
FBL (MNase)
1500
1000
500
0
NOP56
2000
1500
1000
500
0
NOP58 (rep A)
5000
4000
3000
2000
1000
0
DKC1
1500
1000
500
0
SNORD30
C
D'
C'
D

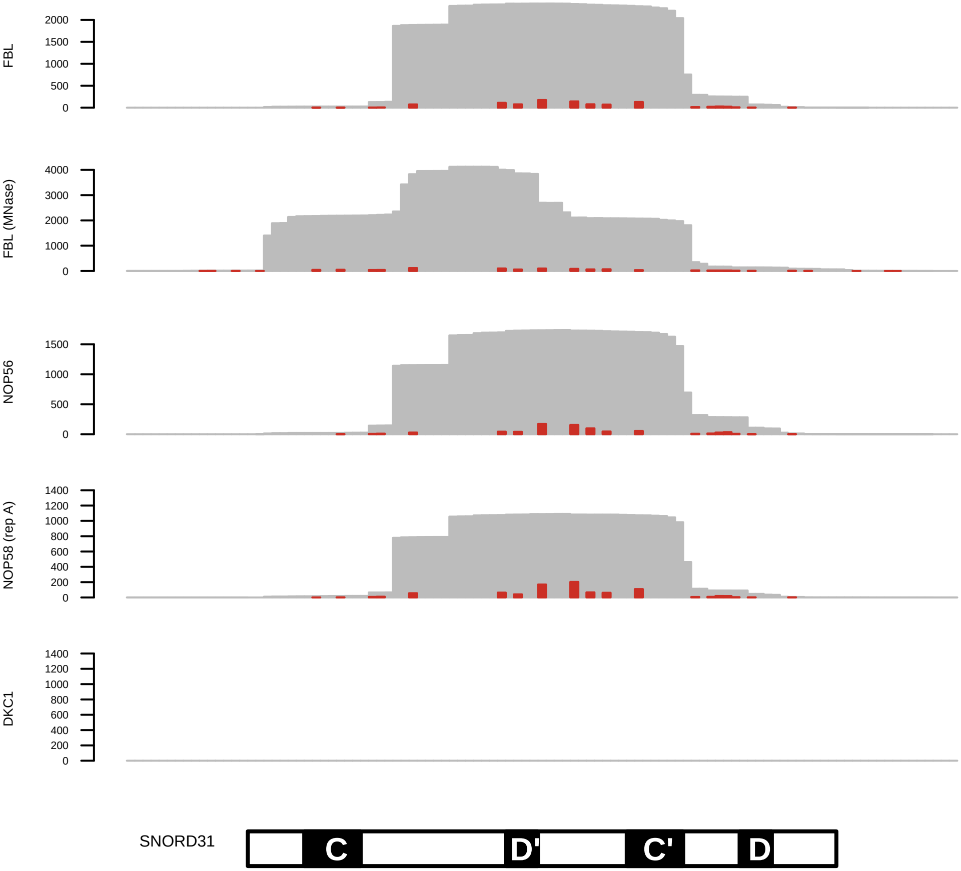
FBL
2000
1500
1000
500
0
FBL (MNase)
4000
3000
2000
1000
0
NOP56
1500
1000
500
0
NOP58 (rep A)
1400
1200
1000
800
600
400
200
0
DKC1
1400
1200
1000
800
600
400
200
0
SNORD31
C
D'
C'
D

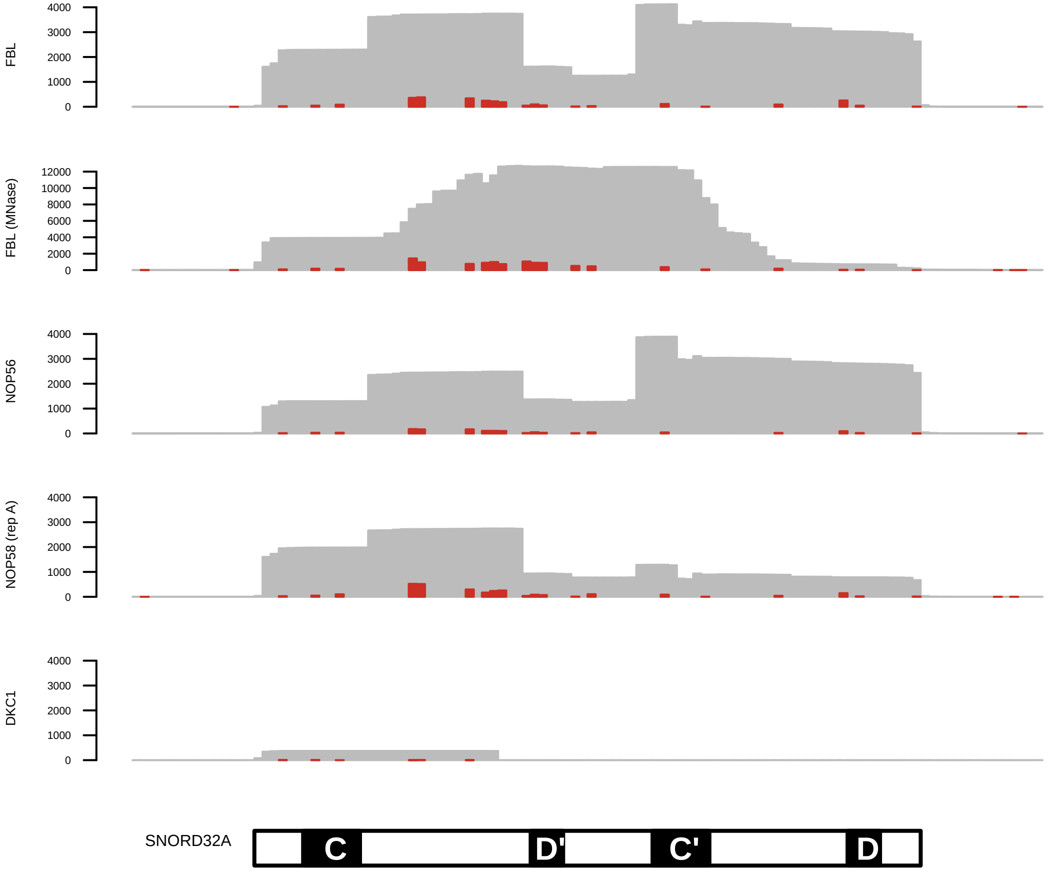

FBL
0
1000
2000
3000
4000
FBL (MNase)
0
2000
4000
6000
8000
10000
12000
NOP56
0
1000
2000
3000
4000
NOP58 (rep A)
0
1000
2000
3000
4000
DKC1
0
1000
2000
3000
4000
SNORD32A
C
D'
C'
D

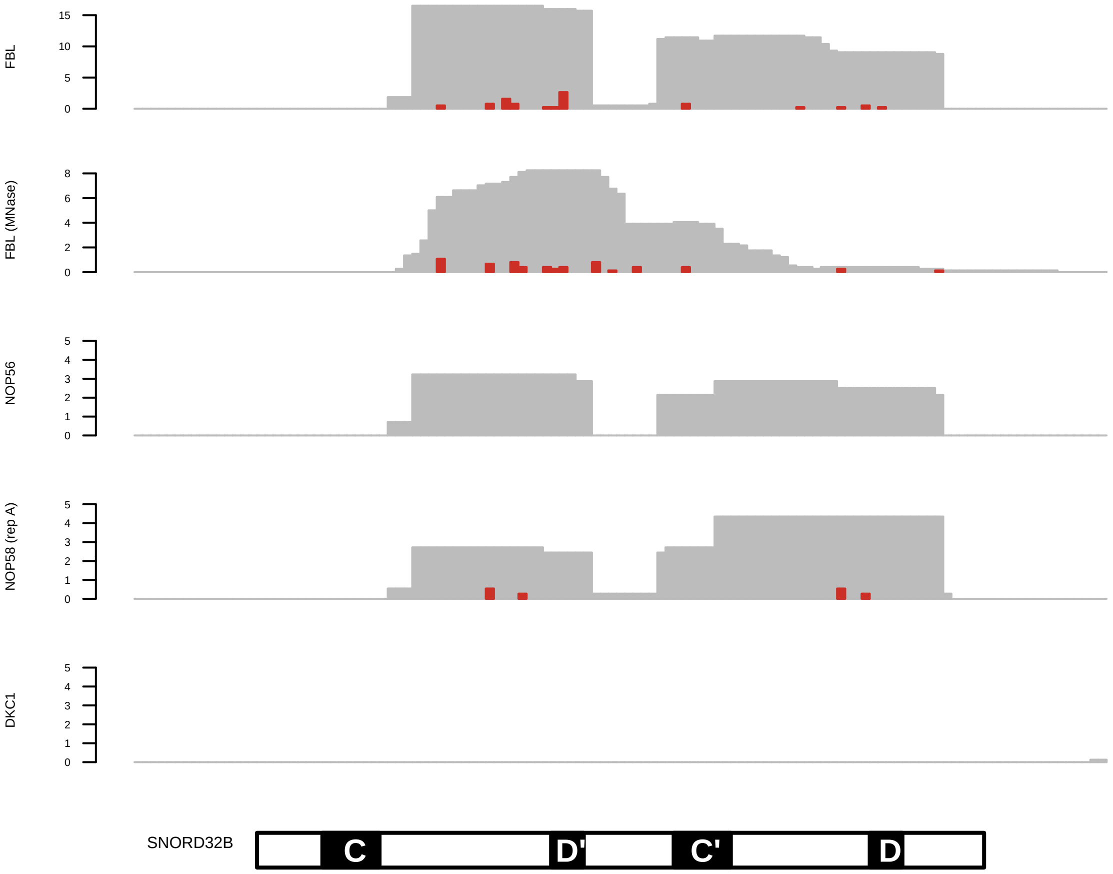

FBL
15
10
5
0
FBL (MNase)
8
6
4
2
0
NOP56
5
4
3
2
1
0
NOP58 (rep A)
5
4
3
2
1
0
DKC1
5
4
3
2
1
0
SNORD32B
C
D'
C'
D

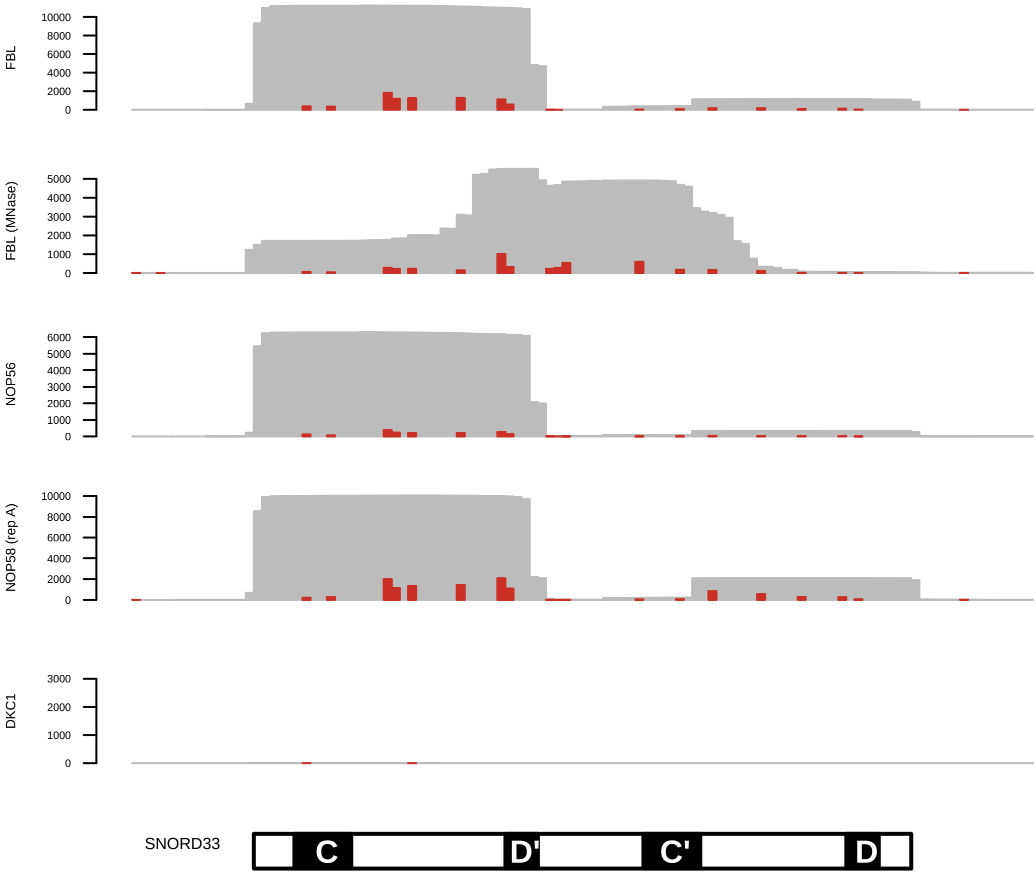

FBL
10000
8000
6000
4000
2000
0
FBL (MNase)
5000
4000
3000
2000
1000
0
NOP56
6000
5000
4000
3000
2000
1000
0
NOP58 (rep A)
10000
8000
6000
4000
2000
0
DKC1
3000
2000
1000
0
SNORD33
C
D'
C'
D

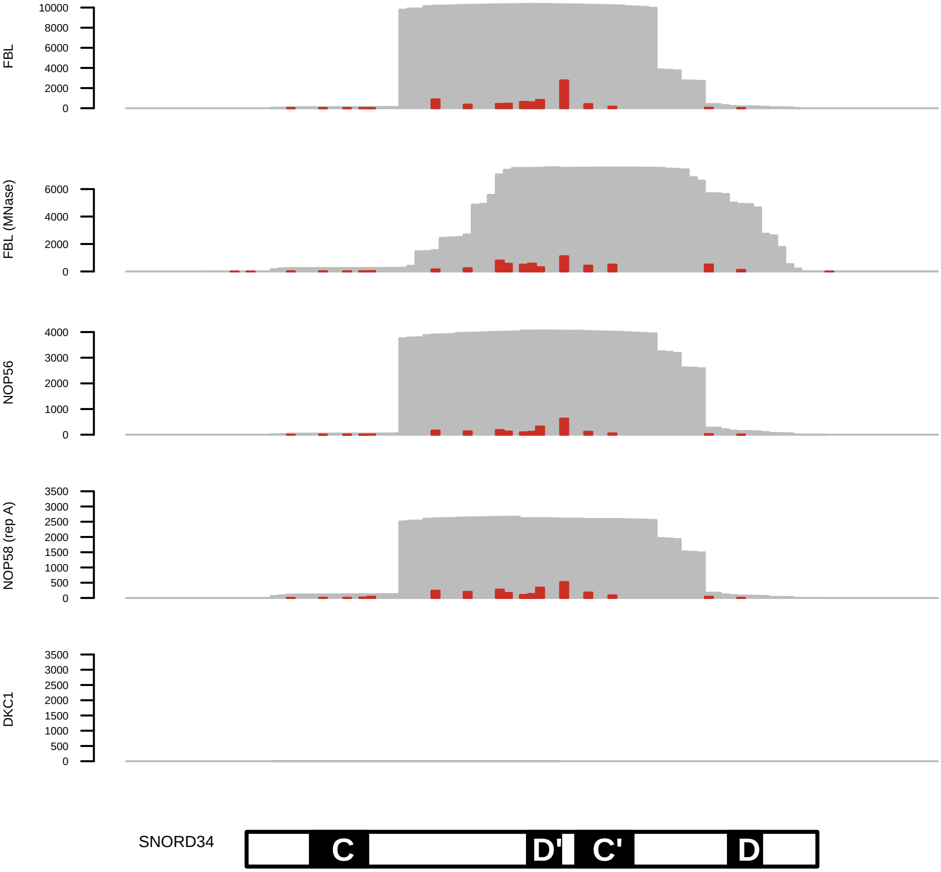

FBL
10000
8000
6000
4000
2000
0
FBL (MNase)
6000
4000
2000
0
NOP56
4000
3000
2000
1000
0
NOP58 (rep A)
3500
3000
2500
2000
1500
1000
500
0
DKC1
3500
3000
2500
2000
1500
1000
500
0
SNORD34
C
D'
C'
D

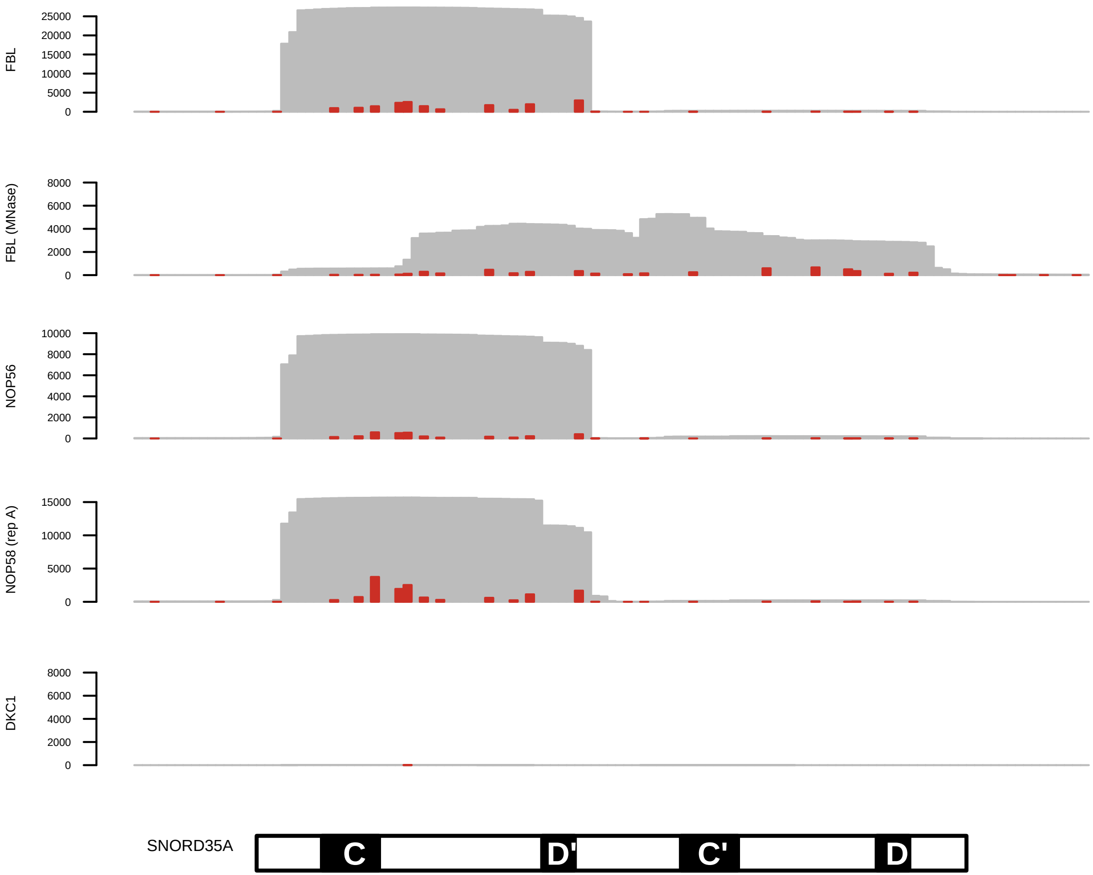
FBL
25000
20000
15000
10000
5000
0
FBL (MNase)
8000
6000
4000
2000
0
NOP56
10000
8000
6000
4000
2000
0
NOP58 (rep A)
15000
10000
5000
0
DKC1
8000
6000
4000
2000
0
SNORD35A
C
D'
C'
D

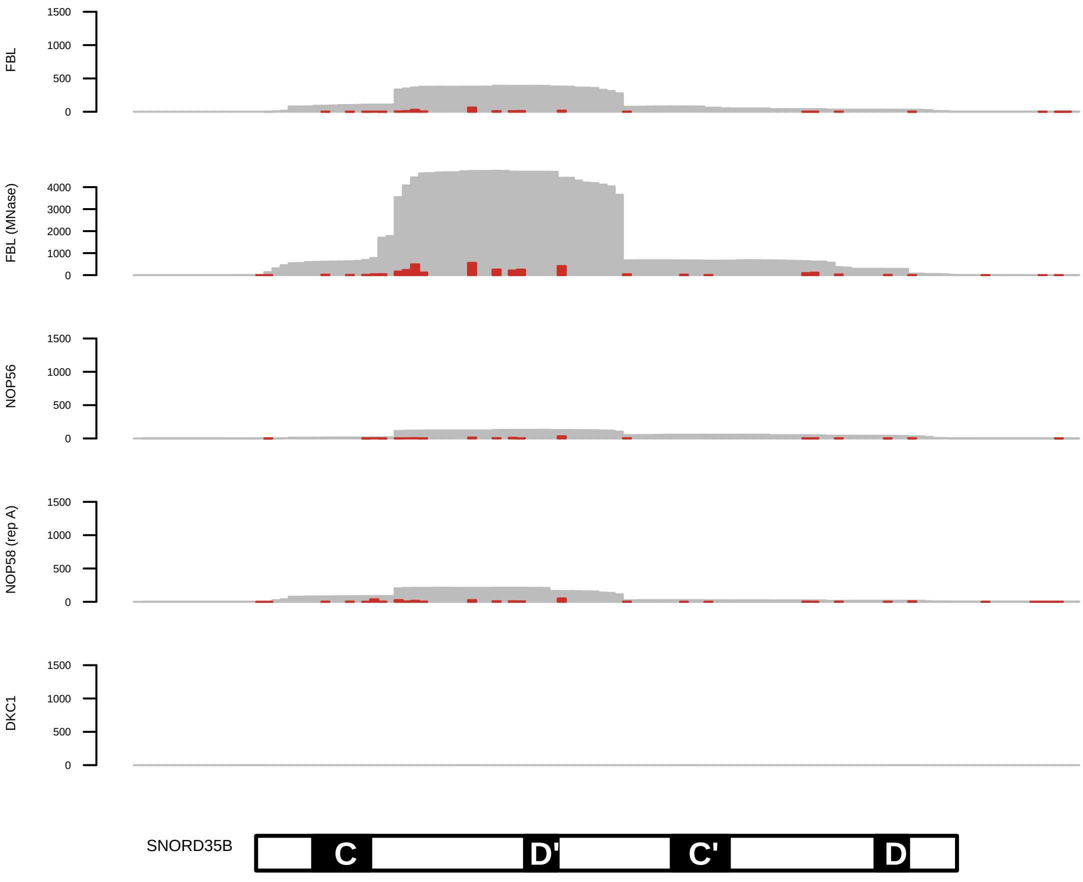
FBL
1500
1000
500
0
FBL (MNase)
4000
3000
2000
1000
0
NOP56
1500
1000
500
0
NOP58 (rep A)
1500
1000
500
0
DKC1
1500
1000
500
0
SNORD35B
C
D'
C'
D

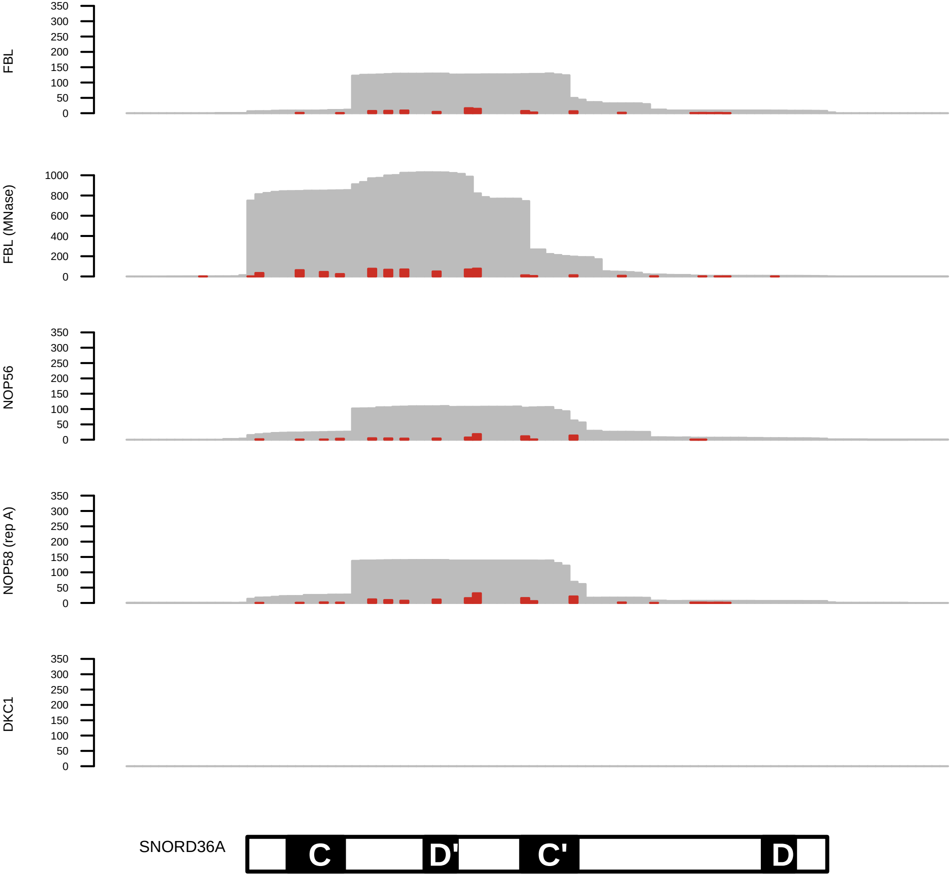

FBL
FBL (MNase)
NOP56
NOP58 (rep A)
DKC1
350
300
250
200
150
100
50
0
1000
800
600
400
200
SNORD36A
C
D'
C'
D

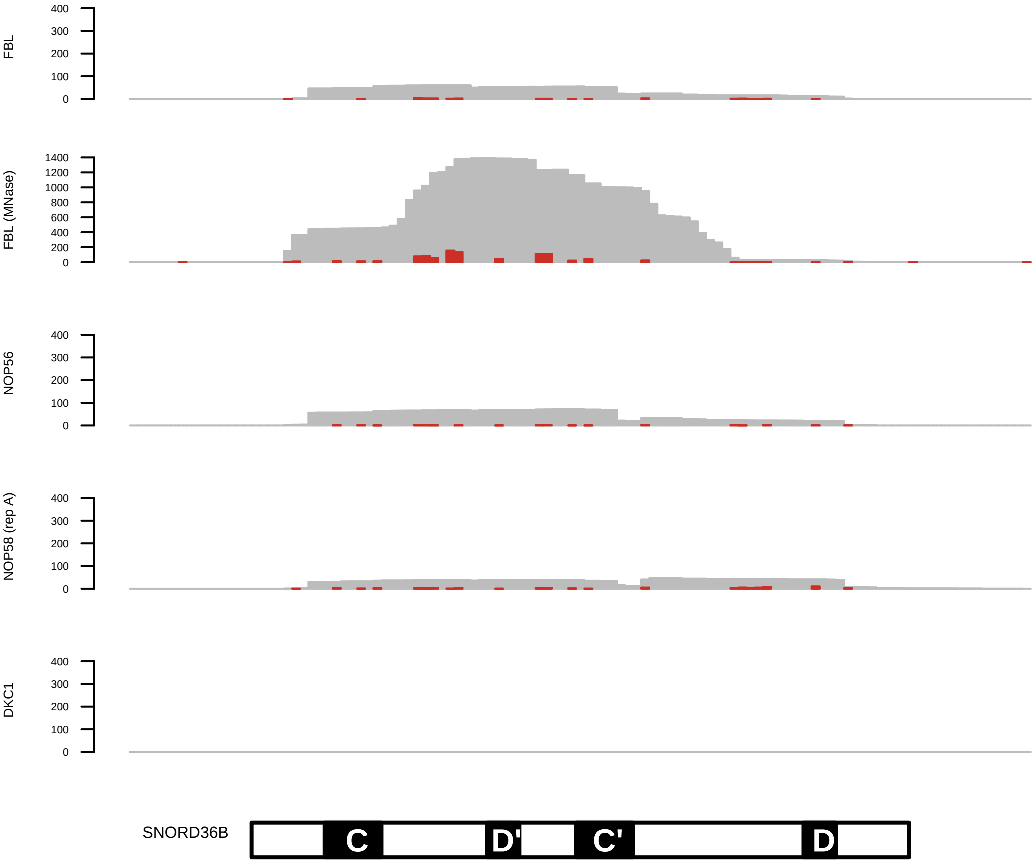

FBL
0
100
200
300
400
FBL (MNase)
0
200
400
600
800
1000
1200
1400
NOP56
0
100
200
300
400
NOP58 (rep A)
0
100
200
300
400
DKC1
0
100
200
300
400
SNORD36B
C
D'
C'
D

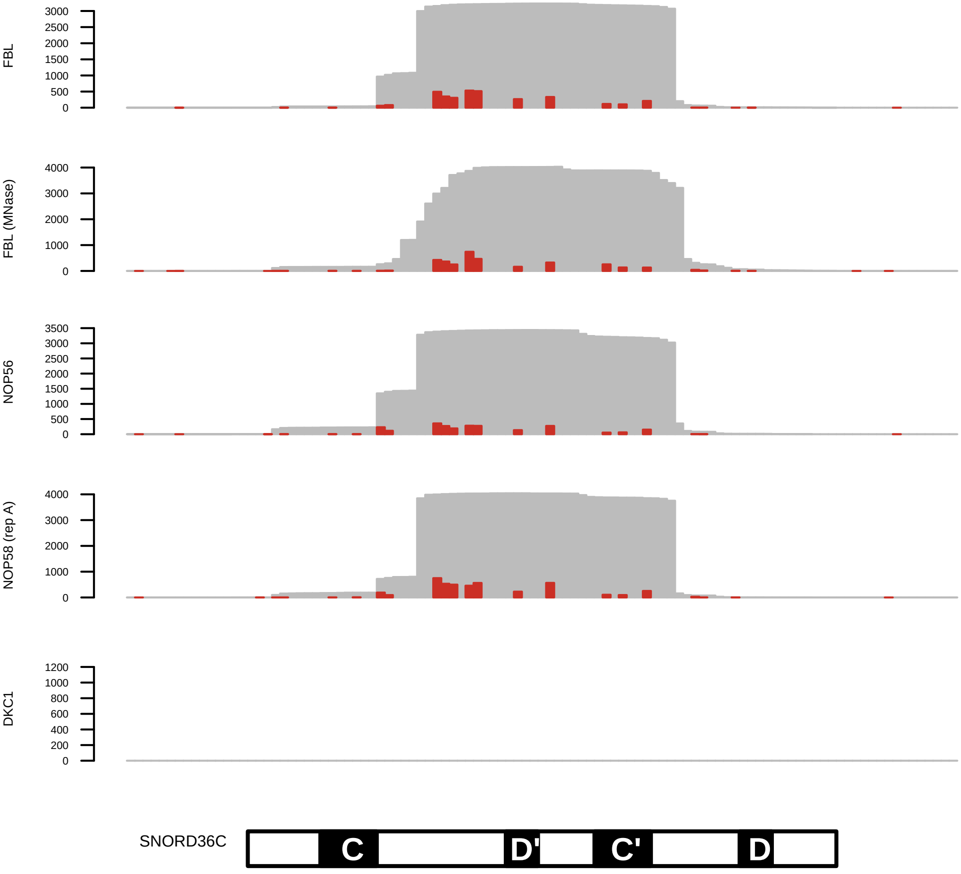

FBL
3000
2500
2000
1500
1000
500
0
FBL (MNase)
4000
3000
2000
1000
0
NOP56
3500
3000
2500
2000
1500
1000
500
0
NOP58 (rep A)
4000
3000
2000
1000
0
DKC1
1200
1000
800
600
400
200
0
SNORD36C
C
D'
C'
D

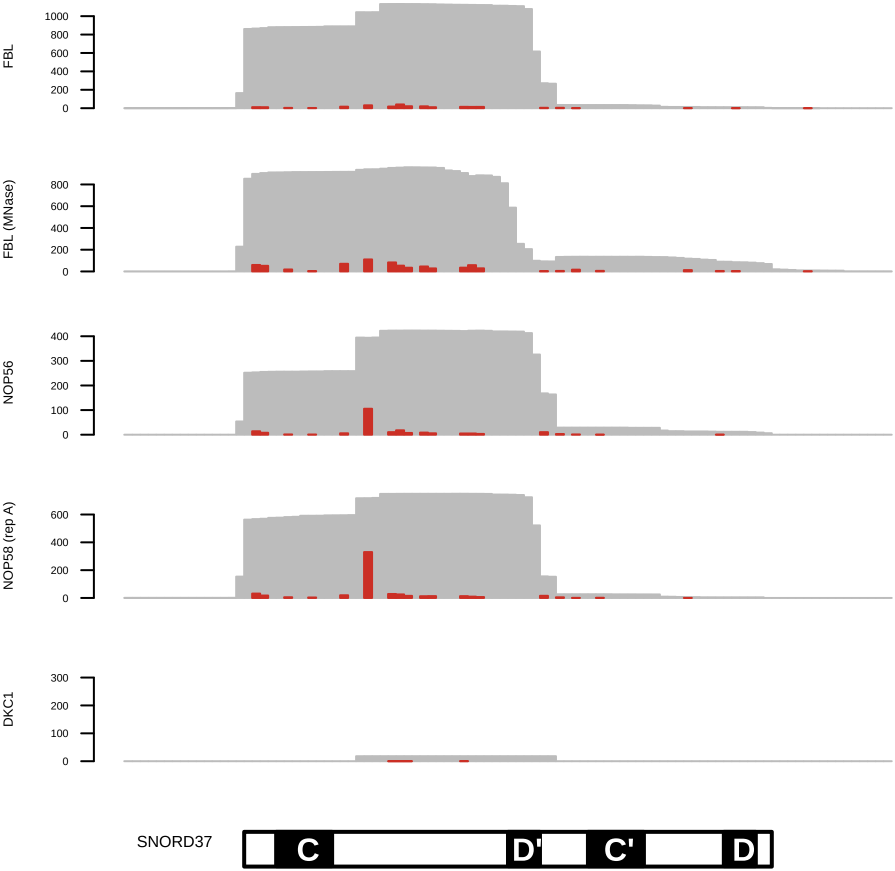

FBL
1000
800
600
400
200
0
FBL (MNase)
800
600
400
200
0
NOP56
400
300
200
100
0
NOP58 (rep A)
600
400
200
0
DKC1
300
200
100
0
SNORD37
C
D'
C'
D

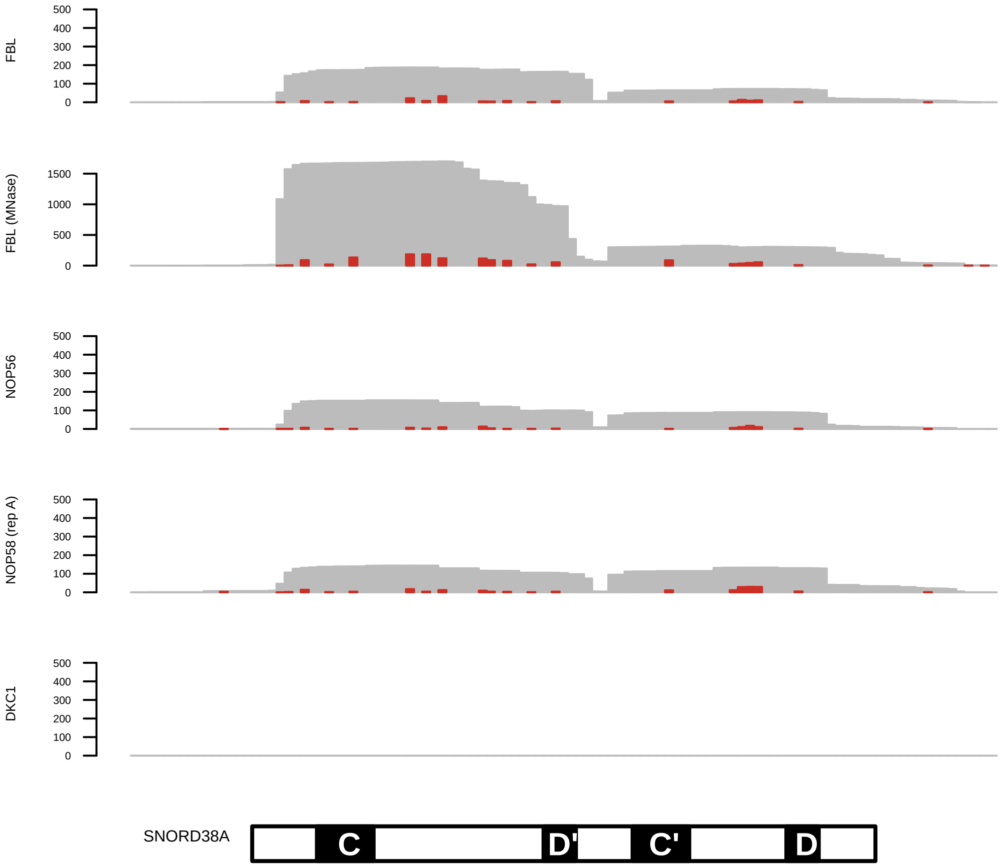

FBL
500
400
300
200
100
0
FBL (MNase)
1500
1000
500
0
NOP56
500
400
300
200
100
0
NOP58 (rep A)
500
400
300
200
100
0
DKC1
500
400
300
200
100
0
SNORD38A
C
D'
C'
D

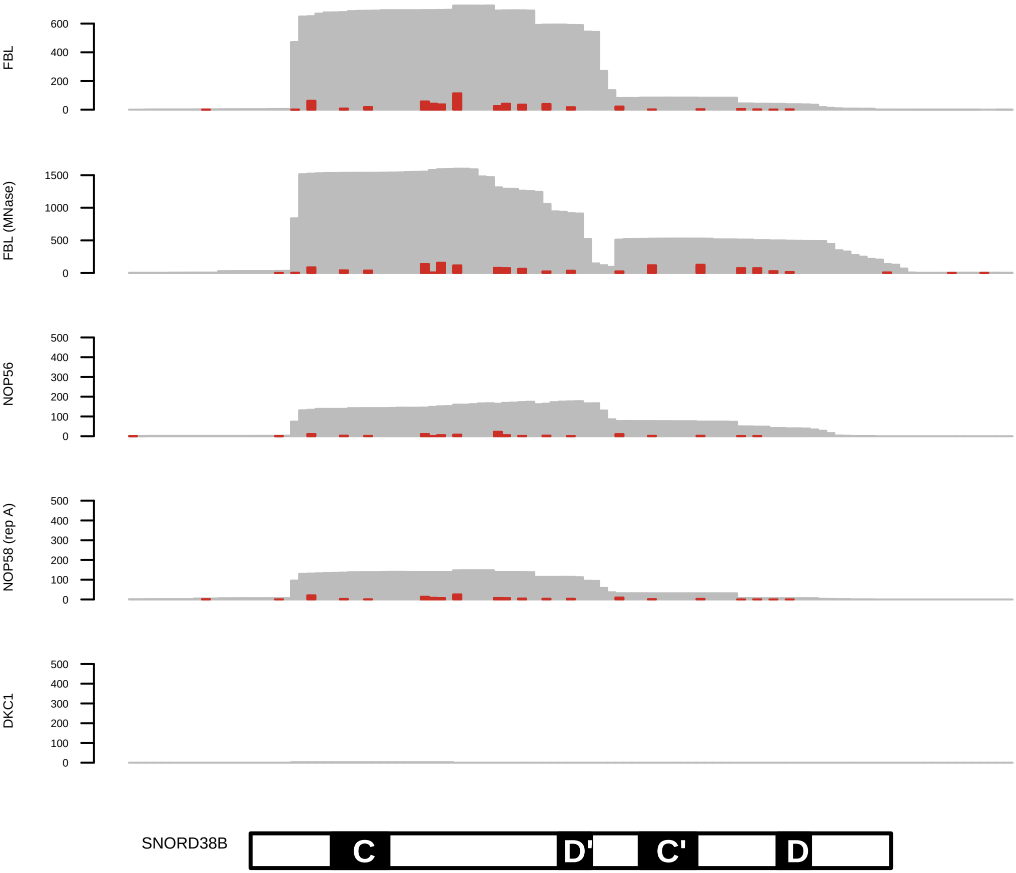

FBL
600
400
200
0
FBL (MNase)
1500
1000
500
0
NOP56
500
400
300
200
100
0
NOP58 (rep A)
500
400
300
200
100
0
DKC1
500
400
300
200
100
0
SNORD38B
C
D'
C'
D

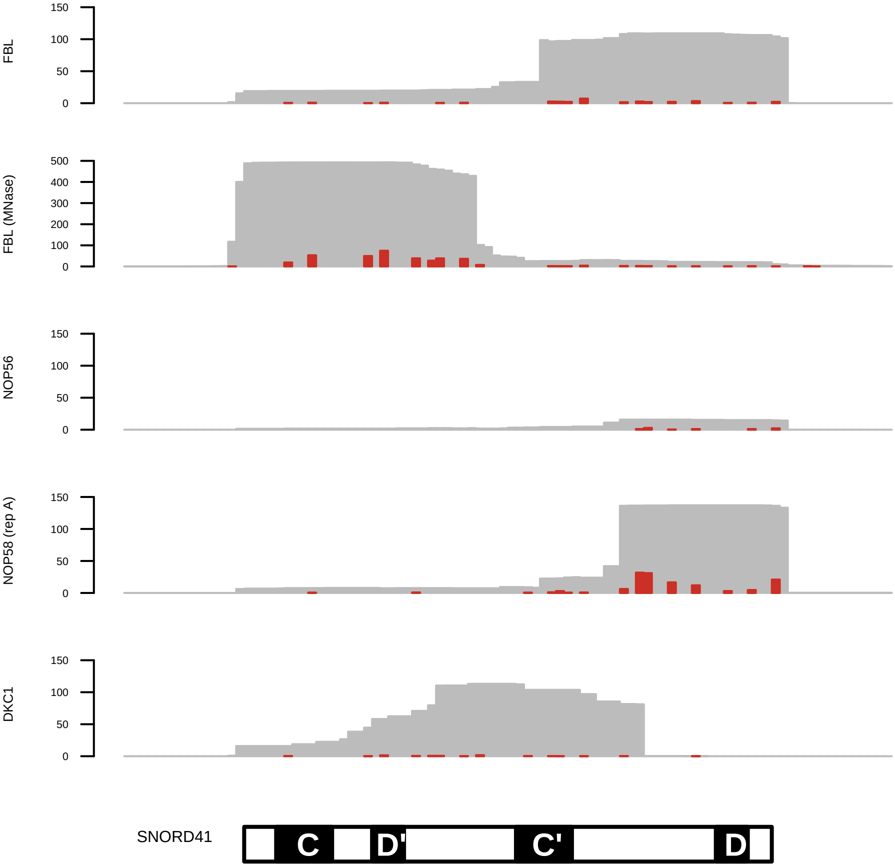

FBL
150
100
50
0
FBL (MNase)
500
400
300
200
100
0
NOP56
150
100
50
0
NOP58 (rep A)
150
100
50
0
DKC1
150
100
50
0
SNORD41
C
D'
C'
D

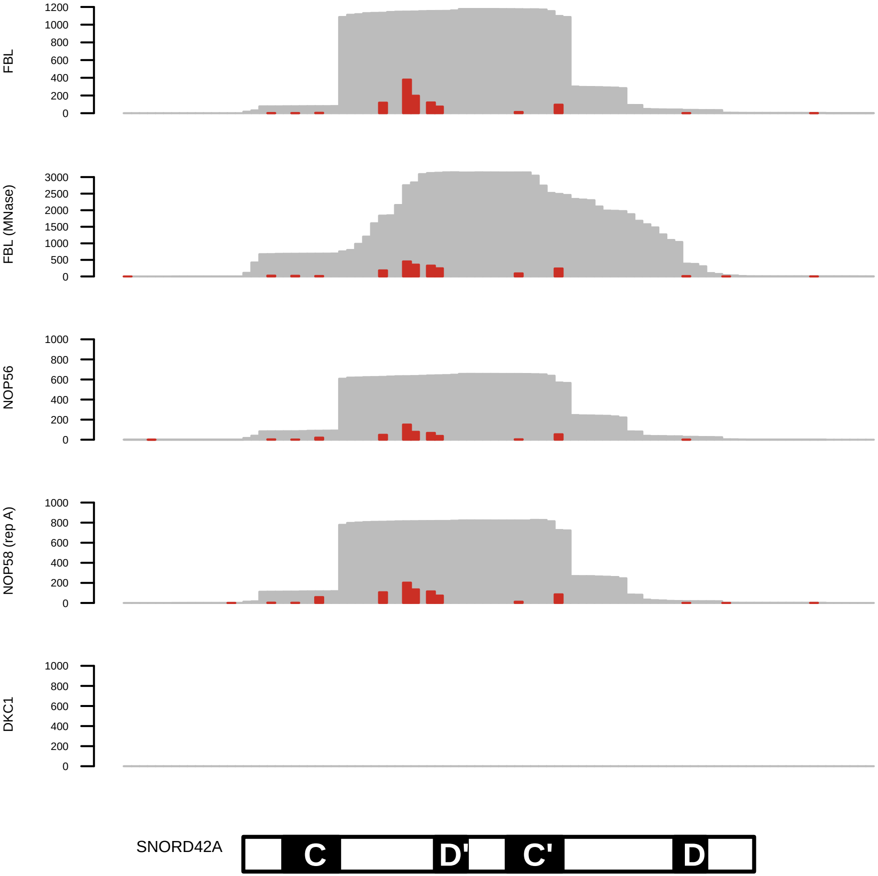

FBL
1200
1000
800
600
400
200
0
FBL (MNase)
3000
2500
2000
1500
1000
500
0
NOP56
1000
800
600
400
200
0
NOP58 (rep A)
1000
800
600
400
200
0
DKC1
1000
800
600
400
200
0
SNORD42A
C
D'
C'
D

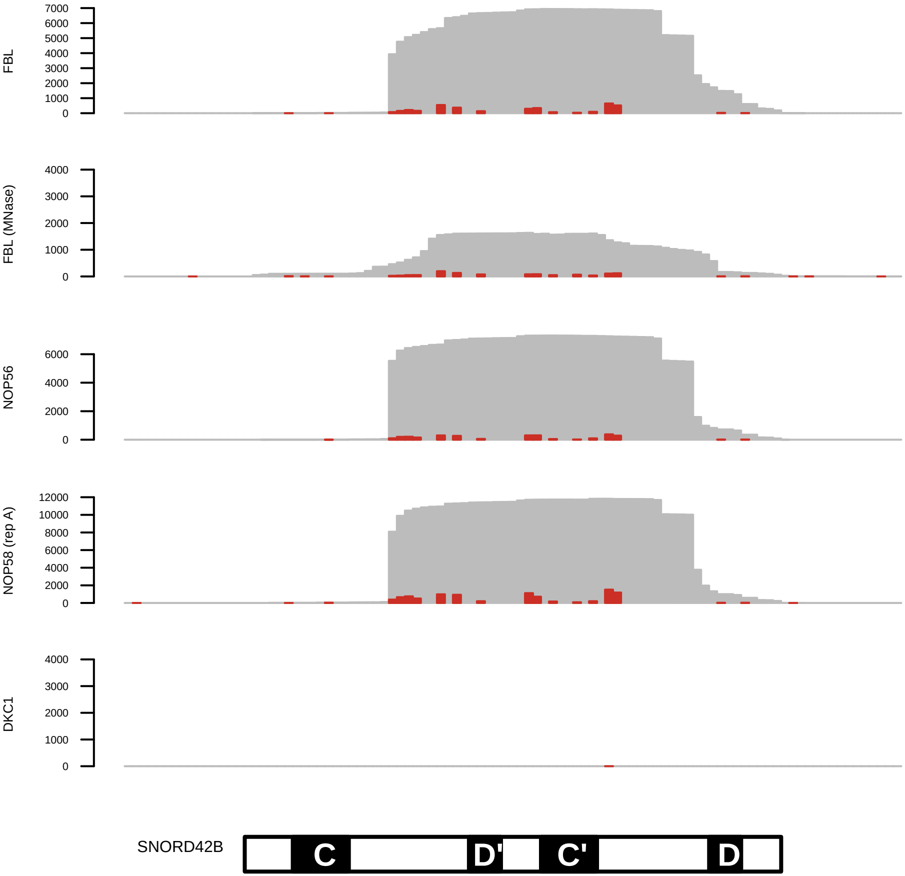

FBL
7000
6000
5000
4000
3000
2000
1000
0
FBL (MNase)
4000
3000
2000
1000
0
NOP56
6000
4000
2000
0
NOP58 (rep A)
12000
10000
8000
6000
4000
2000
0
DKC1
4000
3000
2000
1000
0
SNORD42B
C
D'
C'
D

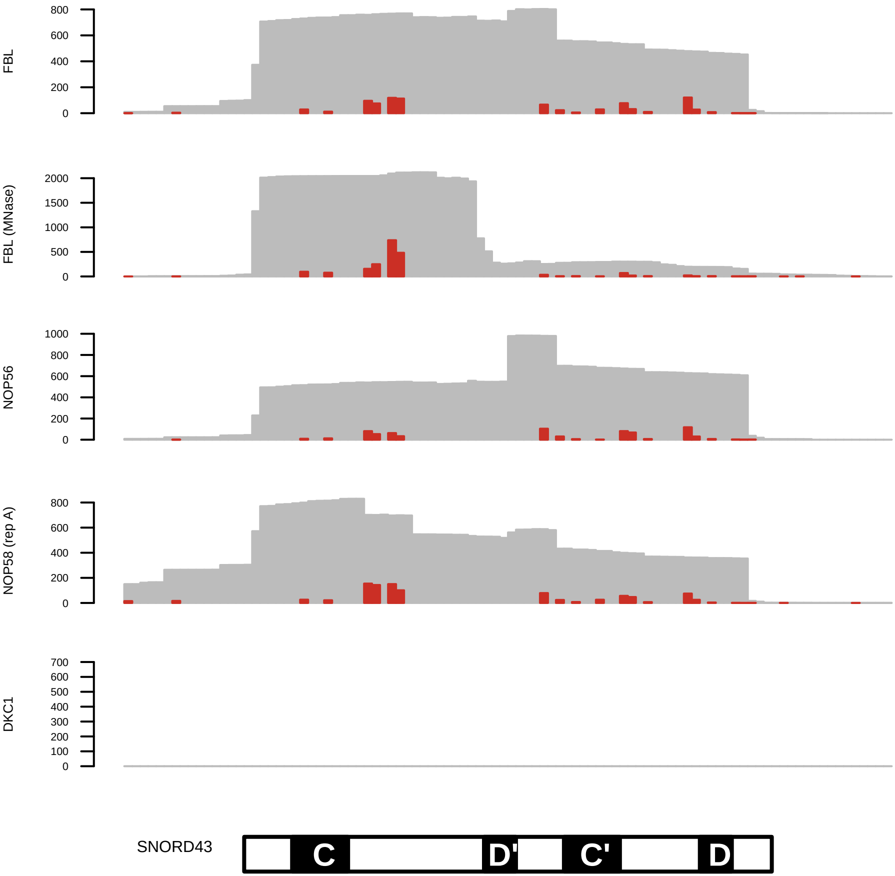
FBL
800
600
400
200
0
FBL (MNase)
2000
1500
1000
500
0
NOP56
1000
800
600
400
200
0
NOP58 (rep A)
800
600
400
200
0
DKC1
700
600
500
400
300
200
100
0
SNORD43
C
D'
C'
D

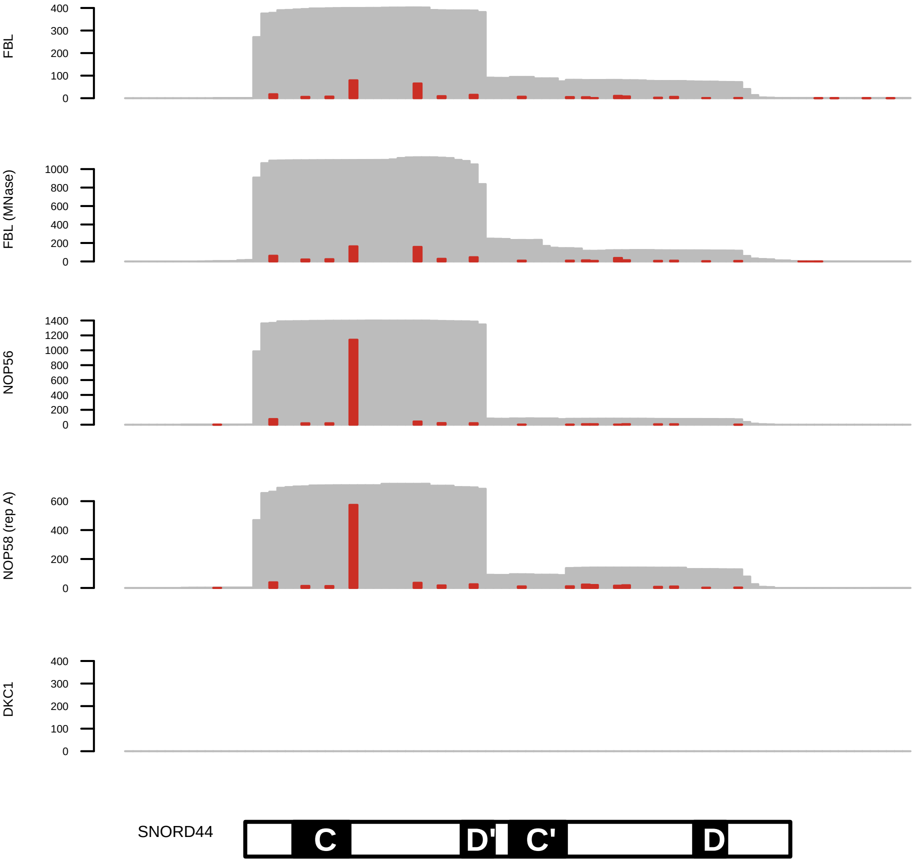
FBL
400
300
200
100
0
FBL (MNase)
1000
800
600
400
200
0
NOP56
1400
1200
1000
800
600
400
200
0
NOP58 (rep A)
600
400
200
0
DKC1
400
300
200
100
0
SNORD44
C
D'
C'
D

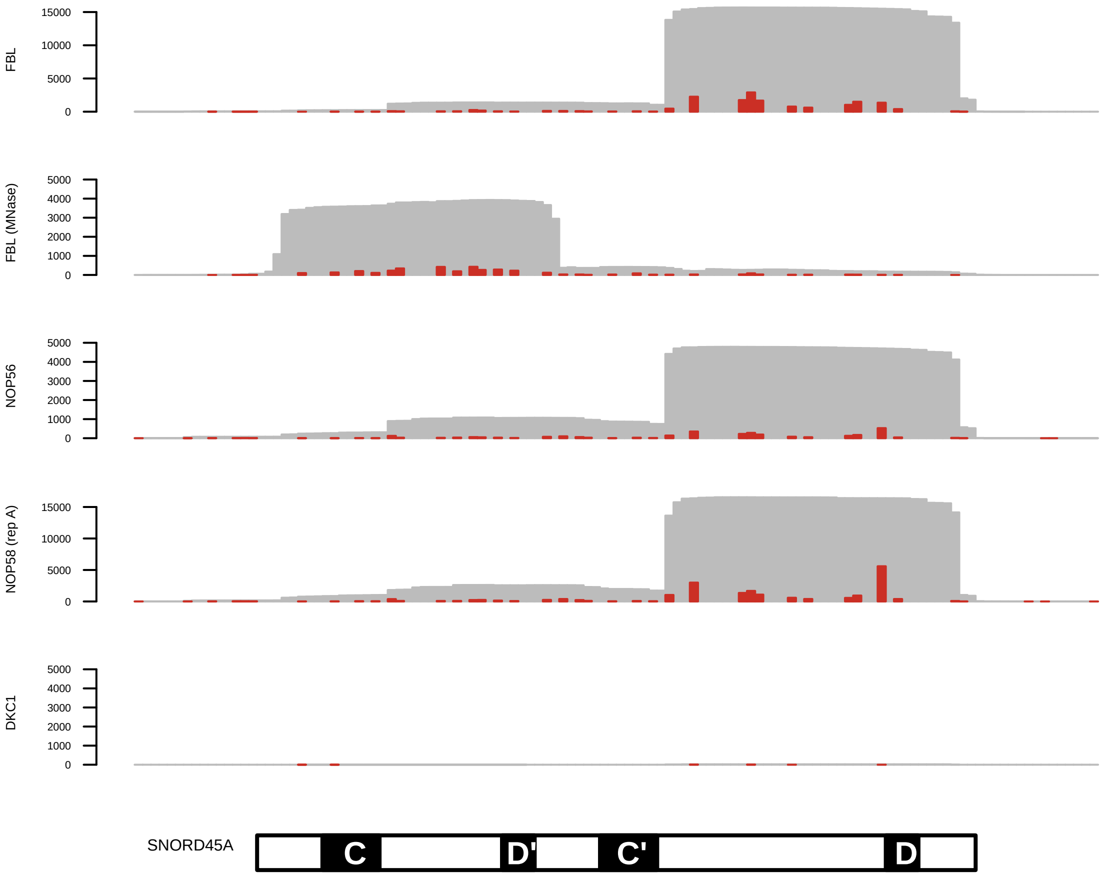
FBL
15000
10000
5000
0
FBL (MNase)
5000
4000
3000
2000
1000
0
NOP56
5000
4000
3000
2000
1000
0
NOP58 (rep A)
15000
10000
5000
0
DKC1
5000
4000
3000
2000
1000
0
SNORD45A
C
D'
C'
D

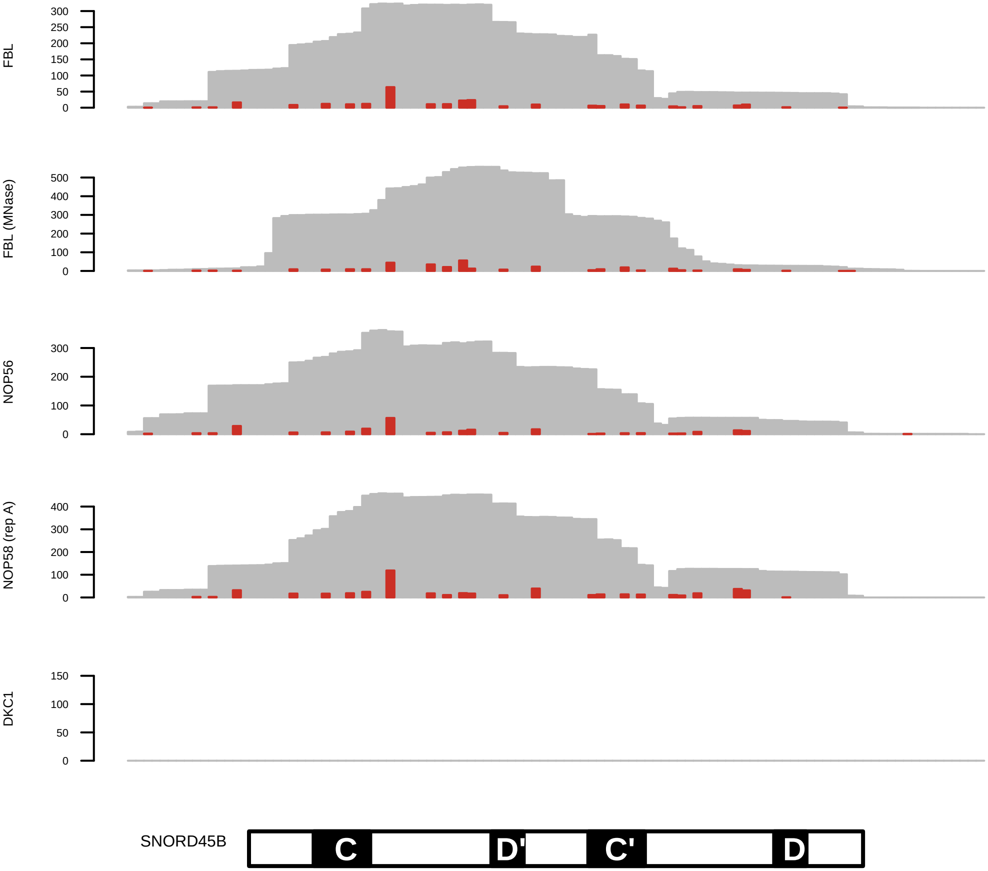

FBL
300
250
200
150
100
50
0
FBL (MNase)
500
400
300
200
100
0
NOP56
300
200
100
0
NOP58 (rep A)
400
300
200
100
0
DKC1
150
100
50
0
SNORD45B
C
D'
C'
D

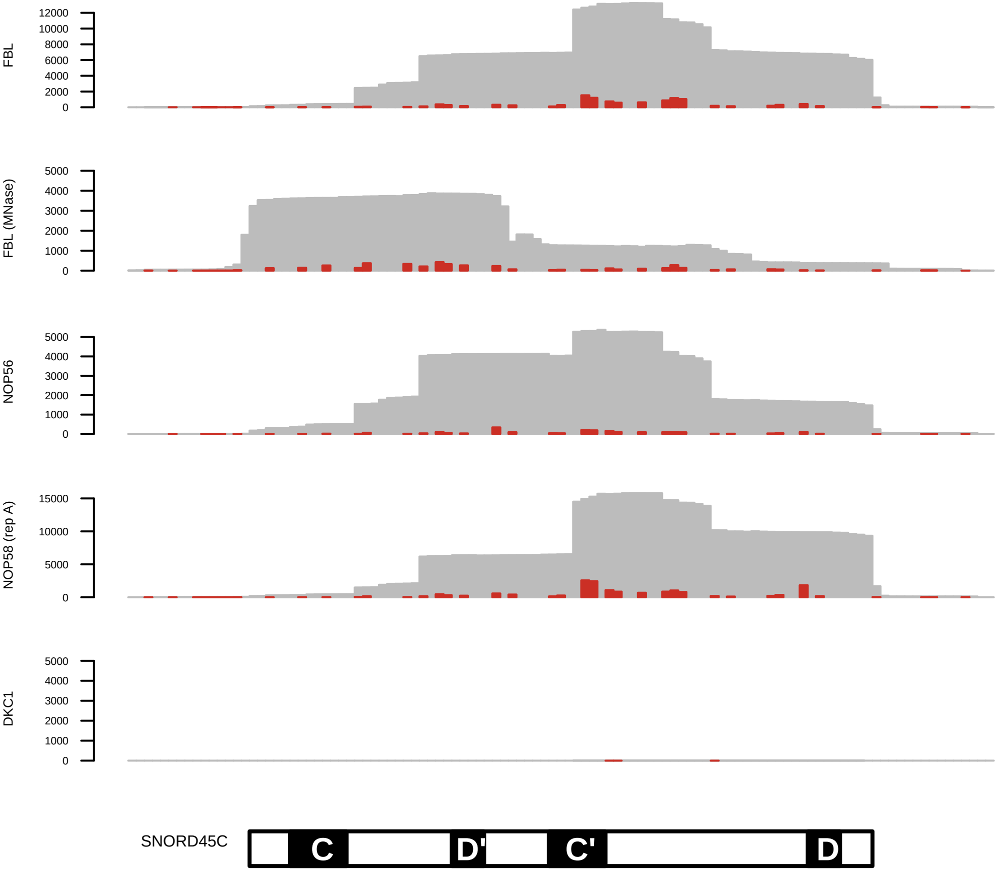
FBL
12000
10000
8000
6000
4000
2000
0
FBL (MNase)
5000
4000
3000
2000
1000
0
NOP56
5000
4000
3000
2000
1000
0
NOP58 (rep A)
15000
10000
5000
0
DKC1
5000
4000
3000
2000
1000
0
SNORD45C
C
D'
C'
D

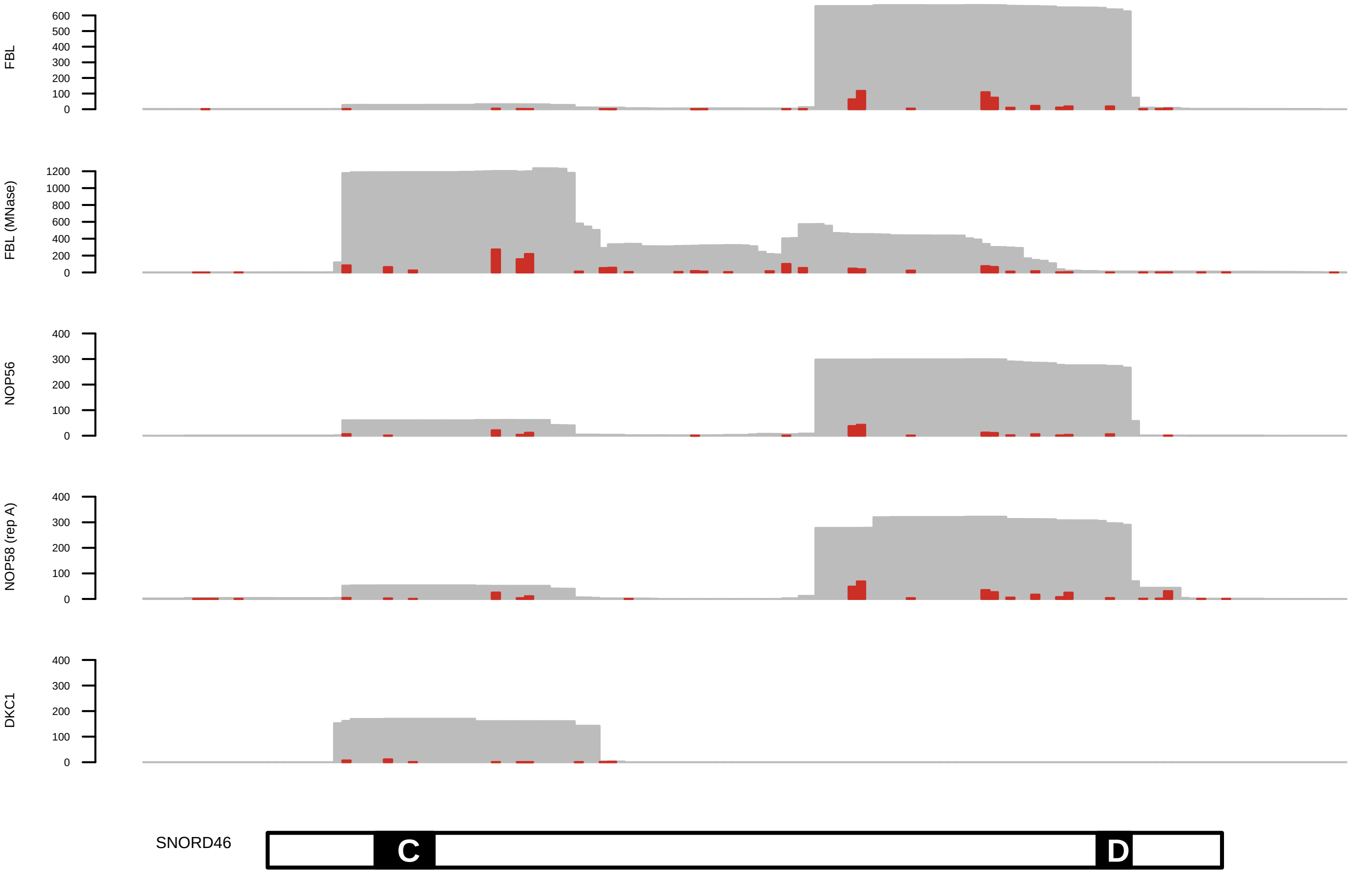

FBL
600
500
400
300
200
100
0
FBL (MNase)
1200
1000
800
600
400
200
0
NOP56
400
300
200
100
0
NOP58 (rep A)
400
300
200
100
0
DKC1
400
300
200
100
0
SNORD46
C
D

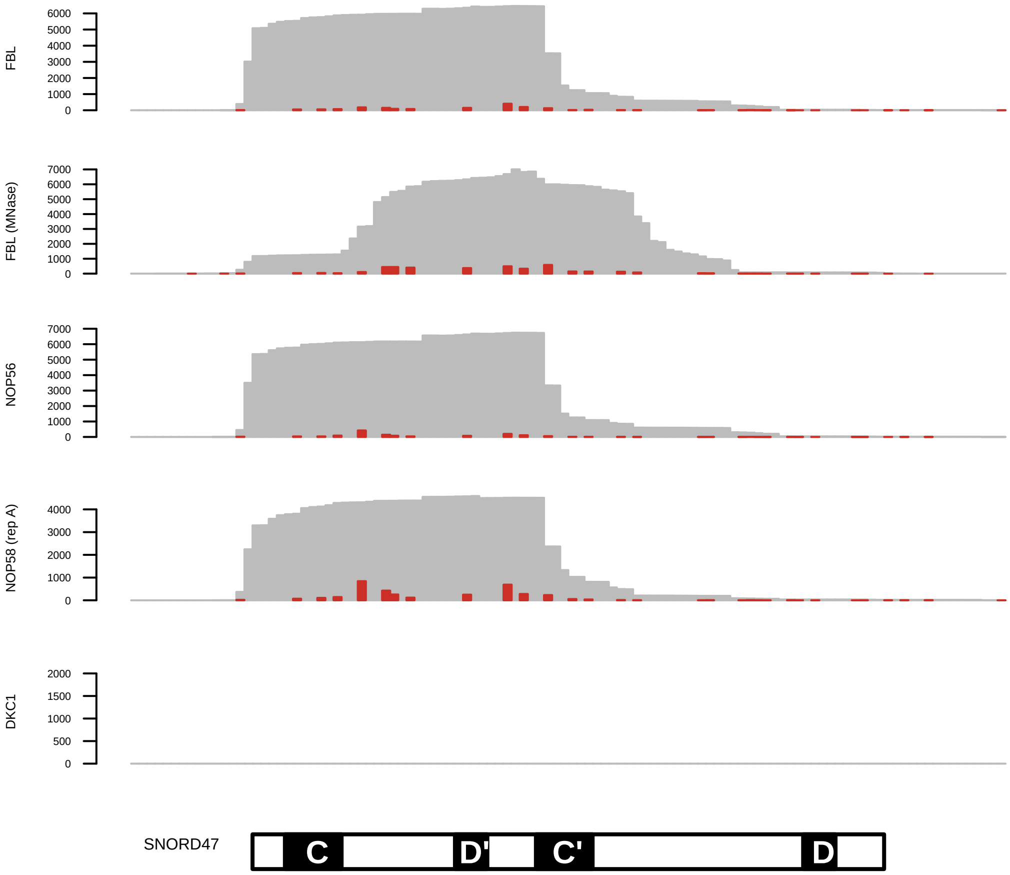
FBL
6000
5000
4000
3000
2000
1000
0
FBL (MNase)
7000
6000
5000
4000
3000
2000
1000
0
NOP56
7000
6000
5000
4000
3000
2000
1000
0
NOP58 (rep A)
4000
3000
2000
1000
0
DKC1
2000
1500
1000
500
0
SNORD47
C
D'
C'
D

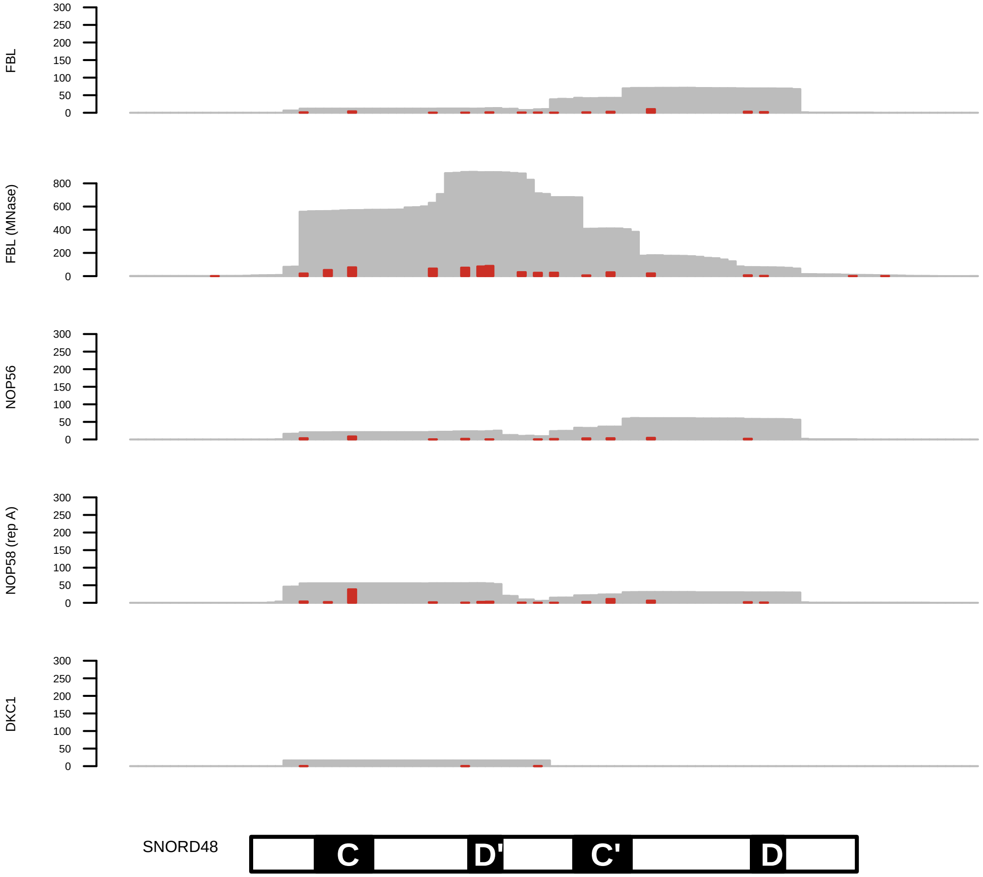

FBL
FBL (MNase)
NOP56
NOP58 (rep A)
DKC1
0
50
100
150
200
250
300
200
400
600
800
SNORD48
C
D'
C'
D

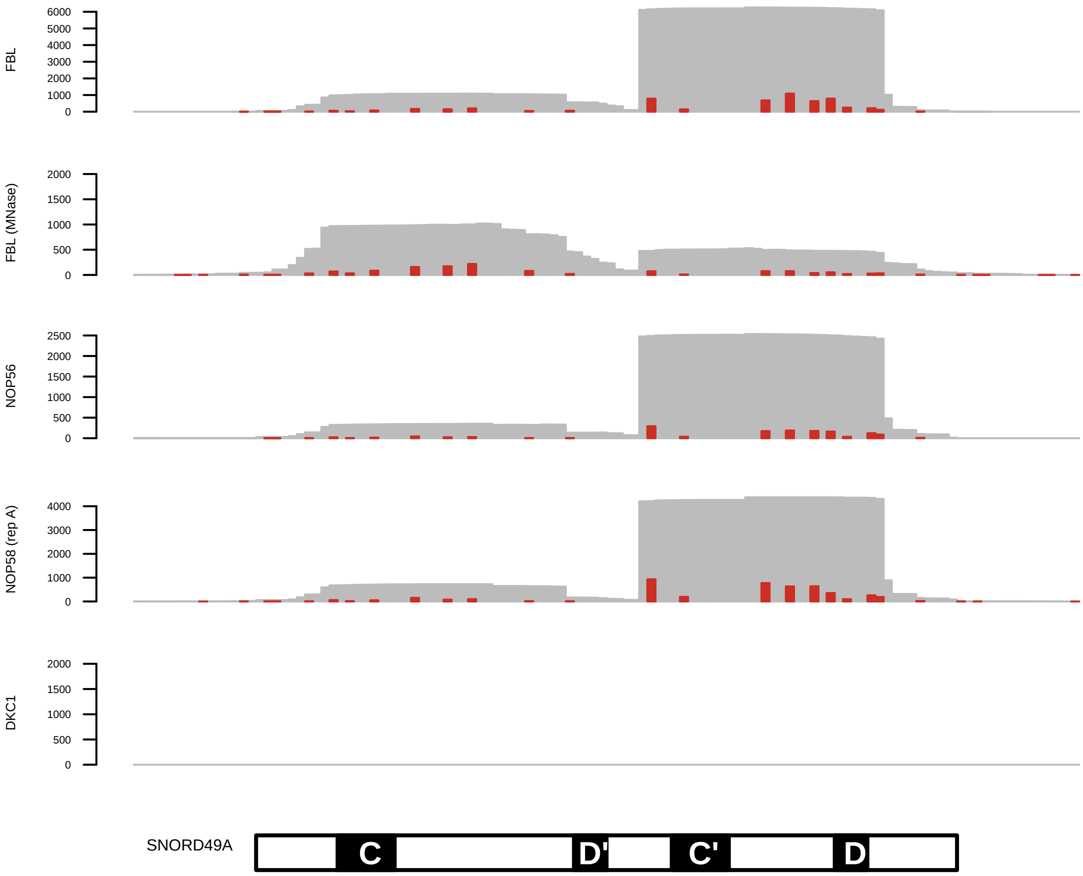
FBL
6000
5000
4000
3000
2000
1000
0
FBL (MNase)
2000
1500
1000
500
0
NOP56
2500
2000
1500
1000
500
0
NOP58 (rep A)
4000
3000
2000
1000
0
DKC1
2000
1500
1000
500
0
SNORD49A
C
D'
C'
D

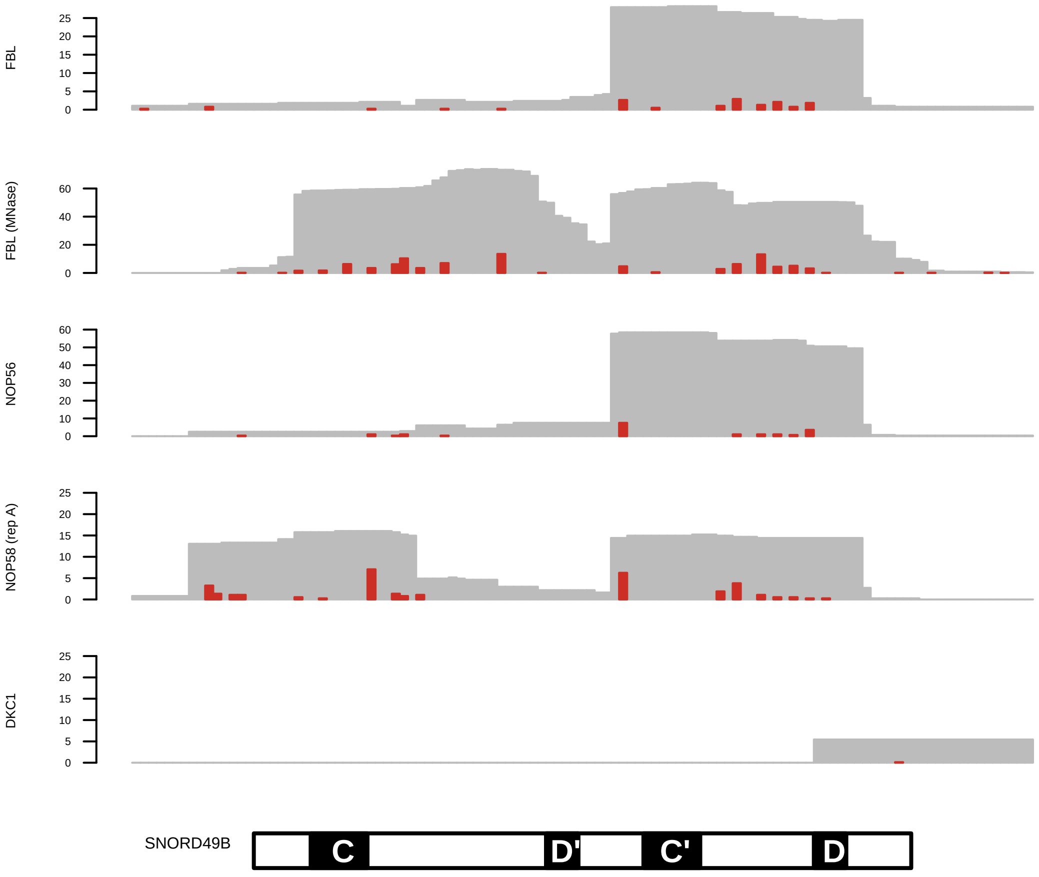

FBL
0 5 10 15 20 25
FBL (MNase)
0 20 40 60
NOP56
0 10 20 30 40 50 60
NOP58 (rep A)
0 5 10 15 20 25
DKC1
0 5 10 15 20 25
SNORD49B
C
D'
C'
D

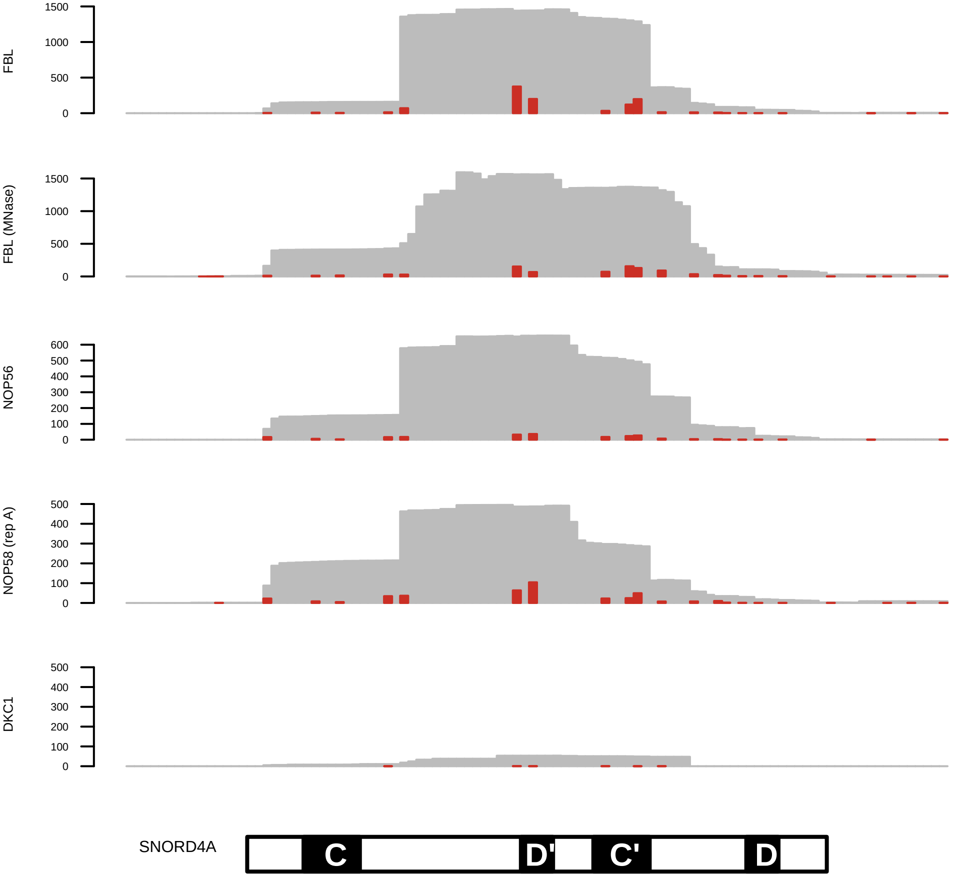
FBL
0
500
1000
1500
FBL (MNase)
0
500
1000
1500
NOP56
0
100
200
300
400
500
600
NOP58 (rep A)
0
100
200
300
400
500
DKC1
0
100
200
300
400
500
SNORD4A
C
D'
C'
D

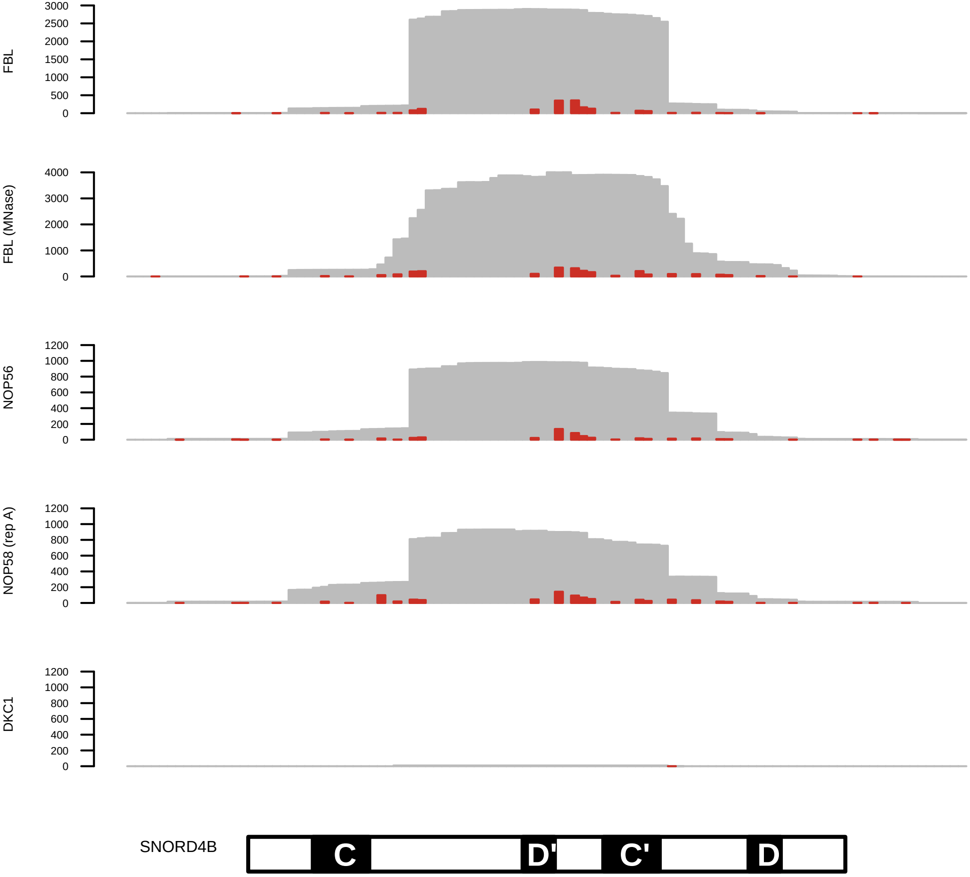
FBL
3000
2500
2000
1500
1000
500
0
FBL (MNase)
4000
3000
2000
1000
0
NOP56
1200
1000
800
600
400
200
0
NOP58 (rep A)
1200
1000
800
600
400
200
0
DKC1
1200
1000
800
600
400
200
0
SNORD4B
C
D'
C'
D

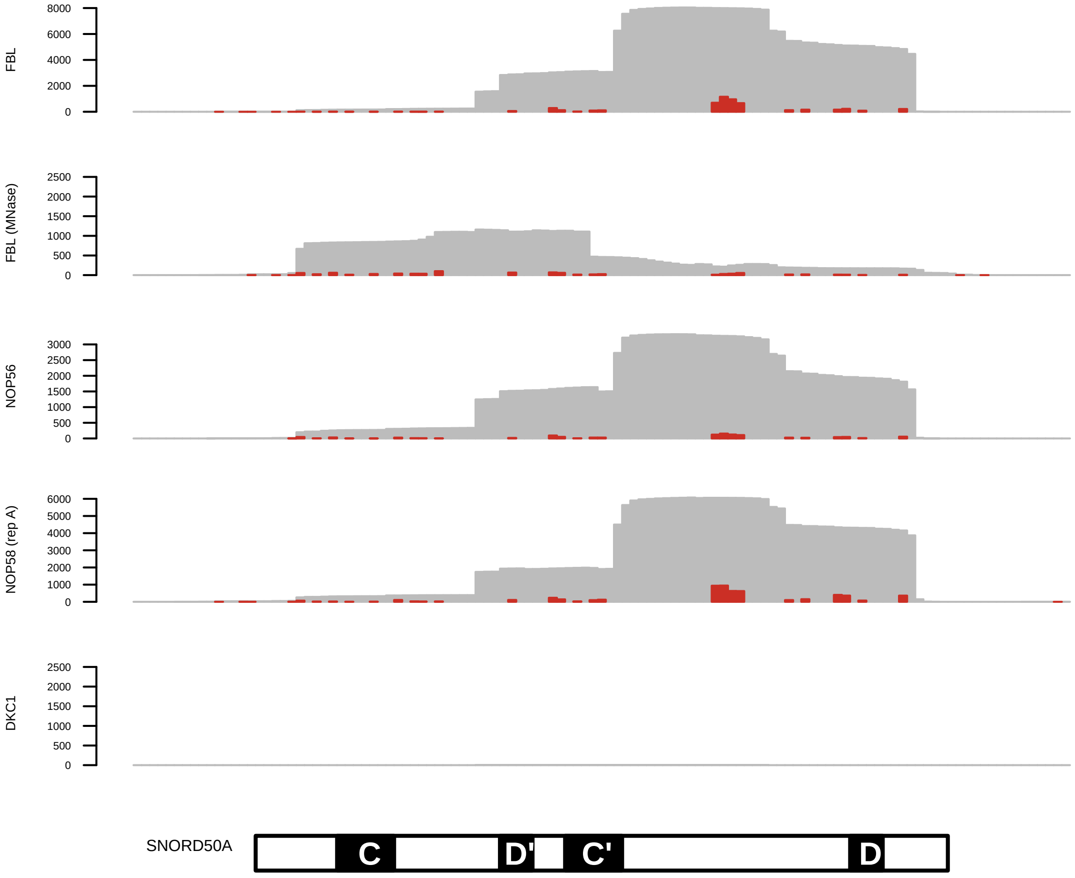

FBL
8000
6000
4000
2000
0
FBL (MNase)
2500
2000
1500
1000
500
0
NOP56
3000
2500
2000
1500
1000
500
0
NOP58 (rep A)
6000
5000
4000
3000
2000
1000
0
DKC1
2500
2000
1500
1000
500
0
SNORD50A
C
D'
C'
D

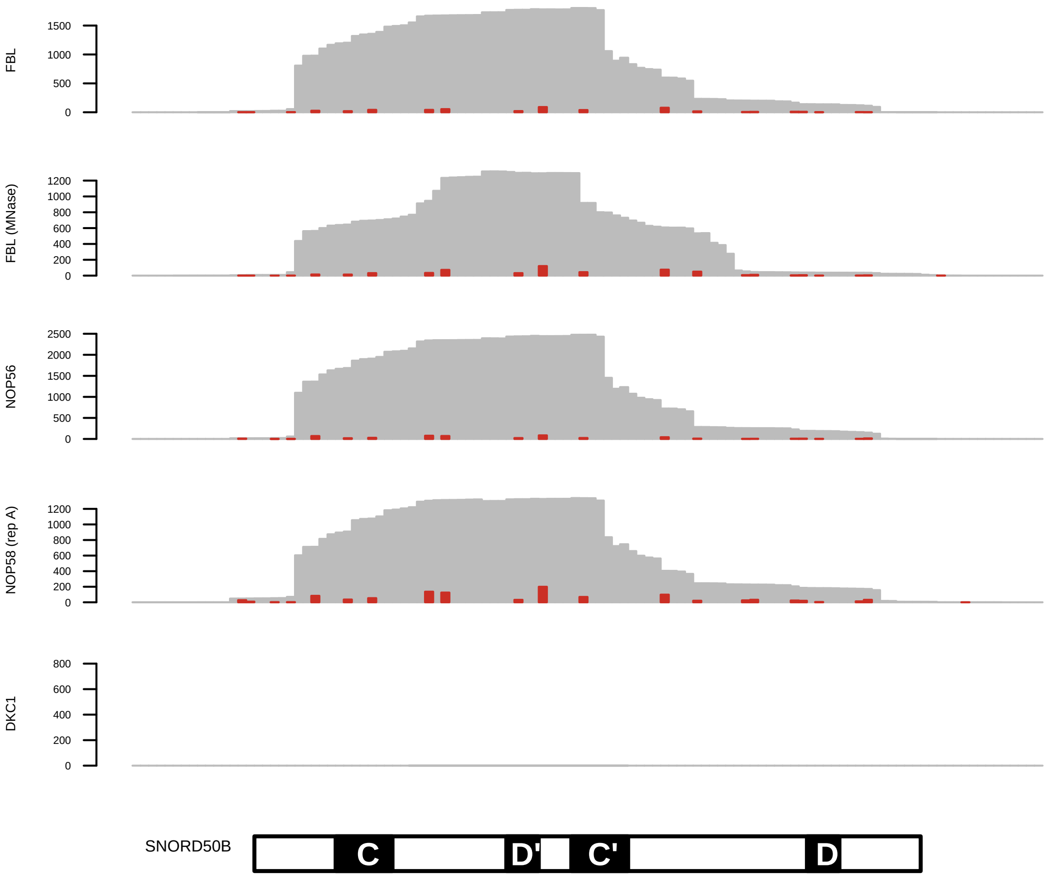
FBL
1500
1000
500
0
FBL (MNase)
1200
1000
800
600
400
200
0
NOP56
2500
2000
1500
1000
500
0
NOP58 (rep A)
1200
1000
800
600
400
200
0
DKC1
800
600
400
200
0
SNORD50B
C
D'
C'
D

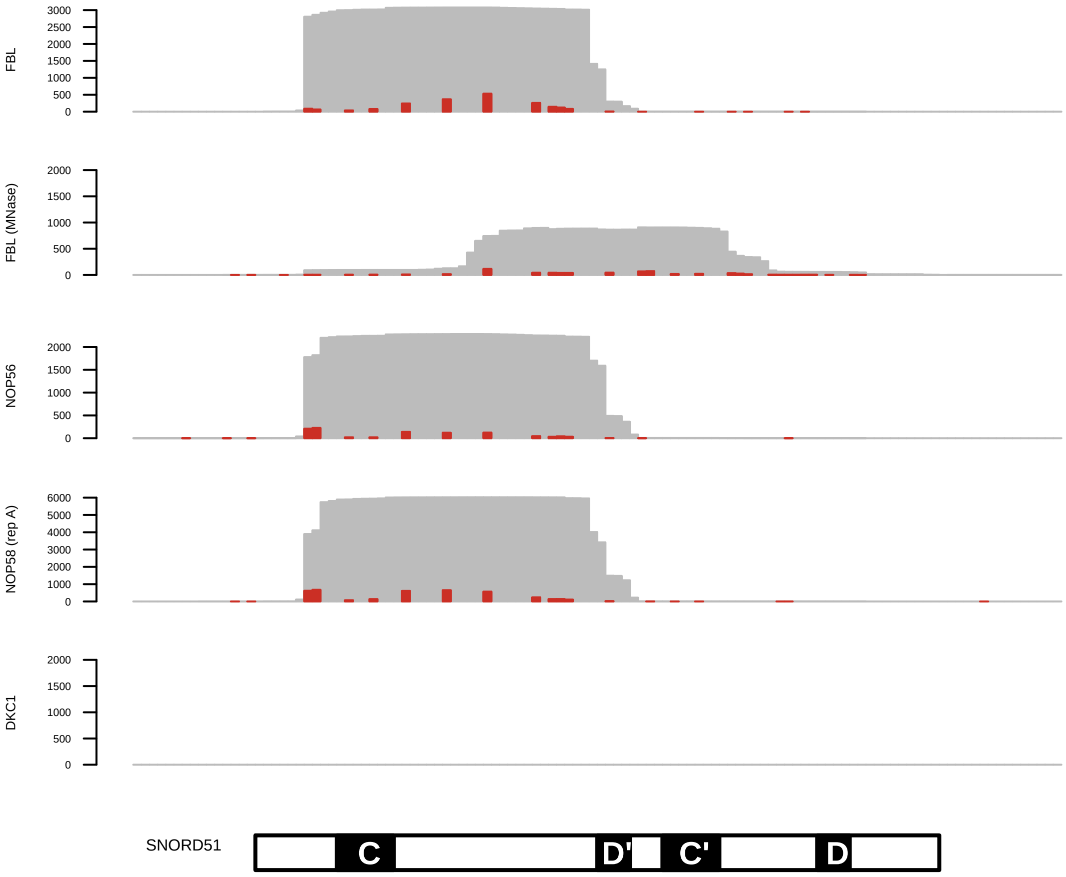
FBL
3000
2500
2000
1500
1000
500
0
FBL (MNase)
2000
1500
1000
500
0
NOP56
2000
1500
1000
500
0
NOP58 (rep A)
6000
5000
4000
3000
2000
1000
0
DKC1
2000
1500
1000
500
0
SNORD51
C
D'
C'
D

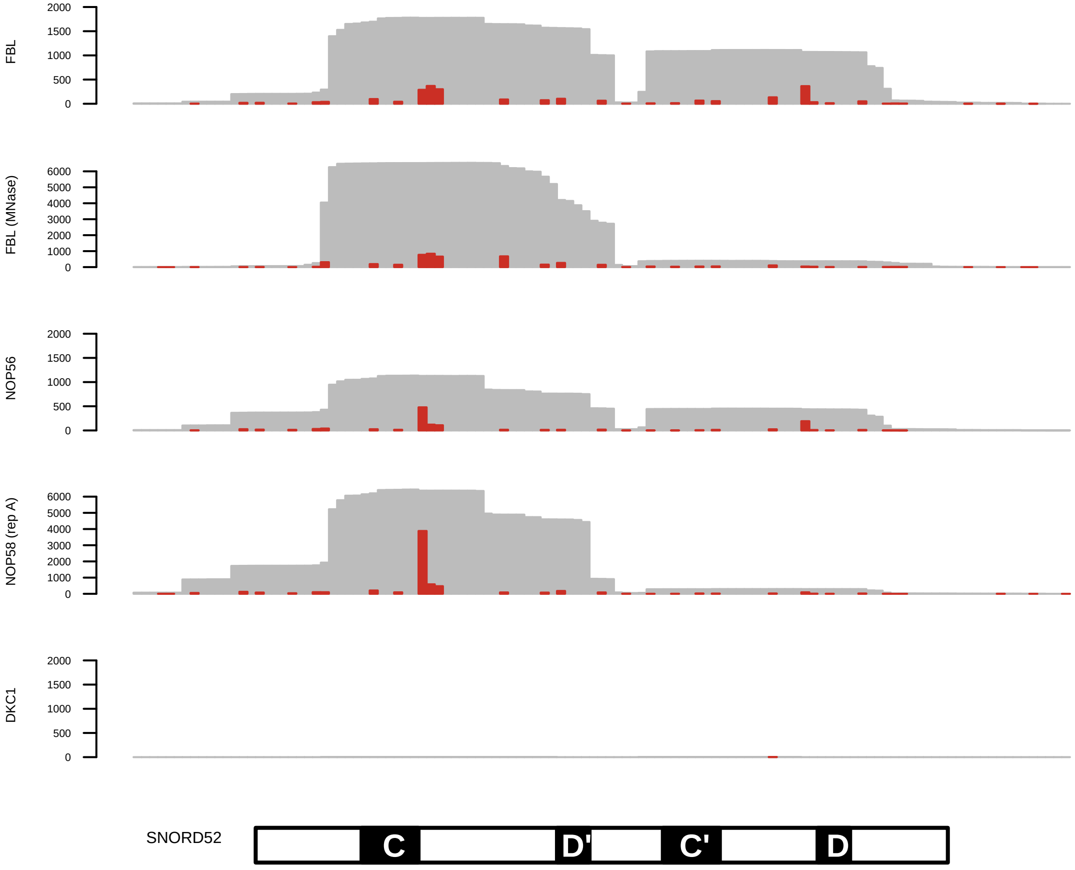

FBL
2000
1500
1000
500
0
FBL (MNase)
6000
5000
4000
3000
2000
1000
0
NOP56
2000
1500
1000
500
0
NOP58 (rep A)
6000
5000
4000
3000
2000
1000
0
DKC1
2000
1500
1000
500
0
SNORD52
C
D'
C'
D

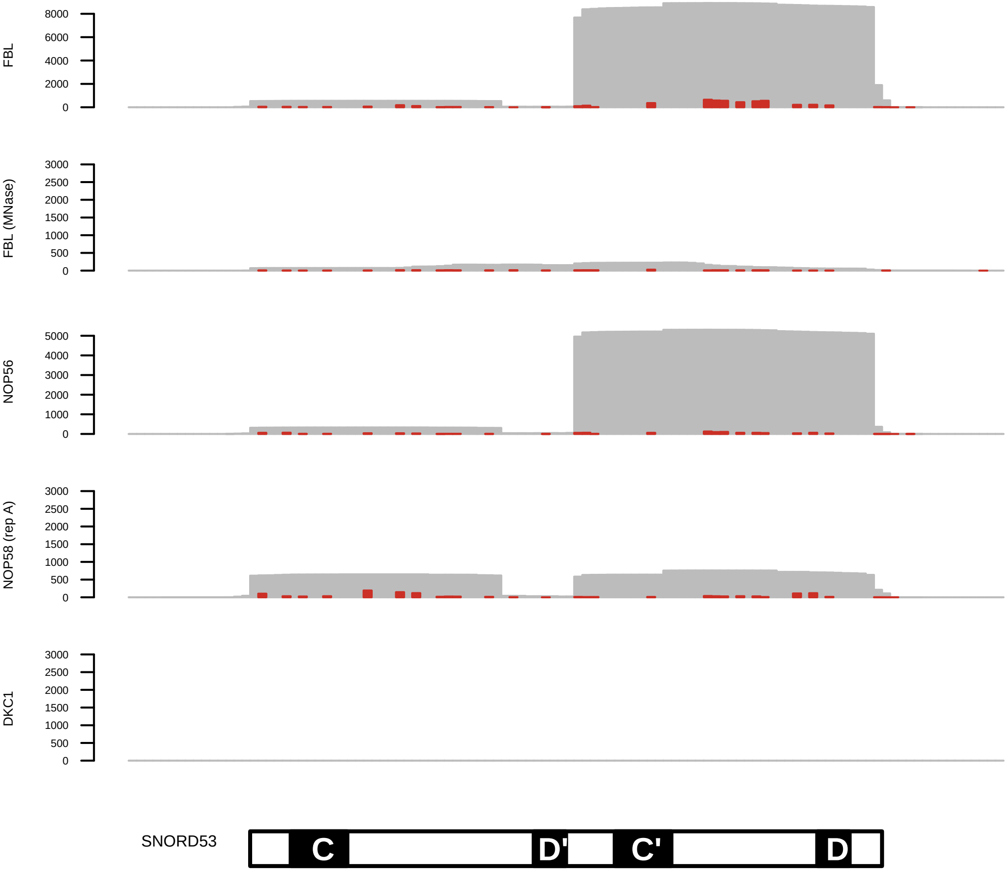

FBL
8000
6000
4000
2000
0
FBL (MNase)
3000
2500
2000
1500
1000
500
0
NOP56
5000
4000
3000
2000
1000
0
NOP58 (rep A)
3000
2500
2000
1500
1000
500
0
DKC1
3000
2500
2000
1500
1000
500
0
SNORD53
C
D'
C'
D

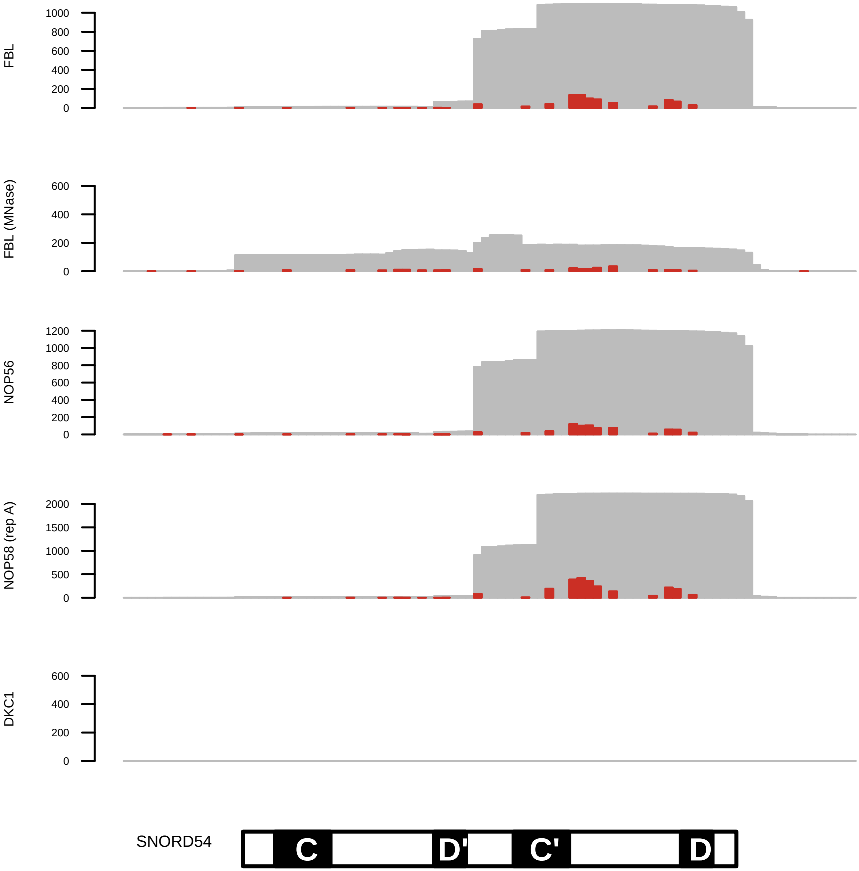
FBL
1000
800
600
400
200
0
FBL (MNase)
600
400
200
0
NOP56
1200
1000
800
600
400
200
0
NOP58 (rep A)
2000
1500
1000
500
0
DKC1
600
400
200
0
SNORD54
C
D'
C'
D

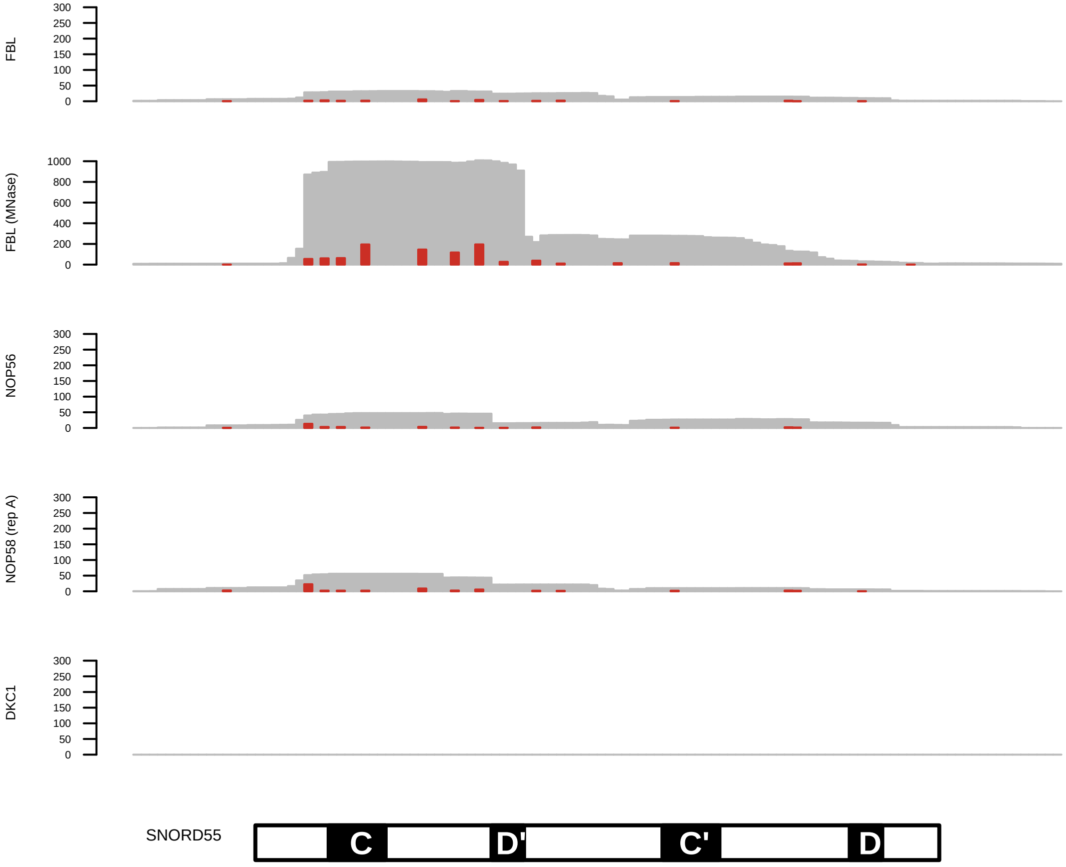

FBL
FBL (MNase)
NOP56
NOP58 (rep A)
DKC1
0
50
100
150
200
250
300
200
400
600
800
1000
SNORD55
C
D'
C'
D

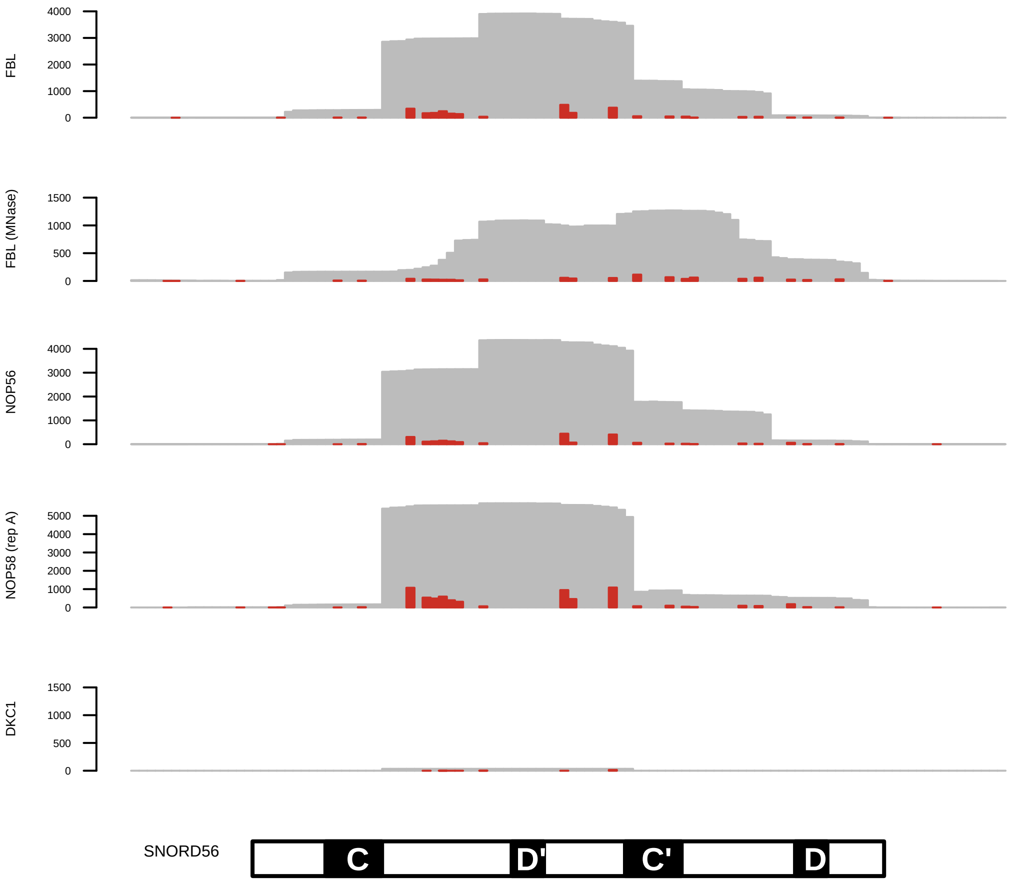
FBL
4000
3000
2000
1000
0
FBL (MNase)
1500
1000
500
0
NOP56
4000
3000
2000
1000
0
NOP58 (rep A)
5000
4000
3000
2000
1000
0
DKC1
1500
1000
500
0
SNORD56
C
D'
C'
D

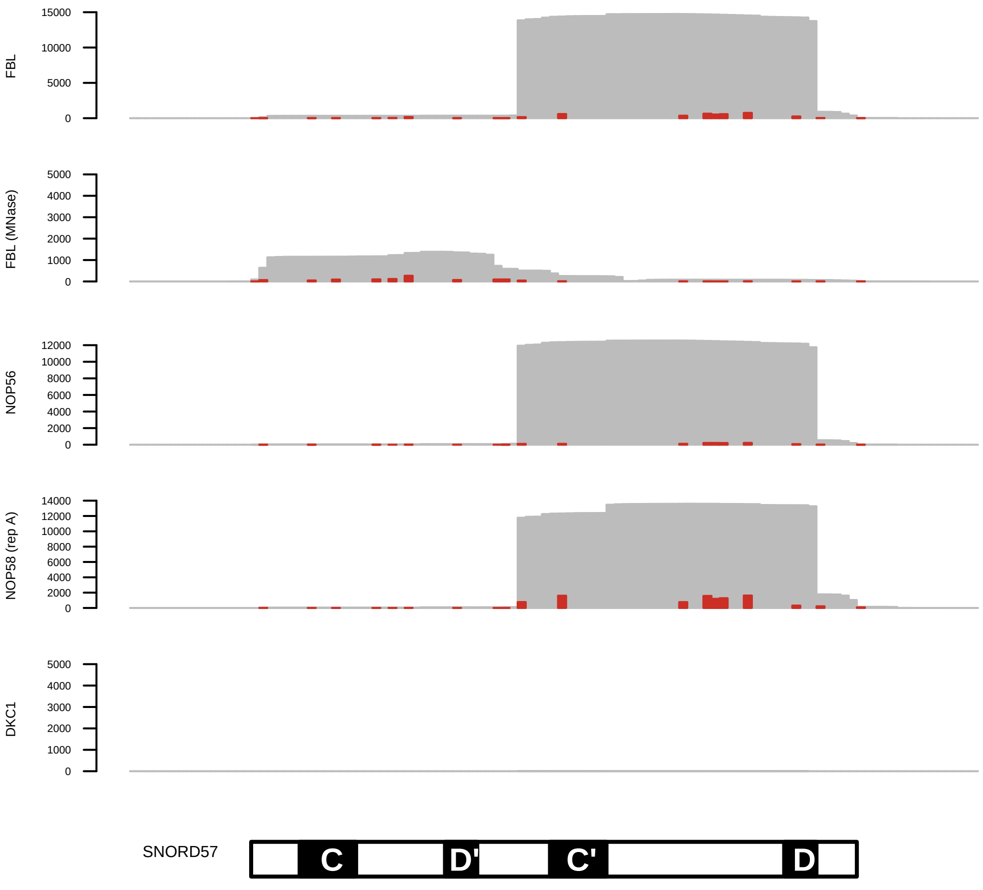

FBL
15000
10000
5000
0
FBL (MNase)
5000
4000
3000
2000
1000
0
NOP56
12000
10000
8000
6000
4000
2000
0
NOP58 (rep A)
14000
12000
10000
8000
6000
4000
2000
0
DKC1
5000
4000
3000
2000
1000
0
SNORD57
C
D'
C'
D

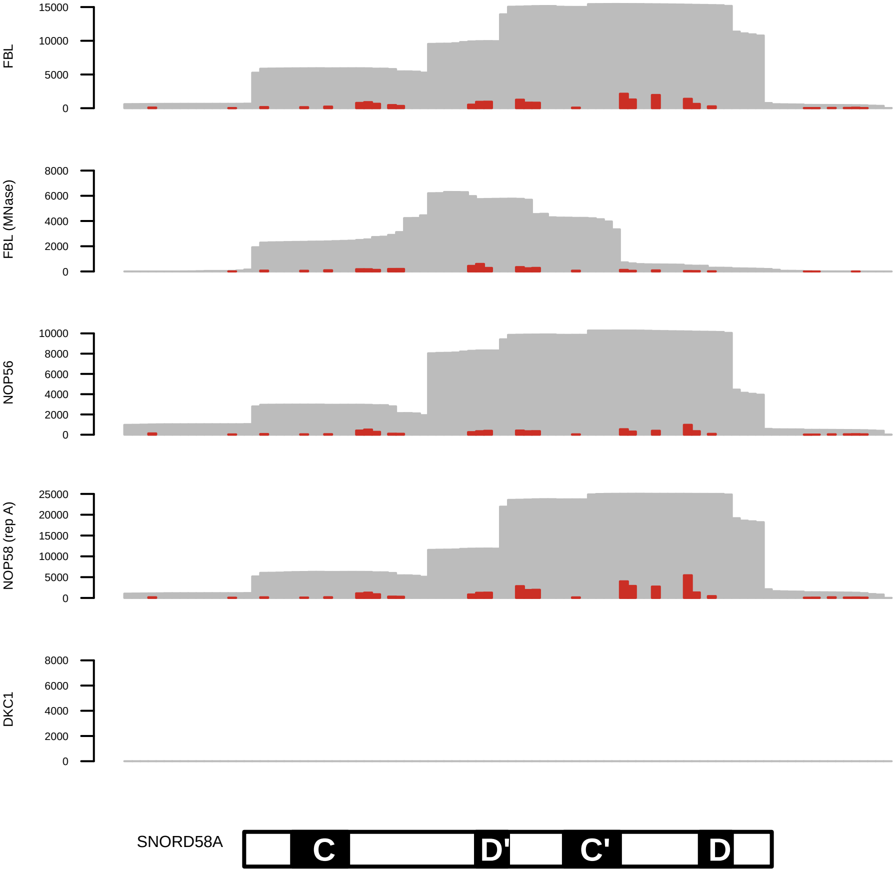
FBL
15000
10000
5000
0
FBL (MNase)
8000
6000
4000
2000
0
NOP56
10000
8000
6000
4000
2000
0
NOP58 (rep A)
25000
20000
15000
10000
5000
0
DKC1
8000
6000
4000
2000
0
SNORD58A
C
D'
C'
D

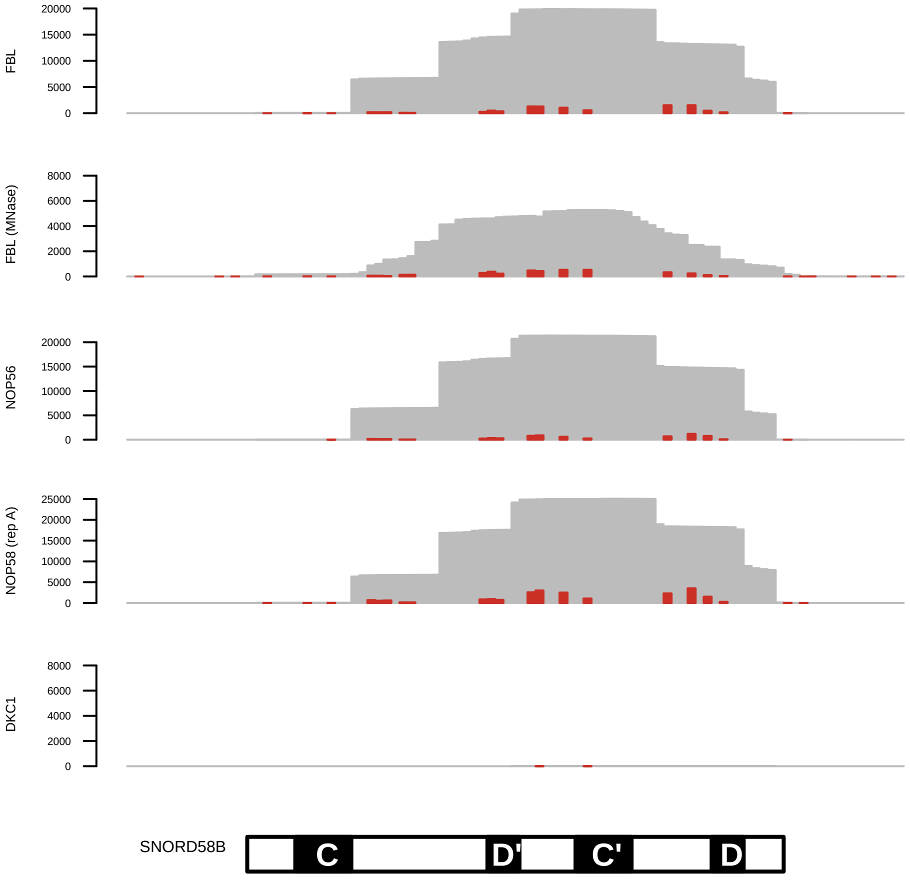

FBL
20000
15000
10000
5000
0
FBL (MNase)
8000
6000
4000
2000
0
NOP56
20000
15000
10000
5000
0
NOP58 (rep A)
25000
20000
15000
10000
5000
0
DKC1
8000
6000
4000
2000
0
SNORD58B
C
D'
C'
D

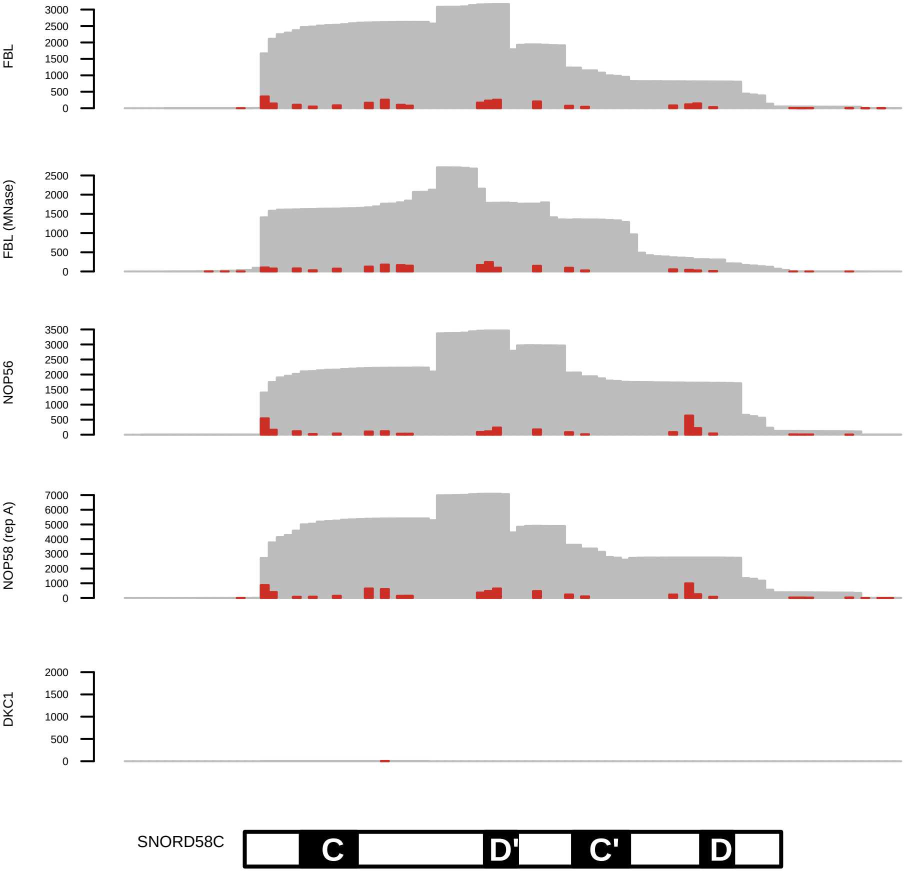
FBL
3000
2500
2000
1500
1000
500
0
FBL (MNase)
2500
2000
1500
1000
500
0
NOP56
3500
3000
2500
2000
1500
1000
500
0
NOP58 (rep A)
7000
6000
5000
4000
3000
2000
1000
0
DKC1
2000
1500
1000
500
0
SNORD58C
C
D'
C'
D

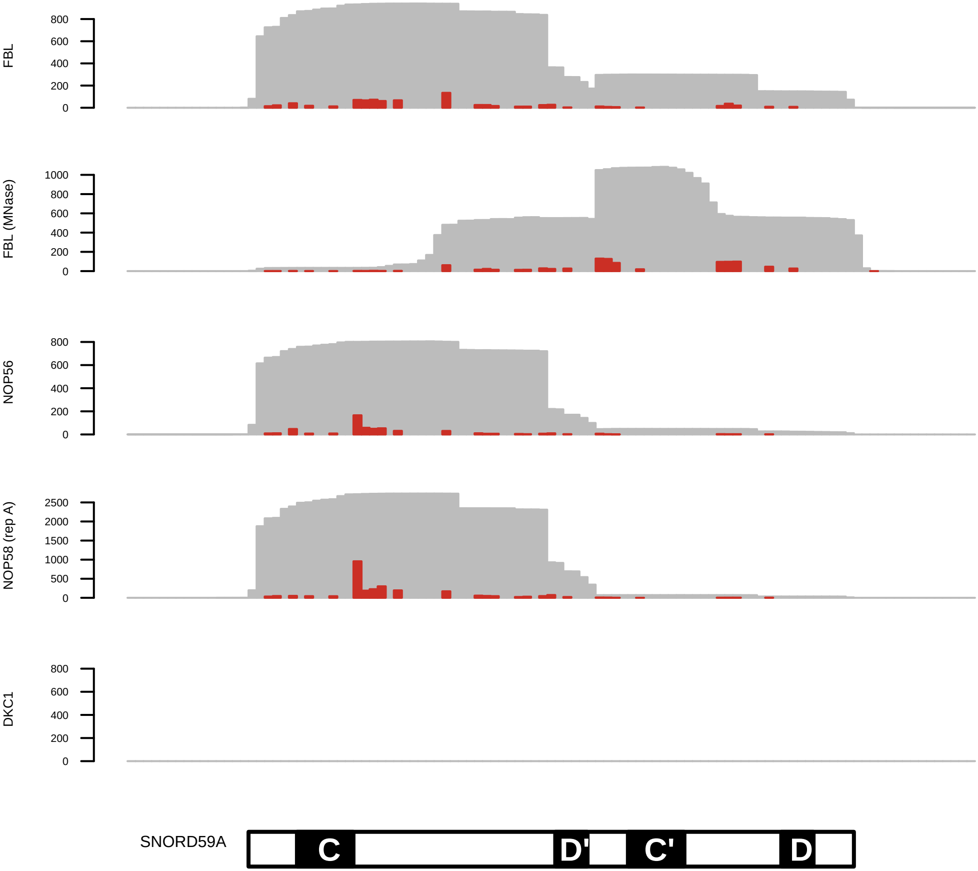

FBL
0
200
400
600
800
FBL (MNase)
0
200
400
600
800
1000
NOP56
0
200
400
600
800
NOP58 (rep A)
0
500
1000
1500
2000
2500
DKC1
0
200
400
600
800
SNORD59A
C
D'
C'
D

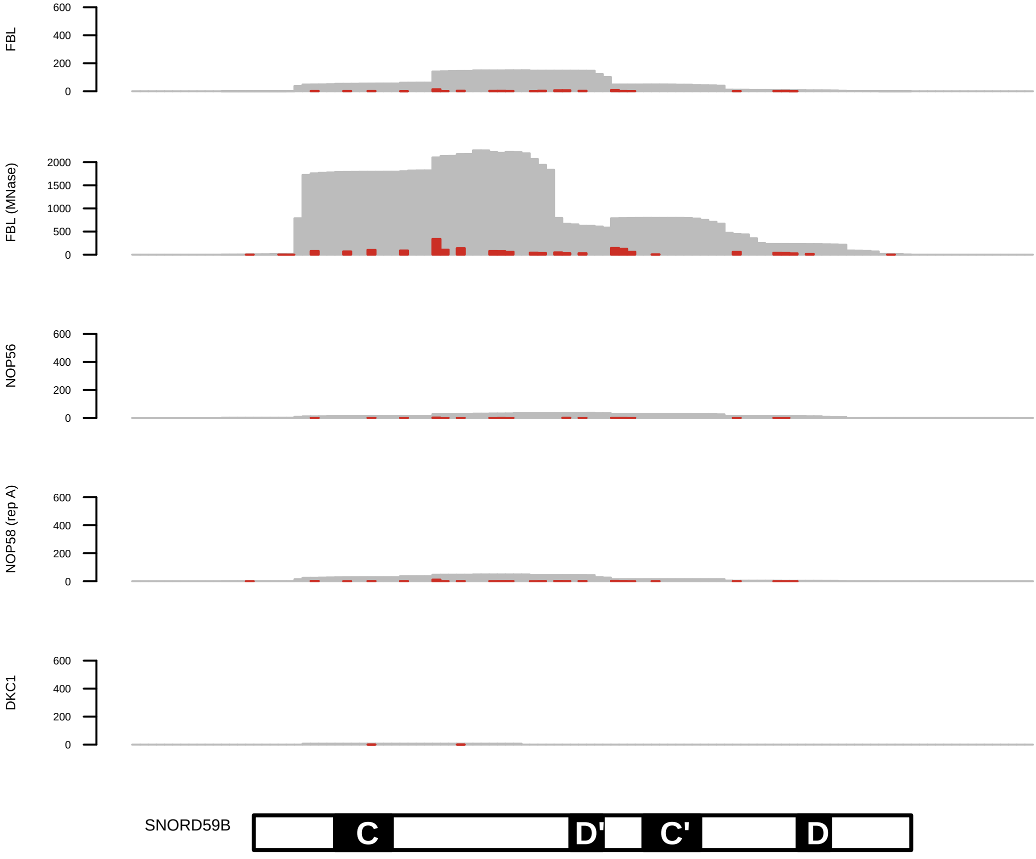

FBL
600
400
200
0
FBL (MNase)
2000
1500
1000
500
0
NOP56
600
400
200
0
NOP58 (rep A)
600
400
200
0
DKC1
600
400
200
0
SNORD59B
C
D'
C'
D

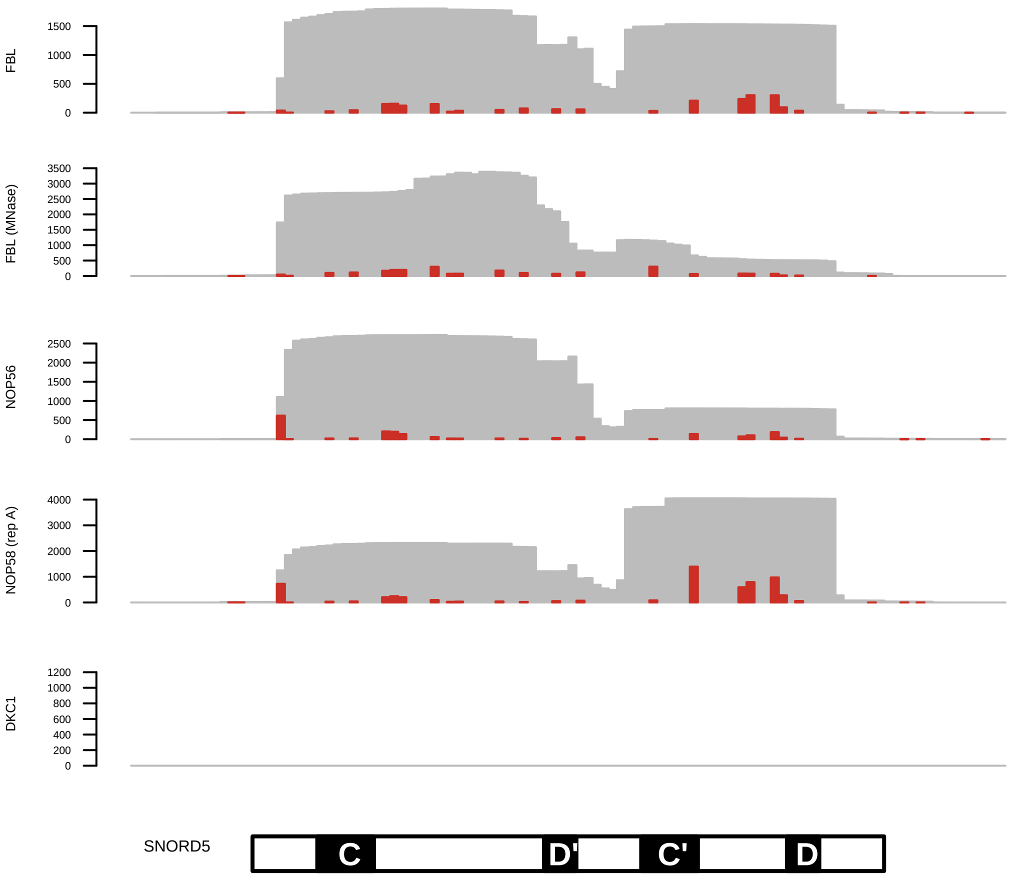
FBL
1500
1000
500
0
FBL (MNase)
3500
3000
2500
2000
1500
1000
500
0
NOP56
2500
2000
1500
1000
500
0
NOP58 (rep A)
4000
3000
2000
1000
0
DKC1
1200
1000
800
600
400
200
0
SNORD5
C
D'
C'
D

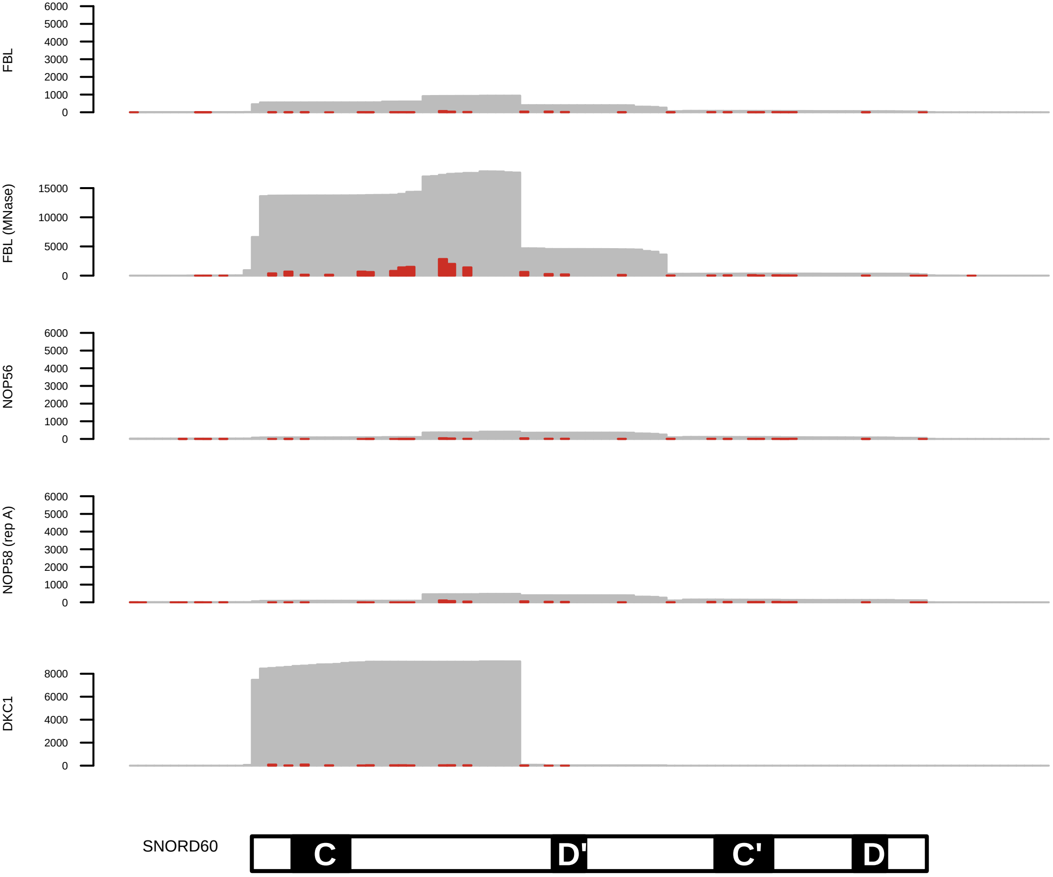
FBL
6000
5000
4000
3000
2000
1000
0
FBL (MNase)
15000
10000
5000
0
NOP56
6000
5000
4000
3000
2000
1000
0
NOP58 (rep A)
6000
5000
4000
3000
2000
1000
0
DKC1
8000
6000
4000
2000
0
SNORD60
C
D'
C'
D

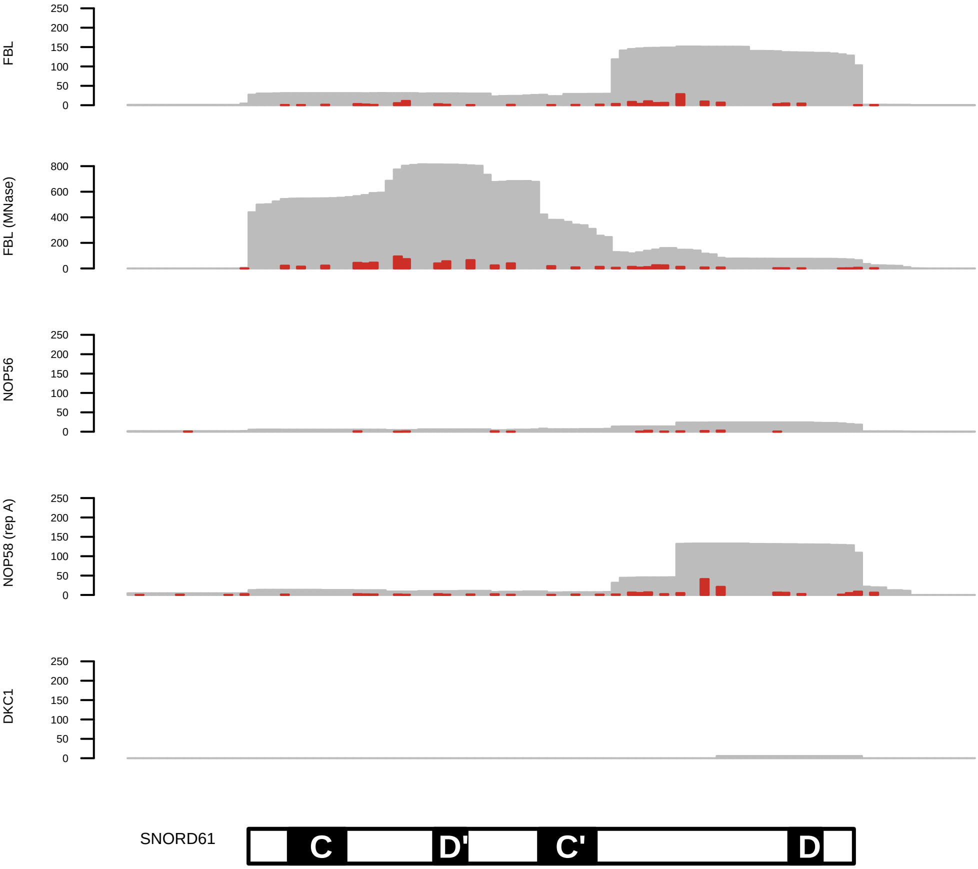

FBL
250
200
150
100
50
0
FBL (MNase)
800
600
400
200
0
NOP56
250
200
150
100
50
0
NOP58 (rep A)
250
200
150
100
50
0
DKC1
250
200
150
100
50
0
SNORD61
C
D'
C'
D

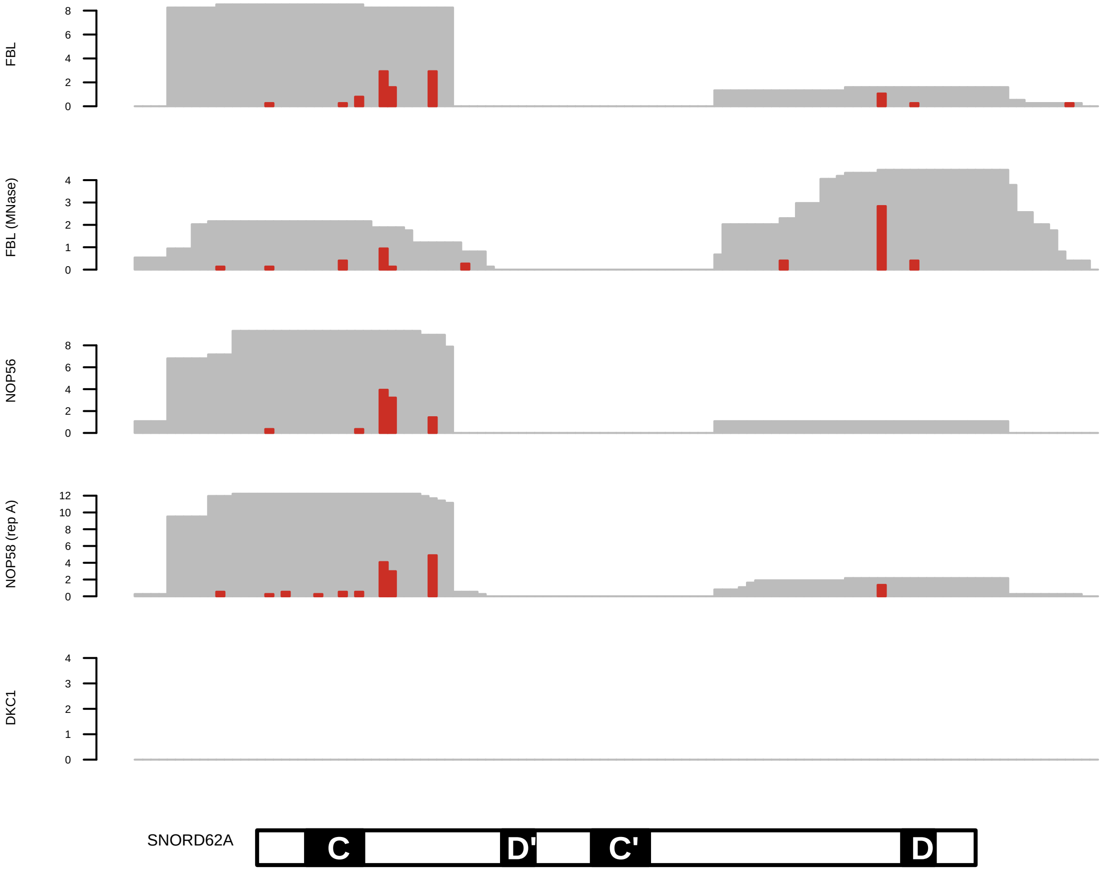
FBL
8
6
4
2
0
FBL (MNase)
4
3
2
1
0
NOP56
8
6
4
2
0
NOP58 (rep A)
12
10
8
6
4
2
0
DKC1
4
3
2
1
0
SNORD62A
C
D'
C'
D

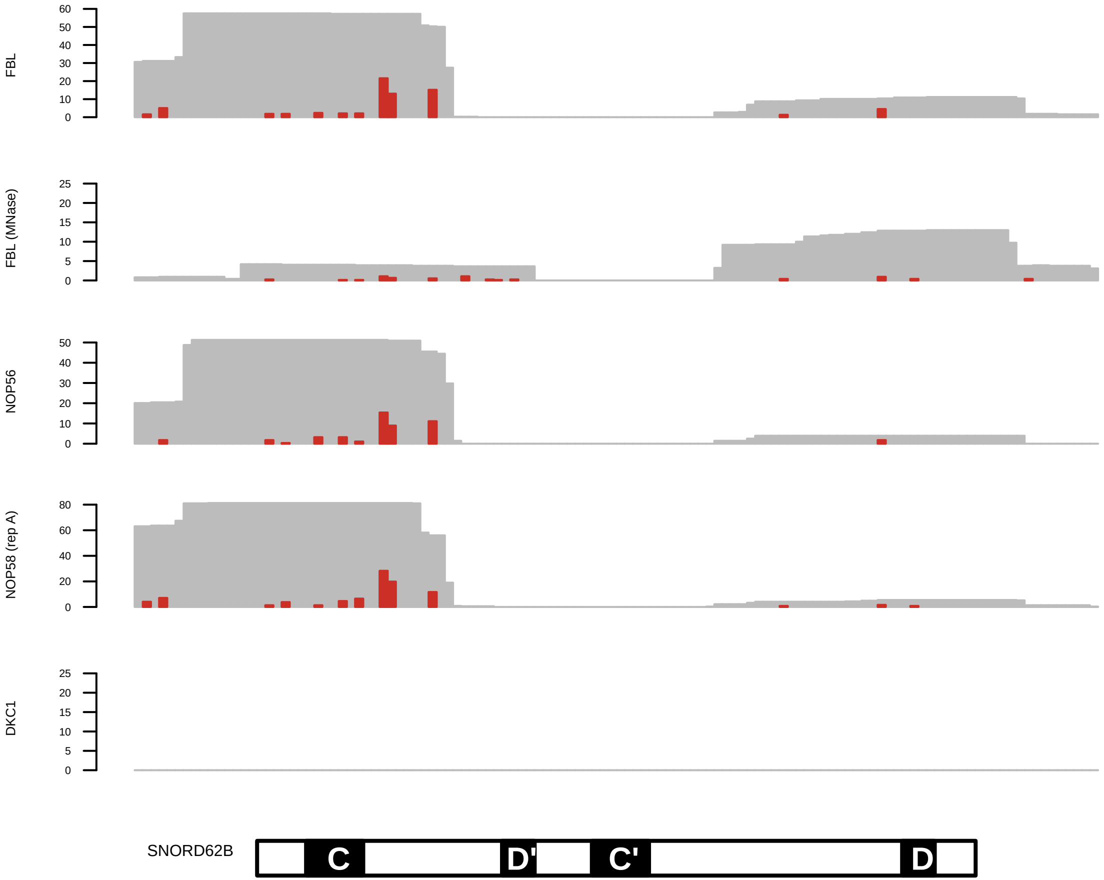
FBL
60
50
40
30
20
10
0
FBL (MNase)
25
20
15
10
5
0
NOP56
50
40
30
20
10
0
NOP58 (rep A)
80
60
40
20
0
DKC1
25
20
15
10
5
0
SNORD62B
C
D'
C'
D

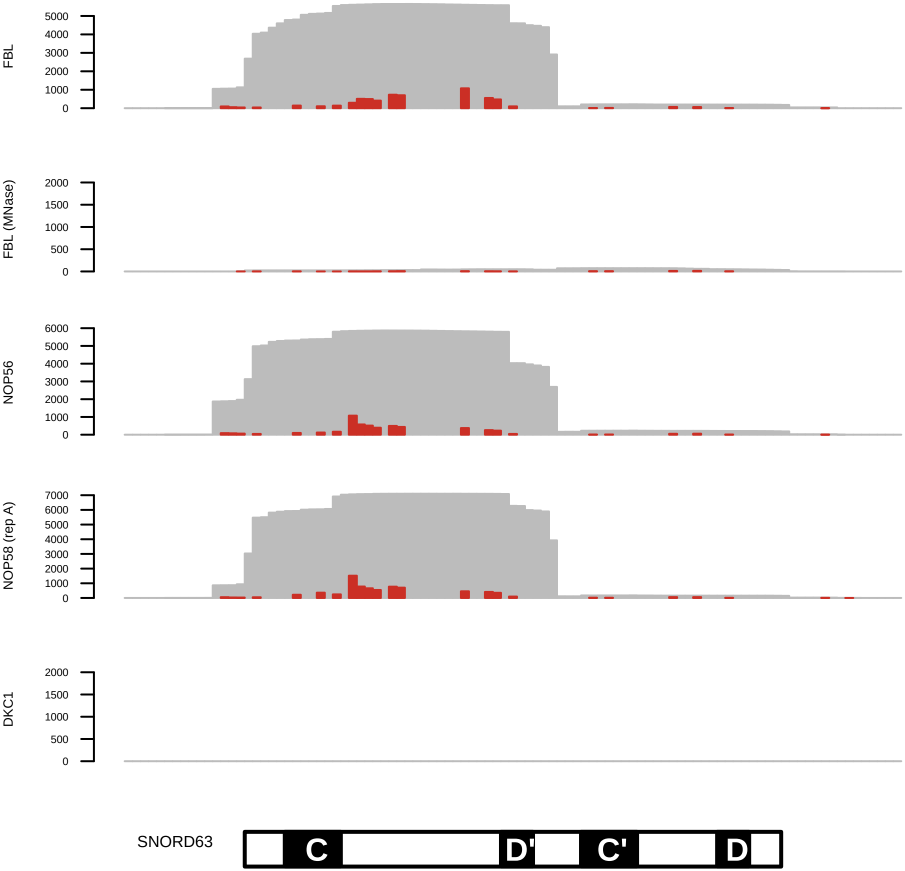
FBL
5000
4000
3000
2000
1000
0
FBL (MNase)
2000
1500
1000
500
0
NOP56
6000
5000
4000
3000
2000
1000
0
NOP58 (rep A)
7000
6000
5000
4000
3000
2000
1000
0
DKC1
2000
1500
1000
500
0
SNORD63
C
D'
C'
D

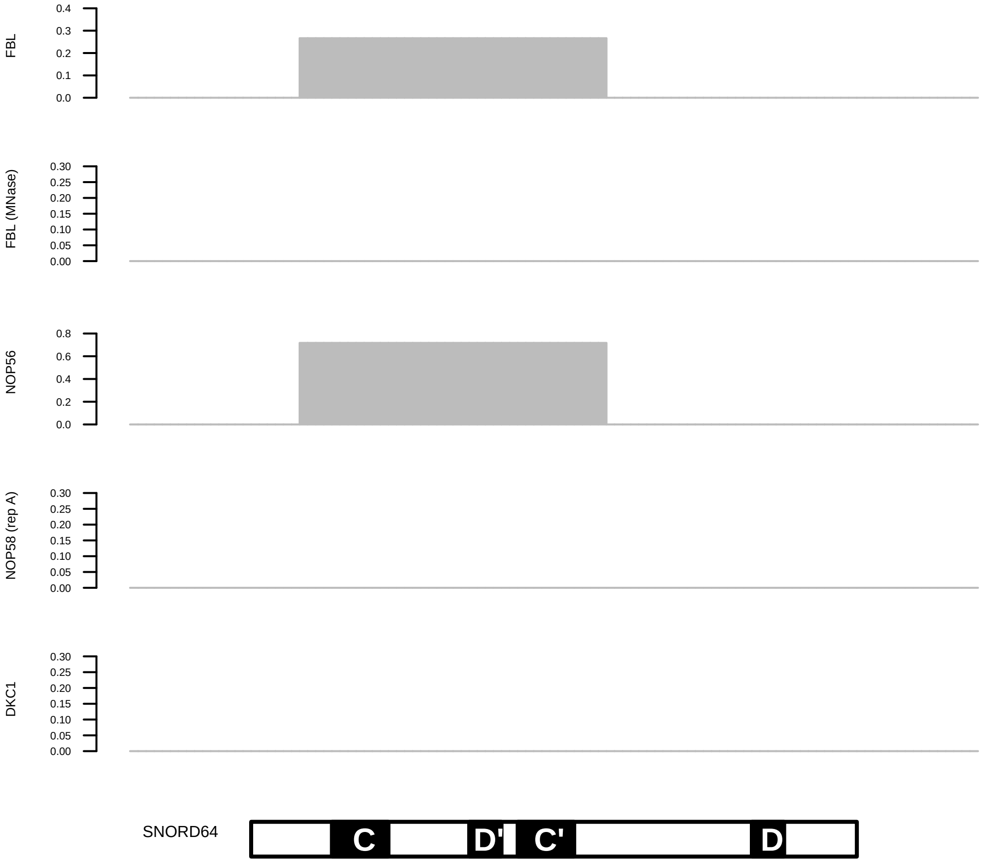
FBL
0.4
0.3
0.2
0.1
0.0
FBL (MNase)
0.30
0.25
0.20
0.15
0.10
0.05
0.00
NOP56
0.8
0.6
0.4
0.2
0.0
NOP58 (rep A)
0.30
0.25
0.20
0.15
0.10
0.05
0.00
DKC1
0.30
0.25
0.20
0.15
0.10
0.05
0.00
SNORD64
C
D'
C'
D

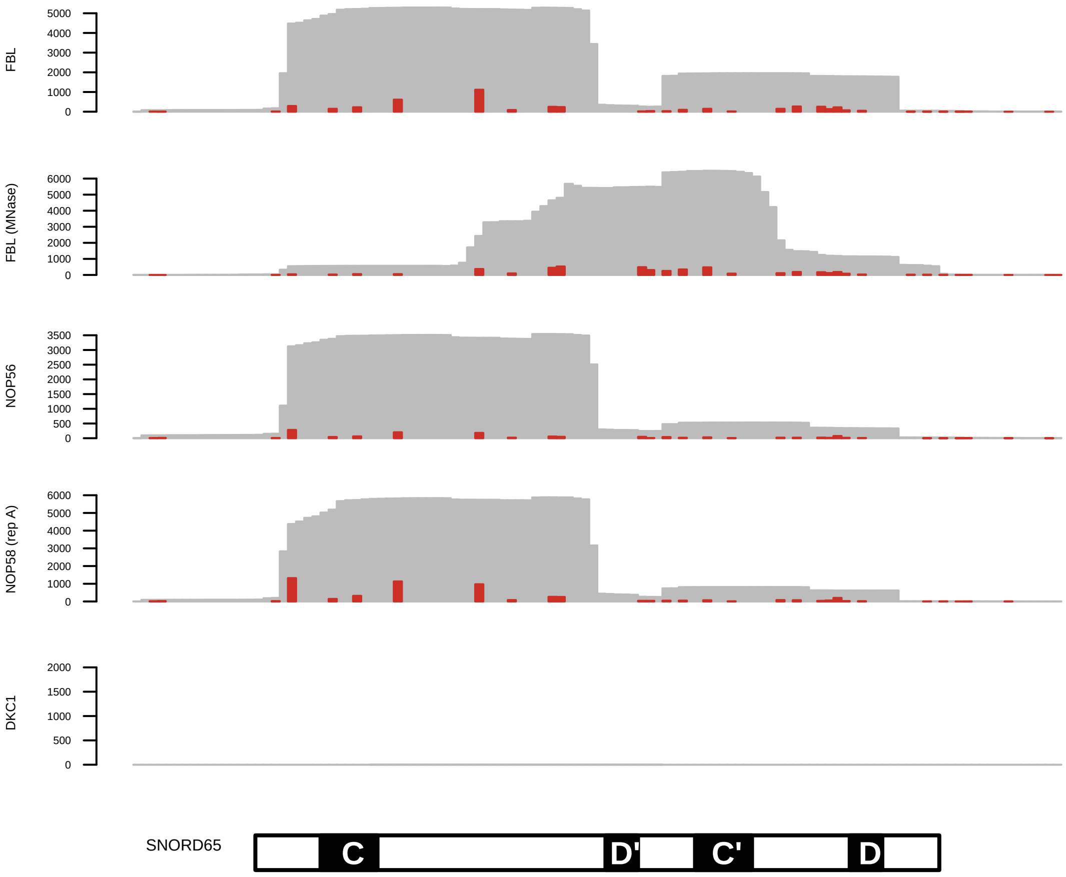
FBL
FBL (MNase)
NOP56
NOP58 (rep A)
DKC1
SNORD65
C
D'
C'
D

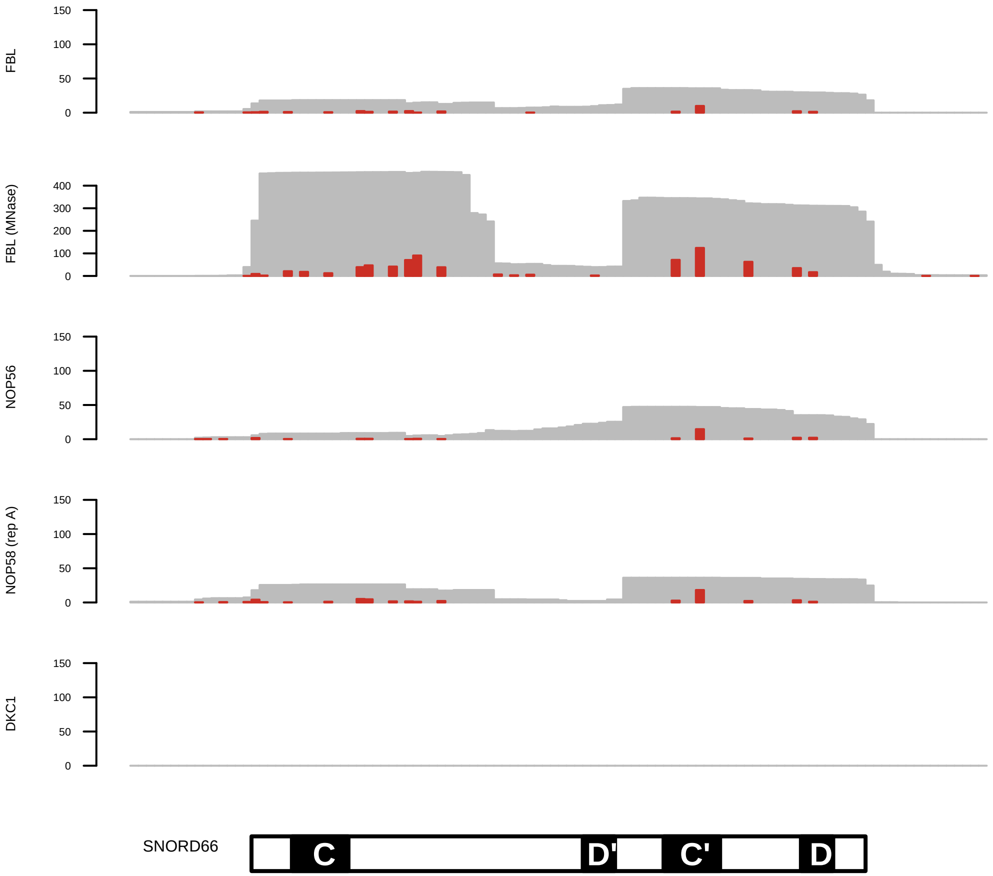

FBL
150
100
50
0
FBL (MNase)
400
300
200
100
0
NOP56
150
100
50
0
NOP58 (rep A)
150
100
50
0
DKC1
150
100
50
0
SNORD66
C
D'
C'
D

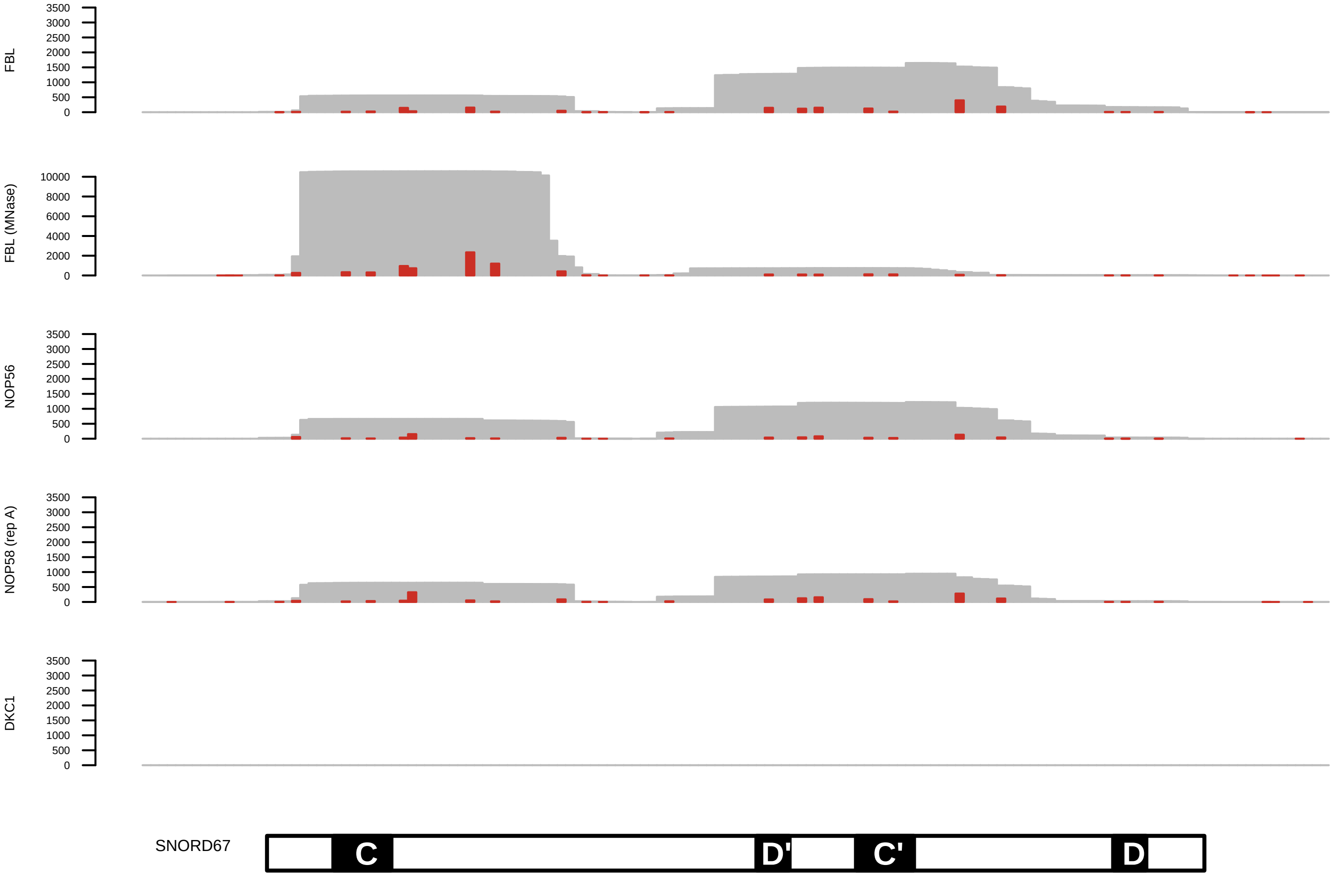
FBL
FBL (MNase)
NOP56
NOP58 (rep A)
DKC1
3500
3000
2500
2000
1500
1000
500
0
10000
8000
6000
4000
2000
SNORD67
C
D'
C'
D

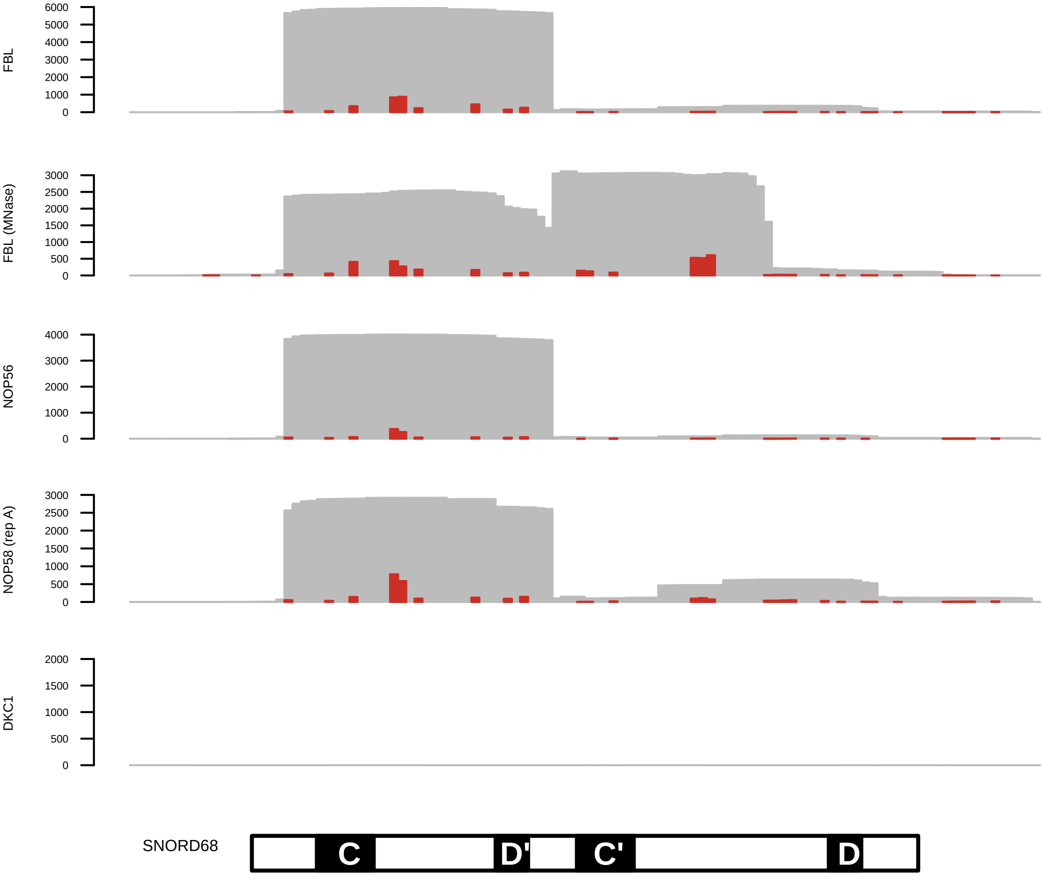

FBL
6000
5000
4000
3000
2000
1000
0
FBL (MNase)
3000
2500
2000
1500
1000
500
0
NOP56
4000
3000
2000
1000
0
NOP58 (rep A)
3000
2500
2000
1500
1000
500
0
DKC1
2000
1500
1000
500
0
SNORD68
C
D'
C'
D

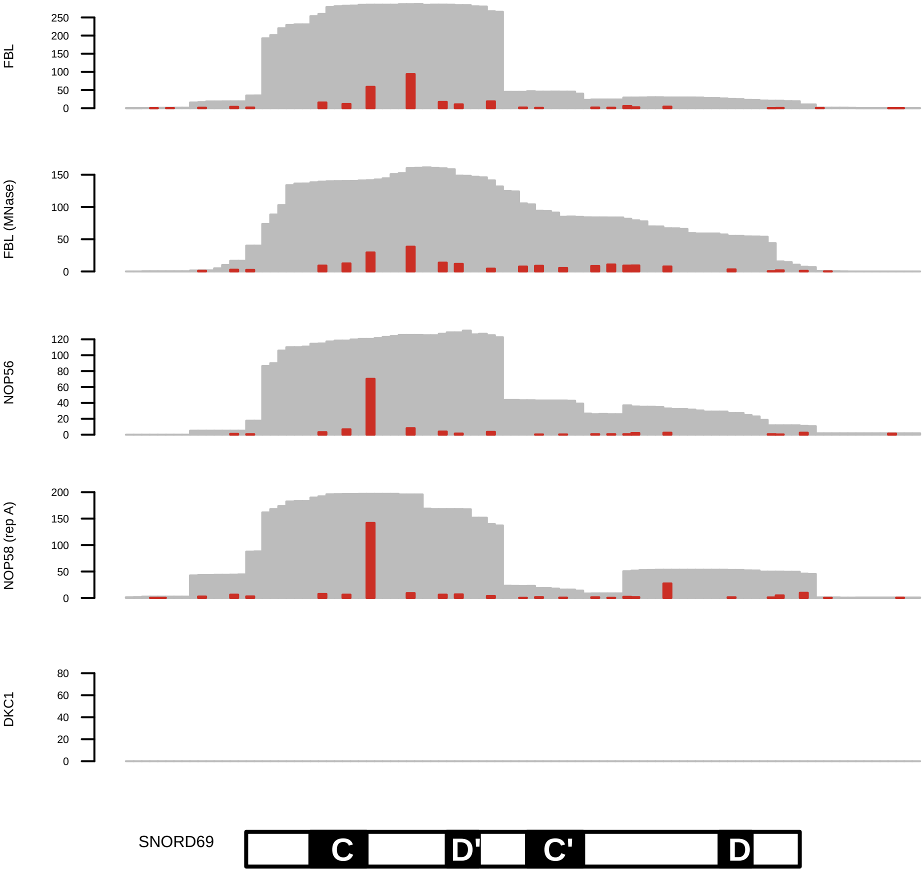
FBL
250
200
150
100
50
0
FBL (MNase)
150
100
50
0
NOP56
120
100
80
60
40
20
0
NOP58 (rep A)
200
150
100
50
0
DKC1
80
60
40
20
0
SNORD69
C
D'
C'
D

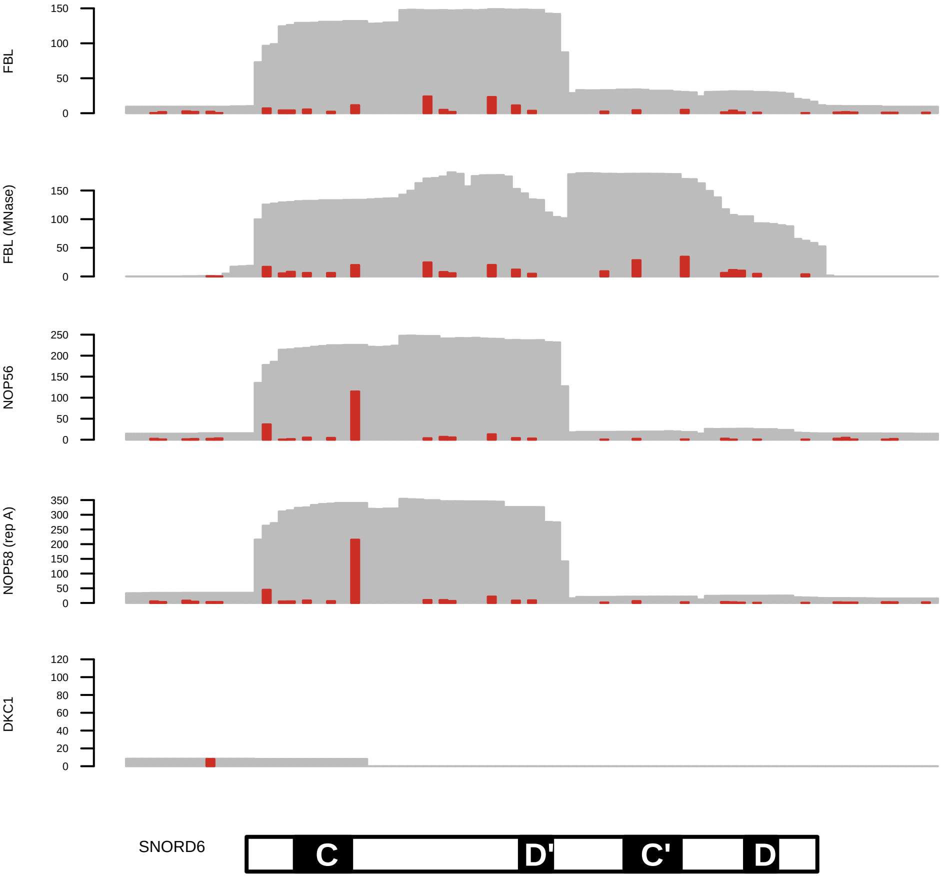
FBL
150
100
50
0
FBL (MNase)
150
100
50
0
NOP56
250
200
150
100
50
0
NOP58 (rep A)
350
300
250
200
150
100
50
0
DKC1
120
100
80
60
40
20
0
SNORD6
C
D'
C'
D

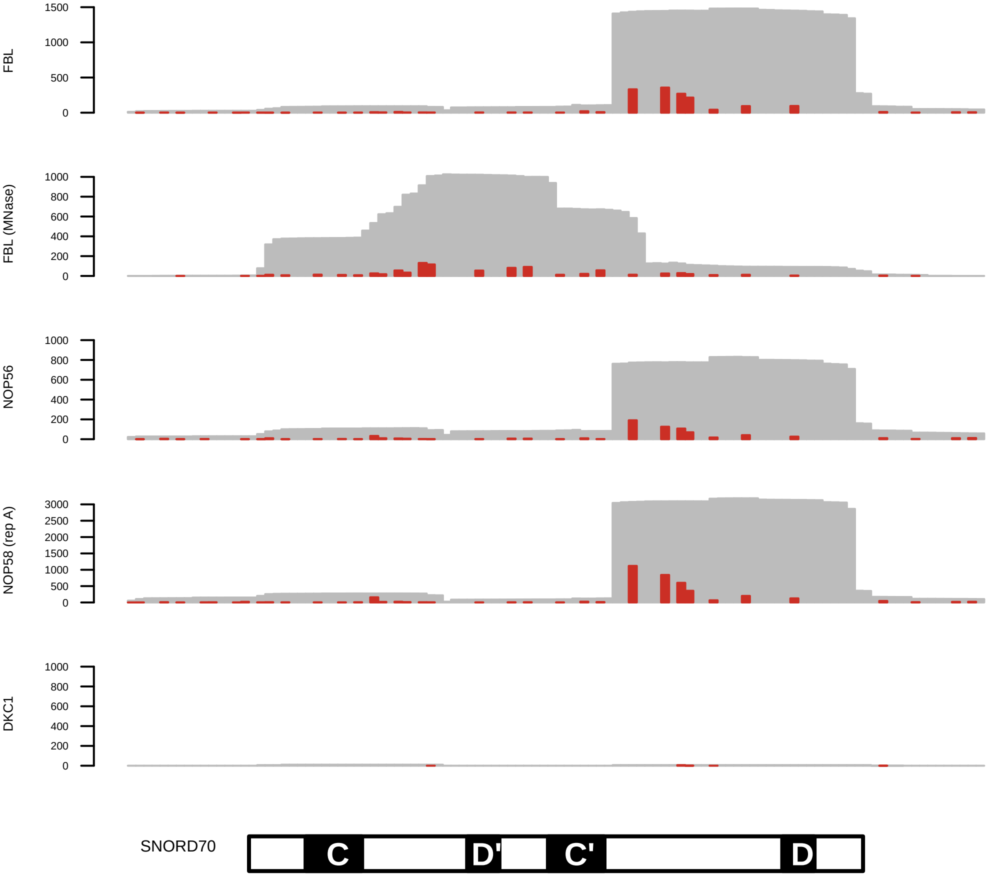
FBL
1500
1000
500
0
FBL (MNase)
1000
800
600
400
200
0
NOP56
1000
800
600
400
200
0
NOP58 (rep A)
3000
2500
2000
1500
1000
500
0
DKC1
1000
800
600
400
200
0
SNORD70
C
D'
C'
D

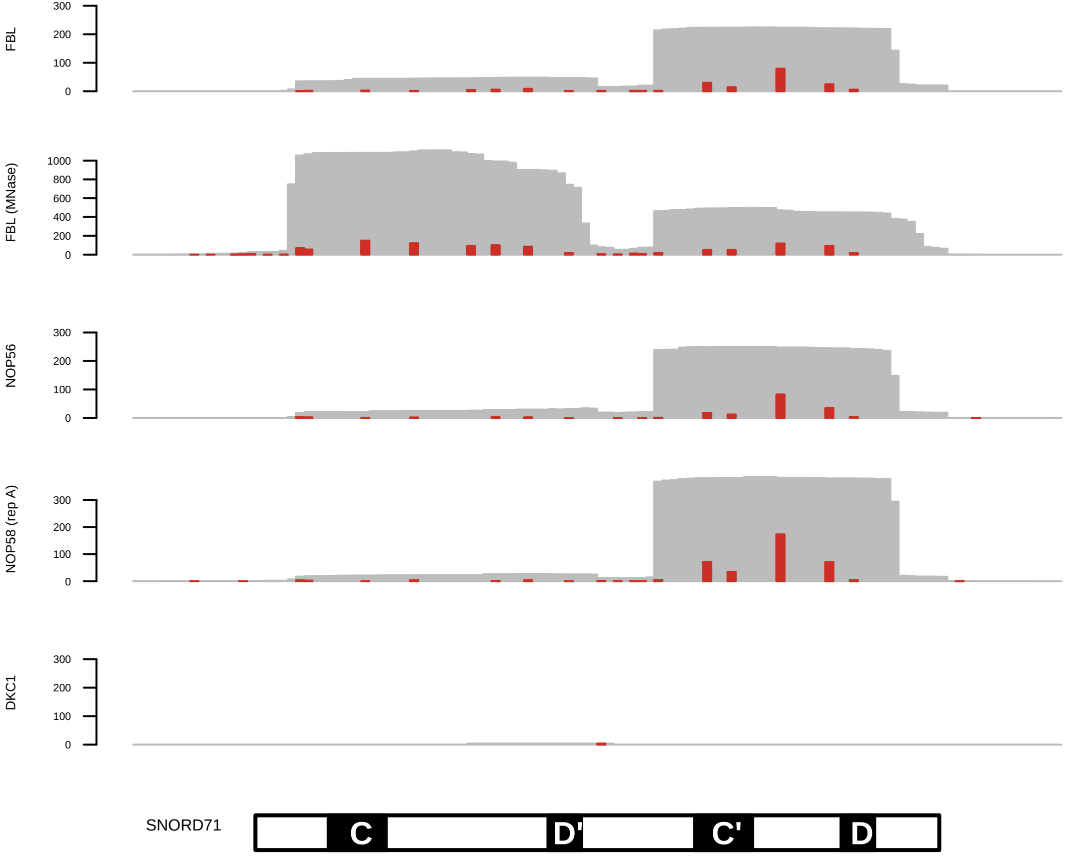
FBL
300
200
100
0
FBL (MNase)
1000
800
600
400
200
0
NOP56
300
200
100
0
NOP58 (rep A)
300
200
100
0
DKC1
300
200
100
0
SNORD71
C
D'
C'
D

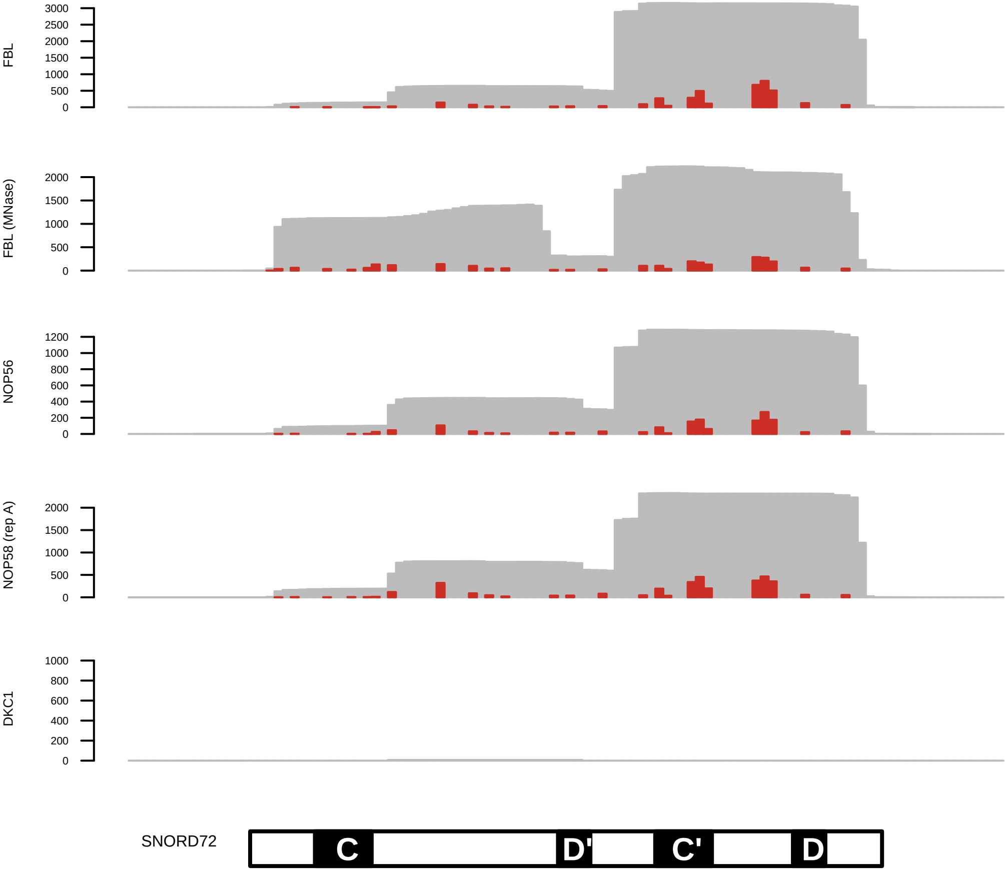

FBL
3000
2500
2000
1500
1000
500
0
FBL (MNase)
2000
1500
1000
500
0
NOP56
1200
1000
800
600
400
200
0
NOP58 (rep A)
2000
1500
1000
500
0
DKC1
1000
800
600
400
200
0
SNORD72
C
D'
C'
D

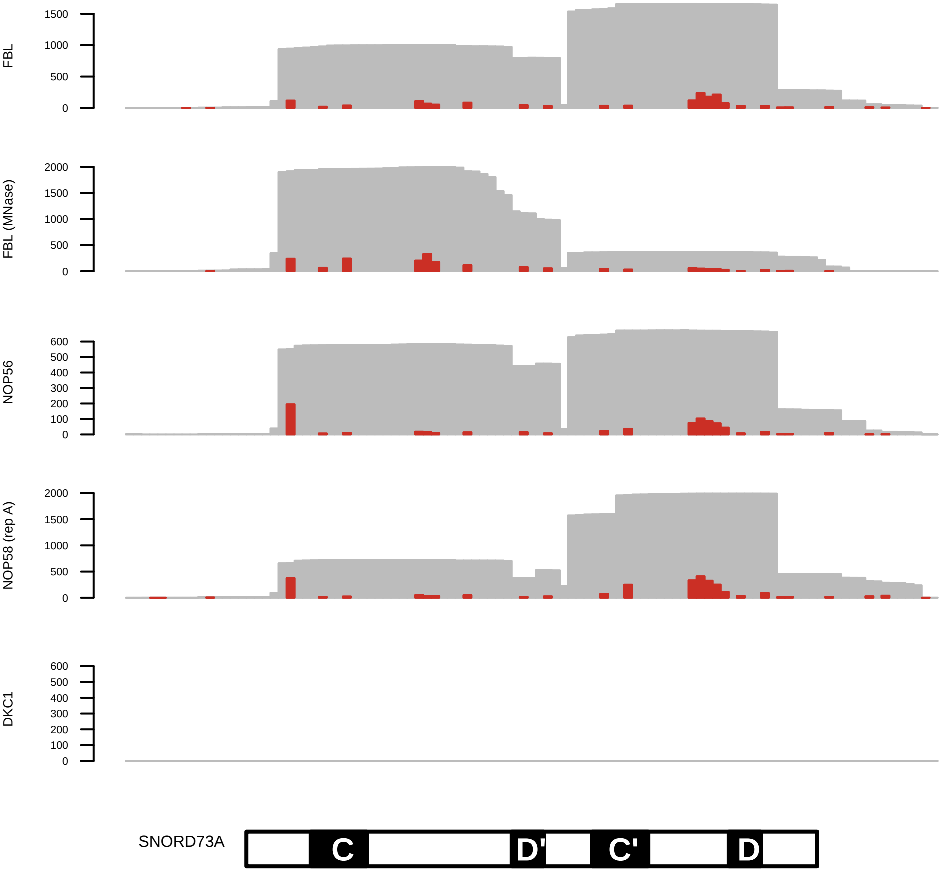
FBL
0
500
1000
1500
FBL (MNase)
0
500
1000
1500
2000
NOP56
0
100
200
300
400
500
600
NOP58 (rep A)
0
500
1000
1500
2000
DKC1
0
100
200
300
400
500
600
SNORD73A
C
D'
C'
D

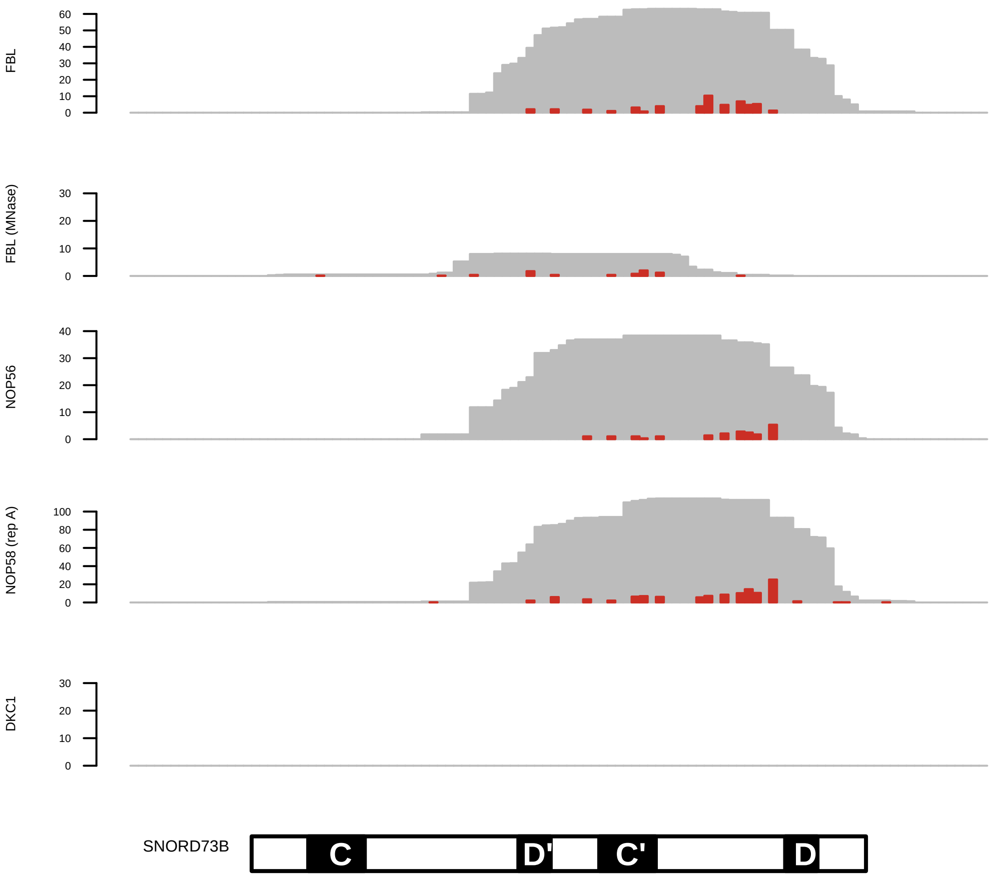
FBL
60
50
40
30
20
10
0
FBL (MNase)
30
20
10
0
NOP56
40
30
20
10
0
NOP58 (rep A)
100
80
60
40
20
0
DKC1
30
20
10
0
SNORD73B
C
D'
C'
D

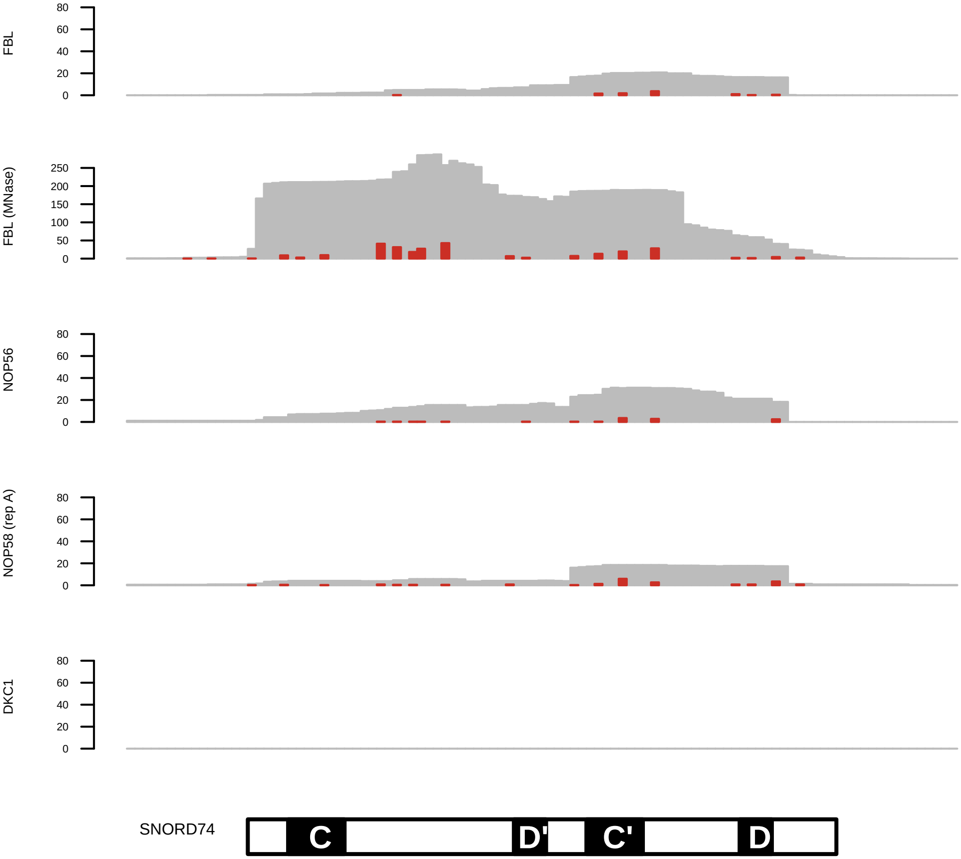
FBL
FBL (MNase)
NOP56
NOP58 (rep A)
DKC1
0
20
40
60
80
50
100
150
200
250
SNORD74
C
D'
C'
D

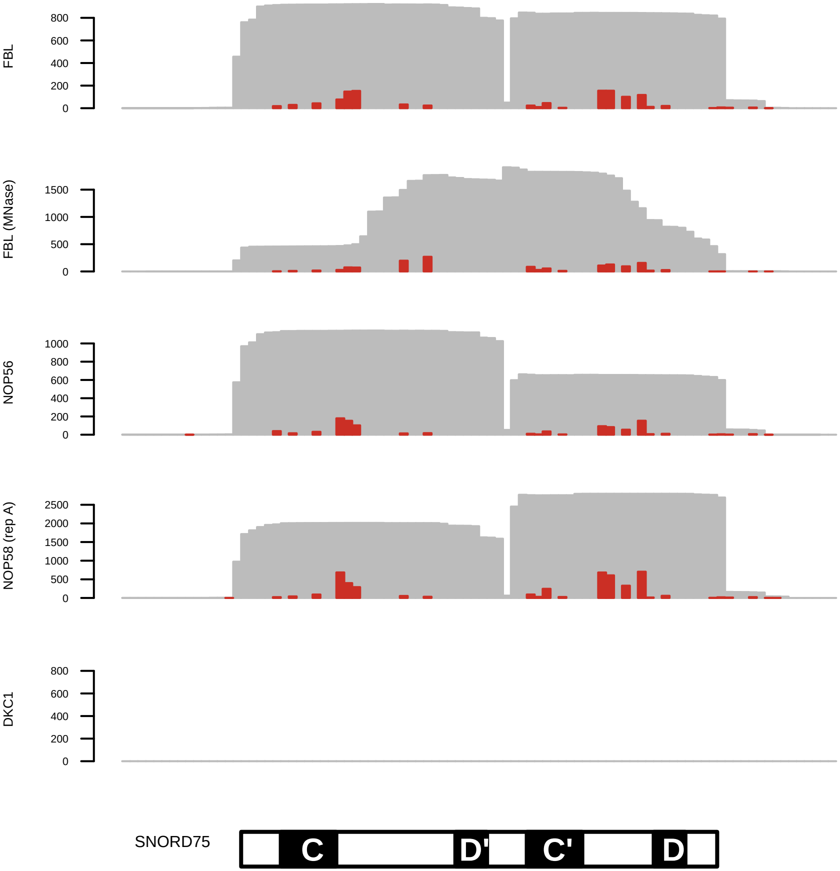
FBL
800
600
400
200
0
FBL (MNase)
1500
1000
500
0
NOP56
1000
800
600
400
200
0
NOP58 (rep A)
2500
2000
1500
1000
500
0
DKC1
800
600
400
200
0
SNORD75
C
D'
C'
D

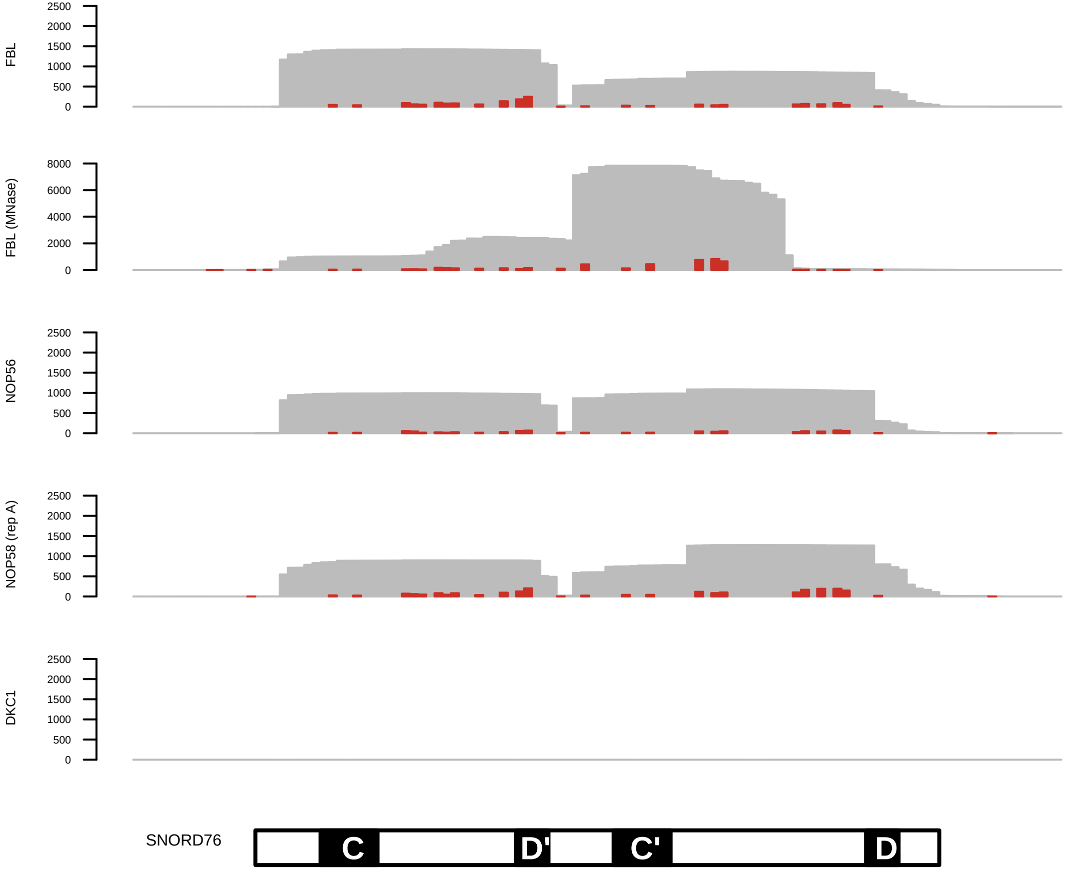
FBL
FBL (MNase)
NOP56
NOP58 (rep A)
DKC1
SNORD76
C
D'
C'
D

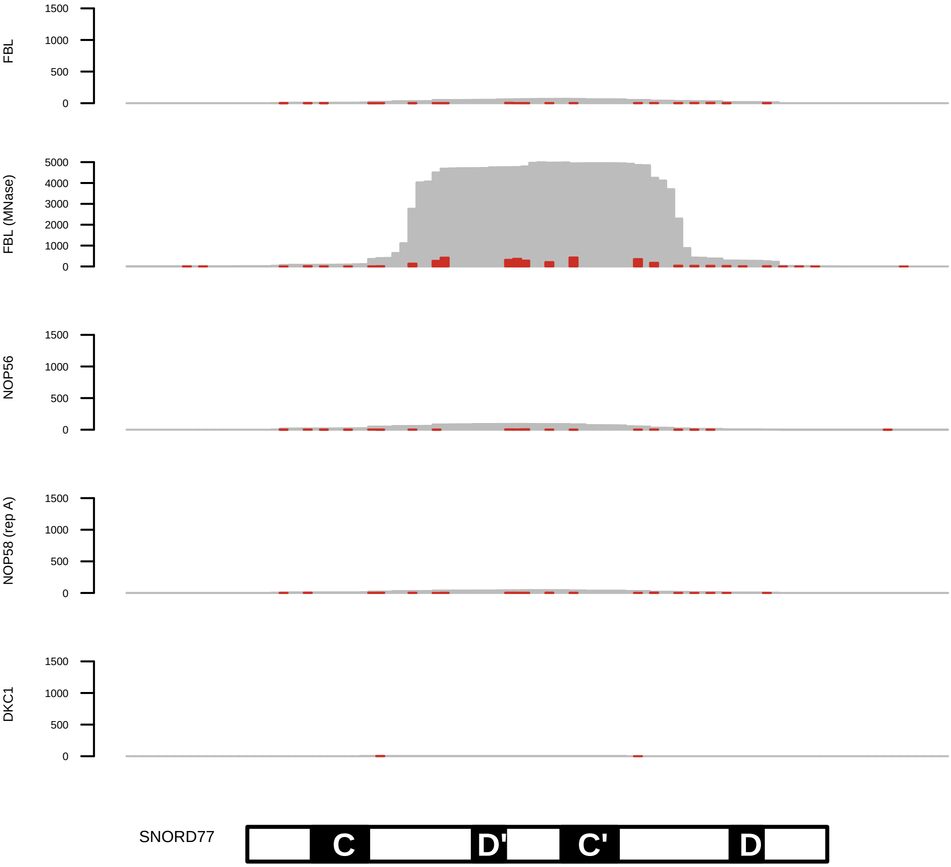

FBL
1500
1000
500
0
FBL (MNase)
5000
4000
3000
2000
1000
0
NOP56
1500
1000
500
0
NOP58 (rep A)
1500
1000
500
0
DKC1
1500
1000
500
0
SNORD77
C
D'
C'
D

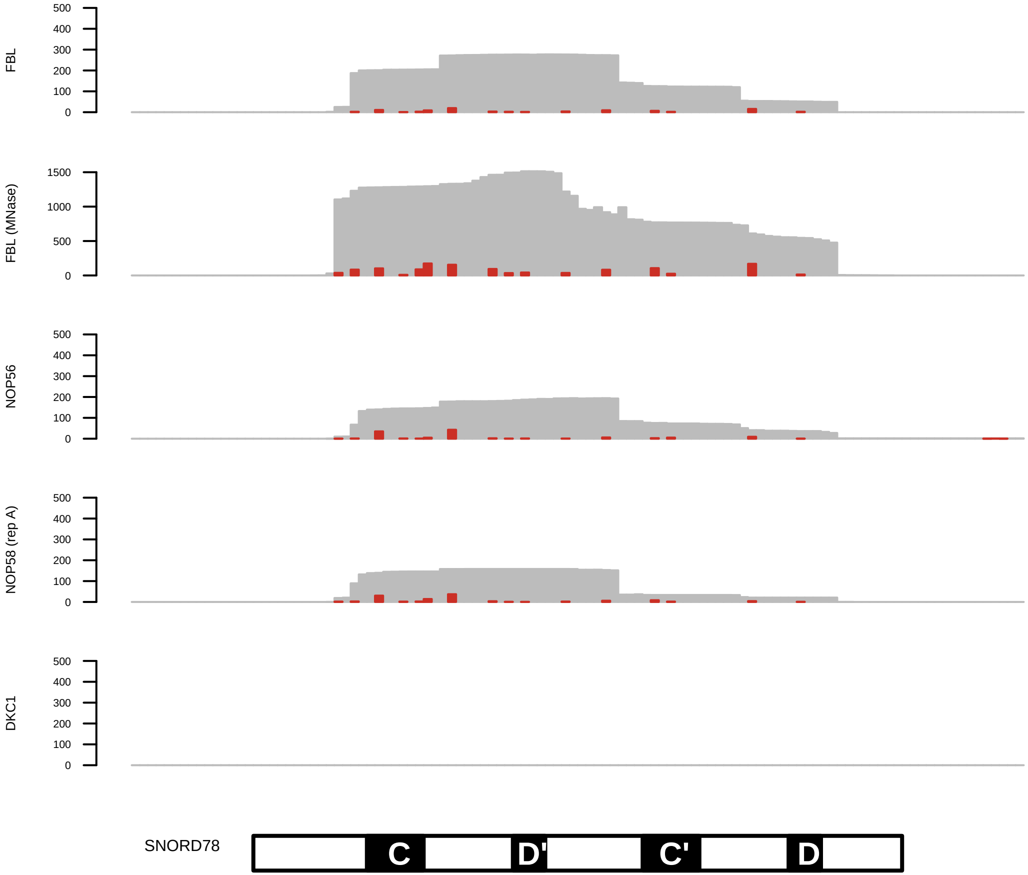
FBL
500
400
300
200
100
0
FBL (MNase)
1500
1000
500
0
NOP56
500
400
300
200
100
0
NOP58 (rep A)
500
400
300
200
100
0
DKC1
500
400
300
200
100
0
SNORD78
C
D'
C'
D

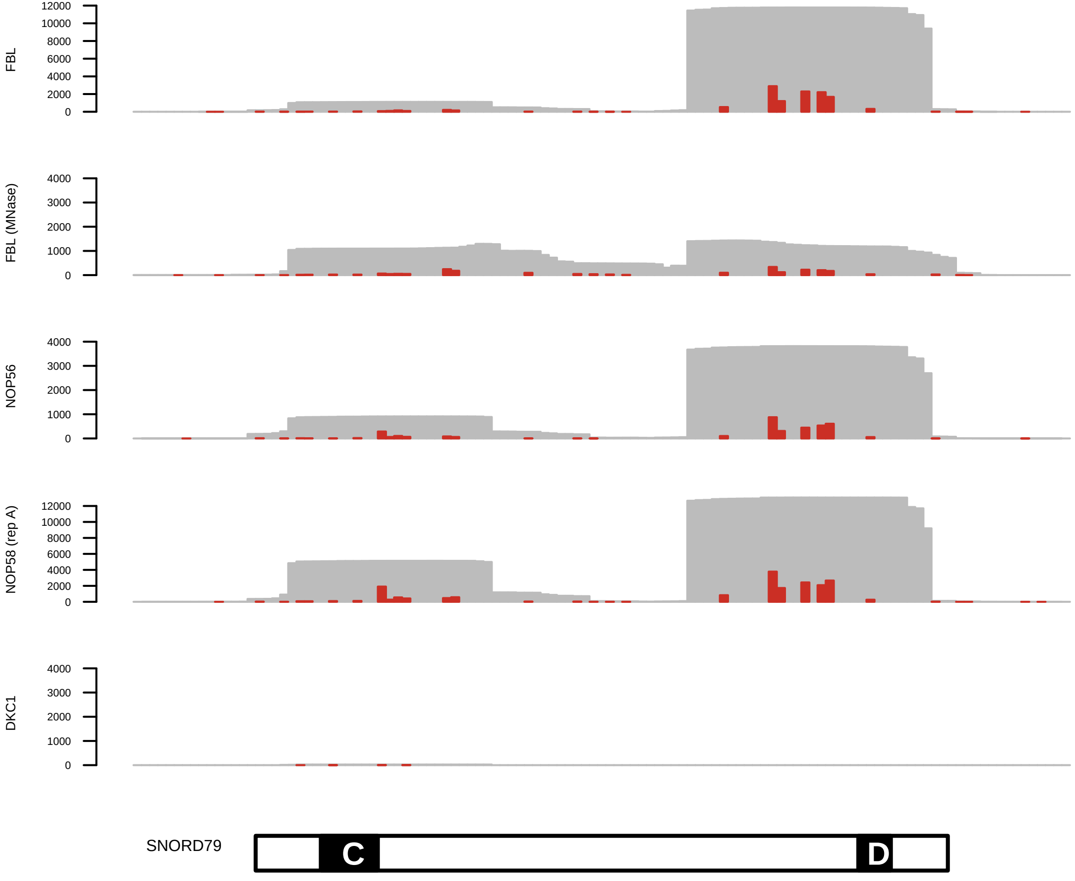
FBL
FBL (MNase)
NOP56
NOP58 (rep A)
DKC1
12000
10000
8000
6000
4000
2000
0
4000
3000
2000
1000
SNORD79
C
D

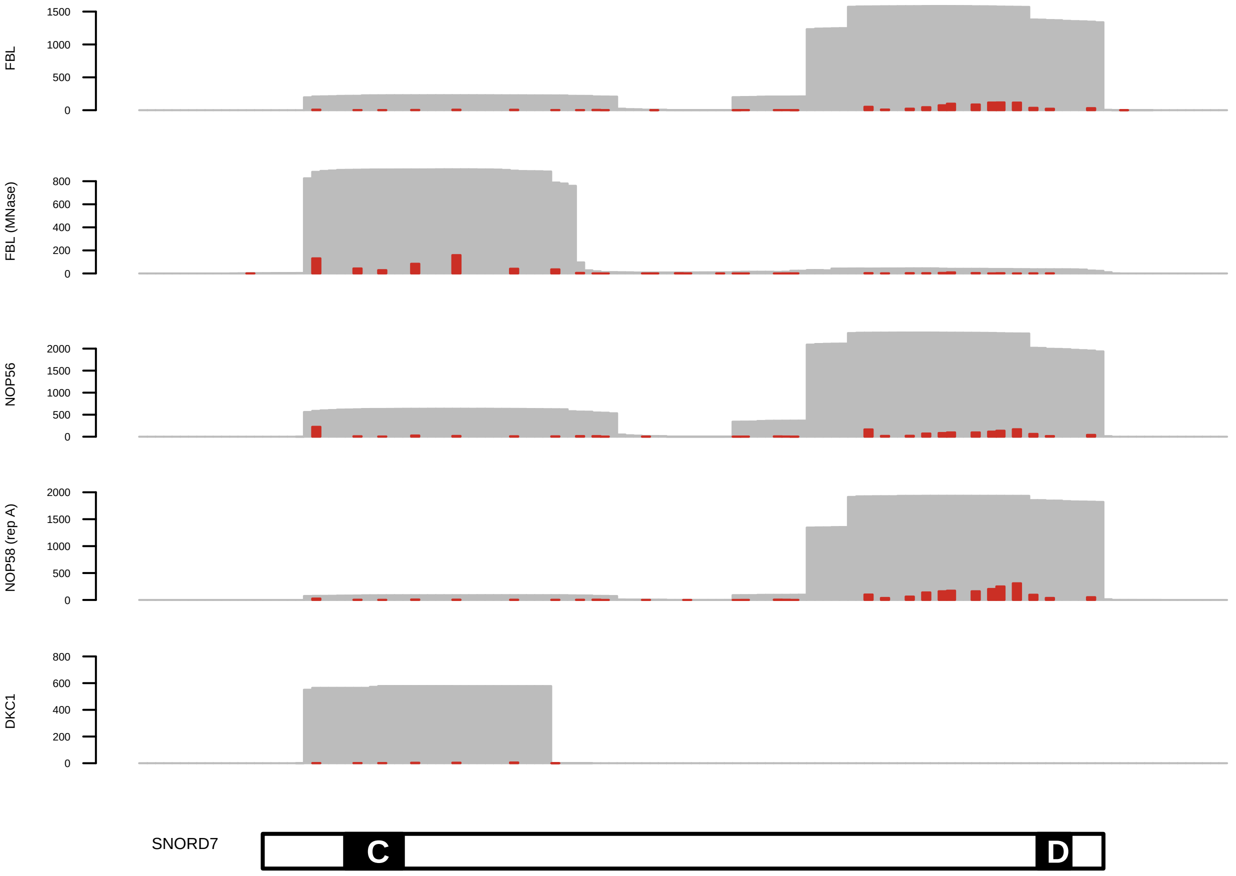
FBL
1500
1000
500
0
FBL (MNase)
800
600
400
200
0
NOP56
2000
1500
1000
500
0
NOP58 (rep A)
2000
1500
1000
500
0
DKC1
800
600
400
200
0
SNORD7
C
D

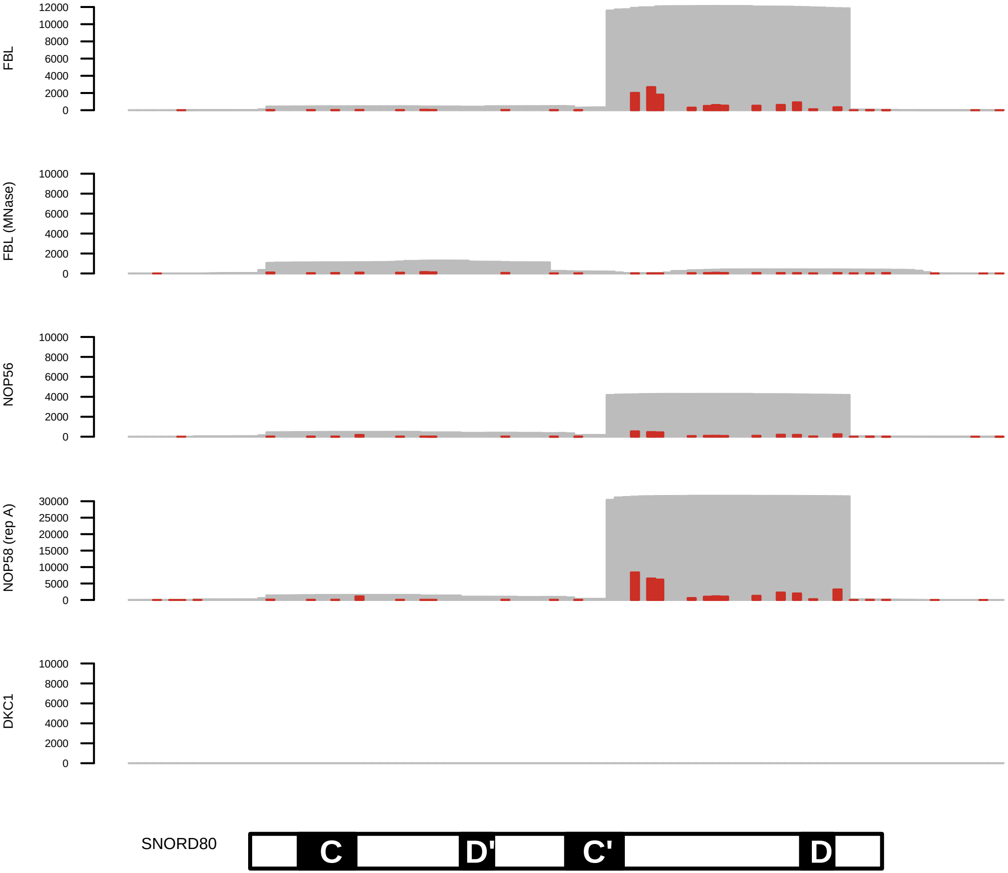
FBL
12000
10000
8000
6000
4000
2000
0
FBL (MNase)
10000
8000
6000
4000
2000
0
NOP56
10000
8000
6000
4000
2000
0
NOP58 (rep A)
30000
25000
20000
15000
10000
5000
0
DKC1
10000
8000
6000
4000
2000
0
SNORD80
C
D'
C'
D

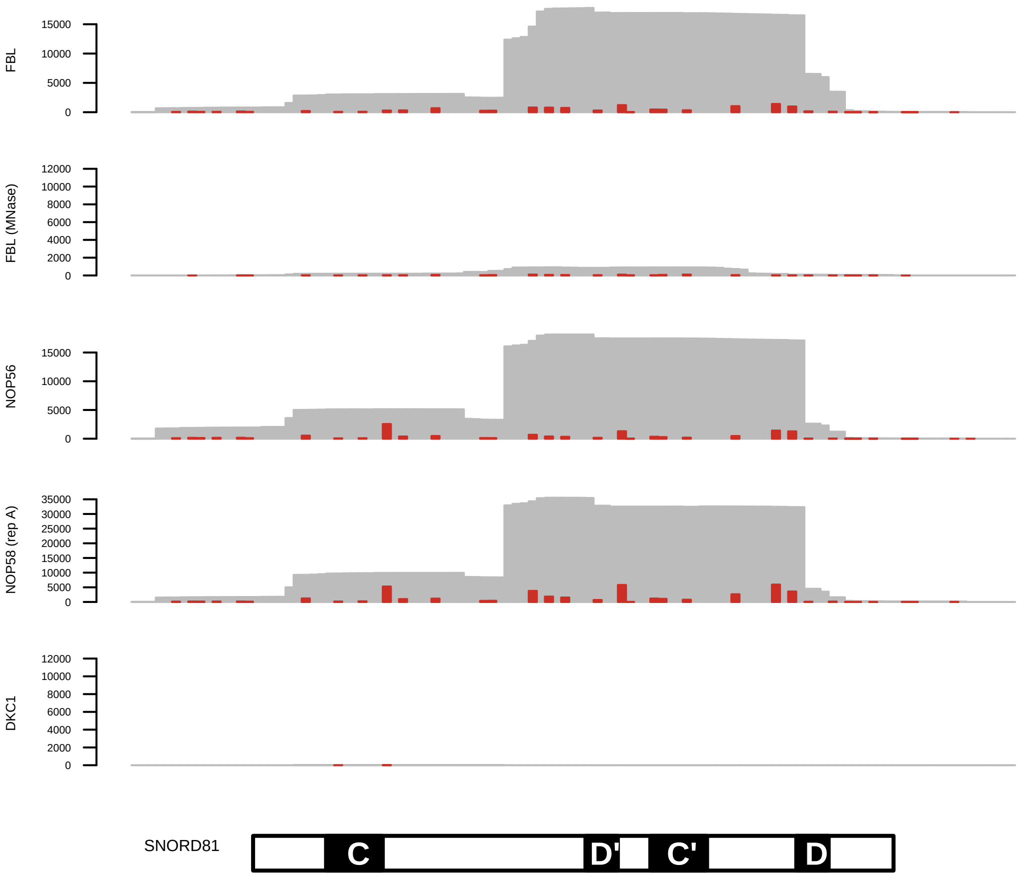

FBL
15000
10000
5000
0
FBL (MNase)
12000
10000
8000
6000
4000
2000
0
NOP56
15000
10000
5000
0
NOP58 (rep A)
35000
30000
25000
20000
15000
10000
5000
0
DKC1
12000
10000
8000
6000
4000
2000
0
SNORD81
C
D'
C'
D

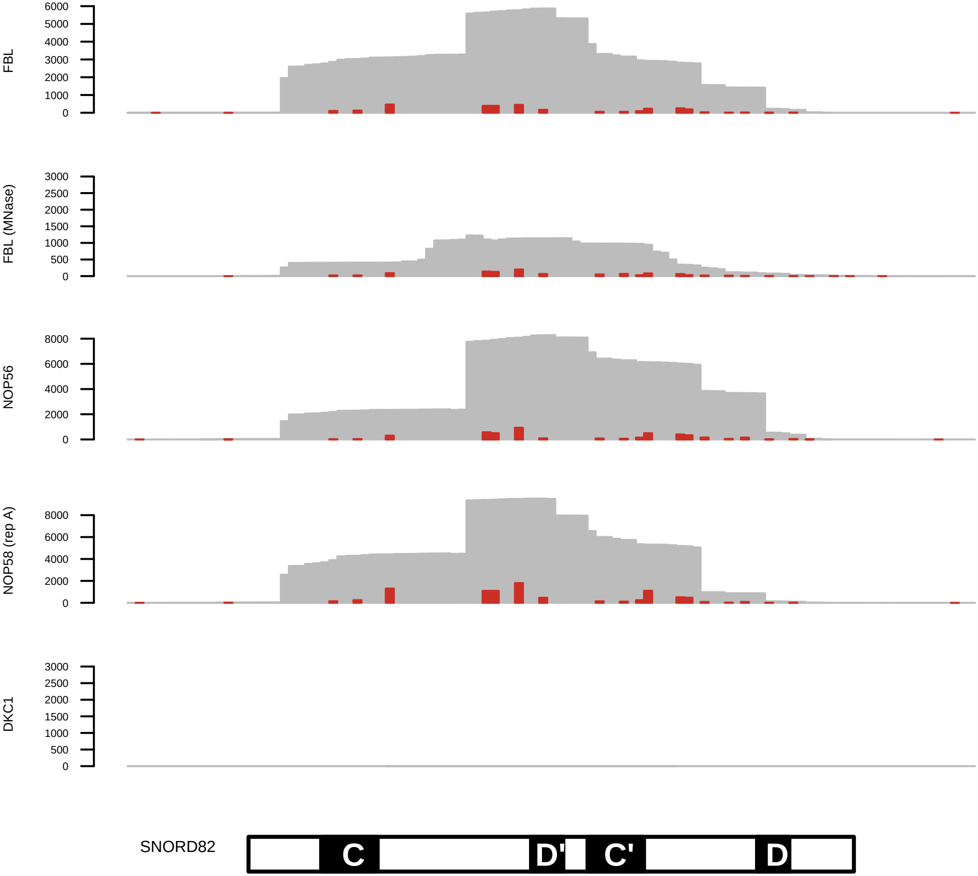

FBL
6000
5000
4000
3000
2000
1000
0
FBL (MNase)
3000
2500
2000
1500
1000
500
0
NOP56
8000
6000
4000
2000
0
NOP58 (rep A)
8000
6000
4000
2000
0
DKC1
3000
2500
2000
1500
1000
500
0
SNORD82
C
D'
C'
D

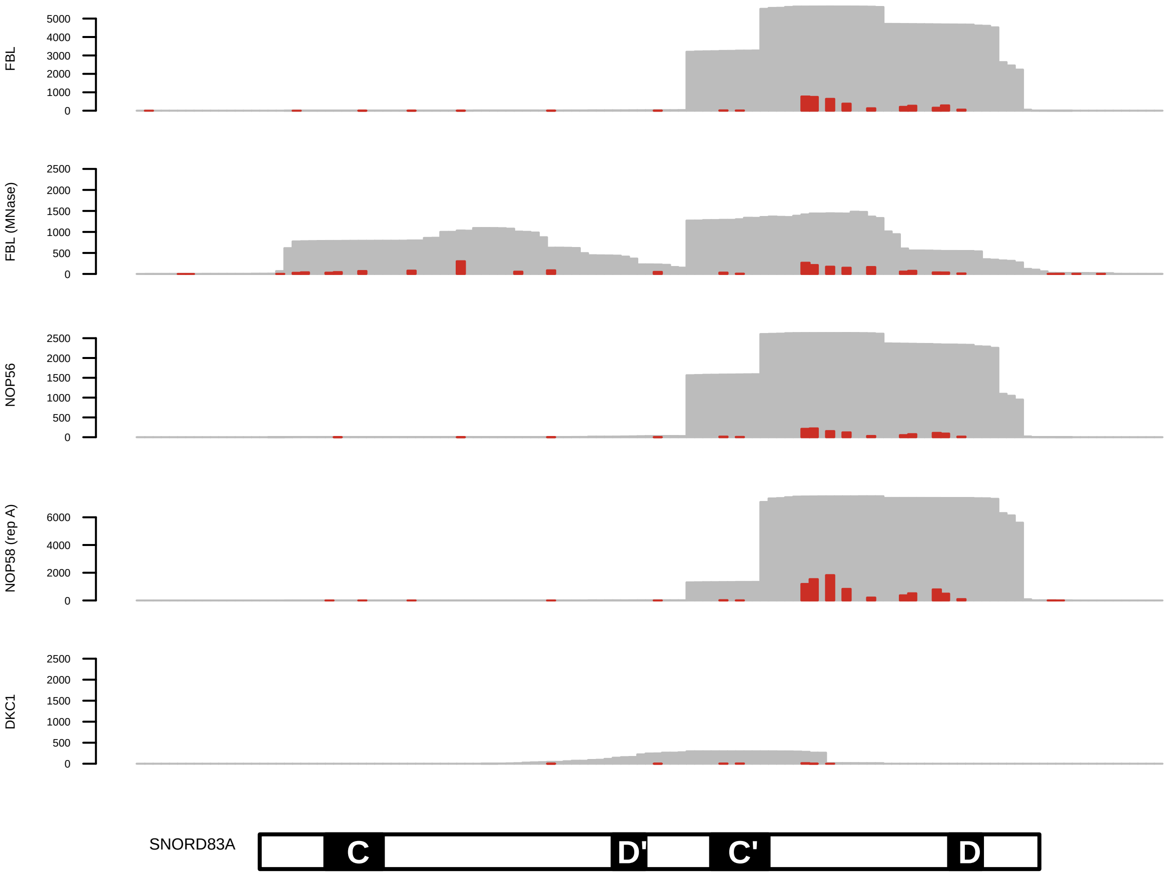
FBL
5000
4000
3000
2000
1000
0
FBL (MNase)
2500
2000
1500
1000
500
0
NOP56
2500
2000
1500
1000
500
0
NOP58 (rep A)
6000
4000
2000
0
DKC1
2500
2000
1500
1000
500
0
SNORD83A
C
D'
C'
D

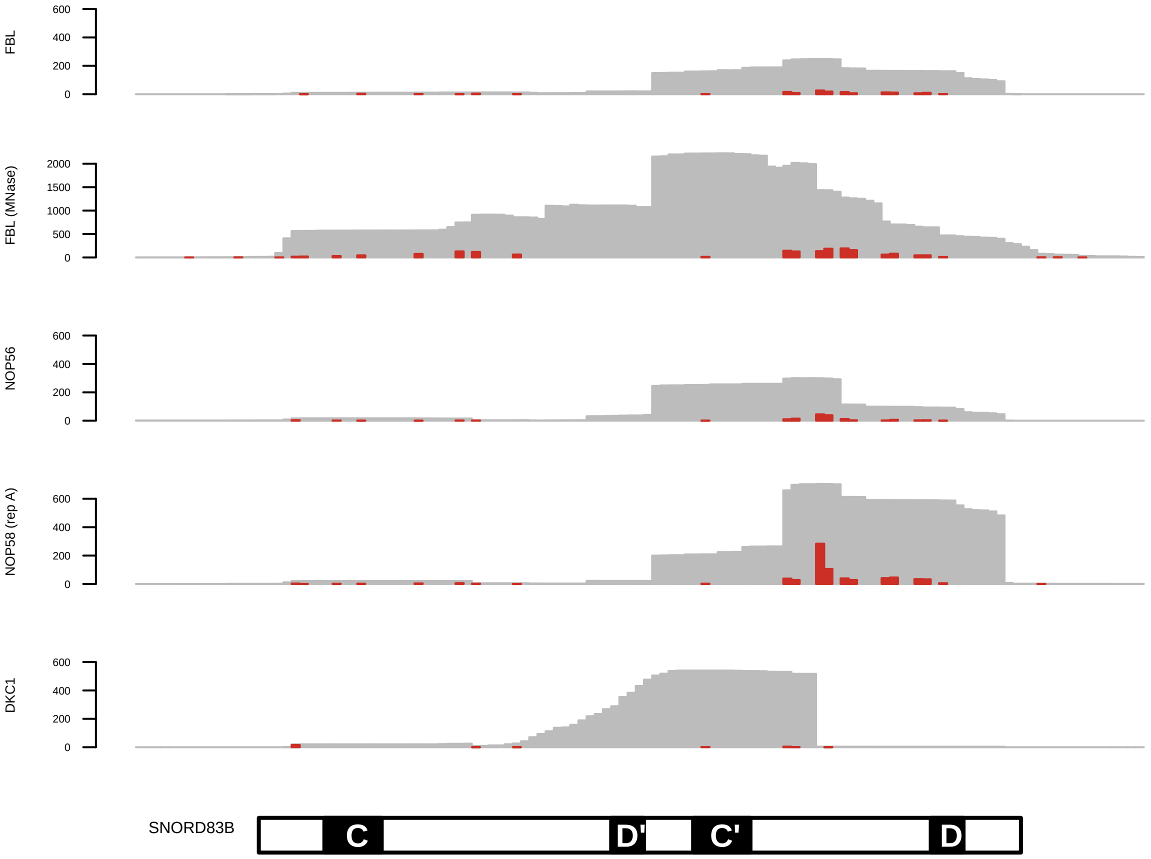
FBL
600
400
200
0
FBL (MNase)
2000
1500
1000
500
0
NOP56
600
400
200
0
NOP58 (rep A)
600
400
200
0
DKC1
600
400
200
0
SNORD83B
C
D'
C'
D

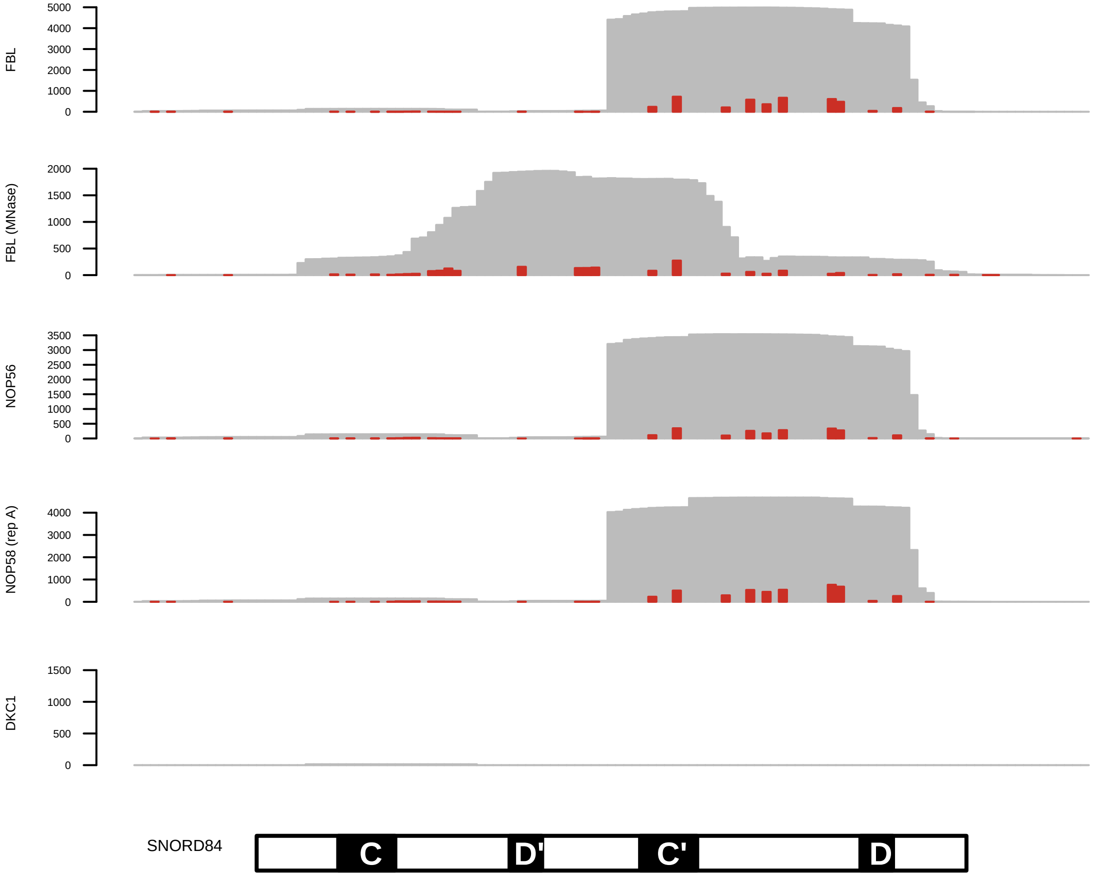
FBL
5000
4000
3000
2000
1000
0
FBL (MNase)
2000
1500
1000
500
0
NOP56
3500
3000
2500
2000
1500
1000
500
0
NOP58 (rep A)
4000
3000
2000
1000
0
DKC1
1500
1000
500
0
SNORD84
C
D'
C'
D

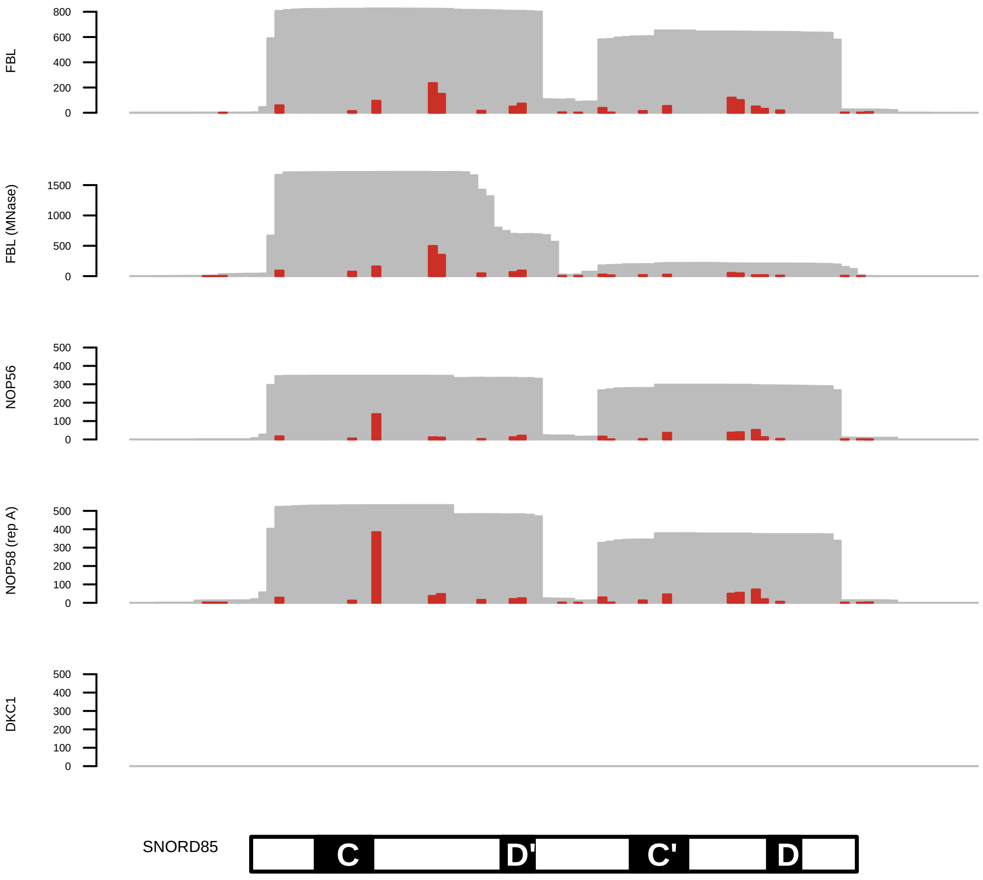

FBL
800
600
400
200
0
FBL (MNase)
1500
1000
500
0
NOP56
500
400
300
200
100
0
NOP58 (rep A)
500
400
300
200
100
0
DKC1
500
400
300
200
100
0
SNORD85
C
D'
C'
D

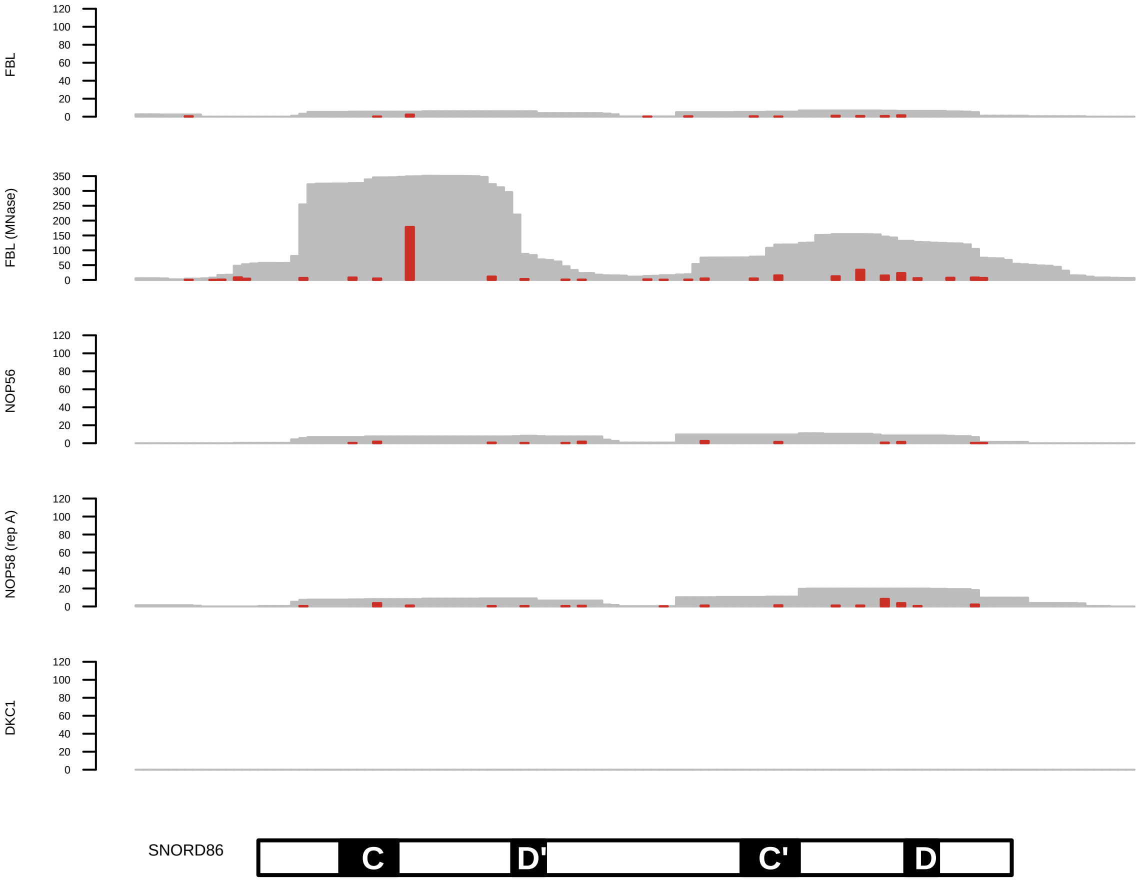
FBL
FBL (MNase)
NOP56
NOP58 (rep A)
DKC1
SNORD86
C
D'
C'
D

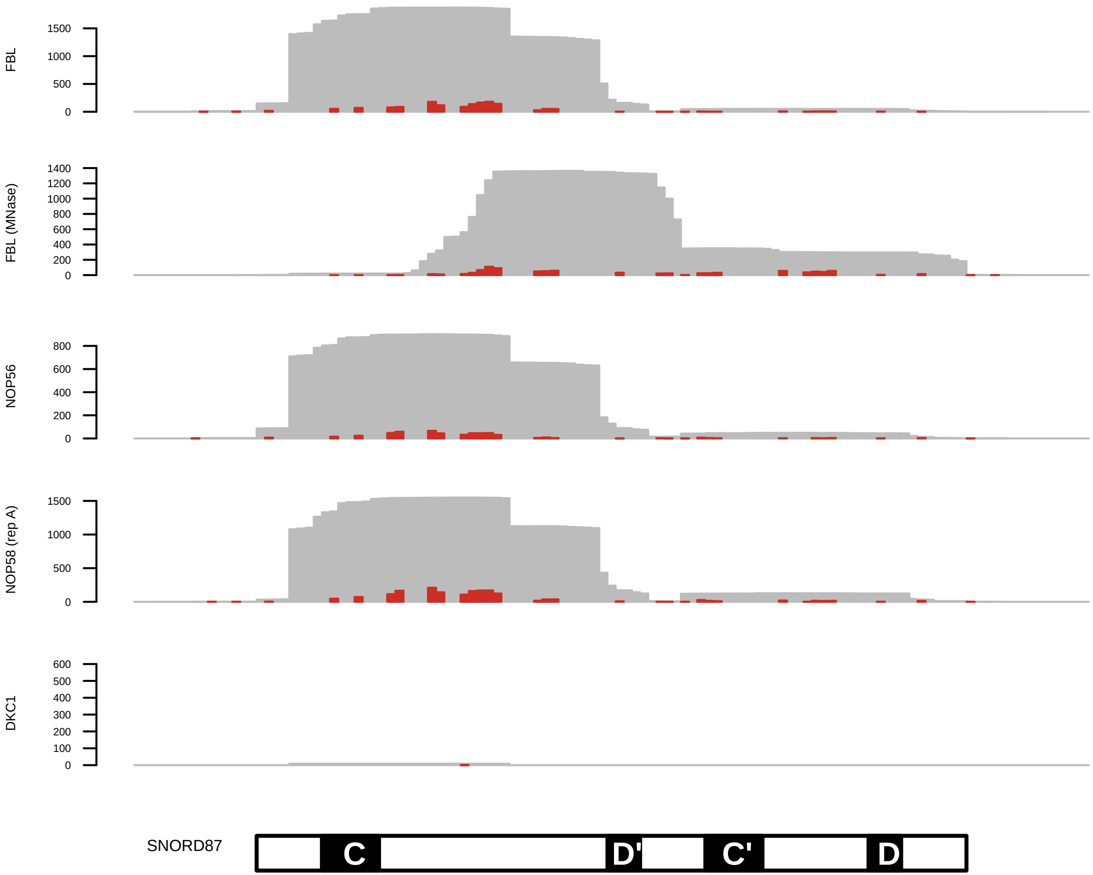
FBL
1500
1000
500
0
FBL (MNase)
1400
1200
1000
800
600
400
200
0
NOP56
800
600
400
200
0
NOP58 (rep A)
1500
1000
500
0
DKC1
600
500
400
300
200
100
0
SNORD87
C
D'
C'
D

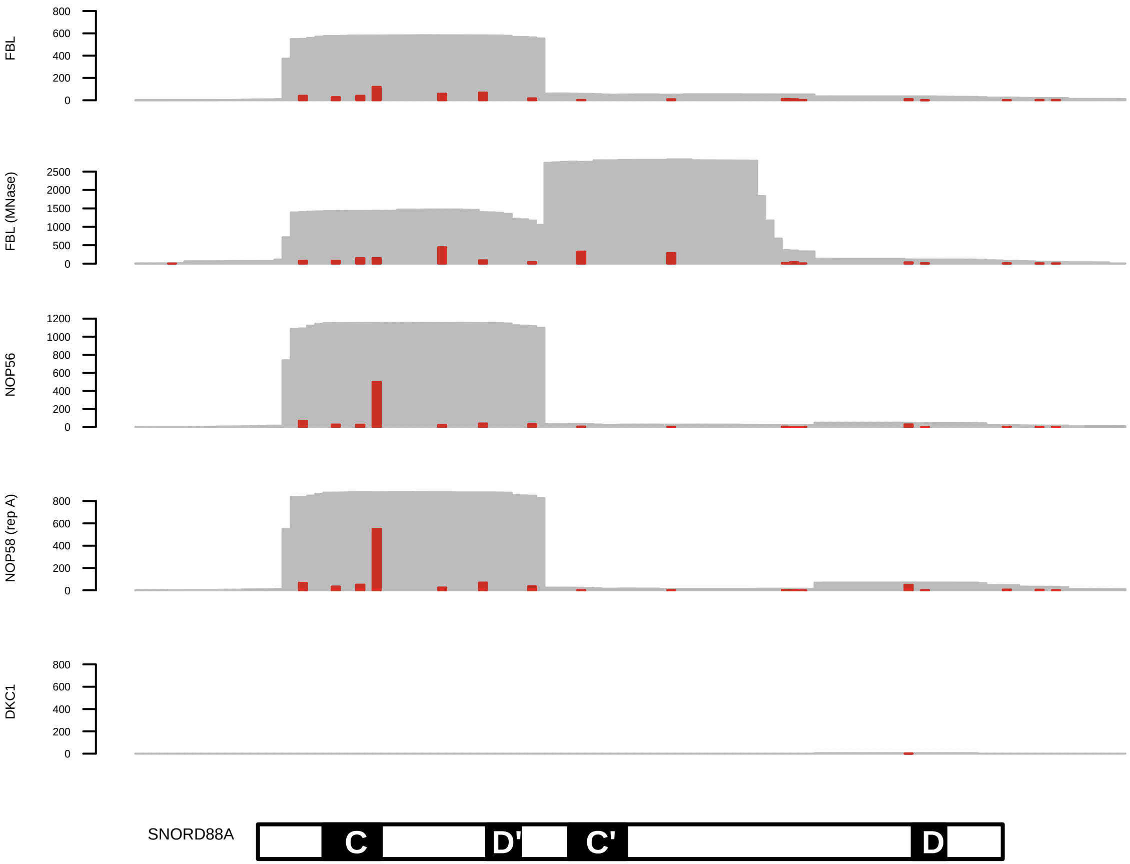
FBL
800
600
400
200
0
FBL (MNase)
2500
2000
1500
1000
500
0
NOP56
1200
1000
800
600
400
200
0
NOP58 (rep A)
800
600
400
200
0
DKC1
800
600
400
200
0
SNORD88A
C
D'
C'
D

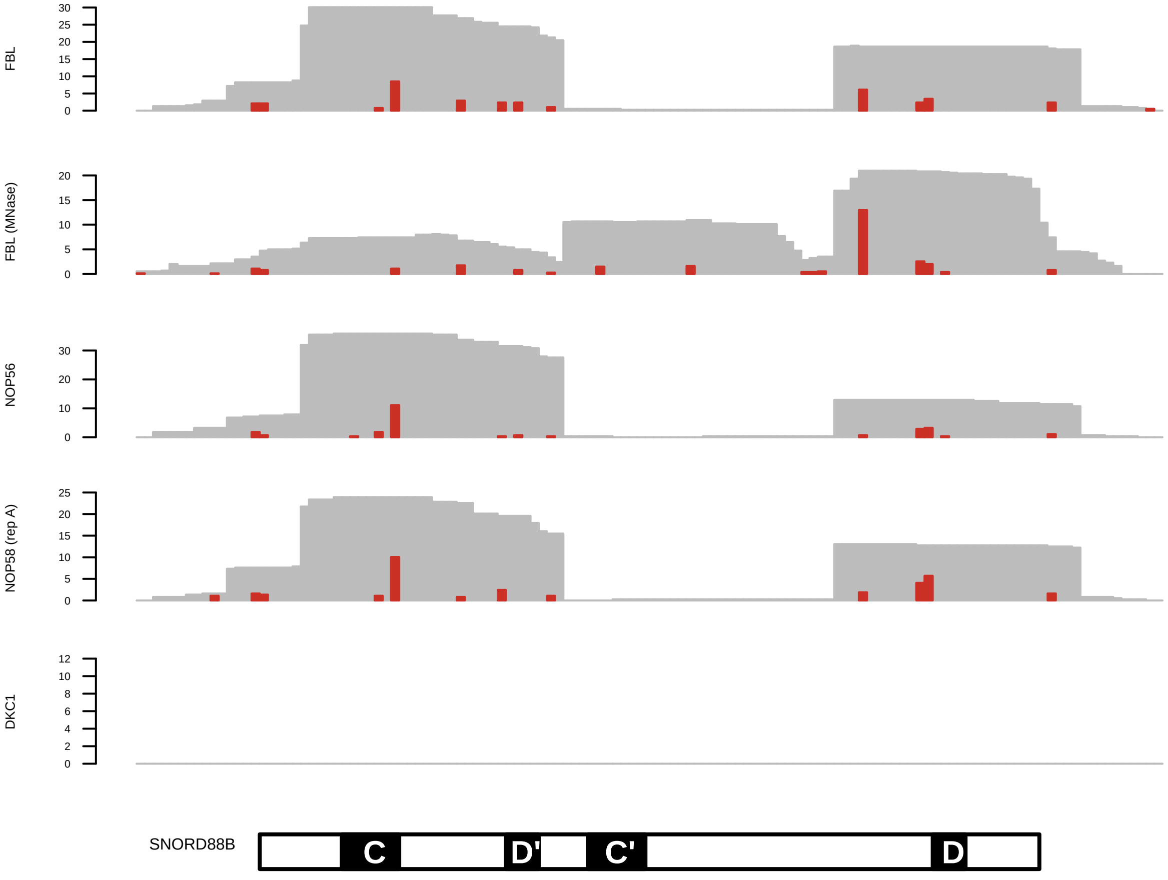
FBL
30
25
20
15
10
5
0
FBL (MNase)
20
15
10
5
0
NOP56
30
20
10
0
NOP58 (rep A)
25
20
15
10
5
0
DKC1
12
10
8
6
4
2
0
SNORD88B
C
D'
C'
D

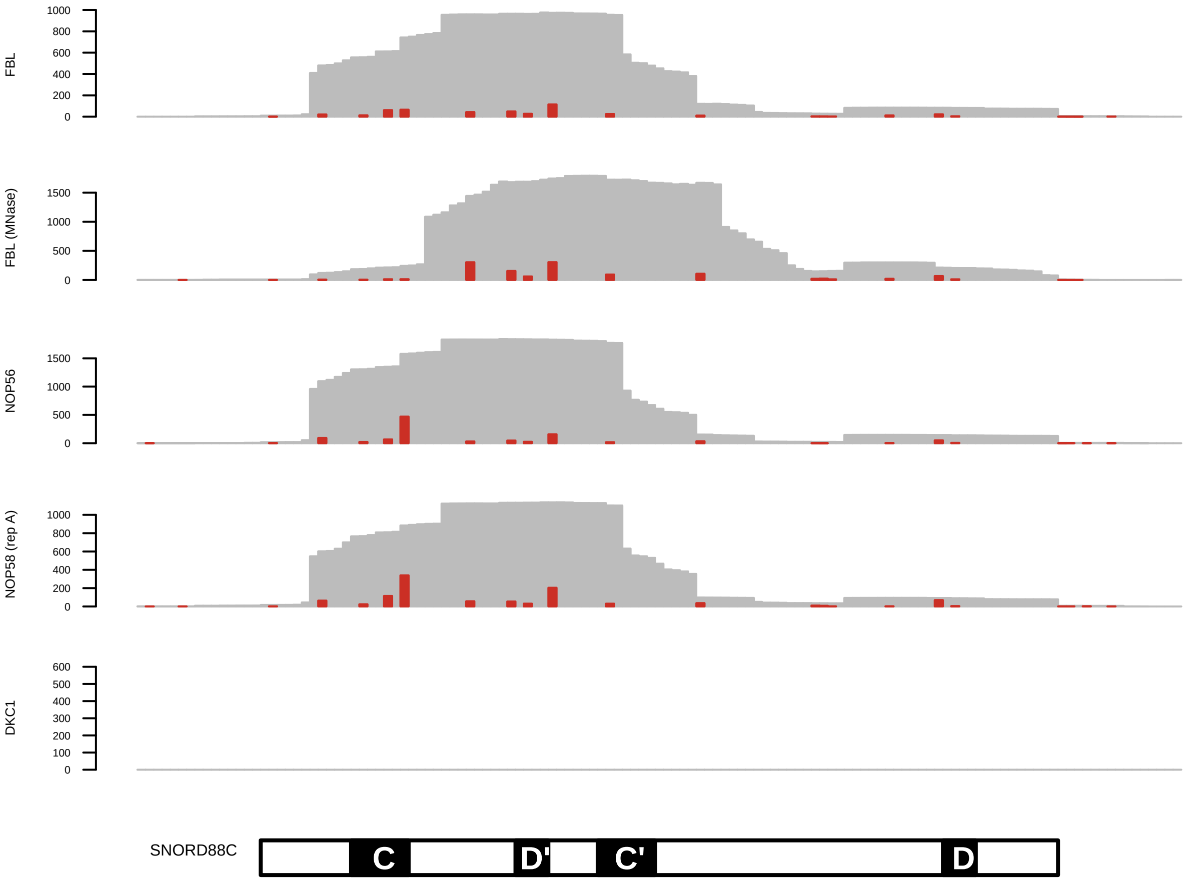

FBL
FBL (MNase)
NOP56
NOP58 (rep A)
DKC1
SNORD88C
C
D'
C'
D

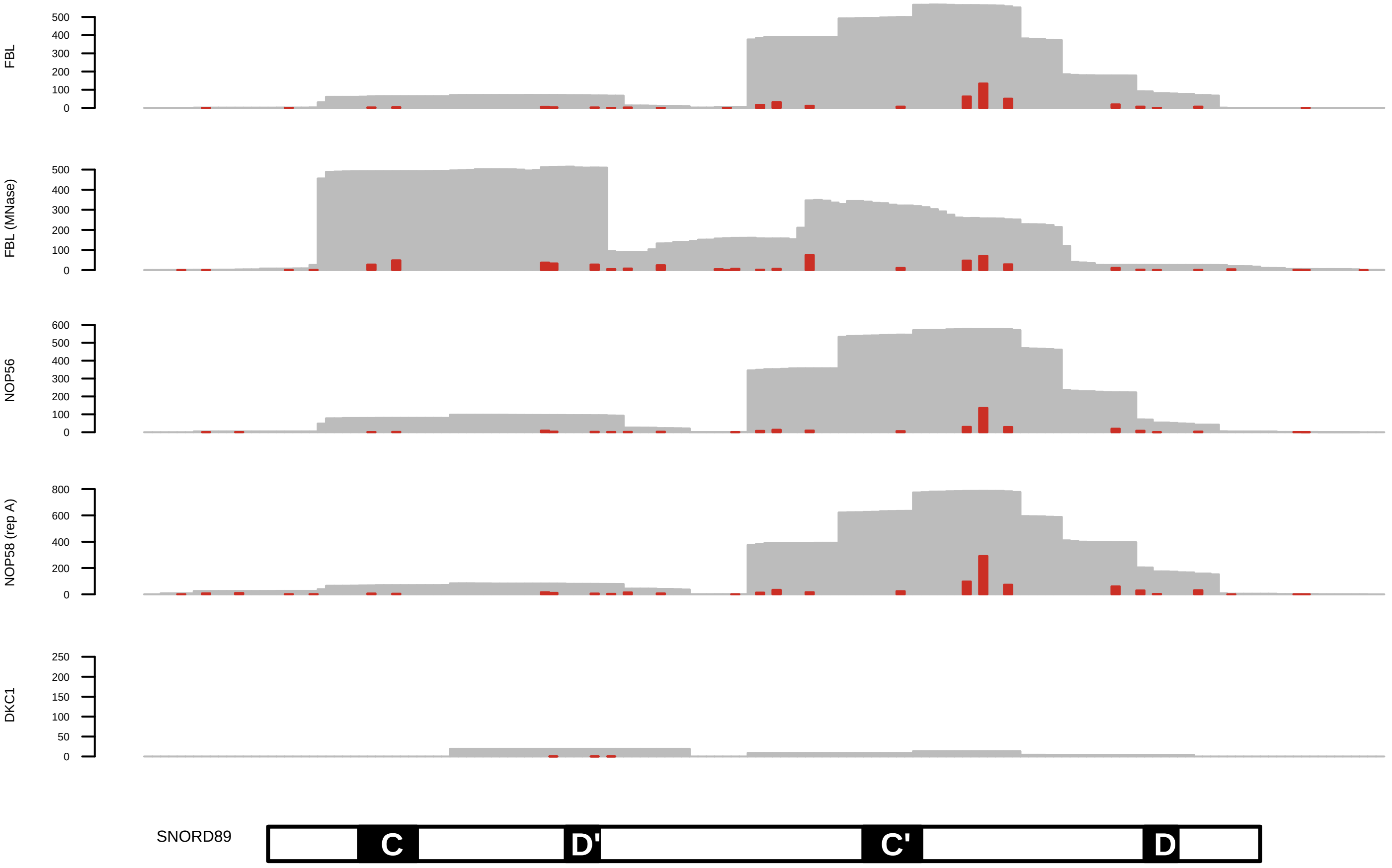

FBL
500
400
300
200
100
0
FBL (MNase)
500
400
300
200
100
0
NOP56
600
500
400
300
200
100
0
NOP58 (rep A)
800
600
400
200
0
DKC1
250
200
150
100
50
0
SNORD89
C
D'
C'
D

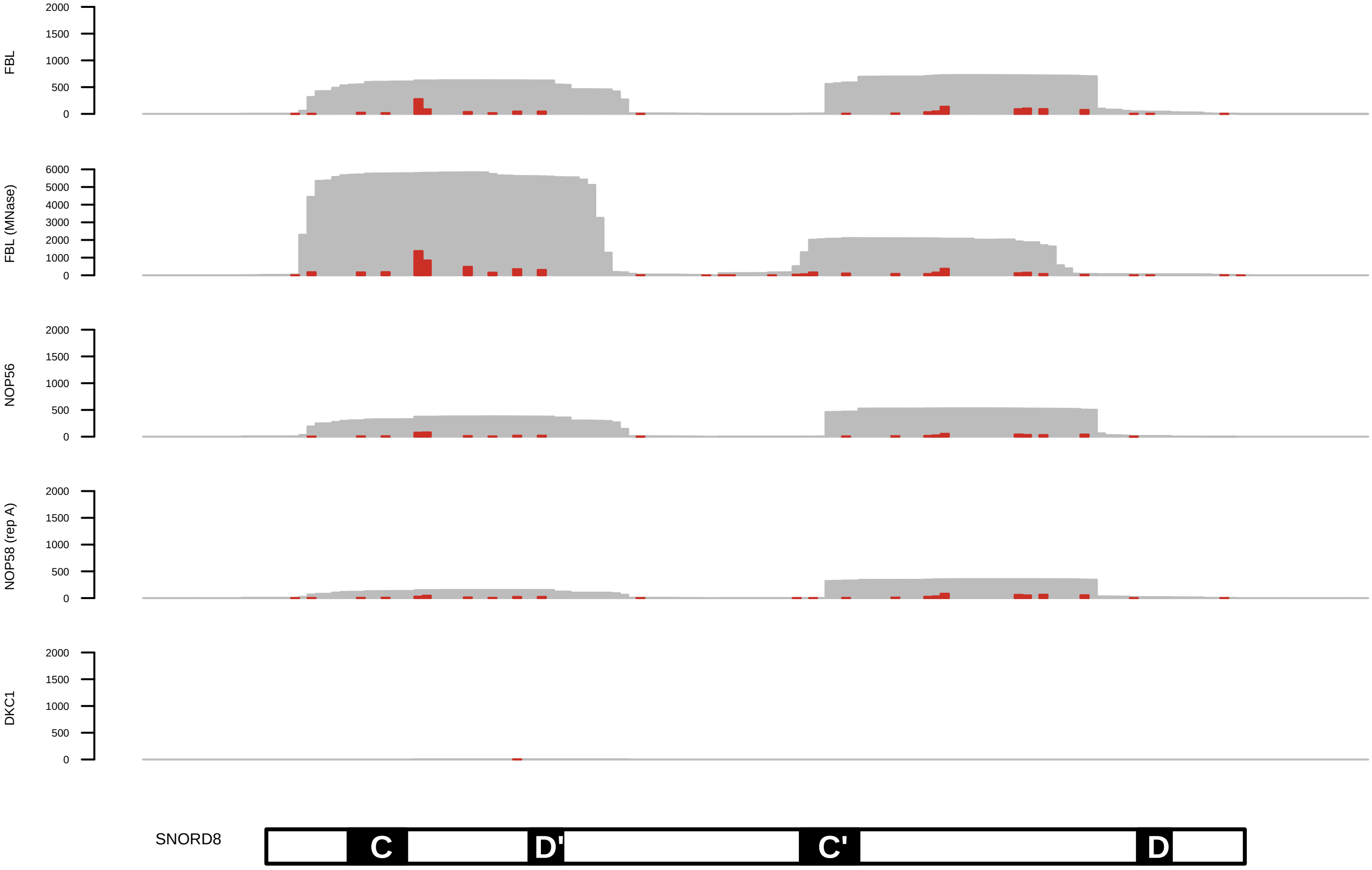
FBL
2000
1500
1000
500
0
FBL (MNase)
6000
5000
4000
3000
2000
1000
0
NOP56
2000
1500
1000
500
0
NOP58 (rep A)
2000
1500
1000
500
0
DKC1
2000
1500
1000
500
0
SNORD8
C
D'
C'
D

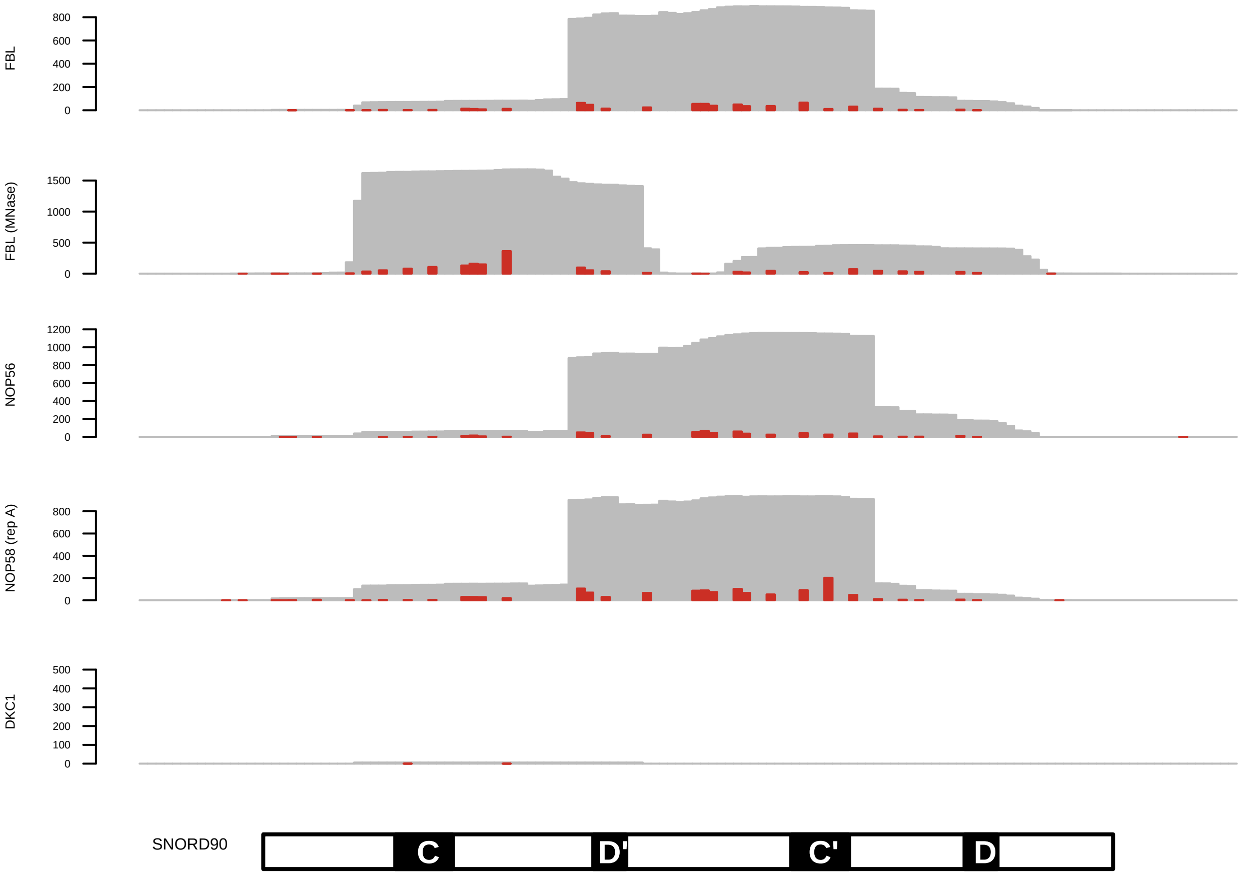
FBL
800
600
400
200
0
FBL (MNase)
1500
1000
500
0
NOP56
1200
1000
800
600
400
200
0
NOP58 (rep A)
800
600
400
200
0
DKC1
500
400
300
200
100
0
SNORD90
C
D'
C'
D

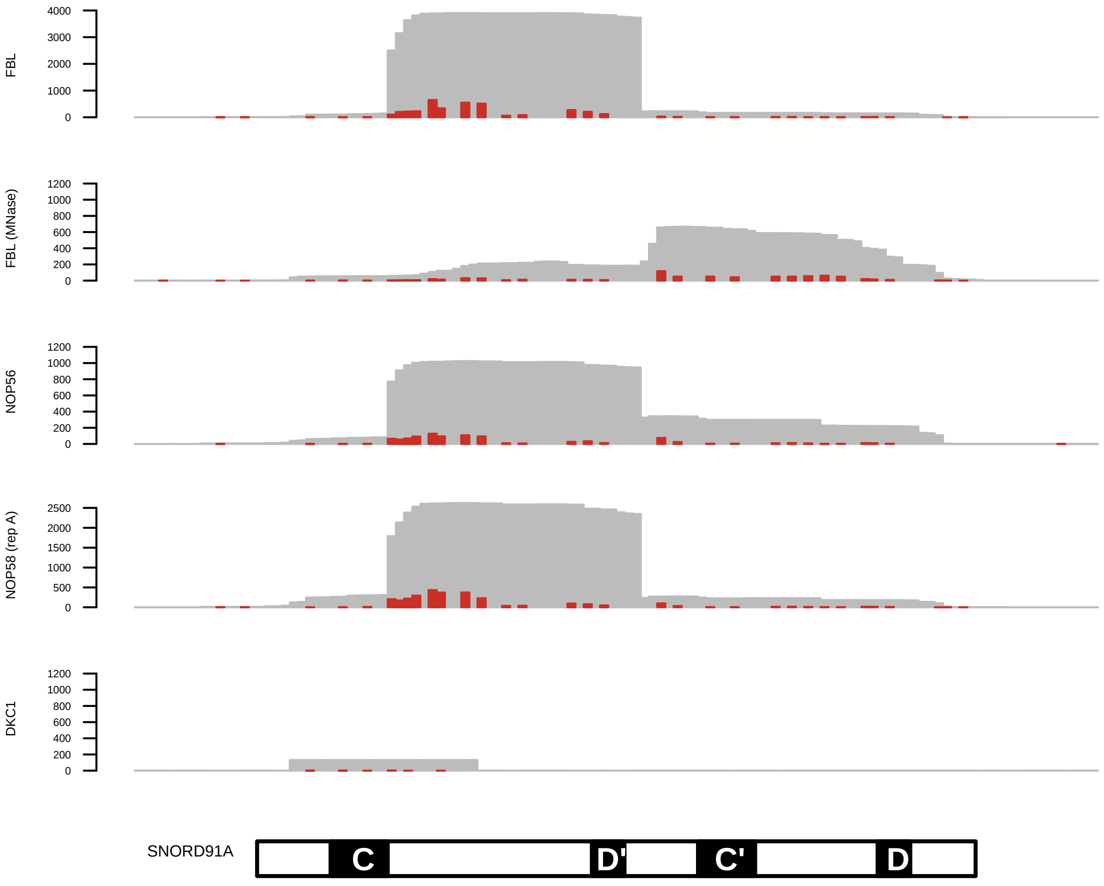
FBL
4000
3000
2000
1000
0
FBL (MNase)
1200
1000
800
600
400
200
0
NOP56
1200
1000
800
600
400
200
0
NOP58 (rep A)
2500
2000
1500
1000
500
0
DKC1
1200
1000
800
600
400
200
0
SNORD91A
C
D'
C'
D

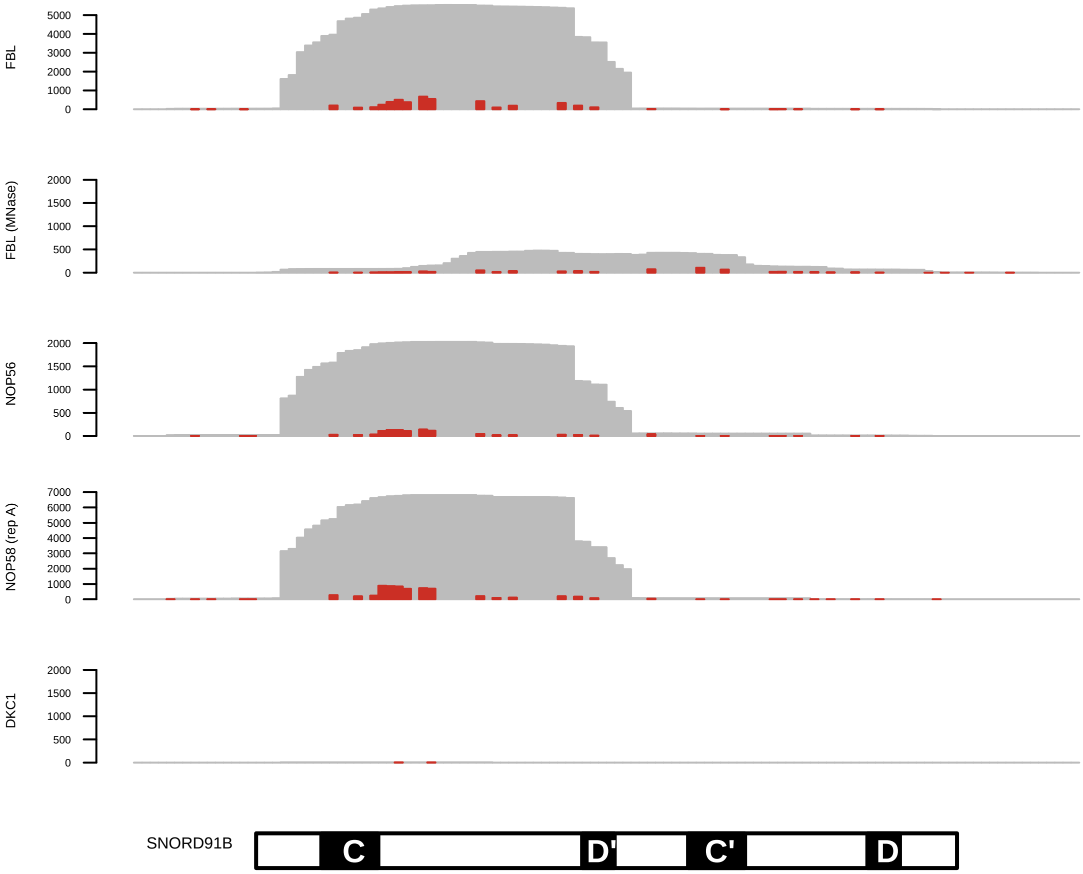
FBL
5000
4000
3000
2000
1000
0
FBL (MNase)
2000
1500
1000
500
0
NOP56
2000
1500
1000
500
0
NOP58 (rep A)
7000
6000
5000
4000
3000
2000
1000
0
DKC1
2000
1500
1000
500
0
SNORD91B
C
D'
C'
D

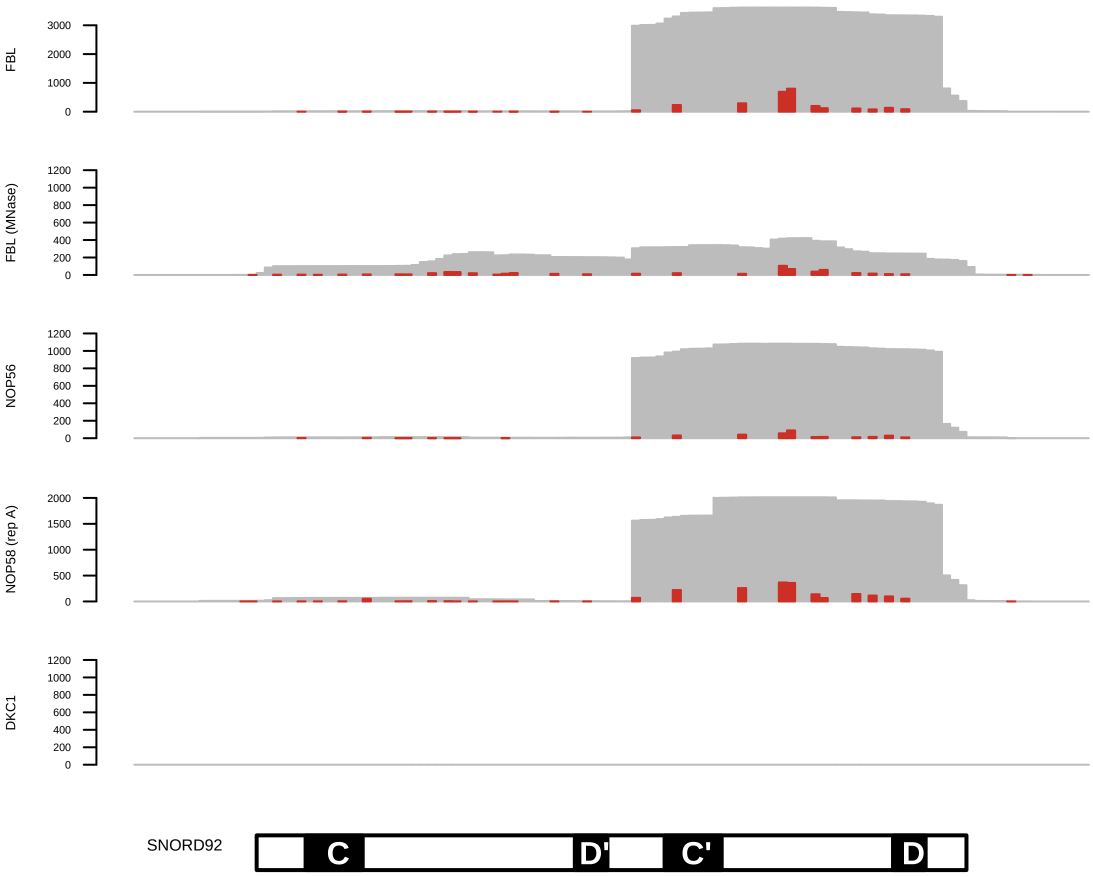

FBL
3000
2000
1000
0
FBL (MNase)
1200
1000
800
600
400
200
0
NOP56
1200
1000
800
600
400
200
0
NOP58 (rep A)
2000
1500
1000
500
0
DKC1
1200
1000
800
600
400
200
0
SNORD92
C
D'
C'
D

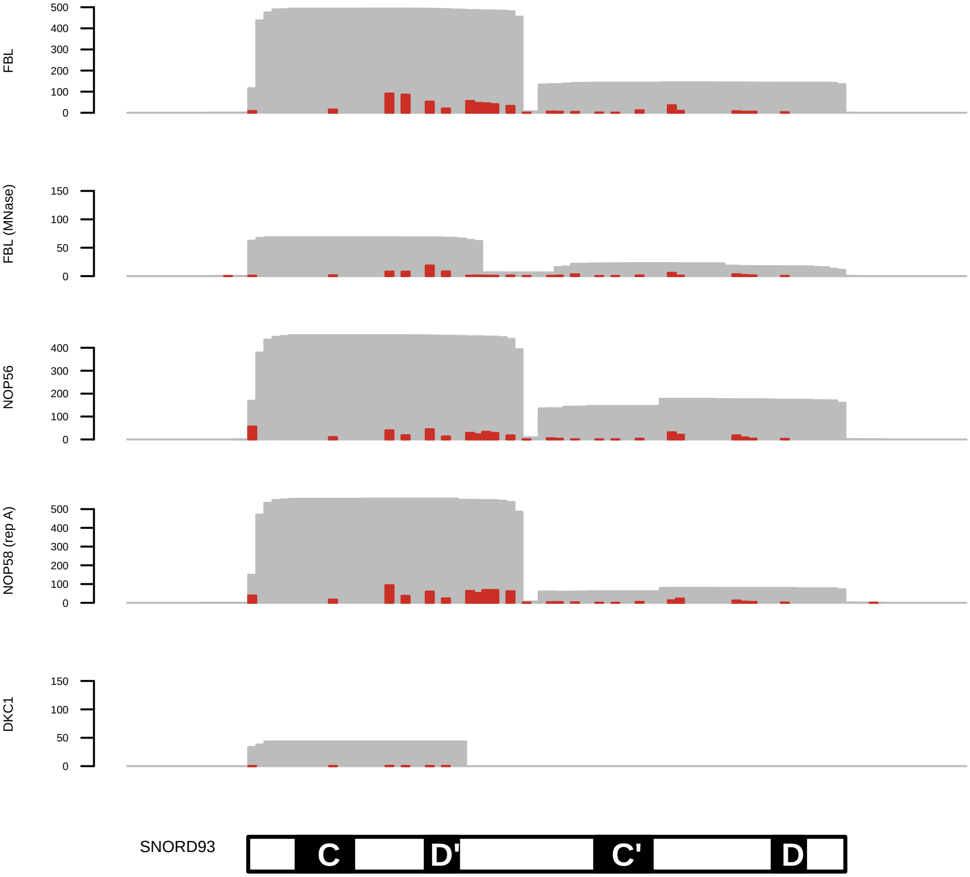
FBL
FBL (MNase)
NOP56
NOP58 (rep A)
DKC1
SNORD93
C
D'
C'
D

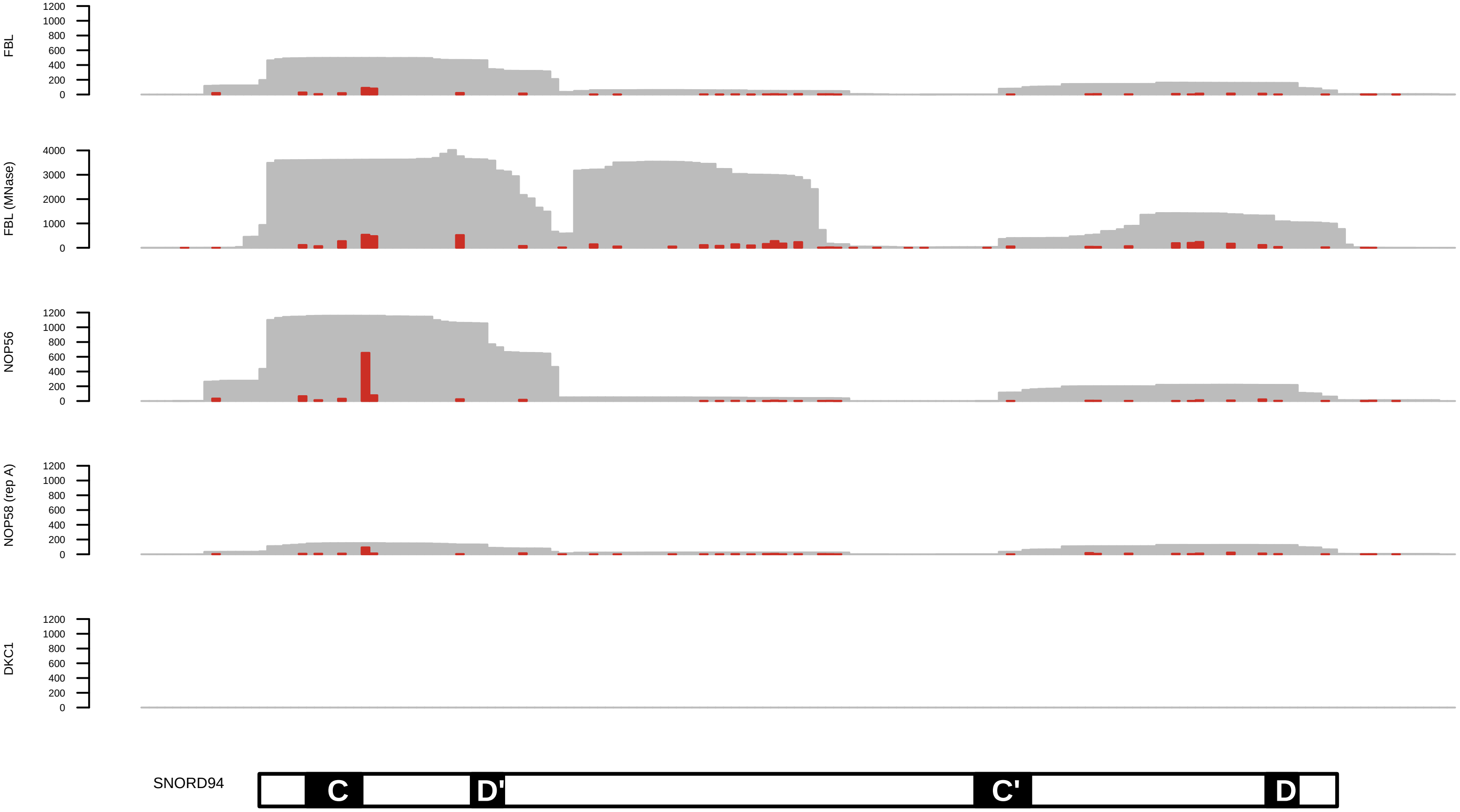
FBL
1200
1000
800
600
400
200
0
FBL (MNase)
4000
3000
2000
1000
0
NOP56
1200
1000
800
600
400
200
0
NOP58 (rep A)
1200
1000
800
600
400
200
0
DKC1
1200
1000
800
600
400
200
0
SNORD94
C
D'
C'
D

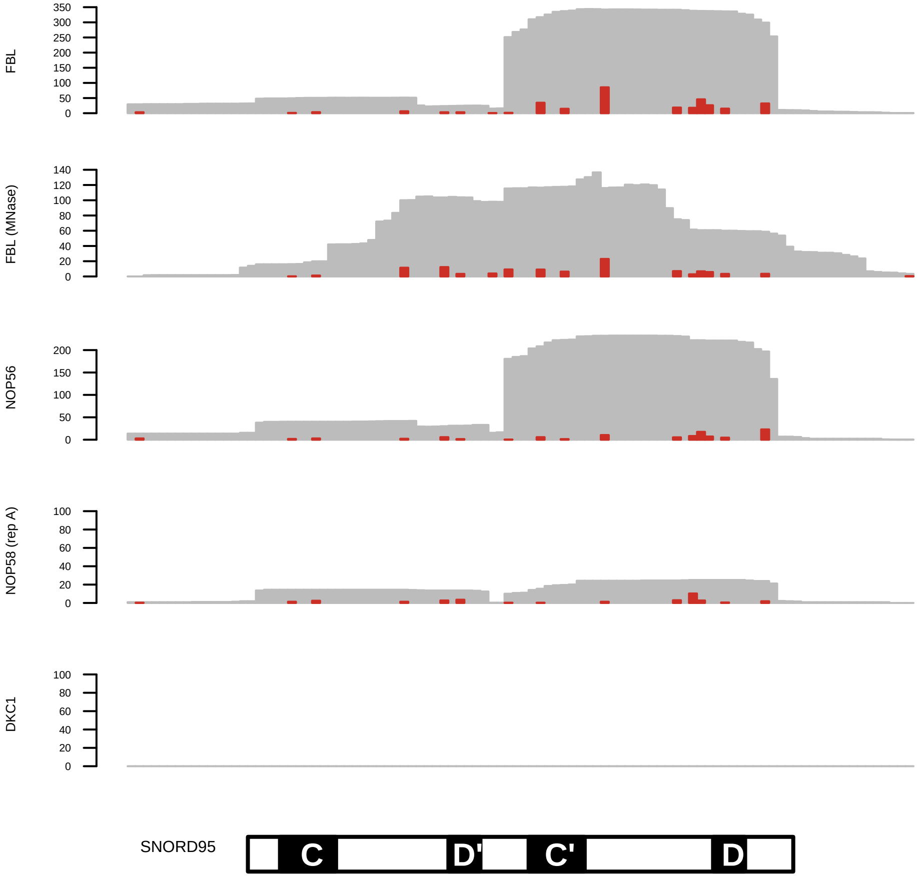
FBL
350
300
250
200
150
100
50
0
FBL (MNase)
140
120
100
80
60
40
20
0
NOP56
200
150
100
50
0
NOP58 (rep A)
100
80
60
40
20
0
DKC1
100
80
60
40
20
0
SNORD95
C
D'
C'
D

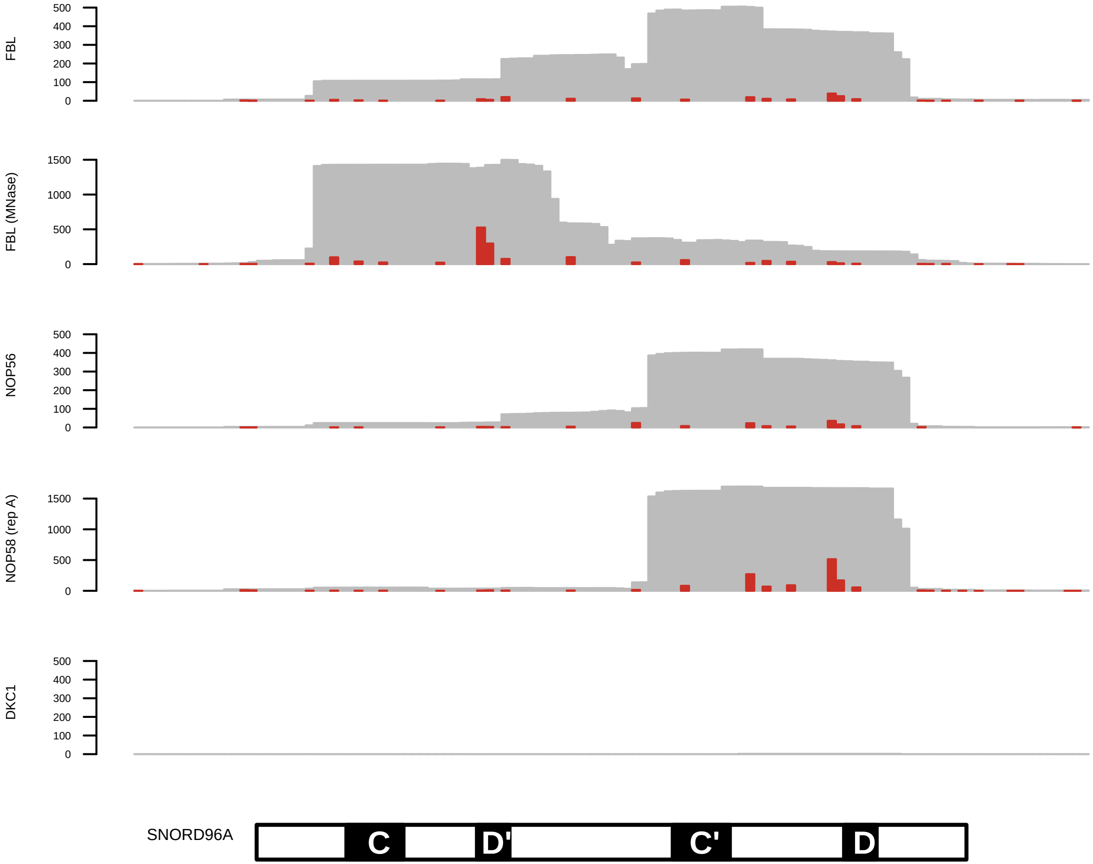
FBL
500
400
300
200
100
0
FBL (MNase)
1500
1000
500
0
NOP56
500
400
300
200
100
0
NOP58 (rep A)
1500
1000
500
0
DKC1
500
400
300
200
100
0
SNORD96A
C
D'
C'
D

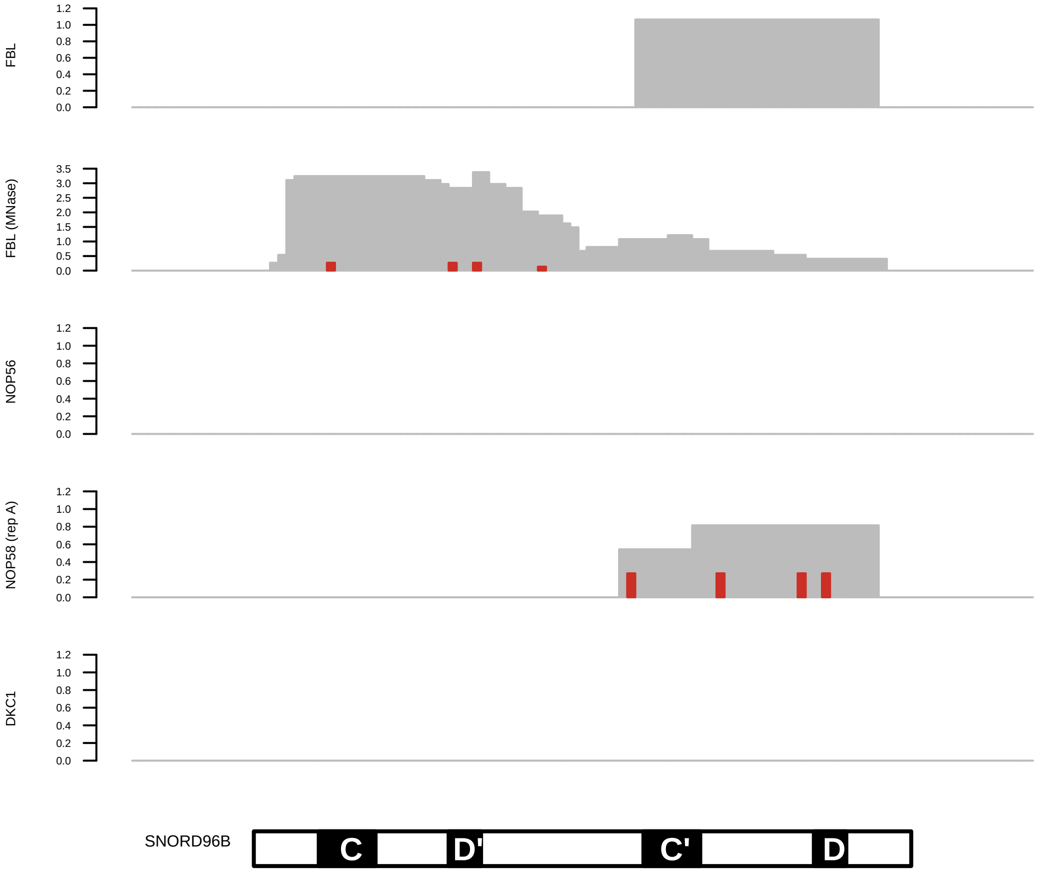
FBL
1.2
1.0
0.8
0.6
0.4
0.2
0.0
FBL (MNase)
3.5
3.0
2.5
2.0
1.5
1.0
0.5
0.0
NOP56
1.2
1.0
0.8
0.6
0.4
0.2
0.0
NOP58 (rep A)
1.2
1.0
0.8
0.6
0.4
0.2
0.0
DKC1
1.2
1.0
0.8
0.6
0.4
0.2
0.0
SNORD96B
C
D'
C'
D

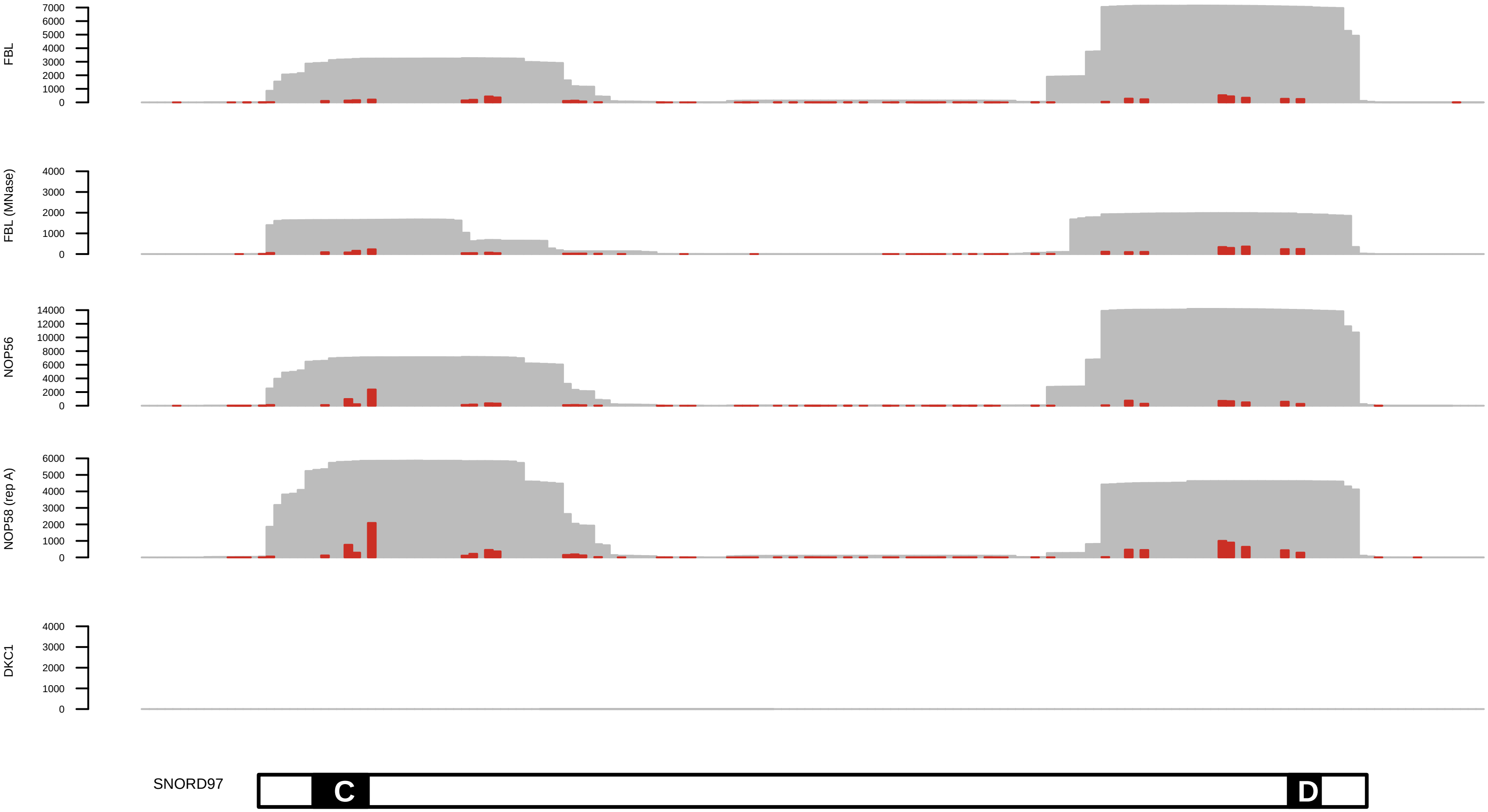

FBL
7000
6000
5000
4000
3000
2000
1000
0
FBL (MNase)
4000
3000
2000
1000
0
NOP56
14000
12000
10000
8000
6000
4000
2000
0
NOP58 (rep A)
6000
5000
4000
3000
2000
1000
0
DKC1
4000
3000
2000
1000
0
SNORD97
C
D

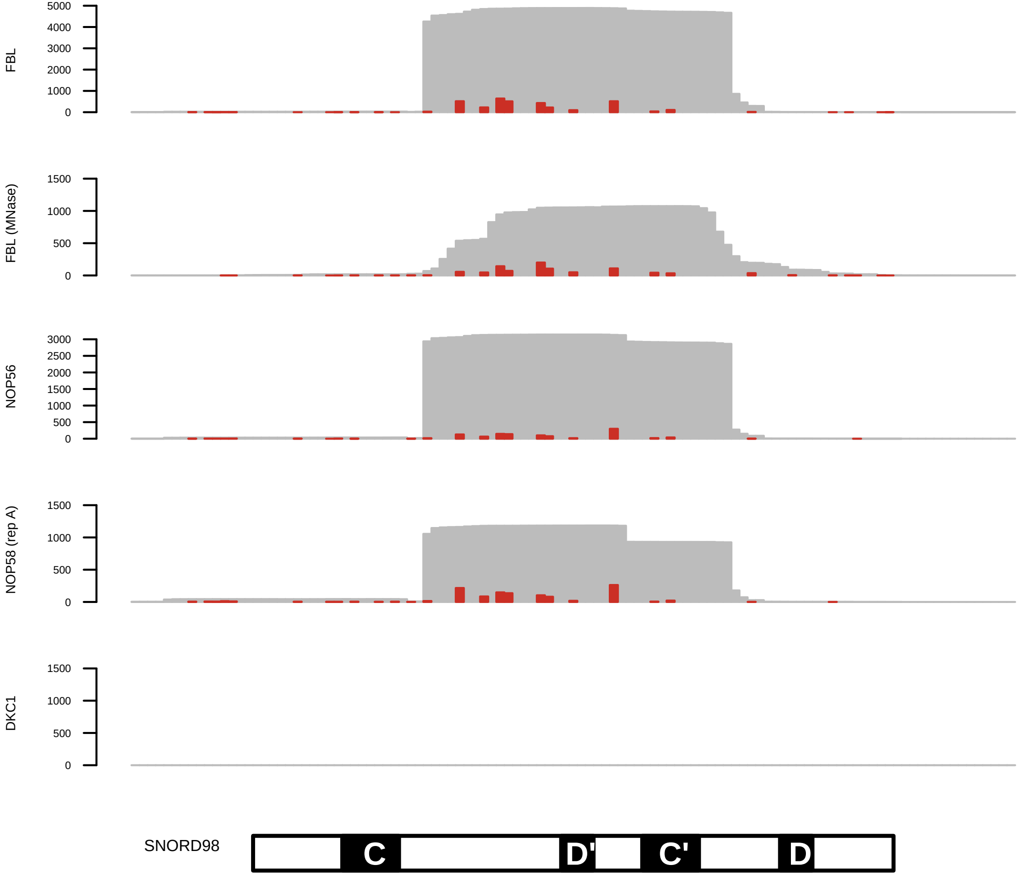

FBL
5000
4000
3000
2000
1000
0
FBL (MNase)
1500
1000
500
0
NOP56
3000
2500
2000
1500
1000
500
0
NOP58 (rep A)
1500
1000
500
0
DKC1
1500
1000
500
0
SNORD98
C
D'
C'
D

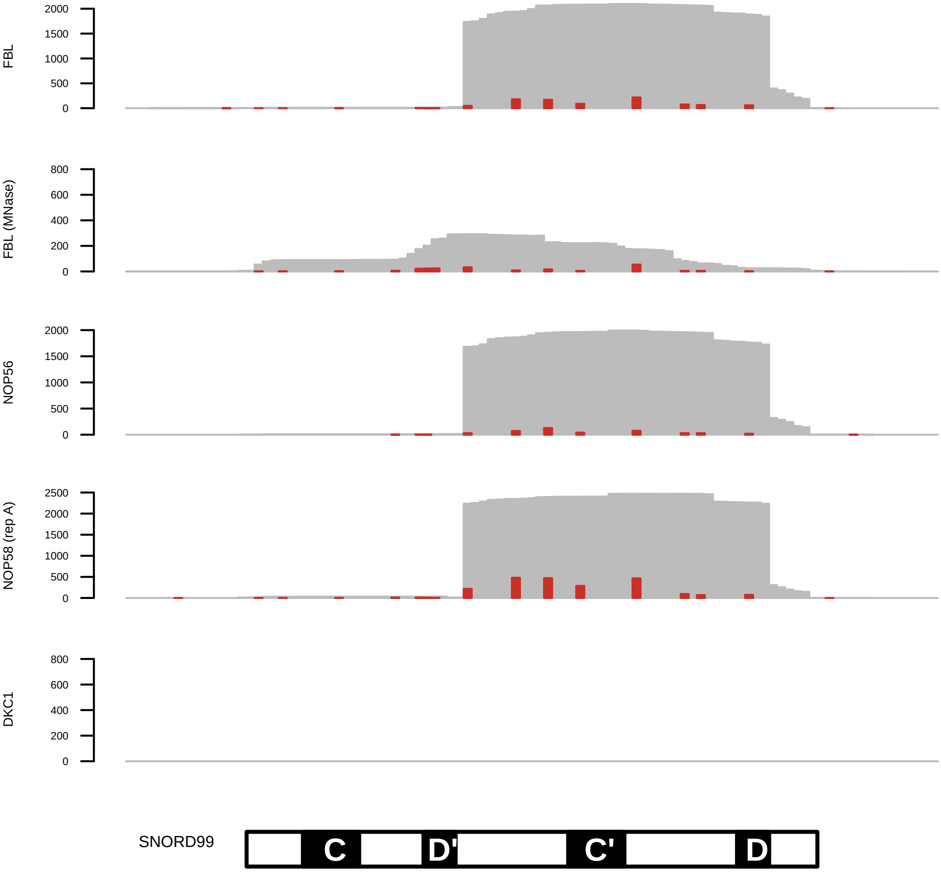
FBL
2000
1500
1000
500
0
FBL (MNase)
800
600
400
200
0
NOP56
2000
1500
1000
500
0
NOP58 (rep A)
2500
2000
1500
1000
500
0
DKC1
800
600
400
200
0
SNORD99
C
D'
C'
D

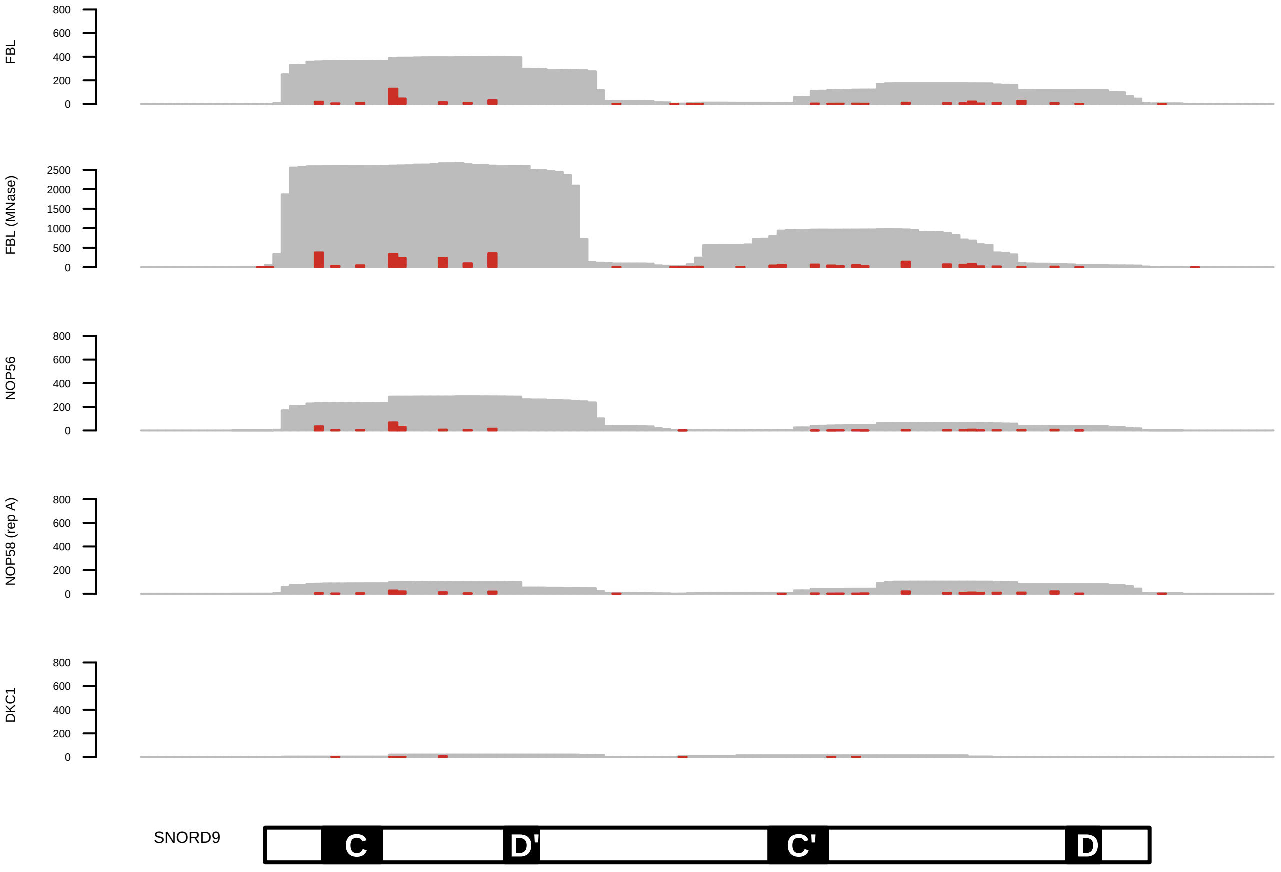
FBL
FBL (MNase)
NOP56
NOP58 (rep A)
DKC1
0
200
400
600
800
500
1000
1500
2000
2500
SNORD9
C
D'
C'
D
