# Supplementary material for: Insights into snoRNA biogenesis and processing from PAR-CLIP of snoRNA core proteins and small RNA sequencing
Source: Genome Biol. 2013 May 26;14(5):R45. doi: 10.1186/gb-2013-14-5-r45 (PMC4053766; doi:10.1186/gb-2013-14-5-r45)
Supplement: Additional file 2 — List of novel C/D box, C/D box-like snoRNAs and mini-snoRNAs obtained in this study. [file gb-2013-14-5-r45-S2.PDF]

Supplementary Table. Novel C/D box, C/D box-like snoRNAs, and mini-snoRNAs.
* in the "target prediction" field marks known modifications sites, or modification sites that have been determined by primer extension assays in this work.

	ID	Location	Host gene	BLAST hits	Length	C box phastcons score (Mammals)	D box phastcons score (Mammals)	C box phastcons score (Primates)	D box phastcons score (Primates)	Target prediction
1	ZL1	chr7:98479316-98479514 (+)	TRRAP	SCARNA9	199	0.993	1.000	0.987	0.993	U2@U47*
2	ZL2	chr19:12814410-12814486 (-)	TNPO2		77	1.000	0.718	0.986	0.338	28S@U4276* , U4571
3	ZL5	chr4:83817006-83817081 (-)	LOC100499177		76	1.000	1.000	0.986	0.994	28S@U2402*
4	ZL6	chr4:83819293-83819378 (-)	LOC100499177		86	0.557	0.920	0.977	0.991	28S@U2402*
5	ZL7	chr2:25467962-25468024 (-)	DNMT3A		63	0.030	0.000	0.105	0.002	
6	ZL8	chr6:31805240-31805322 (+)	C6orf48		83	0.938	0.832	0.665	0.337	
7	ZL9	chr14:70444850-70444913 (+)	SMOC1		64	0.002	0.000	0.064	0.015	
8	ZL11	chr2:29150840-29150934 (+)	WDR43	SNORD53	95	1.000	1.000	0.986	0.969	28S@C3848*
9	ZL12	chr17:80047836-80048015 (-)	FASN		180	1.000	0.000	0.020	0.001	
10	ZL13	chr4:4666053-4666150 (+)	LOC100507266		98	0.000	0.000	0.012	0.003	
11	ZL17	chr4:6281978-6282046 (+)	WFS1		69	0.000	0.002	0.004	0.012	
12	ZL18	chr11:134021184-134021259 (-)	JAM3 3' UTR		76	0.000	0.000	0.010	0.012	
13	ZL19	chr12:123270512-123270631 (+)	CCDC62		120	0.000	0.001	0.040	0.005	18S@G16
14	ZL22	chr3:47703061-47703178 (-)	SMARCC1		118	0.000	0.001	0.153	0.056	
15	ZL23	chr17:37881867-37881893 (+)	ERBB2		27	0.019	0.015	0.001	0.024	
16	ZL24	chr10:76781491-76781549 (+)	KAT6B		59	1.000	0.929	0.993	0.597	
17	ZL25	chr8:144588356-144588419 (-)	ZC3H3		64	0.001	0.000	0.003	0.001	
18	ZL27	chr17:41105504-41105535 (-)	AARSD1		32	0.044	0.001	0.006	0.004	18S@U169
19	ZL30	chr6:31506788-31506858 (-)	DDX39B		71	0.022	0.000	0.390	0.076	
20	ZL31	chrX:79955292-79955351 (-)	BRWD3		60	0.001	0.000	0.098	0.004	
21	ZL34	chr2:74685527-74685566 (+)	WBP1 5'UTR		40	0.284	0.004	0.043	0.120	
22	ZL35	chr17:26648515-26648560 (+)	TMEM97		46	0.002	0.011	0.134	0.599	
23	ZL37	chr5:14464092-14464176 (+)	TRIO		85	0.000	0.000	0.014	0.362	
24	ZL40	chr19:33692841-33692973 (+)	LRP3		133	0.001	0.006	0.018	0.045	
25	ZL41	chr15:22872576-22872695 (+)	TUBGCP5		120	0.006	0.001	0.530	0.001	
26	ZL43	chr1:8554860-8554973 (-)	RERE		114	0.001	0.000	0.015	0.003	

27	ZL45	chr1:161325012-161325057 (+)	SDHC	46	0.002	0.001	0.002	0.005	
28	ZL46	chr12:124854765-124854805 (-)	NCOR2	41	0.001	0.000	0.002	0.000	
29	ZL47	chrX:68858403-68858442 (+)	EDA	40	0.021	0.000	0.541	0.010	
30	ZL48	chr6:35855531-35855613 (-)	SRPK1	83	0.321	0.001	0.083	0.011	
31	ZL49	chr1:38265102-38265139 (+)	MANEAL	38	0.002	0.000	0.006	0.004	
32	ZL50	chr2:101647748-101647801 (-)	TBC1D8	54	0.000	0.001	0.005	0.000	
33	ZL51	chr2:29147423-29147485 (+)	WDR43	63	0.001	0.000	0.059	0.004	
34	ZL52	chr19:55913957-55914028 (-)	UBE2S	72	0.022	0.000	0.011	0.009	
35	ZL53	chr2:241757267-241757374 (-)	KIF1A	108	0.002	0.001	0.058	0.006	
36	ZL54	chr20:3293135-3293241 (-)	C20orf194	107	0.000	0.003	0.003	0.009	
37	ZL56	chr13:61036311-61036429 (+)	TDRD3	119	0.030	0.811	0.023	0.190	
38	ZL60	chr17:25932218-25932298 (+)	KSR1	81	0.000	0.000	0.000	0.003	
39	ZL62	chr1:222831739-222831801 (-)	MIA3	63	0.000	0.001	0.282	0.008	
40	ZL63	chr8:104321264-104321309 (+)	FZD6	46	0.007	0.000	0.345	0.000	
41	ZL64	chr13:48952644-48952705 (+)	RB1	62	0.001	0.014	0.001	0.013	
42	ZL65	chr19:5587445-5587547 (-)	SAFB2	103	0.000	0.000	0.001	0.010	18S@U1017
43	ZL68	chr10:3176296-3176490 (+)	PFKP	195	0.000	0.000	0.002	0.004	
44	ZL69	chr15:89846546-89846578 (+)	FANCI	33	0.000	0.001	0.079	0.124	
45	ZL71	chr1:45136627-45136723 (-)	TMEM53	97	0.003	0.001	0.050	0.043	
46	ZL72	chr8:118933088-118933160 (-)	EXT1	73	0.001	0.000	0.024	0.003	
47	ZL73	chr6:609183-609252 (-)	EXOC2	70	0.001	0.010	0.006	0.004	
48	ZL75	chr7:42976788-42976827 (+)	MRPL32	40	0.004	0.003	0.717	0.608	
49	ZL76	chr9:131844912-131844939 (+)	DOLPP1	28	0.000	0.001	0.229	0.553	
50	ZL77	chr10:70496931-70496957 (+)	CCAR1	27	0.000	0.001	0.007	0.004	
51	ZL78	chr16:72133249-72133286 (+)	DHX38	38	0.004	0.000	0.855	0.057	
52	ZL79	chr3:14535495-14535531 (-)	GRIP2	37	0.003	0.007	0.030	0.015	
53	ZL81	chr4:2261982-2262014 (-)	MXD4	33	0.002	0.002	0.048	0.023	
54	ZL84	chr6:47503420-47503453 (+)	CD2AP	34	0.000	0.000	0.016	0.019	
55	ZL89	chr13:50858040-50858119 (+)	DLEU1	80	0.003	0.521	0.005	0.022	
56	ZL92	chr19:36106857-36106899 (+)	HAUS5	43	0.000	0.003	0.000	0.004	

57	ZL98	chr1:1240102-1240198 (-)	ACAP3		97	0.010	0.992	0.063	0.344	
58	ZL99	chr17:15561137-15561187 (-)	TRIM16		51	0.003	0.001	0.027	0.015	
59	ZL101	chr16:67837905-67837935 (-)	RANBP10		31	0.000	0.023	0.338	0.532	U6@A45
60	ZL102	chr12:132395704-132395806 (+)	ULK1		103	0.000	0.000	0.002	0.003	
61	ZL103	chr7:28602948-28602985 (+)	CREB5		38	0.000	0.007	0.003	0.009	
62	ZL104	chr1:11080232-11080326 (+)	TARDBP		95	0.004	0.005	0.004	0.008	
63	ZL107	chr5:85916323-85916386 (+)	COX7C		64	0.960	1.000	0.987	0.960	18S@C797*
64	ZL109	chr5:137894658-137894730 (-)	HSPA9	SNORD63	73	0.537	0.71	0.078	0.408	
65	ZL114	chr9:20442184-20442242 (-)	MLLT3		59	0.002	0.002	0.025	0.049	
66	ZL116	chr19:39958862-39958933 (+)	SUPT5H		72	0.000	0.034	0.008	0.463	
67	ZL118	chr15:100588039-100588172 (-)	ADAMTS17		134	0.000	0.001	0.006	0.040	28S@C544
68	ZL119	chr8:59509871-59509905 (-)	NSMAF		35	0.058	0.000	0.159	0.012	
69	ZL120	chr10:74113771-74113928 (-)	DNAJB12		158	0.003	0.000	0.009	0.006	
70	ZL121	chr18:76918328-76918363 (+)	ATP9B		36	0.052	0.024	0.052	0.024	
71	ZL122	chr16:10845220-10845342 (+)	NUBP1		123	0.000	0.002	0.020	0.128	
72	ZL126	chr3:52722904-52722971 (+)	GNL3	SNORD19;SNORD19B	68	0.890	0.282	0.664	0.399	18S@G683*
73	ZL127	chr3:52725392-52725462 (+)	GNL3	SNORD19 (ENSEMBL)	71	0.370	1.000	0.523	0.980	18S@G683*
74	ZL132	chr2:203142832-203142914 (+)	NOP58	SNORD70	83	1.000	1.000	0.981	0.995	18S@A512*
75	ZL142	chr12:50850353-50850570 (+)	LARP4	SNORD97	218	0.997	1.000	0.943	0.988	
76	ZL149	chr22:42004850-42004874 (-)	PPPDE2		25	0.001	0.024	0.035	0.102	
77	ZL150	chr19:38653734-38653791 (+)	SIPA1L3		58	0.000	0.003	0.004	0.234	
