# Supplementary material for: Insights into snoRNA biogenesis and processing from PAR-CLIP of snoRNA core proteins and small RNA sequencing
Source: Genome Biol. 2013 May 26;14(5):R45. doi: 10.1186/gb-2013-14-5-r45 (PMC4053766; doi:10.1186/gb-2013-14-5-r45)
Supplement: Additional file 3 — List of novel H/ACA snoRNAs or homologs of known snoRNAs (indicated in the 'BLAST hits' column) that were obtained in this study. [file gb-2013-14-5-r45-S3.PDF]

**Supplementary Table.** Novel H/ACA snoRNAs.

| | ID | Location | Host gene | BLAST hits | Length |
|---|---|---|---|---|---|
| 1 | ZL4 | chr14:36232006-36232136 (-) | RALGAPA1 | | 131 |
| 2 | ZL36 | chr4:185561291-185561394 (-) | CASP3 | | 104 |
| 3 | ZL105 | chrX:54953738-54953866 (+) | TRO | SNORA11C;SNORA11D; SNORA11;SNORA11B; SNORA11E | 129 |
| 4 | ZL106 | chrX:114360879-114361007 (-) | LRCH2 | SNORA35(ENSEMBL) | 129 |
| 5 | ZL108 | chr17:37007762-37007911 (-) | RPL23 | SNORA21(ENSEMBL) | 150 |
| 6 | ZL110 | chr5:138611868-138612011 (+) | SNHG4 | SNORA74B;SNORA74A | 144 |
| 7 | ZL111 | chr10:74885838-74885965 (-) | NUDT13 | SNORA11C;SNORA11D; SNORA11;SNORA11B; SNORA11E | 128 |
| 8 | ZL112 | chr1:154232203-154232337 (+) | UBAP2L | SNORA58 | 135 |
| 9 | ZL113 | chr13:45910449-45910582 (-) | INTERGENIC | SNORA31(ENSEMBL) | 134 |
| 10 | ZL129 | chr3:183169639-183169778 (+) | LOC100505687 | SNORA63 | 140 |
| 11 | ZL130 | chr3:183171600-183171735 (+) | LOC100505687 | SNORA63 | 136 |
| 12 | ZL131 | chr7:6056509-6056644 (+) | AIMP2 | SNORA80B;SNORA42; SNORA80 | 136 |
| 13 | ZL133 | chr22:20113923-20114047 (+) | RANBP1 | SNORA77 | 125 |
| 14 | ZL134 | chr14:20791338-20791487 (-) | CCNB1IP1 | SNORA79 | 150 |
| 15 | ZL135 | chr15:65577799-65577930 (-) | PARP16 | SNORA24 | 132 |
| 16 | ZL138 | chr1:193026411-193026546 (-) | UCHL5 | SCARNA18 | 136 |
| 17 | ZL143 | chr7:56123060-56123195 (+) | CCT6A | SNORA22 | 136 |
| 18 | ZL144 | chr12:124101257-124101387 (+) | DDX55 | SNORA9 | 131 |
| 19 | ZL146 | chr9:20786924-20787054 (+) | KIAA1797 | SNORA30;SNORA37 | 131 |
| 20 | ZL147 | chr22:34100772-34100906 (-) | LARGE | SNORA50 | 135 |
