# Supplementary material for: Insights into snoRNA biogenesis and processing from PAR-CLIP of snoRNA core proteins and small RNA sequencing
Source: Genome Biol. 2013 May 26;14(5):R45. doi: 10.1186/gb-2013-14-5-r45 (PMC4053766; doi:10.1186/gb-2013-14-5-r45)

ZL1 chr7:98479316-98479514 (+)

AGAGAAGCAAAGTGATGAGTAATACTGGCTGGAGCCCCAAAGAGGCACGTGTGTGTGTTTGTGTGTGTGTGTATATGCTTGTCAGTGCATGCACGTGTATGTCTGGGAGTACAAATGGGTGCGACTGGTTGTAGGGAACTAGCTATGTGCCTTCTATTAGGCCATGACAGTCAAACTGATAAGATCTGATTGCTTC

((((((((((...)))))))

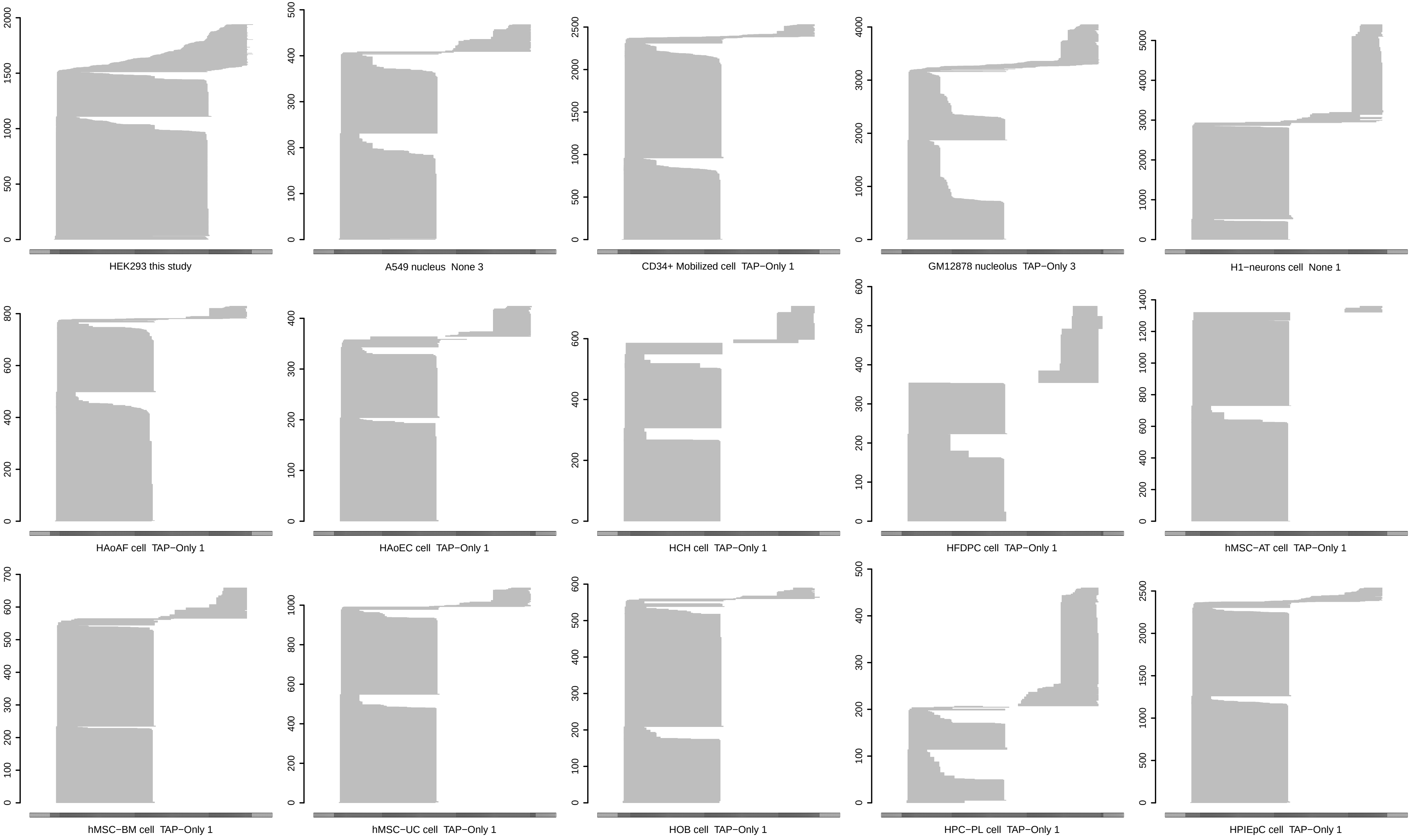

ZL2 chr19:12814410-12814486 (-)

GGCCTCAAGTGATGGAGAGCAATACCCGGGGATGACTGTGACCACATTGGGATGTATTCGTACTGTCTGATGGGGCC

(((((((........................dddd..ccccccc..........................)))))))

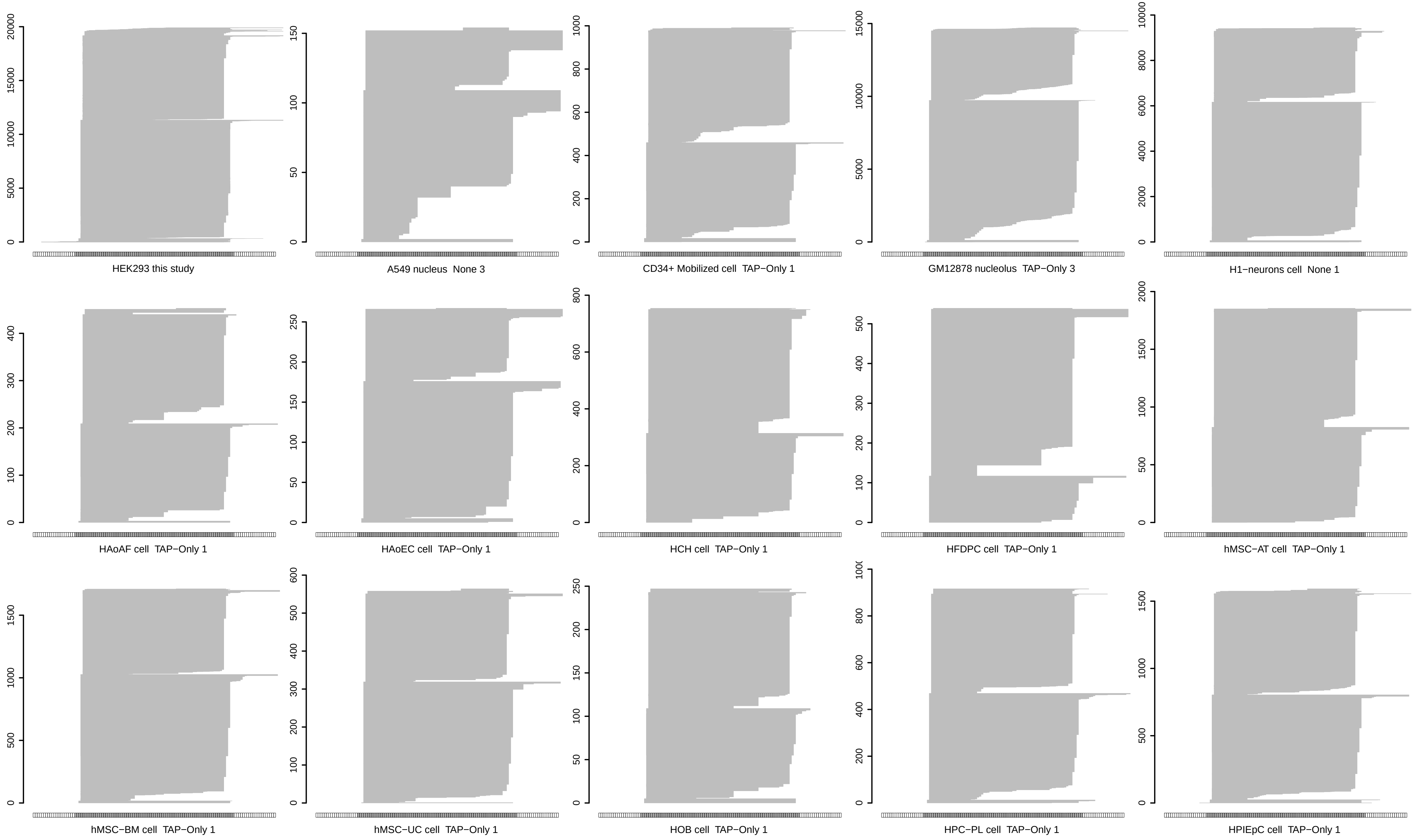

ZL4 chr14:36232006-36232136 (-)

GGTGATTCTTTTTATATGCCCTTCTGATCCCAAGTATGGCAGAAGGATATAAAGGTCACCAAGAATAATTTGGAGGTTGCCAAGGACTTATTTAAAAGTGTTTTCAAGTCCGACCGAGAACTCTGACATTT

((((((...((((((((..(((((((...(((....))))))))))))))))))))))))............(((.(((....((((((................))))))...)))...)))........

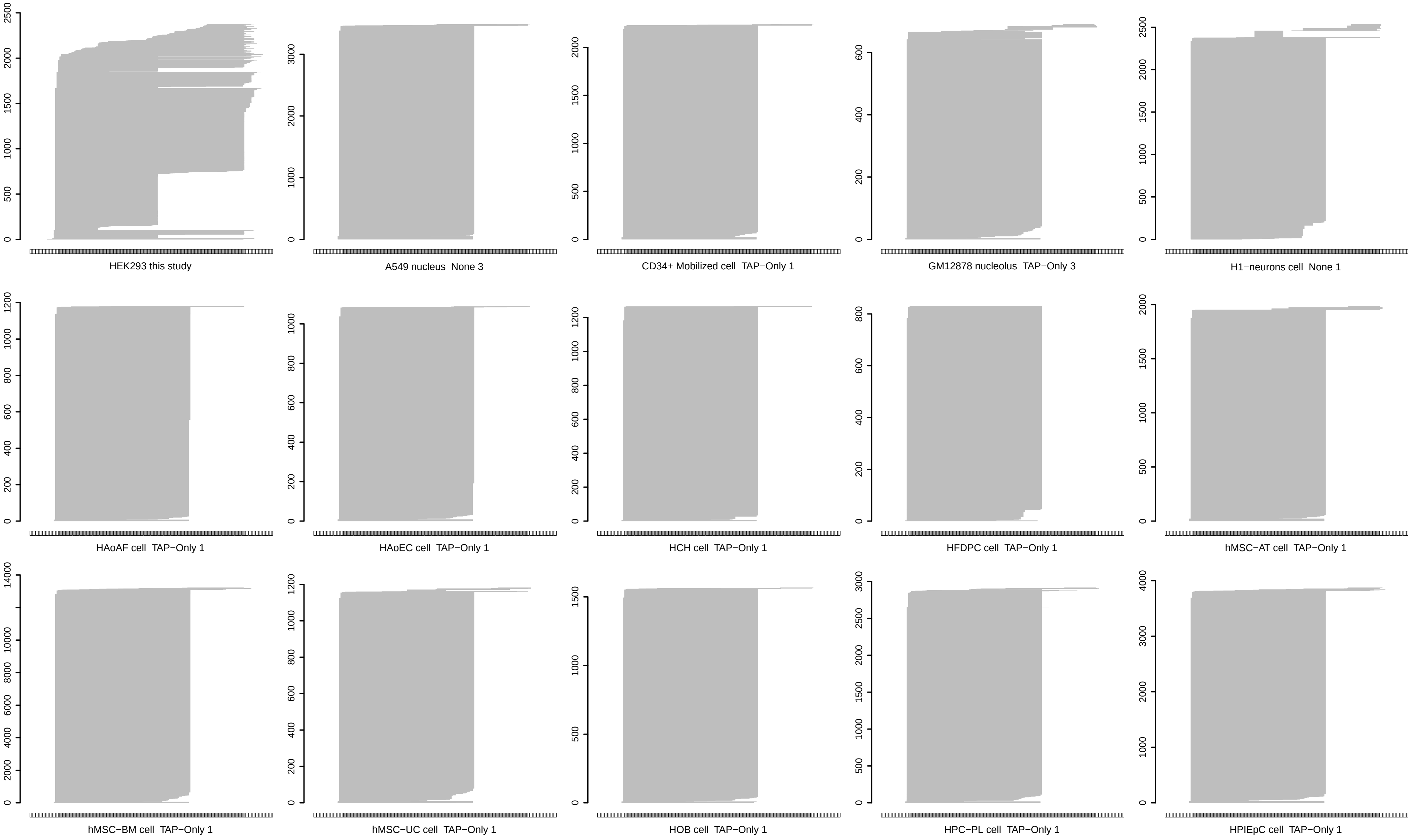

ZL5 chr4:83817006-83817081 (-)

ATGTCCAATGATTAAATTTTTTCCACTGCTGTTCACATCATGATTTGTTGTGATTAATGTATGTGGATGAGGACAT

((((((..))))))

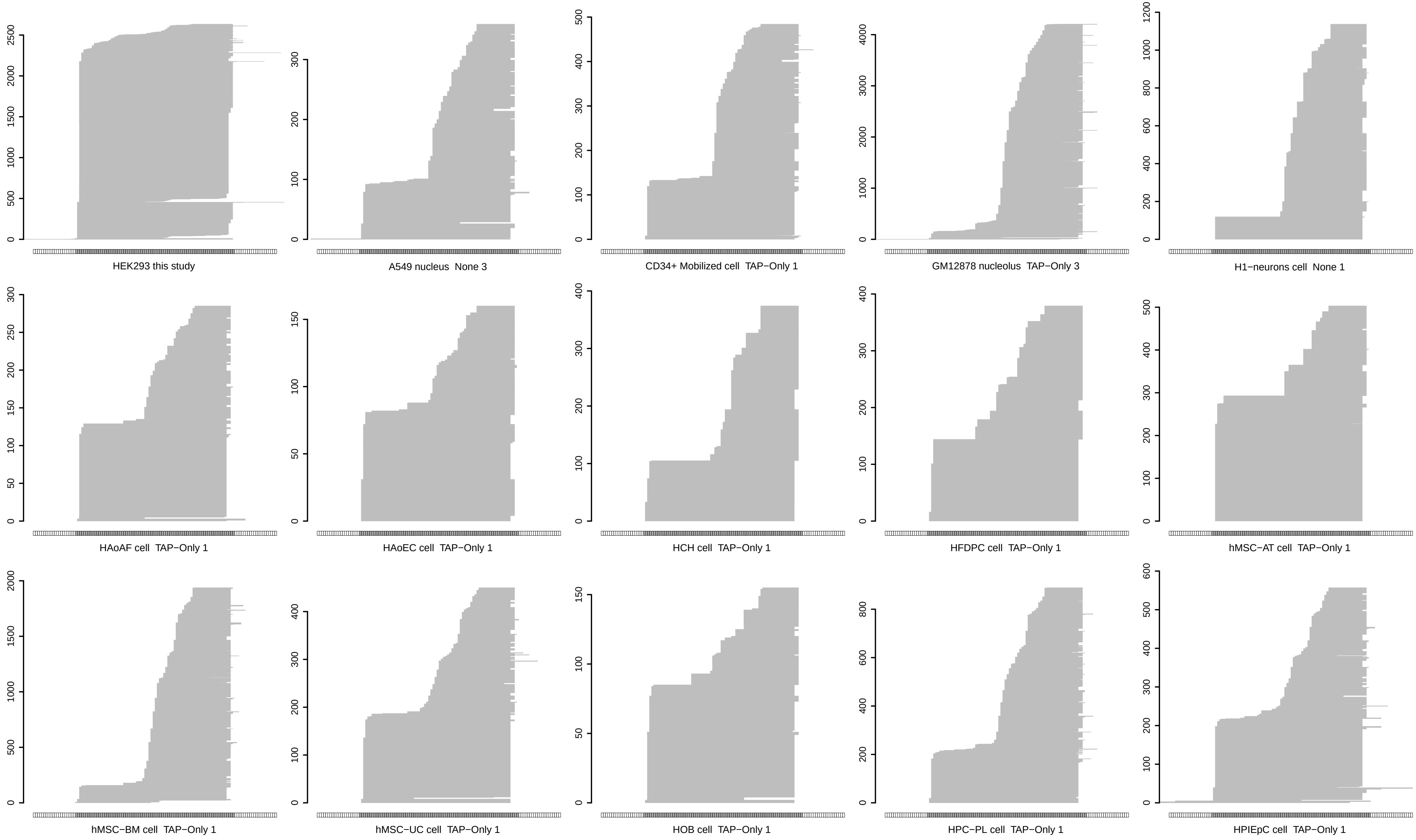

ZL6 chr4:83819293-83819378 (-)

CTTCATATCCAGTGATTAAACCTTTTCACTGCTGTTCACATAATGAACCGTTGTGATTATTGAATACTTAAATGAGGATATGTAAG

((((((((((...))))))).)))

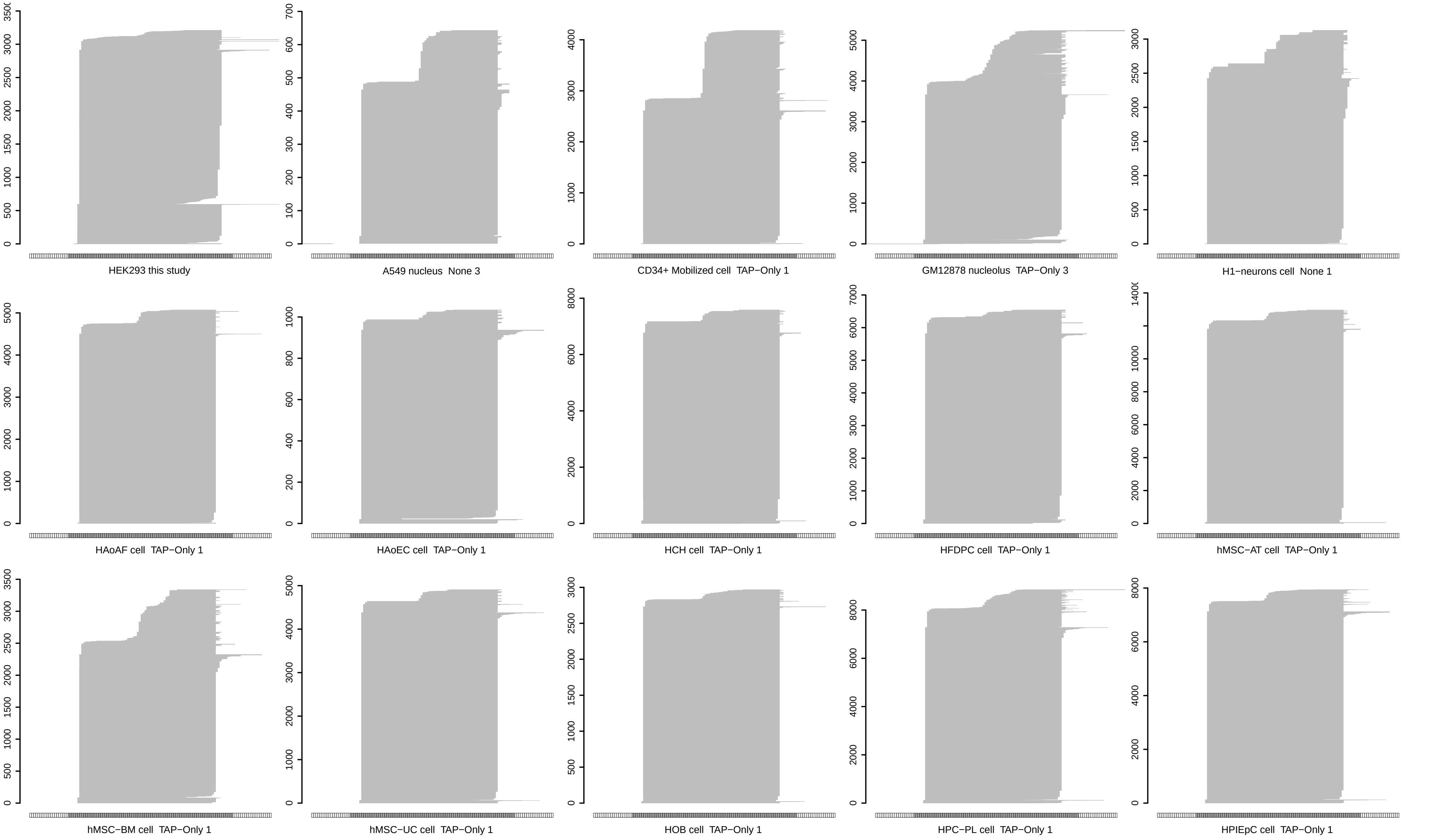

ZL7 chr2:25467962-25468024 (-)

CCCGGTGTGCGTGTGATGTAGACTTCAGAGCTTCCTCTGGAAGCAGAGTCCTGATGACACGGG

(((.((((...)))))))

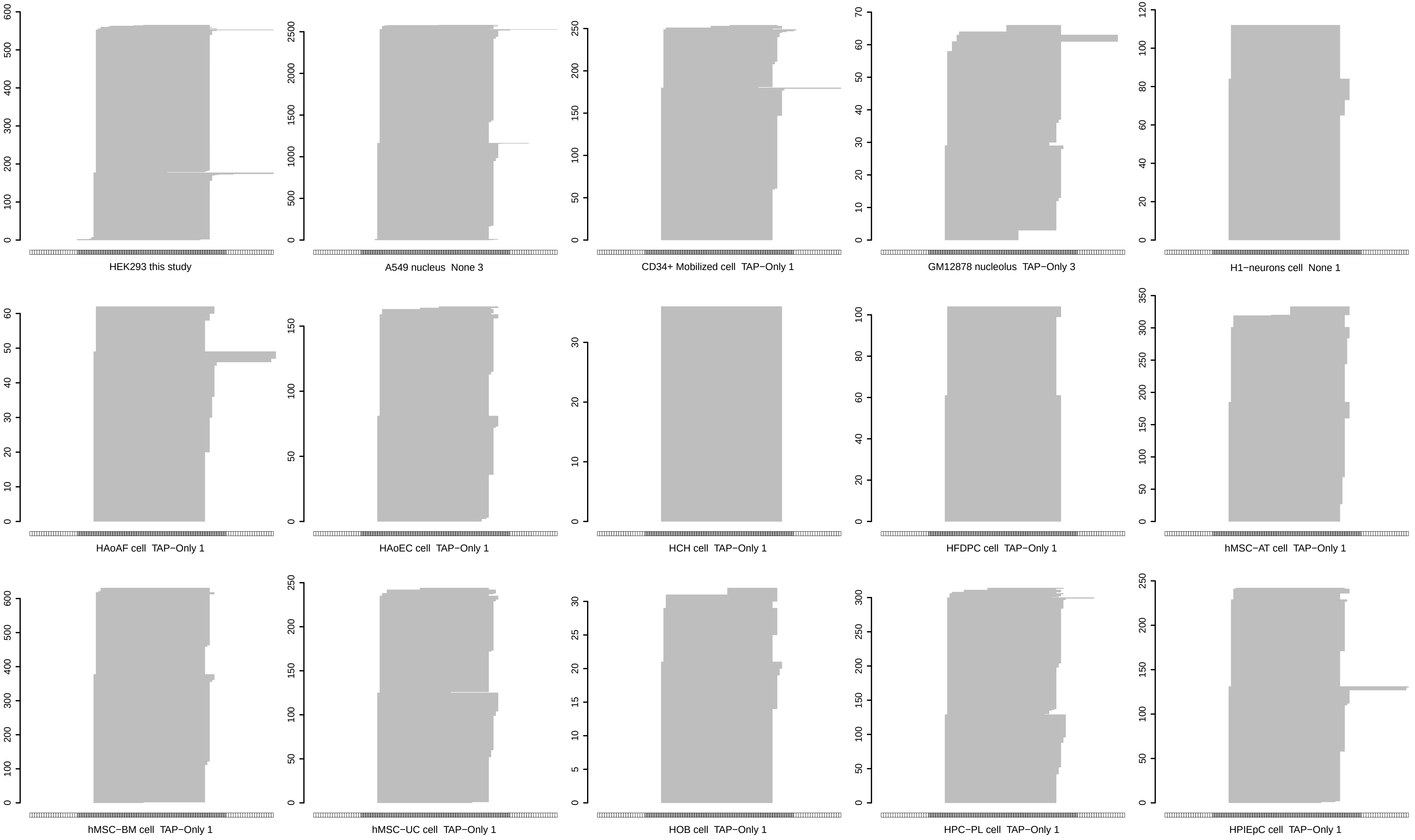

ZL8 chr6:31805240-31805322 (+)

TGGGAGTGGTAATGATGATCTGGTTGGACAAGAGTCTCTGAGCTTTTCTCTGAGGATCTTTGAACCCACCTGATCCACCTTCA

((((.((((...)))).))))

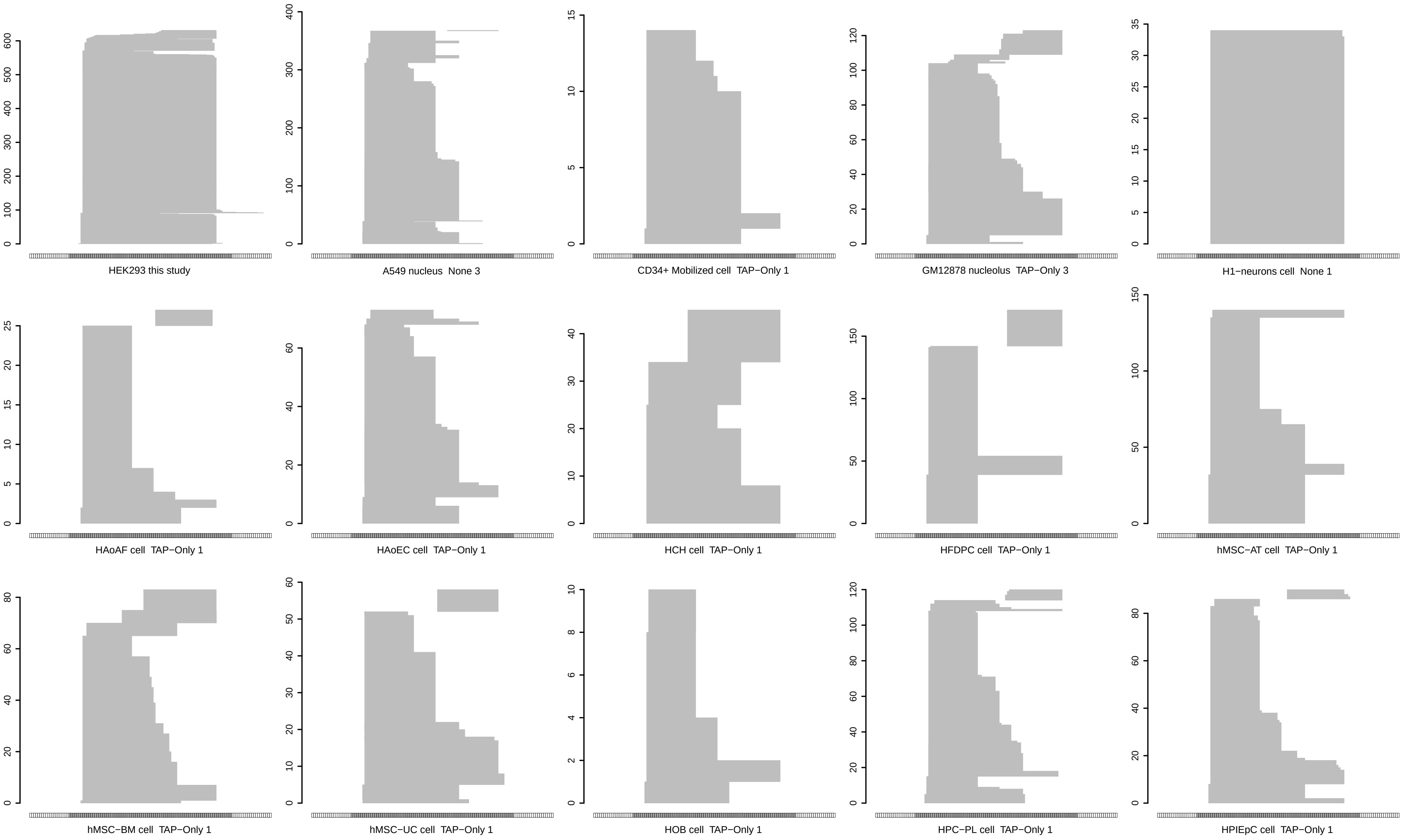

ZL9 chr14:70444850-70444913 (+)

GGCTCATACAGTGATGGACACCAAGCTCCCAGATTTCCCAAGGGAGGTGACTGAGTATAGAGCC

(((((((((...)))).)))))

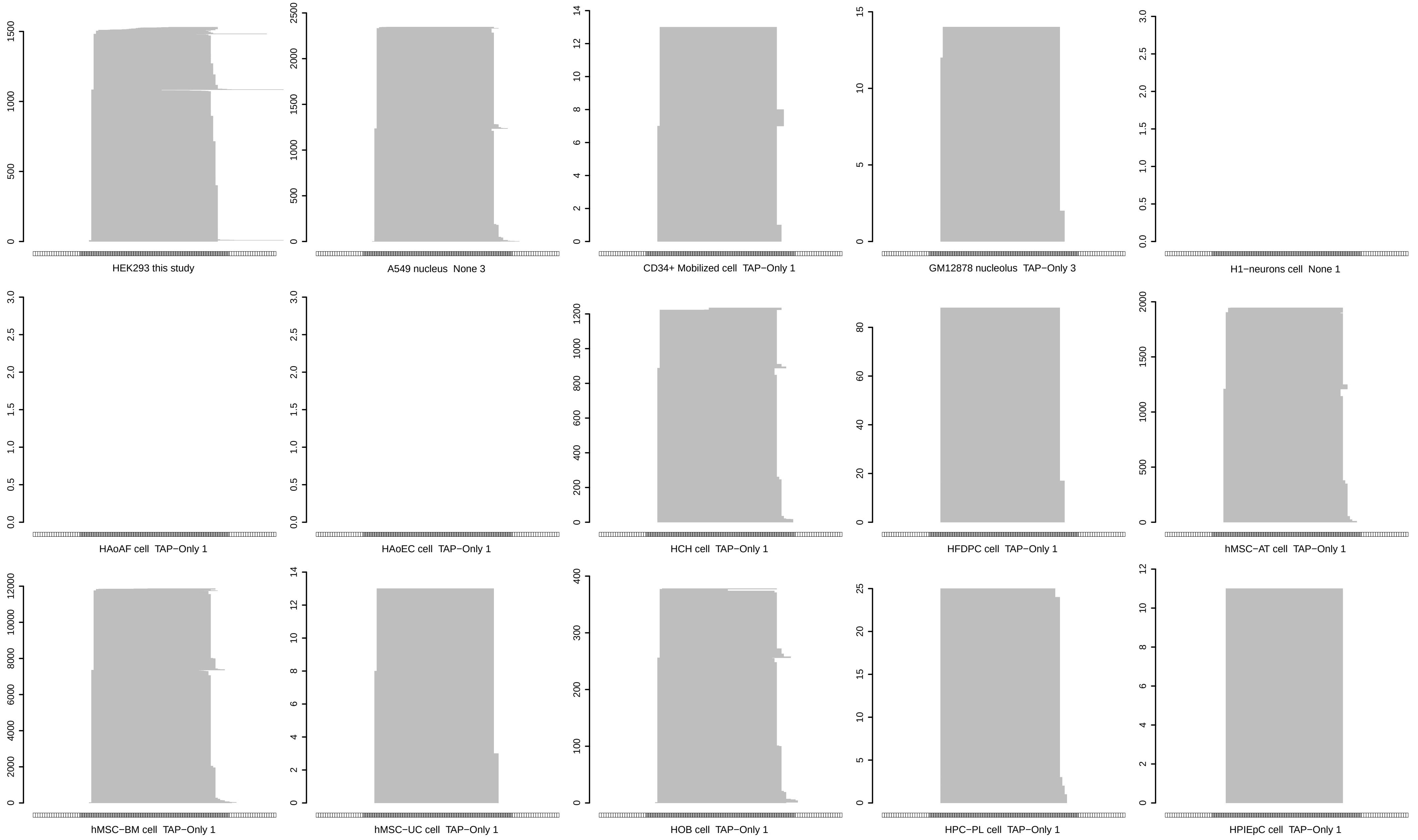

ZL12 chr17:80047836-80048015 (-)

CCCCGTGTGATGAGCACACAGCTGCAGCATAGCAAGAACCCAGGGGCTCTGGTGAAGCCAACACACGGGTCATCAGGGTTCCCTCAGACTGGTGCCCTCTGTTGGCACCCATCTCTGAGCCCCATGGGCCACATGTGGGCAGGACCTGGAAAGAACCTTGGCTGCAGAGGACTGAGGGGG

((((..))))

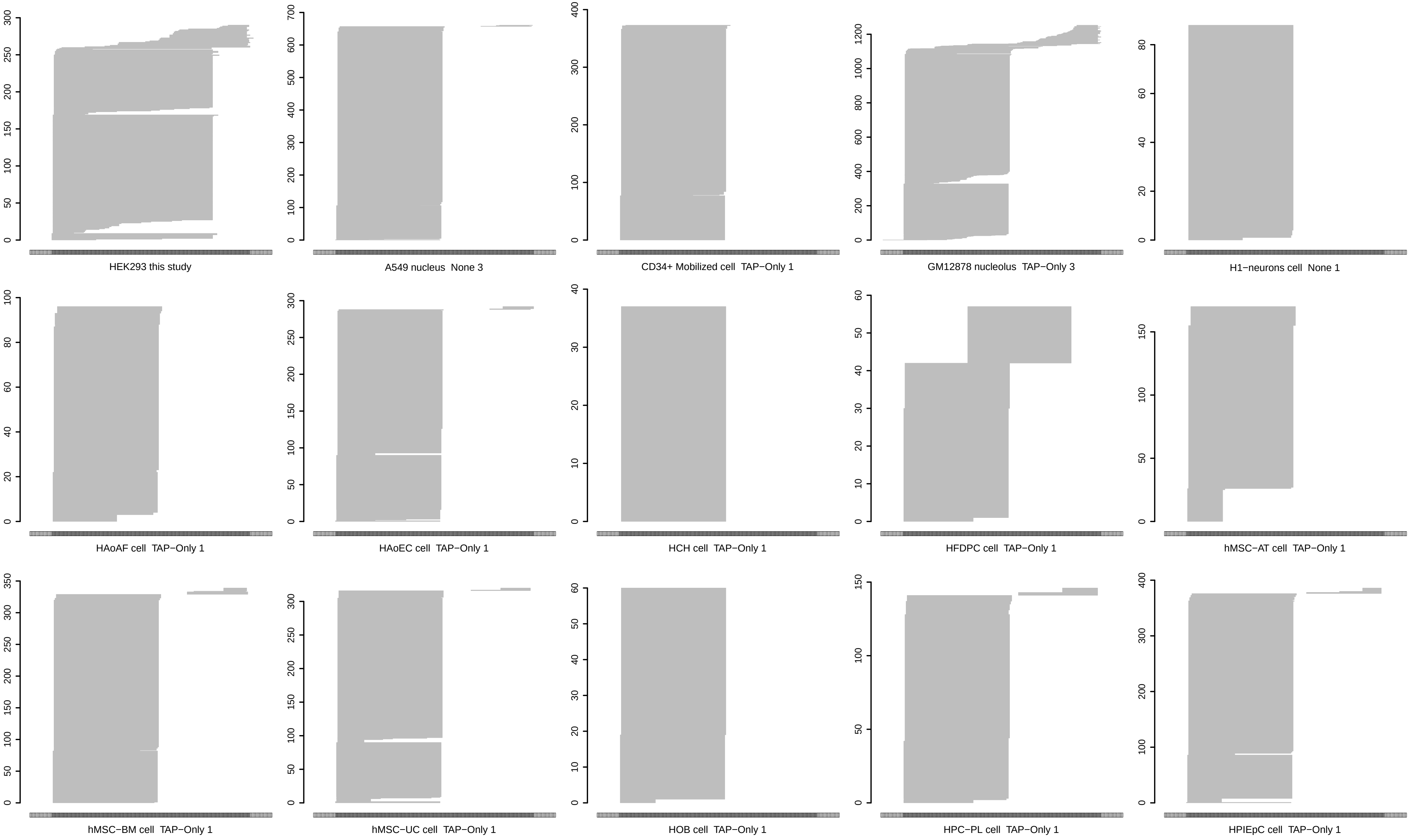

ZL13 chr4:4666053-4666150 (+)

GGGCTGGTGATGAAATGCAAGAGGTAGCCGGACACTCGTGAGCAGGGCTCATATGTCCAGCTGAGCGAGTGCAGGCTTCACTGCTCATACTGAAGCCC

(((((..)))))

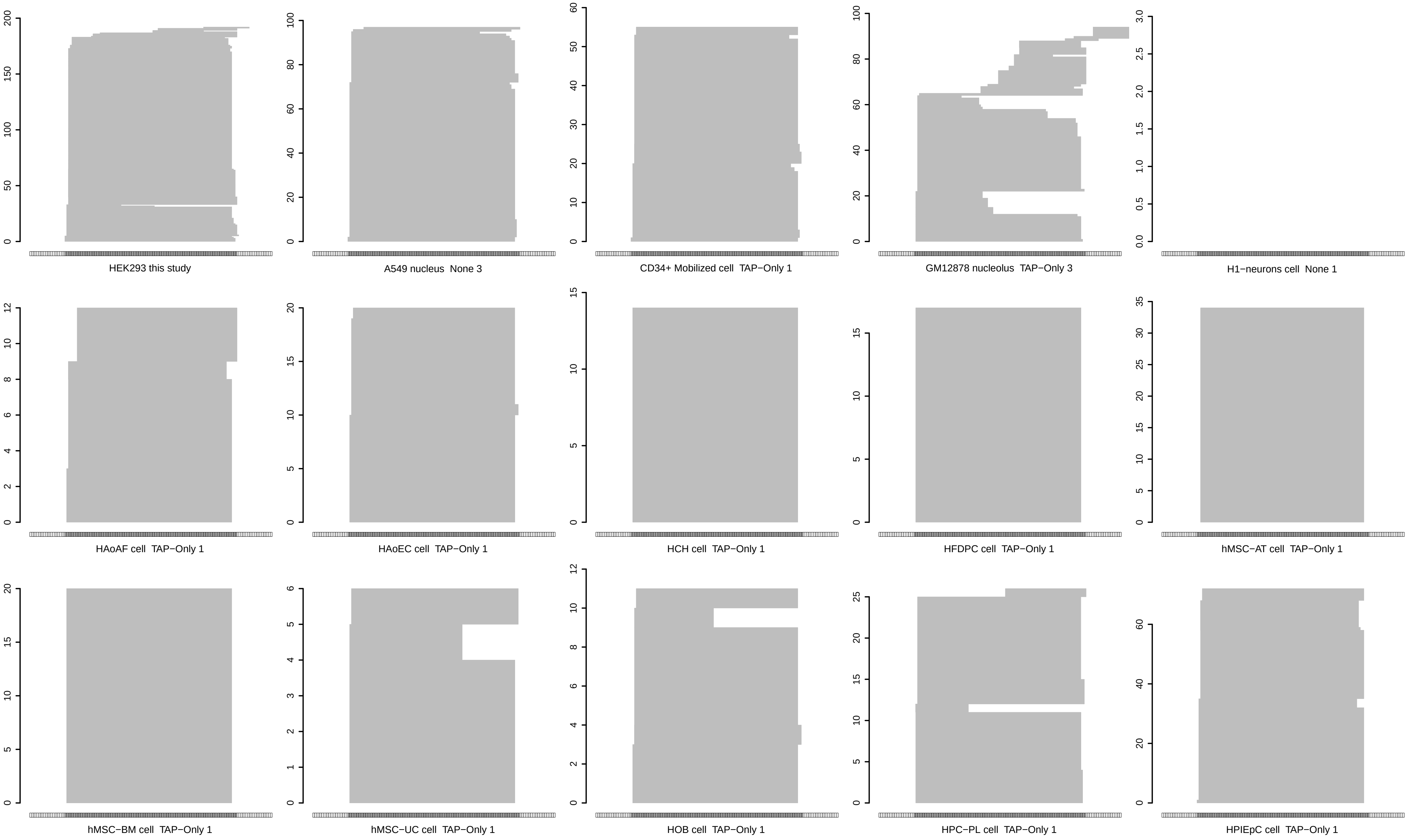

ZL17 chr4:6281978-6282046 (+)

GCCCAAGTGATGTGCCAGTGGGCCAGGGCCGGCACCTGCCTGTCGGTGGCTGCCCCAGGGCTGATGGGC

(((((...)))))

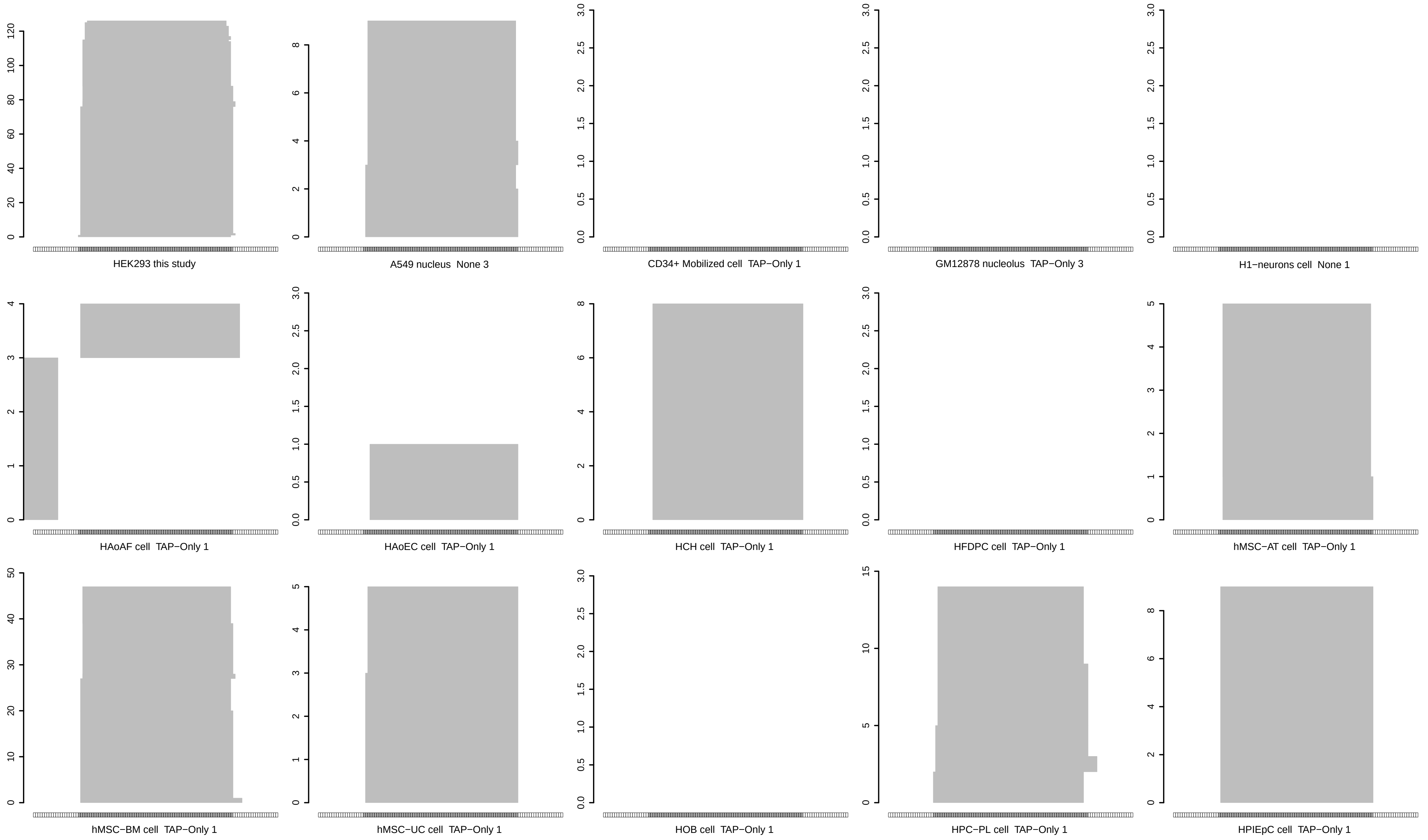

ZL18 chr11:134021184-134021259 (-)

GTGGCAATGATGGAGGGAACTTGCATGCTAAGCATCCAGGATGCCATGCAGCAGACACCAGGGAGCACTGAGCCAC

(((((..)))))

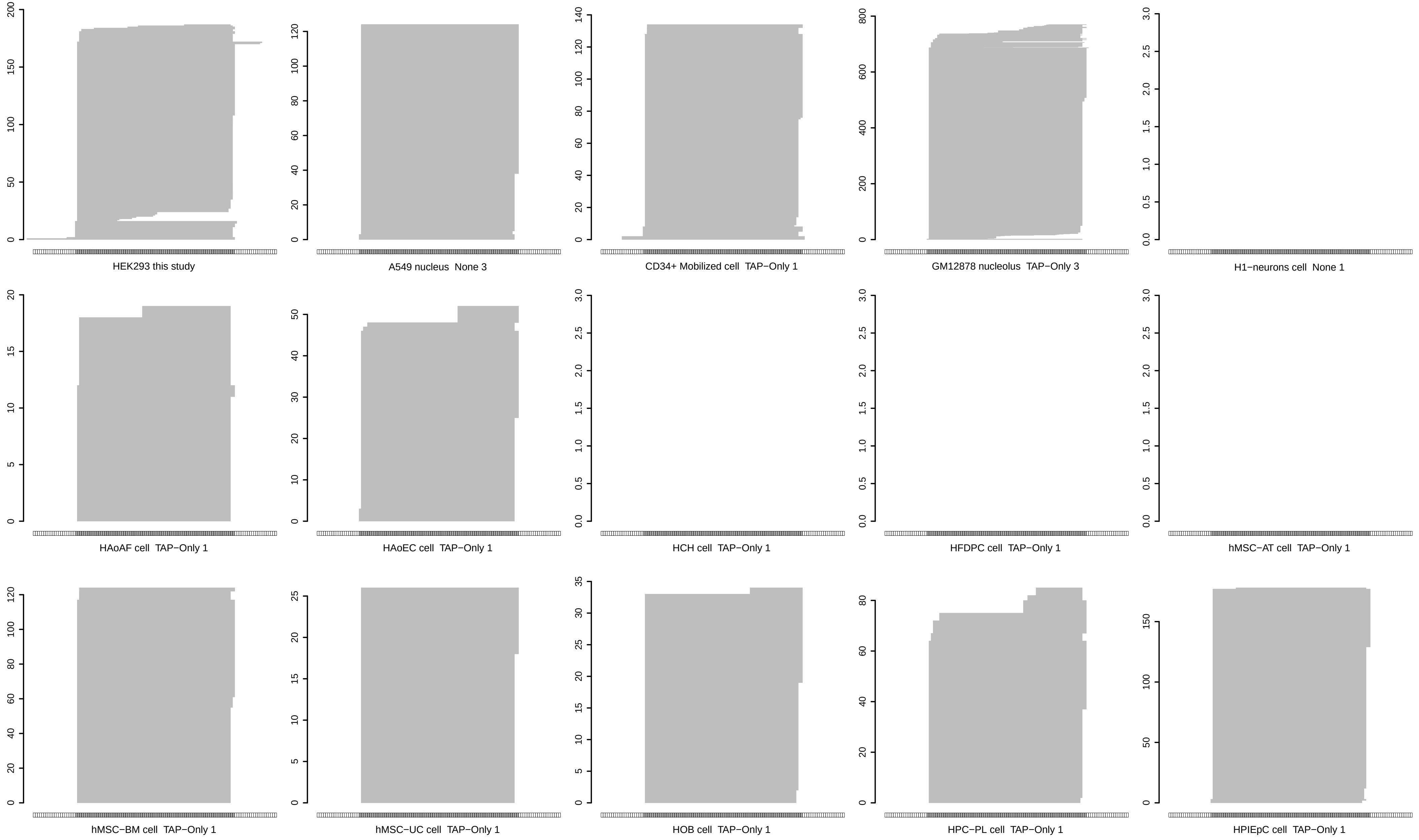

ZL19 chr12:123270512-123270631 (+)

GGGAAACAAGTTTAAAATGATGTTCCCAAGGCATGAAGAAAGGAGGCAGGACTCTGCTTCTGGTCTTCTGTCTCCAAGGCCCATTCTGTGGGCTGGCAGGTCTGAATTAACTTGTTTTCT

((((((((((.((((...))))))))))))))

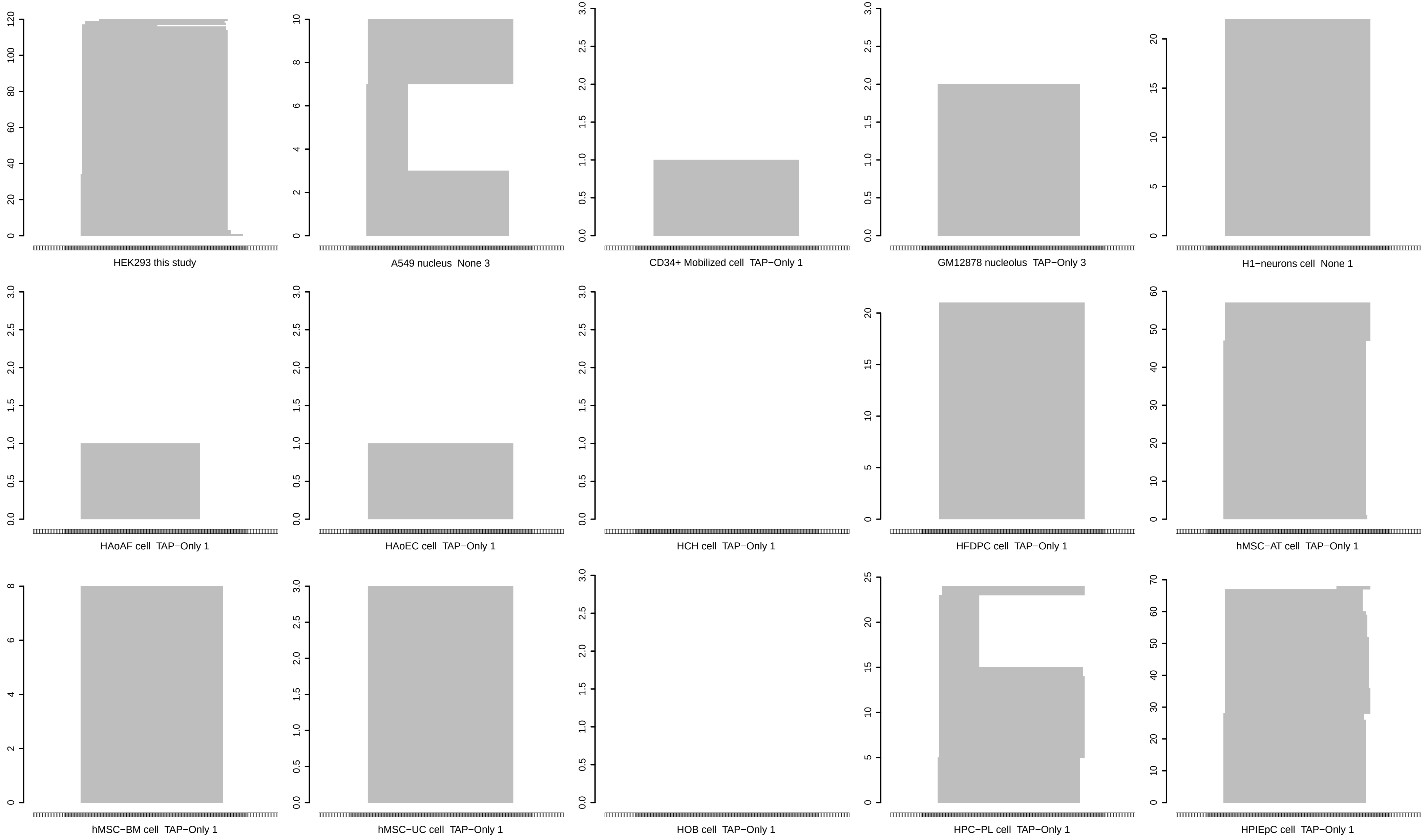

ZL22 chr3:47703061-47703178 (-)

TTCAAGGCTCTGTGATGTGTAGGAGAAAATGTAGAACCATAGAATTTCATTATTATTACTATTTTTTGTCTCATTTAGTCCTCTCATTTTTCTGGTATAGAGGGACTGAGAGCCTGAA

(((.((((((...)))))))))

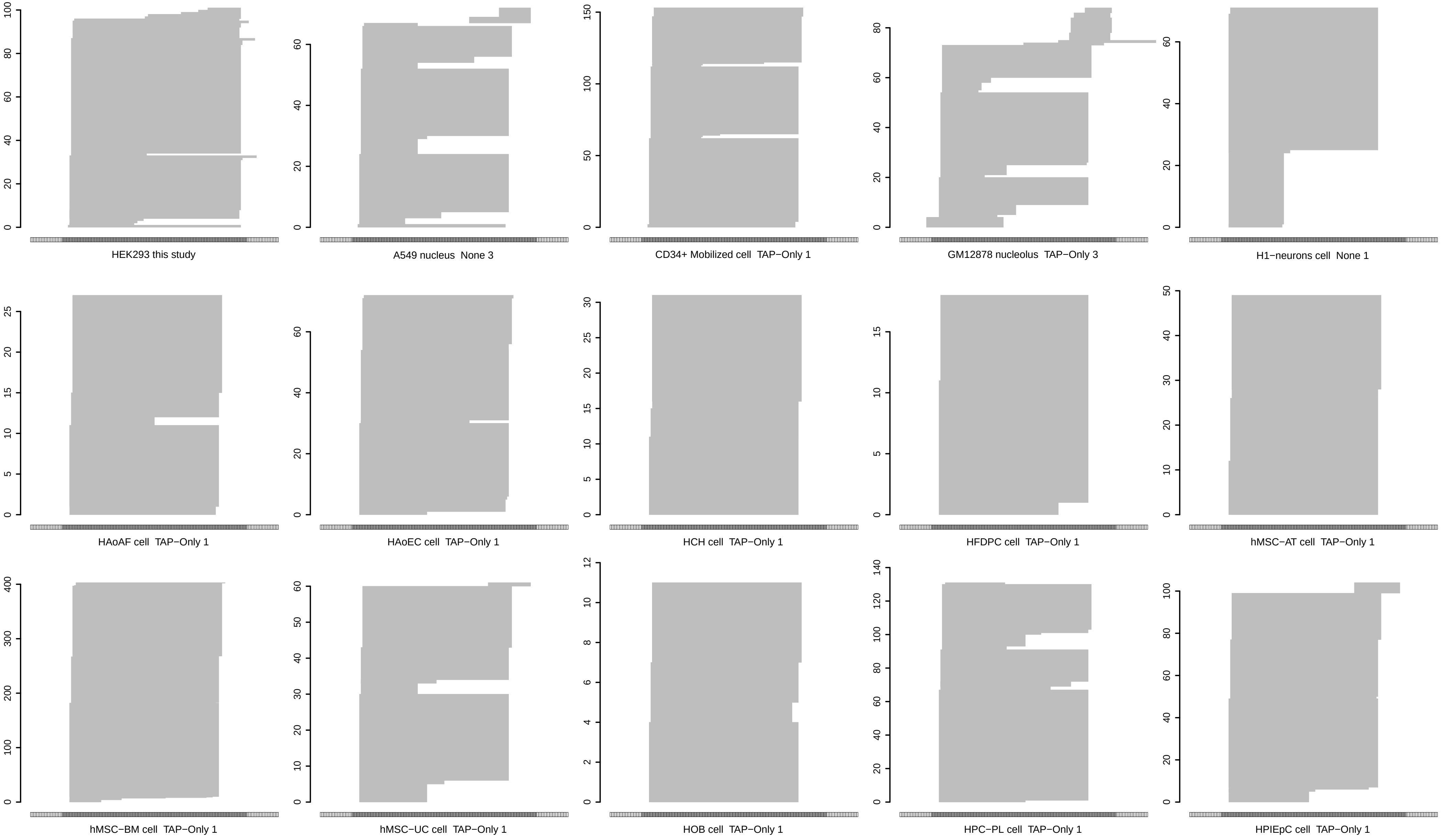

ZL23 chr17:37881867-37881893 (+)

CTGCCATGATGCTAGACTCCTGAGCAG

((((...................))))

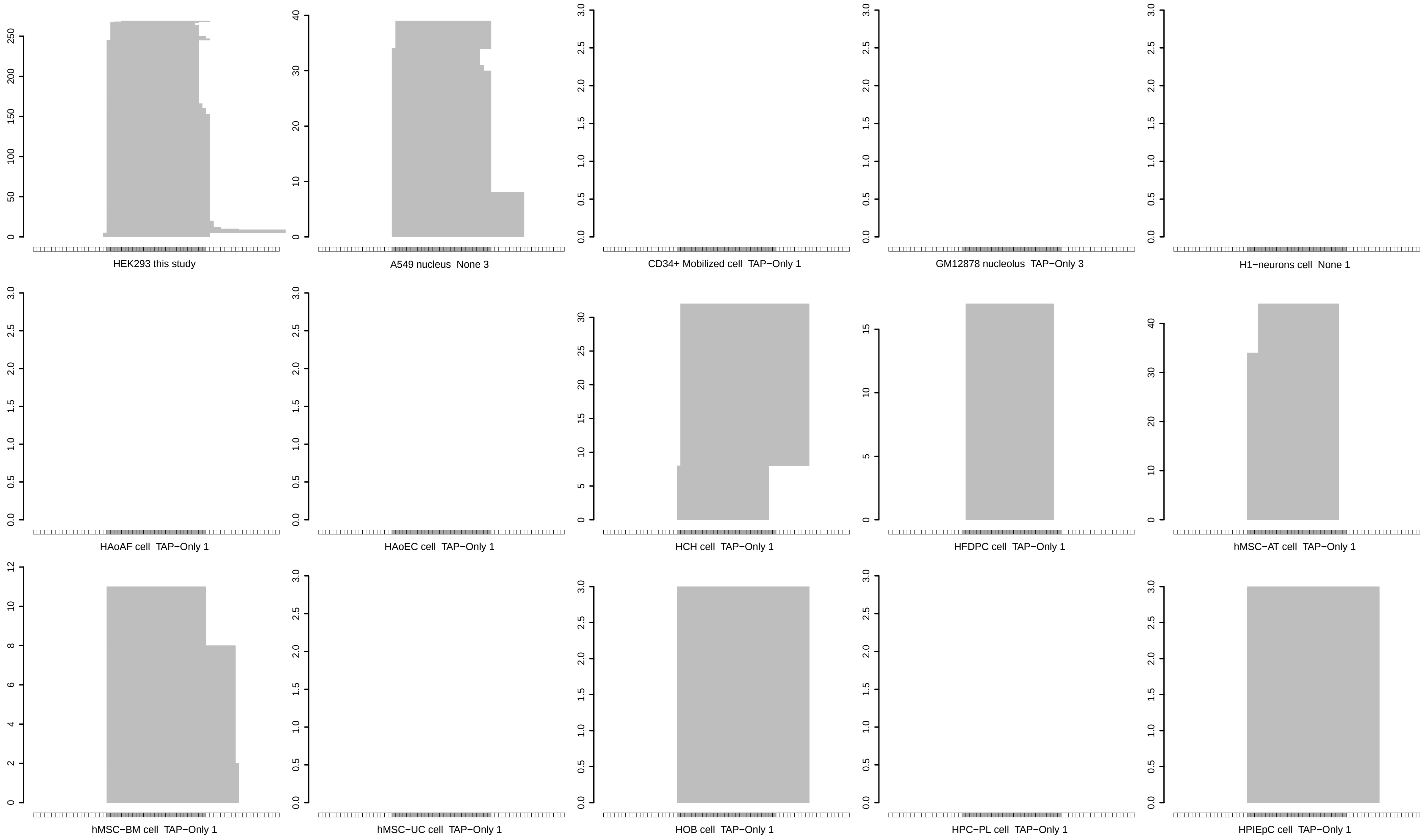

ZL24 chr10:76781491-76781549 (+)

GGTTCCTGAGATGATGCAGCTGGAATGTGCTTGGCTGGCTGGCTGTCCTGATCAGAACC

((((.((((..))))))))

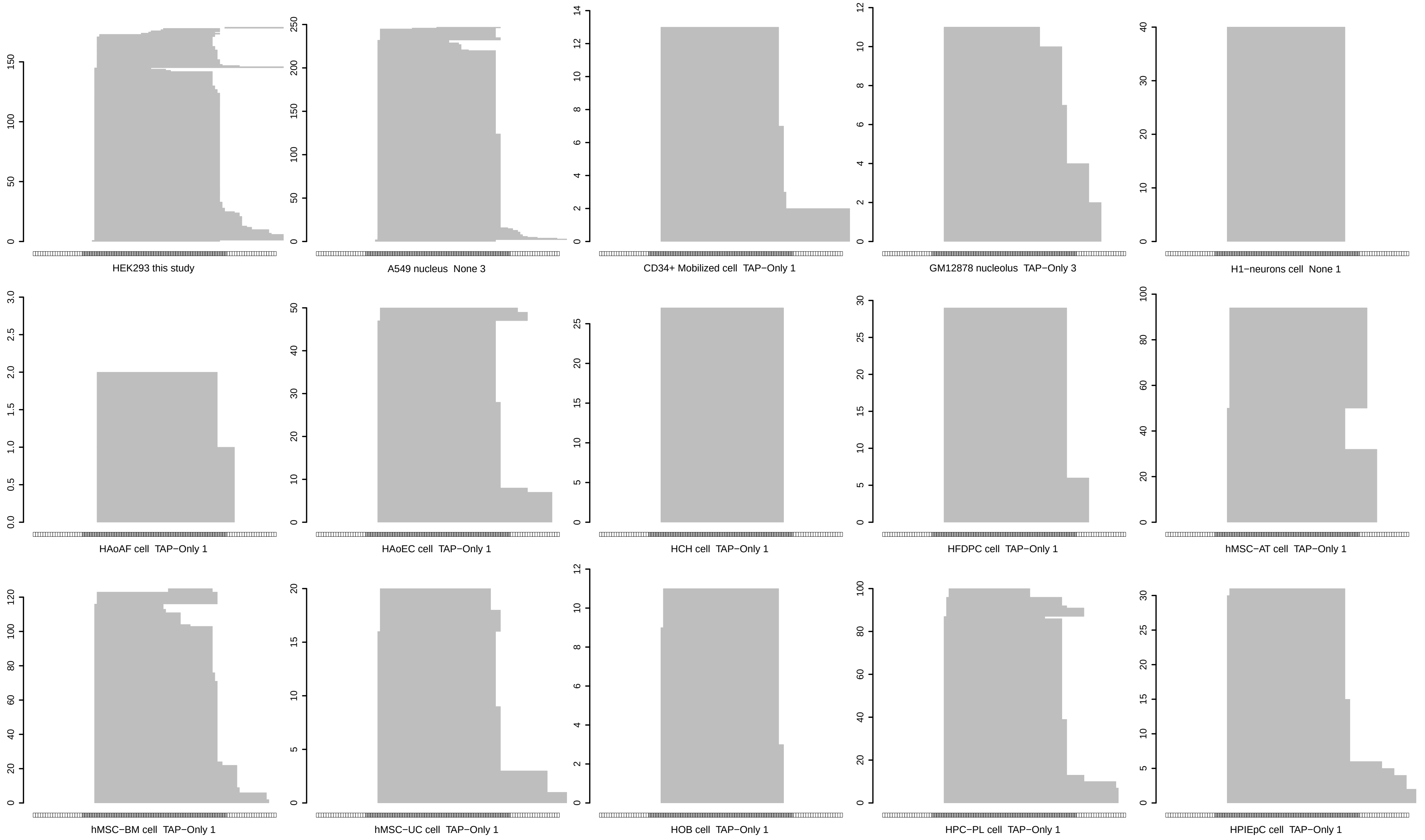

ZL25 chr8:144588356-144588419 (-)

GGCCAAGTGATGATTTTCCTCAGGGCGGACCCAGCAGGGCCTCTCCTTCTGGATTCTGAAGGCC

((((..))))

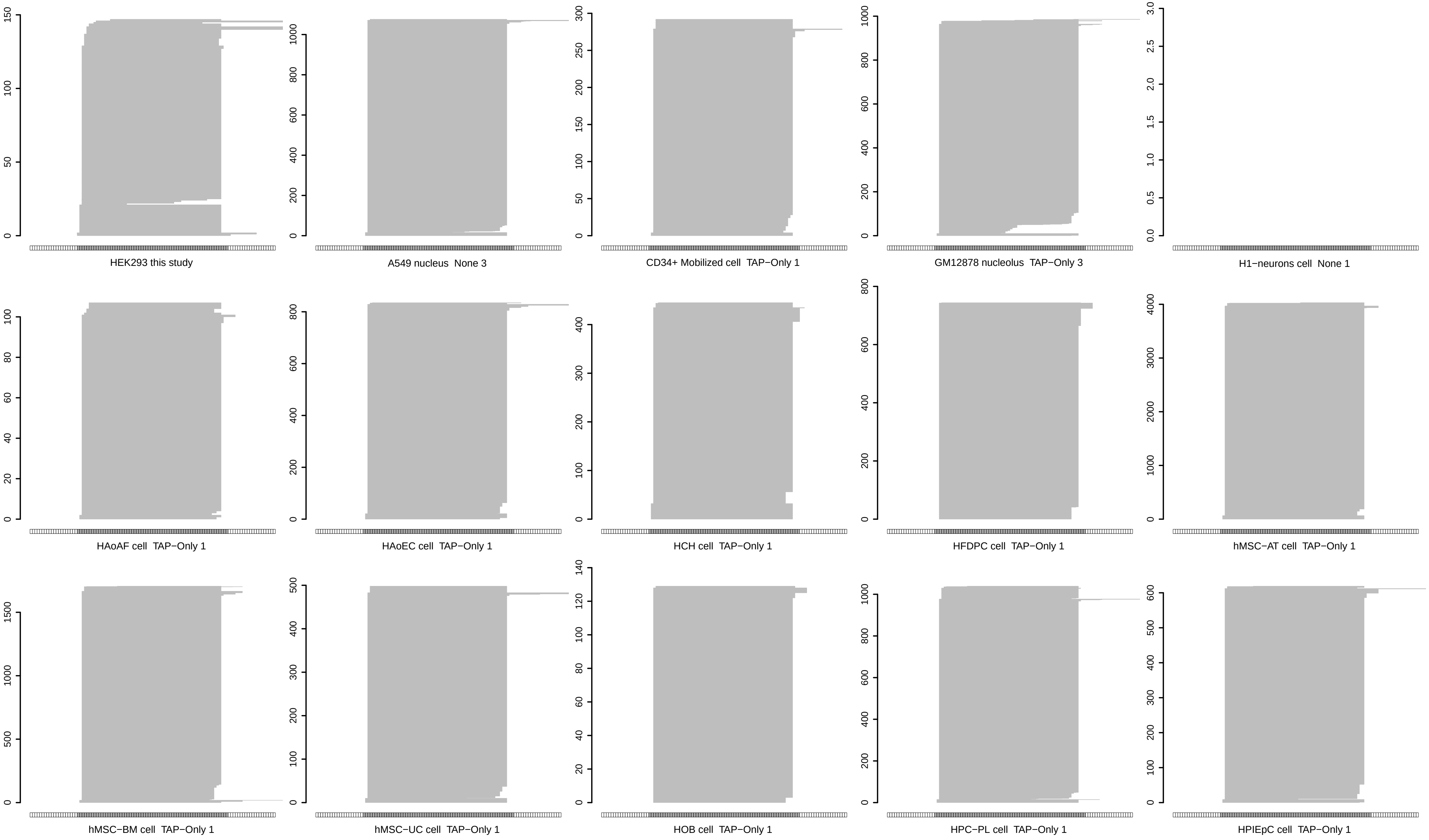

ZL27 chr17:41105504-41105535 (-)

GGGGCTGTGATGTTTATTAGCTTCTGAGCTCC

((((((....................))))))

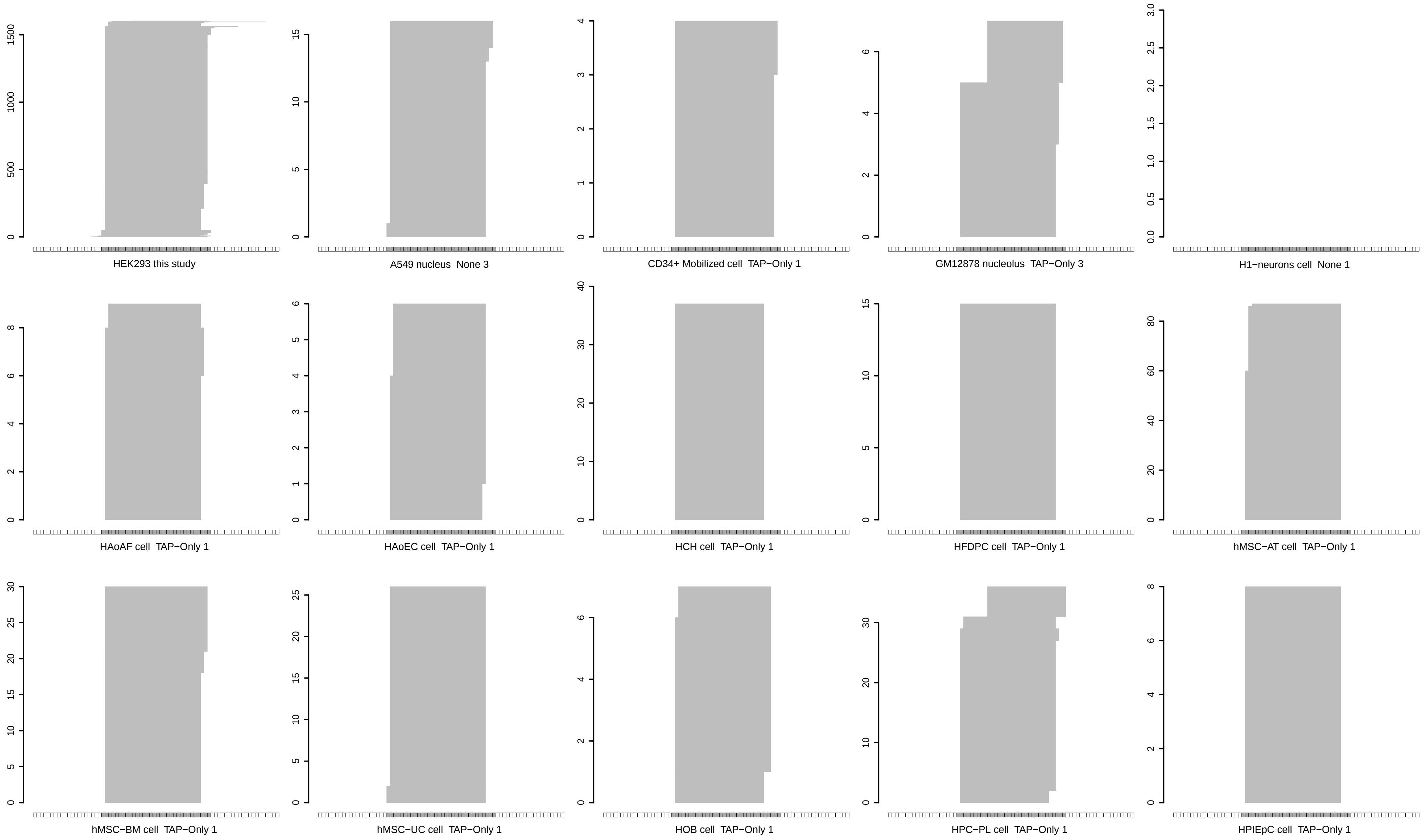

ZL30 chr6:31506788-31506858 (-)

CCTAGCTACATGATGCTTGCAGAGCCATGAGCACATGACCTCTGTTACCCTTGACAACCTGACAGCTGTGG

(((((((..))))).))

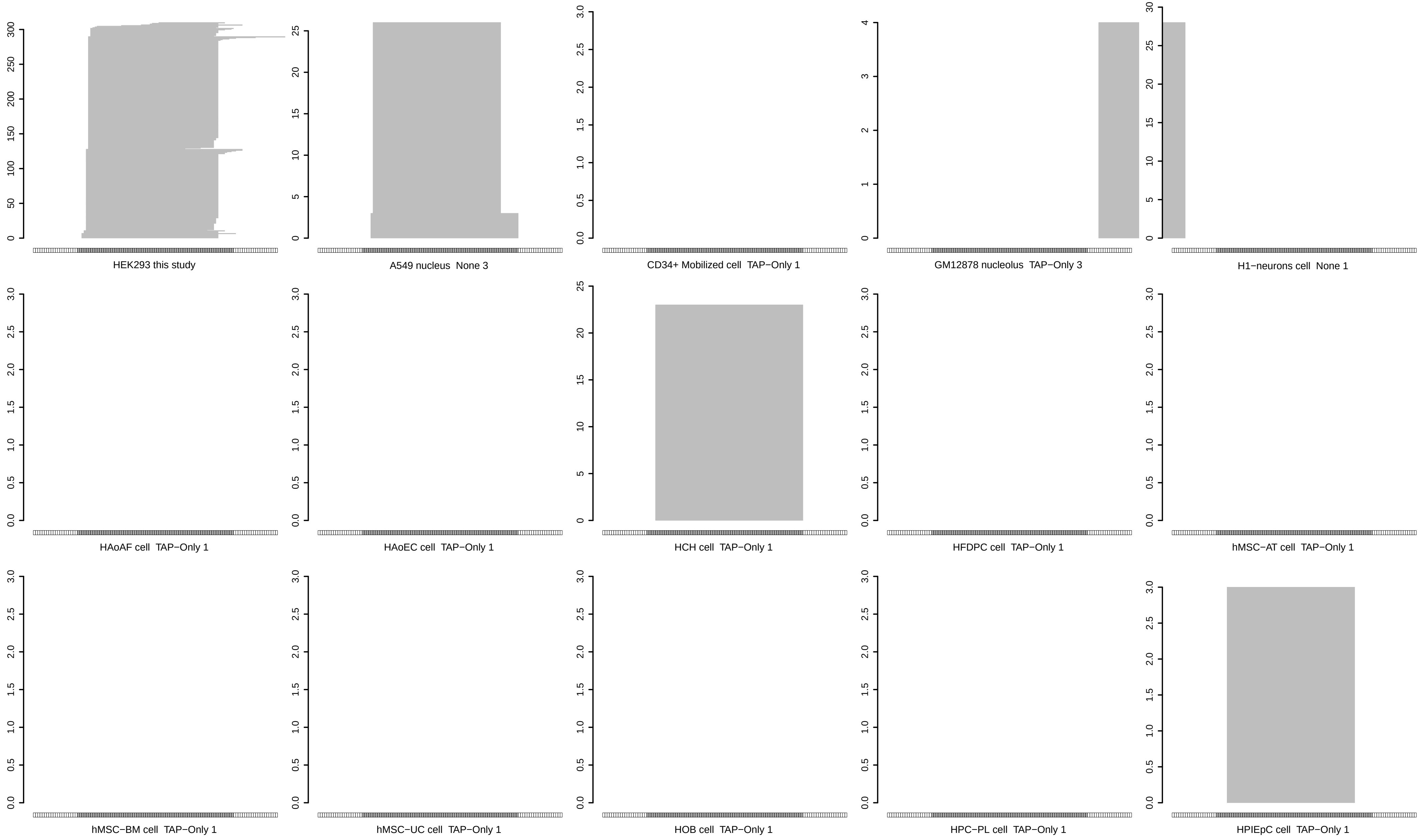

ZL31 chrX:79955292-79955351 (-)

TGTGTATCCAAGATAAGATGATGATCTATACTCTTGTAGAACTGAATATTATGGATCACA

((((.(((((.((((...............................)))).)))))))))

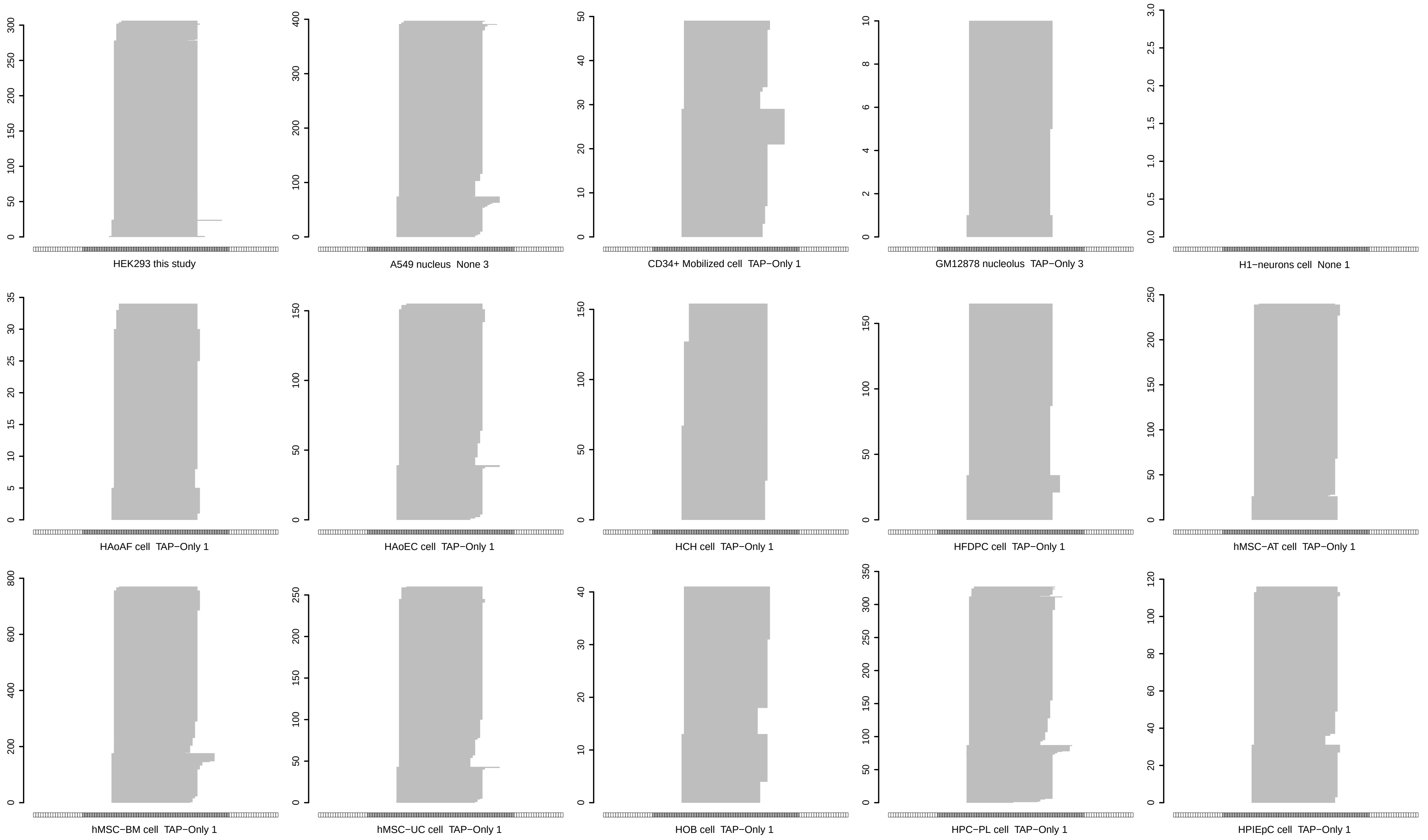

ZL34 chr2:74685527-74685566 (+)

GGGACGATGATGATTGGTAGGGCAGAGCAATCTGAGTCCT

(((((..............................)))))

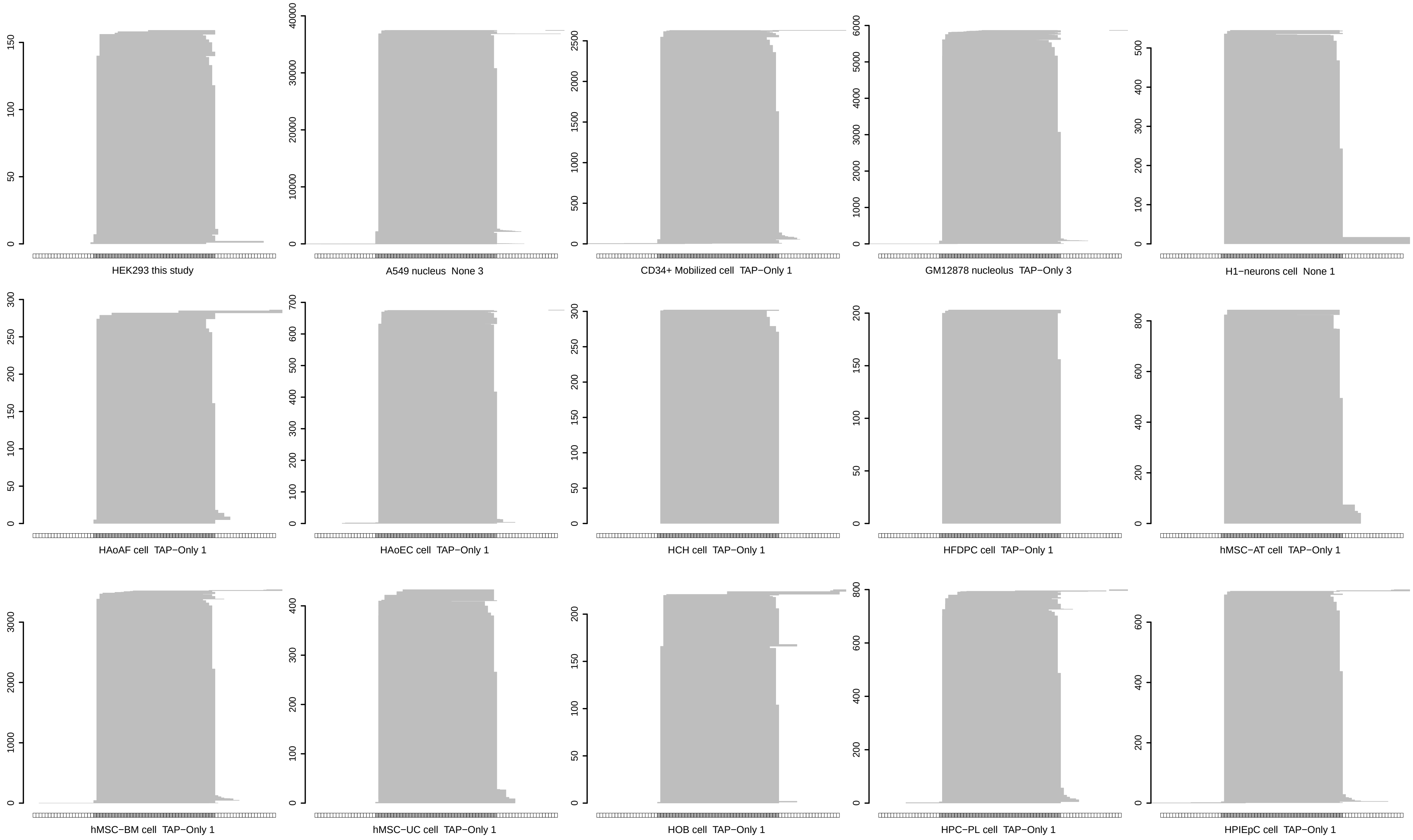

ZL35 chr17:26648515-26648560 (+)

GTGCTGTGATGCAGTCATGGCTTACCACAGCCTTGAACCTGAGCAC

((((......................................))))

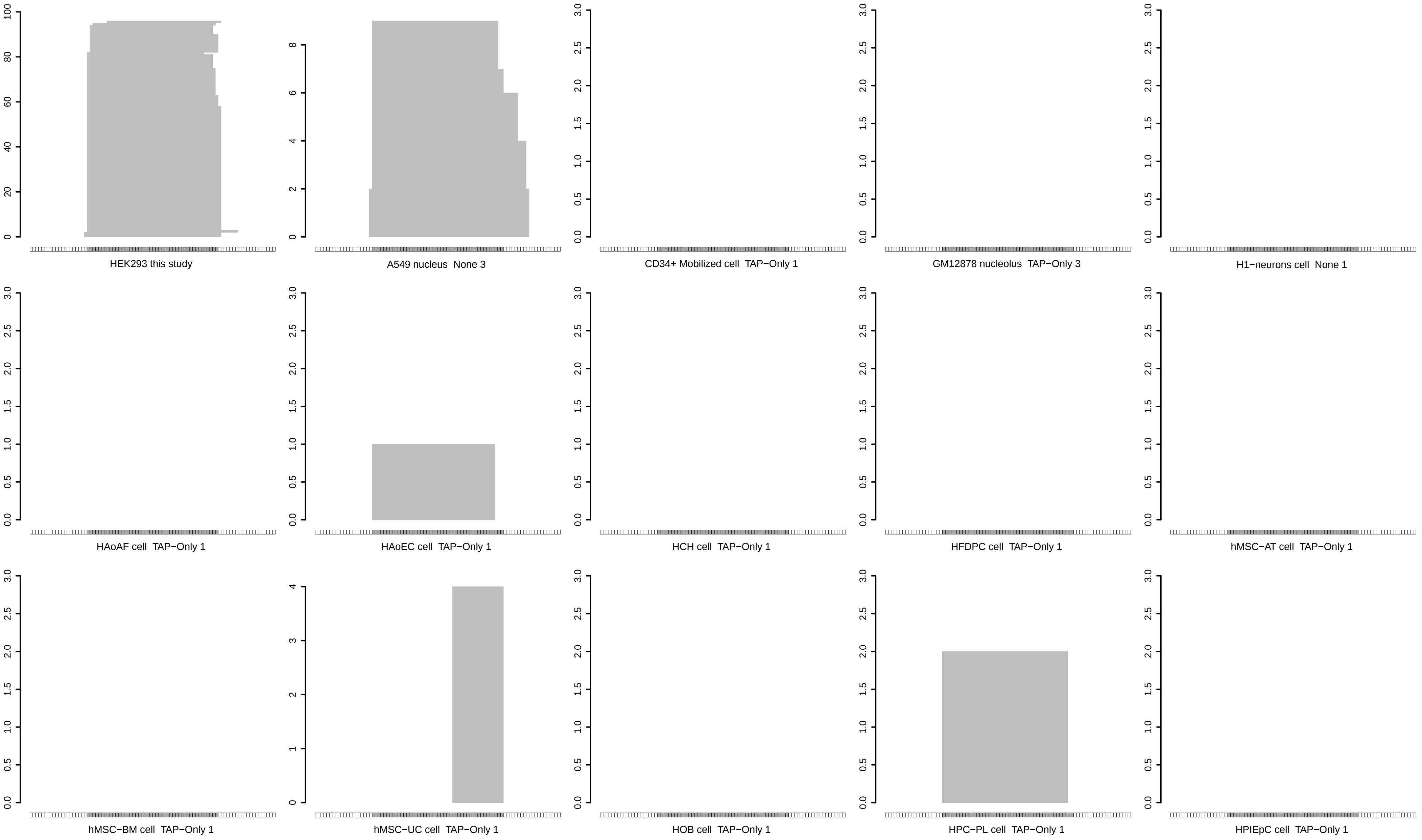

ZL36 chr4:185561291-185561394 (-)

TTGGAGAAGGTCAAACATCACCCTCTCCTGTGGTTAAATTGAGATGGTGGCACTGGCTGTCTTCTATATTATTGCTGCACCTTTCCTCACCAGGGGTGCACACA

..(((((.(((((....(((((.((((..((......)).)))).)))))...))))).)))))........((.((((((((((.......))))))))))))..

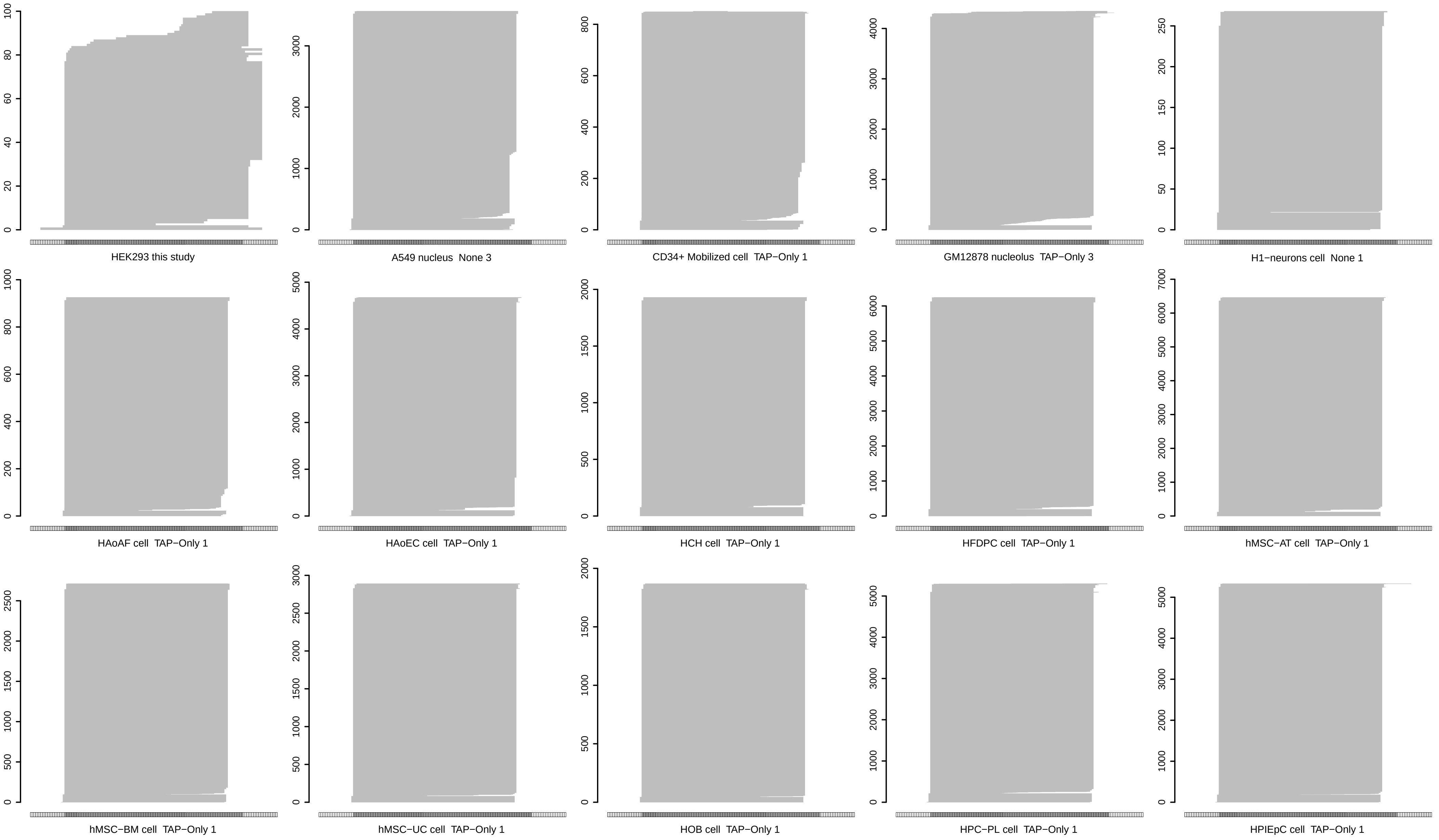

ZL37 chr5:14464092-14464176 (+)

```
GGGGCCTGCAATGATGGCTGTGAGCTGCAGGACTGAGCACAGGTTGGAGTCCTGGAGTTTTGTGTGCATCTGAGGTTCAGGCTCC
((((((((.....................................................................))))))))
```

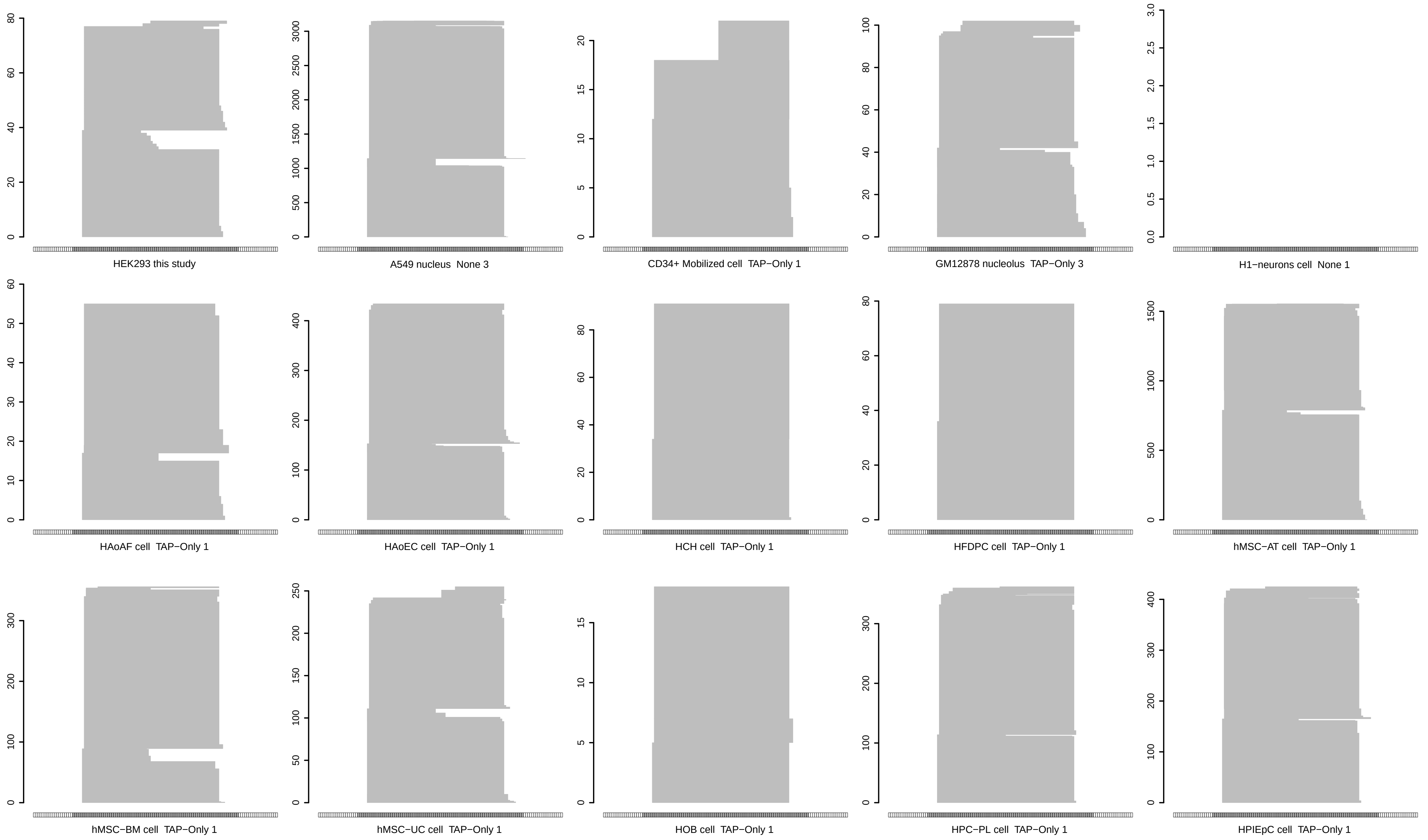

ZL40 chr19:33692841-33692973 (+)

GAGCTGGCGGGCGTGATGCTTTGCTCAACCTGAGCGGTATACAGTTGGCACTCCATGAGTGCATGCTGGCTCAGTGCTGCAGCAAGAAGCTGAGGCCCCCAGAGGCCTACAGTGATTCCTGACTCTGCCCCTC

(((..((((((..))))))).)))

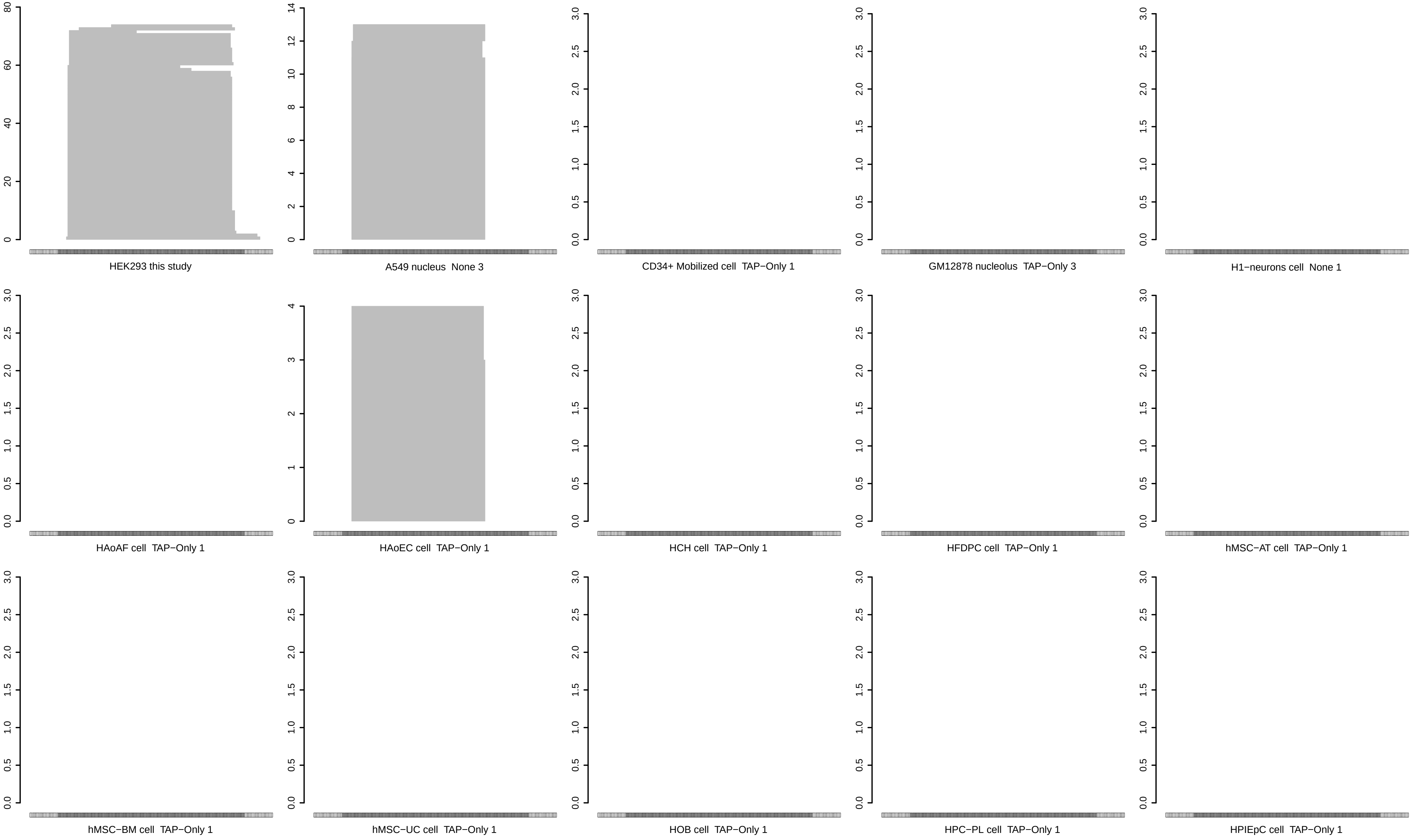

ZL41 chr15:22872576-22872695 (+)

GATCCCATGAAGAATTAGAAATTCTCACCATTTTCAAAACTGAAAAGTCCCTTAGCAGTTGTCCCATGCTTTGAATTGCTTTTTAAGCATTACGGTGAGGTGAGTGTAAGCCTGAGGATC

(((((..)))))

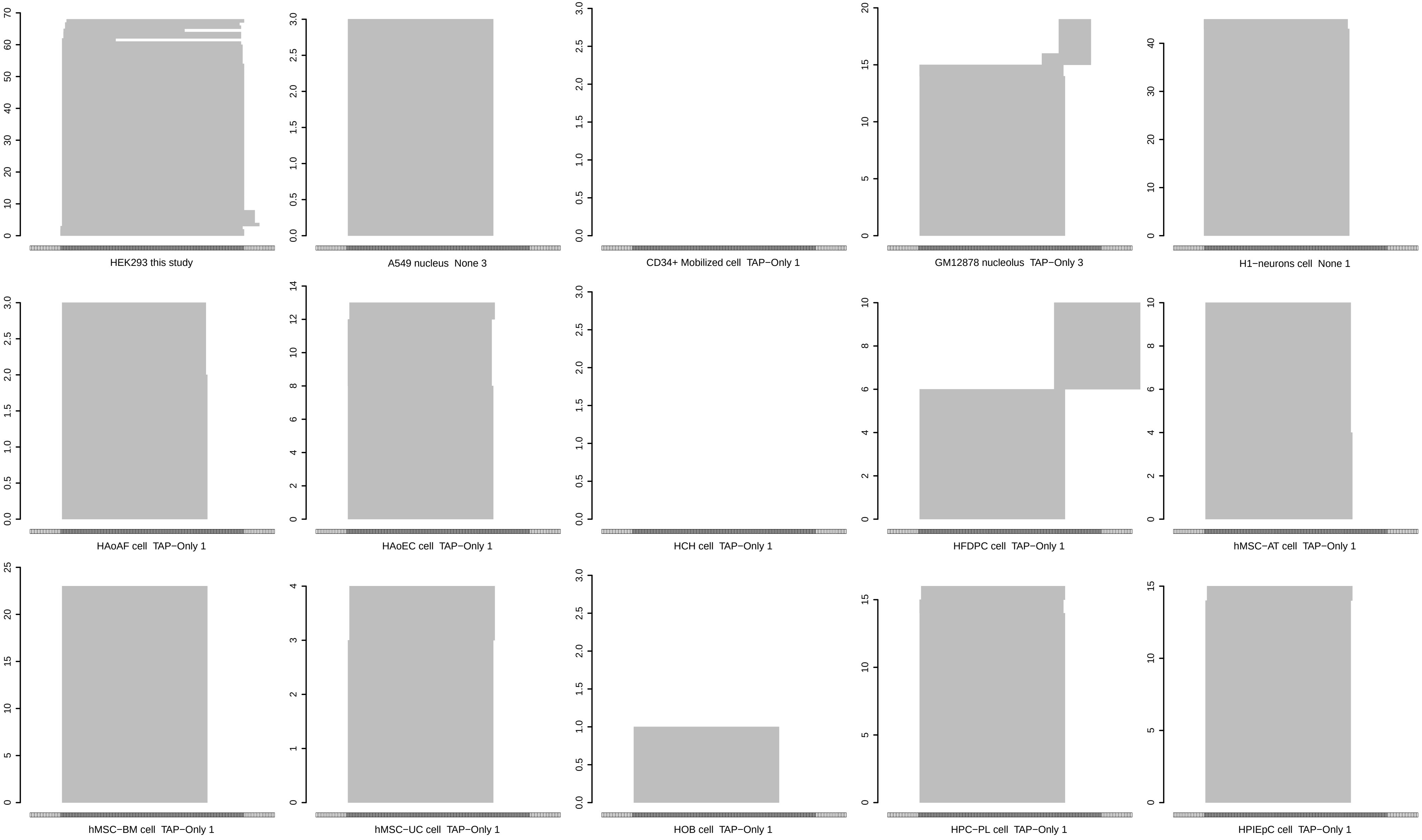

ZL43 chr1:8554860-8554973 (-)

AGGCCAGTGATGTGGCACCTGCAGGTTCATTTCCTAATCTGTGTCTGTGCTGCAGGTTTGAGGTGACTCTCAAATGAGATCCTAATAATAGCACCTGATACTTAACTGAGGCCT

(((((..)))))

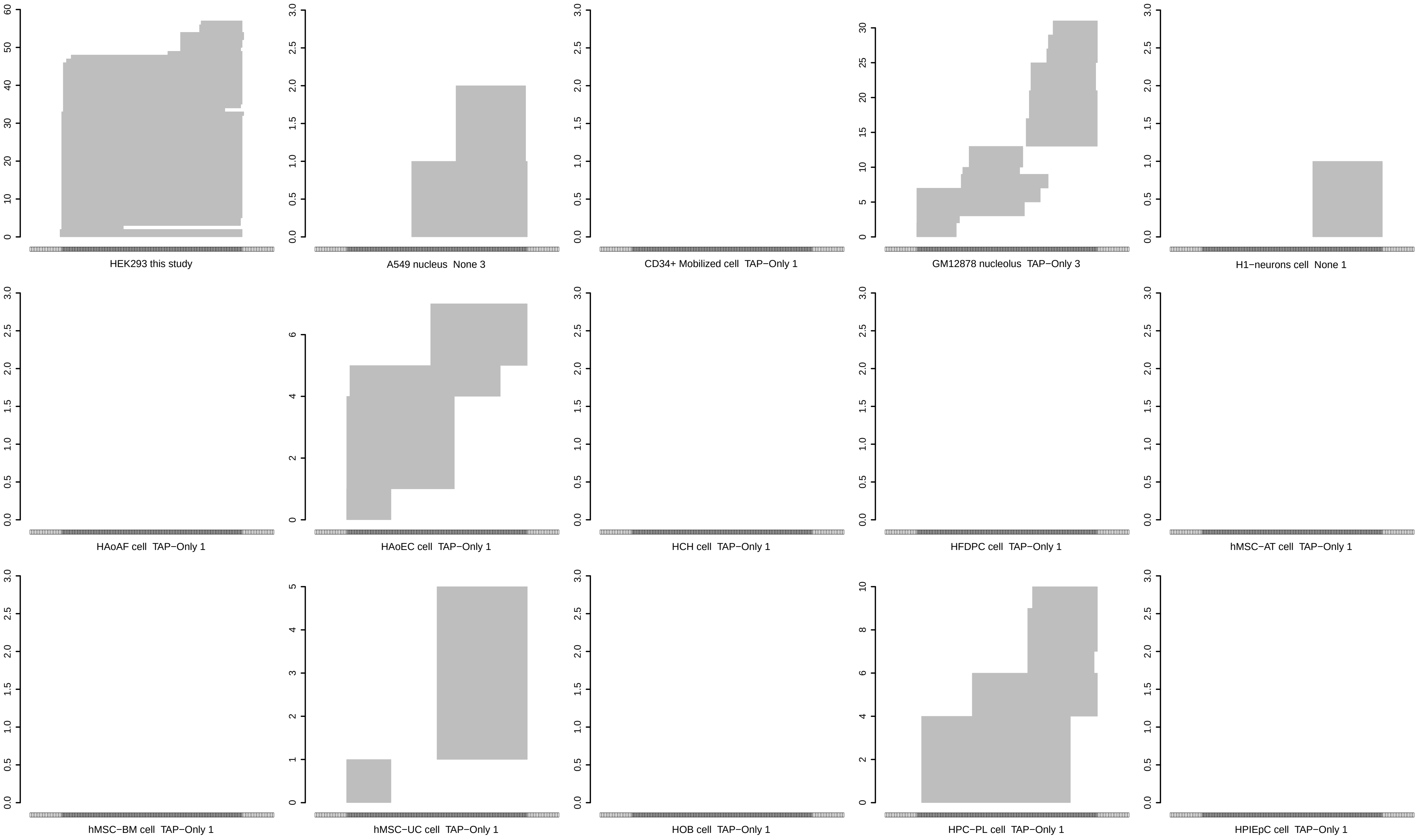

ZL45 chr1:161325012-161325057 (+)

AATGGCTAATGATGTTAAGCACCTGTTCATGCACTGATTGGCCATT

(((((((((............................)))))))))

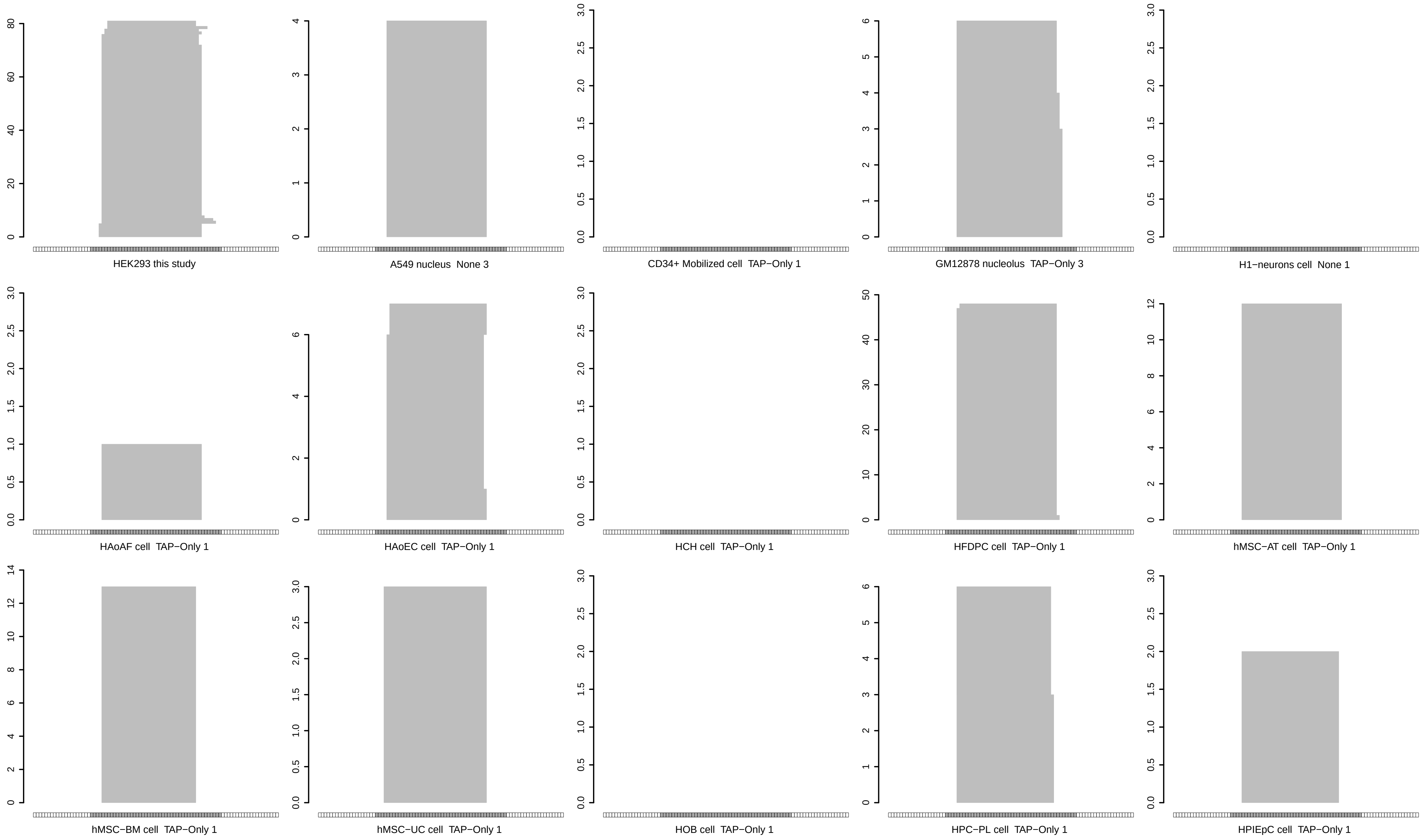

ZL46 chr12:124854765-124854805 (-)

CCCTGTGATGAGCAAAGGCCTGGCCTGCGGGTTTCTGAGGG

((((.................................))))

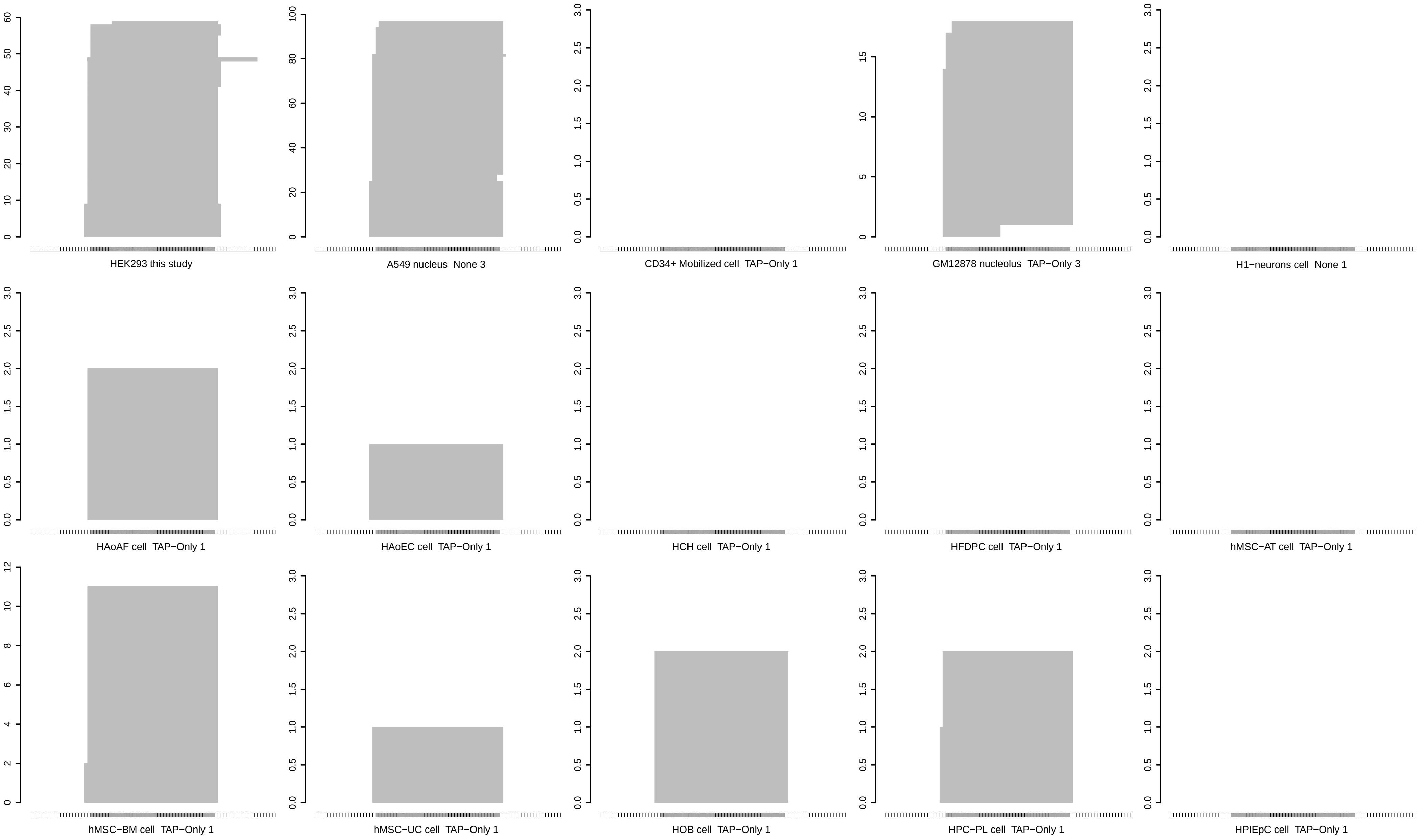

ZL47 chrX:68858403-68858442 (+)

AAATAGCATTGTGATGATGCCAAGAGCTCTGAATGCTTTT

(((.((((((....................)))))))))

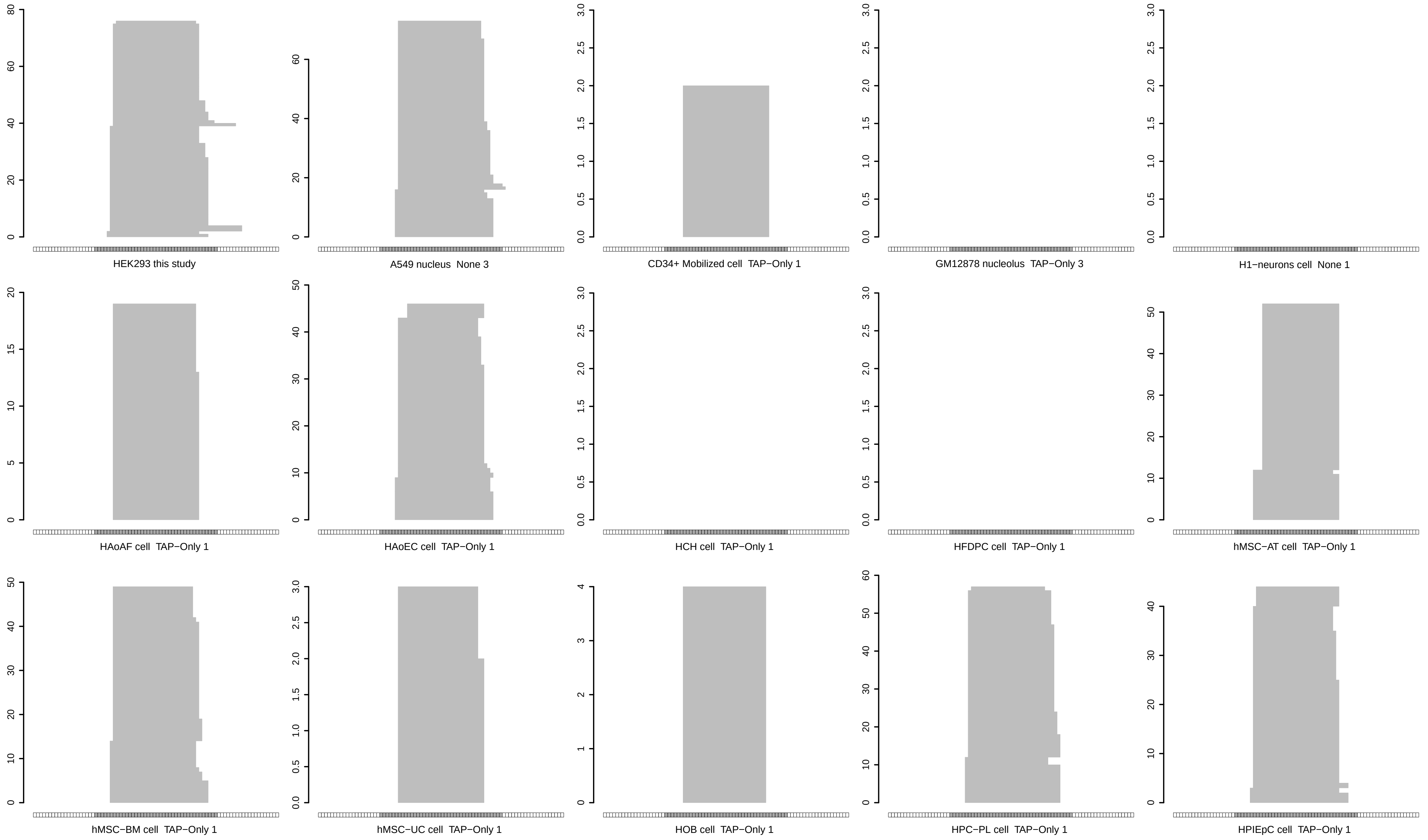

ZL48 chr6:35855531-35855613 (-)

AAATGCTATGATGAGAATTCTTCTTACACGCATGAACCAAAAAAAAACCTTTCAAATGAATAAAAATATTCACTGAGCATGTT

(((((((...)))))).))

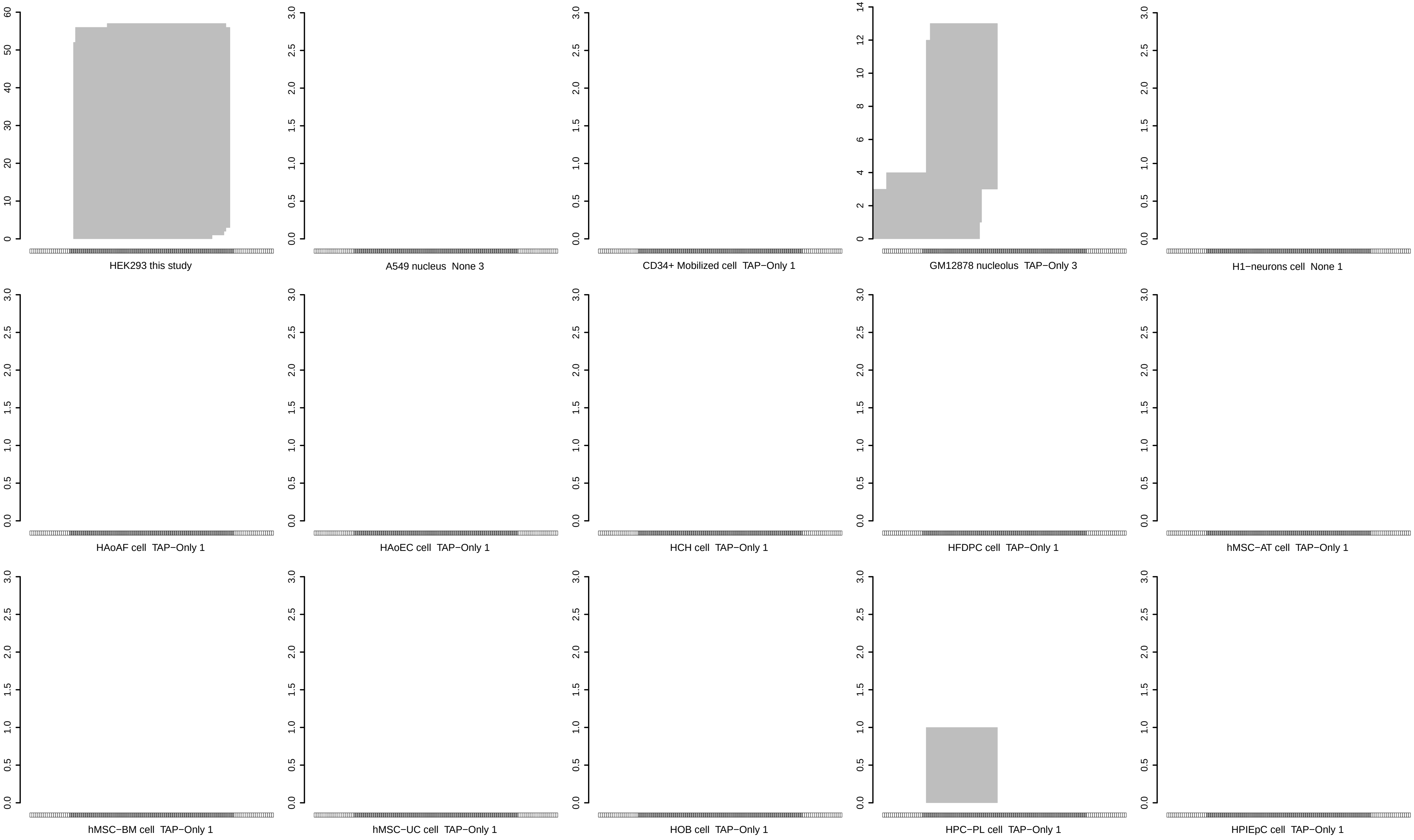

ZL49 chr1:38265102-38265139 (+)

GAGGCATGATGACTCCAACTGTGGAGACTGACTGGCTC

(((.((..........................)).)))

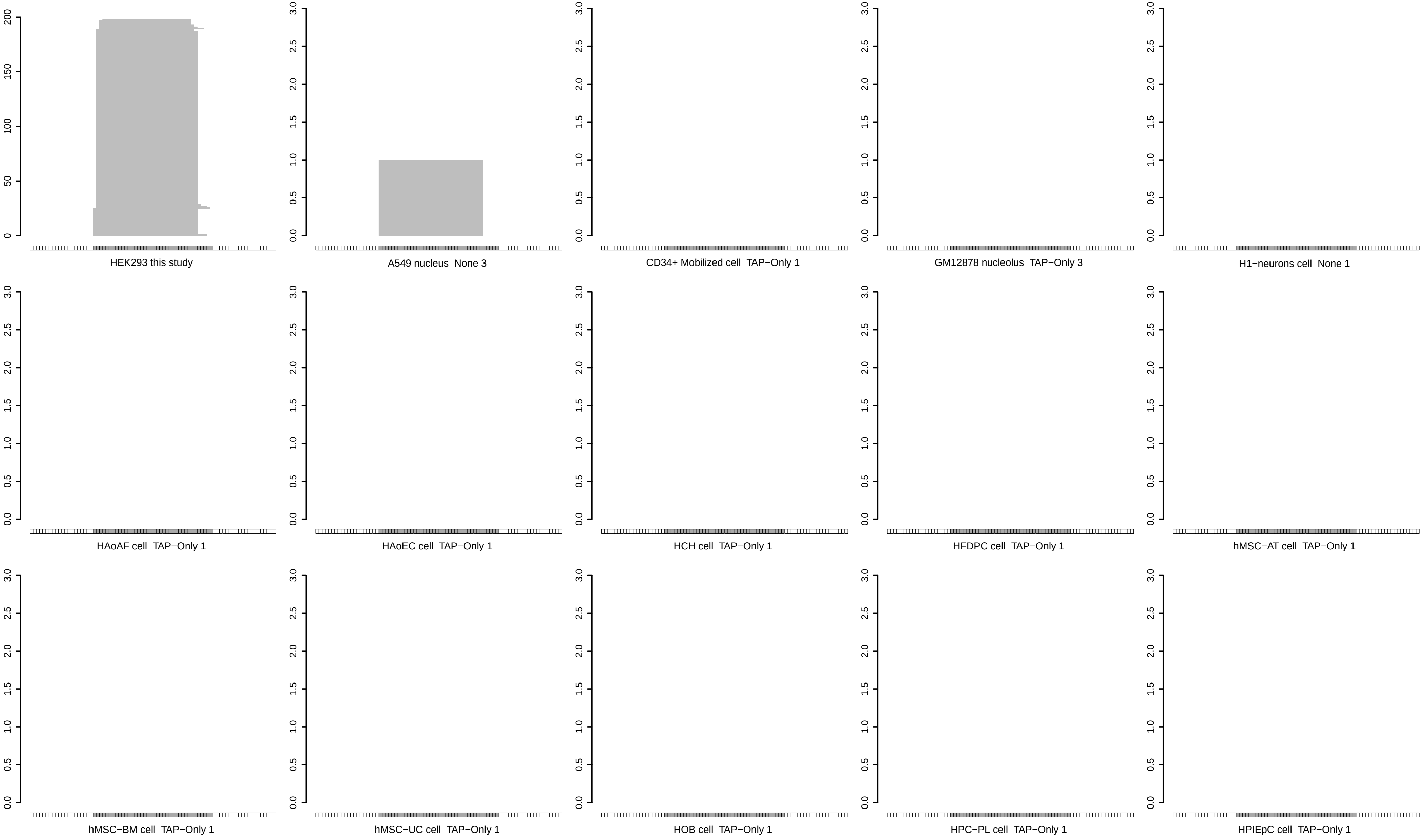

ZL50 chr2:101647748-101647801 (-)

AAGGCAGTGATGGCTCTGTGCCTTCTGGCCCAGGTGTACCCTTTCCTGAGCCTT

(((((..)))))

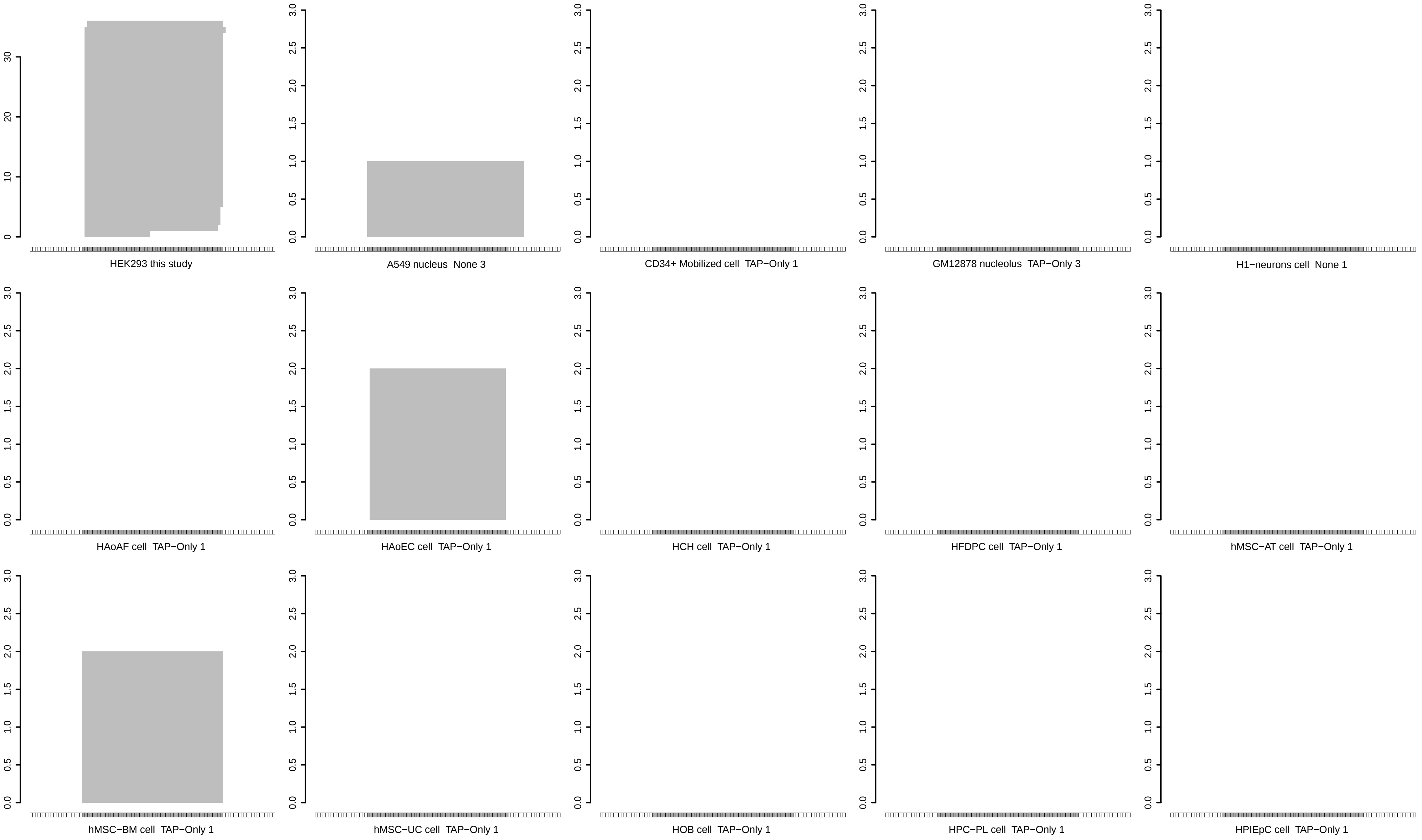

ZL51 chr2:29147423-29147485 (+)

AGAAGCCATTGAGGACACAATTTTGCTGCTGGAACTACACTCCAGAGGCTTGCTGAGGCTTTT

(((((((...)))))))

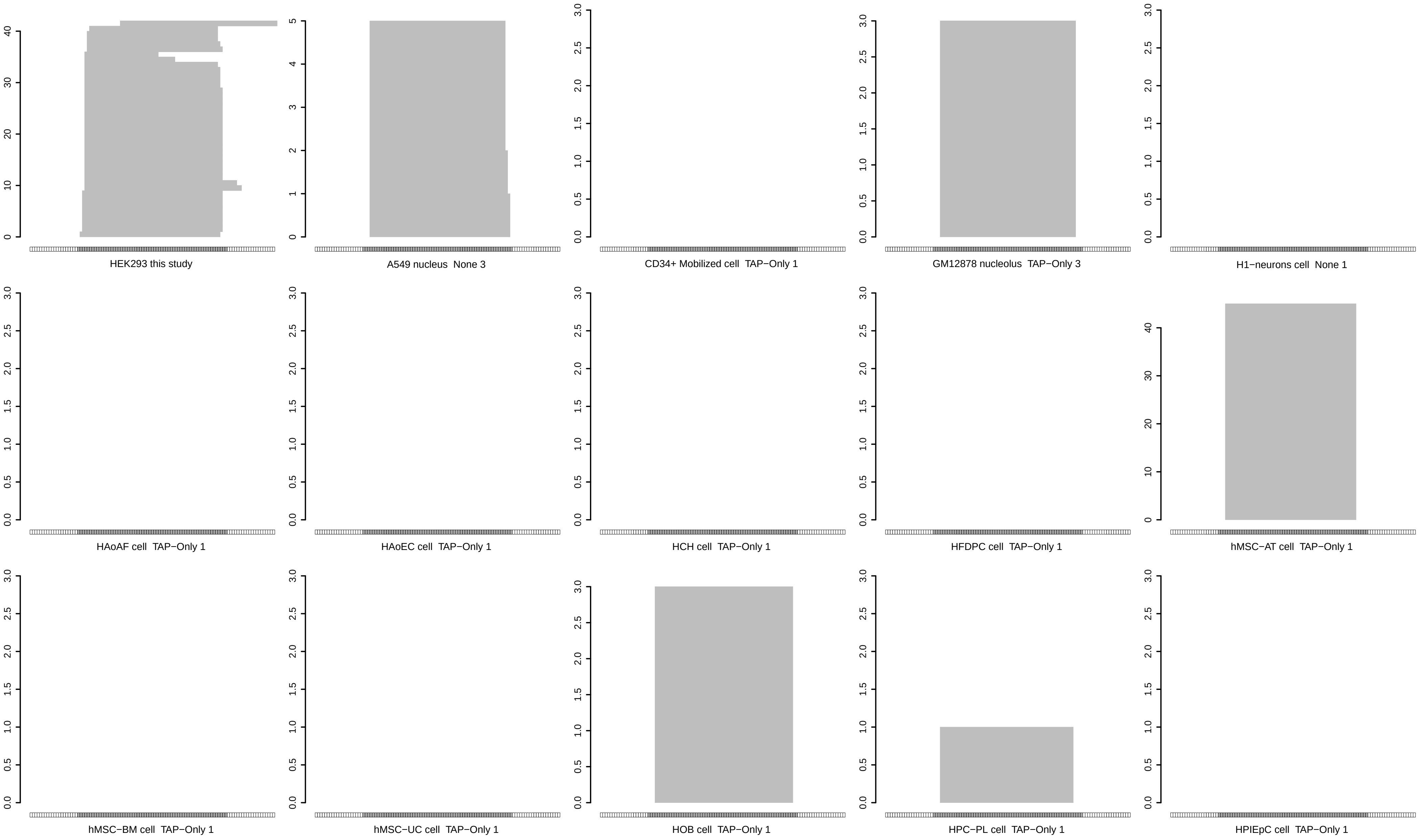

ZL52 chr19:55913957-55914028 (-)

TGGGTGCAGTGATGAGAATTAGGGTGGGAACCTGTGTCCTGGTCACCATCAGTGATATCTGCTGAGCATCTG

(((((((..)))))))

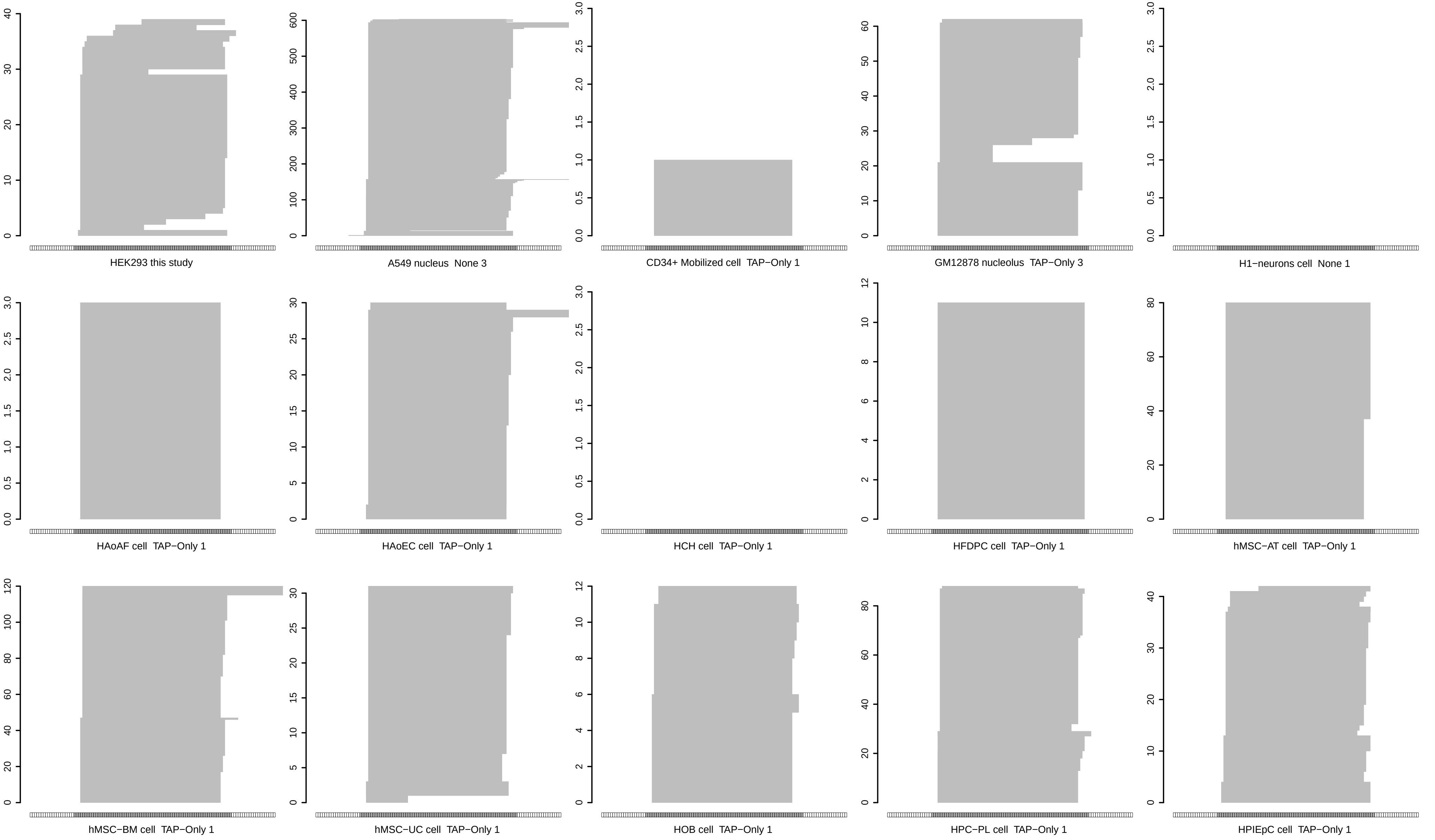

ZL53 chr2:241757267-241757374 (-)

GCCCAGGGATGATGCTTGGCTGGATCAGGATTAAAACTGGGGTCACTGCCCGGCCGCCCAGCCTGTGCCCCGCAGTCCTCACTGATGTCATGACTGCTGACCTTGGGC

((((((((..))))))))

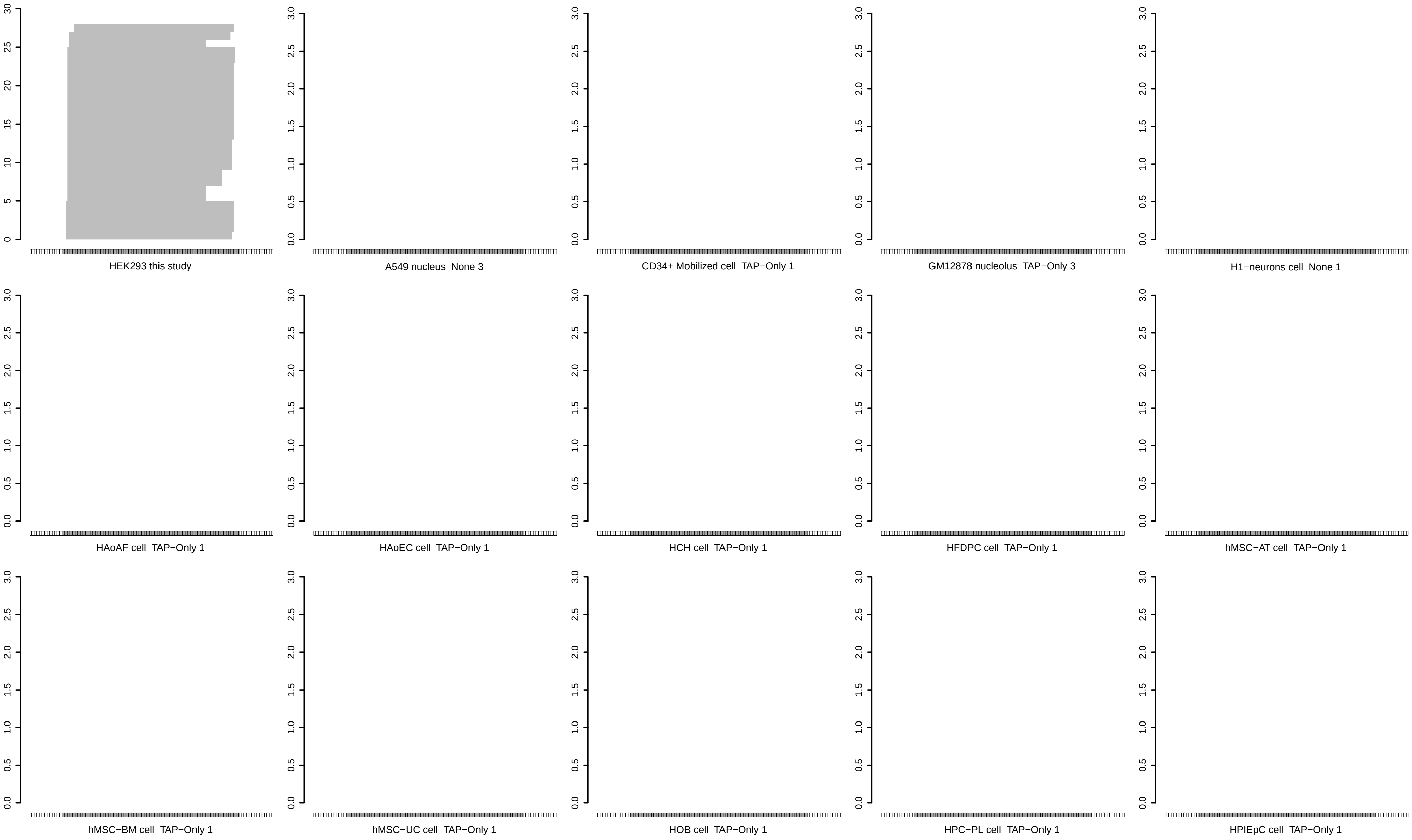

ZL54 chr20:3293135-3293241 (-)

TGGGACTGTGATGAGTTTGAAATTTATCTGTTCCCATCCCCTGAAGGTACTTATCTTCAGCTCCTCAGGATGCATTATCTTTATTGATCTTCATATCCTGAGTCCCA

(((((((...)))))))

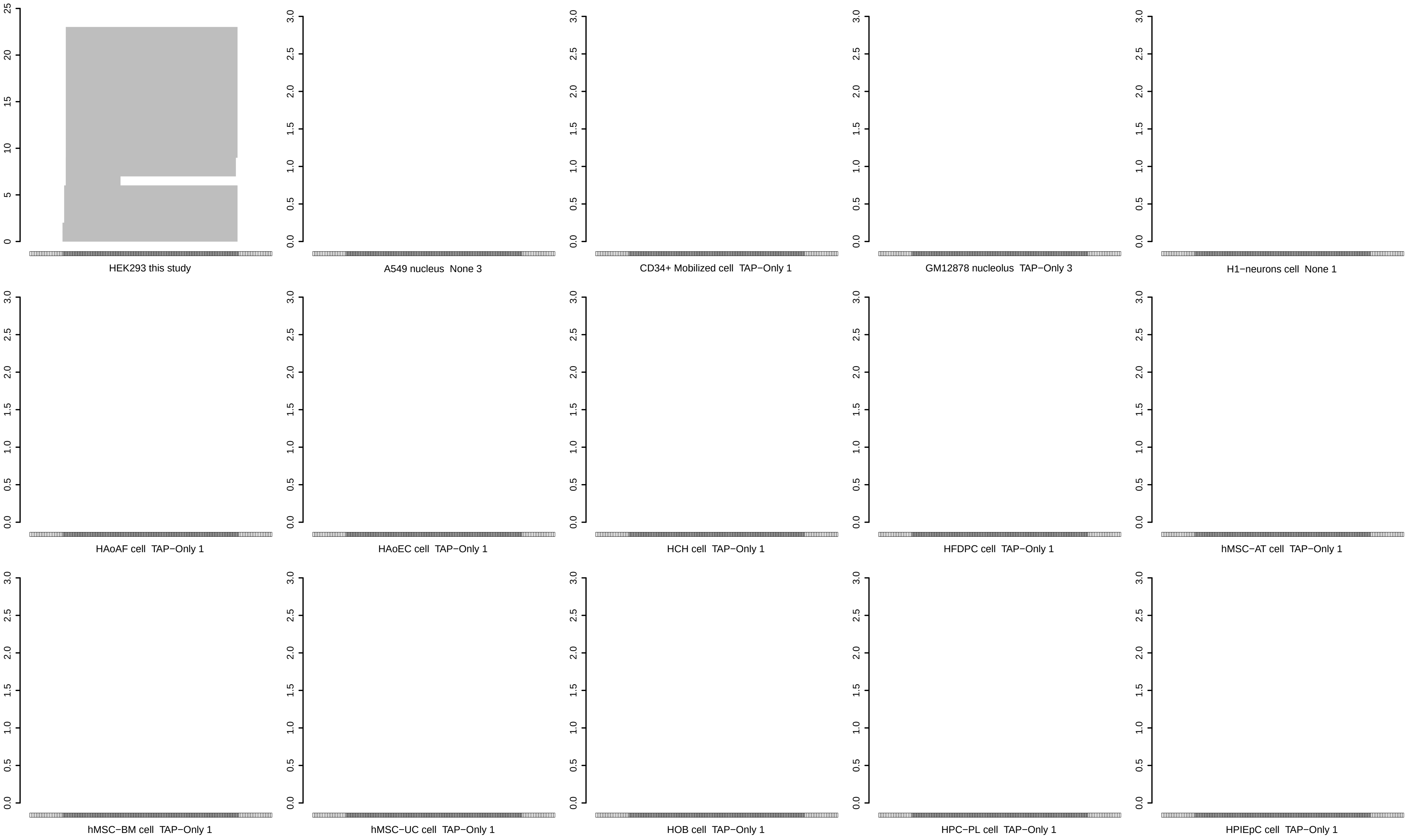

ZL56 chr13:61036311-61036429 (+)

ATTCCTATGACGAATTCAGTCACACCCCTTGCAAGGGAGCTCACAGATGACTGAGAAGAAAGACTTGCCATGTAATGGCTCATTCTCTGATTTTATGGCTGTATATTTCTCTGAGGAAT
((((((...))))))

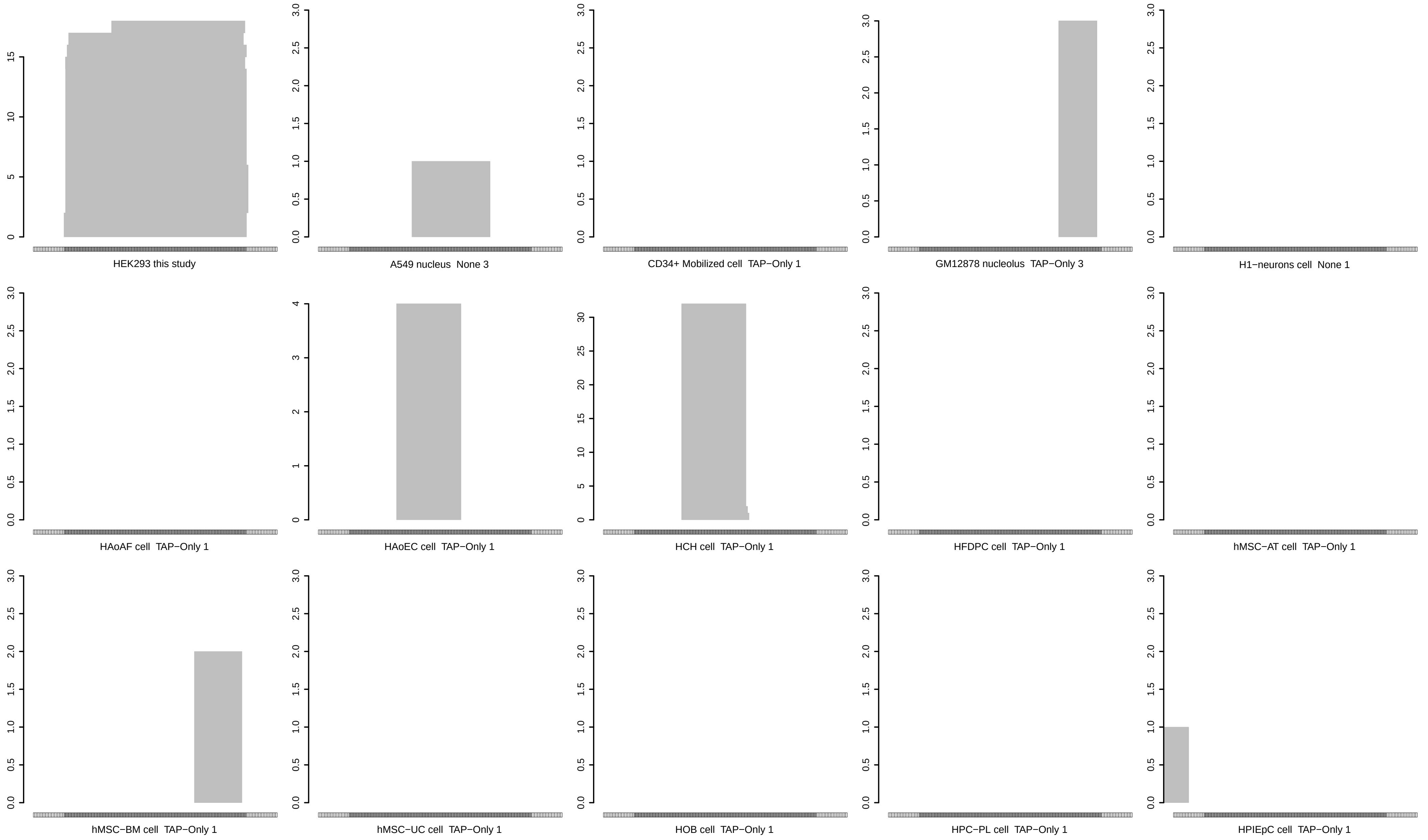

ZL60 chr17:25932218-25932298 (+)

TTAGCATGATGAAGGGCCTCAGGTCACCGGGGCCAGCCGCCACATATTGTGGATGAGGACATTGAGGTCCACTGATGCTAA

((((((...))))))

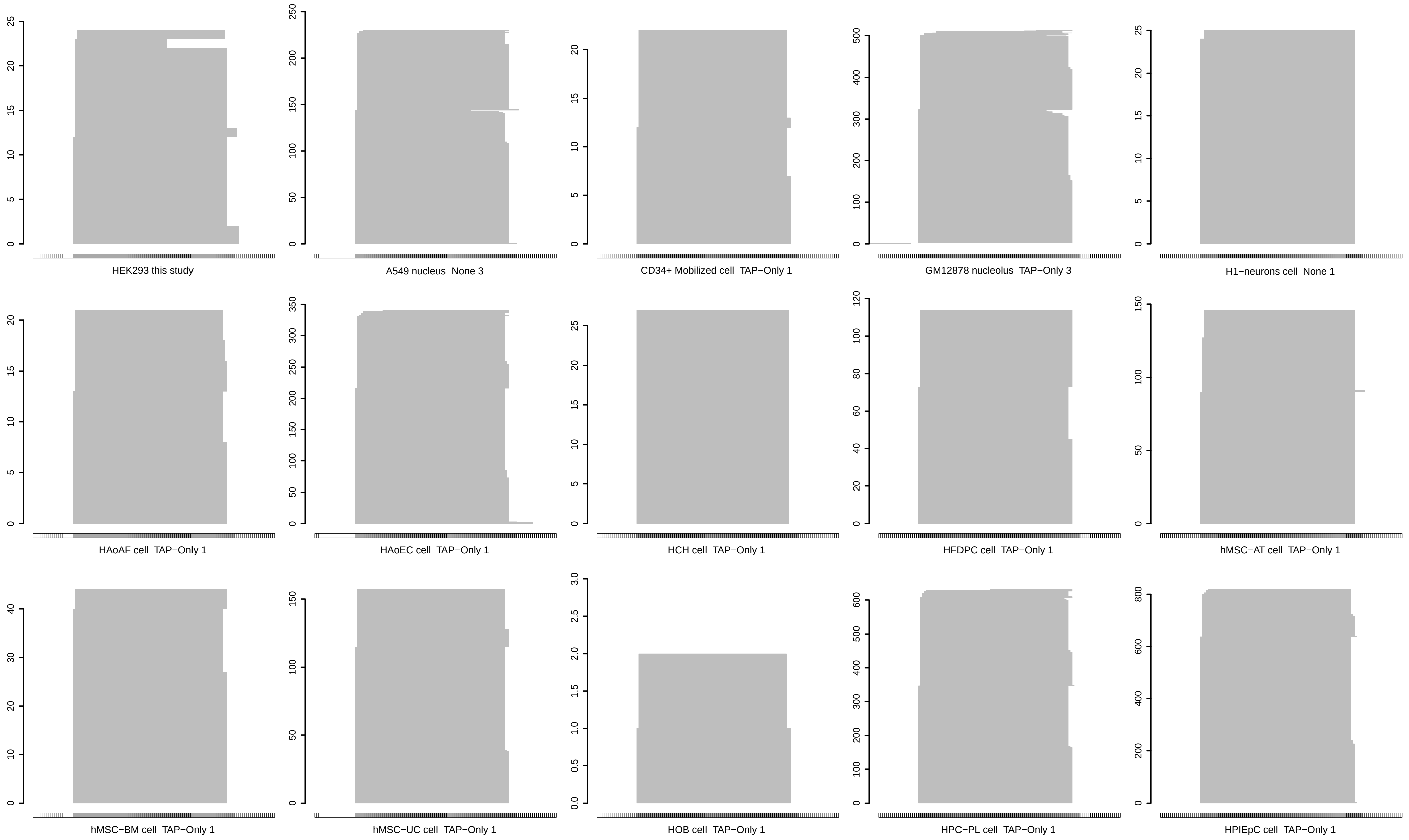

ZL62 chr1:222831739-222831801 (-)

TGTTGGGTTGAAATGATGCTCACTTTTCTTCAATGTAGAAAGCCAGGACTGATCAACCTAACA
(((((((((((...)))))))))))

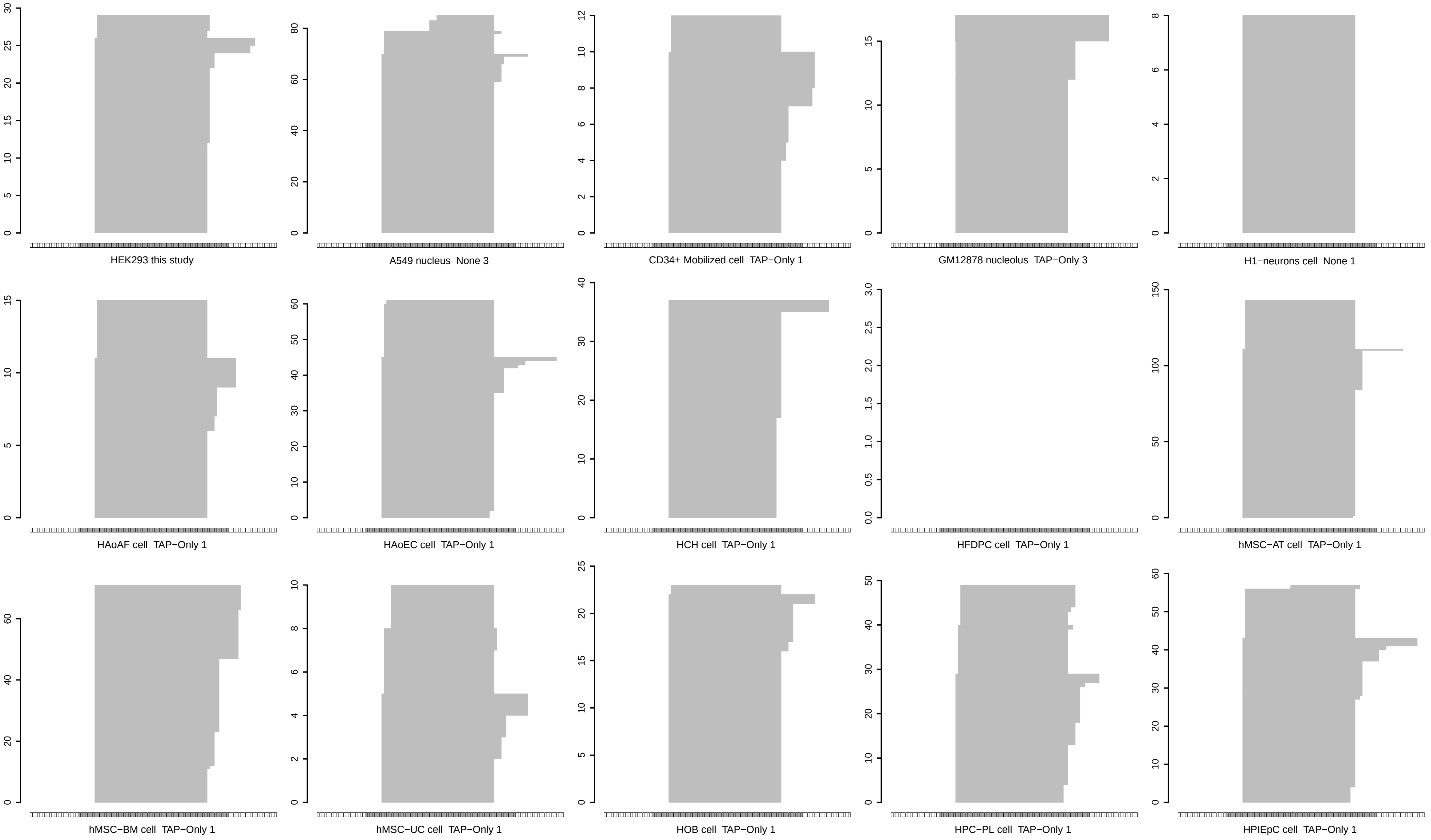

ZL63 chr8:104321264-104321309 (+)

AGCTGAAGAGCTGATGATGTTCCCCTAAAGCTGAAGCTCTTAAGCT

((((.((((((.......................)))))).))))

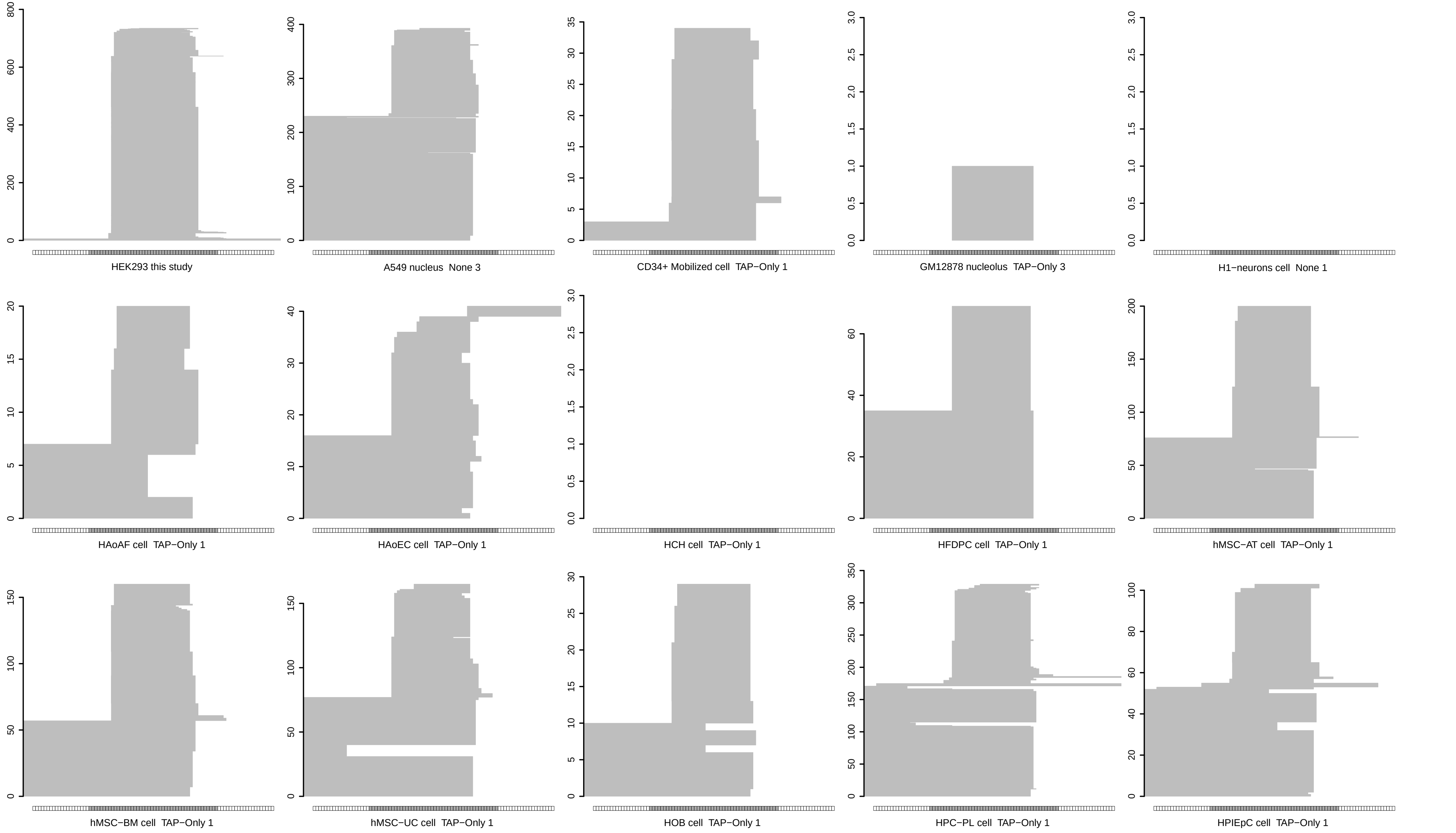

ZL64 chr13:48952644-48952705 (+)

AACTCCTATGATGATGATGCTATCTTCTGGAATATCTCCTGAAGGAGCTGCCTGAGGGTGTT

(((((((...)))).)))

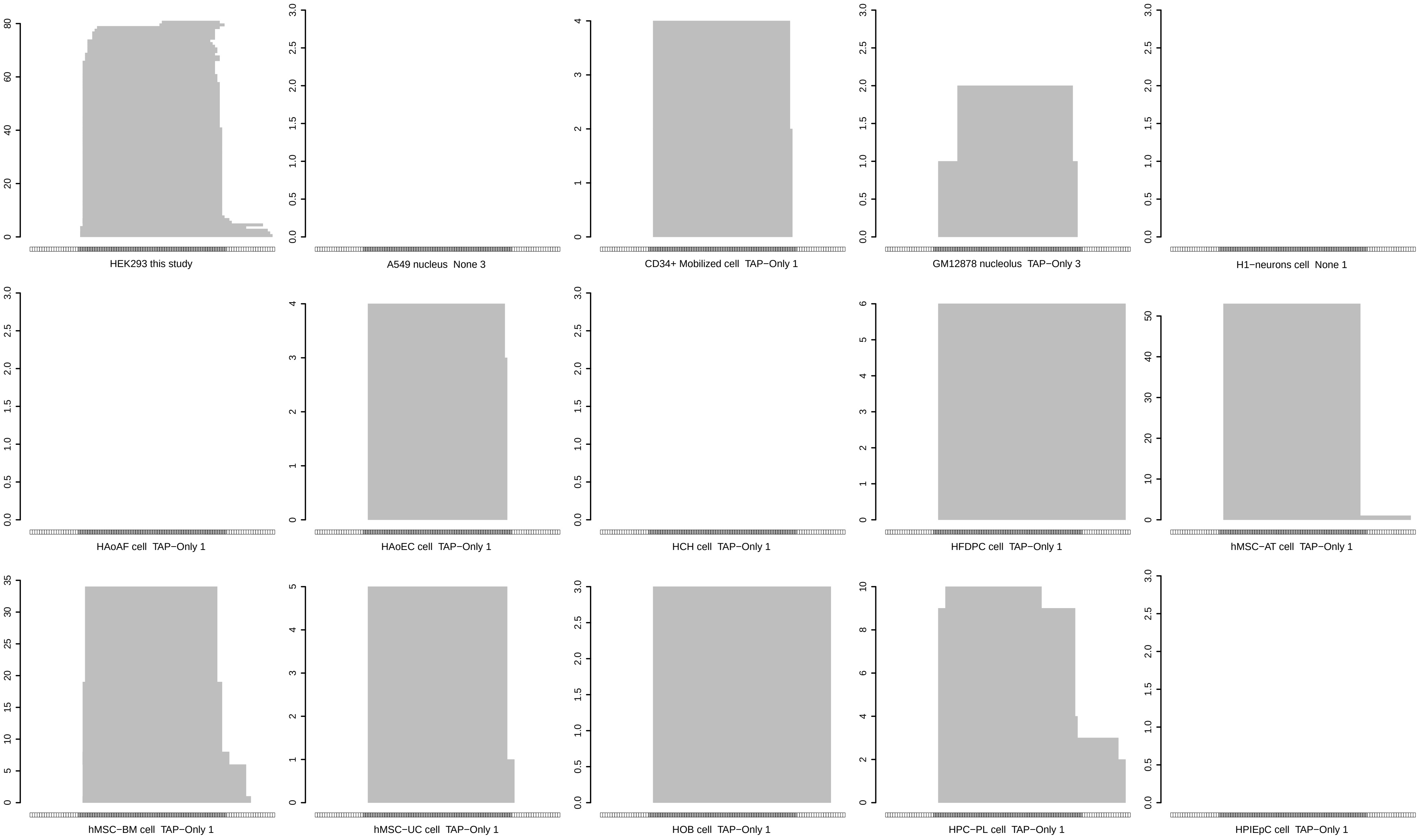

ZL65 chr19:5587445-5587547 (-)

AATGTGCGATGATGATTTTTCTCACCTGGAAAAAAAACCCAAGCGCTCTAAAGGCTGCGGGACCGTTACTGAACCCATGTTACGATGAAAACGCTGAGCACTT

((.((((..))))))

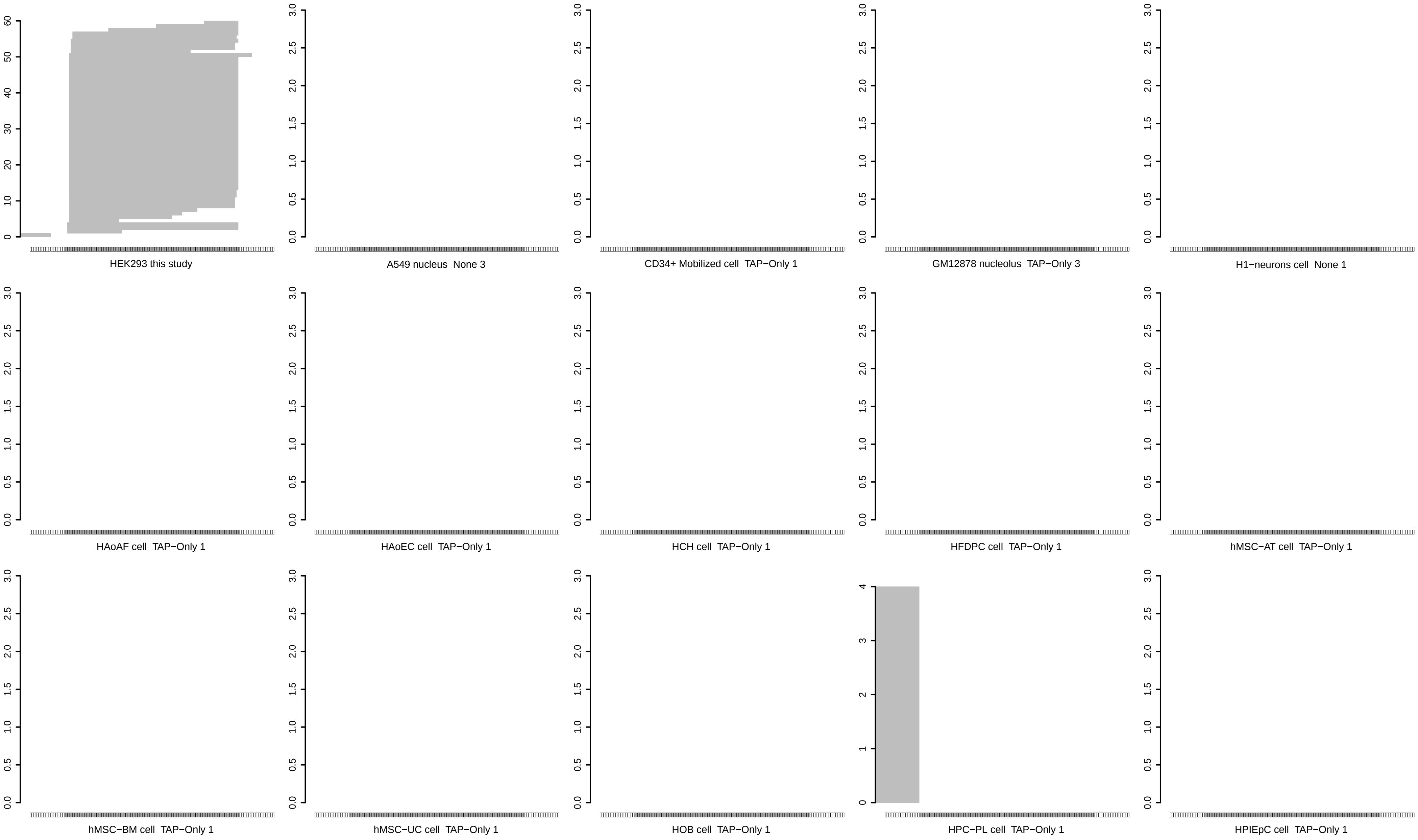

ZL68 chr10:3176296-3176490 (+)

TCCCCTGCAATGATGATGAAATAGTAGGCATATGTGTGATCTTCTTAAAGAATCCACAACGGTTATCAGATTAAAGCTGTGACCCATCCCCAGCTCAGATGAGTTCATGAAATCCTGATTTAAGATTTTAAAATAATGAAGATTGTTAGTTGACTTGTTCGGTTTATTAAAATCCTCATCTTACTGAGCGGGGGG

((((((((...))))))))

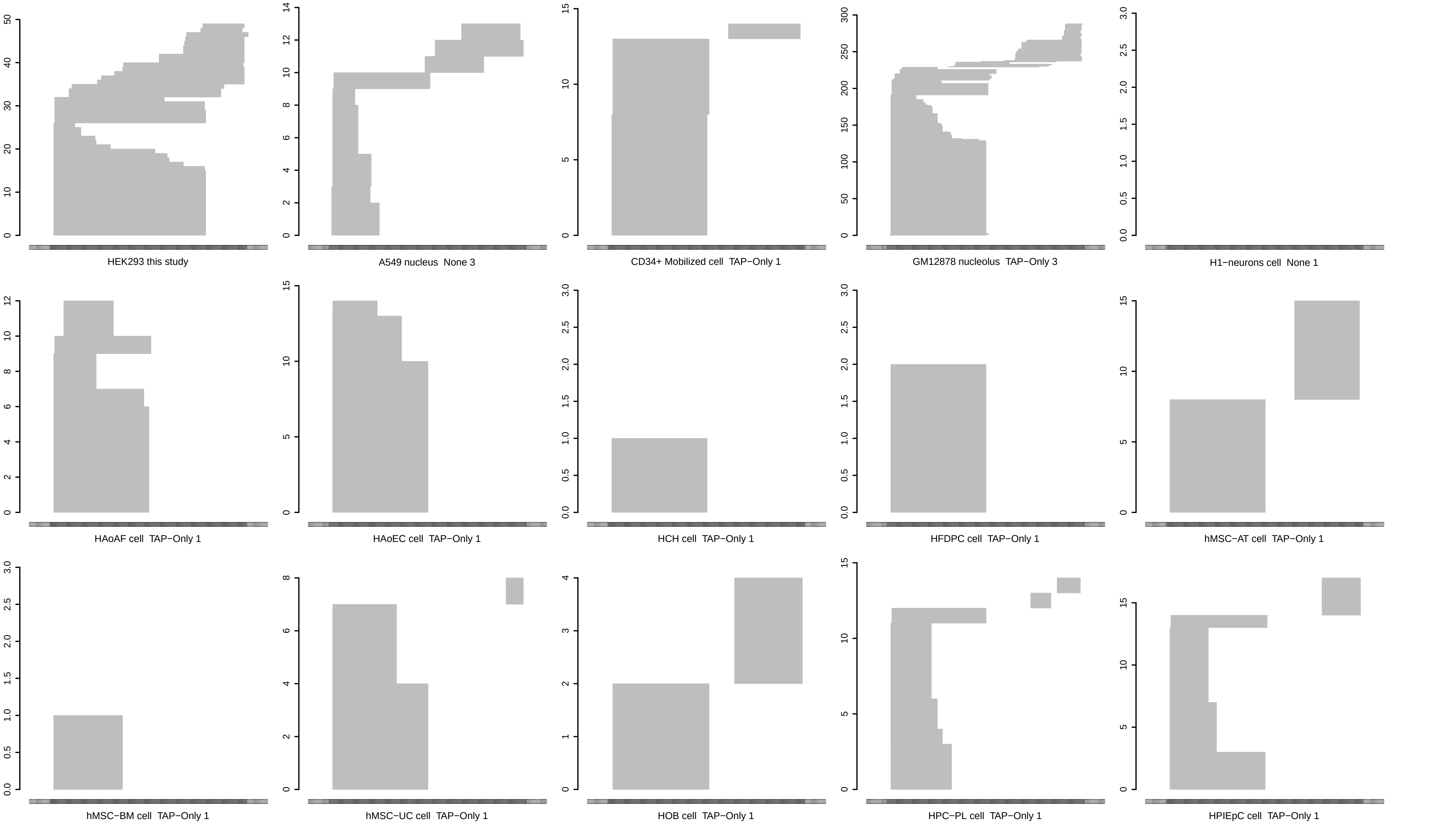

ZL69 chr15:89846546-89846578 (+)

GTCTGGTGATGCAGATGCTTGCTATCTGAAGAC

((((.........................))))

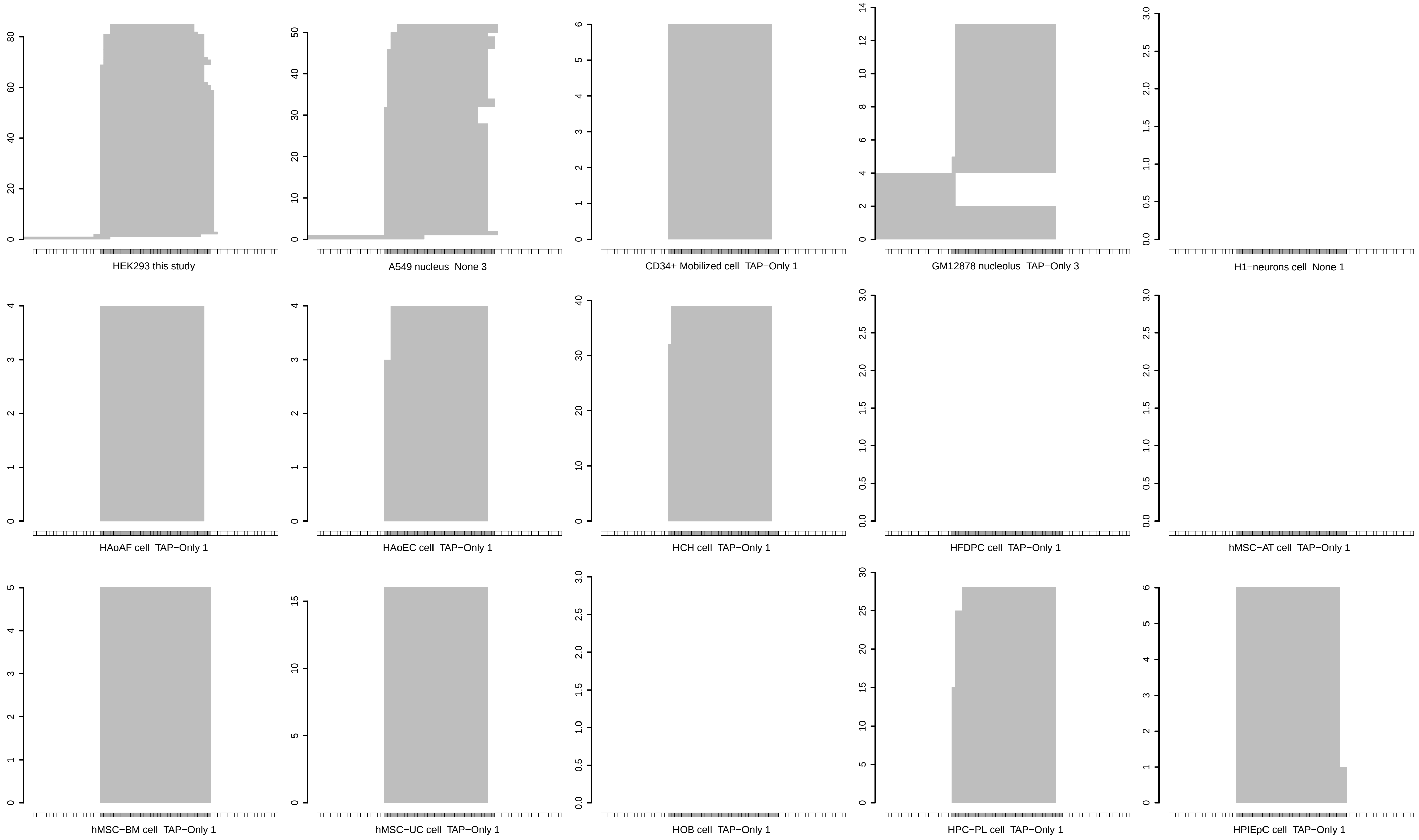

ZL71 chr1:45136627-45136723 (-)

GGCCTGGGAGGGATGATGTACCAAGTGAGCTGAATGGAATCACTTGGCAGCACTGTAGGCCCAAGCCAGAGCTGCTCTGACATCCCAGGCCCAGGCC

((((((((.((((((...))))))...))))))))

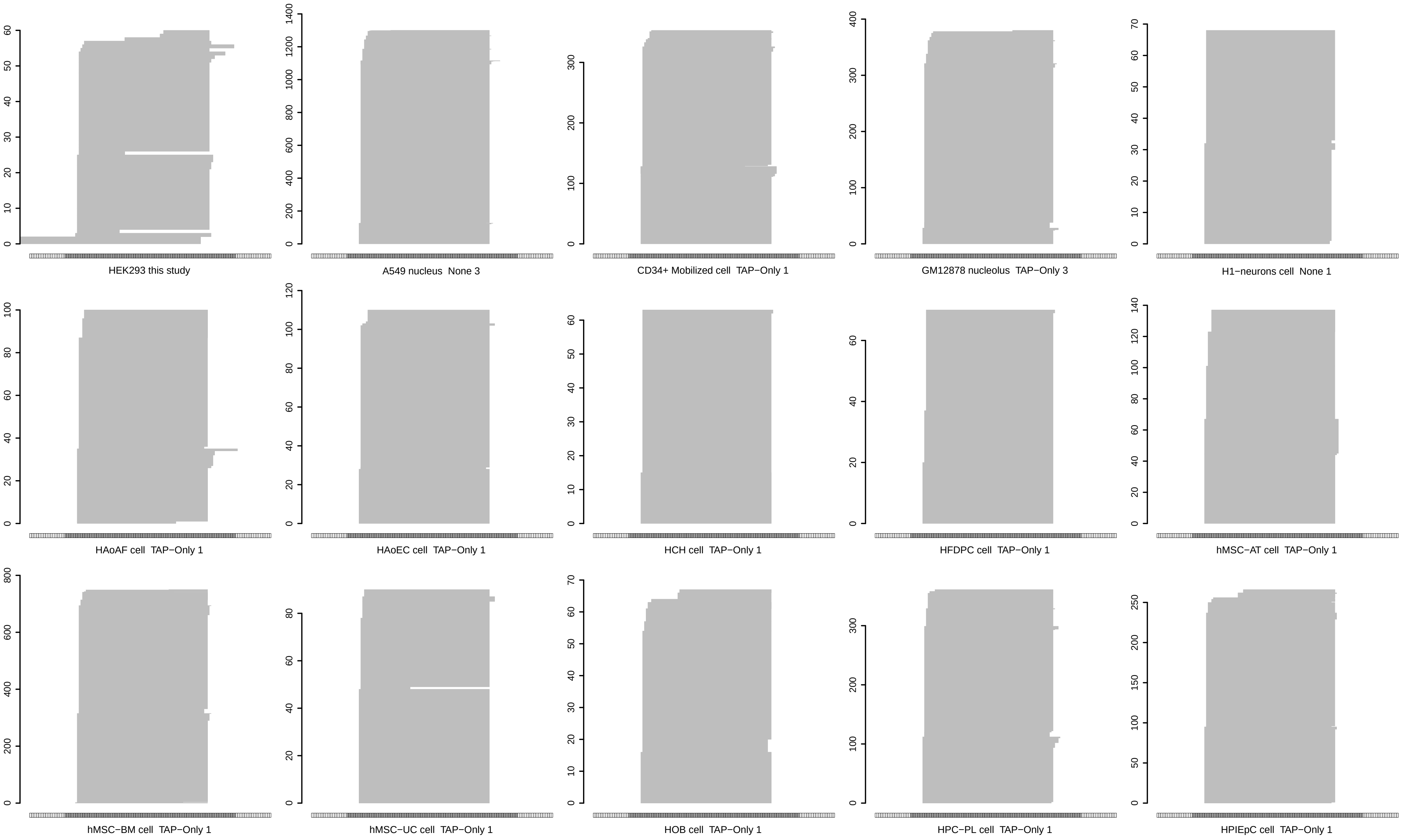

ZL72 chr8:118933088-118933160 (-)

GGAGTCCTGCTTGATGATGTGTTGGAGGAGCAAACTGGTGGCACCCAAGGCTCTGTTCTGAAGCAGCCACTCC

(((((.((((((..))))))..)))))

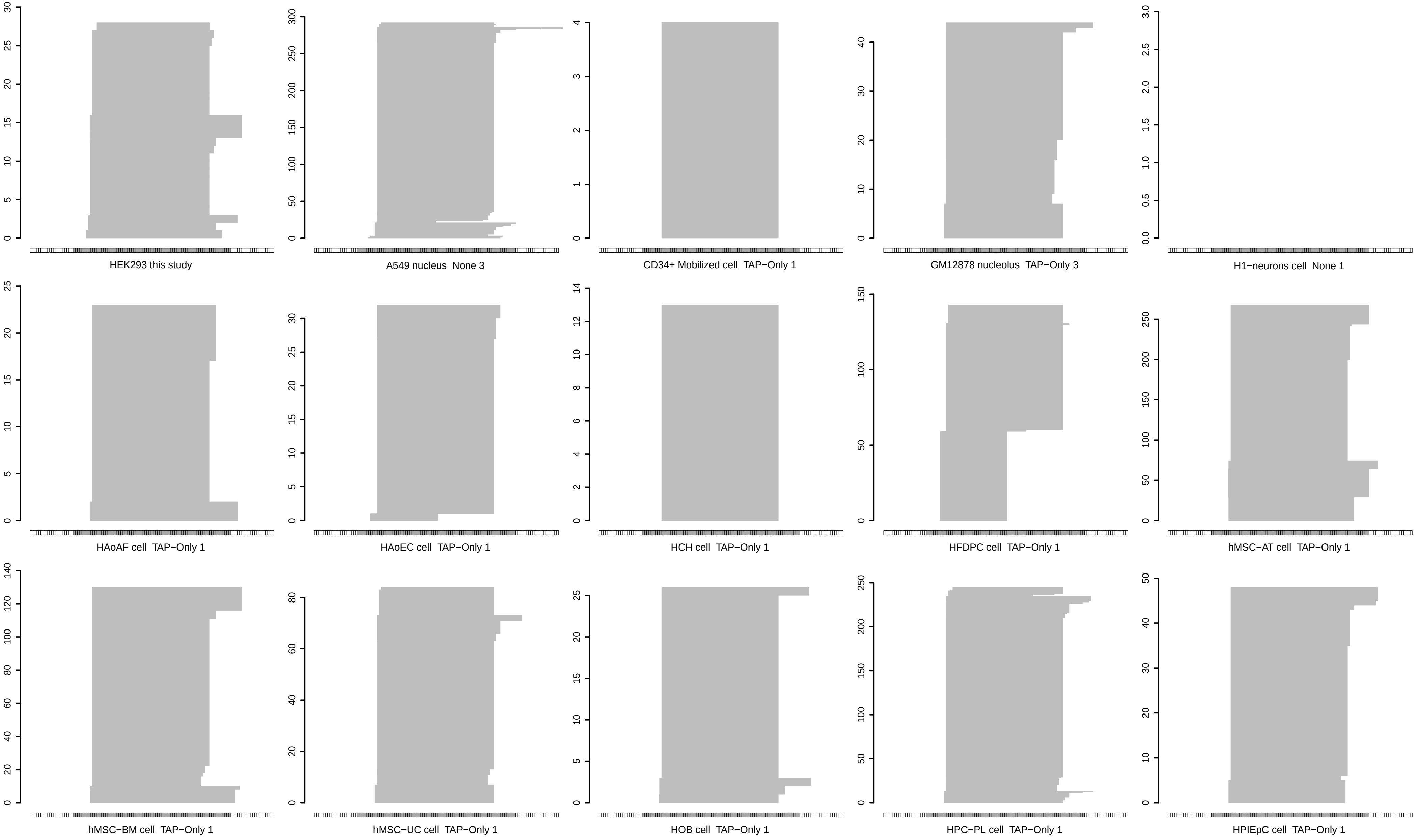

ZL73 chr6:609183-609252 (-)

AGGCTGTGATGTGAAGCTAGTTTTGCCCTTCCGGCTTAAGTGAGTCCCGTGGGTGGAACGTTCTGAGCCT
(((((..)))))

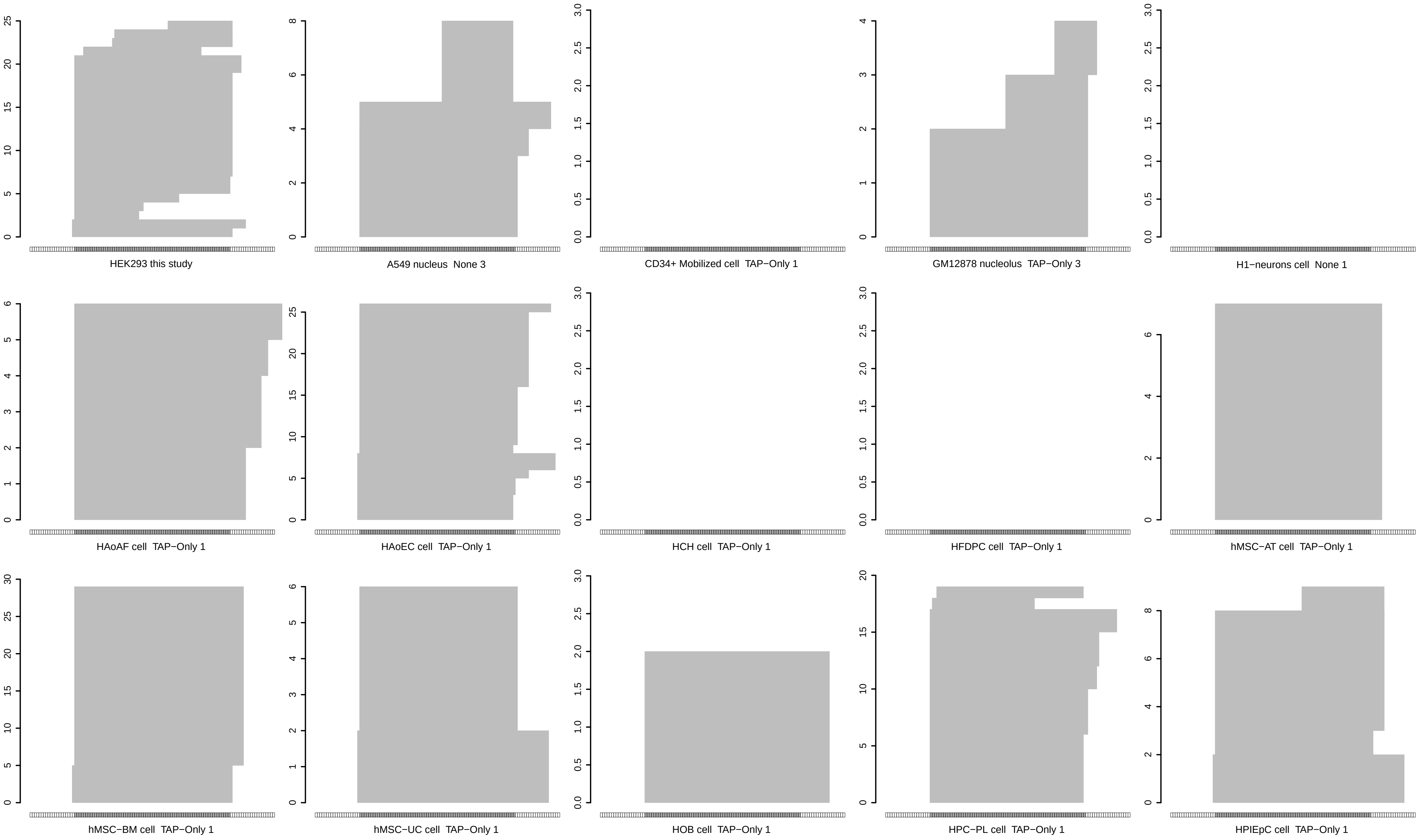

ZL75 chr7:42976788-42976827 (+)

TAGATGGTGTATGATGACCTCATGTAGCCTGAACATCCTA

(((..(((((.....................))))))))

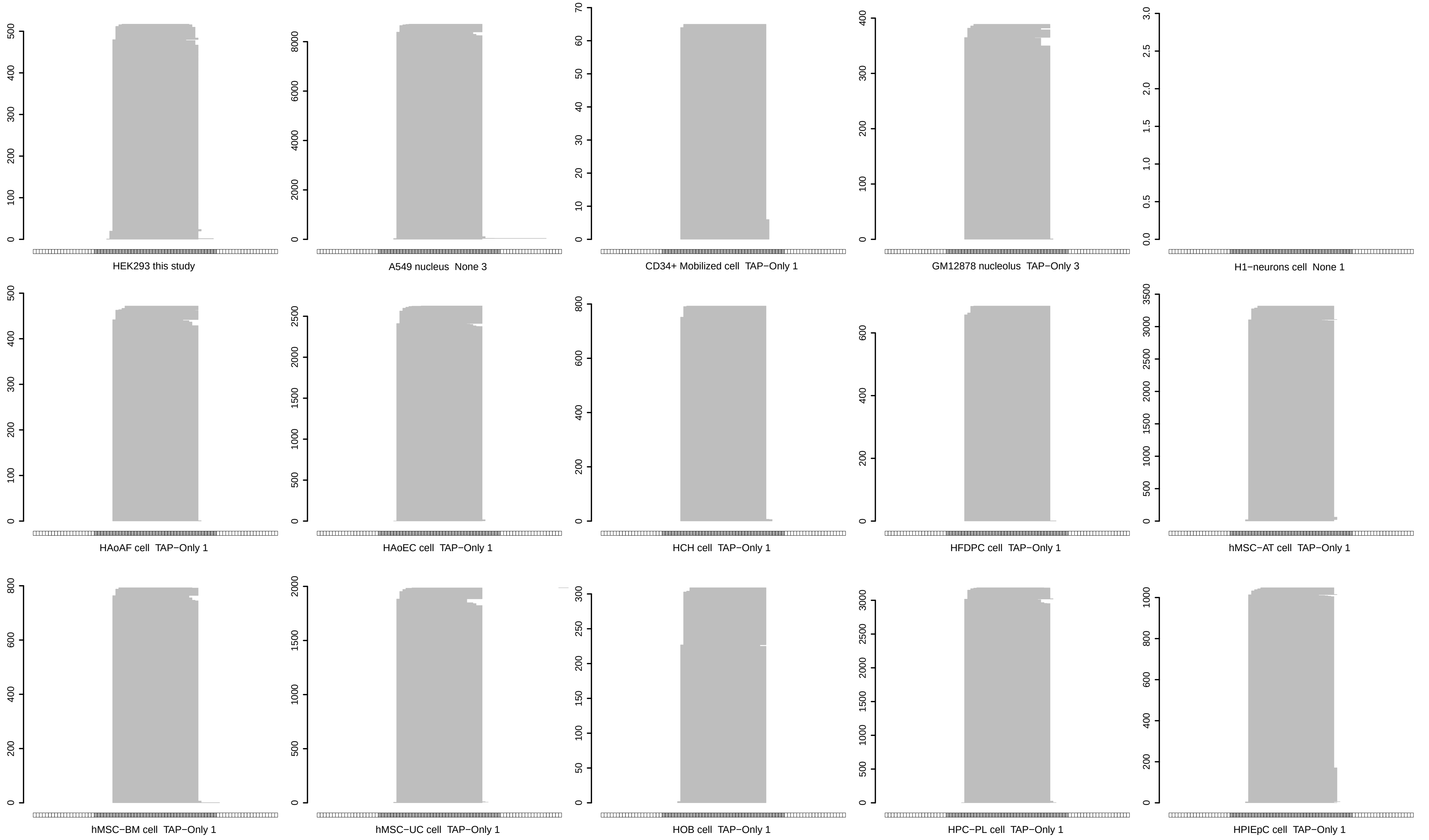

ZL76 chr9:131844912-131844939 (+)

CAGGCTGTGATGGACCTGGCTGAGCCTG

((((((................))))))

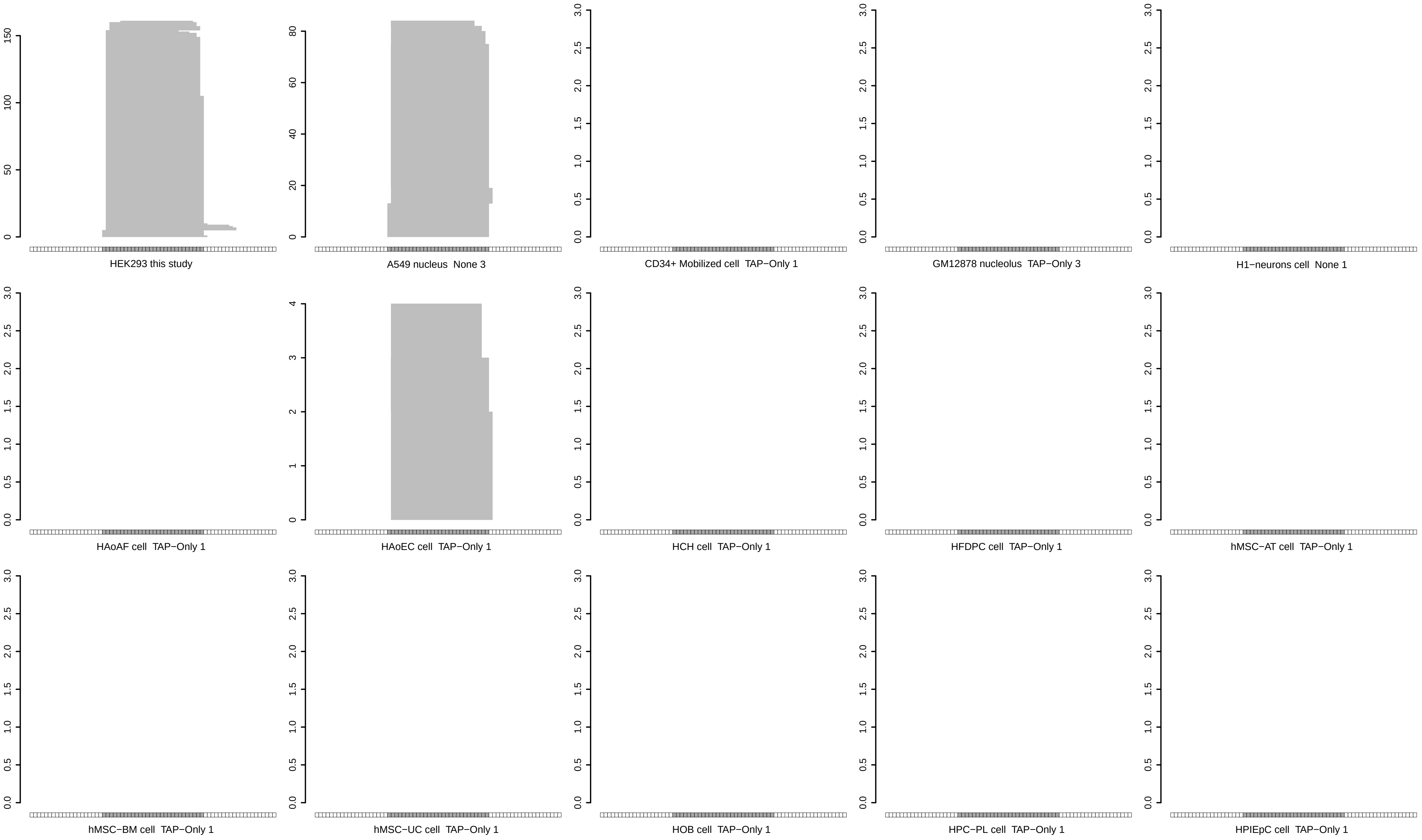

ZL77 chr10:70496931-70496957 (+)

CTGGTGATGAATATTATATCTGACCAG

(((((.................)))))

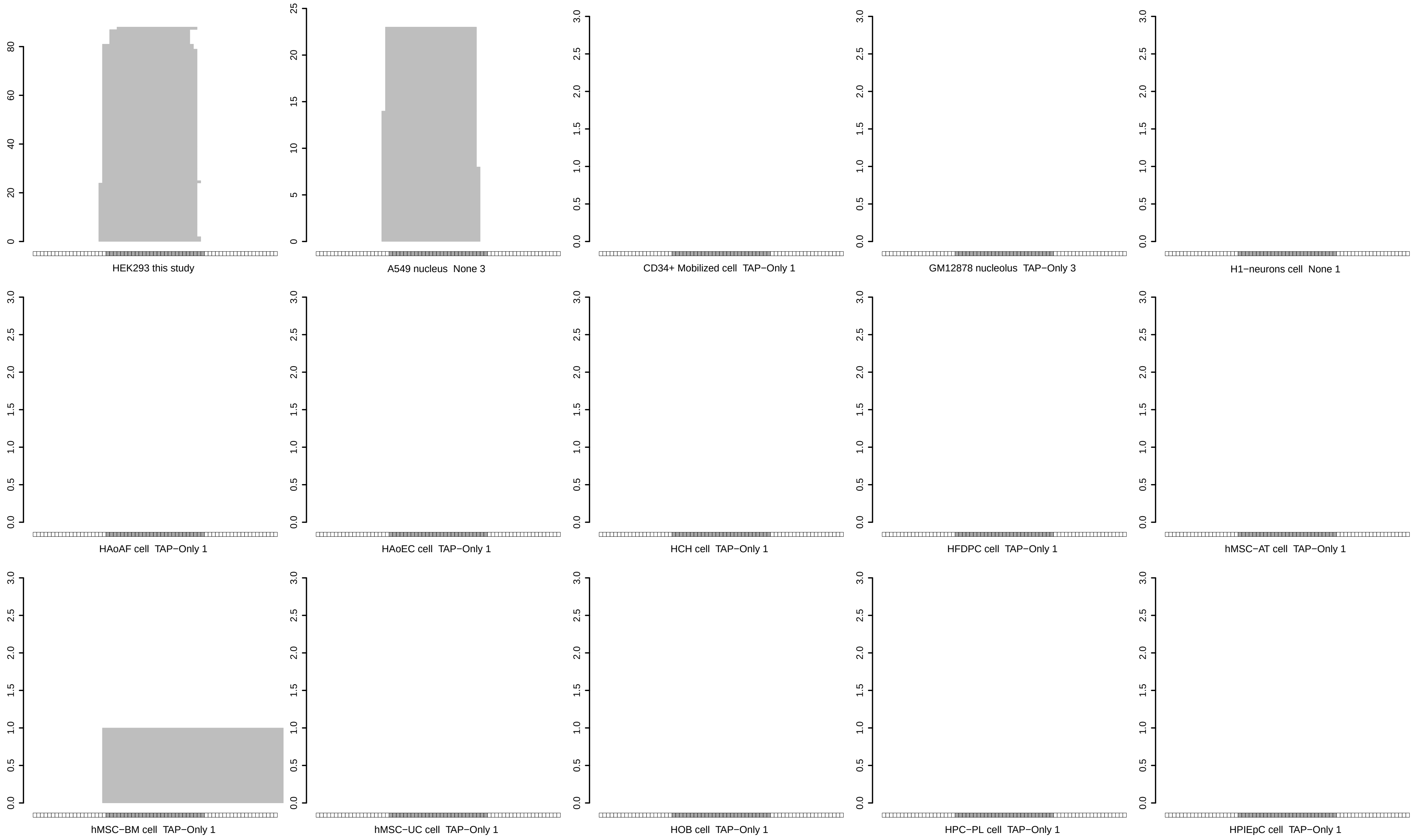

ZL78 chr16:72133249-72133286 (+)

GGACTGGGCTTTGATGAGGGTACACCTGAGCCCTGTTC

((((.(((((..................))))).))))

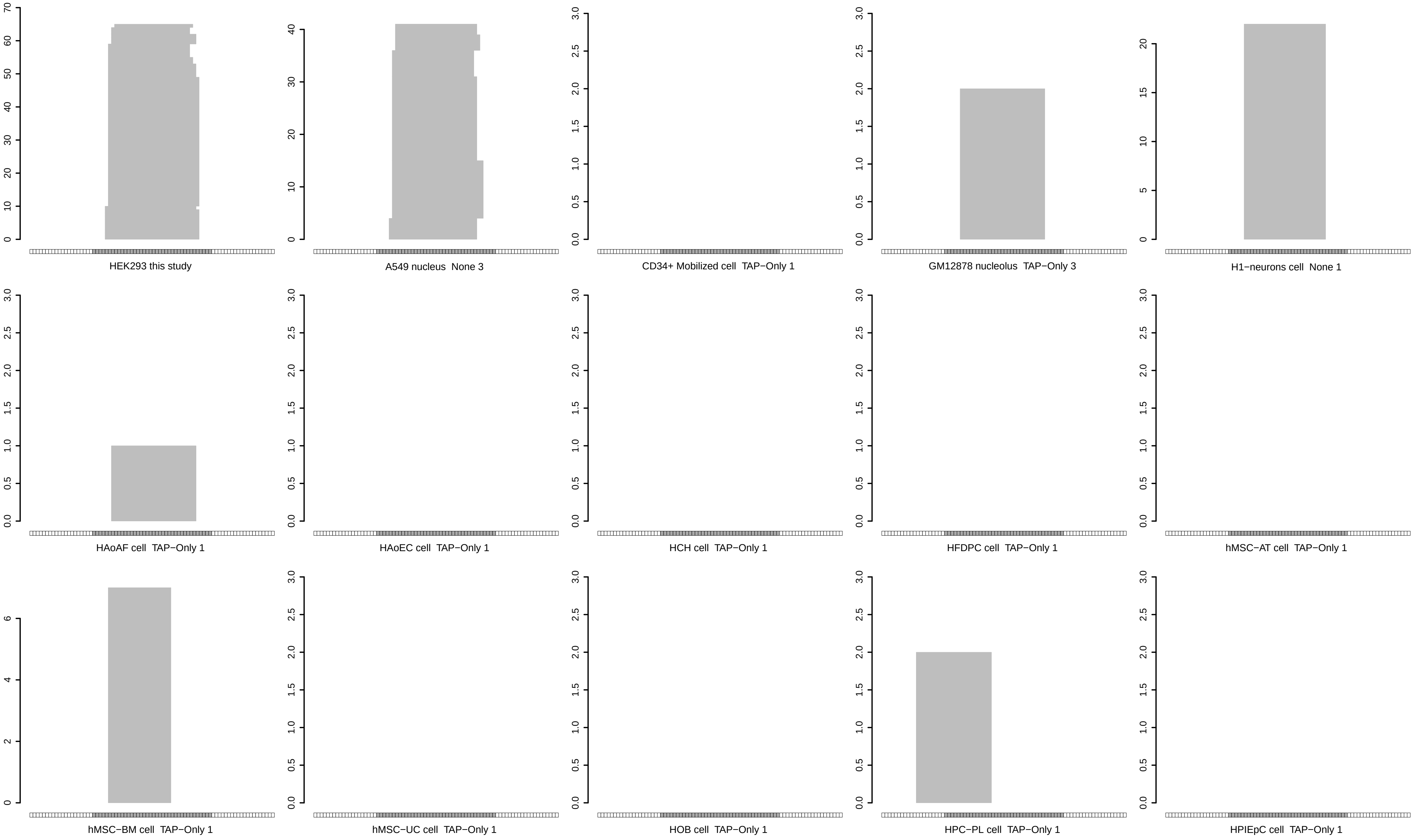

ZL79 chr3:14535495-14535531 (-)

TCTCTTTGATGAAACCTAACAGAAGGACTGAGAGAGA

(((((((.......................)))))))

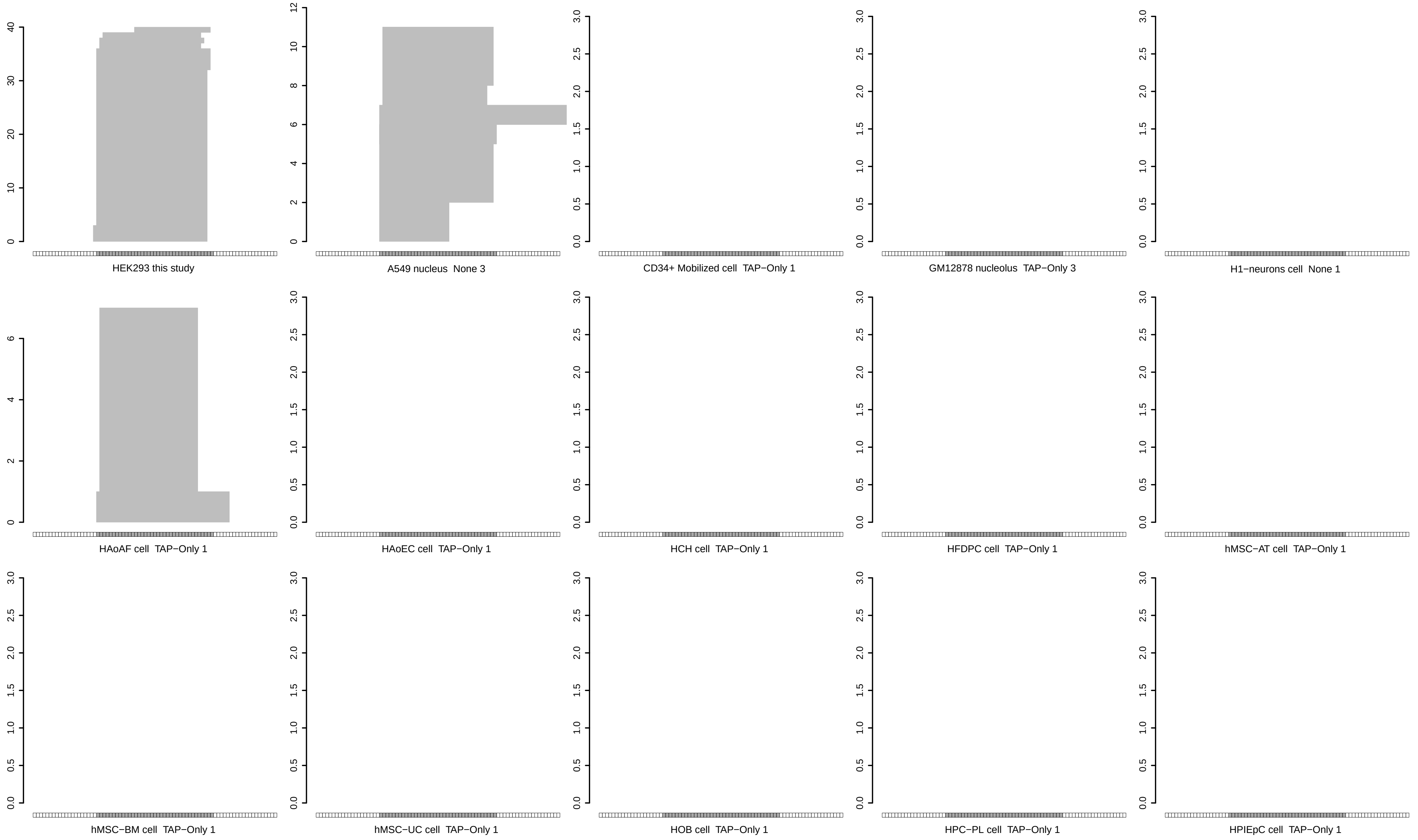

ZL81 chr4:2261982-2262014 (-)

CCCATCTCTGATGACGCCTGTTCCTGAGATGGG

(((((((...................)))))))

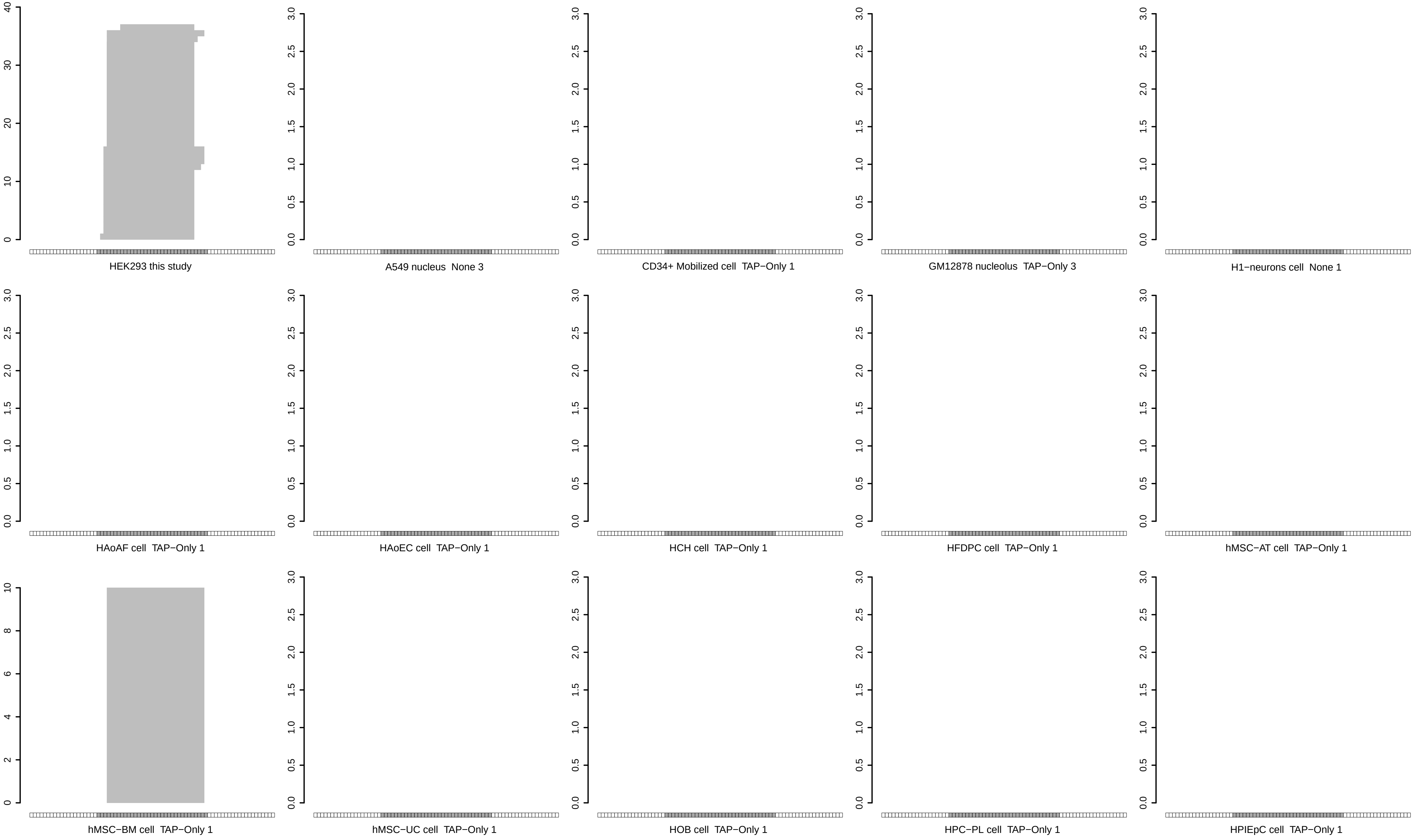

ZL84 chr6:47503420-47503453 (+)

AGCAGTTCTGATGATGATGAATTCTGAAACTGCT

(((((((....................)))))))

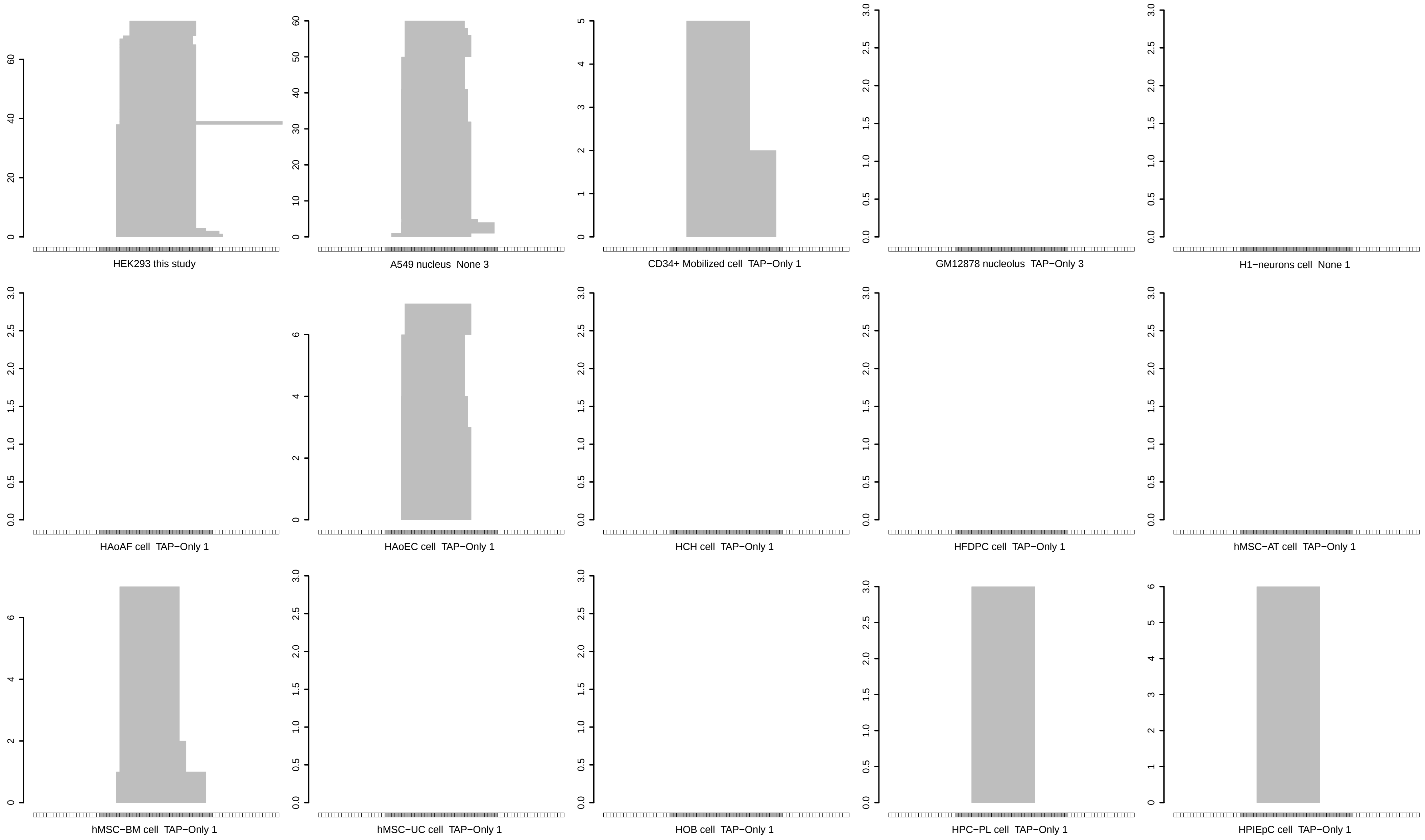

ZL89 chr13:50858040-50858119 (+)

AGGTTGAGGATGATGAATGGCAGTTTCCAAATGTATTTCTGAAAAAGATGAATAAGAAAGAGCTGTTTCTGATTCAACCT

((((((((..))))))))

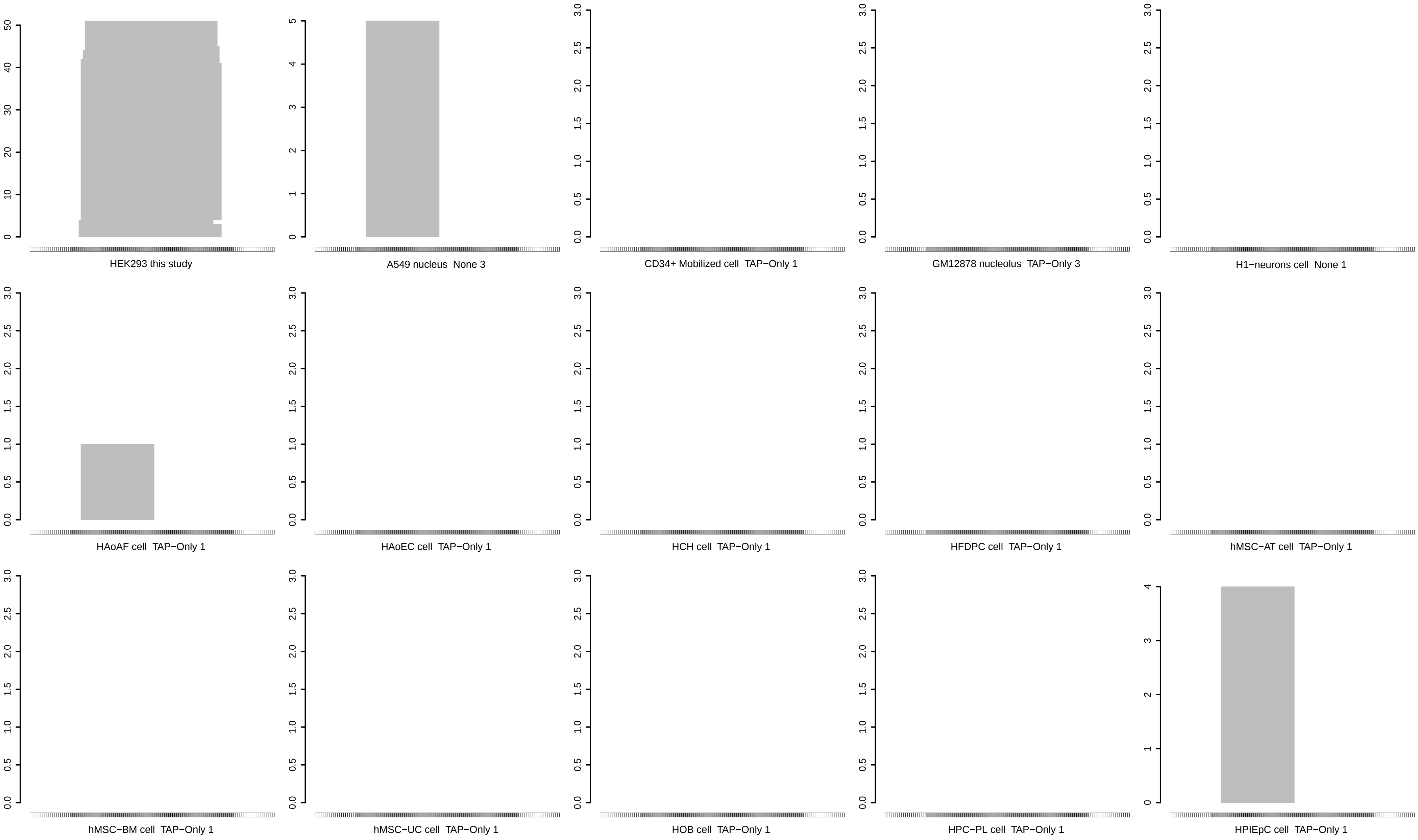

ZL92 chr19:36106857-36106899 (+)

GAACTGTGGATGATGTGTTGCGATTTTACCCTGACACTGGTTC

((((((((..........................))).)))))

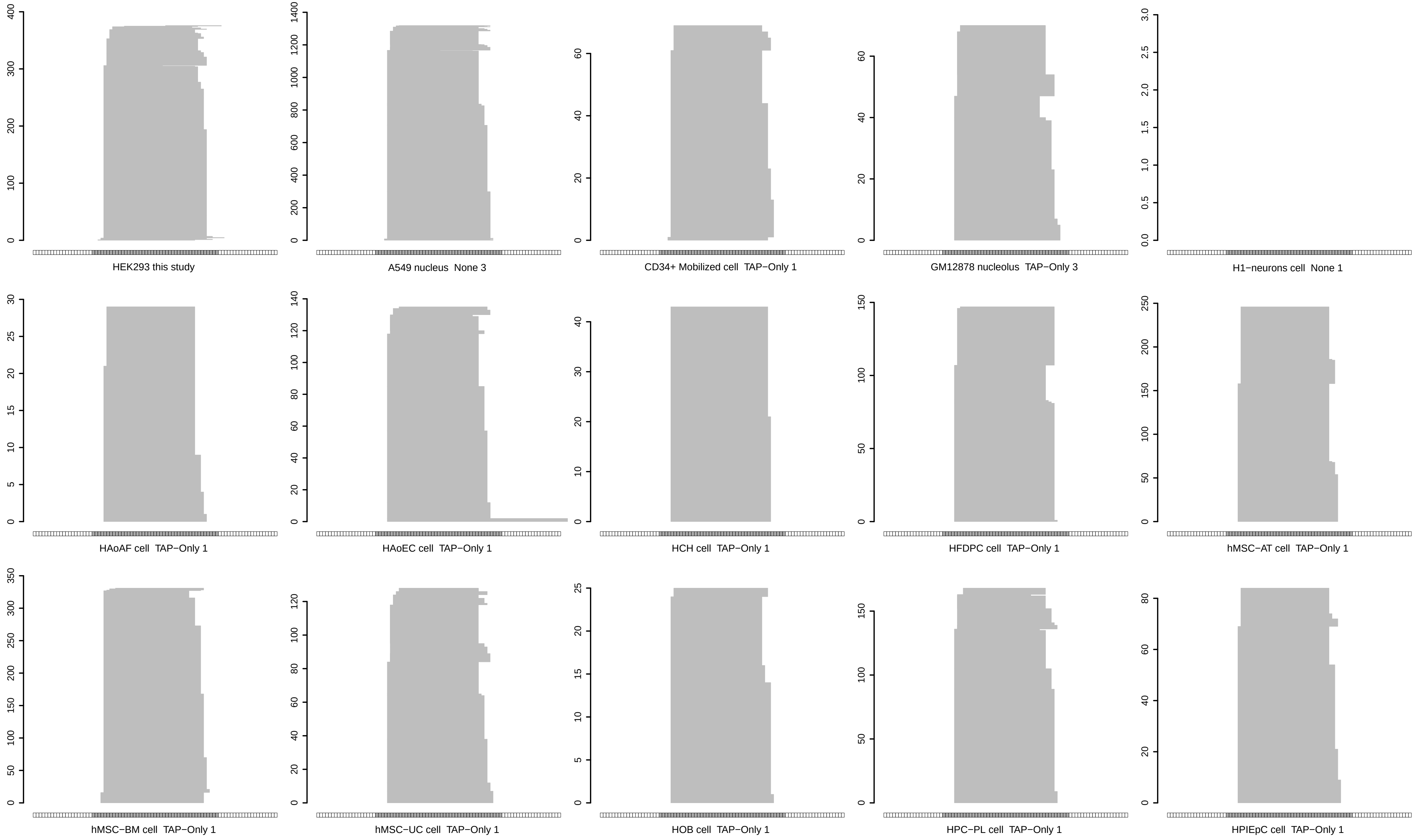

ZL98 chr1:1240102-1240198 (-)

AGGCTTCTCTGGTGATGTCATGCCTTAACTCTTCTCTCCCCAGGCAGCCCCACTGTCAGGCTGTCCTGGTGGGAGTGGGCTCTGAGGAGGATGGCCT

((((((((((...))))))..))))

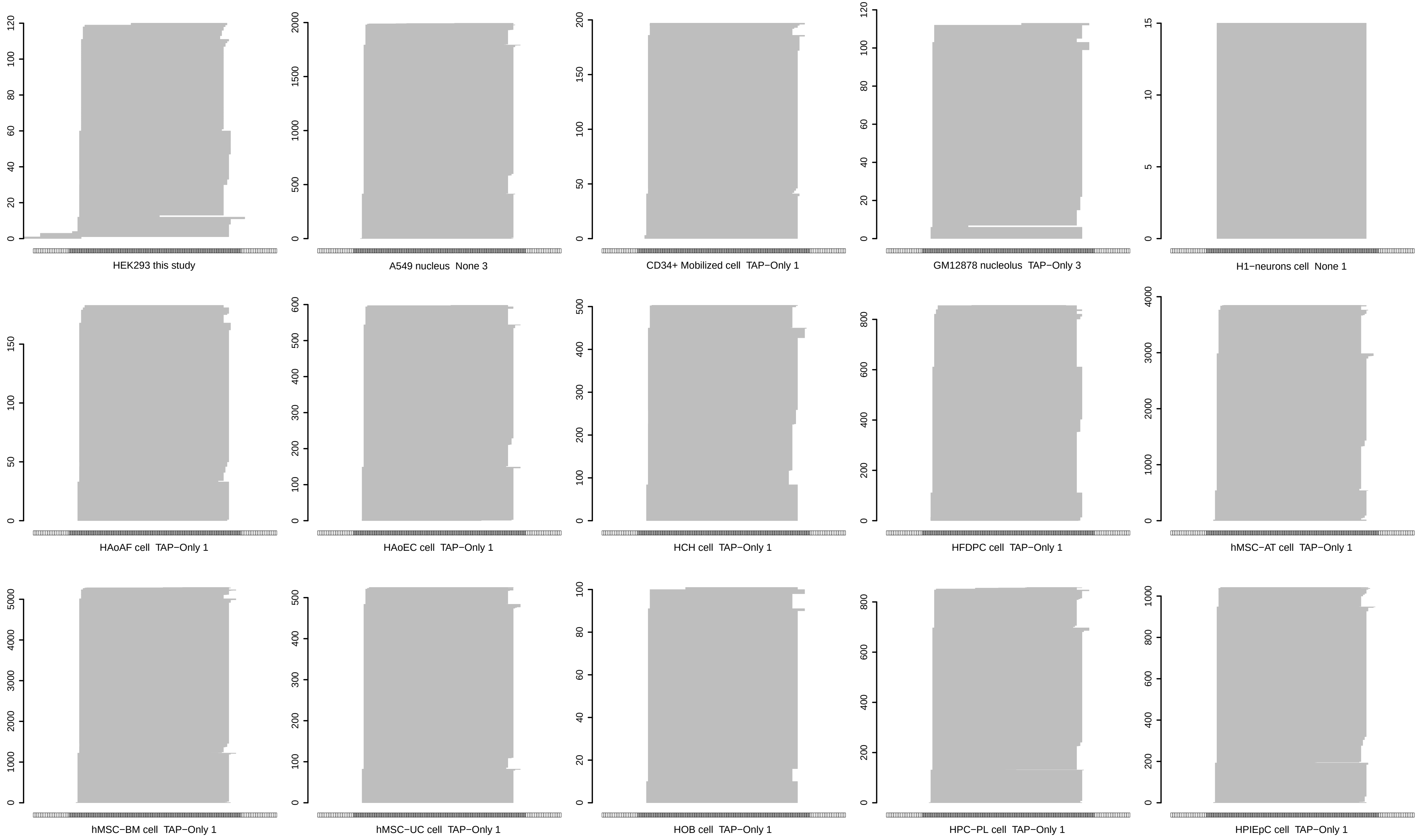

ZL99 chr17:15561137-15561187 (-)

GAGGAAGCCAATGATGTCCAATCAACACCTGATGAACCTGAGGCATTTTTT

((((((((((..............................))).))))))

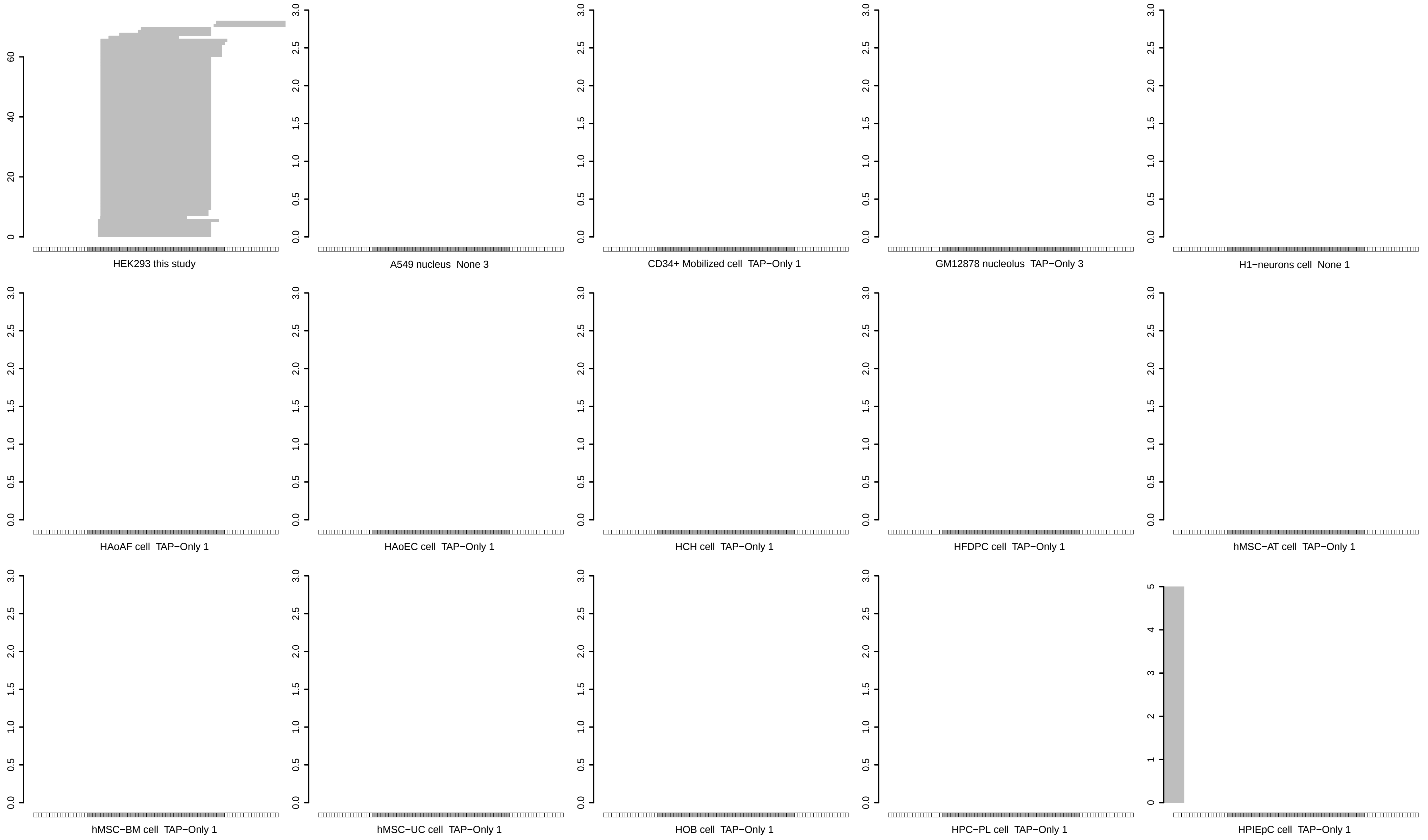

ZL101 chr16:67837905-67837935 (-)

CCTCAGTGATGTAACTTCTCTGCCTGAGAGG
((((.......................))))

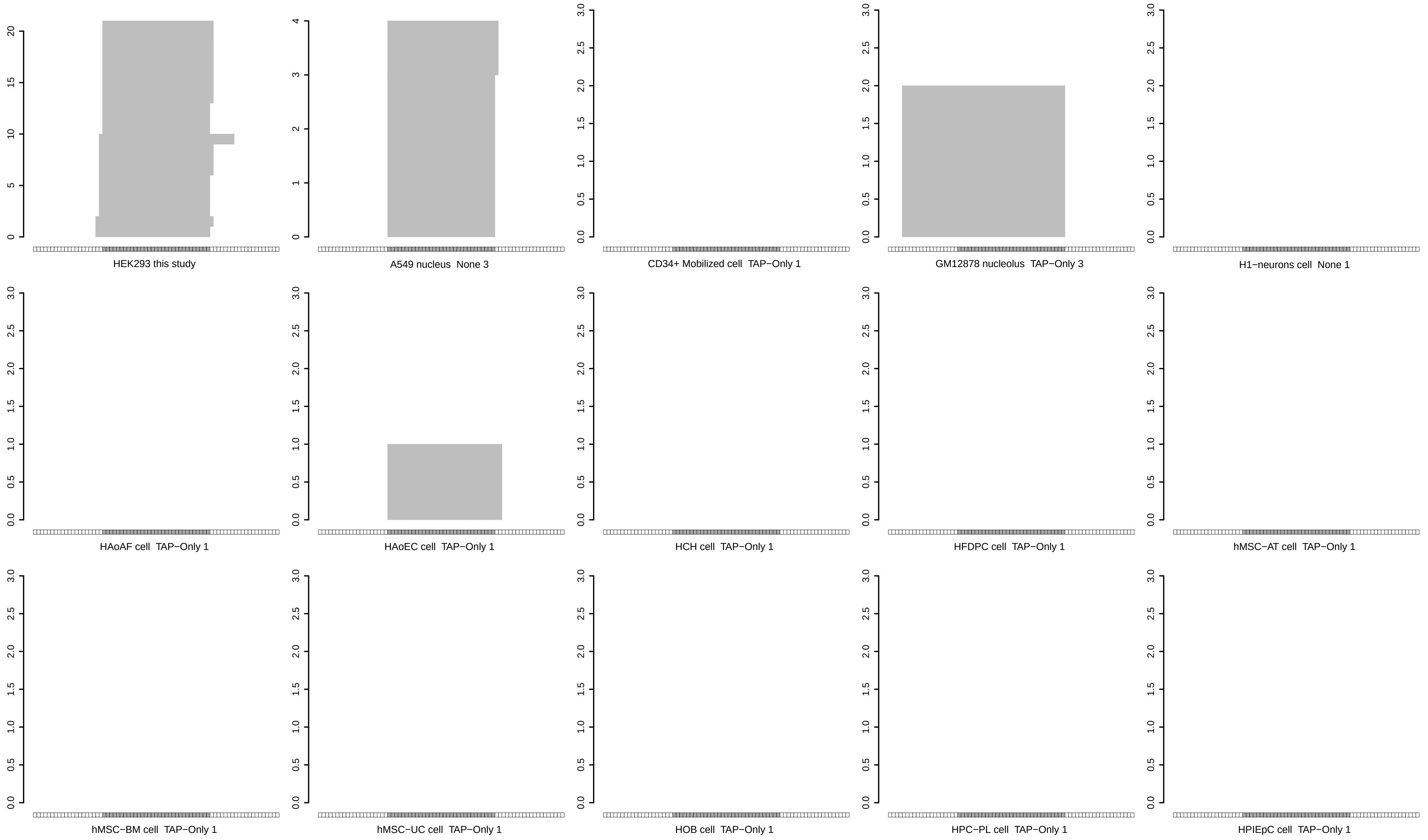

ZL102 chr12:132395704-132395806 (+)

GGCCCCACATGATGATGGCACTCCTTGTGTGACATGGGAGGGGTCTGCAGAGCACCTGAGTCGGCCATGAACCCCCGTCCCTCTGTCCATCTGACTGGGAGCC

(((((((..)))).)))

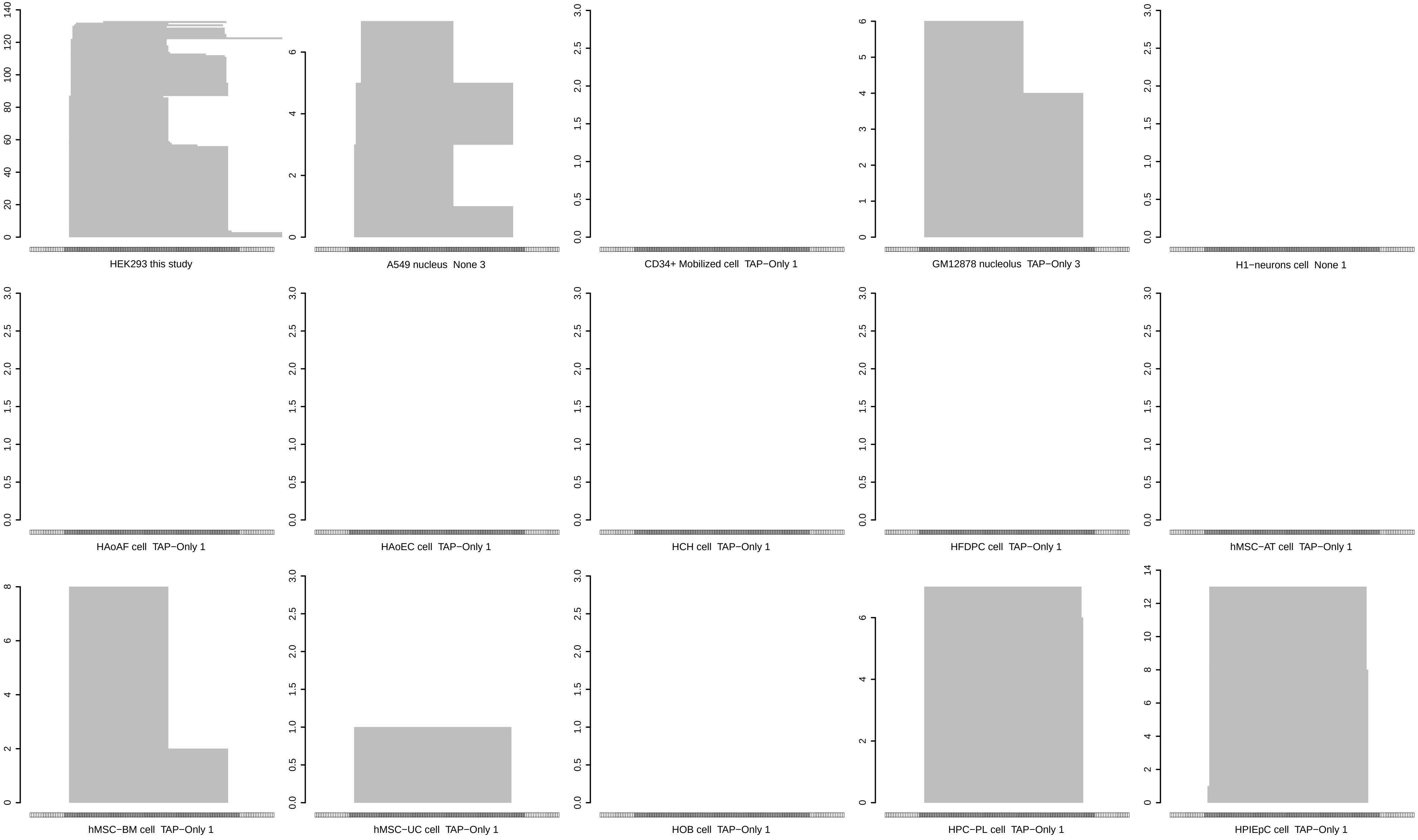

ZL103 chr7:28602948-28602985 (+)

CCTTTCGATGATTATCTCCATTTTAAAGATGAGAAAGG

((((((..........................))))))

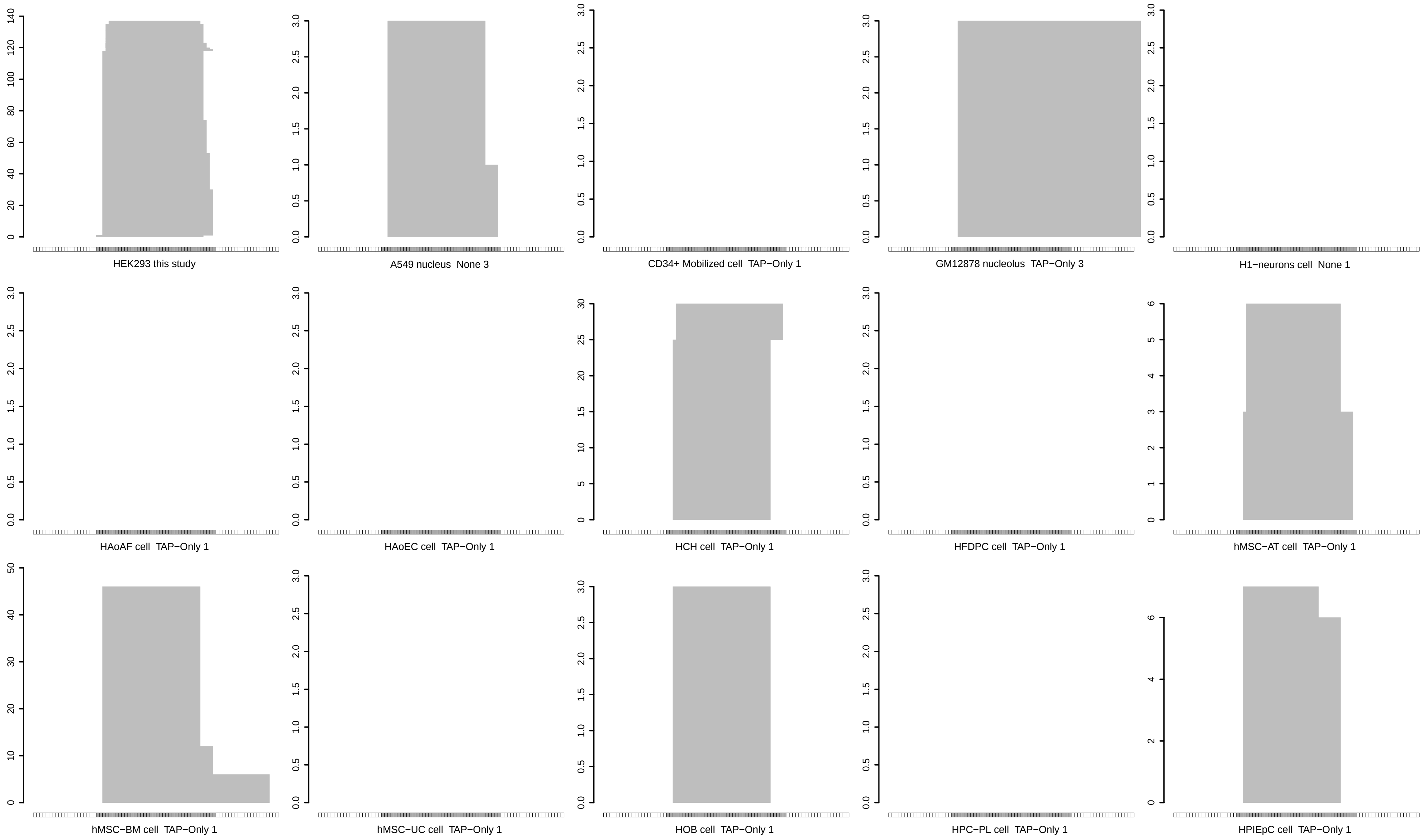

ZL104 chr1:11080232-11080326 (+)

TGTAATGGCTCATGATGTATTTATATCCATCATTATGTGATGCATGACTGTGTACTTTTGTAACTTAAAACGTTACTCTTCACTGAAGCCTTACA

(((((.((((..)))))))))

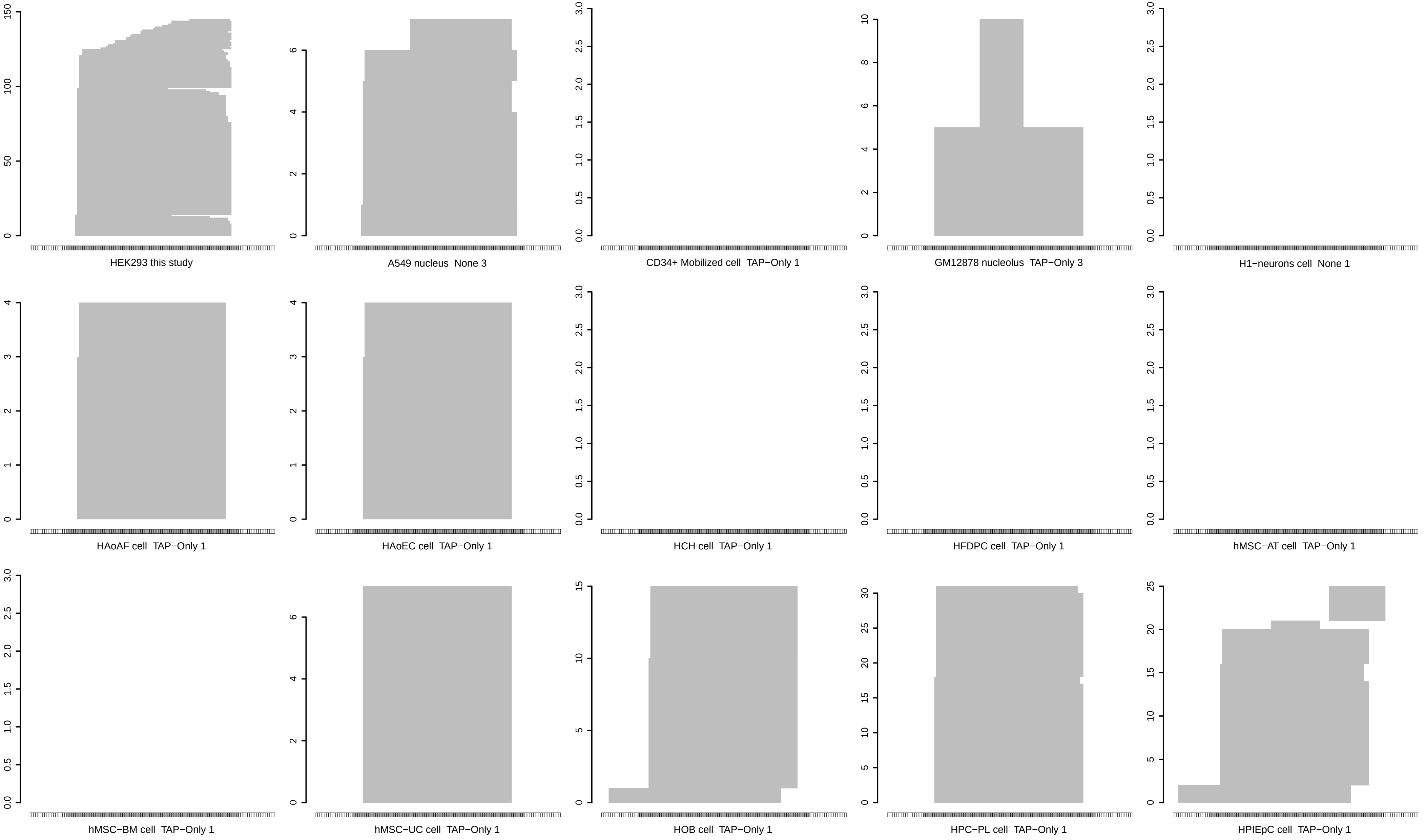

ZL105 chrX:54953738-54953866 (+)

GGGGTGCGCTCAGAGCAGAGGGCCTTAAGAATGGCTCCTCTGTTTACAACACACCCAACAGGAATCTGGGCTTACTCCTCACAGGTGGCATGATACTTTGGCCTTCCTGTGACATCATGCCCTATACAT

.(((((.(....((((((((((((........))).)))))))))....).)))))............((((.......(((((((.(((..........)))..)))))))......)))).......

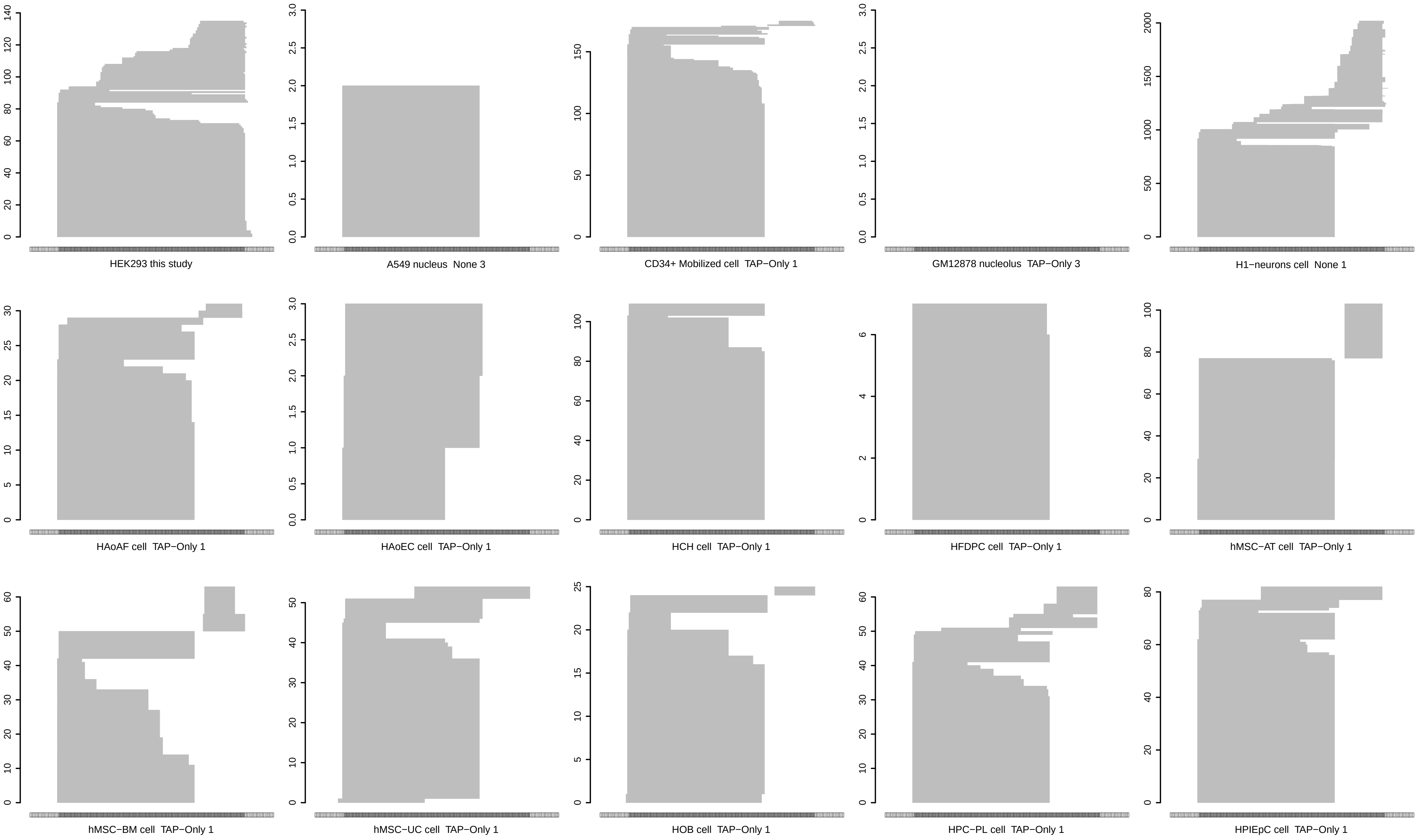

ZL106 chrX:114360879-114361007 (-)

CAGCATTGTGCAATGGCCTTTGGTTTTTCTTGTAACCAGTTAGGTCTGAAACCAATGCAAAAGAAATTTCCTTTTTCCACGAGAAACATCCCTGCAGTGGGTTGTTCCTATCGACAGGAGGTTACACGT

..((((((......((((((((((((........))))))..)))))......))))))......((((((((...((....((.((((.(((......))).)))).))...)).))))))))......

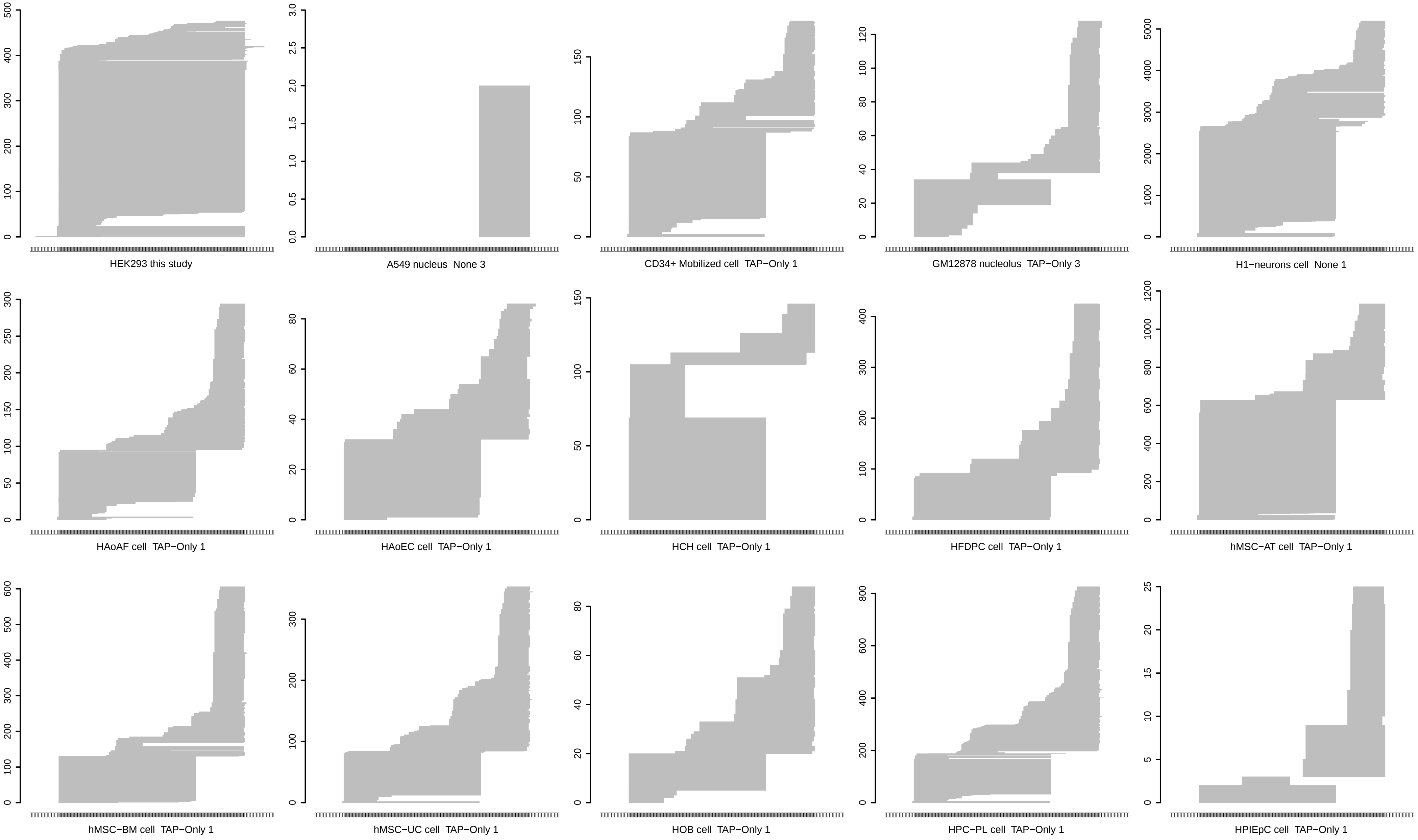

ZL107 chr5:85916323-85916386 (+)

GTGCATGTGATGAAGCAAATCAGTATGAATGAATTCATGATACTGTAAACGCTTTCTGATGTAC

((((((..))))))

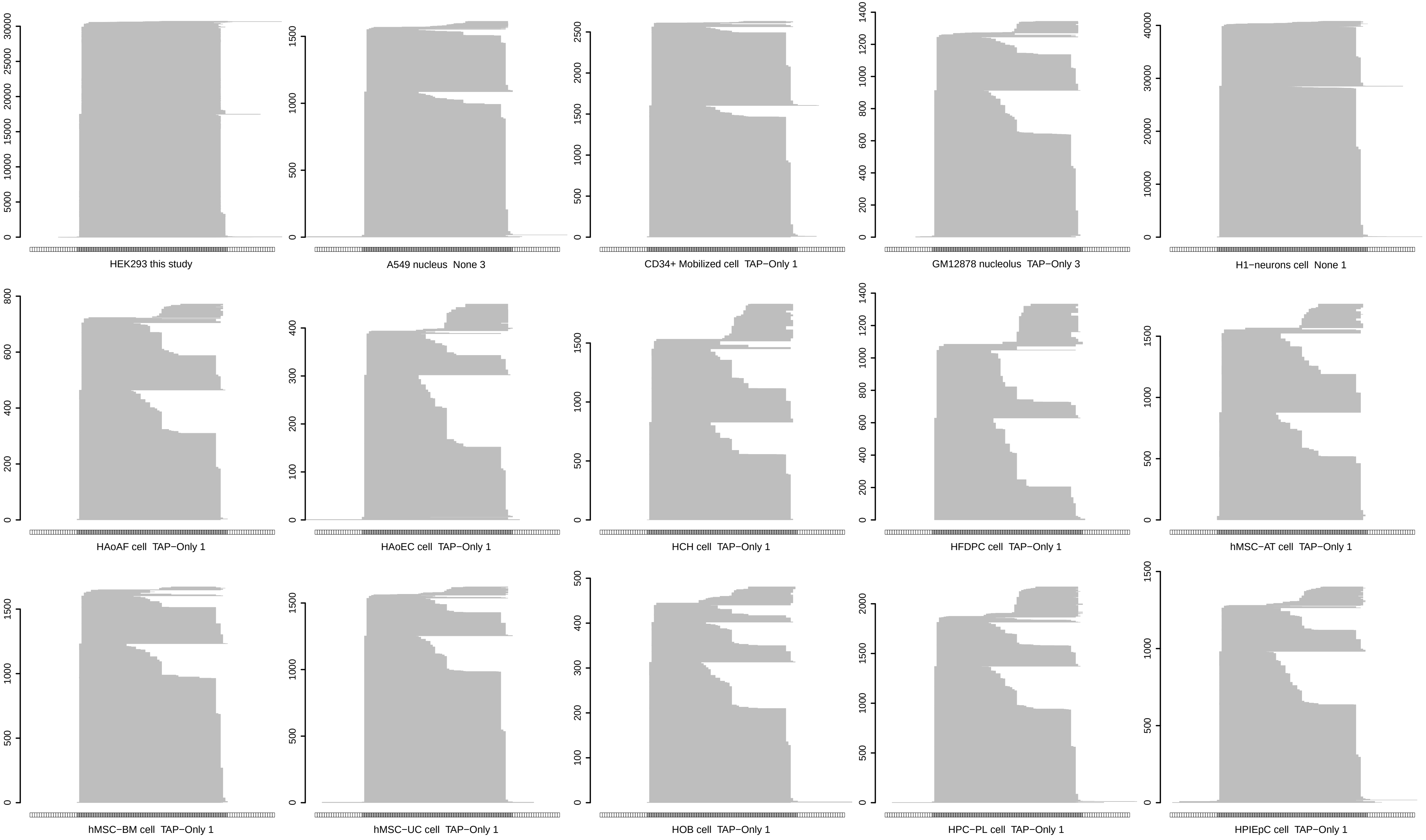

ZL108 chr17:37007762-37007911 (-)

CTCTTCTCAGAACACTTCCTGGGTCTGATTGGTGGCCCAGGGAGCTGTCAGAGAAGAGCAGAGCAAATGGCCTTCACTTTGTAGATGAGATGGCAGGAGGGTGGATTGTTGGTCTCAGTCAGTGGTGGGACAGACCAGAGCTCCGAACAC

((((((((.((.((((((((((((((........)))))))))))).)))).))))))))..((((...(((.((((((((((....((((((.((((.........))).).)))))))..))).)))))...)).)))..)))).......

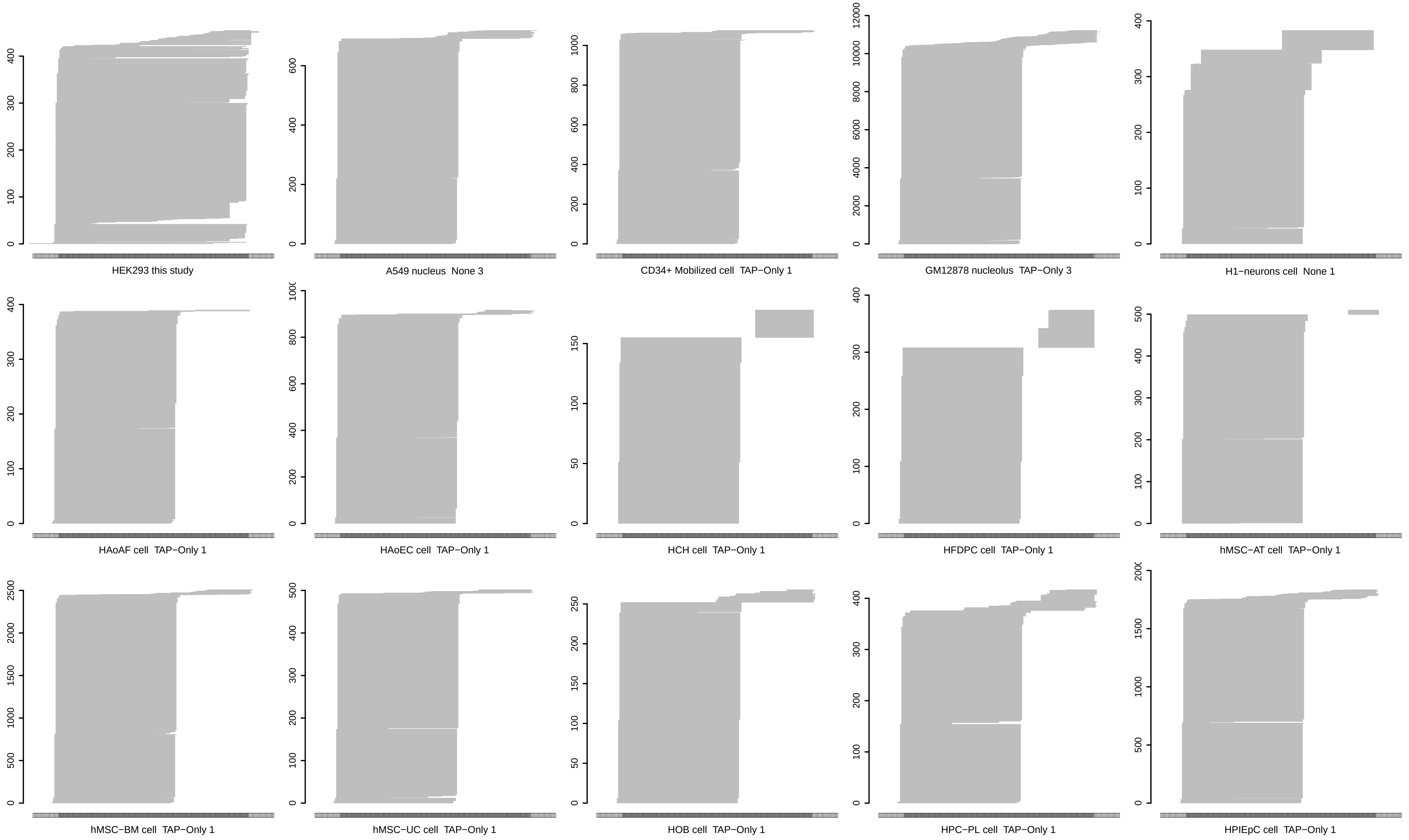

ZL109 chr5:137894658-137894730 (-)

TTGTGCCGTGATGTATTTGTCAACACATCACTCTGAAGAAAAGTATGTGGTGACTTTCTGTGACTGAGCATGA

((((((...))))))

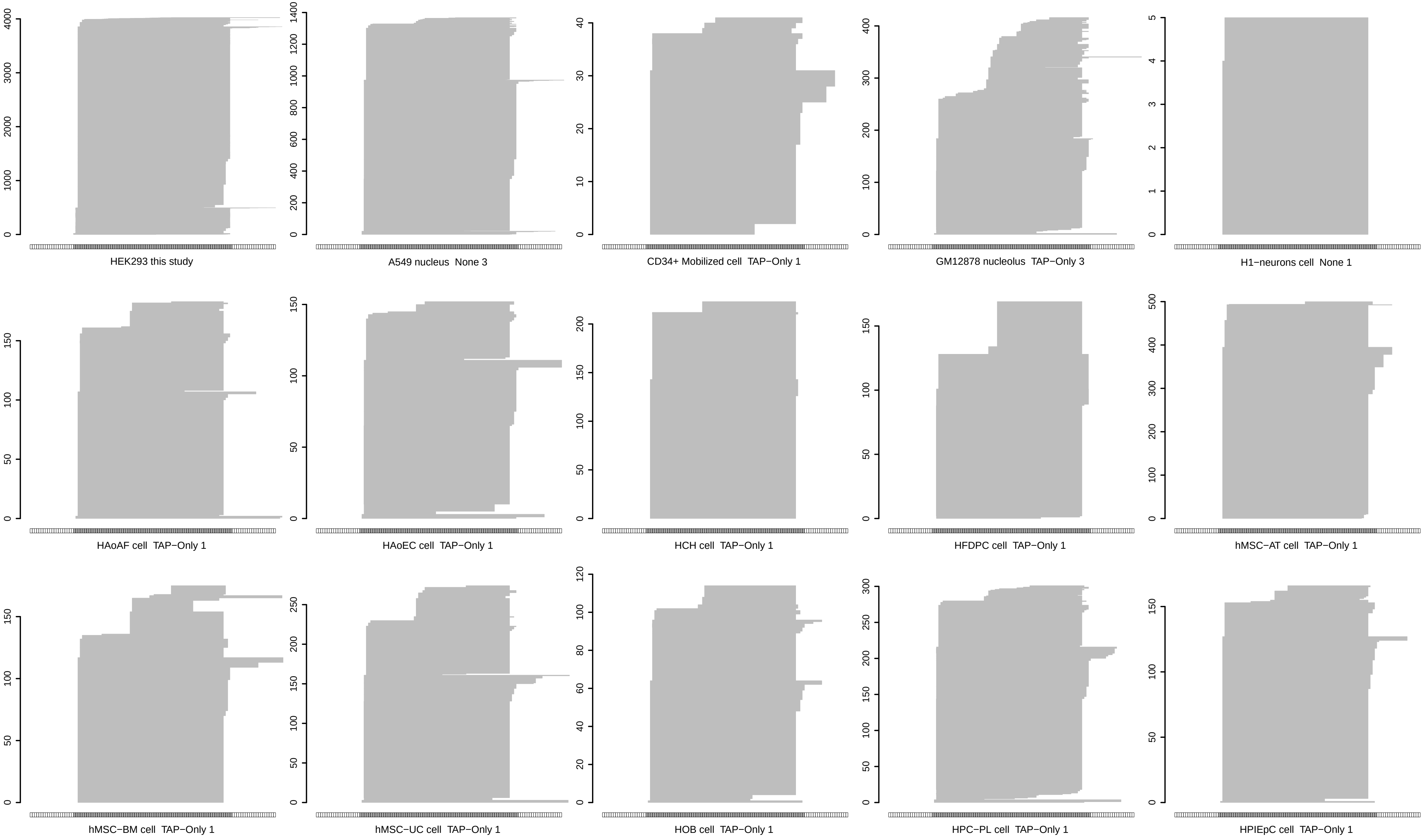

ZL110 chr5:138611868-138612011 (+)

CTCCAGCTGTAGGCAGCTGCCTAGGTTGTCTTGTACCTAGGCAAGTGTTACACTGCTGGGAGAACAGCAGCCAATAGCTGGTTGGCATTCTGGCCCTGGTTCATGCCAACTCTTGTGTTGACTACCCCAGGATGCCAGCATAGT

(((((((........((((((((((((........)))))))).)))........))).)))).................((((((((((((...(((((.((((........)))).)))))..))))))))))))......

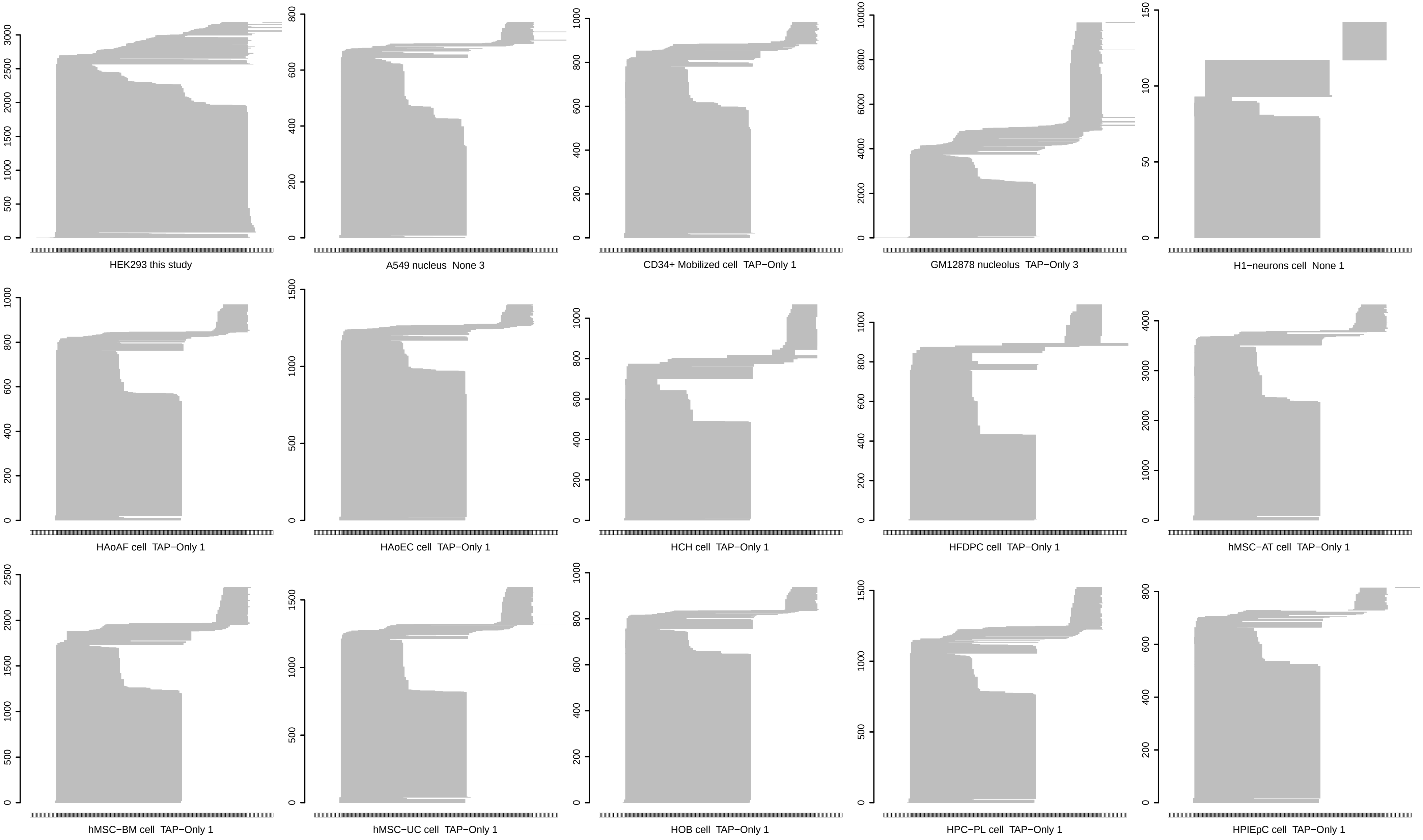

ZL111 chr10:74885838-74885965 (-)

GGGGTGTGCTCAGAGCAGGGGGCCTAAAGAATGGCTCCTCTGTTTATAACACACCCAACAGGAATCTGGGGTCAATGTGATGAGAGGCACAAAGCTTGTGGCCTCCCTACAAACAAATGCCTACATGT

.((((((....((((((((((((........))).)))))))))....)))))))...........((((...(((...(.((((((((.....))).))))).).....)))...))))......

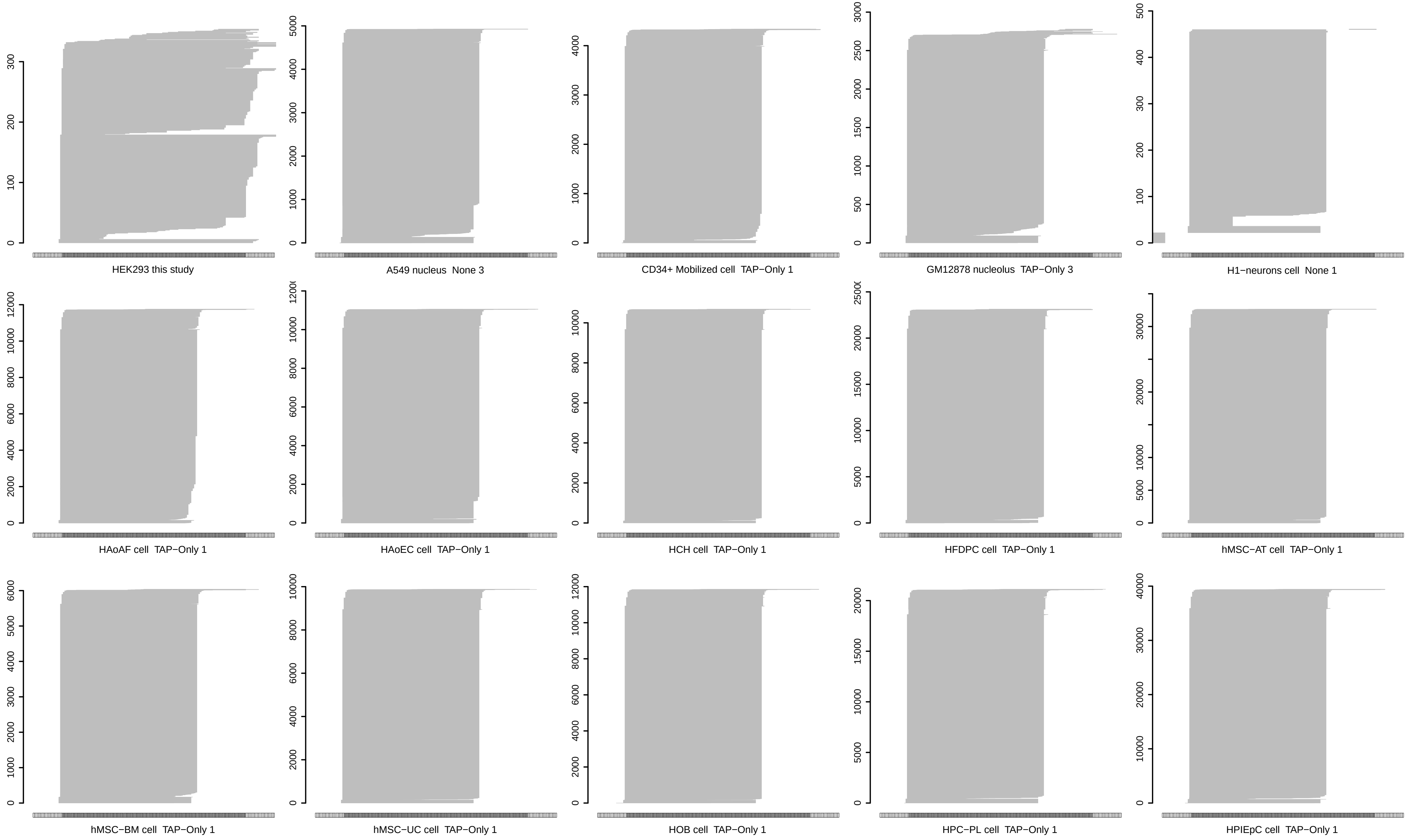

ZL112 chr1:154232203-154232337 (+)

GGGCATACCCGTAGACCTTGTCTGACTGTGCTCATGGCCAGGCAGGGGGGACAGTGTATGCAAGAGTAATGTGGAGTTTGTGCTAACTCTAGCCAGCTTAATTAGTGACTGGATAAATTGCACAACTCTCACATT

..(((((((.((...(((((((((.((((....)))).)))))))))...)).).))))))...........(((((.((((....(((((...(((.....)))..))))).......)))))))))).......

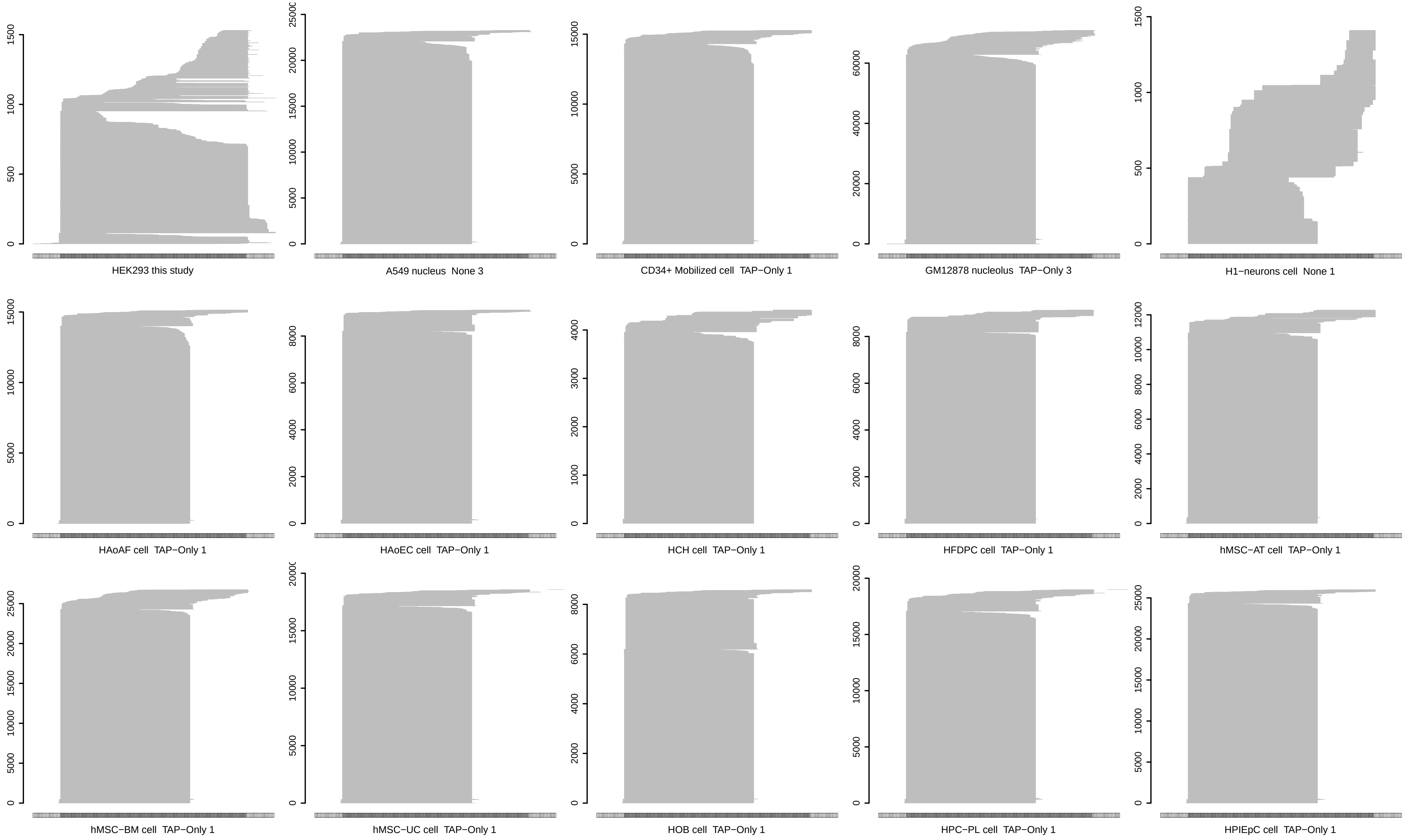

ZL113 chr13:45910449-45910582 (-)

ATGCATCTATTTGACAGACCTGGAGCAGTTGCTATCTGCTGCTATGGTTTCCACCACAGATGCAAGAAGAACATGTCCTTGCGCTTTCCGTCTGTCTAATTGTGGCAGCTGAGATTGAATAGAGGAATACAGGA

.((((((.......(((((...((((((........))))))..))))).......)))))))..........(((((.((.((((.(.((((((.......)))))).)))).))....)))))........

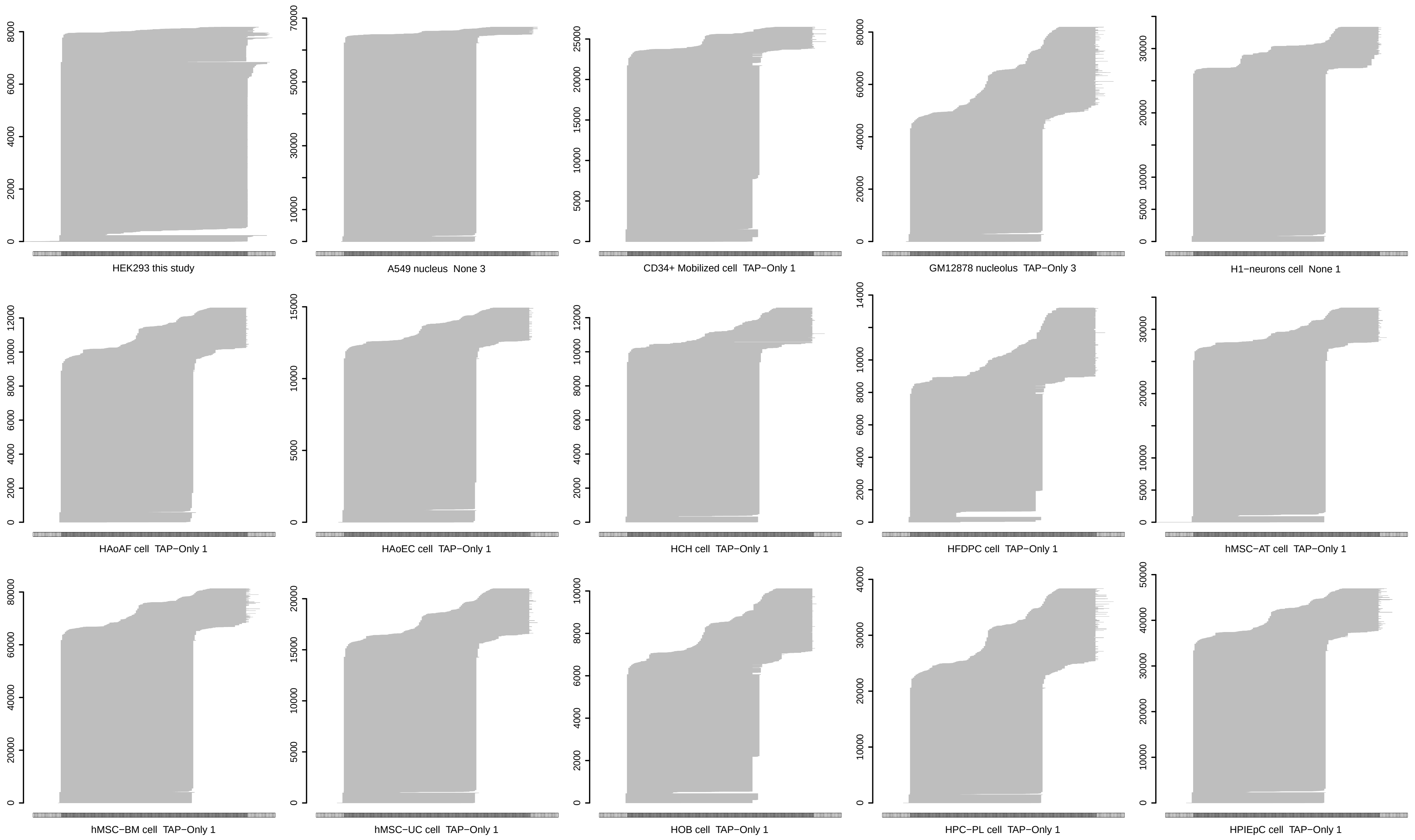

ZL114 chr9:20442184-20442242 (-)

TTTTTCTCCACAGGATGATTTATTAAACTACCCTCTCCCCCAATCTGATGGATGAAAAA

((((((((((......................................)))).))))))

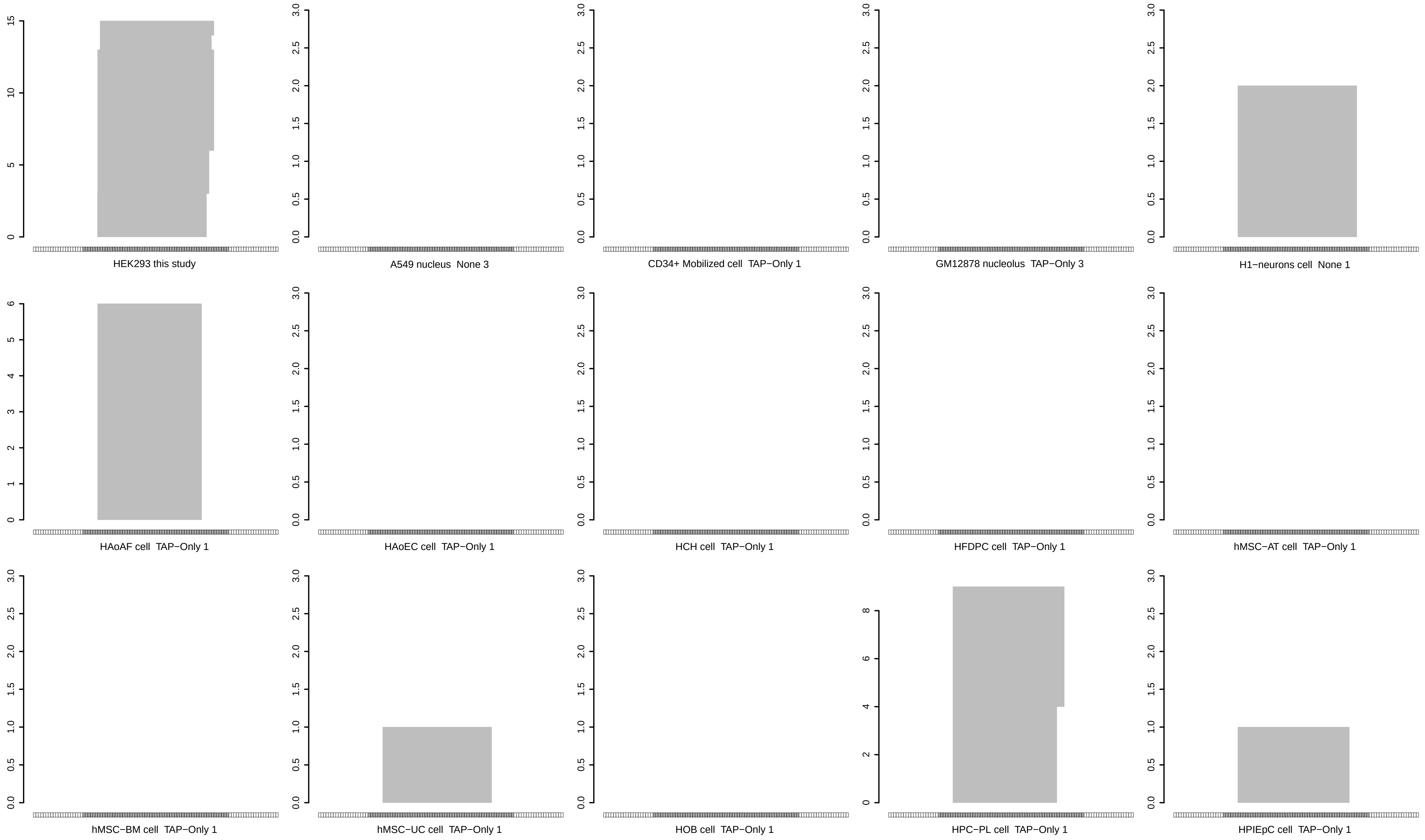

ZL116 chr19:39958862-39958933 (+)

TGGGCATGTGATGTGACCTTCCTGAGCCTGCATTCCCTCACCTGGAAATGATGAGCAGTTACTGATGCCACA

(((((((...))))).))

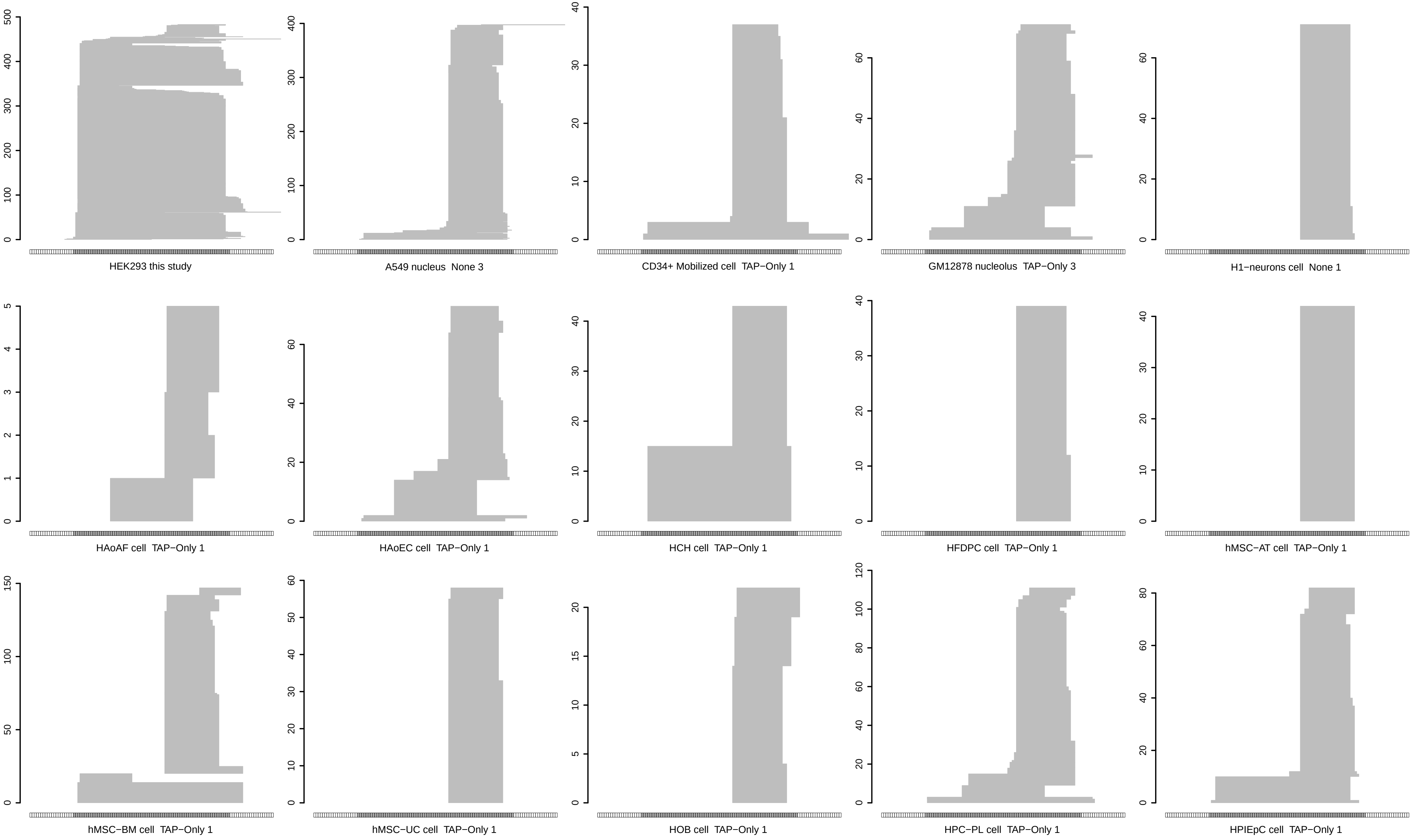

ZL118 chr15:100588039-100588172 (-)

GGTGAGGCTTGCTGTGATGATTTTCTGTGCTCCTTGCCAAATTAAGAATGAAATTCCACCCTAATGCTGAAGGCCTGGAGGAAGAGCTCACTGCAGGAAATCTGCTTGAGCACCTCGGTATGAGCAGCTGTACC

((((....((((...))))...))))

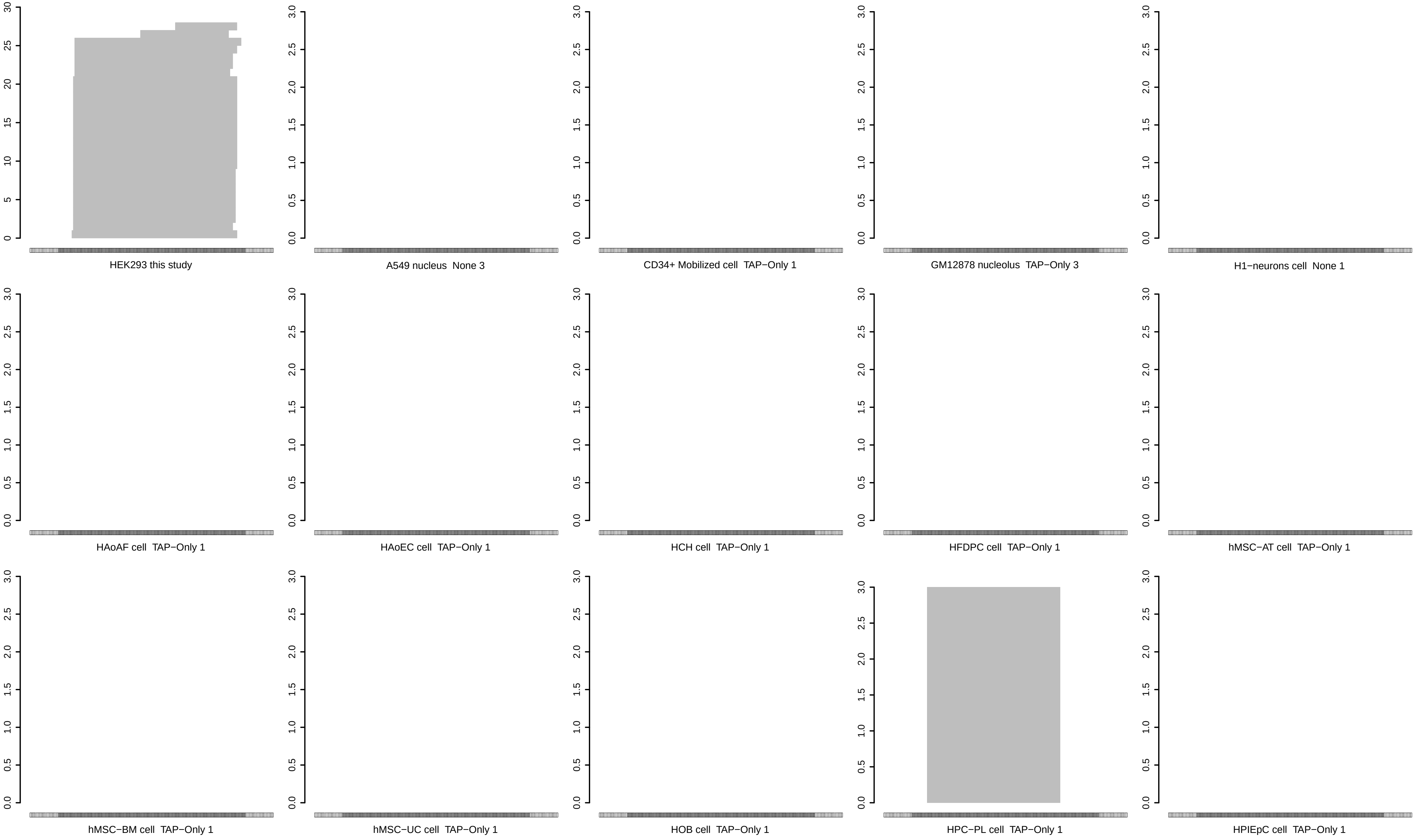

ZL119 chr8:59509871-59509905 (-)

TCTCTTCTGTGATGAAAATGTTTCTGAAGAGGAGA

((((((((...................))))))))

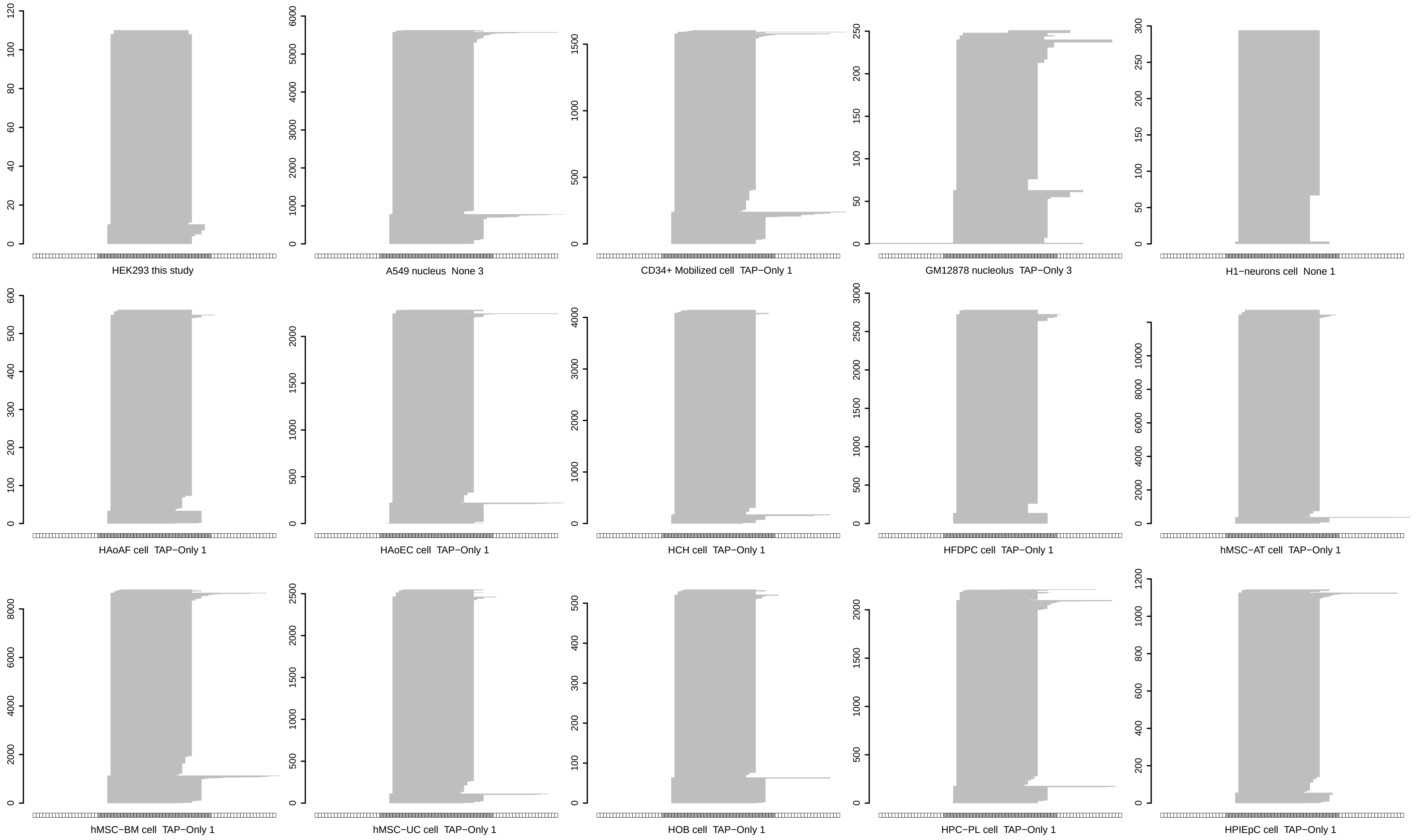

ZL120 chr10:74113771-74113928 (-)

CCTCCCGATGATGAGATTCGGAGCGGGTGGCCTGGTGGGAGGTGCTGACCTCAGCCACCGCCAGTCCTCCATCGCCCGCAAGAGTCCAGCGGCGGTCAGCCATGTGGACGCCGAGGCTGCTGAGTTGCCCTGGACAGGAGCCTCCTGAGCCAGGGAGG

((((((..))))))

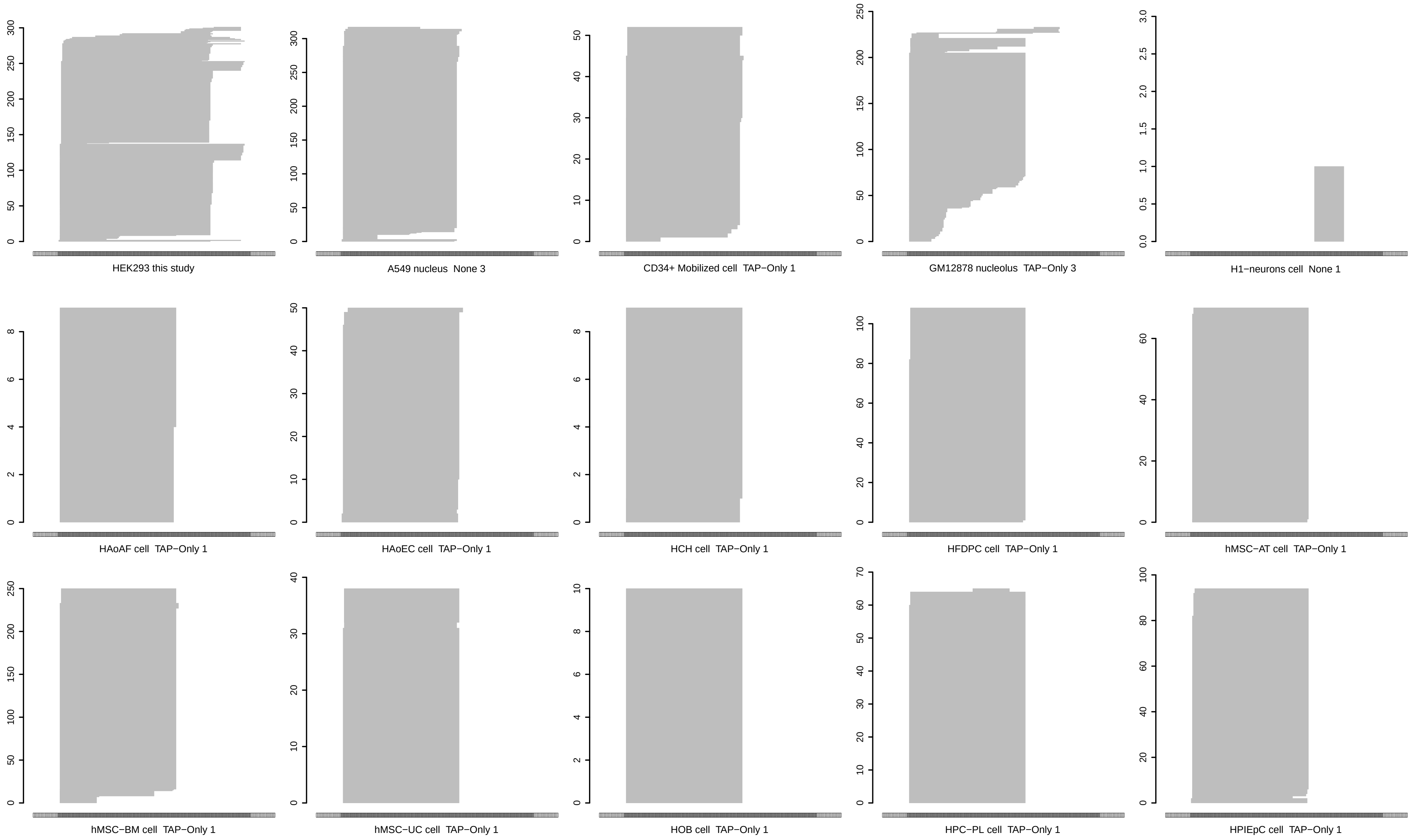

ZL121 chr18:76918328-76918363 (+)

CAGTGACATGATTATAGTTCACTATGATGATCACTG
((((((.......................))))))

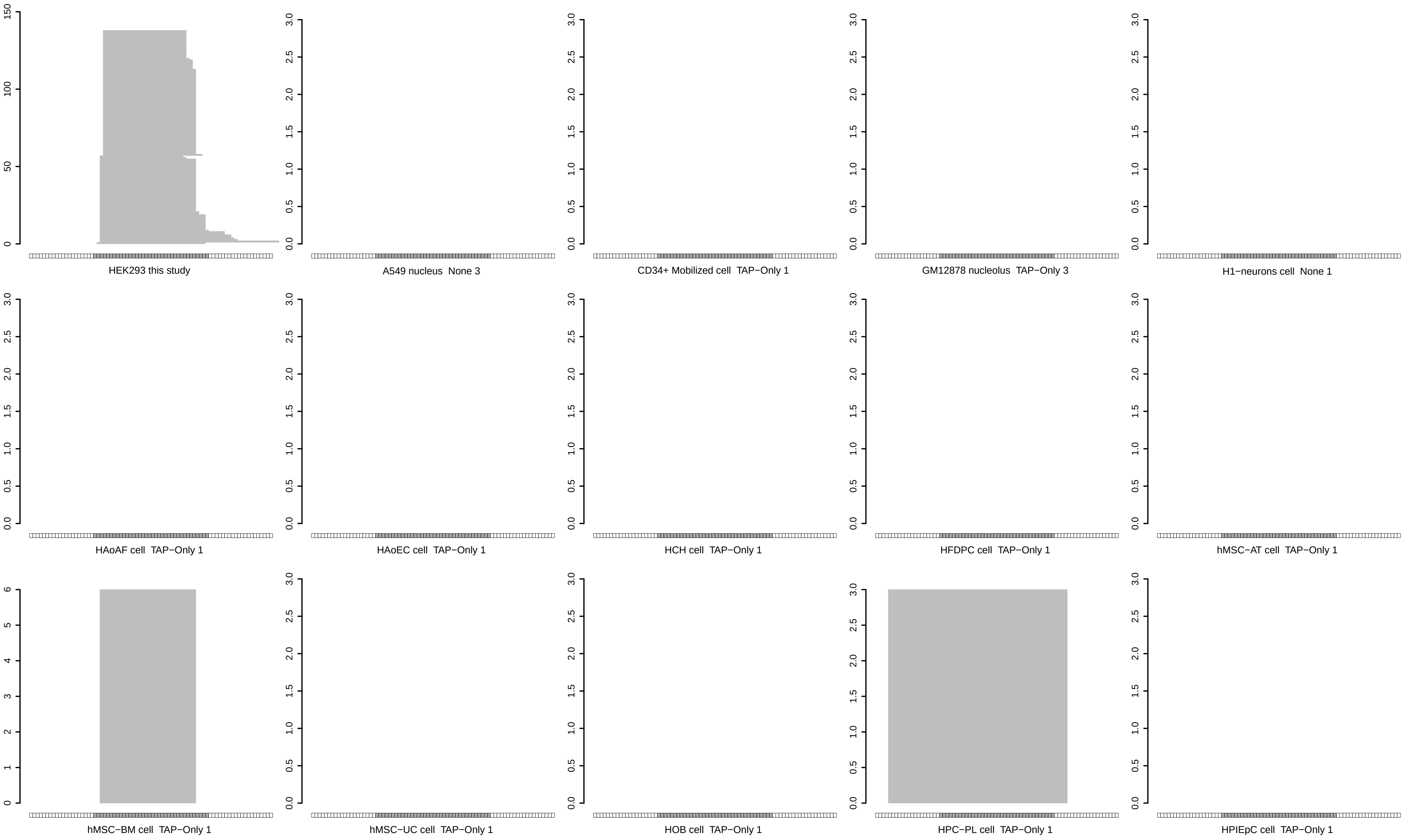

ZL122 chr16:10845220-10845342 (+)

TTCCGATGATGTCGCTCTGCTGTTCTCCCCGGATGGCTCCATAGAGGGCACCTGAGGCCAGTGCTGGTCTGTGGATGGCGTTTTCCCGTGCACAAGTTTGAGACAGCGCTGGGTGCTGAGGAA

((((...))))

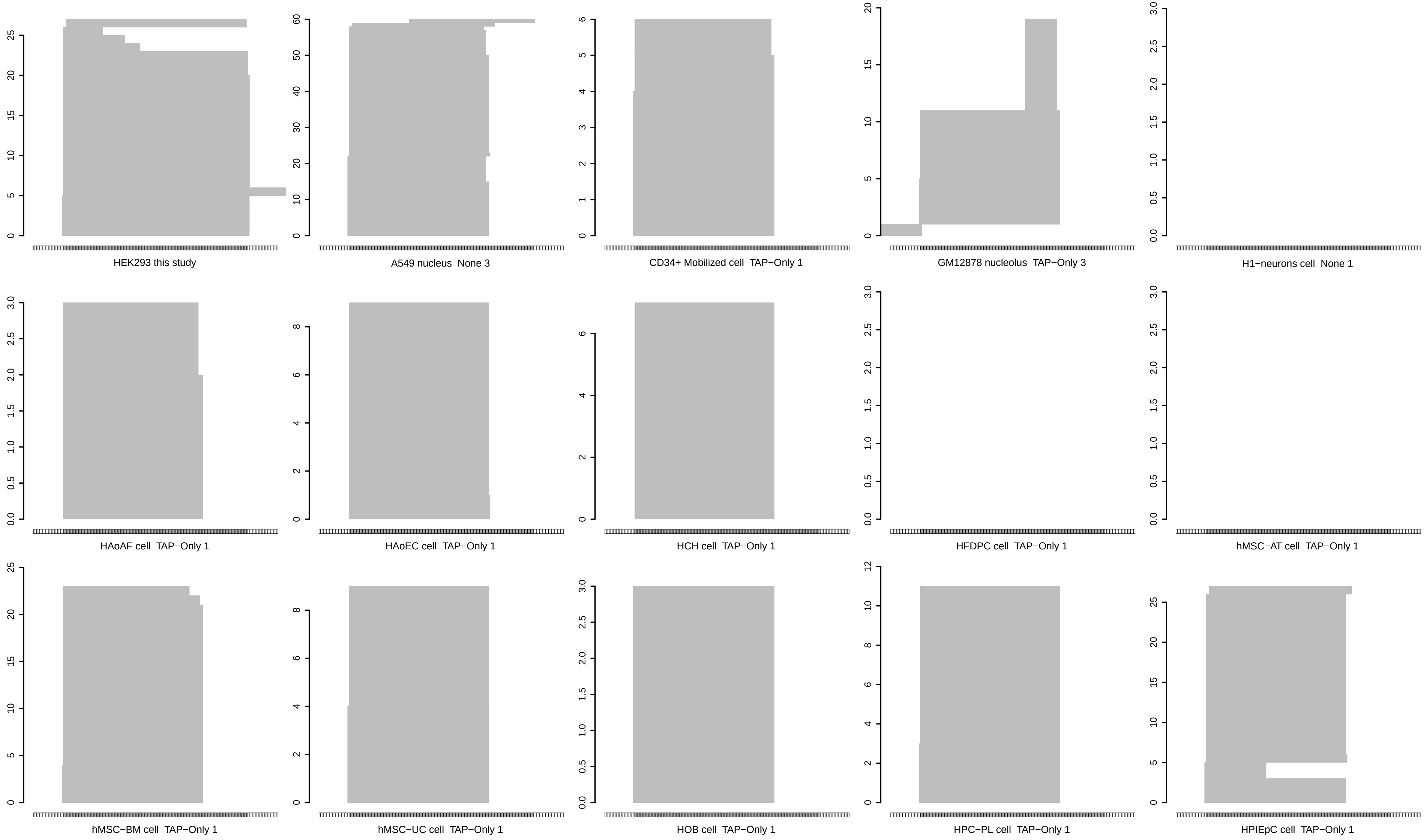

ZL125 chr10:75526199-75526232 (+)

CAGCCACAGATGATGGTTGTGTCTGATGTGGCTG

((((((((..................))))))))

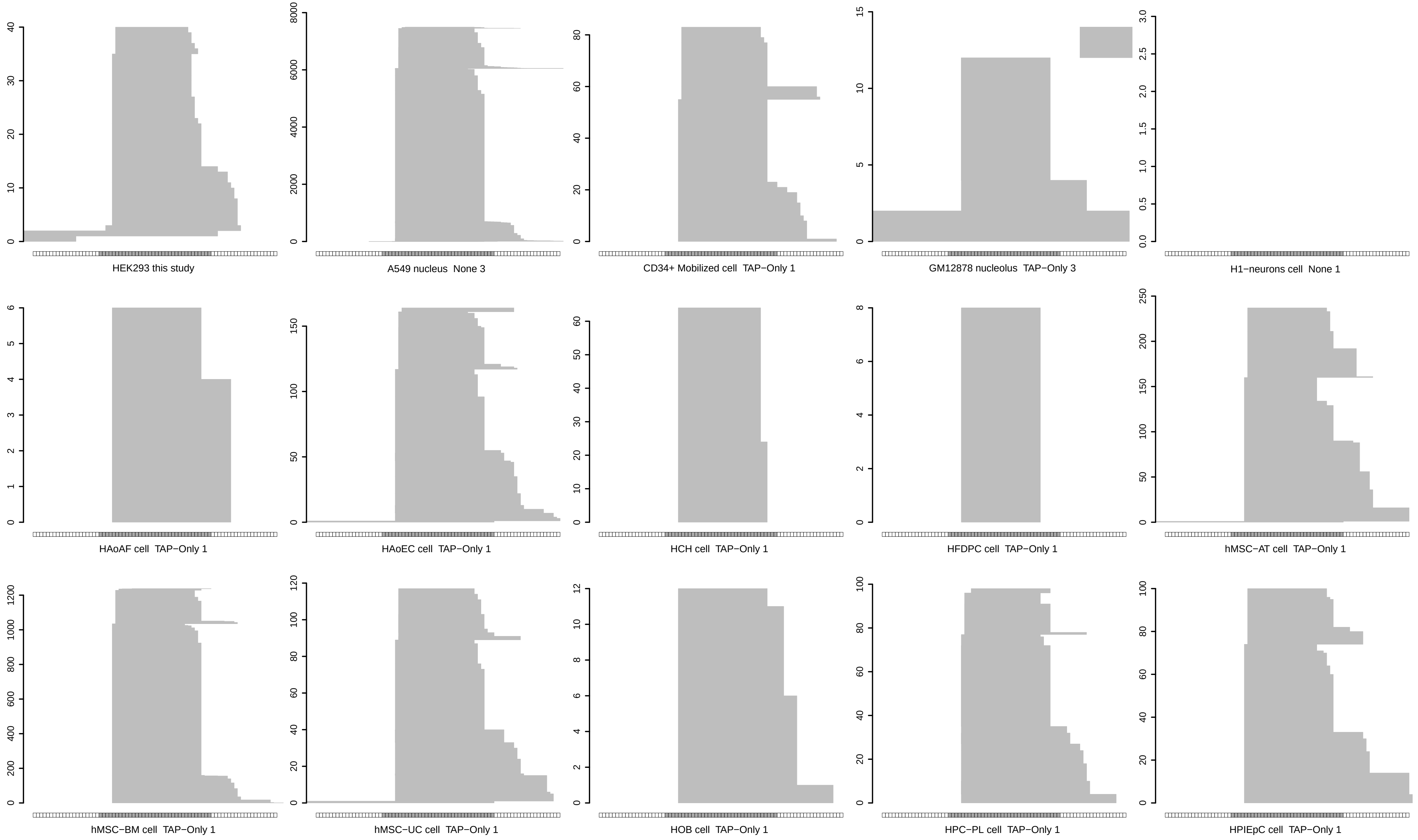

ZL126 chr3:52722904-52722971 (+)

GTGAGAAATGATGAGGGTCAACATTCTTCATACCAAAGTGAAGACATGAGATCCAACTCTGAGCTCAC

(((((..)))))

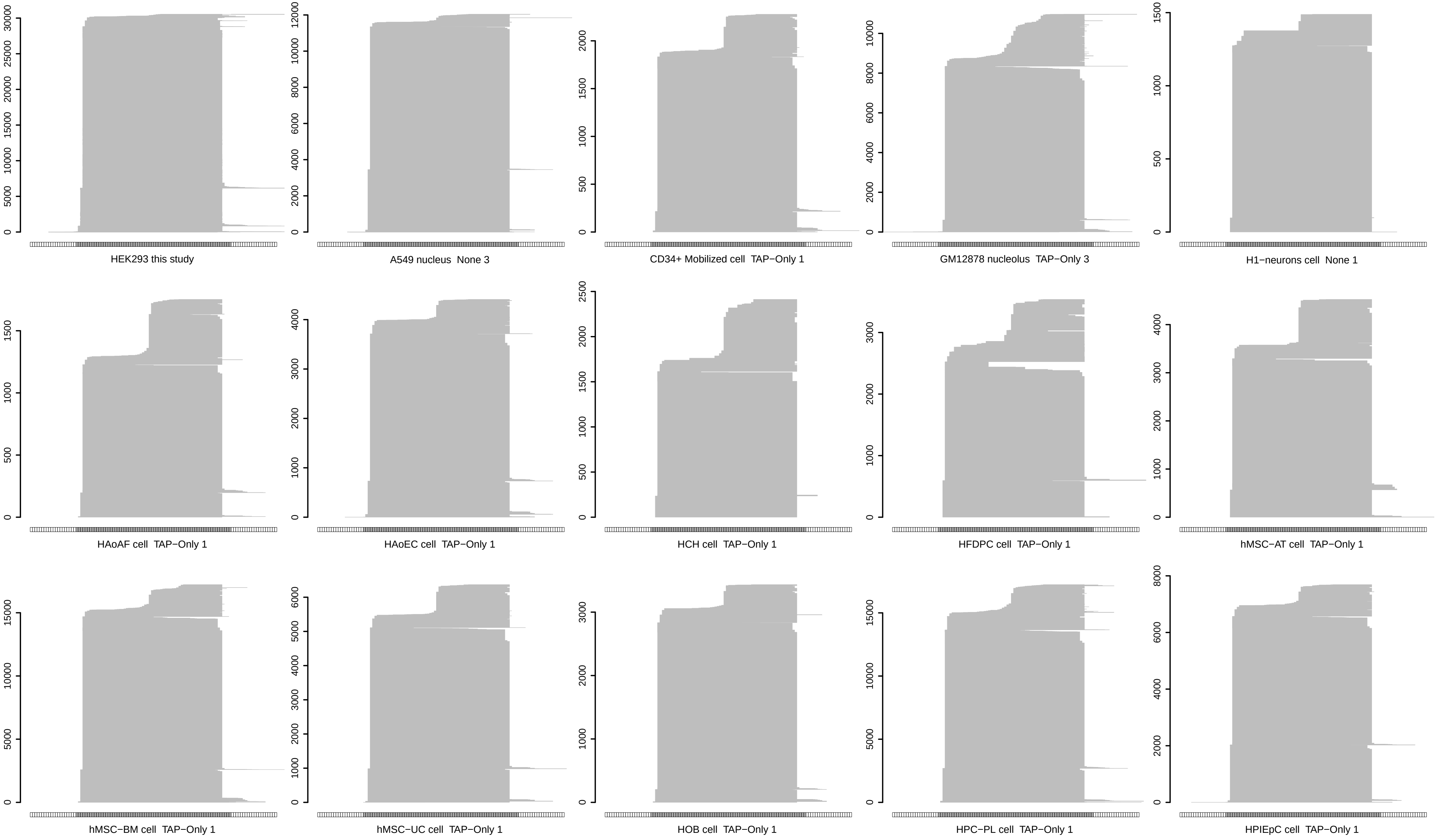

ZL127 chr3:52725392-52725462 (+)

GCAAGAAATGAAGAAACTAAAATTGGTCTTAGTATTGAAGTGAAGACACTGAGATCCAACTCTGATCTTGC

((((((...))))))

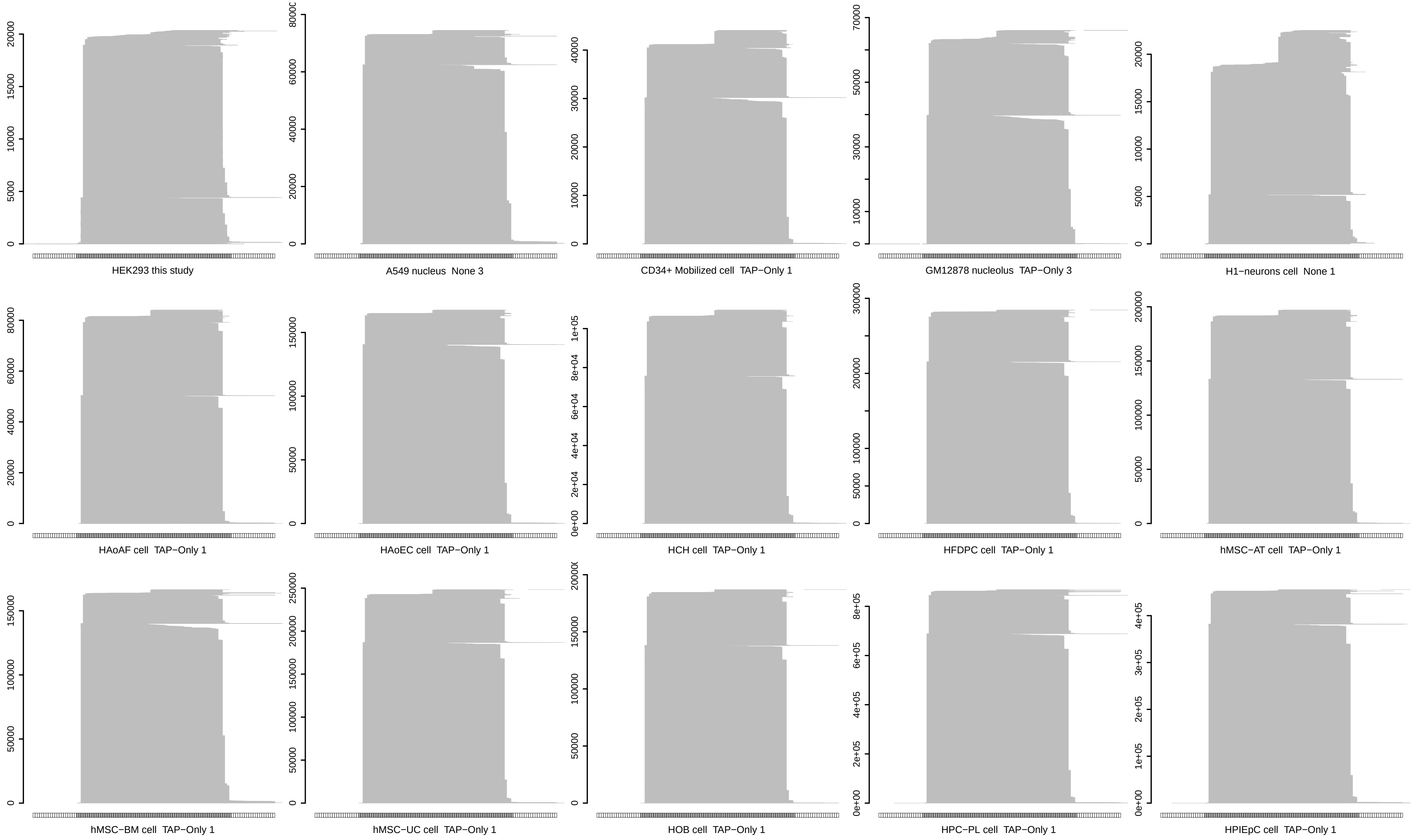

ZL129 chr3:183169639-183169778 (+)

GCTGGCAGGTTGGATTCATTGTTACAATATAGCTGTGAAGTGCTATGTTGTCCTTGCCCCCTGCTCAAAGCAATTGTTTCCCACAATCTCTGTCTCACTCGGTTCTATAGTAAGACATGGACGCTTGGTCTCAAACATTA

...((((((..((..........((((((((((........))))))))))).....)).))))))........(((((.(((...(((.(((((.(((.........))).))))).)))....)))....)))))...

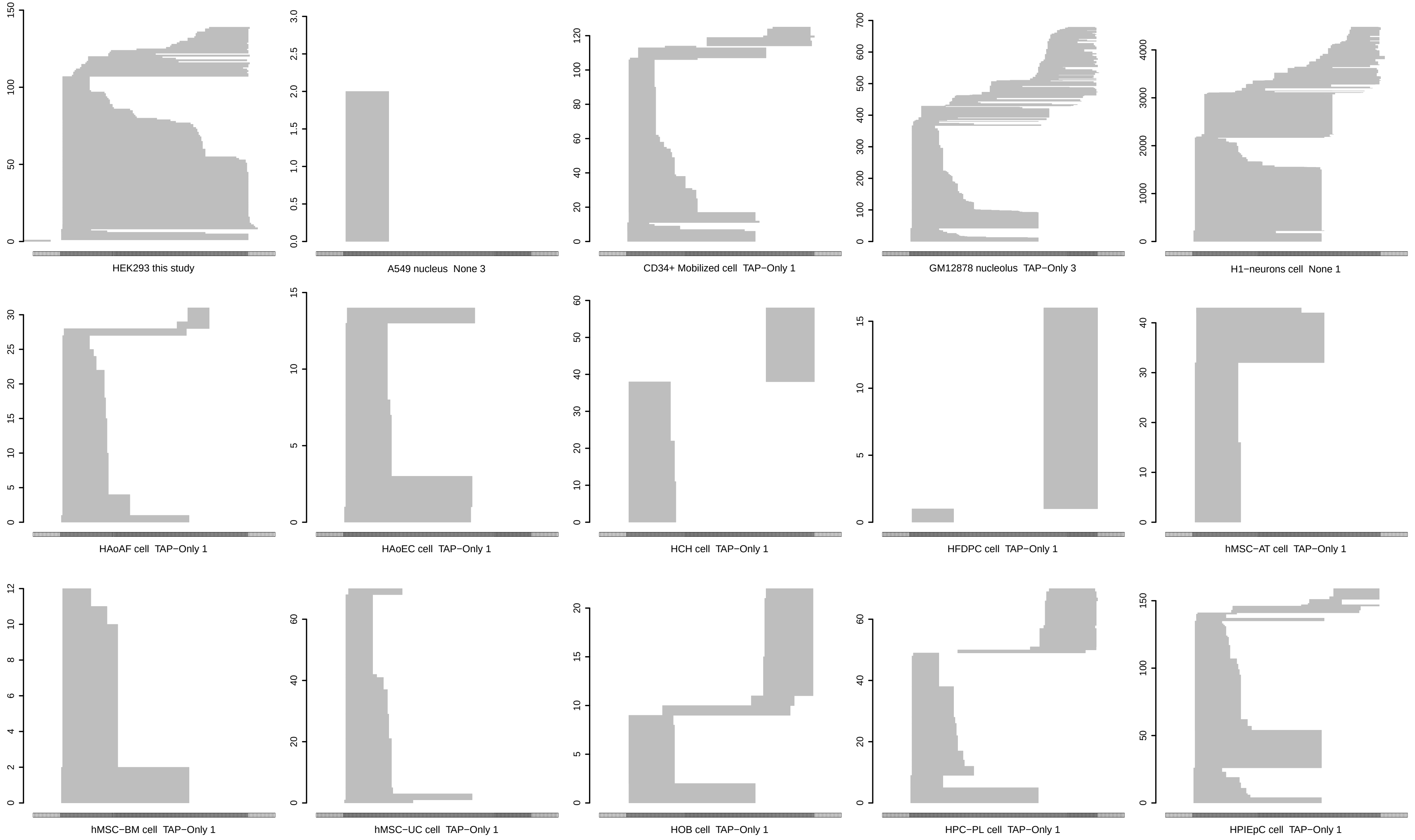

ZL130 chr3:183171600-183171735 (+)

TGAGGCAGGATCTAGTTACATTGTAGCTGTGAAGTGCTGCATTGTCTTTGCCCCCTGCTCAAAATAAAACTGTTACCTTTCAAGCCCTGTCTGCCATGGTGCTGTAGCAGCAGGGATGTTTGGTCTCATACATGTT

((((.((((...(((..(((.((((((........)))))).)))..)))...))))))))............(((...((..(((((.((((.((......)).))))))))).))...)))............

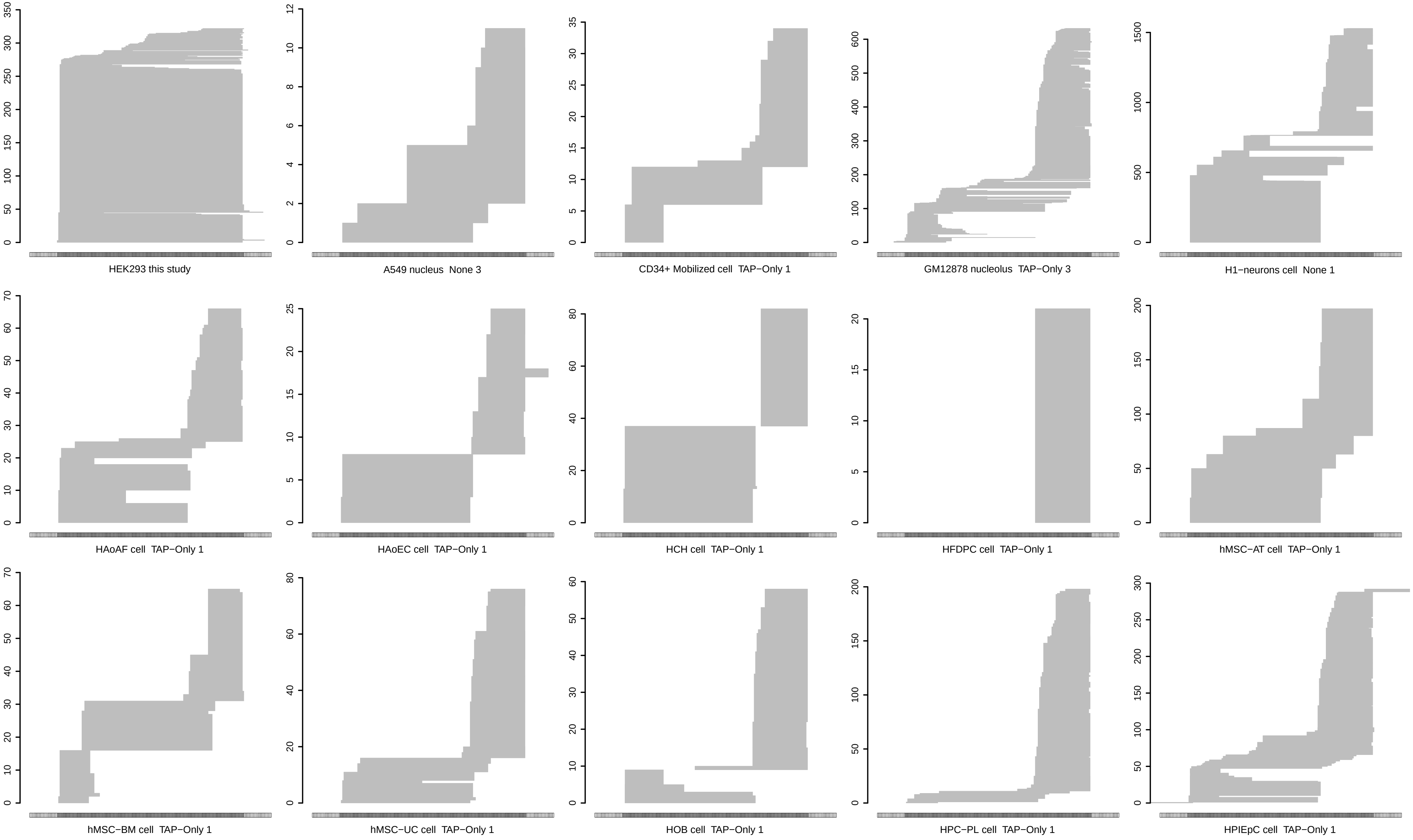

ZL131 chr7:6056509-6056644 (+)

CATGGGTTTGGATTATGACAGGCCCATCCCCCTGGACCTCTCATAGTGCCCCATGCCAGAGCAAACTGTGGCCCCGAACCATTGCCTGGCTTCTGTACCCGTGGGCCACTGGCACTGAAGAGGGTTACACAGTGGG
((((((...(.((((((((.(((.(((......))).))).))))))).)))))))...........((((((((....((.((((((((..((....))..))))))..)))).))..).)))))))........

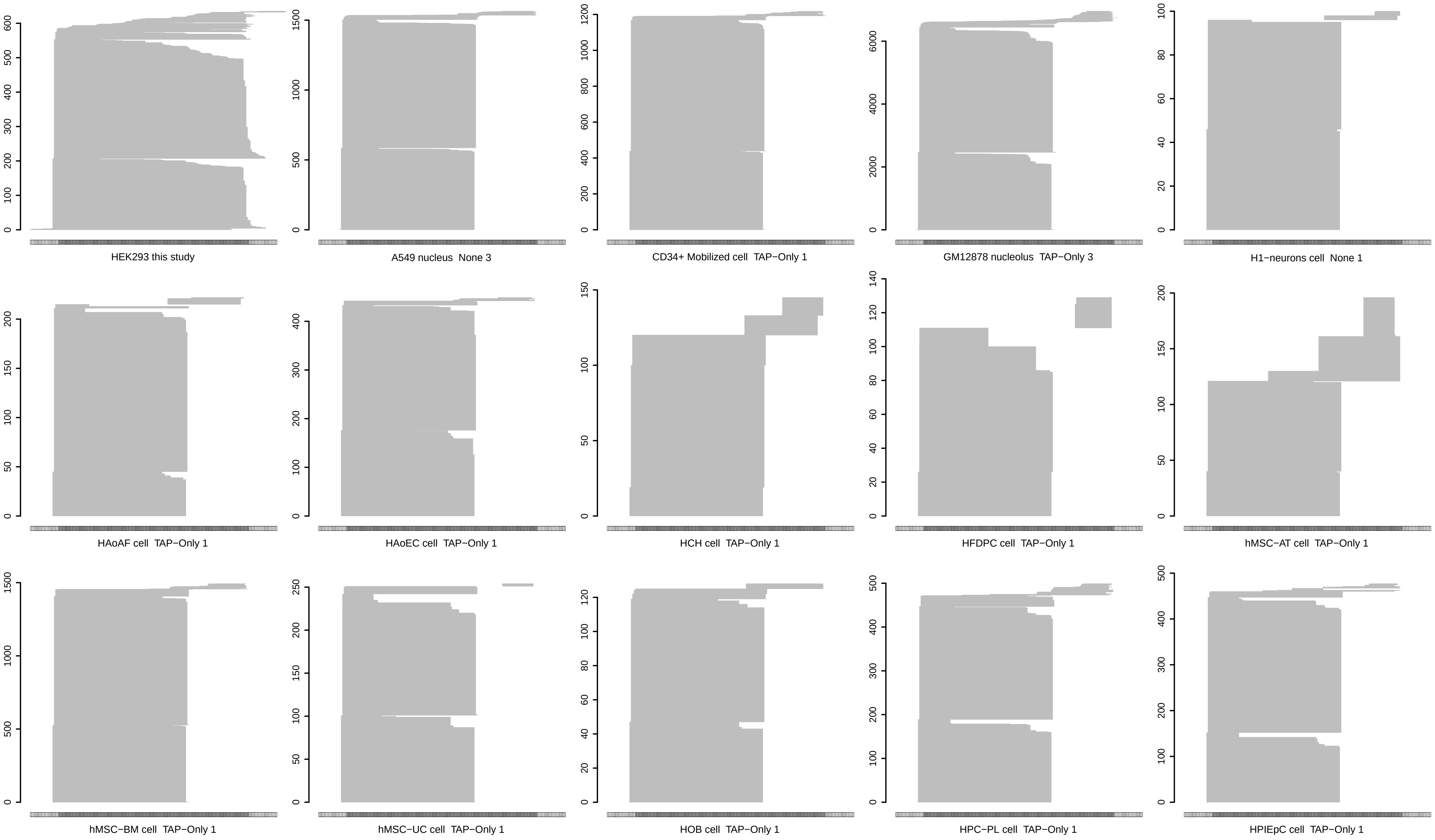

ZL132 chr2:203142832-203142914 (+)

TCAATGTTGTCAATGATGCATTCTTATTGGAACTGAATTTAAGTGATCTGACTCATTCGTCACTACCACTGAGACAACATTGA
(((((((((((...)))))))))))

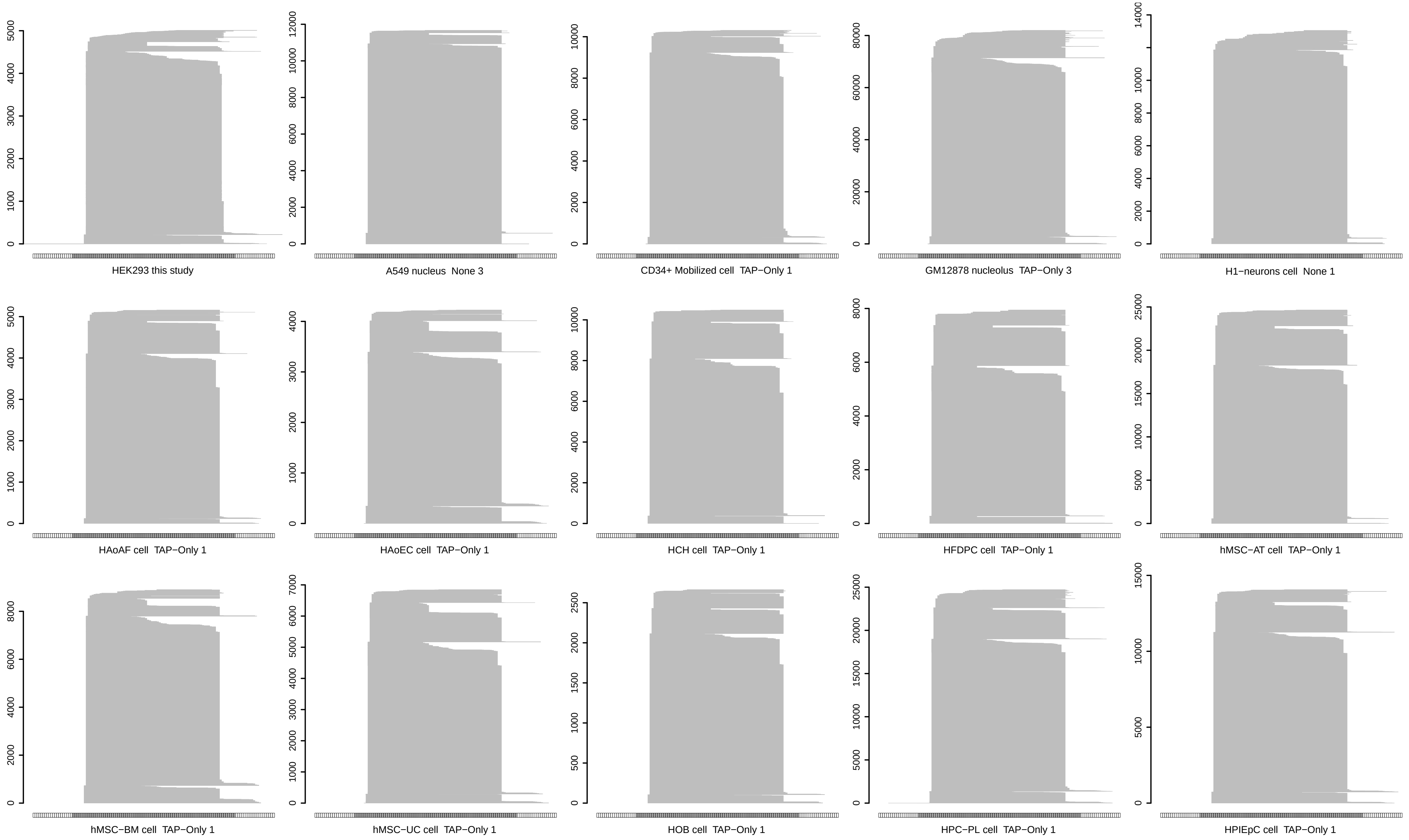

ZL133 chr22:20113923-20114047 (+)

TTGCAGACTCACTCTGCATCTGACTATACTTCCAGGCGGGTGCTTTTTCTGTCTGCCAGATAAACATTCCAGGGTGCTGTGGCCGCCTCACGTATCCAGAGTGATGCAGCTCCCTGGGGACACAG

..((((((.......(((((((.((.........)).))))))).......))))))..........((((((((.((((((..(((.((.........))))).)))))).))))))))......

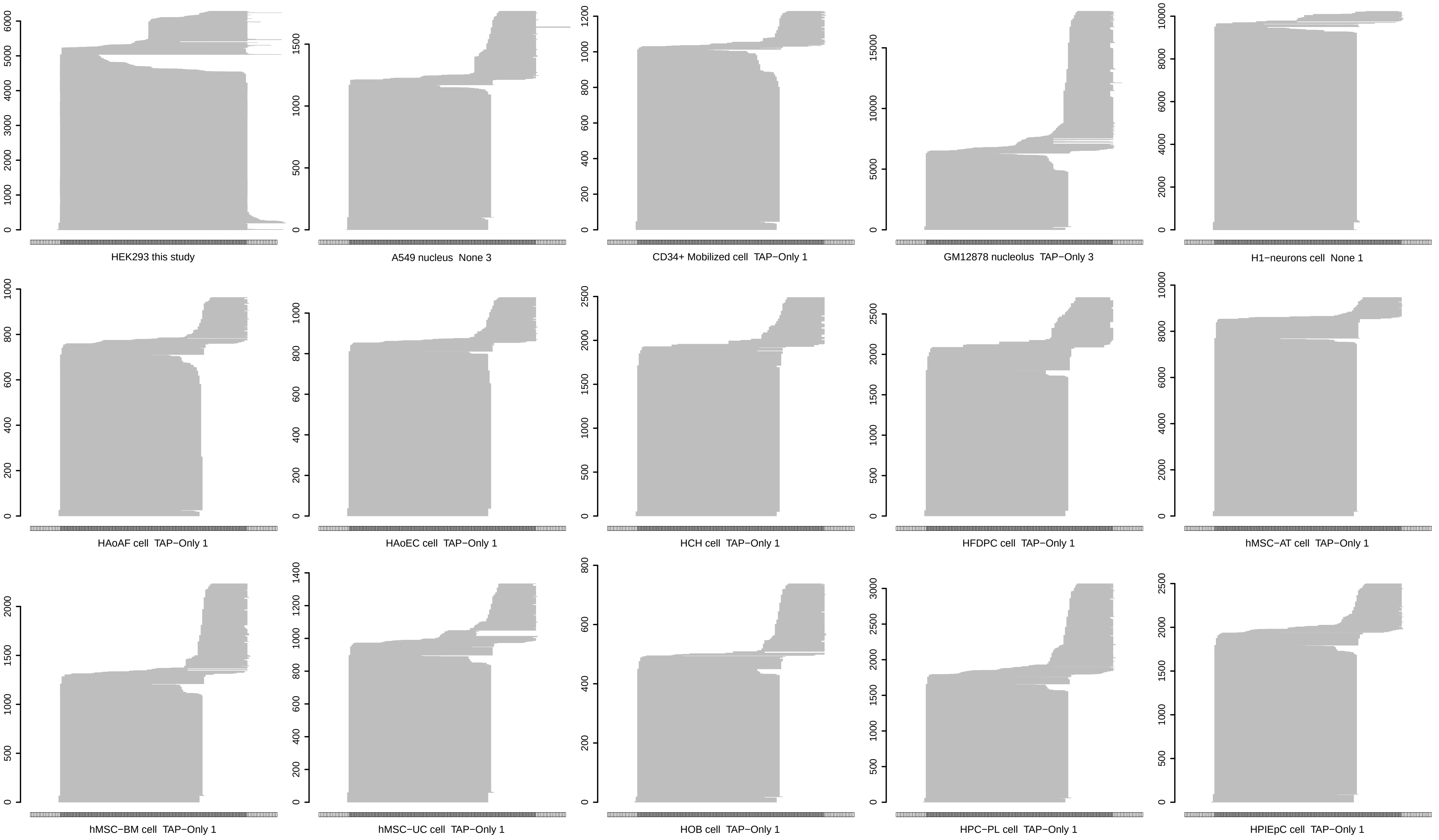

ZL134 chr14:20791338-20791487 (-)

TTTGATGGCTGTTCCTCTCACTGCTTGAAGCCTTAGGCAGTGGGATTTTGATCCATCATATATCAAAAATGGCTTATCTTCACTCAGGGCACCATGAGGATGGGCTGGCTGTCCGTTAGTGCCTTCTGATTTTTGCGGAGTCAAACAATT

..((((((.......(((((((((((((....))))))))))))).......))))))...........((((((........(((((((((......(((((((.....))))))).)))))..))))........)))))).......

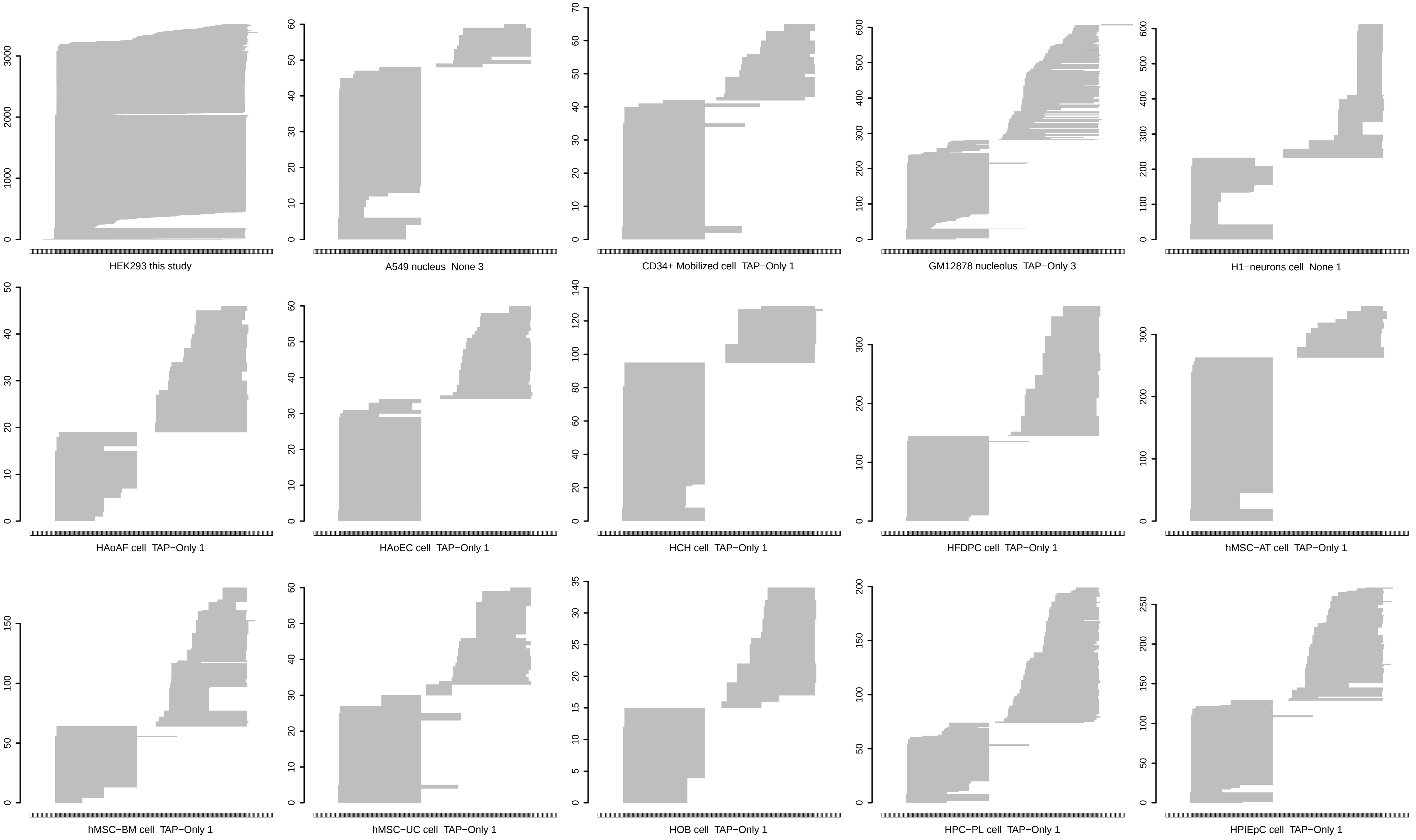

ZL135 chr15:65577799-65577930 (-)

ACTCCATGTATCTTTGGGACCTGTCAAGTGTGGCAGTCTCCCTTCCTTGCCATGGAAGAGCATATTCTTGTTTACCAGCAAAGCTGTCACCATTTAATTGGTATCAGATTCTGACTTGCACAAGTAACATTC

..((((((((.....((((.((((((....))))))..)))).....)).)))))).........(((((.((.(((.....(((..((((......))))..)))...)))...)).))))).........

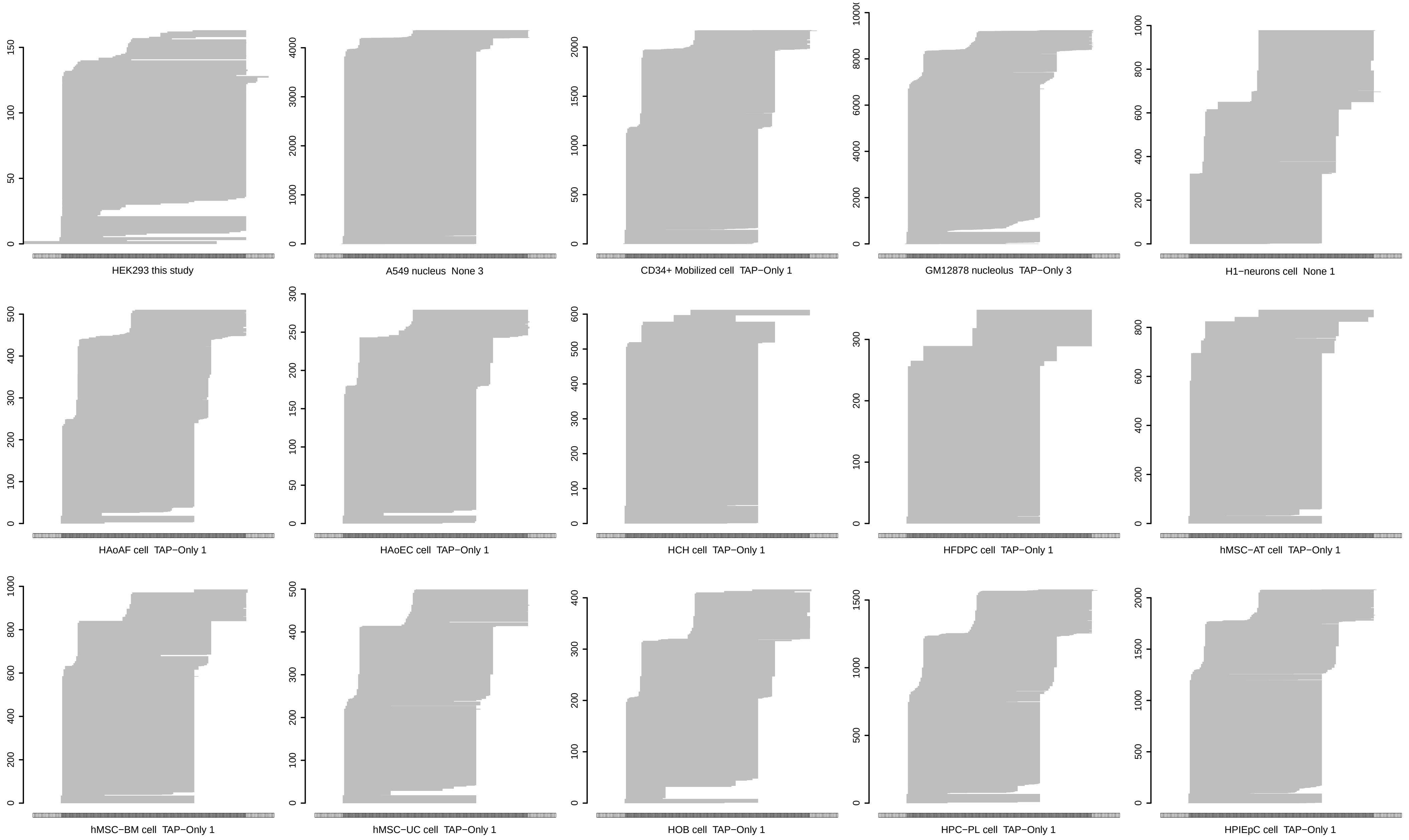

ZL138 chr1:193026411-193026546 (-)

GTTGCATGTGGTAATGTCTGCTCTTCATTCCTTTGGGAGCAGAAATACATCCATAACACGCTAAATTAAAAAAGGAGTTCTCAGGGCTGCCAACCTTACAGTAGAGGTTGAGTGGTAGTATATTACTCCTACATAA

......(((((.....((((((((.((......)))))))))).......)))))................((((((.......((..((((((((......)))))))).)..))........))))))......

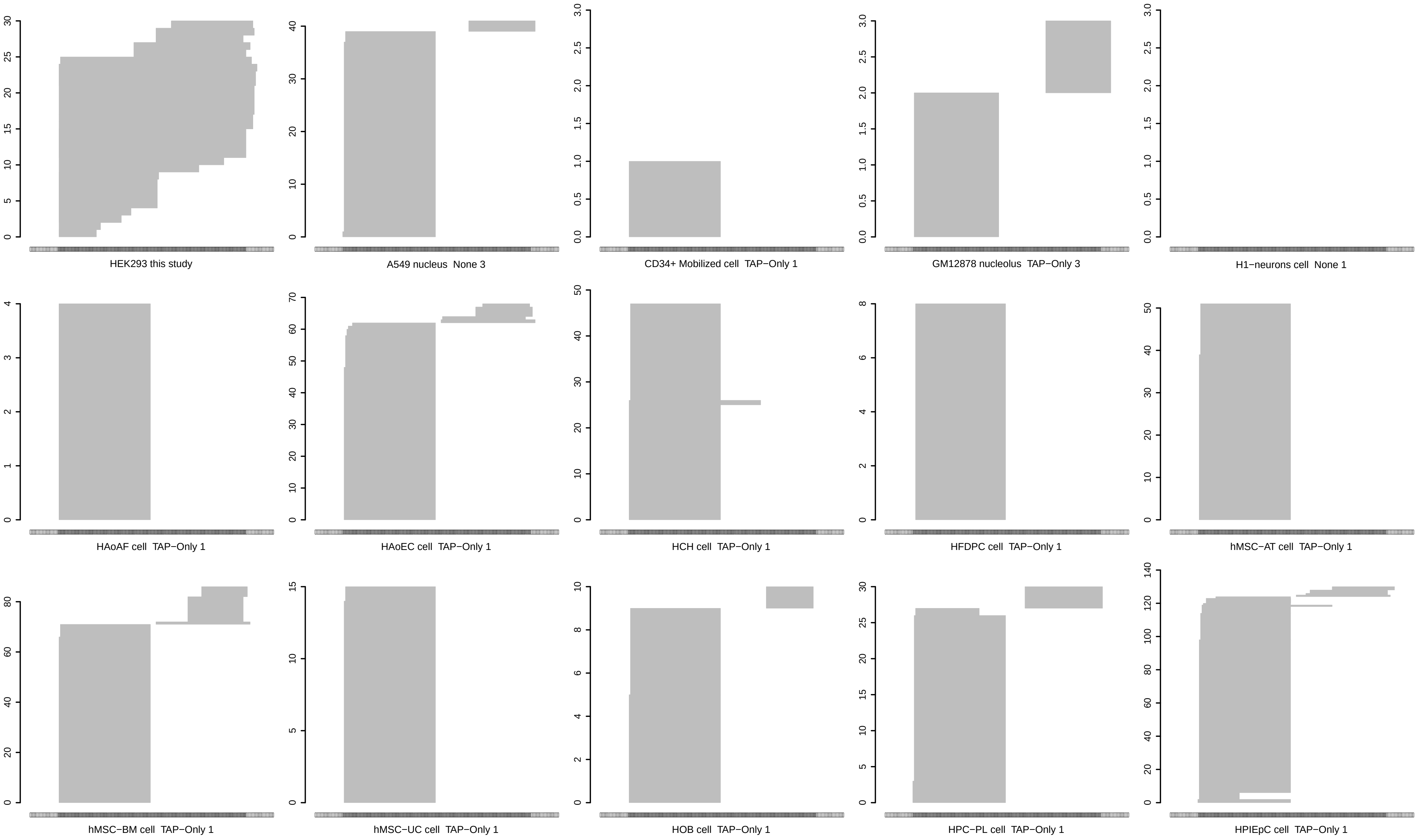

ZL142 chr12:50850353-50850570 (+)

ACAGCCCAGTGATGATCACTATTCCTACTTAGAGAGAATAGGACTAACTTTCAGAAATCCAGGCATTTTTCTACCTTTCATACTATCTTTCTTTCACTTTACTTCTCTTTTCTGTCTTTTATCACTTCTTTCTTTCTTCATTCTTTCTCTCTTTTGCCTGGATCGAGATTGTTAAGTCCCTCTCAGTGAAGGGTAA

(((((((...

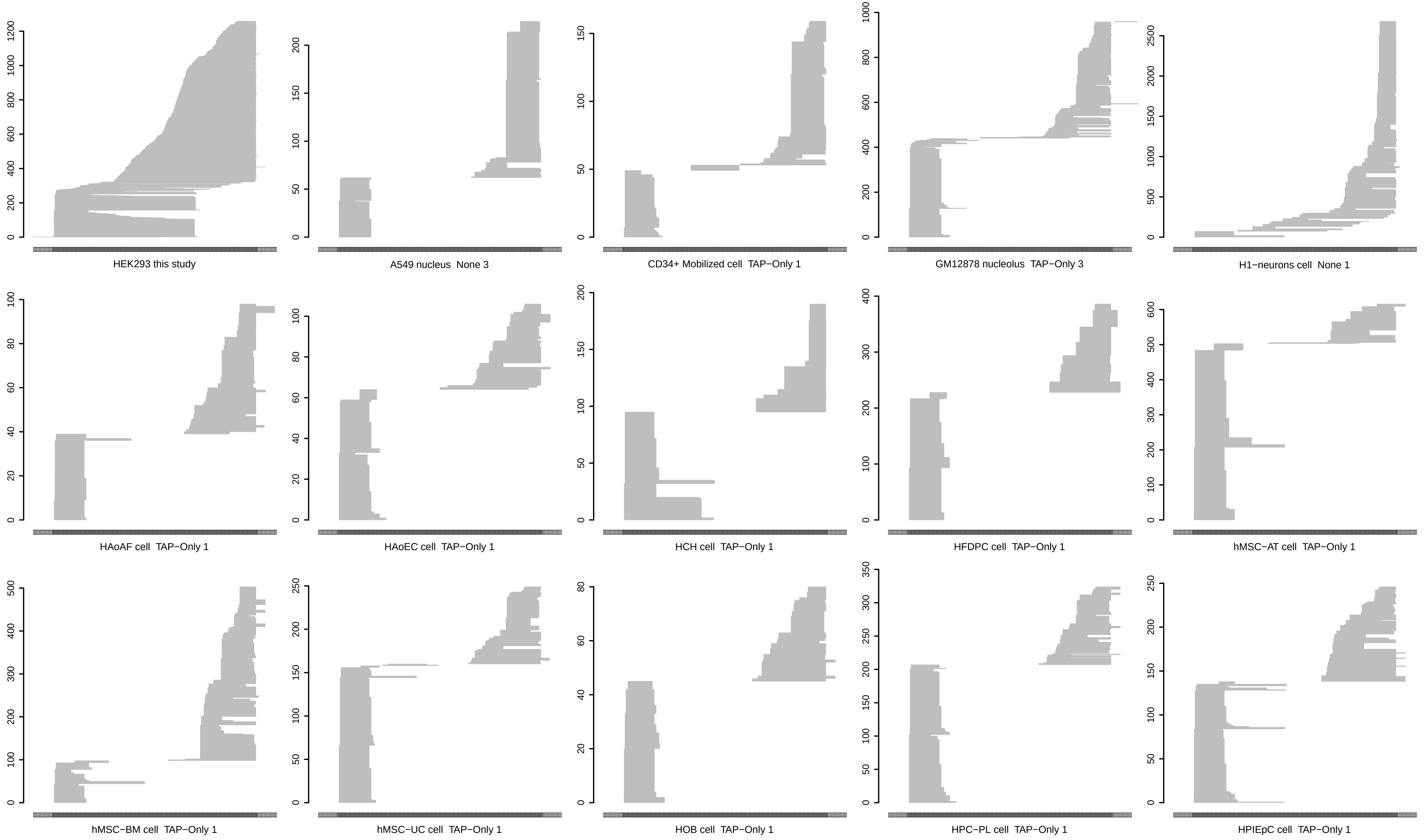

ZL143 chr7:56123060-56123195 (+)

GCACAGTGAACACCCAAGTGTGCTTTATAGTTCCCTTGGCTTTGACCCTGTGCTAGAGCATTCATTACCTGTTCATCTGCTCTGCATTGAAAGGAATATTTGTCCTTTTAAATGTATTCAGAAAGCCAGCACATTA

((((((.(..((.(((((.(.(((....))).).)))))...))..))))))).................(((....((((...(((((((((((((.......))))))).))))))..))))....)))........

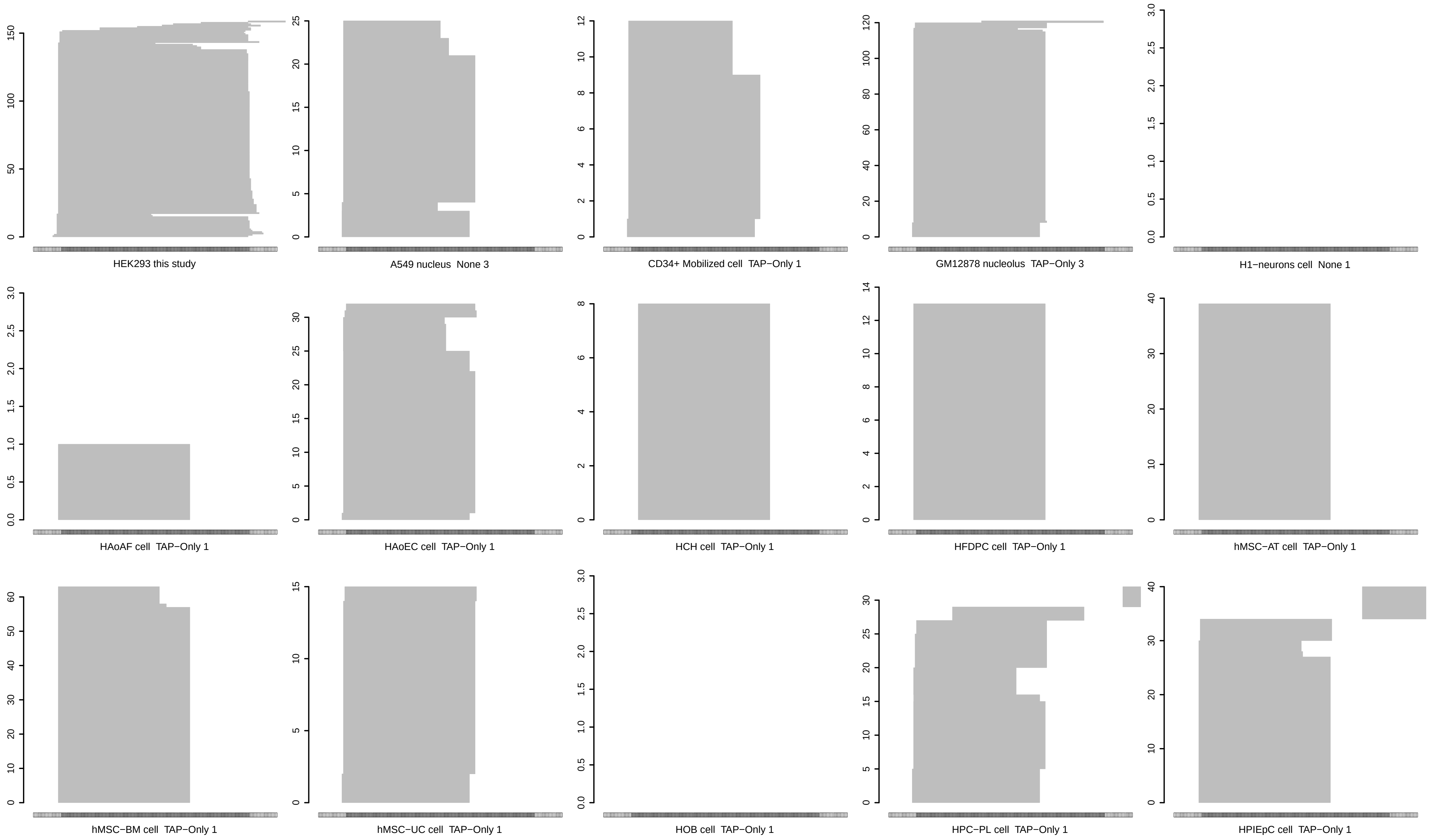

ZL144 chr12:124101257-124101387 (+)

GCAAGCCTCCAGCGTGCTTGGGTCTGCAGTGACCCCGTGGATTCCTACAGGGCTTGCCAGAACAGTTTTGAAATGGTTTGAGGCCTTGCCGTGCTCCATGTAGAGCAAGGTTATAGAAATTTCAGACAATG

((((((((((((.((.....(((((......)))))))))))).........)))))).........((((((((..((((..((((((((..(((.....)))..))))))))..)))).)))))))).....

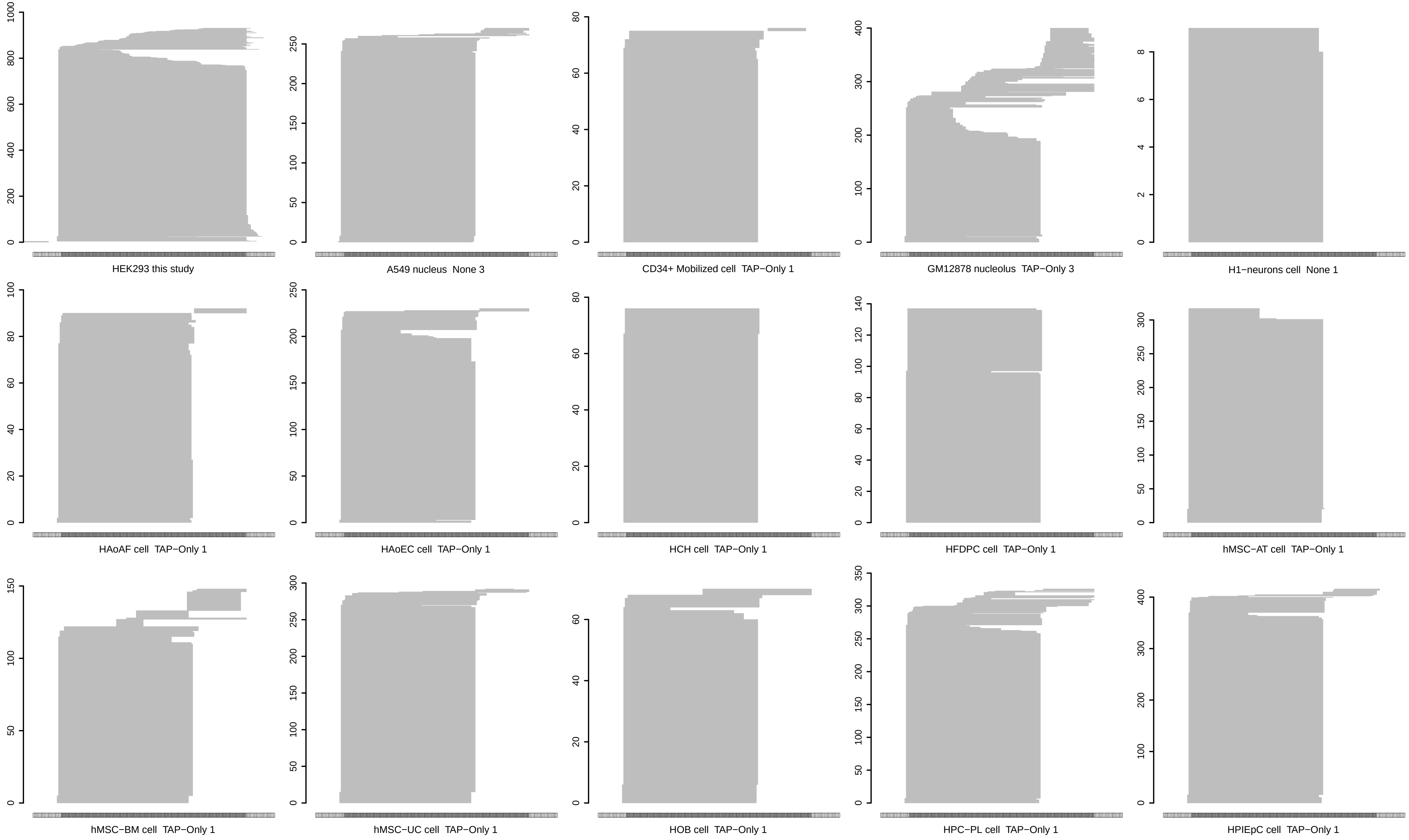

ZL146 chr9:20786924-20787054 (+)

TACTAGCATTTTCACAGGTCCTCCCTCAGGCTATGGGGCCAGGATTTGGTAGCTGGTGCTGAGAGAAAACGCTTGATTGCATCCTTGCCCTGGGACTGTGCCAGTGGCAGCTGTCACTCAATGGGACAATT

(((((((.......((((((((.(((((.....)))))..))))))))...))))))).........(.(((((.((.((..(((((((((.......)))).)))))..)))).)))).).)......

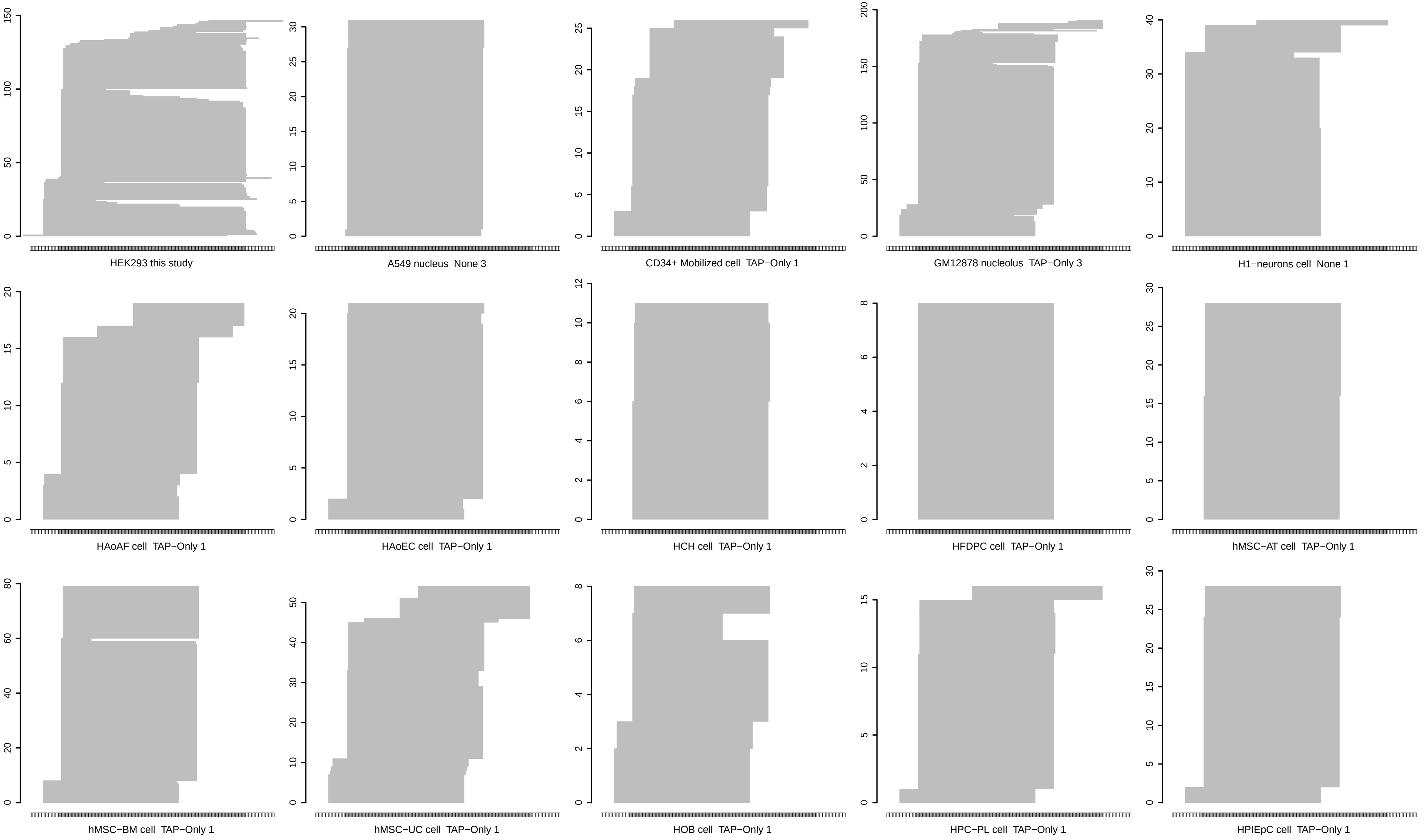

ZL147 chr22:34100772-34100906 (-)

GCACTGCTTTAGAGCCTCTGCGTGTCTGGCTGTGGCCTCAGGGGTCTAGCAGCAGTGCTAGAGCAGACCCAGCTTGTCAGATCCGGGGCTGCTTAGAGGCCACCTAAGTGATTCCTTTGGCAGCAAGCAACATTC

((((((((((((.((((((.(.(((.......)))).))))))))))).))))))))............(((.((((((...((((..((((((.(....)))))))..)))))))))))))...........

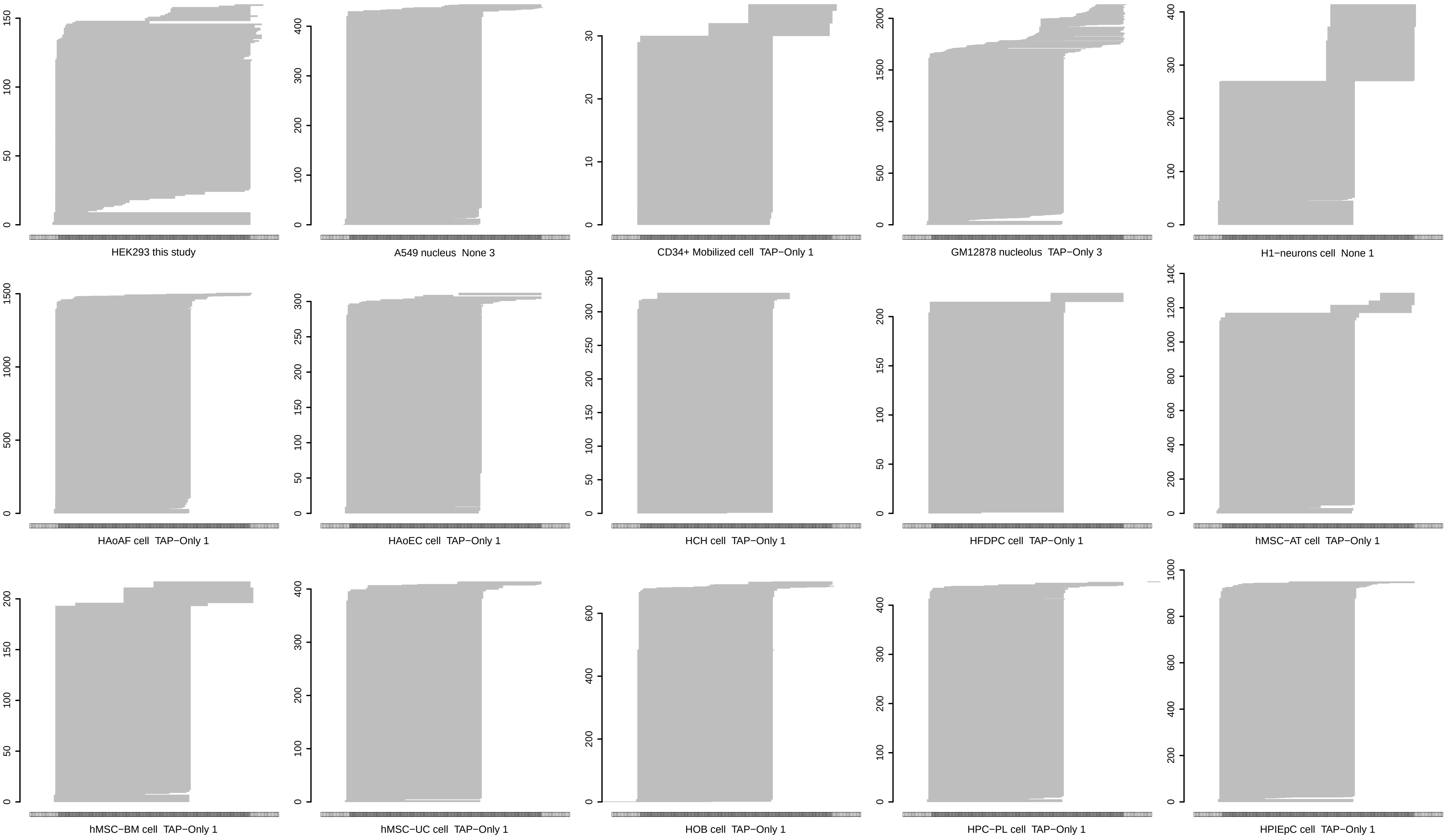

ZL149 chr22:42004850-42004874 (-)

GAGTGGGTGATGTTTGCTGACACTC

(((((...............)))))

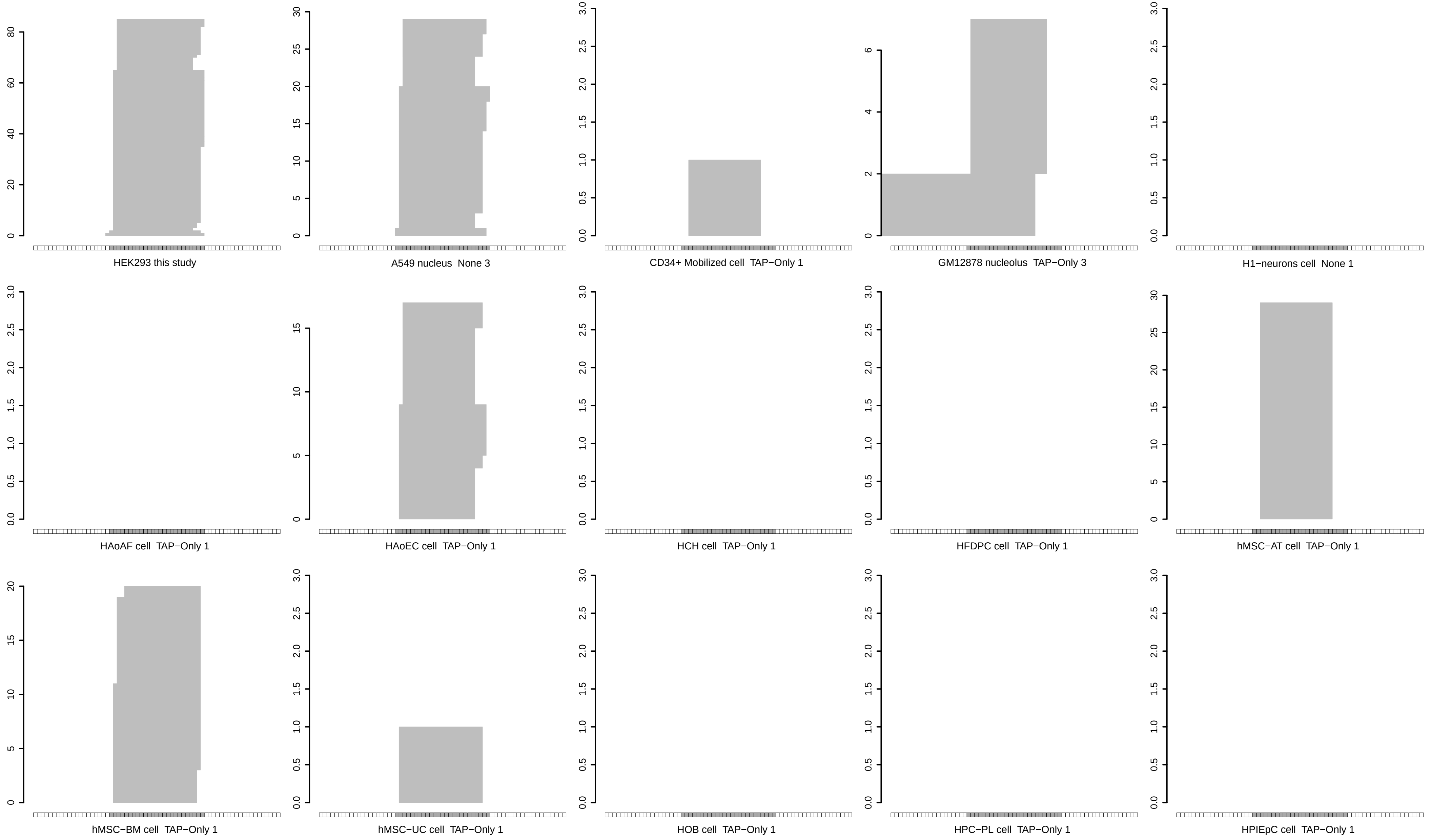

ZL150 chr19:38653734-38653791 (+)

TGGGACCATGATGATGAAGAGGACAGACCAGCCCTGTCCTCAGGAAGCTGACTTCCCA
(((((..)))))

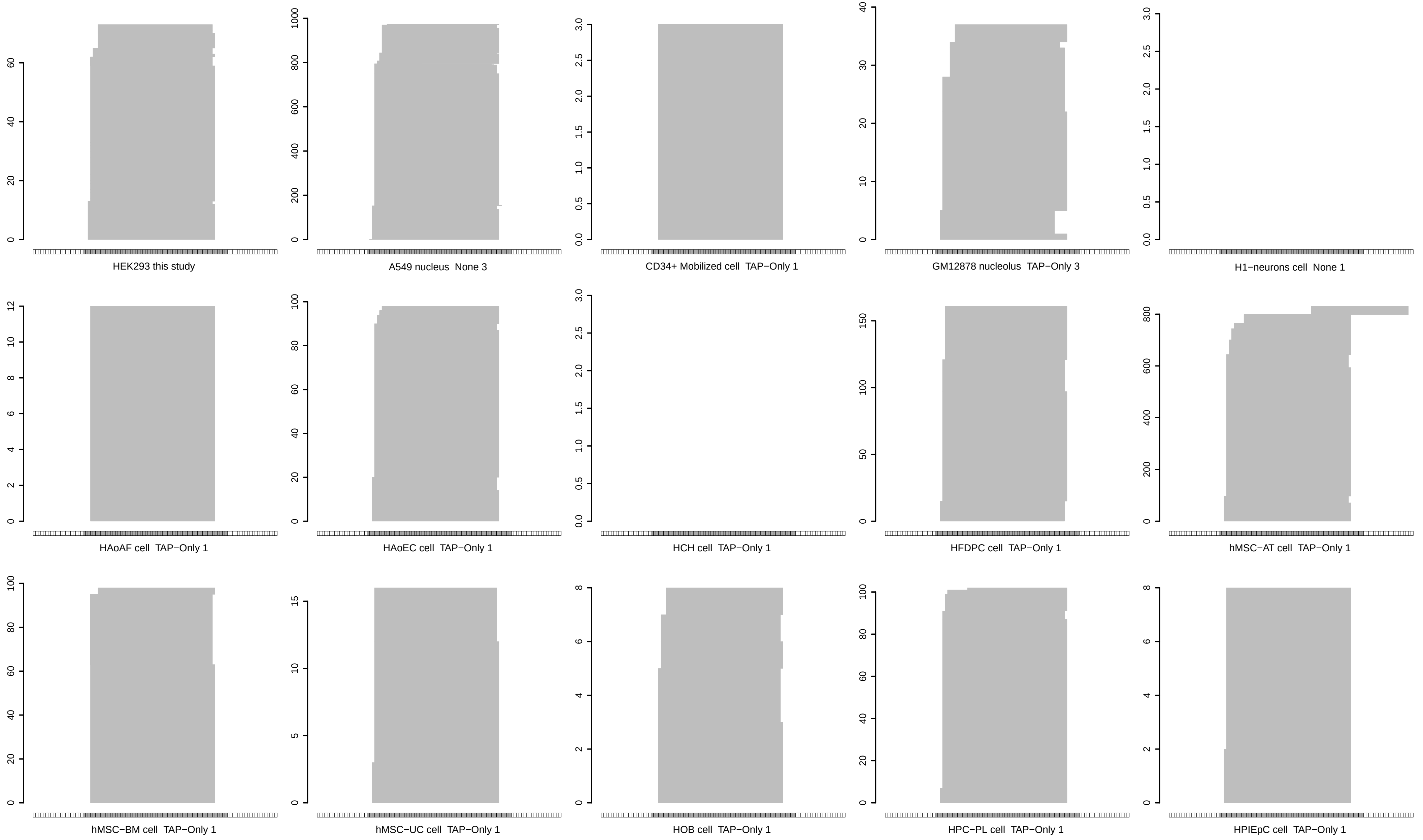

Supplement: Additional file 4 — RNA-seq read profiles from selected ENCODE small RNA-seq samples along the novel C/D box and H/ACA box snoRNA loci identified in our study. [file gb-2013-14-5-r45-S4.PDF]
