# Supplementary figures and images for: Insights into snoRNA biogenesis and processing from PAR-CLIP of snoRNA core proteins and small RNA sequencing
Source: Genome Biol. 2013 May 26;14(5):R45. doi: 10.1186/gb-2013-14-5-r45 (PMC4053766; doi:10.1186/gb-2013-14-5-r45)

20 bp ladder

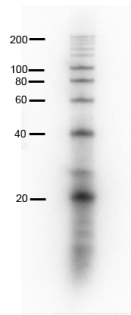

ZL1

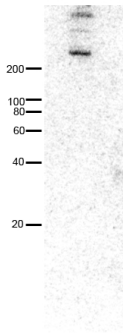

ZL2

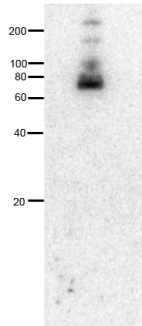

ZL8

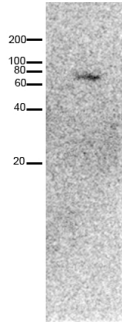

ZL63

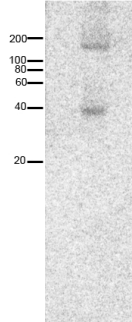

ZL107

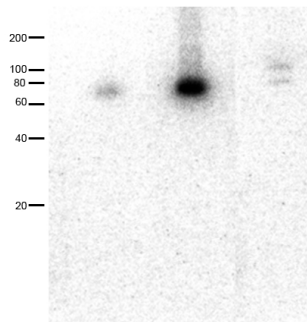

ZL126

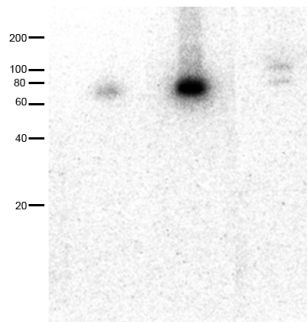

ZL11

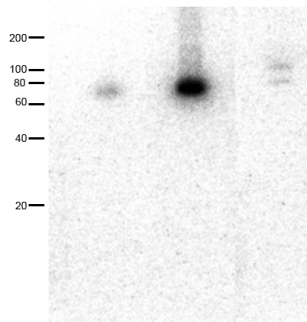

ZL116

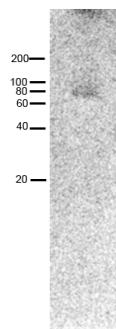

ZL127

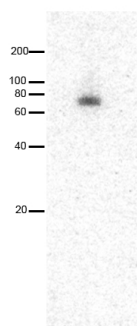

Supplement: Additional file 5 — Northern blots for selected novel C/D box snoRNAs. Among the 20 most abundantly expressed (in the small RNA-seq data) novel C/D box snoRNAs we could confirm the presence of ZL1, ZL2, ZL8, ZL11, ZL63, ZL107, ZL116, ZL126 and ZL127 by Northern blotting. [file gb-2013-14-5-r45-S5.PDF]

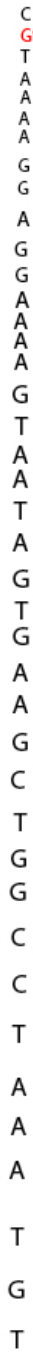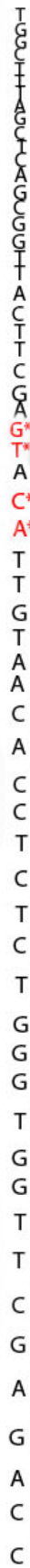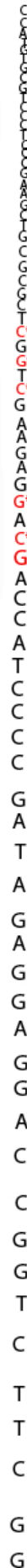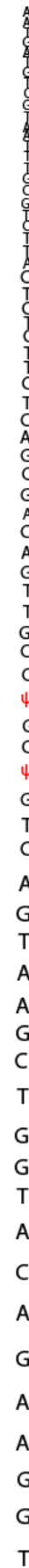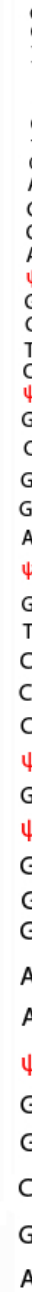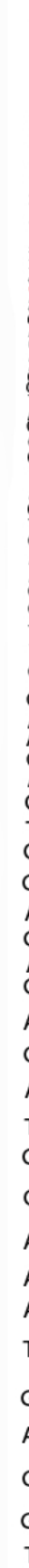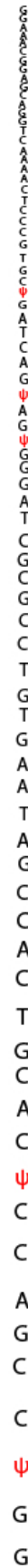

Supplement: Additional file 9 — Primer extension assays for non-canonical snoRNA targets. Primer extension runs reveal 2'-O-methyl (A-C) and pseudouridine (D-G) modification sites in several non-canonical RNAs. (A) SNORA61: G50. (B) VTRNA1-2: G30, U31, C33, A34. (C) 7SK RNA: C137, G139, C141, G148, C150, G151. (D) SNORD16: U52, U55. (E) SNORD35A: U26, U31, U37, U43, U45, U51. (F) 7SK RNA: U250. (G) 7SL RNA: U226, U233, U236, U266, U273. [file gb-2013-14-5-r45-S9.PDF]

**A**

final dNTP conc.

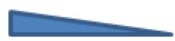

C G A T

0.5 mM  
0.3 mM  
0.03 mM  
0.003 mM

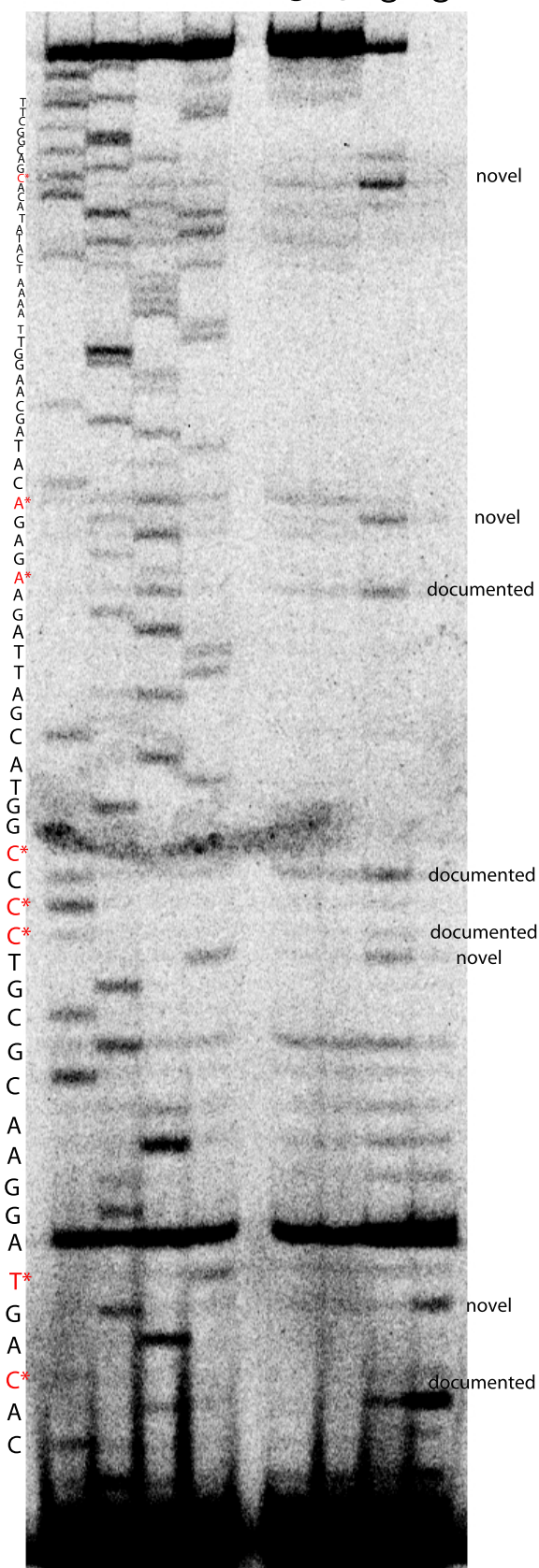

**B**

C T G A CTRL + CMC

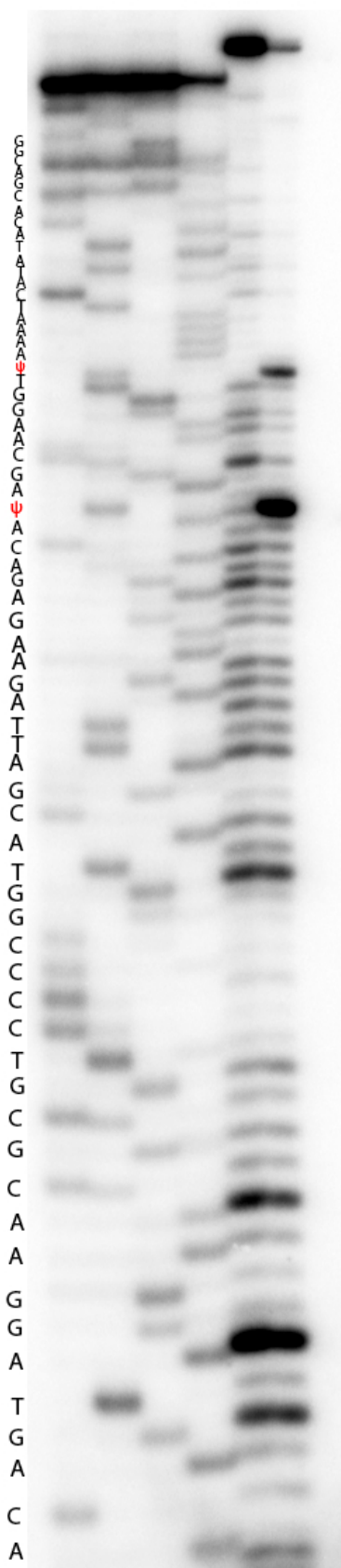

Supplement: Additional file 14 — Primer extension assays on spliceosomal RNA U6. (A) Primer extension assay on spliceosomal RNA U6 detected documented 2'-O-methylation as well as potentially novel 2'-O-methylation sites. (B) Primer extension assay detected documented pseudouridine sites in U6. CTRL indicates the untreated sample, +CMC the sample treated with 1-cyclohexyl-3-(2-morpholinoethyl)carbodiimide metho-p-toluenesulfonate (CMC). [file gb-2013-14-5-r45-S14.PDF]

**A**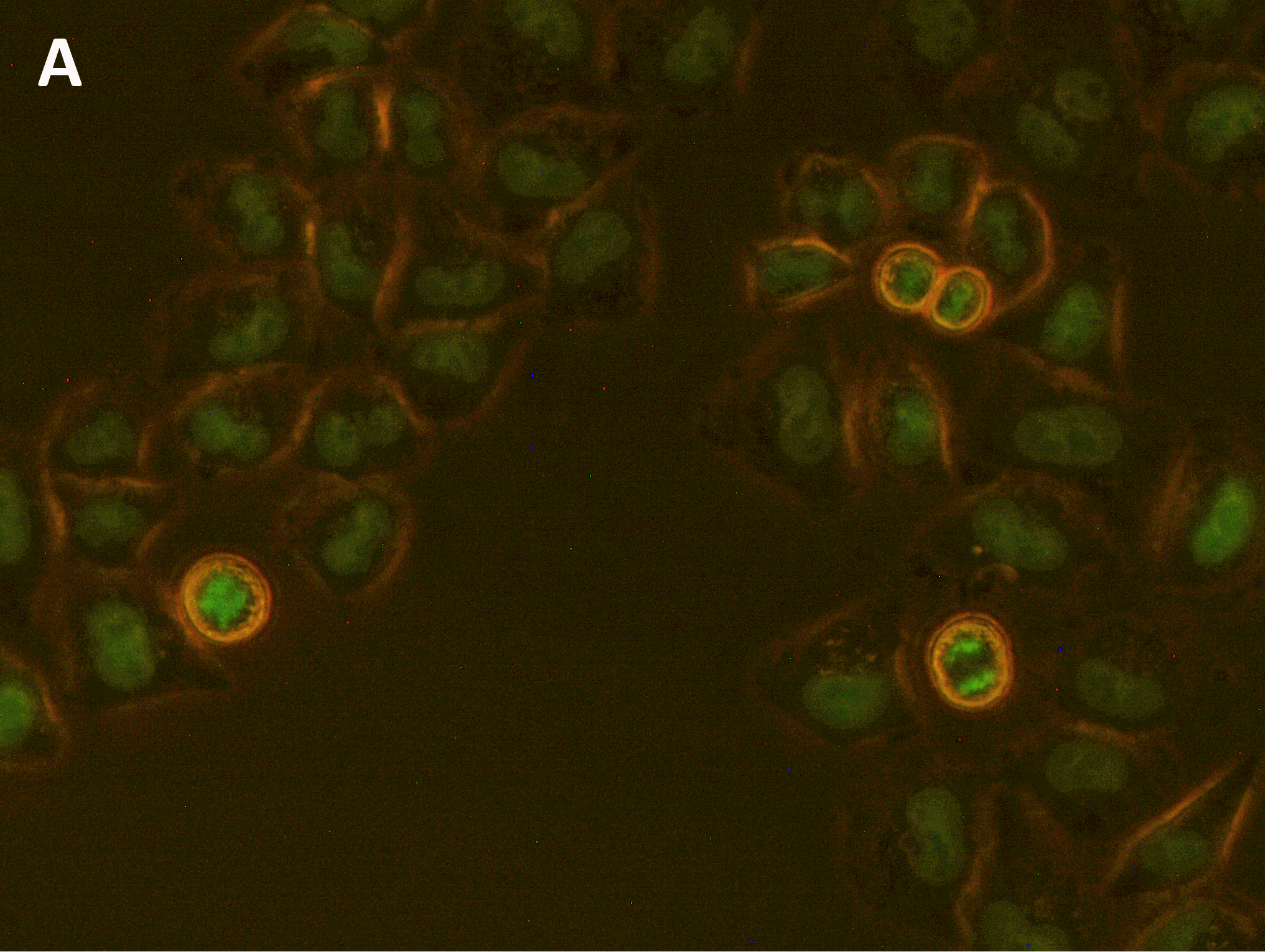**B**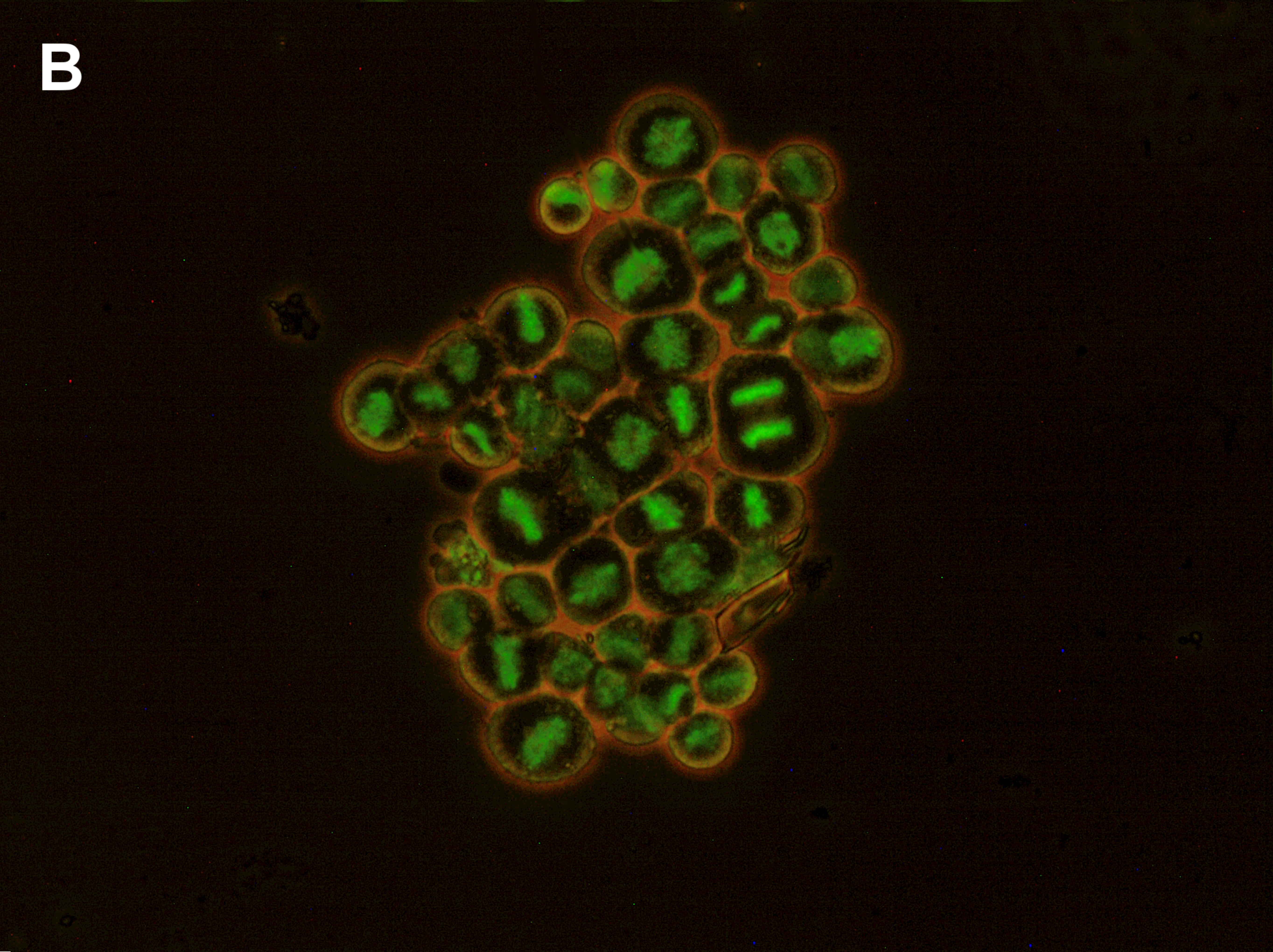

Supplement: Additional file 15 — Asynchronous and mitotic GFP-tagged HeLa cells. Green fluorescent protein appears in green and cell boundaries in orange. (A) In an asynchronous cell culture only a few cells are in the mitotic phase, which can be seen from the condensed chromatin and the rounded cell morphology. (B) Cell obtained with mitotic shake-off. The procedure enriches for round cells containing condensed chromatin. [file gb-2013-14-5-r45-S15.PDF]

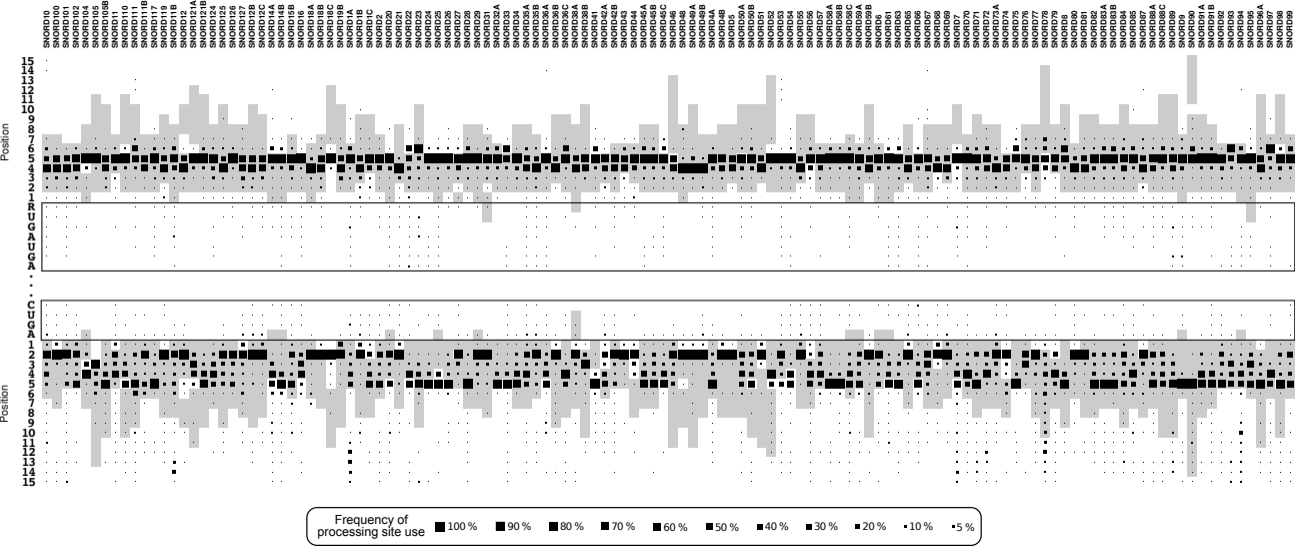

Supplement: Additional file 16 — Extended version of Figure 4B showing all snoRNA genes expressed in HEK293 cells. [file gb-2013-14-5-r45-S16.PDF]
