# Supplementary material for: Insights into snoRNA biogenesis and processing from PAR-CLIP of snoRNA core proteins and small RNA sequencing
Source: Genome Biol. 2013 May 26;14(5):R45. doi: 10.1186/gb-2013-14-5-r45 (PMC4053766; doi:10.1186/gb-2013-14-5-r45)
Supplement: Additional file 6 — Expression of C/D box and C/D box-like snoRNAs in our small RNA-seq run (20 to 200 nucleotides; sequenced 150 cycles). Only reads that cover at least 50% of the snoRNA locus were considered. [file gb-2013-14-5-r45-S6.PDF]

Expression of C/D box and C/D box-like snoRNAs in small RNA-seq (20-200 nt; sequenced 150 cycles). Only reads that cover at least 50% of the snoRNA locus were considered.

ID	TPM/nuc	ID	TPM/nuc	ID	TPM/nuc	ID	TPM/nuc
SNORD18B	15010.62	SNORD61	1832.68	SNORD90	278.54	ZL17	8.47
SNORD43	12622.48	SNORD38B	1823.2	SNORD1B	274.03	ZL19	8
SNORD2	10171.89	SNORD33	1792.01	ZL109	269.4	ZL98	7.93
SNORD58A	10135.86	SNORD58C	1640.24	SNORD35B	234.92	ZL119	7.39
SNORD18C	9088.05	SNORD12C	1522.89	SNORD72	227.05	ZL35	6.45
SNORD58B	8955.57	SNORD60	1443.84	SNORD66	224.63	ZL22	6.32
SNORD29	8799.9	SNORD59B	1401.9	SNORD105	224.56	ZL77	5.91
SNORD18A	8595.1	SNORD118	1376.49	ZL6	209.58	ZL149	5.71
SNORD44	8546.17	SNORD88A	1343.56	SNORD93	208.57	ZL69	5.65
SNORD32A	7231.91	ZL127	1337.17	SNORD14A	195.26	ZL45	5.44
SNORD27	7024.15	ZL2	1323.8	SNORD77	185.65	ZL64	5.31
SNORD4B	6951.29	SNORD63	1300.2	ZL5	169.38	ZL47	5.11
SNORD26	6831.58	SNORD1C	1285.01	SNORD9	160.91	ZL84	4.91
SNORD49A	6660.99	SNORD57	1278.09	SNORD53	154.06	ZL40	4.91
SNORD75	6556.2	SNORD50B	1162.21	SNORD1A	140.14	ZL150	4.91
SNORD104	6189.41	SNORD10	1147.96	SNORD125	136.98	ZL99	4.71
SNORD68	5935.74	SNORD56	1085.65	SNORD86	114.8	ZL37	4.71
SNORD46	5666.41	SNORD59A	1069.46	SNORD22	114.6	ZL41	4.5
SNORD88C	5374.84	SNORD38A	1040.82	SNORD15A	109.56	ZL78	4.37
SNORD100	5178.77	SNORD87	1016.76	ZL27	107.75	ZL46	3.97
SNORD5	5051.67	SNORD99	976.16	SNORD36A	103.85	ZL48	3.83
SNORD28	4793.63	SNORD119	970.78	ZL9	101.9	ZL65	3.7
SNORD52	4770.98	SNORD98	961.31	ZL1	96.86	ZL71	3.7
SNORD102	4653.28	SNORD51	956.27	SNORD88B	90.87	SNORD96B	3.56
SNORD30	4585.67	SNORD127	948.94	SNORD54	84.62	ZL89	3.43
SNORD81	4564.96	SNORD4A	945.71	SNORD67	84.49	ZL43	3.16
SNORD48	4438.33	SNORD37	936.17	SNORD97	76.62	ZL51	2.76
SNORD76	4189.03	SNORD79	838.91	SNORD111	71.45	ZL79	2.69
SNORD55	4097.95	ZL11	825.2	SNORD85	64.19	ZL125	2.62
SNORD80	3874.47	SNORD36B	741.51	SNORD11B	51.89	SNORD62A	2.49
SNORD19B	3676.58	SNORD101	713.89	ZL63	49.34	ZL81	2.49
SNORD31	3530.26	SNORD16	707.71	ZL8	40.73	ZL52	2.49
SNORD12B	3504.72	SNORD17	701.19	ZL7	37.77	ZL50	2.35
SNORD117	3442.74	SNORD111B	697.62	ZL75	34.75	ZL68	2.29
SNORD82	3146.6	SNORD126	672.55	SNORD94	34.35	ZL62	1.95
SNORD96A	3146.13	SNORD92	670.4	SNORD7	31.86	ZL53	1.88
SNORD25	3100.76	SNORD8	668.65	ZL142	31.79	SNORD62B	1.88
SNORD69	3065.53	SNORD41	663.81	ZL116	29.71	ZL72	1.88
SNORD65	3065.13	SNORD91A	644.72	SNORD89	27.63	ZL118	1.75
SNORD45A	3061.64	SNORD36C	618.58	SNORD73B	25.21	ZL122	1.61
SNORD24	2872.02	SNORD49B	612.8	ZL92	25.14	ZL60	1.61
SNORD83A	2811.33	SNORD13	578.18	ZL30	20.63	ZL73	1.48
SNORD45C	2775.3	SNORD11	566.42	ZL31	20.5	ZL54	1.48
SNORD50A	2742.3	SNORD15B	521.59	ZL120	18.89	ZL101	1.41
SNORD42A	2720.86	SNORD20	444.83	ZL23	18.08	ZL56	1.21
SNORD6	2385.66	SNORD121A	437.43	ZL12	16.47	ZL114	1.01
SNORD21	2341.9	SNORD73A	436.56	SNORD23	15.39	SNORD103A	0
SNORD34	2262.12	SNORD78	431.32	ZL49	13.31	SNORD108	0
SNORD110	2218.29	SNORD45B	430.85	ZL13	12.5	SNORD109B	0
SNORD95	2109.54	SNORD124	409.2	ZL18	12.1	SNORD64	0
SNORD12	2069.75	SNORD84	400.8	ZL24	11.56	SNORD32B	0
ZL107	2058.59	SNORD70	387.76	ZL76	10.82	SNORD112	0
ZL126	2036.14	SNORD35A	371.09	ZL34	10.69	SNORD103B	0
SNORD47	1994.4	SNORD91B	352.07	ZL25	9.81	SNORD123	0
SNORD83B	1989.09	SNORD71	328.61	ZL121	9.28	SNORD19	0
SNORD105B	1929.67	SNORD14B	328.55	ZL104	9.28	SNORD107	0
SNORD74	1845.25	SNORD121B	315.84	ZL103	9.21	SNORD109A	0
SNORD42B	1836.85	ZL132	313.76	ZL102	8.94		
