# Supplementary material for: Insights into snoRNA biogenesis and processing from PAR-CLIP of snoRNA core proteins and small RNA sequencing
Source: Genome Biol. 2013 May 26;14(5):R45. doi: 10.1186/gb-2013-14-5-r45 (PMC4053766; doi:10.1186/gb-2013-14-5-r45)
Supplement: Additional file 7 — SCARNA21 has a C/D box H/ACA box hybrid structure. (A) Screenshot from the UCSC genome browser showing conserved C and D box elements. (B) Northern blot probing for H/ACA box structure only (left) and for the hybrid structure (right). [file gb-2013-14-5-r45-S7.PDF]

A

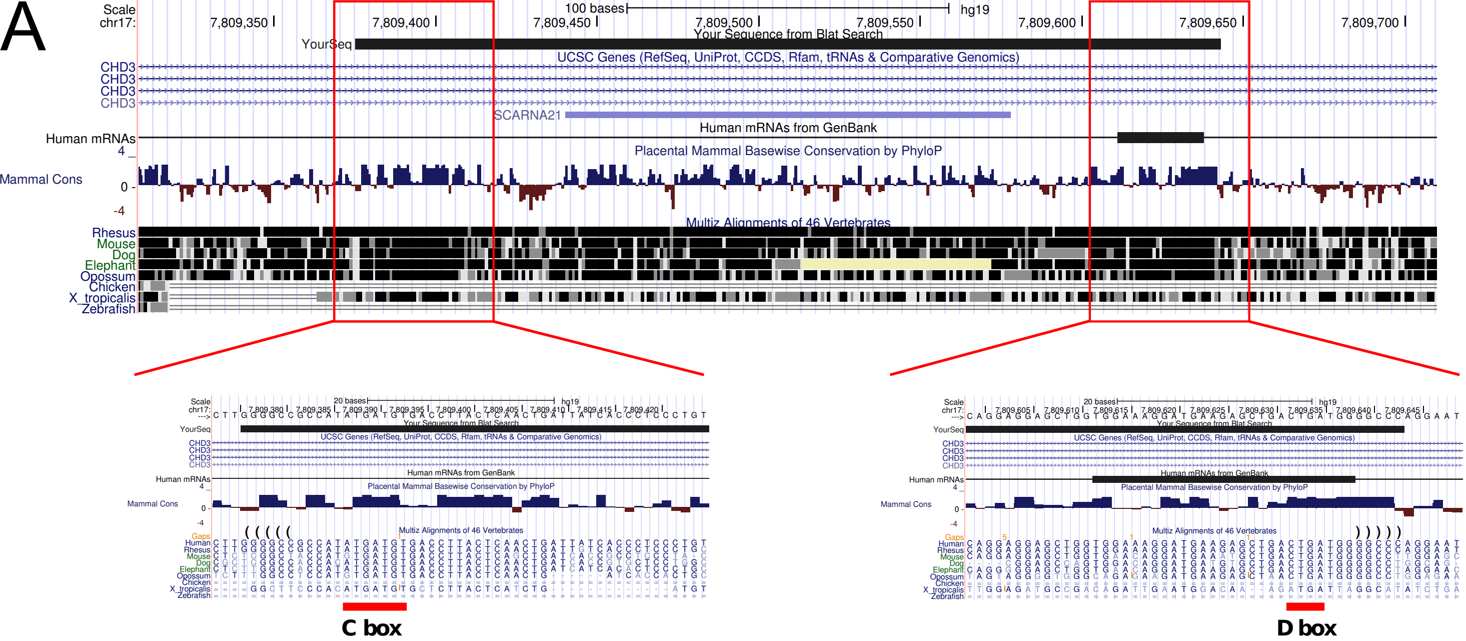

B

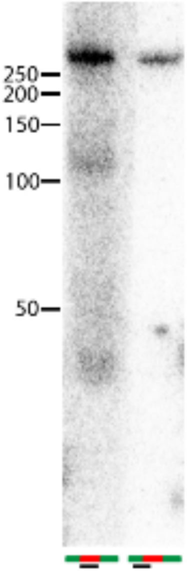

Scarna21 138 bp

Scarna21 embedded in C/D box snoRNA: 268 bp

GGGGCCGCCATATGATGTGACCTTACTCAACTGATTATCACCCTCCCTGTCATACAATACTTCCTGGCTCGATTTCCTGGGGGGT
GGTCTCAGCCCACTCCACCTCCCCTCAGCCGAGCCTAGAGTAGAGGGGCCAGGCATCCTCCCCAGGGGAGGGGCGTTGAAGCA
AGGAGCCTCTCCTGGGCTGTCCTAGCCTCACATTTACTTGACCACAATAACCTGGCAGGAGGAGCTGGTGGAAAGGATGAAGA
GCTGACTGATGGGGCCC
