# Supplementary material for: Insights into snoRNA biogenesis and processing from PAR-CLIP of snoRNA core proteins and small RNA sequencing
Source: Genome Biol. 2013 May 26;14(5):R45. doi: 10.1186/gb-2013-14-5-r45 (PMC4053766; doi:10.1186/gb-2013-14-5-r45)
Supplement: Additional file 8 — Primer extension assays for U2 snRNA. Primer extension assay reveals a 2'-O-methyl modification site for nucleotide U47. [file gb-2013-14-5-r45-S8.PDF]

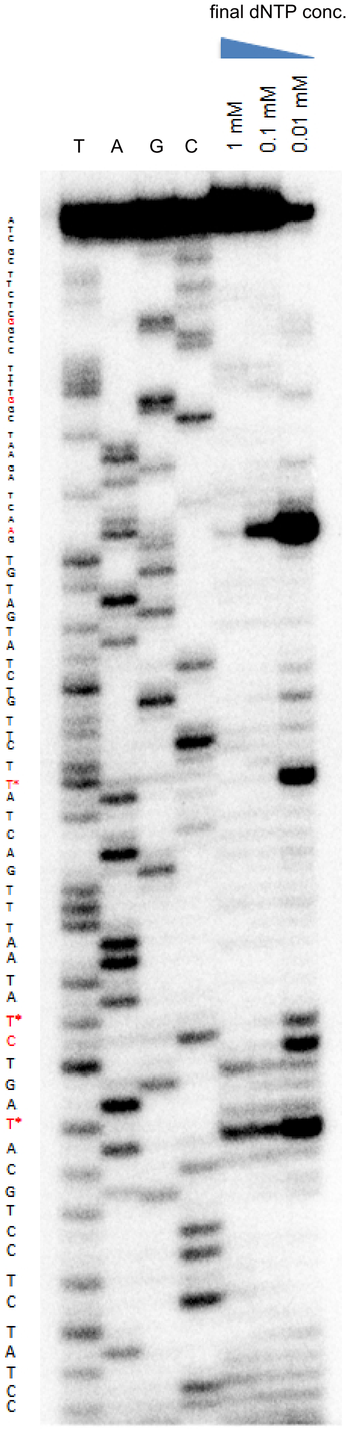
final dNTP conc.
1 mM
0.1 mM
0.01 mM
T
A
G
C
T G T A G T A T C T G T T C T T* A T C A G T T T A A T A T* C T G A T* A C G T C C T C T A T C C
