# Supplementary material for: Insights into snoRNA biogenesis and processing from PAR-CLIP of snoRNA core proteins and small RNA sequencing
Source: Genome Biol. 2013 May 26;14(5):R45. doi: 10.1186/gb-2013-14-5-r45 (PMC4053766; doi:10.1186/gb-2013-14-5-r45)
Supplement: Additional file 10 — Summary of nucleotide modifications detected by primer extension assays and predicted guide snoRNA-target interactions. [file gb-2013-14-5-r45-S10.PDF]

SNORA61

1 AUCCUCCUGAUCCCUUUCCCAUCGGAUCUGAACACUGGUCUUGGUGGUCGUAAAAGGAGGAAAAGUAAUAGUGAAGCUGG
(m at position 50)

D' box GUAUCACUU ZL121

81 CCUAAAUGUUGUAAUCUGGUAUAUGGCAUGUGGGCUAGUUUCAGACAGGU

D' box UACCGUACA SNORD95

SNORD16

1 UUGCAAUGAUGUCGUAAUUUGCGUCUUACUCUGUUCUCAGCGACAGUUGCCψGCψGUCAGUAAGCUGGUACAGAAGGUUG

81 ACGAAAAUUCUUACUGAGCAA

SNORD35A

1 GGCAGAUGAUGUCCUUAUCUCACGAψGGUCψGCGGAψGUCCCψGψGGGAAψGGCGACAAUGCCAAUGGCUUAGCUGAUGC

81 CAGGAG

VTRNA1-2

1 GGCUGGCUUUAGCUCAGCGGUUACUUCGAGUACAUUGUAACCACCUCUCUGGGUGGUUCGAGACCCGCGGGUGCUUUCCA
(m m under GU, m m under CA)

81 GCUCUUUU

RN7SK

1 GGAUGUGAGGGCGAUCUGGCUGCGACAUCUGUCACCCCAUUGAUCGCCAGGGUUGAUUCGGCUGAUCUGGCUGGCUAGGC

81 GGGUGUCCCCUUCCUCCCUCACCGCUCCAUGUGCGUCCCUCCCGAAGCUGCGCGCUCGGUCGAAGAGGACGACCAUCCCC
(m m m m m m)

161 GAUAGAGGAGGACCGGUCUUCGGUCAAGGGUAUACGAGUAGCUGCGCUCCCCUGCUAGAACCUCCAAACAAGCUCUCAAG

D box AGUUCCCA SNORD84 — D' box UGUUC

241 GUCCAUUUGψAGGAGAACGUAGGGUAGUCAAGCUUCCAAGACUCCAGACACAUCCAAAUGAGGCGCUGCAUGUGGCAGUC

CAGG SNORD107 — D' box AAGGUUCU SNORD78 — D box UGUAGGUU SNORD14A/B

321 UGCCUUUCUUUU

UCAAGGUCCAUUUGψA GGAG
GGUUCCAGGGAAUC UUCCUU SNORA70 2nd stem

RN7SL

1 GCCGGGCGCGGUGGCGCGUGCCUGUAGUCCCAGCUACUCGGGAGGCUGAGGCUGGAGGAUCGCUUGAGUCCAGGAGUUCU

81 GGGCUGUAGUGCGCUAUGCCGAUCGGGUGUCCGCACUAAGUUCGGCAUCAAUAUGGUGACCUCCCGGGAGCGGGGGACCA

161 CCAGGUUGCCUAAGGAGGGGUGAACCGGCCCAGGUCGGAAACGGAGCAGGUCAAAACUCCCGUGCψGAUCAGψAGψGGGA

CACG UAGUCA SNORA31 1st stem

241 UCGCGCCUGUGAAUAGCCACUGCACψCCAGCCψGGGCAACAUAGCGAGACCCCGUCUCU
