# Supplementary material for: Insights into snoRNA biogenesis and processing from PAR-CLIP of snoRNA core proteins and small RNA sequencing
Source: Genome Biol. 2013 May 26;14(5):R45. doi: 10.1186/gb-2013-14-5-r45 (PMC4053766; doi:10.1186/gb-2013-14-5-r45)
Supplement: Additional file 11 — Analysis of PAR-CLIP clusters overlapping with mRNA exon annotation. Shown are genome coordinates, host transcript and exon identifier, the number of C and D boxes predicted within the genomic region, snoRNAs to whose guide regions these mRNA fragments are complementary and the number of (normalized) reads obtained from the regions in various PAR-CLIP libraries. [file gb-2013-14-5-r45-S11.PDF]

Supplementary Table. Analysis of PAR-CLIP clusters overlapping with mRNA exon annotation.

	Location	mRNA annotation	Occurance C box motif	Occurences D box motif	8-mer complementarity to guide regions	NOP58 rep A	NOP58 rep B	NOP56	FBL	FBL (MNase)
1	chr14:102549987-102550038 (-)	HSP90AA1_exon4	0	0		132.0	126.7	0.0	67.0	0.0
2	chr19:48893690-48893732 (-)	KDELR1_exon4	0	0		131.5	132.5	21.4	43.6	0.0
3	chr1:62907905-62907969 (+)	USP1_exon4	1	1		79.4	34.5	0.0	11.3	0.0
4	chr1:35654622-35654691 (-)	SFPQ_exon5	1	2		71.9	77.8	0.0	20.5	0.0
5	chr19:3981340-3981440 (-)	EEF2_exon9	1	2	SNORD94,SNORD12	68.7	75.0	0.0	17.4	15.6
6	chr17:76219547-76219597 (+)	BIRC5_exon4|BIRC5_exon3|BIRC5_exon5	0	0	SNORD16,SNORD125	66.0	71.8	0.0	26.6	0.0
7	chr1:236757326-236757419 (-)	HEATR1_exon37	0	2	SNORD17	64.5	31.6	0.0	25.1	0.0
8	chr19:10795159-10795209 (+)	ILF3_exon18	0	0	SNORD111B	60.1	42.5	25.6	37.8	0.0
9	chr11:122928994-122929106 (-)	HSPA8_exon2	3	3	SNORD103B,SNORD103A,SNORD70,SNORD14A,SNORD14B	58.4	48.7	0.0	29.0	0.0
10	chr2:232325381-232325590 (-)	NCL_exon11	20	15	SNORD125	58.2	61.0	0.0	18.5	18.7
11	chr11:77336018-77336074 (-)	CLNS1A_exon4	3	4	SNORD63	54.3	61.5	0.0	16.6	0.0
12	chr5:170832287-170832350 (+)	NPM1_exon9|NPM1_exon8	0	0		50.6	34.3	0.0	15.3	0.0
13	chr17:38563806-38563882 (-)	TOP2A_exon23	6	4	SNORD42A	50.1	92.9	0.0	42.9	0.0
14	chr17:37360800-37360915 (+)	RPL19_exon6	1	0	SNORD36B,SNORD111B,SNORD111	49.3	62.0	0.0	19.7	0.0
15	chr6:30548207-30548267 (+)	ABCF1_exon8	1	1	SNORD125	49.0	30.0	0.0	12.3	0.0
16	chr1:35653596-35653705 (-)	SFPQ_exon4	2	3		46.6	34.1	0.0	33.8	0.0
17	chr1:214791974-214792035 (+)	CENPF_exon4	0	0		44.1	33.2	0.0	24.6	0.0
18	chr14:90871039-90871128 (+)	CALM1_exon6	2	3		43.9	27.9	0.0	19.9	0.0
19	chr1:153634617-153634662 (-)	ILF2_exon1	0	0		42.5	24.9	19.5	15.9	0.0
20	chr22:43570504-43570555 (-)	TTLL12_exon8	0	0	SNORD125	41.0	46.4	0.0	15.6	0.0
21	chr17:59934443-59934517 (-)	BRIP1_exon17	0	3		40.9	25.0	0.0	19.5	0.0
22	chr8:117861195-117861258 (-)	RAD21_exon2	0	0		40.7	31.0	0.0	11.4	0.0
23	chr3:88190187-88190228 (-)	CGGBP1_exon3	0	0		40.2	24.0	0.0	11.7	0.0
24	chr4:174253173-174253379 (-)	HMGB2_exon1	11	6	SNORD59B,SNORD5	39.7	85.0	0.0	23.1	0.0

					9A,SNORD125					
25	chr21:47771349-47771396 (+)	PCNT_exon9	1	2		39.5	23.2	0.0	18.9	0.0
26	chr5:56509977-56510024 (+)	GPBP1_exon1| GPBP1_exon2| GPBP1_exon3	0	0		39.3	44.6	15.5	10.8	0.0
27	chr22:41645756-41645810 (-)	RANGAP1_exon3	0	0		38.6	35.9	0.0	20.0	0.0
28	chr14:105927102-105927176 (+)	MTA1_exon10	0	0	SNORD6,SNORD55	38.4	64.5	0.0	15.9	0.0
29	chr1:70705122-70705170 (+)	SRSF11_exon8	0	0	SNORD125	37.9	39.3	0.0	13.3	0.0
30	chr6:31635639-31635691 (+)	CSNK2B_exon3	1	1		36.2	37.6	0.0	20.9	0.0
31	chr9:91926200-91926269 (+)	CKS2_exon1	0	0		35.9	32.4	0.0	19.7	0.0
32	chr17:30320264-30320322 (+)	SUZ12_exon11	0	1		35.9	26.2	0.0	10.2	0.0
33	chr2:201434462-201434511 (+)	SGOL2_exon6	0	1		35.8	11.7	0.0	16.8	0.0
34	chr16:89629292-89629353 (+)	RPL13_exon7| RPL13_exon6| RPL13_exon5	0	1	SNORD16	35.5	43.1	0.0	10.9	19.1
35	chr14:102550944-102551373 (-)	HSP90AA1_exon7	8	9	SNORD125,SNORD36<br>C	34.8	48.3	0.0	0.0	10.1
36	chr17:7414740-7414801 (+)	POLR2A_exon24	0	0	SNORD125	34.0	26.1	0.0	13.2	0.0
37	chr14:61278710-61278807 (+)	MNAT1_exon5	0	1	SNORD125	33.5	27.0	0.0	11.3	0.0
38	chr5:150110619-150110674 (-)	DCTN4_exon7	0	2		33.1	20.7	0.0	13.5	0.0
39	chr10:96109908-96109947 (-)	NOC3L_exon13	0	0		33.1	26.5	0.0	25.7	0.0
40	chr2:61647888-61647943 (-)	USP34_exon79	0	0		32.5	24.8	0.0	28.6	0.0
41	chr17:37360353-37360405 (+)	RPL19_exon5	0	0		32.5	20.2	0.0	28.9	0.0
42	chr20:5099392-5099570 (-)	PCNA_exon5	1	1	SNORD126	32.4	46.7	0.0	41.4	0.0
43	chr1:163317589-163317632 (+)	NUF2_exon12	0	2	SNORD36C	31.8	12.7	0.0	12.5	0.0
44	chr17:3627404-3627432 (+)	GSG2_exon1	0	1		31.5	36.8	38.6	32.6	0.0
45	chr5:134164324-134164396 (+)	DDX46_exon23	0	0		31.3	21.3	0.0	32.6	0.0
46	chr19:34712339-34712486 (+)	LSM14A_exon9	2	5	SNORD42B,SNORD7<br>2,SNORD36C	31.2	66.8	0.0	38.6	0.0
47	chr5:138643919-138643967 (+)	MATR3_exon4| MATR3_exon5| MATR3_exon2	0	0	SNORD76	30.1	44.8	0.0	31.0	0.0
48	chr15:80181579-80181643 (-)	MTHFS_exon2|ST20-<br>MTHFS_exon2	1	1	SNORD4A	30.1	25.7	0.0	11.7	0.0

49	chr2:170667449-170667543 (+)	SSB_exon10	0	1	SNORD36C	29.9	32.8	0.0	10.9	0.0
50	chr2:38975222-38975292 (-)	SRSF7_exon4|SRSF7_exon3	0	0		29.2	27.2	0.0	0.0	13.8
51	chr4:57865806-57865861 (+)	POLR2B_exon7	0	0		29.0	33.3	0.0	17.7	0.0
52	chr6:49440521-49440571 (+)	CENPQ_exon5	0	1	SNORD36C,SNORD125	28.9	18.9	0.0	12.6	0.0
53	chr17:80529687-80529737 (+)	FOXK2_exon4	0	0		28.7	10.5	15.9	18.1	0.0
54	chr14:55642649-55642704 (-)	DLGAP5_exon11|DLGAP5_exon12	0	0		28.7	18.1	0.0	16.4	0.0
55	chr15:91525083-91525297 (-)	PRC1_exon11|PRC1_exon12	0	2		28.5	27.9	0.0	31.3	0.0
56	chr1:8928048-8928110 (-)	ENO1_exon8	1	2	SNORD7	28.4	15.5	0.0	0.0	11.5
57	chr21:34931559-34931690 (+)	SON_exon5	4	4	SNORD125	28.2	34.0	0.0	18.7	0.0
58	chr1:71537813-71537922 (-)	ZRANB2_exon6|ZRANB2_exon5	4	2	SNORD100	27.4	20.3	0.0	14.2	0.0
59	chr6:64286626-64286667 (+)	PTP4A1_exon2	0	0		27.3	14.3	14.4	13.4	0.0
60	chr12:69983255-69983461 (+)	CCT2_exon7	0	3	SNORD61	27.0	75.4	31.5	61.1	0.0
61	chr1:89206870-89206935 (+)	PKN2_exon2	0	0		26.8	20.8	0.0	20.4	0.0
62	chr2:61633103-61633149 (-)	USP34_exon78	1	1		26.6	36.3	0.0	20.1	0.0
63	chr10:129909922-129909981 (-)	MKI67_exon5	0	0		26.2	23.4	0.0	10.9	0.0
64	chr1:60330835-60330899 (+)	HOOK1_exon18	1	1		25.8	19.0	0.0	18.9	0.0
65	chr14:45583711-45583764 (+)	PRPF39_exon13	0	1		25.2	21.9	0.0	14.7	0.0
66	chr14:69846928-69846971 (-)	ERH_exon1	0	0		25.1	15.9	19.1	14.5	0.0
67	chr1:115280561-115280725 (-)	CSDE1_exon17|CSDE1_exon16	3	3	SNORD59B,SNORD59A	24.8	29.6	0.0	16.8	0.0
68	chr5:138650367-138650426 (+)	MATR3_exon5|MATR3_exon2|MATR3_exon3|MATR3_exon6	0	0		24.7	16.1	0.0	20.1	0.0
69	chr1:173915886-173915949 (-)	RC3H1_exon5	0	0		24.6	24.5	0.0	13.9	0.0
70	chr12:58112840-58112877 (+)	OS9_exon11|OS9_exon12	0	0		24.6	12.6	0.0	13.2	0.0
71	chr6:35825108-35825145 (-)	SRPK1_exon4	0	1	SNORD1B	24.2	23.8	0.0	36.4	0.0
72	chr5:54456113-54456153 (+)	GPX8_exon1	0	0		24.2	21.6	0.0	17.8	0.0
73	chr6:24781720-24781778 (+)	GMNN_exon4	0	1		24.1	29.4	0.0	10.7	0.0

74	chr21:34142130-34142277 (-)	GCFC1_exon15|GCFC1_exon17	2	1	SNORD37,SNORD36C	23.9	25.2	0.0	16.9	0.0
75	chr14:31085484-31085525 (+)	G2E3_exon15	0	0		23.0	16.1	0.0	23.3	0.0
76	chr17:62496641-62496927 (-)	DDX5_exon2	2	3		23.0	19.5	10.3	19.9	0.0
77	chr2:232326251-232326495 (-)	NCL_exon12	30	17	SNORD53	22.8	19.8	0.0	0.0	10.5
78	chr5:137342749-137342829 (-)	FAM13B_exon17|FAM13B_exon16	1	2		22.7	16.9	0.0	10.7	0.0
79	chr10:89692773-89692807 (+)	PTEN_exon5	0	0	SNORD124	22.6	21.1	0.0	11.3	0.0
80	chr22:39137508-39137558 (-)	SUN2_exon9	0	0		22.6	16.7	0.0	13.1	0.0
81	chr20:32693178-32693294 (-)	EIF2S2_exon8	3	4		22.4	13.7	0.0	0.0	12.3
82	chr3:41267176-41267217 (+)	CTNNB1_exon6	0	0		22.0	28.3	0.0	31.3	0.0
83	chr1:94018768-94018807 (+)	FNBP1L_exon14|FNBP1L_exon17|FNBP1L_exon15	0	0	SNORD64	21.9	20.5	0.0	17.6	0.0
84	chr8:26218548-26218590 (+)	PPP2R2A_exon6	0	0		21.9	33.2	28.4	51.7	0.0
85	chr1:24088287-24088323 (+)	TCEB3_exon11	0	0	SNORD82	21.6	16.2	12.3	0.0	0.0
86	chr1:235274005-235274038 (-)	TOMM20_exon1	0	0	SNORD28	21.5	14.2	44.6	25.6	0.0
87	chr5:74072472-74072556 (+)	NSA2_exon6	0	0	SNORD36C	21.4	22.1	0.0	13.9	0.0
88	chr10:7840971-7841017 (+)	ATP5C1_exon4	0	1	SNORD125	21.3	21.0	0.0	10.8	0.0
89	chr13:20567445-20567535 (+)	ZMYM2_exon4|ZMYM2_exon3	1	2		20.7	26.5	0.0	13.9	0.0
90	chr19:46355745-46355792 (-)	SYMPK_exon24	0	1		20.5	31.1	0.0	15.7	0.0
91	chr6:17649378-17649457 (-)	NUP153_exon11	0	1		20.3	12.0	0.0	19.5	0.0
92	chr8:124382116-124382279 (-)	ATAD2_exon22	31	22		20.2	21.8	0.0	15.2	0.0
93	chr10:96361232-96361363 (+)	HELLS_exon22	0	2	SNORD7	20.2	24.3	0.0	35.3	0.0
94	chr17:27613848-27613936 (-)	NUFIP2_exon3	0	0		20.1	21.9	0.0	24.2	0.0
95	chr3:185407344-185407381 (-)	IGF2BP2_exon10|IGF2BP2_exon11	1	1		20.1	21.0	0.0	14.3	0.0
96	chr21:34931875-34931993 (+)	SON_exon6	0	0		19.9	21.9	0.0	11.1	0.0
97	chr6:44216431-44216534 (+)	HSP90AB1_exon2	0	1		19.9	48.0	35.8	39.0	0.0
98	chr16:88798743-88798788 (-)	PIEZO1_exon31	0	0	SNORD125	19.7	24.2	0.0	10.3	0.0
99	chr11:63967644-63967701 (+)	STIP1_exon10	0	1	SNORD32A,SNORD51,SNORD32B,SNORD2	19.5	15.7	0.0	13.0	0.0

100	chr12:102120116-102120157 (+)	CHPT1_exon8	0	0		19.4	29.2	0.0	26.7	0.0
101	chr3:156262098-156262199 (-)	SSR3_exon2	1	1		19.2	14.6	0.0	12.2	0.0
102	chr3:47770519-47770556 (-)	SMARCC1_exon23	0	0		18.5	11.9	0.0	13.7	0.0
103	chr13:107196340-107196543 (-)	ARGLU1_exon1	2	2	SNORD12,SNORD125,SNORD57	18.5	24.5	0.0	13.1	0.0
104	chr17:46673268-46673321 (-)	HOXB6_exon1	0	0		18.1	25.6	0.0	11.4	0.0
105	chr2:153537774-153537887 (-)	PRPF40A_exon21	0	3		18.0	19.9	0.0	15.4	0.0
106	chr2:38527433-38527469 (-)	ATL2_exon4	0	0	SNORD18C,SNORD47	17.5	21.4	0.0	11.3	0.0
107	chr2:85769370-85769468 (+)	MAT2A_exon6	3	3	SNORD53,SNORD31	16.7	13.4	0.0	12.6	0.0
108	chr19:41809912-41809974 (+)	HNRNPUL1_exon13	0	0		16.7	22.4	0.0	11.1	0.0
109	chr14:91937239-91937277 (-)	SMEK1_exon6	0	0		16.7	19.1	0.0	10.5	0.0
110	chr12:124104478-124104553 (+)	DDX55_exon14	2	2	SNORD61,SNORD110	16.6	26.0	0.0	12.0	0.0
111	chr3:52727676-52727768 (+)	GNL3_exon13	1	2	SNORD98,SNORD124	16.6	23.0	0.0	10.2	0.0
112	chr19:30503299-30503417 (+)	URI1_exon10	1	2		16.6	35.7	0.0	47.4	0.0
113	chr3:160131227-160131402 (+)	SMC4_exon8|SMC4_exon7	2	6	SNORD14A,SNORD14B	16.4	13.6	0.0	39.8	0.0
114	chr9:100760884-100760925 (+)	ANP32B_exon3	0	1		16.2	12.5	37.5	24.8	0.0
115	chr12:7046133-7046171 (+)	ATN1_exon5	0	0		16.1	13.2	0.0	13.2	0.0
116	chr2:20454955-20455035 (-)	PUM2_exon4	0	0		15.7	17.5	0.0	29.5	0.0
117	chr19:39957121-39957181 (+)	SUPT5H_exon11|SUPT5H_exon12	1	2		15.7	19.9	0.0	11.4	0.0
11	chr1:173780364-173780400 (-)	CENPL_exon5|	0	1		15.3	21.1	0.0	11.4	0.0

8		CENPL_exon4								
119	chr12:49087416-49087474 (-)	CCNT1_exon1	0	0		15.0	10.2	0.0	18.9	0.0
120	chr18:20572711-20572755 (+)	RBBP8_exon11	0	0	SNORD125	15.0	10.9	0.0	10.5	0.0
121	chrX:100356154-100356252 (+)	CENPI_exon2	4	4		14.9	20.6	0.0	11.6	0.0
122	chr12:56575282-56575378 (-)	SMARCC2_exon19|SMARCC2_exon21|SMARCC2_exon20	0	0		14.8	11.5	0.0	13.6	0.0
123	chr15:42005350-42005392 (+)	MGA_exon9	0	0		14.7	14.1	0.0	10.9	0.0
124	chr2:47388862-47388996 (-)	CALM2_exon2	6	5		14.5	27.8	0.0	18.4	0.0
125	chr20:17930977-17931030 (-)	SNX5_exon7	0	0	SNORD54	14.4	15.6	0.0	13.8	0.0
126	chr3:47125318-47125429 (-)	SETD2_exon10	3	5	SNORD36C	14.4	14.4	0.0	11.3	0.0
127	chr19:44739145-44739265 (+)	ZNF227_exon6	2	4	SNORD36C	14.0	23.8	0.0	31.8	0.0
128	chr17:36943072-36943131 (-)	PIP4K2B_exon9	0	1		14.0	12.4	0.0	10.8	0.0
129	chr6:136582406-136582611 (-)	BCLAF1_exon2	6	4	SNORD36C,SNORD111B,SNORD111	13.8	21.3	0.0	14.3	0.0
130	chr5:145859600-145859682 (+)	TCERG1_exon11|TCERG1_exon12	2	2	SNORD45B,SNORD45A	13.8	11.2	0.0	17.9	0.0
131	chr16:89799734-89799819 (+)	ZNF276_exon7	0	1	SNORD125	13.6	29.7	0.0	17.1	0.0
132	chr17:65822286-65822398 (+)	BPTF_exon1	2	3		13.6	10.5	0.0	12.4	0.0
133	chr3:133336070-133336154 (-)	TOPBP1_exon7	0	3	SNORD14A,SNORD14B	13.6	28.5	0.0	16.1	0.0
134	chr12:111162430-111162583 (-)	PPP1CC_exon5|PPP1CC_exon4	0	2	SNORD7	13.5	13.1	0.0	18.5	0.0
135	chr9:79985381-79985440 (+)	VPS13A_exon64|VPS13A_exon65	1	3		13.4	17.7	0.0	10.7	0.0
13	chr18:2772272-2772359 (+)	SMCHD1_exon41	0	1	SNORD36C,SNORD1	13.4	10.2	0.0	10.1	0.0

6					25					
137	chr11:93470202-93470407 (-)	TAF1D_exon3	4	8	SNORD71	13.4	11.0	0.0	15.9	0.0
138	chr3:56658541-56658620 (-)	FAM208A_exon2	0	1	SNORD90	13.0	13.2	0.0	13.8	0.0
139	chr13:107211849-107212002 (-)	ARGLU1_exon3	1	1		13.0	17.1	0.0	10.9	0.0
140	chr17:4624451-4624495 (+)	ARRB2_exon15| ARRB2_exon14	0	0		12.9	13.0	0.0	29.3	0.0
141	chr17:60042568-60042625 (-)	MED13_exon11	0	0		12.5	11.4	0.0	14.2	0.0
142	chr12:56553821-56553851 (+)	MYL6_exon4	1	1		12.4	20.6	12.2	0.0	0.0
143	chr12:56713170-56713267 (-)	PAN2_exon4	0	0	SNORD20	12.4	18.3	0.0	13.5	0.0
144	chr1:224345072-224345136 (+)	FBXO28_exon5| FBXO28_exon4	0	0	SNORD55	12.3	10.2	0.0	10.5	0.0
145	chr19:38872775-38872926 (+)	PSMD8_exon6	1	2	SNORD124	12.2	14.9	0.0	10.2	0.0
146	chr8:17074716-17074754 (+)	ZDHHC2_exon12	0	0		12.0	11.3	0.0	11.0	0.0
147	chr1:91383640-91383697 (-)	ZNF644_exon2	0	0	SNORD125	12.0	22.2	0.0	14.2	0.0
148	chr6:80751981-80752026 (+)	TTK_exon22	0	1		11.6	21.5	0.0	12.8	0.0
149	chr10:88197192-88197250 (-)	WAPAL_exon1	1	1		11.5	16.7	0.0	12.3	0.0
150	chr12:53862567-53862651 (+)	PCBP2_exon12| PCBP2_exon11| PCBP2_exon13	1	2		11.1	29.7	0.0	16.9	0.0
151	chr1:220145421-220145454 (-)	EPRS_exon3	0	0		10.9	10.9	0.0	10.1	0.0
152	chr7:26236448-26236661 (-)	HNRNPA2B1_exon8	2	3		10.6	28.1	0.0	11.0	0.0
153	chr16:30723156-30723346 (+)	SRCAP_exon12	9	9		10.6	10.8	0.0	12.5	0.0
15	chr10:123722759-123722854 (-)	NSMCE4A_exon6	0	0	SNORD125	10.4	28.1	0.0	24.6	0.0

4									
155	chr6:90564578-90564634 (+)	CASP8AP2_exon4	1	3	10.3	11.7	0.0	0.0	10.3
156	chrX:40994009-40994068 (+)	USP9X_exon5	1	1	10.2	13.7	0.0	10.4	0.0
157	chr8:30938479-30938597 (+)	WRN_exon9	1	2	10.2	21.6	0.0	13.3	0.0
