# Supplementary material for: Insights into snoRNA biogenesis and processing from PAR-CLIP of snoRNA core proteins and small RNA sequencing
Source: Genome Biol. 2013 May 26;14(5):R45. doi: 10.1186/gb-2013-14-5-r45 (PMC4053766; doi:10.1186/gb-2013-14-5-r45)
Supplement: Additional file 12 — Detailed list of reads mapping to snoRNA loci in Ago2 IP-seq libraries. [file gb-2013-14-5-r45-S12.PDF]

Supplementary Table. Detailed listing of reads from Ago2 IP-seq libraries that mapped to snoRNA loci.

HeLa Ago2 IP-seq (asynchronous cells)				HeLa Ago2 IP-seq (mitotic cells)			
**C/D box snoRNA ID**	**Number of reads**	**H/ACA box snoRNA ID**	**Number of reads**	**C/D box snoRNA ID**	**Number of reads**	**H/ACA box snoRNA ID**	**Number of reads**
SNORD1A	1140 (46.74 %)	SCARNA15	3636 (29.16 %)	SNORD45C	877 (5.82 %)	SCARNA15	1904 (14.95 %)
SNORD80	116 (4.76 %)	SNORA25	1776 (14.24 %)	SNORD65	761 (5.05 %)	SNORA73B	1393 (10.94 %)
SNORD14B	94 (3.85 %)	SNORA33	1497 (12.01 %)	SNORD45A	586 (3.89 %)	SNORA25	748 (5.87 %)
SNORD14A	42 (1.72 %)	SNORA63	1034 (8.29 %)	SNORD79	516 (3.42 %)	SNORA66	724 (5.69 %)
SCARNA9L	33 (1.35 %)	SNORA32	661 (5.30 %)	SNORD104	460 (3.05 %)	SNORA33	704 (5.53 %)
SNORD104	31 (1.27 %)	SNORA3	627 (5.03 %)	SNORD18C	430 (2.85 %)	SNORA73A	692 (5.43 %)
SNORD45C	31 (1.27 %)	SCARNA3	531 (4.26 %)	SNORD1A	427 (2.83 %)	SNORA63	616 (4.84 %)
SNORD65	30 (1.23 %)	SCARNA16	287 (2.30 %)	SNORD81	419 (2.78 %)	SCARNA3	530 (4.16 %)
SNORD12B	26 (1.07 %)	SNORA74A	192 (1.54 %)	SNORD50A	418 (2.77 %)	SNORA24	374 (2.94 %)
SNORD22	24 (0.98 %)	SNORA19	146 (1.17 %)	SNORD18B	369 (2.45 %)	SNORA32	363 (2.85 %)
SNORD49B	24 (0.98 %)	SNORA51	143 (1.15 %)	SNORD27	364 (2.41 %)	SNORA75	359 (2.82 %)
SNORD93	23 (0.94 %)	SNORA45	107 (0.86 %)	SNORD80	354 (2.35 %)	SNORA3	355 (2.79 %)
SNORD95	22 (0.90 %)	SNORA78	97 (0.78 %)	SNORD45B	351 (2.33 %)	SNORA61	238 (1.87 %)
SNORD55	22 (0.90 %)	SNORA65	93 (0.75 %)	SNORD34	302 (2.00 %)	SCARNA16	214 (1.68 %)
SNORD45A	22 (0.90 %)	SCARNA18	90 (0.72 %)	SNORD33	207 (1.37 %)	SNORA4	194 (1.52 %)
SNORD83B	21 (0.86 %)	SNORA40	89 (0.71 %)	SNORD83B	204 (1.35 %)	SNORA81	165 (1.30 %)
SNORD81	21 (0.86 %)	SNORA26	80 (0.64 %)	SNORD14A	201 (1.33 %)	SNORA8	159 (1.25 %)
SNORD27	19 (0.78 %)	SNORA41	69 (0.55 %)	SNORD22	193 (1.28 %)	SNORA74A	137 (1.08 %)
SNORD118	18 (0.74 %)	SNORA10	65 (0.52 %)	SNORD28	193 (1.28 %)	SNORA62	119 (0.93 %)
SNORD119	17 (0.70 %)	SCARNA8	65 (0.52 %)	SNORD118	186 (1.23 %)	SNORA58	115 (0.90 %)
SNORD48	17 (0.70 %)	SNORA76	62 (0.50 %)	SNORD25	177 (1.17 %)	SNORA26	112 (0.88 %)
SNORD2	16 (0.66 %)	SNORA61	61 (0.49 %)	SNORD32A	172 (1.14 %)	SNORA19	110 (0.86 %)
SNORD33	16 (0.66 %)	SNORA56	57 (0.46 %)	SNORD47	170 (1.13 %)	SNORA67	91 (0.71 %)
SNORD12C	16 (0.66 %)	SNORA71B	55 (0.44 %)	SNORD55	166 (1.10 %)	SNORA56	87 (0.68 %)
SNORD32A	16 (0.66 %)	SNORA28	53 (0.43 %)	SNORD11B	161 (1.07 %)	SNORA40	86 (0.68 %)
SNORD50A	15 (0.62 %)	SNORA73B	49 (0.39 %)	SNORD52	158 (1.05 %)	SNORA65	84 (0.66 %)
SNORD29	15 (0.62 %)	SNORA67	44 (0.35 %)	SNORD12C	154 (1.02 %)	SNORA45	75 (0.59 %)
SNORD25	14 (0.57 %)	SCARNA14	42 (0.34 %)	SNORD46	152 (1.01 %)	SNORA53	73 (0.57 %)
SNORD18B	14 (0.57 %)	SNORA50	42 (0.34 %)	SNORD12B	149 (0.99 %)	SNORA64	70 (0.55 %)
SNORD24	14 (0.57 %)	SNORA69	38 (0.30 %)	SNORD24	146 (0.97 %)	SNORA1	69 (0.54 %)
SNORD96A	14 (0.57 %)	SNORA22	32 (0.26 %)	SNORD85	138 (0.92 %)	SNORA6	68 (0.53 %)
SNORD51	13 (0.53 %)	SNORA60	31 (0.25 %)	SNORD2	135 (0.90 %)	SNORA51	63 (0.49 %)
SNORD58C	13 (0.53 %)	SNORA17	31 (0.25 %)	SNORD96A	135 (0.90 %)	SNORA9	61 (0.48 %)
SNORD52	13 (0.53 %)	SNORA73A	30 (0.24 %)	SNORD43	129 (0.86 %)	SCARNA18	56 (0.44 %)
SNORD31	11 (0.45 %)	SNORA48	23 (0.18 %)	SNORD18A	129 (0.86 %)	SNORA41	49 (0.38 %)
SNORD18C	11 (0.45 %)	SNORA36C	23 (0.18 %)	SNORD29	117 (0.78 %)	SNORA34	48 (0.38 %)
SNORD46	11 (0.45 %)	SNORA75	21 (0.17 %)	SNORD87	115 (0.76 %)	SNORA38	45 (0.35 %)
SNORD45B	10 (0.41 %)	SNORA70	20 (0.16 %)	SNORD14B	114 (0.76 %)	SNORA14B	45 (0.35 %)

SNORD62A	10 (0.41 %)	SNORA20	20 (0.16 %)	SNORD95	113 (0.75 %)	SNORA76	44 (0.35 %)
SNORD11B	10 (0.41 %)	SNORA7A	18 (0.14 %)	SNORD26	109 (0.72 %)	SNORA70	43 (0.34 %)
SNORD83A	10 (0.41 %)	SNORA79	18 (0.14 %)	SNORD6	108 (0.72 %)	SNORA23	41 (0.32 %)
SNORD87	10 (0.41 %)	SCARNA4	17 (0.14 %)	SNORD38B	106 (0.70 %)	SCARNA23	40 (0.31 %)
SNORD79	9 (0.37 %)	SNORA38	17 (0.14 %)	SNORD13	105 (0.70 %)	SNORA31	39 (0.31 %)
SNORD34	9 (0.37 %)	SCARNA27	16 (0.13 %)	SNORD99	103 (0.68 %)	SNORA55	38 (0.30 %)
SNORD26	9 (0.37 %)	SNORA55	15 (0.12 %)	SNORD61	101 (0.67 %)	SCARNA11	37 (0.29 %)
SNORD21	9 (0.37 %)	SNORA39	14 (0.11 %)	SNORD51	95 (0.63 %)	SCARNA14	37 (0.29 %)
SNORD41	8 (0.33 %)	SNORA44	14 (0.11 %)	SNORD42B	95 (0.63 %)	SNORA18	36 (0.28 %)
SCARNA9	8 (0.33 %)	SNORA31	14 (0.11 %)	SNORD117	92 (0.61 %)	SNORA13	35 (0.27 %)
SNORD49A	8 (0.33 %)	SNORA64	14 (0.11 %)	SNORD15B	90 (0.60 %)	SNORA10	35 (0.27 %)
SNORD38A	8 (0.33 %)	SNORA24	14 (0.11 %)	SNORD76	90 (0.60 %)	SNORA52	35 (0.27 %)
SNORD10	8 (0.33 %)	SNORA66	14 (0.11 %)	SNORD83A	90 (0.60 %)	SNORA78	34 (0.27 %)
SNORD68	8 (0.33 %)	SNORA54	13 (0.10 %)	SNORD77	87 (0.58 %)	SNORA36C	34 (0.27 %)
SCARNA2	8 (0.33 %)	SNORA8	11 (0.09 %)	SNORD11	86 (0.57 %)	SNORA28	33 (0.26 %)
SNORD74	8 (0.33 %)	SNORA1	11 (0.09 %)	SNORD97	85 (0.56 %)	SNORA2B	31 (0.24 %)
SNORD69	8 (0.33 %)	SNORA4	11 (0.09 %)	SCARNA2	79 (0.52 %)	SNORA39	30 (0.24 %)
SNORD77	7 (0.29 %)	SNORA53	11 (0.09 %)	SNORD68	72 (0.48 %)	SCARNA13	30 (0.24 %)
SNORD75	7 (0.29 %)	SNORA38B	11 (0.09 %)	SNORD91A	70 (0.46 %)	SCARNA8	30 (0.24 %)
SNORD17	7 (0.29 %)	SNORA80	10 (0.08 %)	SNORD60	67 (0.44 %)	SNORA16A	30 (0.24 %)
SNORD15B	7 (0.29 %)	SNORA7B	10 (0.08 %)	SNORD38A	65 (0.43 %)	SNORA7B	28 (0.22 %)
SNORD13	7 (0.29 %)	SNORA81	10 (0.08 %)	SNORD88C	61 (0.40 %)	SNORA71B	27 (0.21 %)
SNORD42B	7 (0.29 %)	SNORA5A	9 (0.07 %)	SNORD119	61 (0.40 %)	SCARNA4	25 (0.20 %)
SNORD85	7 (0.29 %)	SCARNA13	9 (0.07 %)	SNORD93	60 (0.40 %)	SNORA69	25 (0.20 %)
SNORD99	7 (0.29 %)	SNORA2B	9 (0.07 %)	SNORD59A	59 (0.39 %)	SNORA48	25 (0.20 %)
SNORD92	6 (0.25 %)	SCARNA11	8 (0.06 %)	SNORD49A	59 (0.39 %)	SNORA21	25 (0.20 %)
SNORD61	6 (0.25 %)	SNORA16A	7 (0.06 %)	SNORD10	58 (0.38 %)	SNORA15	24 (0.19 %)
SNORD1B	6 (0.25 %)	SNORA14B	7 (0.06 %)	SNORD44	57 (0.38 %)	SNORA57	24 (0.19 %)
SNORD57	6 (0.25 %)	SNORA43	7 (0.06 %)	SNORD5	57 (0.38 %)	SNORA72	24 (0.19 %)
SNORD36C	6 (0.25 %)	SNORA72	7 (0.06 %)	SNORD1B	55 (0.36 %)	SNORA60	22 (0.17 %)
SNORD76	6 (0.25 %)	SNORA57	7 (0.06 %)	SNORD84	55 (0.36 %)	SNORA50	22 (0.17 %)
SNORD11	6 (0.25 %)	SNORA29	6 (0.05 %)	SNORD16	54 (0.36 %)	SNORA22	21 (0.16 %)
SNORD54	6 (0.25 %)	SNORA58	6 (0.05 %)	SNORD48	54 (0.36 %)	SNORA44	19 (0.15 %)
SNORD47	5 (0.21 %)	SNORA23	6 (0.05 %)	SCARNA9	54 (0.36 %)	SNORA46	19 (0.15 %)
SNORD102	5 (0.21 %)	SNORA42	5 (0.04 %)	SNORD57	53 (0.35 %)	SNORA17	19 (0.15 %)
SNORD60	5 (0.21 %)	SNORA6	5 (0.04 %)	SNORD101	53 (0.35 %)	SNORA38B	18 (0.14 %)
SNORD12	5 (0.21 %)	SNORA62	5 (0.04 %)	SNORD50B	52 (0.34 %)	SNORA68	17 (0.13 %)
SCARNA12	5 (0.21 %)	SNORA46	5 (0.04 %)	SNORD58C	52 (0.34 %)	SNORA27	15 (0.12 %)
SNORD30	5 (0.21 %)	SNORA77	5 (0.04 %)	SNORD49B	51 (0.34 %)	SNORA5A	14 (0.11 %)
SNORD20	5 (0.21 %)	SNORA2A	4 (0.03 %)	SNORD17	50 (0.33 %)	SNORA70E	13 (0.10 %)
SNORD18A	5 (0.21 %)	SNORA71D	4 (0.03 %)	SNORD54	50 (0.33 %)	SCARNA1	13 (0.10 %)
SNORD28	5 (0.21 %)	SNORA11	3 (0.02 %)	SNORD74	50 (0.33 %)	SNORA12	13 (0.10 %)
SNORD43	5 (0.21 %)	SNORA13	3 (0.02 %)	SNORD111B	49 (0.32 %)	SNORA14A	12 (0.09 %)

SNORD58A	4 (0.16 %)	SCARNA22	3 (0.02 %)	SNORD75	49 (0.32 %)	SNORA77	11 (0.09 %)
SNORD97	4 (0.16 %)	SNORA14A	3 (0.02 %)	SNORD62A	48 (0.32 %)	SNORA20	11 (0.09 %)
SNORD44	4 (0.16 %)	SNORA34	3 (0.02 %)	SCARNA9L	47 (0.31 %)	SNORA43	11 (0.09 %)
SNORD53	4 (0.16 %)	SNORA11E	2 (0.02 %)	SCARNA12	47 (0.31 %)	SNORA5C	10 (0.08 %)
SNORD38B	4 (0.16 %)	SNORA71A	2 (0.02 %)	SNORD12	47 (0.31 %)	SCARNA27	10 (0.08 %)
SNORD50B	4 (0.16 %)	SNORA70B	2 (0.02 %)	SNORD1C	46 (0.31 %)	SNORA84	8 (0.06 %)
SNORD88C	4 (0.16 %)	SNORA52	2 (0.02 %)	SNORD36B	46 (0.31 %)	SNORA80	8 (0.06 %)
SNORD117	4 (0.16 %)	SNORA74B	2 (0.02 %)	SNORD92	46 (0.31 %)	SNORA29	7 (0.05 %)
SNORD6	4 (0.16 %)	SNORA21	2 (0.02 %)	SNORD36A	45 (0.30 %)	SNORA80B	7 (0.05 %)
SNORD84	4 (0.16 %)	SNORA9	2 (0.02 %)	SNORD20	44 (0.29 %)	SNORA42	7 (0.05 %)
SNORD66	3 (0.12 %)	SNORA36B	2 (0.02 %)	SNORD110	44 (0.29 %)	SNORA74B	6 (0.05 %)
SNORD1C	3 (0.12 %)	SNORA49	2 (0.02 %)	SNORD72	40 (0.27 %)	SCARNA22	6 (0.05 %)
SNORD127	3 (0.12 %)	SNORA71C	2 (0.02 %)	SNORD100	40 (0.27 %)	SNORA71A	6 (0.05 %)
SNORD103A	3 (0.12 %)	SNORA70F	2 (0.02 %)	SNORD58A	40 (0.27 %)	SNORA54	6 (0.05 %)
SNORD105B	3 (0.12 %)	SNORA84	2 (0.02 %)	SNORD102	37 (0.25 %)	SNORA30	6 (0.05 %)
SNORD4B	3 (0.12 %)	SNORA70E	1 (0.01 %)	SNORD41	36 (0.24 %)	SNORA11	5 (0.04 %)
SNORD110	3 (0.12 %)	SNORA15	1 (0.01 %)	SNORD69	35 (0.23 %)	SNORA2A	5 (0.04 %)
SNORD63	3 (0.12 %)	SNORA12	1 (0.01 %)	SNORD21	35 (0.23 %)	SNORA11E	5 (0.04 %)
SNORD101	3 (0.12 %)	SNORA80B	1 (0.01 %)	SCARNA10	33 (0.22 %)	SNORA37	4 (0.03 %)
SNORD37	3 (0.12 %)	SNORA18	1 (0.01 %)	SNORD88A	32 (0.21 %)	SNORA71D	4 (0.03 %)
SNORD36A	3 (0.12 %)	SNORA30	1 (0.01 %)	SNORD36C	30 (0.20 %)	SNORA71C	4 (0.03 %)
SNORD58B	3 (0.12 %)			SNORD66	30 (0.20 %)	SNORA7A	4 (0.03 %)
SNORD78	3 (0.12 %)			SNORD35A	30 (0.20 %)	SNORA79	4 (0.03 %)
SNORD59A	2 (0.08 %)			SNORD70	27 (0.18 %)	SNORA36B	3 (0.02 %)
SNORD72	2 (0.08 %)			SNORD31	27 (0.18 %)	SNORA70B	2 (0.02 %)
SNORD88B	2 (0.08 %)			SNORD30	27 (0.18 %)	SNORA70D	1 (0.01 %)
SNORD36B	2 (0.08 %)			SNORD63	24 (0.16 %)	SCARNA20	1 (0.01 %)
SNORD70	2 (0.08 %)			SNORD91B	24 (0.16 %)	SNORA49	1 (0.01 %)
SNORD35A	2 (0.08 %)			SNORD15A	24 (0.16 %)	SNORA70G	1 (0.01 %)
SNORD35B	2 (0.08 %)			SNORD90	24 (0.16 %)		
SNORD94	2 (0.08 %)			SNORD103A	22 (0.15 %)		
SNORD67	2 (0.08 %)			SNORD37	22 (0.15 %)		
SNORD121A	1 (0.04 %)			SNORD53	22 (0.15 %)		
SNORD15A	1 (0.04 %)			SNORD105	21 (0.14 %)		
SNORD5	1 (0.04 %)			SNORD78	21 (0.14 %)		
SNORD19	1 (0.04 %)			SNORD19	20 (0.13 %)		
SNORD19B	1 (0.04 %)			SNORD105B	20 (0.13 %)		
SNORD91A	1 (0.04 %)			SNORD7	19 (0.13 %)		
SNORD73A	1 (0.04 %)			SNORD98	18 (0.12 %)		
SNORD111B	1 (0.04 %)			SNORD19B	18 (0.12 %)		
SCARNA7	1 (0.04 %)			SNORD121A	17 (0.11 %)		
SNORD16	1 (0.04 %)			SNORD58B	16 (0.11 %)		
SNORD89	1 (0.04 %)			SCARNA7	14 (0.09 %)		

SNORD91B	1 (0.04 %)
SNORD88A	1 (0.04 %)
SNORD105	1 (0.04 %)
SCARNA10	1 (0.04 %)

SNORD86	12 (0.08 %)
SNORD4A	12 (0.08 %)
SNORD121B	12 (0.08 %)
SNORD67	11 (0.07 %)
SNORD59B	11 (0.07 %)
SNORD4B	11 (0.07 %)
SNORD23	11 (0.07 %)
SNORD73A	10 (0.07 %)
SNORD89	10 (0.07 %)
SNORD94	10 (0.07 %)
SNORD35B	8 (0.05 %)
SNORD32B	7 (0.05 %)
SNORD82	7 (0.05 %)
SNORD111	6 (0.04 %)
SNORD126	6 (0.04 %)
SNORD127	6 (0.04 %)
SNORD56	5 (0.03 %)
SNORD88B	5 (0.03 %)
SNORD9	4 (0.03 %)
SNORD8	4 (0.03 %)
SNORD42A	3 (0.02 %)
SNORD96B	2 (0.01 %)
SNORD62B	1 (0.01 %)
SCARNA17	1 (0.01 %)
SNORD124	1 (0.01 %)
