# Supplementary material for: Insights into snoRNA biogenesis and processing from PAR-CLIP of snoRNA core proteins and small RNA sequencing
Source: Genome Biol. 2013 May 26;14(5):R45. doi: 10.1186/gb-2013-14-5-r45 (PMC4053766; doi:10.1186/gb-2013-14-5-r45)
Supplement: Additional file 13 — Supplementary materials and methods. Detailed information about the experimental methods (PAR-CLIP library preparation, Northern blotting, primer extension assays, mitotic shake-off and Ago2 immunoprecipitation and sequencing). In addition, the annotated C/D and H/ACA snoRNAs used in this study are listed. [file gb-2013-14-5-r45-S13.PDF]

**PAR-CLIP library preparation**

Library preparations from PAR-CLIP experiments were essentially done as described \cite{Hafner2010}. Briefly, RNA recovered by proteinase K digestion from immunoprecipitated complexes was ligated with adenylated 3'-adapter using truncated T4 RNA ligase (New England Biolabs) followed by adapter ligation to the 5' end with T4 RNA ligase (Fermentas). Products were size fractionated on denaturing acrylamide gels. Molecules of appropriate size were reverse transcribed with Superscript III (Invitrogen) reverse transcriptase and RNase H treated to digest remaining RNA. 10% of the reactions was used for a pilot PCR reaction. To this end, aliquots were taken every second cycle between 14 and 28 cycles and analyzed on a 2.5 % agarose gel. The number of cycles causing a first visible amplification was chosen for large scale PCR. PCR products were extracted from agarose gels using a Qiaex II Gel extraction kit (Qiagen). Libraries were sequenced on an Illumina HiSeq2000 deep sequencer.

**Northern Blotting**

scaRNA21

Two probes were designed, 5'-GTG GGC TGA GAC CAC CCC CCA GGA AAT CGA-3' and 5'-CCA GGA AAT CGA GCC AGG AAG TAT TGT ATG, which specifically target the H/ACA box element of SCARNA21 and both the C/D box and the H/ACA box elements, respectively.

HEK293 RNA was isolated using Tri-reagent (Ambion), according to manufacturer's instructions. 15 µg of total RNA were separated on a 8% polyacrylamide gel and blotted onto a nylon membrane (Amersham). RNA was cross-linked to the membrane with 254 nm light twice at 0.1 J cm-2. Membranes were hybridized using ULTRAhyb-Oligo Buffer (Ambion), according to manufacturer's instructions. Blots were exposed overnight using a phosphor screen.

Novel snoRNAs

Northern blots for detection of novel snoRNAs were performed as described above. RNA was separated on a 12% polyacrylamide gel. For a complete list of probes see the Table below.

| **Name** | **Sequence** | **Detected with Northern Blotting** |
|---|---|---|
| ZL1 | GCATGCACTGACAAGCATATACACA | yes |
| ZL2 | ATCCCCGGGTATTGCTCTCC | yes |
| ZL8 | TCTTGTCCAACCAGATCATCAT | yes |
| ZL11 | GAAACCCAATGATAGTCATCACAGGAAGTCCT | yes |
| ZL63 | GCTTCAGCTTTAGGGGAACA | yes |
| ZL107 | ACATCAGAAAGCGTTTACAGTATCATGAATTC | yes |
| ZL116 | GTGGCATCAGTAACTGCTCATCAT | yes |
| ZL126 | TGGATCTCATGTCTTCACTTTGGTATGAA | yes |
| ZL127 | GTCTTCACTTCAATACTAAGACCAAT | yes |

| | | |
|---|---|---|
| ZL5 | TCCACATACATTAATCACAACA | no |
| ZL6 | TCAATAATCACAACGGTTCAT | no |
| ZL7 | AAGCTCTGAAGTCTACATCACA | no |
| ZL9 | CTCTATACTCAGTCACCTCC | no |
| ZL27 | AGCTCAGAAGCTAATAAACATCACA | no |
| ZL30 | CATGGCTCTGCAAGCATCATGTA | no |
| ZL49 | CAGTCAGTCTCCACAGTTGGAGTCATC | no |
| ZL77 | TCAGATATAATATTCATCACCA | no |
| ZL92 | GTCAGGGTAAAATCGCAACACAT | no |
| ZL103 | AATGGAGATAATCATCGAAAGG | no |
| ZL109 | TGACAAATACATCACGGCACA | no |

**Detection of 2'-O-ribose-methyls and pseudouridines**

RNA extraction

Total cellular RNA was extracted from HEK293 cells. Cells were trypsinized and pelleted by centrifugation. Pelleted cells were lysed in TRI Reagent (Sigma) and separated into aqueous and organic phases by chloroform addition and subsequent centrifugation. RNA was precipitated from the aqueous phase in 1 µl of co-precipitant (GlycoBlue), 0.3M NaCl, and 2.5 volumes 100% ethanol. The RNA was stored for 1 hour at -20°C before centrifugation for 30 min at full speed in an Eppendorf centrifuge. The pellet was washed twice with 70% ethanol and then air-dried. Subsequently, the pellet was treated with DNase (DNase RQ1 Promega) for 30 min at 37°C to remove DNA. RNA was then extracted with phenol-chloroform-isoamyl alcohol 25:24:1 pH 5 and precipitated with ethanol as described above.

32P End labeling of reverse Primers

Two hundred picomoles of the reverse primer was run on a 15% 8M UREA-PAGE gel and gel-extracted in order to purify the primers. The primers were phosphorylated in a 25µl reaction containing 12.5 picomoles of the page purified primer, 2.5µl 10X PNK buffer, 1.5µl T4 PNK, 5.0µl [γ-32P] ATP (10µCi/µl) for 1 h at 37°C. Unincorporated label was removed by centrifugation using a Microspin column (GE Healthcare).

2'-O-methylation assay

Mix A: 40 µg of DNase-treated HEK293 total RNA and 7 µl of the radiolabeled reverse primer were dissolved in 10 X RT buffer (500 mM Tris-Cl pH 8.6, 600 mM NaCL, 100 mM dTT) in a 40 µl reaction volume, and heated at 65 °C for 5 minutes, then immediately put on ice. Mix B: 400 U of SuperScript® III Reverse Transcriptase were dissolved in 10X RT buffer in a total volume of 20 µl. Primer Extension Reaction: 12 µl of Mix A were mixed with 6 µl of Mix B, 6 µl 25 mM MgCl2, and 6 µl of either 5 mM dNTPs, 0.5 mM dNTPs, or 0.05 mM dNTPs. The dNTP gradient was varied and optimized for some samples, the final dNTP concentrations are indicated on the figures. The reaction was incubated at 42°C for 20 minutes, 50°C for 20 minutes, and 55°C for 20 minutes. The reaction was RNase A treated at 37°C for 30 minutes. cDNA was

ethanol precipitated in 0.3M NaCl 70% ethanol with 2 µl of co-precipitant and resuspended in 7.5 µl of 1 X RNA loading dye. Dideoxy sequencing products were generated: a PCR on genomic DNA with forward and reverse primers for the gene of interest was performed. The appropriate size amplicon was gel extracted from a 2.5 % agarose gel to serve as template for the sequencing reaction. The sequencing mix was as follows: 5.0 µl PCR gel extracted template, 6.0 µl radio-labeled reverse primer, 5.0µl 5X sequencing buffer (250 mM Tris, and 10mM MgCl2, pH9), 10 U Taq Polymerase. Of this mix 4.0 µl were distributed to four separate tubes and 2.0 µl of individual ddNTP/dNTP mixes were added to the tubes. The ddNTP/dNTP mixes were as follows: all had 20 µM dCTP, dGTP, dTTP, dATP, and 37.5 µM ddGTP, 473.5 µM ddATP, 750 µM ddTTP, 250 µM ddCTP.
A PCR with a total volume of 6.0 µl was performed as follows: 95°C: 1min; (95°C:30s,55°C:30s,72°C:30s) :35 cycles; 4°C stop. The PCR product was diluted 1:4 in 2x RNA loading buffer and 4 µl were loaded onto the gel. DNAs were separated on a 20 cm x 40 cm x 0.75 mm 8% polyacrylamide/8M urea gel run at 40 W until the bromophenol blue dye reached the bottom. The gel was dried at 90°C under vacuum for three hours and analyzed by phosphorimaging.

Pseudouridylation assay

In brief, 25 µg of DNase-treated total RNA were dissolved in 20 µl water, 80 µl BEU buffer(7M Urea, 4 mM EDTA, 50 mM Bicine, pH 8.5) and 20 µl of 1 M 1-Cyclohexyl-3-(2-morpholinoethyl)carbodiimide metho-p-toluenesulfonate (CMC) in BEU buffer was added. This is the plus CMC sample. For a negative control, we added 100 µl of BEU buffer to 25 µg of DNase-treated total RNA in 20 µl water. Both samples were incubated at 37°C for 20 min to allow for CMC modifications of U,G, and ψ. The modified RNA was precipitated by adding 2 µl co-precipitant, 50 µl 3 M sodium acetate, pH 5.5, and 600 µl of ethanol. The samples were vortexed and incubated 5 minutes at room temperature. RNA was pelleted at 13’000 rpm for 10 minutes at room temperature and the supernatant was removed. The pellets were washed twice with 200 µl of -20°C 70% ethanol and then air-dried. The RNA pellets were redissolved in 50 µl sodium carbonate buffer, pH 10.4 and incubated at 37°C for 4 hours to allow removal of CMC from U and G. The RNA was precipitated by adding 2 µl of co-precipitant, 6 µl of 3 M sodium acetate, and 110 µl of ethanol. The samples were incubated 5 minutes at room temperature and the RNA was pelleted and washed twice with ethanol as described above. The RNA pellets were dissolved in 20 µl water.

For reverse transcription, 10 µg of plus CMC-and control RNA were dissolved in a 43.5 µl total reaction containing 5 µl of radiolabeled reverse primer and 5 µl 0.5mM dNTPs. The mixtures were incubated at 65°C for 5 minutes and put on ice for 2 minutes. Subsequently, 12 µl 5X First-Strand Buffer, 3 µl 0.1 M DTT, and 1.5 µl (200 units/µl) of SuperScript III RT enzyme were added to the reaction mixtures. The mixtures were incubated at 42°C for 20 minutes, 50°C for 20 minutes, and 55°C for 20 minutes. The cDNA mixture was treated with RNase A at 37°C for 30 minutes and ethanol precipitated in 0.3M NaCl 70% ethanol with 2 µl of co-precipitant and resuspended in 7.5 µl of 1 X RNA loading dye. Hereinafter, the procedure was the same as described in the 2’-O-methylation assay.

**Mitotic shake-off**

In the evening prior to the shake-off experiment, the cells were plated at approximately 60-70% confluency in DMEM supplemented with 10% FCS in eight T75 flasks (Falcon, 353133). The cells were incubated at 37°C and 5% $CO_2$ overnight. Twelve hours later, the medium over the flask surface was swirled, discarded and replaced with prewarmed fresh medium to remove any unattached cells. Each flask was gently tapped 30 times onto the surface of the bench after which the medium, which now contained the mitotic cells, was swirled and collected into 15 ml Falcon tubes. Prewarmed fresh medium was added to the flasks, which were then put back into the incubator. The collected mitotic fractions were centrifuged 5 min at 500 x g at room temperature to pellet the cells. The supernatant was removed, the pellets snap-frozen in liquid nitrogen, and stored at -80°C. This procedure was repeated at one-hour intervals until 48 mitotic fractions

were collected. One T75 flask was plated with asynchronous HeLa H2B GFP-tagged cells at approximately 60-70% confluency and incubated overnight at 37°C. The cells were harvested and the pellet snap-frozen in liquid nitrogen and stored at -80°C.

**Ago2 immunoprecipitation and small RNA library preparation**

Ago2 immunoprecipitation (IP) was performed on the mitotic and asynchronous cells. As control for antibody specificity an IP on asynchronous cells using unspecific IgG was done. Briefly, monoclonal Anti-Ago2 antibody and IgG antibody were coupled to Dynabeads G. The mitotic fractions were combined in RIPA lysis buffer (50 mM Tris-HCl pH 7.4, 150 mM NaCl, 0.5 % Sodum Deoxycholate, 1% (v/v) NP-40, 0.1% SDS, 1mM EDTA, 0.5 mM DTT(add directly before the experiment), EDTA-free protease inhibitors(add directly before the experiment)) and lysed on ice for 10 minutes together with the asynchronous cells sample and the control. The lysates were centrifuged, filtered through 0.45 micron syringe filters and subjected to IP for one hour. The magnetic beads were collected using a magnetic rack and washed five times with IP wash buffer (50mM HEPES pH 7.5, 300 mM KCl, 0.05% (v/v) NP40, 0.5 mM DTT(add directly before the experiment), EDTA-free protease inhibitors(add directly before the experiment)). IP wash buffer was removed and 500 µl Trizol (Sigma) was added to the beads and vortexed. After 5 minutes of incubation 100 µl chloroform was added to the samples, the samples were vortexed and centrifuged at 12’000 x g for 15 minutes at 4 °C. The aqueous phase was collected and RNA was ethanol precipitated in the presence of a co-precipitant. Recovered RNA was 5’-end radiolabeled with [$\gamma$-$^{32}$P] ATP and separated on a 12 % denaturing Polyacrylamide gel. RNA that was 20-30 nt long was size-selected and extracted from the gel with 0.4 M NaCl solution and ethanol precipitated in the presence of a co-precipitant. A small RNA library preparation protocol was used \cite{Hafner2010} and samples were sequenced 50 cycles using Solexa technology. Reads were preprocessed and mapped as described before.

**Curated C/D and H/ACA box snoRNA annotation**

SnoRNA sequences were initially obtained from snoRNA-LBME-db. BLAT searches at the UCSC Genome Browser (http://genome.ucsc.edu/cgi-bin/hgBlat) were then conducted to map sequences to the human genome assembly hg19. Based on the data available at snoRNA-LBME-db and inspection of evolutionary conservation profiles at the UCSC Genome Browser, we next annotated C, C’, D’ and D box motifs, and the terminal closing stem. For some snoRNAs, terminal end complementarity could be extended beyond the initial sequence obtained from snoRNA-LBME-db. In these cases sequence coordinates were adjusted accordingly.

Each individual snoRNA is represented by 4 lines, which correspond to the description of the snoRNA, the sequence, annotation of C (C), D (D), C'(c) and D'(d) boxes, and the location of the terminal stem region in parenthesis notation. The description line contains the snoRNA name, its coordinates (chromosome, start, stop and strand) in the hg19 version of the human genome assembly from the University of California Santa Cruz, and the modifications sites and target molecules of the guide regions located upstream of the D’ box (d) or D box (D).

```
>SNORD11|chr2|203157774|203157857|+|::|509:18S:D
GTGTTCAATGATGATTTCTATTTGTTTGCCTGATTTCCTTTTGGATAATGAAGGCATCTTTAGTCACTACCTCTTCTGAGACAC
.......CCCCCCC...............dddd..............ccccccc.....................DDDD.....
((((............................................................................))))
>SNORD11B|chr2|203156055|203156144|+|::|509:18S:D
TGATGGCAATGATGATTTTTACACTTATTGTTGTTCACCTGATAACATAAATATGAGGGTGTTCAGTCACTACCTCATCTGATGCCATCA
........CCCCCCC.......................dddd..........ccccccc...................DDDD........
((((((((..........................................................................))))))))
>SNORD12|chr20|47897220|47897309|+|3878:28S:d|::
GCCTTTGCAGCTGATGATACAGCTTCTTTCCCCATCAGATCGACCCTGTTGATCTCTACACTATTGGCCAGTTTTGTCTGATGCATTGGC
..........CCCCCCC..................dddd......ccccccc.........................DDDD.........
(((..((((........................................................................))))..)))
>SNORD12B|chr20|47896856|47896946|+|3878:28S:d|::
GCTGGCATATATGATGACTTAGCTTTTTTCCCCGACAGATCGACTATGTTGATCTAACTTTTCTAAGCCAGTTTCTGTCTGATATGCCAGC
..........CCCCCCC..................dddd......ccccccc..........................DDDD.........
(((((((((.........................................................................)))))))))
>SNORD12C|chr20|47895478|47895564|+|1536:18S:d|1602:18S:D
CTGGTGTAAATGATGACTTCACTTTTTTCCCCATCAGATCGACAATGCTGACGTCTTATATTTTGCCAGTTAGTTCTGATACATCGG
.........CCCCCCC..................dddd......ccccccc........................DDDD........
((((((((.......................................................................))))))))
>SNORD119|chr20|2443605|2443686|-|::|4560:28S:D
GCTGGATTAATGATGAGATATAACCTTGACTGAAGCTGATGATGAGTTTGTATAATTAAGCAGGATTACTCTGAGATCCAGC
.........CCCCCCC.............dddd.....ccccccc.........................DDDD........
(((((((....................................................................)))))))
>SNORD19B|chr3|52724763|52724840|+|::|683:18S:D
TGGTTGAAATATGATGAGTGTACAAAATCTTGATTTAAGTGAATGAAAAATTACAAGATCCAACTCTGATTTCAGCCA
..........CCCCCCC............dddd.........ccccccc................DDDD.........
(((((((((............................................................)))))))))
>SNORD121A|chr9|33952767|33952848|-|4607:28S:d|::
GTCCAGAAAACAATGATGTGGTAATTTCCAAGCACATATCTGATGATTCCATGTGGAATTTAACTACCTGAGTTTCCTGGAC
............CCCCCCC....................dddd.......ccccccc..........DDDD...........
((((((.((((............................................................)))).))))))
>SNORD121B|chr9|33934295|33934373|-|4607:28S:d|::
TGGAAAAGACAATGATGTTTTATTTCCAAGCACATATCTGAGTTGTATGTGTGGACAGCACTGAGACTGAGTCTTTCCA
...........CCCCCCC...................dddd.....ccccccc.............DDDD.........
((((((.(((............................................................)))))))))
>SNORD123|chr5|9548948|9549017|+|::|::
GGTGAAAATGATGAATTCTGGGGCGCTGATTCATGTGACTTGAAAAATGCCATCCATTTCCTGATTCACC
.......CCCCCCC...........dddd..........ccccccc..............DDDD......
((((((..........................................................))))))
>SNORD124|chr17|38183804|38183890|-|::|::
AGGAAGGGATGATGTTCCAGTTGAGACTCAAGAAAAGGATTCTGAGCCTCAGAGCTTTGAAGGAGCCACTTGGTCCCTGACCTTCCT
........CCCCCCC..........................dddd...........ccccccc.............DDDD.......
(((((((.........................................................................)))))))
>SNORD125|chr22|29729158|29729240|-|::|::
GCAGCCCCTCCTGATGATTCTTCTTCCTGAGCACGCTCATGATGAGCAAACTGAGCCTCTAAGAAGTTGACTGAAGGGGCTGC
..........CCCCCCC.........dddd........ccccccc.........................DDDD.........
(((((((((.................................................................)))))))))
>SNORD126|chr14|20794609|20794685|-|::|::
AGTTTGCCATGATGAAATGCATGTTAAGTCCGTGTTTCAGCTGATCAGCCTGATTAAACACATGCTCTGAGCAGACT
........CCCCCCC.........................dddd.....ccccccc..........DDDD.......
(((((((...............................................................)))))))
>SNORD127|chr14|45580086|45580171|+|::|1447:18S:D
TGGCAACTGTGATGAAAGATTTGGTCTGTATGTAATAGATTTTATTACTAAATGAGGACAACAGTCCCTCTAAACTGATGTTGCCA
........CCCCCCC................dddd................ccccccc................DDDD........
(((((((........................................................................)))))))
>SNORD101|chr6|133136446|133136518|+|::|::
GTTTGAATGATGACTTTAATTGTCGGATACCCCTTCACTCCTTTTATGAGTGAAACATAAGAGTCTGACAAAC
......CCCCCCC...................dddd.........ccccccc............DDDD.....
(((((...............................................................)))))
>SNORD102|chr13|27829201|27829272|+|::|4020:28S:D
AGCTTAATGATGACTGTTTTTTTTGATTGCTTGAAGCAATGTGAAAAACACATTTCACCGGCTCTGAAAGCT
......CCCCCCC..................................................DDDD.....
(((((..............................................................)))))
```

>SNORD103A|chr1|31408533|31408622|-|601:18S:d|::
TGTCTGGCAATGATGACCCACTTGCCCTCACTGAGAACAAAGTTCGGTAATGAGAATCTTTGTTAATGGACTCAAGTTCTGAGCCAGACA
.........CCCCCCC..............dddd...............ccccccc......................DDDD........
((((((((..........................................................................))))))))
>SNORD103B|chr1|31421962|31422051|-|601:18S:d|::
TGTCTGGCAATGATGACCCACTTGCCCTCACTGAGAACAAAGTTCGGTAATGAGAATCTTTGTTAATGGACTCAAGTTCTGAGCCAGACA
.........CCCCCCC..............dddd...............ccccccc......................DDDD........
((((((((..........................................................................))))))))

>SNORD104|chr17|62223438|62223517|+|1327:28S:d|::
GGCCTGCTGTGATGACATTCCAATTAAAGCACGTGTTAGACTGCTGACGCGGGTGATGCGAACTGGAGTCTGAGCCTGCC
........CCCCCCC............................dddd.......ccccccc........DDDD.......
(((..(((................................................................)))..)))
>SNORD105|chr19|10218328|10218411|+|::|799:18S:D
CCCTATCTCTCATGATGAACACATATGCCTCTGAGCTGCTGTGATTTCTGGCTTCAAAGTAAACGCTCTGAAGAAGAGATGGGG
...........CCCCCCC............dddd.....ccccccc.....................DDDD.............
((((((((((................................................................))))))))))
>SNORD105B|chr19|10220433|10220511|+|::|799:18S:D
CCACATGCGGCTGATGACAGCACTTCTGCTGAGACGCTGTGATTGCTCTGTCCAAAGTAAACGCCCTGACGCACTGTGG
..........CCCCCCC...........dddd.....ccccccc.....................DDDD..........
(((((((((............................................................)))).)))))
>SNORD13|chr8|33371003|33371096|+|::|::
AGTTCATGAGCGTGATGATTGGGTGTTCATACGCTTGTGTGAGATGTGCCACCCTTGAACCTTGTTACGACGTGGGCACATTACCCGTCTGACC
...........CCCCCCC......................................................................DDDD..
..............................................................................................
>SNORD14A|chr11|17097326|17097415|-|::|462:18S:D
TCACTGTGATGATGGTTTTCCAACATTCGCAGTTTCCACCAGAAAGGTTTTCCTTAGTGTTGGGTAAACCTTCCTTGGATGTCTGAGTGA
.....CCCCCCC...........................dddd.............ccccccc...................DDDD....
(((((................................................................................)))))
>SNORD14B|chr11|17096201|17096291|-|::|462:18S:D
TCACTATGATGATTGGTTGCCAGACATTCGCAGTTTCCACCAGAAATGTTTTTCCTTATGTTGGCCAGTTCTTCCTTGGATGTCTGAGTGA
.....CCCCCCC............................dddd.............ccccccc...................DDDD....
(((((.................................................................................)))))
>SNORD15A|chr11|75111433|75111583|+|::|3764:28S:D
CCCTTCGATGAAGAGATGATGACGAGTCTGACTTGGGGATGTTCTCTTTGCCCAGGTGGCCTACTCTGTGCTGCGTTCTGTGGCACAGTTTAAAGAGCC
CTGGTTGAAGTAATTTCCTAAAGATGACTTAGAGGCATTTGTCTGAGAAGGG
.......CCCCCCC.....................................................................................
..........................................DDDD......
((((((.............................................................................................
..............................................))))))
>SNORD15B|chr11|75115463|75115611|+|::|3764:28S:D
CCCTTCAGTGATGACACGATGACGAGTCAGAAAGGTCACGTCCTGCTCTTGGTCCTTGTCAGTGCCATGTTCTGTGGTGCTGTGCACGAGTTCCTTTGG
CAGAAGTGTCCTATTTATTGATCGATTTAGAGGCATTTGTCTGAGAAGGG
.......CCCCCCC.....................................................................................
........................................DDDD......
((((((.............................................................................................
............................................))))))
>SNORD16|chr15|66795149|66795249|-|484:18S:d|::
TTGCAATGATGTCGTAATTTGCGTCTTACTCTGTTCTCAGCGACAGTTGCCTGCTGTCAGTAAGCTGGTACAGAAGGTTGACGAAAATTCTTACTGAGC
AA
.....CCCCCCC............dddd......................ccccccc....................................DDDD..
..
((((.............................................................................................))
))
>SNORD18A|chr15|66795579|66795653|-|1313:28S:d|::
ACAGTAGTGATGAAATTCCACTTCATTGGTCCGTGTTTCTGAACCACATGATTTTCTCGGATGTTCTGATGCTGT
......CCCCCCC.........................dddd.....ccccccc...........DDDD......
((((((...............................................................))))))
>SNORD18B|chr15|66794355|66794431|-|1313:28S:d|::
TGTCAAAATGATGAGATTCCACTTAATTGGTCCGTGTTTCTGAAACACATGATATTTGTGGAAATTCTGACTTGGCA
.......CCCCCCC.........................dddd.....ccccccc...........DDDD.......
((((((.................................................................))))))
>SNORD18C|chr15|66793581|66793663|-|1313:28S:d|::
TCAGCTTTTGTTATGATGAGATTCCACTTAAGGTCCGTGTTTCTGAAACAAATGATTTTGTGGAAGTTCTGATTTATGGCTGA

```
............CCCCCCC.......................dddd.....ccccccc..........DDDD...........
((((((.......................................................................))))))
>SNORD20|chr2|232321156|232321234|-|::|1804:18S:D
TGGATATGATGACTGATTACCTGAGAAATAATTGATGAAATCTCAAGAAAATTCCTCTAGATAGTCAAGTTCTGATCCA
.....CCCCCCC........dddd.......ccccccc.................................DDDD....
(((((.....................................................................)))))
>SNORD21|chr1|93302843|93302940|+|::|1303:28S:D
GCTGCTGAATGATGATATCCCACTAACTGAGCAGTCAGTAGTTGGTCCTTTGGTTGCATATGATGCGATAATTGTTTCAAGACGGGACTGATGGCAGC
........CCCCCCC...........dddd.............................ccccccc.....................DDDD.......
((((((......................................................................................))))))
>SNORD24|chr9|136216251|136216325|+|2352:28S:d|2338:28S:D
TGCAGATGATGTAAAAGAATATTTGCTATCTGAGAGATGGTGATGACATTTTAAACCACCAAGATCGCTGATGCA
.....CCCCCCC.................dddd......ccccccc.....................DDDD....
((((...................................................................))))
>SNORD25|chr11|62623039|62623102|-|1490:18S:d|::
TCCTATGATGAGGACCTTTTCACAGACCTGTACTGAGCTCCGTGAGGATAAATAACTCTGAGGA
....CCCCCCC.....................dddd.....ccccccc.........DDDD...
((((........................................................))))


>SNORD28|chr11|62622091|62622170|-|::|1391:18S:D
ACTGTCAGATGATTTGAATTGATAAGCTGATGTTCTGTGAGGTACAAAAGTTAATAGCATGTTAGAGTTCTGATGGCAGT
........CCCCCCC...........dddd......ccccccc..........................DDDD.......
(((((((..................................................................)))))))
>SNORD29|chr11|62621374|62621443|-|4493:28S:d|::
CAGTTTCTATGATGAATCAAACTAGCTCACTATGACCGACAGTGAAAATACATGAACACCTGAGAAACTG
........CCCCCCC................dddd......ccccccc...........DDDD.......
((((((((......................................................))))))))
>SNORD31|chr11|62620795|62620867|-|4166:28S:d|::
CTCACCAGTGATGAGTTGAATACCGCCCCAGTCTGATCAATGTGTGACTGAAAGGTATTTTCTGAGCTGTGAG
.......CCCCCCC..................dddd...........ccccccc.......DDDD........
(((((.(((........................................................))))))))
>SNORD32A|chr19|49993223|49993304|+|1511:28S:d|1328:18S:D
AGGTCAGTGATGAGCAACATTCACCATCTTTCGTTTGAGTCTCACGGCCATGAGATCAACCCCATGCACCGCTCTGAGACCT
......CCCCCCC.....................dddd...........ccccccc.................DDDD.....
(((((........................................................................)))))
>SNORD32B|chr6|29550025|29550113|+|1511:28S:d|1326:18S:D
CTGGATTGGTGATGAGCAACAATCACCATCTTTCGTTTGAGTCTCATGGCCATGAGACCAACCCCATGCACTGCTCTGAGACCTGCCAG
........CCCCCCC.....................dddd...........ccccccc.................DDDD..........
((((.................................................................................))))
>SNORD33|chr19|49993874|49993954|+|::|1391:18S:D
GGCCGGTGATGAGAACTTCTCCCACTCACATTCGAGTTTCCCGACCATGAGATGACTCCACATGCACTACCATCTGAGGCC
.....CCCCCCC...................dddd.............ccccccc..................DDDD....
((((.........................................................................))))
>SNORD34|chr19|49994161|49994231|+|2824:28S:d|::
TGGCGTCCATGATGTTCCGCAACTACCTACATTGTTTGATCCTCATGAAAGCAGCACTGGCTGAGACGCCA
........CCCCCCC....................dddd..ccccccc............DDDD.......
(((((((.........................................................)))))))
>SNORD35A|chr19|49994429|49994515|+|4506:28S:d|::
CCTGGCAGATGATGTCCTTATCTCACGATGGTCTGCGGATGTCCCTGTGGGAATGGCGACAATGCCAATGGCTTAGCTGATGCCAGG
........CCCCCCC....................dddd.............ccccccc.................DDDD.......
(((((((.........................................................................)))))))
>SNORD35B|chr19|50000975|50001060|+|4506:28S:d|::
TTGGCAGATGATGTTTGTTTTCACGATGGTCTTCAGATGCCCACGTGGGCACTGCTGAGAAAGCCACTTGGTAAAACTGATGCCGG
.......CCCCCCC...................dddd..............ccccccc..................DDDD......
((((((..........................................................................))))))
>SNORD36A|chr9|136217311|136217382|+|::|668:18S:D
TTGCAATGATGTGAATCTCTCACTGAATTCAACCTTGAAGTGCGAATCCATGAGCTTTTTAACCCTGAGCAA
.....CCCCCCC..........dddd........ccccccc.......................DDDD....
((((................................................................))))
>SNORD36B|chr9|136216946|136217026|+|::|668:18S:D
GCTGTTGCAGTGATGTAAAATTTCTTGGCCTGAAATTACTGTGAAGAGTAAAACCGAGCTTTTTAACACTGAGTCAGCAGC
.........CCCCCCC.............dddd.......ccccccc.....................DDDD.........
```

(((((((...................................................................)))))))
>SNORD36C|chr9|136217698|136217770|+|3703:28S:d|::
CTTTTGCCAATGATGGTTAAGAATTTCTTCACCTGAATAAACCATGTGGTCAGCATTGCATCTGAGGCAAAAG
.........CCCCCCC................dddd.......ccccccc...........DDDD........
((((((((.........................................................))))))))
>SNORD37|chr19|3982505|3982570|-|3697:28S:d|::
ATTCGTGATGACTGATCATTTCTTCACTTTGACCAGATGTCTACTGAAGAAAGCCTGCGTCTGAGG
....CCCCCCC......................dddd......ccccccc..........DDDD..
..................................................................
>SNORD38A|chr1|45243511|45243587|+|1858:28S:d|::
GCCTTCTCGTGATGAAAACTCTGTCCAGTTCTGCTACTGAAGGGAGAGAGATGAGAGCCTTTTAGGCTGAGGAAGGC
........CCCCCCC.....................dddd.......ccccccc............DDDD.......
(((((((((..........................................................))).))))))
>SNORD38B|chr1|45244057|45244135|+|1858:28S:d|::
GGCTGTCTCAGTGATGAAAACTTTGTCCAGTTCTGCTACTGACAGTAAGTGAAGATAAAGTGTGTCTGAGGAGACAGCT
..........CCCCCCC.....................dddd......ccccccc..........DDDD..........
(((((((((.............................................................)))))))))
>SNORD41|chr19|12817265|12817330|-|::|4276:28S:D
GGAAGTGATGACACCTGTGACTGTTGATGTGGAACTGATTTATCGCGTATTCGTACTGGCTGATCC
....CCCCCCC.....dddd..............ccccccc..................DDDD...
(((............................................................)))
>SNORD42A|chr17|27050448|27050511|+|::|116:18S:D
GGCTAATGATGGAAAAATCATTATTGGAAAAGAATGACATGAACAAAGGAACCACTGAAGTGCC
.....CCCCCCC............dddd.....ccccccc..............DDDD......
(((..........................................................)))
>SNORD42B|chr17|27047568|27047634|+|::|116:18S:D
GTGCATATGATGGAAAAGTTTTAATCTCCTGACACTTGTGATGTCTTCAAAGGAACCACTGATGCAC
......CCCCCCC...............dddd.....ccccccc..............DDDD.....
(((((.........................................................)))))

>SNORD43|chr22|39715054|39715119|-|1703:18S:d|::
CCACAGATGATGAACTTATTGACGGGCGGACAGAAACTGTGTGCTGATTGTCACGTTCTGATTTGG
......CCCCCCC.................dddd......ccccccc..........DDDD.....
(((............................................................)))
>SNORD44|chr1|173835100|173835167|-|::|166:18S:D
GCCTGGATGATGATAAGCAAATGCTGACTGAACATGAAGGTCTTAATTAGCTCTAACTGACTAAAGGC
......CCCCCCC..............dddd..ccccccc................DDDD........
((((............................................................))))
>SNORD45A|chr1|76253571|76253658|+|172:18S:d|159:18S:D
CAAGGTCAATGATGTGTTGGCATGTATTATCTGAATCTATTGCTGATGTGTAATAACACTTTAGCTCTAGAATTACTCTGAGACCTTG
........CCCCCCC...............dddd........ccccccc............................DDDD.......
(((((((..........................................................................)))))))
>SNORD45B|chr1|76255159|76255234|+|172:18S:d|::
CAAGGTCAATGATGTAATGGCATGTATTAGCTGAATCTAAAGTTGATGTGAGTTCTAAAATTACACTGAGACCTTG
........CCCCCCC...............dddd........ccccccc................DDDD.......
(((((((..............................................................)))))))
>SNORD45C|chr1|76252757|76252833|+|::|159:18S:D
GGTCAATGATGAGTTGGCATGTATTCTGAATCTAAAGTTGATTATTACTACTTTAGCTCTAGAATTACTCTGAGACC
.....CCCCCCC.............dddd........ccccccc.........................DDDD....
((((.....................................................................))))
>SNORD46|chr1|45242156|45242270|+|::|3739:28S:D
GGAGGCAAGTAGGGTGATGAAAAAGAATCCTTAGGCGTGGTTGTGGCCGTCTTGGTCACCTGTGTGCCACTTGCCAATGCAAGGACTTGTCATAGTTAC
ACTGACTGTTGCCTCC
.............CCCCCCC...............................................................................
.DDDD...........
((((((((.(((.......................................................................................
.....)))))))))))
>SNORD48|chr6|31803036|31803110|+|::|2279:28S:D
AGGGAGTGATGATGACCCCAGGTAACTCTTGAGTGTGTCGCTGATGCCATCACCGCAGCGCTCTGACCGCCCCCT
........CCCCCCC............dddd.........ccccccc...............DDDD.........
((((.(((...........................................................))).))))
>SNORD49A|chr17|16343344|16343429|+|4426:28S:d|::
CTTGACTGCTCTGATGAAATCACTAATAGGAAGTGCCGTCAGAAGCGATAACTGACGAAGACTACTCCTGTCTGATTGCAGTCAAG
..........CCCCCCC......................dddd........ccccccc.............DDDD...........

(((((((((....................................................................)))))))))
>SNORD49B|chr17|16342816|16342896|+|4426:28S:d|::
GACTGTCCTGATGATACTTGTAATAGGAAGTGCCGTCAGAAGCGATAACTGACGACGTCTAATGTCTATCTGACCGCAGTC
.......CCCCCCC......................dddd........ccccccc..............DDDD........
((((((.....................................................................))))))
>SNORD50A|chr6|86387007|86387091|-|2863:28S:d|2848:28S:D
GGTCTTATCTGTGATGATCTTATCCCGAACCTGAACTTCTGTTGAAAAAAAAAAACTTTTACGGATCTGGCTTCTGAGATGGACC
..........CCCCCCC.............dddd....ccccccc............................DDDD........
(((((.(((....................................................................))))))))
>SNORD50B|chr6|86387300|86387381|-|::|::
GGTCTAATCAATGATGAAACCTATCCCGAAGCTGATAACCTGAAGAAAAATAAGTACGGATTCGGCTTCTGAGATTAAGACC
..........CCCCCCC..............dddd....ccccccc......................DDDD..........
(((((((((...............................................................)))).)))))
>SNORD51|chr2|207026600|207026683|+|1511:28S:d|::
GACTGGTTGCATGATGAATAAAATCAAATCACCATCTTTCGGCTGAGTTCGTGATGGATTTGCTTTTTTCTGATTAAGCCAGTC
..........CCCCCCC.........................dddd....ccccccc............DDDD...........
((((((((....................................................................))))))))
>SNORD52|chr6|31804844|31804928|+|3904:28S:d|::
TCCCTAATTGGGAATGATGATTTCACAGACTAGAGTCTCCGATGCTGGTCATGATGTCAAAACTAAGTTCTGACTCATTTAGGGA
.............CCCCCCC.................dddd.........ccccccc............DDDD............
(((((((.((((.............................................................)))).)))))))
>SNORD55|chr1|45241532|45241615|+|::|2791:28S:D
CGCACGTGTATGATGACAACTCGGTAATGCTGCATACTCCCGAGTGCGCGGTGGGGAAGCCAACCTTGGAGAGCTGAGCGTGCG
.........CCCCCCC.............dddd.................ccccccc................DDDD.......
(((((((......................................................................)))))))
>SNORD57|chr20|2637584|2637658|+|::|99:18S:D
TTGGAGGTGATGAACTGTCTGAGCCTGACCTTGTAGAATGGAGGCAAAAAAACTGATTTAATGAGCCTGATCCAA
......CCCCCCC...........dddd.........ccccccc......................DDDD.....
(((((.................................................................)))))
>SNORD58A|chr18|47017653|47017718|-|4198:28S:d|::
GCTGCAGTGATGACTTTCTTAGGACACCTTTGGATTTACCGTGAAAATTAATAAATTCTGAGCAGC
......CCCCCCC................dddd.......ccccccc..........DDDD.....
(((((........................................................)))))
>SNORD58B|chr18|47018034|47018100|-|4198:28S:d|::
GCTGCGATGATGGCATTTCTTAGGACACCTTTGGATTAATAATGAAAACAACTACTCTCTGAGCAGC
......CCCCCCC.................dddd.......ccccccc..........DDDD.....
(((((.........................................................)))))

>SNORD58C|chr18|47015614|47015680|-|4198:28S:d|4198:28S:D
GGTTGCTGTGATGACTATCTTAGGACACCTTTGGAATAACTATGAAAGAAAACTATTCTGAGCAACC
.......CCCCCCC................dddd.......ccccccc.........DDDD......
(((((((.....................................................)))))))
>SNORD59A|chr12|57038811|57038885|-|1031:18S:d|::
CCTTCTATGATGATTTTATCAAAATGACTTTCGTTCTTCTGAGTTTGCTGAAGCCACATTTAGGTACTGAGAAGG
......CCCCCCC.........................dddd.....ccccccc............DDDD.....
((((((...............................................................))))))
>SNORD59B|chr12|57037459|57037539|-|1031:18S:d|::
CTATTCCTCACTGATGAGTACGTTCTGACTTTCGTTCTTCTGAGTTTGCTGAAGCCAGATGCAATTTCTGAGAAGGAATAG
..........CCCCCCC......................dddd.....ccccccc............DDDD..........
((((((((.................................................................))))))))
>SNORD60|chr16|2205024|2205106|-|4340:28S:d|::
AGTCTGTGATGAATTGCTTTGACTTCTGACACCTCGTATGAAAACTGCACGTGCAGTCTGATTATTTAGCAAGACTGAGGCTT
.....CCCCCCC.........................dddd................ccccccc..........DDDD.....
...................................................................................
>SNORD61|chrX|135961358|135961432|-|::|1442:18S:D
AAGCTATGATGAATTTGATTGCATTGATCGTCTGACATGATAATGTATTTTTGTCCTCTAAGAAGTTCTGAGCTT
.....CCCCCCC...........dddd.........ccccccc........................DDDD....
(((((.................................................................)))))
>SNORD62A|chr9|134361051|134361138|+|590:18S:d|::
GTCTCAGTGATGTAATTCCAATAGATCCTTCTGACCCTCCACTGTGGACTCAATAGCAGGGAGATGAAGAGGACAGTGACTGAGAGAC
......CCCCCCC.................dddd.......ccccccc...............................DDDD.....
(((((..............................................................................)))))
>SNORD62B|chr9|134365872|134365959|+|590:18S:d|::

GTCTCAGTGATGTAATTCCAATAGATCCTTCTGACCCTCCACTGTGGACTCAATAGCAGGGAGATGAAGAGGACAGTGACTGAGAGAC
......CCCCCCC..................dddd......ccccccc...............................DDDD.....
(((((..............................................................................)))))
>SNORD73A|chr4|152024976|152025046|+|1747:28S:d|::
GGGAATAAGTGATGAAAAAAGTTTCGGTCCCAGATGATGGCCAGTGATAACAACATTTTTCTGATGTTCCC
........CCCCCCC..................dddd......ccccccc..........DDDD.......
(((((((.........................................................)))))))
>SNORD73B|chr4|152023207|152023282|+|::|::
GGAATGAATGATGACAAAATGTTTCAGTCCCAAATGATACATACTGATTATACCATTATATTTATCCTGACATTCC
.......CCCCCCC...................dddd......ccccccc................DDDD......
((((((................................................................))))))
>SNORD75|chr1|173836017|173836076|-|4032:28S:d|::
AGCCTGTGATGCTTTAAGAGTAGTGGACAGAAGGGATTTCTGAAATTCTATTCTGAGGCT
.....CCCCCCC...............dddd.....ccccccc.........DDDD....
((((....................................................))))
>SNORD76|chr1|173835771|173835854|-|2350:28S:d|::
GTGCCACAATGATGACAGTTTATTTGCTACTCTTGAGTGCTAGAATGATGAGGATCTTAACCACCATTATCTTAACTGAGGCAC
........CCCCCCC.................dddd......ccccccc..........................DDDD.....
(((((..........................................................................)))))
>SNORD77|chr1|173835438|173835509|-|1521:28S:d|::
CAGATACTATGATGGTTGCATAGTTCAGCAGATTTAATCATGAAGAGATGTACTATCTGTCTGATGTATCTG
........CCCCCCC.............dddd.......ccccccc..............DDDD........
(((((((..........................................................)))))))
>SNORD78|chr1|173834754|173834833|-|4593:28S:d|::
ACGGGGTTTGTGTAATGATGTTGATCAAATGTCTGACCTGAAATGAGCATGTAGACAAAGGTAACACTGAAGAACCCTGT
..............CCCCCCC.............dddd..........ccccccc...........DDDD..........
(((((((((..............................................................)))))))))
>SNORD81|chr1|173833283|173833361|-|391:28S:d|::
GCAGAATACATGATGATCTCAATCCAACTTGAACTCTCTCACTGATTACTTGATGACAATAAAATATCTGATATTCTGC
.........CCCCCCC.........................dddd....ccccccc...........DDDD........
((((((((...............................................................))))))))
>SNORD82|chr2|232325079|232325153|-|1678:18S:d|::
ACAGCACAAATGATGAATAACAAAGGGACTTAATACTGAAACCTGATGTTACATTGTAGTGTGCTGATGTGCTGT
.........CCCCCCC...................dddd...ccccccc..............DDDD........
((((((((...........................................................))))))))
>SNORD117|chr6|31504150|31504228|-|::|::
GAGCCAAATGATGTTTATTTGAAACAGGAGCACCTCAGTGCAAGGACGACTCTTATCTATCACCCATGACTGATGGCTC
.......CCCCCCC.......................................................DDDD......
((((((...................................................................))))))
>SNORD83A|chr22|39711218|39711312|-|::|::
GCTGTTCGTTGATGAGGCTCAGAGTGAGCGCTGGGTACAGCGCCCGAATCGGACAGTGTAGAACCATTCTCTACTGCCTTCCTTCTGAGAACAGC
........CCCCCCC............................dddd........ccccccc......................DDDD.......
(((((((.................................................................................)))))))
>SNORD83B|chr22|39709824|39709916|-|::|::
GCTGTTCAGTGATGAGGCCTGGAATGTGCGCTGGGCACAGCGCCCGAGACAGACTGCGGAACCGTTCCTTGTTGCCTTCCTTCTGAGAACAGC
........CCCCCCC............................dddd......ccccccc......................DDDD.......
(((((((...............................................................................)))))))

>SNORD84|chr6|31508874|31508960|-|::|::
CGGCGGCCATATGATGTTTTCTTTTCGAAAGGTGAGCGCTTTGCGCAGTGATGACCCTCATCTATCACCCTTGACTGATGGCTGCTG
..........CCCCCCC..............dddd............ccccccc....................DDDD.........
(((((((((.....................................................................)))))))))
>SNORD86|chr20|2636740|2636831|+|::|::
GGGGATCACGGTGATGGCTGACCAGGGCTCCCTGACCTATACAGGCCTCTGCTATGGGGGTGATGGCCAGTCCTGGTGTCTGAGTGATTCCC
..........CCCCCCC..............dddd........................ccccccc.............DDDD.........
(((((((((..........................................................................)))))))))
>SNORD94|chr2|86362993|86363129|+|62:U6:d|::
CAGGCTGTGATGATTGGCGCAGGGGTACGGACCTCAGCTGAGTCATGGGAGCTGAATGTATGTGTTTCTCCTTTGTCCTGCATGTGGCAGGCTGATGGG
GAGCACTTACATGAGACTGTTGCCTCAATCTGAGCCTG
......CCCCCCC..............dddd............................................................ccccccc.
.............................DDDD.....
((((((.............................................................................................
................................))))))
>SNORD95|chr5|180670310|180670377|-|2811:28S:d|2802:28S:D

GGCGGTGATGACCCCAACATGCCATCTGAGTGTCGGTGCTGAAATCCAGAGGCTGTTTCTGAGCTGCC
....CCCCCCC..............dddd......ccccccc................DDDD......
((((((........................................................))))))
>SNORD96A|chr5|180668810|180668896|-|::|75:58S:D
AGAGGGTCCTGGTGATGACAGATGGCATTGTCAGCCAATCCCCAAGTGGGAGTGAGGACATGTCCTGCAATTCTGAAGGGATTCTCT
...........CCCCCCC.........dddd....................ccccccc..............DDDD...........
((((((((((...................................................................))))))))))
>SNORD96B|chrX|109468212|109468292|-|::|75:58S:D
GGATCCTGGTGATGACAGACGACATTGTCAGCCAATCCCCATGTGGTAGTGAGGACATGTCCTGCAGTTCTGAAGGGATTC
........CCCCCCC.........dddd....................ccccccc..............DDDD........
(((((((...................................................................)))))))
>SNORD112|chr14|101364257|101364331|+|::|::
TGGACCAATGATGAGACAGTGTTTATGAACAAAAGATCATGATTAATCCAGTTCTGCACAAAACACTGAGGTCCA
.......CCCCCCC.........dddd..........ccccccc.....................DDDD......
((((((...............................................................))))))
>SNORD4A|chr17|27049600|27049671|+|121:18S:d|::
GGTGCAGATGATGACACTGTAAAGCGACCAAAGTCTGAACAAAGTGATTGGTACCTCGTTGTCTGATGCACC
.......CCCCCCC....................dddd.....ccccccc............DDDD......
((((((............................................................))))))
>SNORD4B|chr17|27050699|27050772|+|121:18S:d|::
GGGTGCAAATGATGCATATGTTAGCGACCAAAGCCTGATCTTTGCTGATTAGTCATAATTAACTGACTGCACCC
........CCCCCCC...................dddd......ccccccc...........DDDD........
(((((((............................................................)))))))
>SNORD7|chr17|33900671|33900772|+|::|47:U6:D
CAGCGATGCGATGATGAGTGAAGTAGAGCCTGACCTGGTATTGCCATTGCTTCACTGTTGGCTTTGACCAGGGTATGATCTCTTAATCTTCTCTCTGAG
CTG
..........CCCCCCC.............................................................................DDDD.
...
((((..............................................................................................)
)))

>SNORD8|chr14|21865447|21865565|-|53:U6:d|::
ACATGTCCCAATGATGAGTTGCCATGCTAATACTGAGCCACCAGGTAGGGCAGTGTTGCCCTGGTTTGGGTGCCAGTGAGTTTAACAAAACTTCTCACA
TGAAGATCTGAGGGGCATGT
..........CCCCCCC...............dddd.............................ccccccc...........................
.......DDDD.........
(((((((((..........................................................................................
...........)))))))))
>SNORD9|chr14|21860308|21860414|-|53:U6:d|::
TGCCCCTGTGATGAGTTGCCATGCTAATACGGAGACACCAGGTAGGGAGTTTTACCCTAACTTGGGTGTTGTTGAAATAAACTCTTTCTCGTAAATGCT
GAGGGGCA
.......CCCCCCC...............dddd............................ccccccc.............................DD
DD......
(((((((............................................................................................
.)))))))
>SNORD10|chr17|7480127|7480272|+|3787:28S:d|::
CTGCTCTGTGATGGAGCCCATGCGTGTCATCTGAGCCTCTGGCTTCCCTGCCAGTGCAGCCCTGGCAGTGTCCTACTTCCCAGGGCTGTTGTCTGCCTG
GCGGGAAGGTCCTGGGCAAAGGATCAGTCTTTGTACTCTGAGAGCAG
.......CCCCCCC................dddd.................................ccccccc.........................
.....................................DDDD......
((((((.............................................................................................
.........................................))))))
>SNORD5|chr11|93466391|93466468|-|2409:28S:d|::
CAGTTCAGATGATGAATTTAACTGTTCAACTGCTGAATGATAACGGGCATGAACTAAAACTTAATTCTGACAGAGCTG
........CCCCCCC....................dddd........ccccccc............DDDD........
((((((..................................................................))))))

>SNORD6|chr11|93464669|93464739|-|2411:28S:d|::
GATGTTATGATGATGGGCGAAATGTTCAACTGCTCTGAAGGGGCTGAATGAAAATGGCCTTTCTGAACATC
......CCCCCCC......................dddd......ccccccc..........DDDD.....
((((((...........................................................))))))

```
>SNORD1A|chr17|74557718|74557785|+|4362:28S:d|::
AAGCCTATGATGGTTAGTTATCCCTGTCTGAAAATCTGGACTGAGGGAAATAATCTATTCTGAGGCTT
......CCCCCCC..............dddd.........ccccccc............DDDD.....
((((((..........................................................)))))
>SNORD1B|chr17|74557190|74557275|+|4362:28S:d|::
GCTGAGTCCATGATGATTTCAAGTTATCCCTGTCTGAAGGCAAAGAAAGGCCTTTCTGTGTGGAATTTGAATATCTGAAACTCAGT
.........CCCCCCC.................dddd....................ccccccc..........DDDD........
(((((((........................................................................)))))))
>SNORD17|chr20|17943353|17943589|-|3797:28S:d|::
GTGAAATGATGATTCAGTTTATCCATTCGCTGAGTGCGCTGCACTGACCTTCTTCCAAGCCTCAGTTCCTGTTCTAGGAACTTGAGGCTATGTAGCCTG
AAAATGCCCTGCAGTCTGCAGTGTTCTACTGTGAACTGCTTGTGTGTTGGCAGGCTACCGGTAAGAATGGTTGGTGTCAGCAGGGACGGGGCCCTCTGA
GACCCATCTCACAAAGATGAGTGGTGAAAATCTGATCAC
.....CCCCCCC.................dddd...................................................................
......................................................................ccccccc.......................
...............................DDDD....
((((................................................................................................
....................................................................................................
...................................))))
>SNORD19|chr3|52723259|52723323|+|::|683:18S:D
TGGGTGAGGTACGAGGAAACAGTCTGATAGTCACTGAAGACTGATTAGATCCAACTCTGATCTCA
...CCCCCCC.............dddd......ccccccc................DDDD.....
(((...........................................................)))
>SNORD23|chr19|48259106|48259223|+|::|::
CCCGTGCCCAGTGATGACACCATCCTTGCTCCCCGTGCCCCCCAGGGGCTATGGGCGACACCATGGCTGCCCCTGGGCTGGGCCAGTGGGGCCAATGCC
CAGGGGCTGAGGGCACGGG
..........CCCCCCC.................dddd........................ccccccc...............................
......DDDD.........
(((((((((...........................................................................................
..........)))))))))
>SNORD64|chr15|25230243|25230317|+|::|::
GGCTGGATTTGTGATGAGCTGTGTTTACTGAGCATGATGAAGTAAAGCTCAACGTGATTACTCTGAAGTCCAGCC
..........CCCCCCC..........dddd..ccccccc......................DDDD.........
(((((((((.........................................................)))))))))
>SNORD66|chr3|184043484|184043559|+|1272:18S:d|::
TTCCTCTGATGACTTCCTGTTAGTGCCACGTGTCTGGGCCACTGAGACACCATGATGGAACTGAGGATCTGAGGAA
.....CCCCCCC.............................dddd......ccccccc..........DDDD....
((((....................................................................))))
>SNORD67|chr11|46783938|46784050|-|::|60:U6:D
CTGTGAGAGTGATGAGTTGCACACTGGTGGAGCCATGGTATCAGGTGATACAGGCACCACTCAGTATCACCCTGGTGACAAAATCAAGTGCACAGGGGC
CATCTGACTCACAG
........CCCCCCC.............................................dddd........ccccccc.....................
...DDDD.......
(((((((.............................................................................................
.......)))))))
>SNORD88A|chr19|51302699|51302789|-|3680:28S:d|::
GGGCCTCCATGATGTCCAGCACTGGGCTCCGACTGCCACTGAGGACACGGTGCCCCCCGGGACCTTTGACACCCGGGGGTCTGAGGGGCCC
........CCCCCCC.............dddd......ccccccc...................................DDDD.......
(((((((.............................................................................)))))))
>SNORD88B|chr19|51302287|51302381|-|3680:28S:d|::
TGGGGACCCCGTGATGTCCAGCACTGGGCTCTGACTGCCCCTGAGGACACGGTGCACCCCGGGACCTTTGACATCCGGGGTTCTGAGGGGCCCCA
..........CCCCCCC.............dddd......ccccccc...................................DDDD.........
(((((.((((............................................................................)))))))))
>SNORD88C|chr19|51305582|51305678|-|3680:28S:d|::
CTGGGGCTCCCATGATGTCCAGCACTGGGCTCTGATCACCCCTGAGGACACAGTGCACCCCAGGACCTTTGACACCTGGGGGTCTGAGGGGCCCCAG
...........CCCCCCC.............dddd......ccccccc...................................DDDD..........
((((((((((.............................................................................))))))))))
>SNORD69|chr3|52726756|52726824|+|::|4464:28S:D
TGAAGCAAATGATGATAAACTGGATCTGACTGACTGTGCTGAGTCTGTTCAATCCAACCCTGAGCTTCA
........CCCCCCC..........dddd......ccccccc.................DDDD......
((((((.........................................................))))))
>SNORD70|chr2|203141160|203141235|+|::|512:18S:D
GTTGTCAATGATGTATTCTTCTTGGAACTGAATCTAAGTGATCTGACTCAATATTCGTCACTACCACTGAGACAAC
.......CCCCCCC.............dddd......ccccccc......................DDDD......
((((((................................................................))))))
```

>SNORD72|chr5|40832759|40832836|-|4590:28S:d|::
GCTTATCAGTGATGTTGTAAAAATAAATGTCTGAACATATGAATGCAGTATTGATTTCAGCATTTAACTGAGATAAGC
........CCCCCCC.......................dddd........ccccccc..........DDDD.......
(((((((................................................................)))))))

>SNORD87|chr8|67834703|67834789|-|3723:28S:d|::
CTGGCACAATGATGACTTAAATTACTTTTTGCCGTTTACCCAGCTGAGGTTGTCTTTGAAGAAATAATTTTAAGACTGAGATGCCAG
........CCCCCCC............................dddd........ccccccc.............DDDD........
((((((...........................................................................))))))
>SNORD89|chr2|101889395|101889514|-|::|::
GCTACTGAGGAATGATGACAAGAAAAGGCCGAATTGCAGTGTCTCCATCAGCAGTTTGCTCTCCATGGGCACACGATGACAAAATATCCTGAAGCGAAC
CACTAGTCTGACCTCAGTAGC
...........CCCCCCC..................dddd................................ccccccc....................
.......DDDD..........
((((((((((.........................................................................................
...........))))))))))
>SNORD90|chr9|125642494|125642596|-|::|::
GTTTTCTAAGTGTCTAATGATGAATTTCATAGGGCAGATTCTGAGGTGAAAATTTAATTCATCACTGATACTCCTACTGTGGAATCTGAAGACACTTGA
AAAC
................CCCCCCC.................dddd....................ccccccc..............DDDD..........
....
((((((.((((((((..........................................................................))))))))))
))))
>SNORD91A|chr17|2233575|2233662|-|4588:28S:d|::
GAGAAGTCAATGATGGTTTTATTCATATCGTCTGAACCTGTCTGAAGCATCTCAGTGATGCAATCTCTGTGTGGTTCTGAGACTTCTC
.........CCCCCCC.........................dddd.........ccccccc...............DDDD........
((((((((........................................................................))))))))
>SNORD91B|chr17|2232419|2232504|-|4588:28S:d|::
AAGAGCCAATGATGTTTTTATTCAAAATGTCTGAACCTGTCTGAAGCATCCCAGTGATGCAACTTCTGTGTGATACTGAGGCTTTT
........CCCCCCC.........................dddd.........ccccccc...............DDDD.......
(((((((........................................................................)))))))
>SNORD92|chr2|29136529|29136615|+|::|3846:28S:D
GGTGCTGTGATGATGCCTTAATATTGTGGTTTCGACTCACTGAGAGTAAAATGAGGACCTACAATTCCTTGGCTGTGTCTGAGCACC
......CCCCCCC..........................dddd.......ccccccc.....................DDDD.....
(((((.............................................................................)))))
>SNORD98|chr10|70514923|70515001|+|867:18S:d|::
AAGGATGAGTTATGATGTGTGTAAATCCTATTCCATTGCTGAAATGCAGTGTGGAACACAATGAACTGAACTCTTCCTT
...........CCCCCCC....................dddd......ccccccc..........DDDD..........
(((((.((((...........................................................)))).)))))
>SNORD100|chr6|133137941|133138016|+|436:18S:d|::
GCTGTACATGATGACAACTGGCTCCCTCTACTGAACTGCCATGAGGAAACTGCCATGTCACCCTTCTGATTACAGC
.......CCCCCCC................dddd......ccccccc..................DDDD.......
((((((................................................................))))))
>SNORD107|chr15|25227135|25227221|+|::|::
TGGCTAGGTTCATGATGACACAGGACCTTGTCTGAACATAATGATTTCAAAATTTGAGCTTAAAAATGACACTCTGAAATCCAGTCA
...........CCCCCCC.............dddd.....ccccccc..........................DDDD..........
(((((.((........................................................................)))))))
>SNORD109A|chr15|25287121|25287187|+|::|::
GGATCGATGATGAGAATAATTGTCTGAGGATGCTGAGGGACTCATTCCAGATGTCAATCTGAGGTCC
......CCCCCCC...................dddd....ccccccc...........DDDD.....
(((((.........................................................)))))
>SNORD109B|chr15|25523490|25523556|+|::|::
GGATCGATGATGAGAATAATTGTCTGAGGATGCTGAGGGACTCATTCCAGATGTCAATCTGAGGTCC
......CCCCCCC...................dddd....ccccccc...........DDDD.....
(((((.........................................................)))))
>SNORD93|chr7|22896232|22896305|+|::|576:18S:D
TGGCCAAGGATGAGAACTCTAATCTGATTTTATGTGCTTCTGCTGTGATGGATTAAAGGATTTACCTGAGGCCA
......CCCCCCC.........dddd.................ccccccc...............DDDD.....
(((((................................................................)))))
>SNORD54|chr8|56986398|56986459|-|::|644:18S:D
GGCGATGAGGAGGTACCTATTGTGTTGAGTAACGGTGATAATTTTATACGCTATTCTGAGCC
....CCCCCCC.............dddd......ccccccc..............DDDD...
(((........................................................)))
>SNORD26|chr11|62622765|62622838|-|389:28S:d|::

```
CTACGGGGATGATTTTACGAACTGAACTCTCTCTTTCTGATGGATTAGTGGAGAAAACAGAAAATTCTGAGTAG
.....CCCCCCC........................dddd.......ccccccc............DDDD....
((((..................................................................))))
>SNORD47|chr1|173833505|173833582|-|3866:28S:d|::
ACCAATGATGTAATGATTCTGCCAAATGAAATATAATGATATCACTGTAAAACCGTTCCATTTTGATTCTGAGGTTAC
....CCCCCCC..............dddd......ccccccc..........................DDDD......
..............................................................................
>SNORD99|chr1|28905263|28905333|-|::|2774:28S:D
CTGGTCCAGGATGAAACCTAATTTGAGTGGACATCCATGGATGAGAAATGCGGATATGGGACTGAGACCAG
.......CCCCCCC........dddd..............ccccccc..............DDDD......
((((((...........................................................))))))
>SNORD85|chr1|31441010|31441084|-|601:18S:d|::
GACTGGCAAGGATGATACACACTTGCCCTCACTTAGACTATAGTTCACTGATGAGAGCATTGTTCTGAGCCAGTC
........CCCCCCC................dddd............ccccccc..........DDDD.......
(((((((.............................................................)))))))


>SNORD108|chr15|25232070|25232144|+|::|::
TGGCTTAATGATGAGAATCATTATTTCTTGAATTGGATGACACTTTCCATTCCTGCAAAGGGAGCGTGAGGTCCA
.......CCCCCCC...................................................DDDD......
((((..................................................................).)))
>SNORD71|chr16|71792306|71792389|-|14:58S:d|::
GTGTGTTGGAGGATGAAAGTACGGAGTGATCCATCGGCTAAGTGTCTTGTCACAATGCTGACACTCAAACTGCTGACAGCACAC
.........CCCCCCC....................dddd..............ccccccc...........DDDD........
((((((((....................................................................))))))))
>SNORD110|chr20|2634852|2634938|+|1288:18S:d|::
CAGGCTTTGCAGTGATGACTTGCGAATCAAATCTGTCAATCCCCTGAGTGCAATCACTGATGTCTCCATGTCTCTGAGCAATGCCTG
...........CCCCCCC.........................dddd.........ccccccc..........DDDD..........
(((((.((((...................................................................)))).)))))
>SNORD111|chr16|70571907|70572002|+|3923:28S:d|::
ACAGCCTGAAATGATGACTCTTTAAAAAATTTCATGTCTCTTCTCTGACATTTTTCTCTGGACACAGTTTTTGCCTTATGAATCTGATCAGGCTGT
..........CCCCCCC...........................dddd.........ccccccc...................DDDD.........
(((((((((..............................................................................)))))))))
>SNORD111B|chr16|70563414|70563489|+|3923:28S:d|::
GCCTGAAGTGATGATTCACATTCATGTCTCTTCTCTGATAAATTCTTGAAGAAAATTTTTGTGTGTCTGATCAGGC
.......CCCCCCC....................dddd.......ccccccc..............DDDD......
((((((................................................................))))))
>SNORD27|chr11|62622484|62622555|-|18S:27:d|::
ACTCCATGATGAACACAAAATGACAAGCATATGGCTGAACTTTCAAGTGATGTCATCTTACTACTGAGAAGT
.....CCCCCCC......................dddd........ccccccc..........DDDD.....
(((.(..............................................................).)))
>SNORD118|chr17|8076771|8076906|-|::|::
ATCGTCAGGTGGGATAATCCTTACCTGTTCCTCCTCCGGAGGGCAGATTAGAACATGATGATTGGAGATGCATGAAACGTGATTAACGTCTCTGCGTAA
TCAGGACTTGCAACACCCTGATTGCTCCTGTCTGATT
......................................................CCCCCCC......................................
...............................DDDD..
...................................................................................................
.....................................
>SNORD1C|chr17|74554872|74554951|+|28S:4362:d|::
GCTGAGCTGAGGATGATTTAAAGTTATCCCTGTCTGAAATGGTATCTTTTGTGAGGAGGTCTGACTTGCTGAGGCTCAGC
.........CCCCCCC.................dddd.............ccccccc...........DDDD........
((((((....................................................................))))))
>SNORD2|chr3|186502585|186502653|+|28S:1509:d|::
AAGTGAAATGATGGCAATCATCTTTCGGGACTGACCTGAAATGAAGAGAATACTCATTGCTGATCACTT
.......CCCCCCC................dddd......ccccccc............DDDD......
((((((.........................................................))))))
>SNORD22|chr11|62620383|62620507|-|::|::
TCCCAATGAAGAAACTTTCACATGTCTTACTCTCTGTCCTAGTCCCAGAGCCTGTAAAGGTGAACCCACTGGGACTGGCTGGGGGAGAAGAGGAAGATT
TGTTCCAGAAGGAACTGTCTGAGGGA
.....CCCCCCC.......................................................................................
..................DDDD....
((((...............................................................................................
......................))))
>SNORD30|chr11|62621135|62621204|-|28S:3804:d|::
```

```
GTTTGTGATGACTTACATGGAATCTCGTTCGGCTGATGACTTGCTGTTGAGACTCTGAAATCTGATTTTC
....CCCCCCC.....................dddd..........ccccccc......DDDD.......
......................................................................
>SNORD53|chr2|29149933|29150010|+|::|28S:3848:D
ATGCTATGATGACATCCATATGGTTTCGCTGCTGGCTGAGTTTCAGAGATGACACCTTTCTCTTGGCTGTCTGAGCAT
.....CCCCCCC.......................dddd......ccccccc..................DDDD....
((((......................................................................))))
>SNORD63|chr5|137896733|137896799|-|28S:4541:d|::
GTGCAATGATGTATTTTATTCAACACATCATTCTGAAAGAACGTGTGGAAAACTAATGACTGAGCAC
.....CCCCCCC....................dddd......ccccccc..........DDDD....
((((...........................................................))))
>SNORD65|chr17|16344534|16344617|+|18S:627:d|::
GATATCAAATGATGAAATCACCCAAAATAGCTGGAATTACCGGCAGATTGTGTAGTGGTGAACCTATGGTTTTCTGAAGATATC
........CCCCCCC............................dddd.......ccccccc............DDDD.......
((((((........................................................................))))))
>SNORD68|chr16|89627833|89627914|+|18S:428:d|::
TCAGTCGCGTGATGACATTCTCCGGAATCGCTGTACGGCCTTGATGAAAGCACATTTGAACCCTTTTCCATCTGATTGCTGA
........CCCCCCC...............dddd......ccccccc........................DDDD.......
((((..........................................................................))))
>SNORD74|chr1|173836810|173836882|-|28S:3820:d|::
TGCCTCTGATGAAGCCTGTGTTGGTAGGGACATCTGAGAGTAATGATGAATGCCAACCGCTCTGATGGTGGCA
.....CCCCCCC.....................dddd.....ccccccc............DDDD........
((((.................................................................))))


>SNORD79|chr1|173834486|173834570|-|::|::
TACTGTTAGTGATGATTTTAAAATTAAAGCAGATGGGAATCTCTCTGAGAAAGAAAATGGAGATTAATCTTAAACTGAAACAGTA
........CCCCCCC...........................................................DDDD.......
(((((...........................................................................)))))
>SNORD80|chr1|173833967|173834044|-|28S:1521:d|28S:1612:D
GATACAATGATGATAACATAGTTCAGCAGACTAACGCTGATGAGCAATATTAAGTCTTTCGCTCCTATCTGATGTATC
......CCCCCCC.............dddd.........ccccccc......................DDDD......
(((((....................................................................)))))
>SNORD97|chr11|10823014|10823155|-|::|::
TTGCCCGATGATTATAAAAAGACGCGTTATTAAGAGGACTTTATGCTGGAGTTCTTGACGTTTTTCTCTCTTTTCTATACTTCTTTTTCTTTCTTTGAA
TGTCCAGCGTCCTGTGAGCGAAGATTATGAGATATGAGGGCAA
.......CCCCCCC.....................................................................................
.................................DDDD......
((((((.............................................................................................
.....................................))))))
>SNORD56|chr20|2637266|2637343|+|18S:517:d|::
GCTTCCACAATGATGGCAATATTTTTCGTCAACAGCAGTTCACCTAGTGAGTGTTGAGACTCTGGGTCTGAGTGAAGC
.........CCCCCCC................dddd..........ccccccc..............DDDD.......
(((((....................................................................)))))
```

## H/ACA box snoRNA annotation

```
>SNORA1|chr11|93465170|93465299|-|::|::
TGCCTCATTCTAGAGAATGGGCACTGTTGATCATGGTGTCCAAAAATAGTTAATGTGGCTAAATTGAGACAGGTTATGCTTCCATCACAGTATGCATAT
TGCAGTGGTGACAATGAGACCTGTAACATTT
............................................................HHHHHH..................................
.........................AAA...
.(((.(((((((.....((((((((((.....))))))))))....)))..)))).)))..........((((((.........((((((.(((.......
))).))))))........))))))).......
>SNORA10|chr16|2012335|2012467|-|::|::
GGTCTCTCAGCTCCGCTTAACCACACGGGTCCAGTGTGTGCTTGGCGTGTTTTCAGGGAGGCAGAGAAAGGCTCTCCTAATGCACGACAGACCCGCCCA
GAATGGCCTCTCTGTTCCTAGGAGTGCGACAATT
..............................................................HHHHHH................................
............................AAA...
.((((((((((..(((.....(((((.......))))).....))).)))....)))))))..........((.((((((.......(((((...(((..
....)))...)))))...)))))).)).......
>SNORA13|chr5|111497182|111497314|+|::|::
AGCCTTTGTGTTGCCCATTCACTTTGGAAACTAGTGAATGTGGTGTCAAAAAAGGCGTAAATTAAACGCTTTGCAGCCTTTTCCTGCCCTTAAATTTGA
TACCTTTGGTGTAGGAGCTGCATAAGTAACAGTT
..........................................................HHHHHH....................................
............................AAA...
.((((((...(((((((((((((.........)))))))).))...))).))))))............((((((((((....((((((((..........
.......)).)))))))))))).)))).......
>SNORA14A|chr7|75573101|75573234|+|::|::
TGCATTCTTAAACCCTCTTGGTGGCTTCCCTGTAAATGCTTCCAAGATATGAGCGAATGCTATAGAAATTGCAGGAAAGTCCAAAGGGCTGCGCGTCTC
CTGTGGCTCAGTCTTATTTCATACCTGCAACATCT
.............................................................HHHHHH.................................
.............................AAA...
.((((((........((((((..((............))..))))))........)))))).........((((((.........(((((((.((.....
.....)).)))))))........))))))......
>SNORA14B|chr1|235291118|235291252|-|::|::
CTGCATTCTTAAACCCTCTTGGTAGCTTCGTTCTAAGTGCTTCCAAGATATGAGTGAATGCTATAGAAATTGCAGGGGAGTCCAAAGGGCTGCGCTTCT
CCCGTGGCTCAGTCTTATTTCATACCTGCGACATCT
..............................................................HHHHHH................................
..............................AAA...
..((((((........((((((.(((.............))).)))))).......)))))).........(((((((((((....(((((((.((....
......)).))))))))))))...))))))......
>SNORA15|chr7|56128163|56128295|+|::|::
GCATGGCCGAATACTGTGTTTTTATCAGTAGTTTACACAGCCAGACACCATGCAAAAGCAGTCTTCCCTTTAGAATGACTGATGGTATGCTAAGGTTTT
TCATAGCATATCATTATTAAAGGTGAATACAAAT
......................................................HHHHHH........................................
............................AAA...
((((((.......((((((...............)))))).......))))))...........((((((((((.......((((((((((((.......
...)))))))))))).))))))).))).......
>SNORA16A|chr1|28907432|28907565|-|::|::
TTGGCCCTTATCGAAGCTGCAGCTGCTTCCGCATAGCTGCTGTGGTCAAAAAGGAGCCCAGAGTGACAGTTTTCCTTGACGGTCGCCGTTCTGTTTGTT
GTAACTGATCTGCAACATTTTGGGAAAATACAGTT
...........................................................HHHHHH...................................
.............................AAA...
..(((((((...((..(((((((.(((.......))).)))))))...)))))).)))..........(((((((.........................
......................)))))))......
>SNORA17|chr9|139621199|139621331|-|::|::
ACTGCCCCTAGAGGCGTTGCAGCTGTGGCTGCCGTGTCACATCTGTGTCATTAGGTGGCAGAGATTAGAGAGGCTATGTCTACGCTCAGCGTTCTGCCC
CGTGAACGTTTGAATGTTTGATAGTCTCACACTC
.............................................................HHHHHH.................................
............................AAA...
.(((((((((..(((....(((.((((((......)))))).))).)))..)))).)))))........((((((((....((..(((((((((......
...))))))).))..))...))))))))......
>SNORA18|chr11|93466632|93466763|-|::|::
GTTGAGGTCTATCCCGATGGGGCTTTTCCTGTAGCCTGCACATCGTTGGAAACGCCTCATAGAGTAACTCTGTGGTTTTACTTTACTCACAGGACTATT
GTTAGATCTGTGGGAAGGAATTACAAGACAGTT
............................................................HHHHHH..................................
...........................AAA...
```

```
..(((((....((((((((.((((........))))....)))))..)))....)))))........((.(((((((...((((.(((((((..(((..
..)))..))))))))))))))))))))......
>SNORA19|chr10|120819523|120819650|-|::|::
GTGCACATTTCATTGACCTGCTTTCTTTTATGTGAGTAGTGTTATTTCTTATGTGCTATACAAATAATTGAAGGCTAATTAGCAGTATAACTATAAATA
GTAATGCTGCCAGTCTCCTTCAGACAAAA
.........................................................HHHHHH.....................................
.......................AAA...
..((((((.....(((((((((.............))))).))))......))))))...........((((((...(((.((((((((.((((....))
)).))))))).)))..)))))))......
>SNORA20|chr6|160201282|160201413|-|::|::
CTTCCCATTTATTTGCTGCTTGTAGTCTCACAGTGATACGAGCAGTTATACGCATGGGATAAAATAACATTGGGCCACTGTAAATTGAGATGAAGTAAC
CATTTTCATCTCTTCTGCAGGGACTAGACATTG
............................................................HHHHHH..................................
...........................AAA...
..((((((.(((...(((((((((.((.......)))))))))))..)))...))))))...........((((.((.(((((....((((((((.....
....))))))))...))))))).))))......
>SNORA21|chr17|37009116|37009248|-|::|::
CCCCCTTTTAAAAGCACTCAATGGGCCTGTGGCTAATGACCTATTGAGCCGTCAAGAAAGGGGAGAGTGAAAACATCGCTTTTGGGTGAAGTGGCAACA
TGTGTTGTTTGCTTCAATCGGTGGTGTGACAAGG
...............................................................HHHHHH...............................
............................AAA...
.(((((((........(((((((((..............))))))))).......)))))))..........((((((........(((((...(((((.
...))))....)))))....)))))))......
>SNORA22|chr7|65220513|65220646|+|::|::
TTGCACAGTGAACACCCAAGTGTGCTTTATAGTTCCCTTGGCTTTGACCCTGTGCTAGAGCATTGCCTGCTCTTCTCCTCTGCATTAAAAGGAATATTT
ATCCTTTTAAATGTATTCAGAAAGCCAGCACATTA
........................................................HHHHHH......................................
.............................AAA...
..((((((.......(((((....((....))....)))))........))))))..........((..(((.((((....(((((((((((((.......
.))))))).))))))...)))))))..))......
>SNORA23|chr11|9450313|9450501|+|::|::
CATGGCTGCTGTAATGTGTGCATAGGTTTCATCTGTGTCTGGTAGCAGTGTCTGTCTGTGTTTTTCATTCAGATCTTGCTATCCACACAAACATCATGC
GGCCAAAGAGTAACTTGGGATCATAGTACTGGTCTAGTGTTGTCTCTGGACACATCTACCACTGGCCAGCCTCCAAATTCCCACACACAG
....................................................................................................
......HHHHHH........................................................................AAA...
..(((((((((...((((((................................................................))))))......)).))
)))))..........(((((........((((.((((((.((((....))))......))))))))))..........))))).......
>SNORA24|chr4|119200345|119200475|+|::|::
CTCCATGTATCTTTGGGACCTGTCAGCCGTGGCAGTCTCCCTTCCTAGCCATGGAAGAGCATATCCTTGTTTATTGGCAAAGCTGTCACCATTTAATTG
GTATCAGATTCTGACTTGCACAAGTAACATTC
.......................................................HHHHHH.......................................
..........................AAA...
.((((((.......((((.((((((....))))))..))))........))))))..........(((((............(((..((((......))
))..)))............)))))........
>SNORA25|chr11|93463679|93463812|-|::|::
GGGTCATTTCAAAGAGGGCTTATGAGGCTGTGAAACCCAGAGCTCTTAACGCTGTGACCAAAGATGGAAGTTCTCTATAGGAAGCCATAGCACTCCTAA
TGTTTGGTGCTATGTTTTCCTGAGGAGATATAAAA
.............................................................HHHHHH.................................
.............................AAA...
.((((.......(.((.(.(((.(((.((.((.....)).))))).)))).)).)))))............((((...(((((..((((((((......
......))))))))..)))))...)))).......
>SNORA27|chr13|27829538|27829663|+|::|::
TACCCCCTTTTCACTTTGCCAGTTGGACTTATGTCTTTATTGGTCATTCAAGTGGGGCAAAGGAAATATCCTTTTAAAACTCAGGCAAACTGGGTGTTT
GTCTGTATCCTGTCAGAGGAAACAAAT
..........................................................HHHHHH....................................
.....................AAA...
..(((((((.......(((((((..(((....)))...))))).))...))).))))...........((((((.......(((((((((.....))))
)))))..........))))))......
>SNORA28|chr14|103804186|103804312|+|::|::
AAGCAACACTCTGTGGCAGATGATCAAAACTGTCTGACACAATTTGAGCTTGCTATAGCAAGAAAGTCTAACCTATTCCGGTGTTCTCTCTCCCATGAG
ACAAGCCGTTATATAGACTTAAACAGTG
......................................................HHHHHH........................................
......................AAA...
.(((((..(((((((.((((............)))).))))....))).)))))........((((((...(((..((((((.(((........)))
```

)))..)))..))))))))))........
>SNORA29|chr6|160206626|160206765|-|::|::
TTTCTCATTTGACTACCACATTTTCTCCTAATAATAGATTTTAGTGGCTATGCTTATGGGATAGATTAAACTTGCCATGATCTGAAGAGGGAGGGTTTT
TTCATGTCCTCCTCCTATATGAAATGGCTGAACGGATATTA
...............................................................HHHHHH...............................
..................................AAA...
..((((((.......((((....(((.........))).....))))........))))))............(((((..((...((..(((((.....
.......)))))..))....)).))))).............
>SNORA2A|chr12|49050431|49050565|-|::|::
TAGGCCCTGAATCAAGACCAATGGTTTGCTGTAGCTGTTGGTTTCAAACAGGAGCTAAGAGTGATGTCTTCCTTGTGGTCTGTTGGCTATTCAGTATTC
CAGTGCGAATTGCCAATTCAGTTGGAAGAAACATAG
..........................................................HHHHHH....................................
.............................AAA...
..(((((((.....((((((((((((......))))))))))))....)))).)))..........((((((.......((((((((.((((.(((...
...))))))).)))))..)))..)))))).......

```
>SNORA2B|chr12|49061240|49061376|-|::|::
TTGGCCCTGAATCAAGGCCAGCAGTTTGCTGAAGCTGTTGGTTTCAAGCAGGAGCCTAAAGAATTGTCTTTCTATGGTCTGTTGGCCATTTCATAACTT
TGGAAATGTAATGGTCAATTCATTAGAAAGAAACATGA
.........................................................HHHHHH.....................................
................................AAA...
..(((((((.....(((((((((((((....)))))))))))))....)))).)))...........((((((((.......(((((((((.(((.....
.....))).))))))))).....))))))))).......
>SNORA3|chr11|8705774|8705903|+|::|::
ATCGAGGCTAGAGTCACGCTTGGGTATCGGCTATTGCCTGAGTGTGCTAGAGTCCTCGAAGAGTAACTGCTGACCTTATTCACTGGCTGTGGGCCTTAT
GGCACAGTCAGTCACCAGGTTAGAGACATGC
...........................................................HHHHHH...................................
.........................AAA...
.((((((((..((.((((((..((((........))))..))))))))..)).))))))........((.(((((((.....(((((((((..(((....
))))))))))))....)))))))))......
>SNORA45|chr11|8706986|8707116|+|::|::
GCCGAGACTAGAGTCACATCCTGACACAACTCTTGTCCTGGTGTGCTAGAGTACTCGAAGAGAATCTACTGGTCTTGATTCACTGGTGGGGGCAGTCGG
TGCCCCCGTTAGTGCCCAGATCAGAAACATAC
..........................................................HHHHHH....................................
..........................AAA...
..(((((((..((.((((((..((((.......))))..))))))))..))).))))............(((((((.....((((((((((((((.....
))))))))))))))...))))))).........
>SNORA30|chr16|30721858|30721986|+|::|::
CTGGCACTTTCACAGTTCCTTCCCCAGGCAGTGGGGCCAGGATTTGGTAGCTGGTGCTGAGAGAAAACCCTTGATTGTATTCTTGCCCTGGGATTATAC
CAGTGGCAACTGTCACTCAATGGGACAGTG
...........................................................HHHHHH...................................
........................AAA...
..(((((.....(((((((.(((...(((......))).)))...)).))))))))))..........(((((((.((.....(((((((((.......)
))).)))))....)).)))).)))......
>SNORA31|chr13|45911615|45911744|-|::|::
CTGCATCCACTGATAGACCTTGAACAATTTACTGTTGTTCTTTTGGTTTGCACTAGGATGCAAAAGAAAGAAATCCCTGCGCTTTCTGTCTGTCTTTGT
GGCGGCCCAGATTGAATTGGGGAATACATCT
..............................................................HHHHHH................................
.........................AAA...
.(((((((......(((((..((((((.......))))))....)))))......)))))))............(((........((((.(((((.....
)))))..)))).......)))).........
>SNORA32|chr11|93464145|93464265|-|::|::
TGGTCATTACCAAGGCTTTTAGAATGCAGTTTCTCATTTGCTGTGGACATGACCATAAAAAAATTTCCCAGTAGGTTTTCTATCTGCTACTTTGCTAGC
AATCAGCTTATTGGGAACAGTT
........................................................HHHHHH......................................
................AAA...
(((((((..(((..((....((((......)))).....))..)))..)))))))...........((((((((((((.......(((((......))))
)...))))))))))))......
>SNORA33|chr6|133138358|133138490|+|::|::
AAGCCAGCCAATGAATCTGCTTACCTGATTGTGTTTGTGCAGACATACTTTAAAAACTGGCAATAGTAAAGCCATGTTACGAGCCTTAAGGACATTGAA
GTCGTTAAGGTCCCTGAGAATGGCTATAACAAAT
..............................................................HHHHHH................................
............................AAA...
..(((((....(((((((((..((........))....))))).....))))...)))))..........((((((.(((.((.((((((.(((......
))).)))))))))..)))..))))).........
>SNORA34|chr12|49048165|49048301|-|::|::
GTGGCCCTGACTGAAGACCAGCAGTTGTACTGTGGCTGTTGGTTTCAAGCAGAGGCCTAAAGGACTGTCTTCCTGTGGTCTGTTGGCTGTTCTGGGACC
TCAGTAGGGAATGGCTATTTCATTTGGAAGAAACAACC
..........................................................HHHHHH....................................
................................AAA...
..(((((((.((..((((((((((...........))))))))))..))))).))))...........((((((..........(((((((((.....((
(....))))))))))))........)))))).......
>SNORA36A|chrX|153996803|153996934|+|::|::
TTCCAAAGTGTTGAGTTCAGTCCAGGGCAGCTTCCCTGTTCTGTTAATTAAACTTTGGGACATTAAAATGGGCTAAGGGAGATGATTGGGTAGAAAGTA
TTATTCTATTCATTTGCCTCCCAGCCTACAAAA
...........................................................HHHHHH...................................
...........................AAA...
.((((((((.((((...(((..(((((......)))))..)))....))))))))))))...........(((((..(((((.....(((((((((....
...))))))))).....))))))))))......
>SNORA36B|chr1|220373888|220374018|-|::|::
```

TTCCAAAGTGTTAAGTTCAGTTCAGGGTAGCTTCCCTGCTCTGTTAATTAAACTTTGGAACATTGAAACTGGCTAGGGAAAATGATTGGATAGAAACTA
TTATTCTATTCATTTATCCCCAGCCTACAAAA
...........................................................HHHHHH..................................
..........................AAA...
.((((((((.((((...(((..(((((......)))))..)))....))))))))))))...........((((.((((...(((.(((((((((....
...))))))))).))))))).)))).......

```
>SNORA37|chr18|51748654|51748782|-|::|::
TGAGCACTTTCACAGGTCCTCCCTCAGGCTGTGGGGCCAGGATTTGGTAGCTGGTGCTGAGAGAAAACCTTTGTTGGCATCCTTGCCCTGGGACTGTGC
CAGTGGCAGCTGTCATTCAATGGGACAATT
...........................................................HHHHHH...................................
........................AAA...
..(((((.....((((((((.(((((.....)))))..)))))))).......)))))..........((.(((.(((((..((.(((((((.......)
))).))))).)))))..))).)).......
>SNORA38|chr6|31590856|31590987|+|::|::
TCCTCCTACAAAGGCGTGTCTGTGGTTCCCTGTCTTTGGACACGTAAGAATTGGAGGAAAATAAATGTGGATTTGGGAAACTTTGAGGCCAGCTTGCTT
CTTGCAGGCTCATGATCAACCAATCTCACATAA
..........................................................HHHHHH....................................
...........................AAA...
((((((........((((((..................))))))........)))))).................((((....((((....(((((((..
...))))))).....))))....))))......
>SNORA39|chr20|37076726|37076861|+|::|::
CGCCTGCATTCGTAAGTGATCACGGGCTGCCCGTGTCCTGGTCATTGGTAGTGCAGGCAGAGGAAATGCGGGAAAGGTTGCTGTGTTTGGAGGGTCCAC
ATCTTCACCCTCCTGTCCCAGGAGCTTTCCTACACTC
..........................................................HHHHHH....................................
...............................AAA...
.(((((((((....((((((((.((((.......))))))))))))...)))))))))............((((((.(((.(((.....(((((((....
......))))))).....))).)))))))))......
>SNORA4|chr3|186505402|186505539|+|::|::
TACCAAAAGTTAGCTTTTTGGGGGGCAGGTTTTTAAGTAACCTTTGCCAACTTGGGCTATTTGGAAGAGTAAAAGACCACACTCCACAGTGGGCTATAC
CACTTAGTATAGTTCGCTACTATTTTGTGGCCTACATGA
.................................................................HHHHHH.............................
.................................AAA...
..(((((...((((......(..((((((((.......))))..))))..)....)))))))))..........((.(((((......((.(..((((((
......))))))..).)).......))))).))......
>SNORA40|chr11|93468276|93468402|-|::|::
TGCACTTATGTATGTTTTTGTTTAACGTGGACAAAGACTTACAGATAGGTGCAAAAAATAAATCCTCTTTTGCAACCCAGAACTCATTGTTCAGTATGA
GTTTTGATACATATAAGAAGGGATATTA
......................................................HHHHHH........................................
......................AAA...
(((((((((((.((((((((((((...)))))))))))).))))..)))))))..........(((((((........((((((((((........))))
))))))........))).))))......
>SNORA41|chr2|207026952|207027083|+|::|::
TTCCACAGCTACTGGTCTGCAGCTGTTCTTATGGTAGCAGTTGTGGCATTCCTCTGTGGGAAAGAAACTGTTAACACAAACACCTCTTTCTTAGCAAAA
CAGAAAGTGGGTATATATGTGTGACAGACACAA
............................................................HHHHHH..................................
...........................AAA...
.(((((((.....((.((((((((((((........))))))))))))....)).)))))))......((((((.((((.((.((.((((((........
.)))))).)))).....))))))))))......
>SNORA42|chr1|155889700|155889836|-|::|::
GCATGGTAATGGATTTATGGTGGGTCCTTCTCTGTGGGCCTCTCATAGTGTACCCATGCCATAGCAAATGGCAGCCTCGAACCATTGCCCAGTCCCCTT
ACCTGTGGGCTGTGAGCACTGAAGGGGGTTGCACAGTG
..............................................................HHHHHH................................
................................AAA...
..((((((.(((..(((((..((((((........))))))..)))))....)))))))))..........((((((((....((.((((((((((.(..
....).)))))).).))).))...))))))))......
>SNORA43|chr9|139620556|139620691|-|::|::
GCTGTCCTGGACCTGTTGGCACCACAGACAGTTGCTCTGCTGTGCCTGTGGCCTCGGGGCAAAGAGAAAGTGGCGATTTCTACACTCAGTGCTCGGGAA
CCAGTGGGCACTGAGAATGGTTTATGGCCTGACATTA
..............................................................HHHHHH................................
...............................AAA...
..(((((((..((....(((((..((((.......))))..)))))...))...)))))))...........(((.((..(((..((((((((((.....
.....))))))))))..)))...)).)))........
>SNORA44|chr1|28906893|28907024|-|::|::
CAGCATGTTTCCAAGGGCTGTGGCTGGTCATAGCCATGGGATCTCCAACTGCATGCAAGAGCAACCTGGAAAGACTTTGACAGCGCAGGTCAGTACAAT
ACCTGCAAGCTGCCACTCAGCTTTCCTATAATG
.........................................................HHHHHH.....................................
...........................AAA...
..(((((.(((((..(((((((......))))))).)))))...........)))))...........((((((.((.((.((((((((((.........
))))))..)))).))...))))))))).......
>SNORA46|chr16|58582403|58582537|-|::|::
```

AGCACTATATTTAAACCTGTGGATGGGAATATTCCCCATTCTTGGTTACGCTGTAGTGCAAAAGAATTCCTGGCTCTCTGTTGCACAGCTGACTTGTGC
CATTCTGCTGTTGCTGTATAGAGTTAAGGAACATGG

...........................................................HHHHHH..................................
..............................AAA...

.((((((((....((((...(((((((.......)))))))..))))....))))))))........((((....((((((.(((((((.((.......
...)).)))).)))...))))))...))))......

```
>SNORA48|chr17|7478031|7478165|+|::|::
TGTCCCTGACCTGGGTAGAGTGGCATCTGGTTGGTGATGCCCATCTCATATCAGCCAGGGACAAAGCAACTCCTTGTTCATCCCAGCTTGGCTTTTGAT
CCGTGCCCATGCCTGGTTCATGCCTTGGACACATAG
..............................................................HHHHHH................................
..............................AAA...
.(((((((..((.(((((((.((((((........))))))...))).)))))).)))))))............((((.........(((...(((.
((..((....))..)).))).))).)))))......
>SNORA49|chr12|132515769|132515905|+|::|::
CTTCCTCAGCCTTACTCCAGGGACTTTTTGTTGCCTGTAAAGTGCTCTGGCATTGCCTGAGGATAGATGAGAAAGCACATATCCCTCCCCAGTAAGACG
CTGTTTTCTTTTGGGGCCTACAAGTTGAGCTGACAGTA
................................................................HHHHHH..............................
................................AAA...
..(((((((.......(((((((((((...........))))).))))))......))))))).........(((.((.......((((...((((..
......))))..)))).........)).))).......
>SNORA5A|chr7|45143948|45144081|-|::|::
TGCAGCCGTGTCAAATTCAGTACCTGTCCTATGCATGGTAGGCACTGGCCCAGAAGGCTGCCACAGAAACACTGTGACTCATGGGCCCTGTTCCTGTGT
CCCAGGCTCAGGGATAAATTTGGTTACAGACATCA
..............................................................HHHHHH................................
.............................AAA...
.((((((...((.....((((.((((((........)))))).)))).....)).))))))..........(((((((.((....(((((..((((...
..))))..)))))).......)))))))))......
>SNORA50|chr16|58593700|58593835|-|::|::
AAGCACTGCCTTTGAACCTGATGTGTCTTGTTTGTAGCTTCACGGGCCAAGCAACAGTGCTAGAGCATAACGACTTGTTATAACTGGGGCTCTTCAGCT
CTCAACTGAACTGCTCTTTTAAAAACAAGGTACATTT
.............................................................HHHHHH.................................
...............................AAA...
.(((((((.....((((..((....))..))))...((((........))))..)))))))............((((((.......((((..(((((..
.....)))))..)))).......))))))........
>SNORA51|chr20|2635713|2635844|+|::|::
GGCCTCCTGGTGCTTACCACAGGCTGTGTTCTTACACTGACTGTATAGAAAGAGGAGGTAGAGTAAACCTACCCCATATACACCTCAGCTCAGGCCCTG
TGCCTGGTCTGTATTGTGAATGGGGGAACATAG
...........................................................HHHHHH...................................
...........................AAA...
.(((((((....((....((((...((((....))))...))))..))....)))))))...........((((((..(((...(((..(((((....
.)))))..)))...)))..))))))........
>SNORA52|chr11|811681|811814|+|::|::
TGGTCCATCCTAATCCCTGCCGGTCCATCTGTGGCCTGCCAGGTTTCGCTTGTGGACCAGAGCACCCTAGAAGCCTCACCCGAGGAGTGAGCAGGGCTC
CAGTGGGCTCACGTCATGGGCACTTCTAGACACTC
..........................................................HHHHHH....................................
.............................AAA...
.((((((........((((.(((.(((....))).))).)))).........))))))........(((((((.............((((...((((((
....))))))...)))).....)))))))......
>SNORA53|chr12|98993413|98993662|+|::|::
AACATGCTTCCTTAGATCCACCTTTGTGGATGAATCTTGAACTGAGTTCCACTTGTAAACTTCTTGTTTCTTGTGGTTCCAGTAGTCAAAGAAACATCC
AGCAACTTTTTTGGTTGTATAGTCAAAGGTGCTTGAGTCATTGGCATGTAAGAGAAATATACCTGCATGTTAGTCTAACGTTCTGATAGAAATGACATG
CATTTATGCTGCCATTTGTTACTATCAGGACTCGACTCGTGTGCGGACATTT
...........................................................................................HHHHHH......
....................................................................................................
..............................................AAA...
....................................................................................................
....................................................................................................
....................................................
>SNORA54|chr11|2985001|2985123|-|::|::
GAGCACTGTTCGTAACCCGTTAGCCTGGCTGTAGCTAATGGGTTCCATTCCGGTGCAATAGCATTTCCAGCGACACATGACTGACTGACTGGTGGCTTT
CAGTTTCAGGTCTTGGAGACAAAT
.........................................................HHHHHH.....................................
..................AAA...
..((((((.....(((((((((((.........)))))))))))......)))))).......((((...(((...(((...(((((..........)
)))).))).)))..)))).....
>SNORA55|chr1|40033046|40033182|-|::|::
GAGCACCTGAATCTTTCCCATTCCTTGCTGCCTCGTGCCGGTGTGGGGACAGATGGTGCTACAGAATGAGCAGAGGAAATCCAGACAGGTTGTTTTCCA
TTTGTCTTGGGGCCTGTCTCTACAGCTCTGCCACATTT
..............................................................HHHHHH................................
................................AAA...
```

.((((((...((((((((((..((..((........)).))..)))))).))))))))))........((((((....(((((((((((........)
)))))).)))))..((((....))))))))))).......
>SNORA56|chrX|154003273|154003401|+|::|::
CGGCAGACAGTTATCCCTTTCTAGTCTGGCTCGTGGGACTCTAGAGGGAGTCAGTCTGCAACAGTAAGTGGTGAGTTCTTCTGTCCAGCGTCAGTATTT
TGATGGTGGCTTTAGACTTGCCAGATAACA
............................................................HHHHHH..................................
........................AAA...
..((((((.....(((((((..((((..........))))..)))))))....))))))........(((..(((.((.....(((.(((((......
))))).)))....)))))..)))).......
>SNORA58|chr3|131197941|131198077|-|::|::
GGGCATACTCGTAGACCTTGCCTGACTGTGCTCATGTCCAGGCAGGGGGGACAGTGTATGCAAGAATAATTTGGAGTTCCTGCCAGCTCTAACCAGCTT
CATCAGTGGCTGGATAAATTGCAGGACTCTAAACATTT
..............................................................HHHHHH................................
................................AAA...
..((((((..((...(((((((((((((....)).)).)))))))))...))...))))))........(((((((.(((((......((.(((((..
........))))).))....))))))))))))......
>SNORA59A|chr1|12567300|12567451|+|::|::
GCCCAGGGTATGTTCACGGGGCGATGCTGCCCTCCCAGCTGGCCCATGGGTGACCCTGGGAACATTAACTGCCTCACAACGTTTGTGCCTCAGTTACCC
GTAGATGTAGTGAGGGTAACAATACTTACTCTCGTTGGTGATAAGGAACAGCT
.............................................................HHHHHH.................................
...............................................AAA...
.(((((((.....((((.((((...((((......))))..))))....)))))))))))........((...((((((((...((......(((((((
.............)))))))........))...)))).))))..))........
>SNORA59B|chr1|12567300|12567451|+|::|::
GCCCAGGGTATGTTCACGGGGCGATGCTGCCCTCCCAGCTGGCCCATGGGTGACCCTGGGAACATTAACTGCCTCACAACGTTTGTGCCTCAGTTACCC
GTAGATGTAGTGAGGGTAACAATACTTACTCTCGTTGGTGATAAGGAACAGCT
.............................................................HHHHHH.................................
...............................................AAA...
.(((((((.....((((.((((...((((......))))..))))....)))))))))))........((...((((((((...((......(((((((
.............)))))))........))...)))).))))..))........
>SNORA5B|chr7|45145567|45145698|-|::|::
GGCAGCCATGTCAAATTCAGTGCCTGCCCTGTCTATGGTAGGCACTGGCCCAGAAGACTGCCACAGAAACAGTGACTCACAGGCCCTGTTACTGTGTCC
CAGGCTCAGGGATAAATTTGGTTACAGACACCA
..............................................................HHHHHH................................
...........................AAA...
(((((.....((.....(((((((((((........))))))))))).....))...))))).........(((((.((....(((((...(((.....
)))...))))).......))))))).......
>SNORA5C|chr7|45144505|45144641|-|::|::
TGCAGTCAAGTCAAATTCAGTGCCCGTTTCTGTCATAGCGGGGGCTGGCCCAGATGGCTGCCACAGCAAGCTCCACAGCTCATGGGCCCTGGGTCACCT
ACCCTGGGACCTGGGGATAAGTTTGGCTGTGGACAGTG
..............................................................HHHHHH................................
................................AAA...
.(((((((..((.....(((..((((((........))))))..))).....)))))))))..........((((((.((....(((..((((.((.
.....))))))..))).......)))))))))......
>SNORA6|chr3|39449882|39450030|+|::|::
TGCACACTATTAAAGCTCAGGGTGGAGGCCAGTCTTGGCTCATGAACTTCTGAGTGTCGGAAGTGTGCTATATCAATGGCAGGATTTTCGCTAACACCA
GTAGAGCTTGCCTCTATGACTGGAGTTTGGTAGTACTCGCTGCCACATAG
.....................................................................HHHHHH.........................
............................................AAA...
.(((((((.......(((((.((.(((.((......)))))....))..))))).......)))))))........(((((((......(((((.(((
((((((.....)))))...))))).)).)))......)).)))))......
>SNORA60|chr20|37078012|37078149|+|::|::
CACCTGCATTCAAAAATGATCACGGGCTGCCTGTGCTCTGGTCATCAATAACGCAGGGAGAGGAATTGCTGAAAGCCGTTTCCCGTGTTTGGAGGGTTC
ACACCTGTCCCTTTCAAATGCTGGCGCTTTCACACACTC
..........................................................HHHHHH....................................
.................................AAA...
..(((((........(((((((.((((.......)))))))))))......)))))...........(((((((.....((.((((((((((((..(
(....))..)))))))))))).)).))))))).......
>SNORA61|chr1|28906276|28906405|-|::|::
ATCCTCCTGATCCCTTTCCCATCGGATCTGAACACTGGTCTTGGTGGTCGTAAAAGGAGGAAAAGTAATAGTGAAGCTGGCCTAAATGTTGTAATCTGG
TATATGGCATGTGGGCTAGTTTCAGACAGGT
.............................................................HHHHHH.................................
.........................AAA...
.(((((((((((...........))))).((.(((........))).)).....))))))..........(((((((((((.((((((((......

..)))))))).)))))))))))))).......
>SNORA76|chr17|62223699|62223831|+|::|::
GCGCTGTCTTTGAGCCCCCGCCGAGCTTCCTCGTGGCGCCGGGGGTCAATCTGCAGCGCTAGAGCATGTGCTTGCGCATAACTGGGGCCGCCTGGCCTC
CCGCGGGCGGCCTTTTTAACCGCGAGCGACAAGA
............................................................HHHHHH.................................
............................AAA...
((((((...((((.(((((((((((....)))..))))..)))).))))....)))))).........((((((((.........(((((((((.....
...)))))))))........))))))))......
>SNORA77|chr1|203698709|203698833|+|::|::
GCAGACTCACTATGCACCTGACTGTACTTCCAGGCAGGTGCTTTTTCTGTCTGCCAGAGAAACATTCCAGGGTGCTGTGGCTGCCTCACCTATCCAGGG
CGATGCAGCTCCCTGGGGACACAGGT
.......................................................HHHHHH......................................
....................AAA...
((((((.......(((((((.(((......))).))))))).......))))))..........((((((((.......(((((.((.((.......))
.)).))))).))))))))........

>SNORA78|chr16|2015185|2015311|+|::|::
GTTGGTTGAAAATCGCCCCCGGCTTTGGCCGTGGCCGCGGGTGAGATTCGGCGCCCAGAGCCCCCGGGGGCCTCAGCTCACCGCGCGCTGCCCCATGTG
CGGCGGTGAAACCCAGGCCCCGACAGGC
.........................................................HHHHHH.....................................
......................AAA...
....((((((..((((((.((((..........)))).))))))..))))))...............(((((((....(((((((((((........)))
).))))))).....))))))).......
>SNORA79|chr14|81669039|81669178|-|::|::
TGATGACTGTTCTTCCTGTTGCCTTCAGACCTGGCATTAGGATTTAGGTCATCATAAATTAAAATTTGATTTTCTTCACTCAGAGAATATTAGGATAA
TGGAGTTATCTGTTCTCTGAAATTTGCGGAATCAAACATGT
.........................................................HHHHHH.....................................
...................................AAA...
(((((((......((((..((((.........))))..)))).......)))))))...........(((((..((..((.((((((((((....(((((
.....)))))))))))))))....))..)))))))......
>SNORA80|chr21|33749496|33749631|-|::|::
GCATGGGTTTGGATTTATGATGGGCCCGTCCCCCTGGACCTCTCATAGTACCCCATGCCAGAGCAAACTGTAGCCCTGAACCATTGCCTGGCCTCTGTT
CCCGTAGGCTGCTGGCACTGAAGTGGGTTGCACAATA
............................................................HHHHHH..................................
...............................AAA...
(((((((.....(((.((((.(((...((((....))))))))))))))..)))))))............(((((((.....((.((((..((((.....
.....))))....)))).))....)))))))......
>SNORA80B|chr2|10586840|10586975|-|::|::
GCATGGGTTTGGATTTATGACAGGCCCGTCACCCTGGGCCTGTCATAGTACCCCATGCCAGAGCAAACTGTGTCCCCGAACCATTGCCTGGCCTCTGTG
CCCGTAGGCTGCTGGCACTGAAGTGGGTTGCACAGTG
............................................................HHHHHH..................................
...............................AAA...
(((((((.....(((.(((((((((((........))))))))))))))..)))))))...................(((.(((((.....(((.....(
((....)))....))).....))))).))).......
>SNORA7A|chr3|12881811|12881949|-|::|::
GACCTCCTGGGATCGCATCTGGAGAGTGCCTAGTATTCTGCCAGCTTCGGAAAGGGAGGGAAAGCAAGCCTGGCAGAGGCACCCATTCCATTCCCAGCT
TGCTCCGTAGCTGGCGATTGGAAGACACTCTGCGACAGTG
.............................................................HHHHHH.................................
..................................AAA...
..(((((......((...(((((((((((...))))))).))))...)).....)))))..............((((((.......(((((.((((((((
........)))))).)).)))))....)))))).......
>SNORA7B|chr3|129116053|129116191|-|::|::
GACCTCCTGGGATCGCATCTGGAGACTGCCTAGTATTCTGCCAGCTTCGGAAAGGGAGGGAAAGCAAGCCTGGCAGAGGCACCCATTCCATTCCCAGCT
TGCTCCGTAGCTGGTGATTGGAAGACACTCTGCGACAGTG
.............................................................HHHHHH.................................
..................................AAA...
..(((((......((...(((((((.(((...))).))).))))...)).....)))))..............((((((.......(((((.((((((((
........)))))).)).)))))....)))))).......
>SNORA8|chr11|93465527|93465665|-|::|::
TGCACTGCATGGTATCTGCACTCAGCAGTTTACACCTGCTAGGGTGTTCAAAGGTCAGTGCTATAGAAATTCAGTATCTGGCATCGTTGGTTTTCTTGG
CTTTGTGCTTGTTAAACCTGGTATTTCTACTGATACAGTA
...............................................................HHHHHH...............................
..................................AAA...
.((((((......((((.((((((((((.......))))).))))).....))))))))))..........((((((......(((...(((((.....(
(.....)).....))))).))).....)))))).......
>SNORA9|chr7|45024977|45025109|-|::|::
TAGCAAGCCTCCAGCGTGCTTGGGTCTGCGGTGACCCTATGCATTCCTTCAGTGCTTGCTAGAACAGTTTTGAAACGGTTTGAGGCCTTGCCCTGCTCC
ATCCAGAGCAAGGTTATAGAAATTTCAGACAATG
.............................................................HHHHHH.................................
............................AAA...
.(((((((((..((..(((..(((((......)))))...)))...))..)).)))))))..........((((((...(((....((((((.(((....
...))).))))))....)))..))))))......
>SNORA62|chr3|39452545|39452698|+|::|::
TGTGCACATTGTTAGAGCTTGGAGTTGAGGCTACTGACTGGCCGATGAACTCGCAAGTGTAGGTAGTGTGCTACATGAGGGGCAAGTTTTCGCTAACAC
CACAAGGGTCTCTGGCCCAATGAGTGGAGTTTGATAGTAATTCTTGCTACAAGTA
.........................................................................HHHHHH.....................
................................................AAA....
...(((((((.......(((((((((..(((((.....)))))....))))).)))).......)))))))..........((((((..((....(((.(
(((..(((((...)))))....)))).))).......))..)))))).......
>SNORA63|chr3|186505088|186505222|+|::|::

AAAGCAGGATTCAGACTACAATATAGCTGCTAAGTGCTGTGTTGTCGTTCCCCCCTGCTTAAAATAAAGTTGTTTCTTAACTATACCTGTCTGCTATTCT
CCTGTAGCAGCCAGGGACGCTTGGTCTCATACATGT
.............................................................HHHHHH.................................
..............................AAA...
....................................................................................................
....................................

```
>SNORA47|chr5|76376260|76376396|-|::|::
TGGAGGACTGAGAAGGTGAGGCAGTTTTGCCCCGTGCTGCCTTCCACCGGTTAAGACCTCCAAAATCGAAGGGCTGCCCAGGCAGAGGATGTCCCCTTG
CCACCCTTGGAGGGGCAGCGCTGTGCTGGCCGACATTT
...............................................................HHHHHH.............................
...............................AAA...
(((((.((.((..((((((((((((.........))))))).)))).)).)).))))))............(((..((((.((..((((((....
(((....))).))))))...)).)))))))........
>SNORA35|chrX|113865259|113865386|+|::|::
CAGCACTGCCAAGTGACCCATTGGGCTCCATCTTGACCAACTGGGCATCAAGCGGTGCAAAAGCAAATCCCTCTCAAAGCTGGGAGAGTCACACCGTGG
GCTACTCCTGCATGCAGCTGGGTACATAT
..........................................................HHHHHH.................................
.......................AAA...
..(((((((....((.((((((((...........)))).))))))....)))))))..........(((......(((((((((((((........)
))).))))......))))))))......
>SNORA26|chr4|53579416|53579537|+|::|::
GTGCCCTTTTAAGGTTGACCCAGTGCTTTAAGAGGCTAACACAGAAGGGTAAAGTAAGTCTCCATAAAACCCAGAGAAGAGACTGGAAAGCTCCTCTTT
GGATCCTGTCTGGAGTCACAACT
.................................................HHHHHH...........................................
.................AAA...
..((((((((..((....))....((((....))))......))))))))........(((((......((((((.(((.((....))))).)))))
))........)))))........
>SNORA81|chr3|186504464|186504641|+|::|::
ACCTTCTTGTATAAGCACTGTGCTAAAATTGCAGACACTAGGACCATGTCTTGGTTTTTGCAATAATGCTAGCAGAGTACACACAAGAAGAAAAGTAAC
AGCACTAGATTGTAAAGACTGGGGTGGACCTCTTTCTTAATGTCCAATGTCCTTTGTCTTAAGATTTGGTGCAATATCT
..............................................................................................HHHHHH..
........................................................................AAA...
..((((((((......(((.(((((..((((((((.((((((((...)))))))).))))))))).....))))).)))....)))))))).........
.(((((((((..(((.(((.((..(((((...........)))))....))...))))))..))))))))).......
>SNORA11B|chr14|91592769|91592897|+|::|::
GGGGTGTGCTCAGAGCAGGGGGCCCGAAGAATGCCTCCTCTGTTTACAACACACCCAATAGGAATCTGGGGTCATTGTGACAAGGGACACAGAGCTTGT
GACCTCCCTACAAACAAATGCCCTACACAT
........................................................HHHHHH...................................
.......................AAA...
.(((((((.....((((((((((.........))).)).)))))).....))))))).........((((.((((((....((((((((.....)))
)...)))))....)).))))))))......
>SNORA11C|chrX|47248048|47248176|+|::|::
GGGGTGTGCTCAGAGCAGGGGGCCTGAAGAATGCTTCTTCTGTTTACAACACACCCAATAGGAATCTGGGGTCATTGTGACGGGGGGCACAAAACTTGT
GACCTCTCTATGAACAAATGCCCCACATGT
........................................................HHHHHH...................................
.......................AAA...
.(((((((.....((((((((((.........))..))))))))).....))))))).........((((.((((((...(((((((((((...))))
).))))))).....)).))))))))......
>SNORA11D|chrX|51806443|51806570|-|::|::
GGGGTGTGCTCAGAGCAGGGGGCCTGAAGAATGGCTCCTCTGTTTACAACACACCCAACAGGAAGCTGGGGTCATCGTGATGAGGGGCACAAACTTGTG
GCCTCCCTACAGACAAATGCCCTACATGT
........................................................HHHHHH...................................
......................AAA...
.(((((((.....(((((((((((........))).)))))))))).....))))))).........((((.(((..((.((((((((((((....))).
))..)))).))..)).)))))))......
>SNORA11E|chrX|51806443|51806570|-|::|::
GGGGTGTGCTCAGAGCAGGGGGCCTGAAGAATGGCTCCTCTGTTTACAACACACCCAACAGGAAGCTGGGGTCATCGTGATGAGGGGCACAAACTTGTG
GCCTCCCTACAGACAAATGCCCTACATGT
........................................................HHHHHH...................................
......................AAA...
.(((((((.....(((((((((((........))).)))))))))).....))))))).........((((.(((..((.((((((((((((....))).
))..)))).))..)).)))))))......
>SNORA36C|chr2|69747175|69747306|-|::|::
TTCCAAAGTGTTGAGTTCAGTCCAGGGCAGCTTCCCTGTTCTGTTAATTAAACTTTGGGACATTGAAATGGGCTAGGGGAGATGATTGGGTAGAAAGCA
TTATTTTATTCATTTGCCTCCCAGCCTACAAAA
..........................................................HHHHHH.................................
...........................AAA...
.((((((((.((((...(((..(((((......)))))..)))....))))))))))))).........(((((..(((((.....((((((((....
...)))))))))).....)))))))))).....
>SNORA38B|chr17|65736785|65736915|+|::|::
```

CCCTCCTACAAAGGCATGTCTATAGTTCCTTGTCTTTGGACATGTAAGAATTGGAGGCAAAGAAATGTGGACTTGGAGAAATCTGGGGCCAGCTTGCTC
TCCGCAGGCTCAAGATCAACCATCCCACATAG

..........................................................HHHHHH...................................
..........................AAA...

.(((((........((((((((.((........)).))))))))........)))))).........((((....................(((((((..
...)))))))........))))..........

>SNORA84|chr9|95054743|95054875|-|::|::
GCCCTGTGGTTGCTGGATGCTGTTGTGCATGGACAGCTCTCCAGTGGATTCGATGGGCCATAGCAATCCTGTGATTTATGCATGGAGGCTGCTTCTCCT
CAGCAGCTGCCATAGCCCGGTCGCTGGTACATGA
...........................................................HHHHHH..................................
............................AAA...
((((.....(..(((((.((((((.......))))))..)))))..).......))))............((((((...((((((.(((((((......
.))))))).)))).))..))))))..........
>SNORA11|chrX|54840803|54840933|+|::|::
GGGGTGTGCTCAGAGCAGGGGGCCCAAAGAATGGCTCCTCTGTTTACAACACACCCAACAGGAATCTGGGGTCATTGTGATGAGGGCGTCAAACTTGTG
GCTTCCCTATGAACAAACGTCCCCCAACACCT
.........................................................HHHHHH....................................
..........................AAA...
.(((((((.....(((((((((((........))).)))))))).....)))))))..........(((((...((((....((((.((((......))
))..))))....)))).....)))))......
>SNORA12|chr10|101996913|101997059|-|::|::
AGTTGTGGTGGTTTTCTTTTTGGCACATTTGTTAAGTTTTCAAATGGGCCTAACTCTGCCACATATATAATATCGGAGATGGCAAAGGCTTGTGACGGA
GATATCTCTCTTAAGCCTTTCCTGCATCAGAGAATGGCTCCCACATGT
..................................................................HHHHHH...........................
..........................................AAA...
...((((((((..........(((.((((((.........)))))).))).....))))))))...........((((..((.(((((((((.((.(((
....))).)).)))))))))))...............)))).......
>SNORA73A|chr1|28833877|28834083|+|::|::
TCCAACGTGGATACACCCGGGAGGTCACTCTCCCCGGGCTCTGTCCAAGTGGCGTAGGGGAGCATAGGGCTCTGCCCCATGATGTACAAGTCCCTTTCC
ACAACGTTGGAAATAAAGCTGGGCCTCGTGTCTGCGCCTGCATATTCCTACAGCTTCCCAGAGTCCTGTCGACAATTACTGGGGAGACAAACCATGCAG
GAAACAGCC
...................................................................................................
..........HHHHHH...................................................................................
...AAA...
...................................................................................................
...................................................................................................
.........
>SNORA73B|chr1|28835070|28835274|+|::|::
TCCAACGTGGATACCCTGGGAGGTCACTCTCCCCAGGCTCTGTCCAAGTGGCATAGGGGAGCTTAGGGCTCTGCCCCATGATGTACAGTCCCTTTCCAC
AACGTTGAAGATGAAGCTGGGCCTCGTGTCTGCGCCTGCATATTCCTACAGCTTCCCAGAGTCCTGTGGACAATGACTGGGGAGACAAACCATGCAGGA
AACATAT
...................................................................................................
........HHHHHH.....................................................................................
.AAA...
...................................................................................................
...................................................................................................
.......
>SNORA74A|chr5|138614468|138614668|+|::|::
CATCCAGCGGTTGTCAGCTATCCAGGCTCATGTGGTGCCTGTGATGGTGTTACACTGTTGGAAGAGCAAACACTGTCTTTATTGAGGTTTGGCTCCAAG
CACTGTTTTGGTGTTGTAGCTGAGTACCTTTGGGCAGTGTTTTGCACCTCTGAGAGTGGAATGACTCCTGTGGAGTTGATCCTAGTCTGGGTGCAAACA
ATT
..............................................................HHHHHH...............................
................................................................................................AAA
...
...................................................................................................
...................................................................................................
...
>SNORA74B|chr5|172447729|172447932|+|::|::
TTTCCAGCAGTAGTCAGCCATCCGGACAGAACCGTTCCTGTGATGGTGTTACACTGCTGGGAGAGGAATGTCTTGTCTTCATCCGGTTGCCTGCGCCAC
TGTTCTGGTGGCGTCTGGCACTGGTGCAAGACAGAGCTGTGCTTCCCCGAGAGTGTGCTAAGCATTCACTTTGGCTGCTTAGTTCTAGTCTGGGAGCAG
ACAGAG
.............................................................HHHHHH................................
...................................................................................................
AAA...
...................................................................................................
...................................................................................................
......
>SNORA75|chr2|232320511|232320647|-|::|::
GTCTTCTCATTGAGCTCCTTTCTGTCTATCAGTGGCAGTTTATGGATTCGCACGAGAAGAAGAGAGAATTCACAGAACTAGCATTATTTTACCTTCTGT
CTTTACAGAGGTATATTTAGCTGTATTGTGAGACATTC

..............................................................HHHHHH...............................
................................AAA...
.(((((((..((...(((...(((((.......))))).....)))....)).)))))))........((((((..((.(((.......(((((.((((
....)))))))))......)))))..))))))......
>SNORA64|chr16|2012974|2013107|-|::|::
ACTCTCTCGGCTCTGCATAGTTGCACTTGGCTTCACCCGTGTGACTTTCGTAACGGGGAGAGAGAGAAAAGATCTCCTCAGGACCTCGGATGGGCCTTA
CTGTGGCCTCTCTTTCCTTGAGGGGTGCAACAGGC
..................................................................HHHHHH...........................
.............................AAA...
.(((((((..(((((...(((..(((..((.....)).)))..))).......))))).))))))).......((((((((((.....((..((((...
....))))..))..))).)))))))..........
>SNORA65|chr9|130210780|130210916|-|::|::
TCAGCCACCCGCCACTGCACCTGACCAGGTCTCTGTTGGCTGGTGCAATCCAGTGGTGAGCTGATAGTAAACCCCAGCTTAGGAAACAGGGTTGTTCTT
CATGTGGATGACTCTGTGCCGAAAGCATGGGAACAGCC
...............................................................HHHHHH..............................
................................AAA...
.((((....((((((((....((((((.(((......))))))).))...))))))))).)))).........((((((((.((..(((((((..(((..
.....)))..))))))).))..)))).)))).......

>SNORA66|chr1|93306276|93306408|+|::|::
GTGCAAACTCGATCACTAGCTCTGCGTGATGTGGCAGAAGCGAAGGGAACCAGGTTTGCAAAAGTAACTGTGGTGATGGAAATGTGTTAGCCTCAGACA
CTACTGAGGTGGTTCTTTCTATCCTAGTACAGTC
............................................................HHHHHH..................................
............................AAA...
.(((((((....((.((.((((((((........)))).)))..)).))......))))))).........(((.((((((((........((((((...
...))))))......)))))))))))........
>SNORA67|chr17|7481273|7481409|+|::|::
ATCCAAGGTGATTCCCTCTCCAAGGGGACATCAGTGCCTCTCAGGAAAGTAGCAGCTTGGAATAGAATCTGGCATGCCTAAGGCCTTTGGGGAACTGGG
ATGCTTATTTCCTCTGCCTTCCTTGGCTGCCCACATGG
.............................................................HHHHHH.................................
................................AAA...
.((((((.((.....((.(((.((((((((....)).)))))..))).))...)).))))))).........((((.(((.(((((....((((((.((((
...)))).))))))).)))))....)))))))......
>SNORA68|chr19|17973397|17973529|+|::|::
ATTGCACCTAAACCCAAGAATCACTGTTTCTTATAGCGGTGGTTTAAACAGAGGTGCAAACAGCAAGCGGATCTTGTCGCCTTTGGGGGGCTGTGGCCG
TGCCCCTCAAAGTGAATTTGGAGGTTCCACAACT
...........................................................HHHHHH...................................
............................AAA...
.((((((((.........((((((((((......))))))))))......)))))))).........(((.(((.((((.((((((((((........
.))))))))))))))......))).)))......
>SNORA69|chrX|118921316|118921447|-|::|::
AAAGCAGGTTGCAATTACAGTGCTTCATTTTGTGGAAGTACTGCCATTATCCTGCTGAAAGAAAAGCCGTGTTAATCATTTTTGATTTTGCCTTTATGA
GGGTAAAATCATGACAGATTGACATGGACAATT
.........................................................HHHHHH.....................................
...........................AAA...
..((((((....(((..(((((((((........)))))))))..)))..)))))).........(((((((((((.....((((((((((((....)
)))))))))))).....)))))))))))......
>SNORA70|chrX|153628622|153628756|+|::|::
CCGCAGCCAATTAAGCCGACTGAGTTCCTTTCCTCATGGGGACCCAGTGTGCGATGGCTGCACACAGCAGCTTCCTTGGTAGTGTACGCAGCCTGTTGG
TTGTATGGGTTGCTCTAAGGGACCTTGGAGACAGGC
...............................................................HHHHHH...............................
..............................AAA...
..(((((((.....((..((((.(((((..........))))).))))..))..))))))).........((((..(((....((.(((((((((...
....)))))))))..))....)))..))))......
>SNORA70B|chr2|61644379|61644513|-|::|::
CTGCAGCCAATTAAGCTGACTGAATTCCTTTCCTTATGGGGGTCCAGTGTGCAATGGCTGTAAACAGCAGCTTCCTTGGTAGTGTATGCGGCCTGTTTG
TTGTATAGGTTGCTCTAAGGGACCTTGGAGACAGGC
...............................................................HHHHHH...............................
..............................AAA...
.((((((((.....((..((((.(((((..........))))).))))..))..)))))))).........((((..(((.......(((((((((...
....)))))))))........)))..))))......
>SNORA70C|chr9|119943345|119943479|-|::|::
CTGCAGCCAATTAAGCCAACTGGGTTCCTTTCCTCATGGGGGCCCATTGTGCAATGGCTGCAAACAGCAGCTTTCTTGGTAGTGTATGCAGCCTGTTTG
TTGTATGGGTTGCTTTAAGGGACCATGGAAACAGGC
...............................................................HHHHHH...............................
..............................AAA...
.((((((((.....(((((.((((((((..........)))))))))))).))..)))))))).........((((.((((....((.(((((((((...
....)))))))))..))....)))).))))......
>SNORA70D|chr16|71732470|71732604|-|::|::
CTGCAGACAGTTAAGCCAACTGAGTTCCTTTCCTCAGGGAGGCCCAGTGTACAATGGCTGCCCACAGCAGCTTCCTTGGTAGCGTACTCAGCCTGTTTC
TTGTATGGGTTGCTCTAAGGGACCTTAGAGACAGGC
...............................................................HHHHHH...............................
..............................AAA...
....................................................................................................
....................................
>SNORA70E|chr11|82752506|82752640|-|::|::
CTGCAACCAATTAAGCCGACCTAGTTCCTTTCCTCTTTGGGGCCTGGTGTTCAATAGCTGCAAACAGCAGCTTCCTTGGTAGTGTATGCAGCCTGTTTC
TTGTATGGGTTGCTCTAAAGGACCTTGGAGACAGCC
...............................................................HHHHHH...............................
..............................AAA...
.((((....(((.(((..(((..(((((..........)))))..)))))).)))...)))).........((((..(((.......(((((((((...
....)))))))))........)))..))))......
>SNORA70F|chr2|165544153|165544287|-|::|::

```
CTGCAGTCAATTAAGTGTACTGAGTTCCTTTCCTTATGGGGGCCCAGTGTGCAATGGCTGCAAACAGCAGCTTCCTTGGTGGTGTATGCAGCCTGTTTC
CTCTATAGGTTGCTCTAAGGGACCTTGATAATAGGC
...............................................................HHHHHH..............................
..............................AAA...
.((((((((.....((((((((.(((((..........))))).))))))))..)))))))).........((((((((........(((((((((...
....))))))))).))))))))..............
```

```
>SNORA70G|chr12|69021014|69021155|-|::|::
CTGCAGCCTATTAAGCCAACTGAGTTCCTTTCCTCATGGGGGGGCCCAGTGTGCAATGGCTGCAAACAGCAGCTTCCTTGGTAGTGTATGCAGCCTGTG
TGTTGTATTGTATGGGTTGCTCTAAGGGACCCTGGAGACAGTC
.................................................................HHHHHH............................
.....................................AAA...
.(((((((.(((..((..((((....(((((((....)))))))..))))..)))))))))))).........((((..(((.......(((((((((.
...........)))))))))........)))..))))......
>SNORA71A|chr20|37055949|37056086|-|::|::
CTCCTGCATCCGAAAGTGATCATAGGCTGCCTGTGCCTAGGTCATTGATAGTGCAGGGAGAGGAACGCTGAAAGAGGTTGTCCCCGTGTTTGGAGGGTC
CACCCTATCCCATCCGAACCCTGAAGCTTTCACACAACT
..........................................................HHHHHH...................................
.................................AAA...
.(((((((......(((((((.(((((.......)))))))))))).....)))))))..........((((((.............((((((((((..
........))).))))))).......)))))).......
>SNORA71B|chr20|37053843|37053980|-|::|::
TCCCTGTATTCGAAAGTGATCGTGGGCTGCCTTTGCCCTGGTCATTGATAGTGCAGGGAGAGGAATCAATGAAAGCGCTTCCCCGTGTTTGAAGGGTCC
ACTCCTATCCCTTCCAAACTCTGGAGCTTTCGTACATGC
..........................................................HHHHHH...................................
.................................AAA...
.(((((((((....(((((((..((((.......)))).)))))))...)))))))))..........((((((((...(((....((((((((((...
........))))).)))))...)))))))))))......
>SNORA71C|chr20|37058310|37058447|-|::|::
CCCCTGCATTCGAAAGTGATCATGAGCTGCCCGTGCCCTGGTCATTGGTAGTGCAGGGAGAGGAACTGCCGAGAGCACTTCCCCGTGTTTGGAGGGTCC
ACTCCTGTCTCTTCCAAACCCTGGAGCTTTCCCACACCT
..........................................................HHHHHH...................................
.................................AAA...
.(((((((((....((((((((...((.......))..))))))))...))))))))).............((((((...(((....(((((((((....
((....))..)))))))))...)))))))))........
>SNORA71D|chr20|37062505|37062642|-|::|::
CACCTGTATTCGAAAGTGATCGTGGGCTGCCTGTGCCCTGGTCATTGATAGTGCAGGGAAAGAAATCGCGGAAAGTGCTTCCCCGTGTTTGGAGGGTCC
GCTCCTGTCCCTTTCAAACTCTGGAGCTTTCTCACACCT
..........................................................HHHHHH...................................
.................................AAA...
..((((((((....(((((((..((((.......)))).)))))))...))))))))............(((((..(((((.....((((((((((...
.........))))))))))...))))))))))).......
>SNORA72|chr8|99054314|99054445|-|::|::
CTGCGAATATTCTCGCTGTTCTGATTTTGTAATAGTCAGGACAGGCTAAACATTCGCTATATTAAGACCATGCATGTGTCCCCAAACCTAGTTCTTTCC
CTAGGTCTGGTTTTATAAATGCTGGTGATAAAC
..........................................................HHHHHH...................................
...........................AAA...
..((((((......(((((((((((........))))))))))).......)))))).........((((.((((.(((..(((.((((((........
)))))).)))....))).)))))))).......
>SNORA16B|chr1|212526160|212526292|+|::|::
TGGCCTTTATCAAAGCTGCAGCTGCTTCCATGTAGCTGCTGTGGTCAAAAAGAAGCCAAGAGTGACAGTTTTCCTTGATGGTCATAGTTCTGTTTGCTG
TAACTGATCTGCAAGATTTTGGGAAAATACCATT
..........................................................HHHHHH...................................
..........................AAA.....
...................................................................................................
..................................
>SNORA57|chr11|62432894|62433042|+|::|::
CCTCCTTTTCTTGGCGGGGATCGGGCTTGTGGTGCCGCTCCCCGTAATGTACGGAGGAAGAGGGAAAGGGCTCTGGCCCCCTCGGCGTCATGTCTTCGG
TGCTGGCGGCTTCCCATCCGCTGGTTCTATCCTCAAACGCCGGGACACCG
..........................................................HHHHHH...................................
............................................AAA...
(((((........(((((((.(((((.....)).))).))))))).......))))).......................((((((((..((.....((
.((..((((........))))..)).))......)).))))))))......
```

# Conservation ZL1

```
# STOCKHOLM 1.0

hg19           AGAGAA--GCAA--AGTGATGAGTAA-TACTGGCTGGAGCCCC-AAAG----AGGCA--CGTGTGTGTGTTTGT-GTGTGTGTGT----------------------------------ATATGCTTGTCAGTGCATG--CACGTGTATGTCTGGGAGTACAAATGG-GTGCGACTGGTTGTAGGGAACTAGC-TATGTGCCTTCTA------TTAGGCC----ATGACAGTC--AAACTGATAAGATCTGA---TTGCTTCTCT
panTro3        AGAGAA--GCAA--AGTGATGAGTAA-TACTGGCTGGAGCCCC-AAAG----AGGCA--TGTGTGTGTGTTTGT-GTGTGTGTGT----------------------------------ATATGCTTGTCAGTGCATG--CACGTGTATGTCTGGGAGTACAAATGT-GTGCGACTGGTTGTAGGGAACTAGC-TATGTGCCTTCTA------TTAGGCC----ATGACAGTC--AAACTGATAAGATCTGA---TTGCTTCTCT
gorGor3        AGAGAA--GCAA--AGTGATGAGTAA-TACTGGCTGGAGCCCC-AAAG----AGGCACATGTGTGTGTGTTTGT-GTGTGTGTGT----------------------------------ATATGCTTGTCAGTGCATG--CACGTGTGTGTCTGGGAGTACAAATGT-GTGCGACTGGTTGTAGGGAACTAGC-TATGTGCCTTCTA------TTAGGCC----ATGACAGTC--AAACTGATAAGATCTGA---TTGCTTCTCT
ponAbe2        AGAGAA--GCAA--AGTGATGAGTAA-TACTGGCTGGAGCCCC-AAAG----AGGCA--CGTGTGTGTGTTTGT-GTGTGTGTGT----------------------------------ATATGCTTGTCAGCGCATG--CACGTGTATGCCTGGGAGTACAAATGT-GTGCGACTGGTTGTAGGGAACTAGC-TATGTGCCTTCTA------TTAGGCC----ATGACAGTC--AAACTGATAAGATCTGA---TTGCTTCTCT
nomLeu1        AGAGAA--GCAA--AGTGATGAGTAA-TACTGGCTGGAGCCCC-AAAG----AGGCA--CGTGTGTGTGTTTGT-GTGTGTGT------------------------------------ATATGCTTGTCAGTGCATG--CACGTGTATGTCTGGGAGTACAAATGT-GTGCGACTGGTTGTAGGGAACTAGC-TATGTGCCTTCTA------CTAGGCC----ATGACAGTC--AAACTGATAAGATCTGA---TTGCTTCTCT
rheMac2        AGAGAA--GCAA--AGTGATGAGTAA-TACTGGCTGTAGCCCC-AAAG----AGGCACATGTGTGTATGTTTGT-GTGTATGTAT------------------------------------ATGCTTGTCAGTGCATG--CGTGTGTATGTCTGAGAGTACAAATGT-GTGCGACTGGTTGTAGGGAACTAGC-TATGTGCCTTCTA------TTAGGCC----ATGACAGTC--AAACTGATAAGATCTGA---TTGCTTCTCT
calJac3        AGAGAA--GCAA--AGTGATGAGTAA-TACTGGCTGGAGCCCC-AAAG----AGGCACATGTGTGTGTGTGTGT-GTGTGTGTGTTTGTGTGT------------------GTGTATCTTTATGCTTGTCAGTGCATG--CACGTGTATGTCTGGGAGTACAAATGT-GTGCGACTGGTGGTGGGGAACTAGC-TATGTGCCTTCTA------TTAGGCC----ATGACAGTT--AAACTGATAAGATCTGA---TTGCTTCTCT
mm9            GGAGAA--GCAC--AATGATGAACAA-TACT-GCTGGAGCCAC-AGAG----AGGCACAAGTGTGTGTGCCAGA-TTGCCTGTGTGTAT--------------------------GTGTGT--CCTTGTCTGTGCATG--CACACGTGAGTCTGGGAGGATGGATGT-GTGTGACTGGTGGTAGGGACCTAGC-CCTGTGCCATCTT------CTGGGCT----GTGACAGTC--AAACTGATAAGATCTGA---TTGCTTCTCT
rn4            GGAGAA--GCAC--AATGATGAACAA-TACT-GCTGGAGCCCC-ATAG----AGGCACAAGTGTGAGTGC--GA-TTGCCTGTGTGTAT--------------------------GTGTGTGTCCTTGTCTGTGCATG--CACACAAGAGTCTGGGAGGATGGGTGT-GTGTGACTGGTGGTAGGGACCTAGC-CATGTGCCATCTT------CTGGGCT----GTGACAGTC--AAACTGATAAGATCTGA---TTGCTTCTCT
hetGla1        GGAGAA--GCAA--AGTGATGAGCAA-TATTAGCTGGAGTCCC-AAAA----AGGCATGAATG-------------------TGTGTCTGTG--------------------TATCTGT--GTCCTTGTCTGTGCATG--CATATGTGTGTCTGAGTGTACGAATGT-GTGTGACTGGTTGTAGGGAACTAGC-TATATGCCTTGTA------TTGGGCC----ATGACAGTC--AAACTGATAAGATCTGA---TTGCTTCTCT
cavPor3        GGAGAA--GCAA--AATGATGAGCAA-TTCTGGCTGGAGTCCC-AAAA----AGGCATGAGTGAGTGTGTGTGT-GTGTGTATGTGTCTGTGTA----------------TATGTCTGTGTATCCTTGTCTGTGCATG--CACACATGTGTCTGAGT--CCAAATGT-GTGTGACTGGTTTTAGGGAACTAGC-TATGTGCCTTGTA------TTGGGCC----ATGACAGTA--AAACTGATAAGATCTGA---TTGCTTCTCT
oryCun2        GGAGAA--GCAA--AATGATGAGCCA-CACTGGCTGGATCCCC-AAAG----AGGCACGTGTGTGTGTGTGTGT-GTGTGTGTGTGTGTCTG--------------------TCCGTGGCCGTCCTTGTCTGTGCGC---TCCCCGTGTGTCTGGCTATGCGTGTGT-GT----CTGGTTATAGGGAACTAGC-TGTGTGCCTTCTG------TTGGGCC----ATGACAGTC--AAACTGATAAGATCTGA---TTGCTTCTCG
susScr2        GGAGAA--GCAA--CGTGATGAGCAT-TGCTGGCTGGAGCTCC-AAAG----GGGCACCTGTGTGTGTGTG--T-GTGTGTGTGTGTGT--------------------------GTCTGTACCCTTGTCTGTGCATG--CACACGTGTGTCTGGGAGTATGTGTGA-GCGTGGCTGGTTGTAGGGACCTAGT-TATGTGCCTCCTT------TTGGACG----ATGATAGTCAAAAACTGATAAGATCTGA---TTGCTTCTCT
bosTau6        GGAGAA--GCAA--AGTGATGAGCAT-TACTAGCTGGAGCTCC-AAAG----AGGCACGCGCGTGCGTGGGAGT-ATGTGTGTGTGGGT--------------------------GTGTGTTTCCTTGTCTGTGCATG--CACACGTGTGTCTGGGAGTACGAATGC-GTGCGACTGGTTGTAGGGAACTAGT-TATGTGCCTCCTT------TTGGACC----ATGACAGTT--AAACTGATAAGATCTGA---TTGCTTCTTT
equCab2        GGAGAA--GCAA--AGTGATGAGCTT-TACTGGCTGGAGCCCC-AAAG----AGGCACGCGTGTGTGTATGTCT-GT----------G----------------------------TCTGTATCCTTGTCTGTGCATG--CACATGTATGTCTGGGAGTATGAATGT-GTGTGACTGTTAGTAGGGAGCTAGT-TATGTGCCTTCTG------TTGGATGGACCATGACAGTC--AAACTGATAAGATCTGA---TTGCTTCTCT
felCat4        GGAGAA--GCAA--AGTGATGAGCGT-TGCTGGCTGGAGCCCC-AAAG----AGGCACACGTGTGTGTGTGTGT-GTGTATGTATGTGTGTGTGTGTGTGTGTGTATGTATATATCTGTGCCCTTGTCTGTGCATGCACACGTATGTCTGGGAGTTTGAGTGTAT-GTGTGACTGGTGGTAGGGAACAAGT-TACGTGCCTTCTG------TTGGACC----ATGACAGTC--AAACTGATAAGATCTGA---TTGCTTCTCT
canFam2        GGAGAA--GCAA--AGTGATGAGCAT-TATTGGCTAGAGCCCC-AAAG----AGGCACATGTGTGTATGTGTGT-GTATGTATGTGTG----------------------------TCTGTATCCTTGTCTGTGCATG--CACATGTATGTCTGGGAGTACGAATGT-GTCTGACTGATTATAGGGAACTAGTCTATGTGCCTTCTG------TTGGACG----ATGACAGTC--AAACTGATAAGATCTGA---TTGCTTCTCT
ailMel1        GGAGAA--GCGA--AGTGATGAGCAT-TACTAGCTGGAGCCCC-AAAG----AGGCACGTGTGTG-----------TGTGTGTGTGTGT--------------------------ATCTGTATCCTTGTCTGTGCATG--CACACGTGTGTCTGGGAGTGTGAATGT-GTGTGACTGGTTGTAGGGAACTAGT-TATGTGCCTTGTG------TTGGACC----ATGACAGTC--AAACTGATAAGATCTGA---TTGCTTCTCT
myoLuc2        GGAGAA--GCAA--AGTGATGATCAT-TACTCGCTAGAGCCCT-GAAG----AGGCACGTGTGTGTGTGTGTGT-GTGTGTGTGTGTGTGTGT------------------GTGTGTGTGTATCCTTGTCTGTGCACGTACACATGTGTGTCTGGGAGTATGAATGT-GCGTGACTGGTTGTAGGGAACTAGT-TATGTGCCTTCTG------TTGGACT----ATGACAGTC--AAACTGATAAGATCTGA---TTGCTTCTCT
loxAfr3        GTTGAA--GCAA--AGTGATGAGCAT-TACTGGCTGGAGCCCC-AAAG----AGGCACGTGTGTGTATGTGGGT-GTGAATCTGTGT-------------------------------G-----CCTGTCTGGGTGTGAACACGTGTAT---GGGGTGTGTGACTGT-GTCTGACTGGCTGTAGGGAACTAGT-TACGTGCCTTCTG------TTGGACG----ATGACAGTC--AAACTGATAAGATCTGA---TTGCTTCATT
monDom5        TGAGAA--GCAA--AATGATGGATAT-TATTGGTTGGAGCCCT-AAAG----AGGCA-ATATGAATGTATATGA-ATGA----------------------------------------------CTGTATGTGCGCG----CACTTGTGCCTGGATGTATGACTGGAGTATGAATGAGGATAAGGACCTAGT-TATTTGCCTTTTT------TTATAGGG-CCATGATATTC--AAACTGATAAGATCTGA---TTGCTTCTTT
macEug2        AGGGAA--GCAA--AATGATGGATAC-TATTGGTTGGAGCCCC-AAAG----AGGCAAATGTGTGTCTGTATAT-GAA------------------------------------------------TGTATGTGCGCG--CGCACTTGTGCCTGGATGTATGACTGG-GTATGAATGGCGATAGGGACCTAGT-TATATGCCTTTTT------TATAGAGGGCCATGATATTC--AAACTGATAAGATCTGA---TTGCTTCTCT
ornAna1        GGAGAA--GCAA--CATGATGAGCAT-TATCTGTTGGAGCCCT-ACAA----AGGTATAAAAG-GTGTGTTTTT-GTGTGAGTGT----------------------------------GTGCGTTTGTGTGTGCGTGTGCTCAC-------TGAAGACATGCATGT-ATTTGAGTGGAGCTAGGGACTCAAG---TACCTTTTATC------TAGGGCT----ATGATATTC--AAACTGATAAGATCTGA---TGGCTTCTGT
galGal3        GAAGAA--GCAA--AGTGATGAATAA-TATTTCCTTGAGCCAA-AAAG----AGA-----GAGAG------------------------------------------------------------TTGTGTGTGTGTG--------------------TGGTTGTATGACAGGGGTGTTTGT-GAGAATTG-----TGTGCCTTATA-----ATTTGGTG----GTGACAGCT--AAACTGATAAGATCTGA---TTGCTTCTTT
melGal1        GAAGAA--GCAA--AGTGATGAATAA-TATTTCCTTGAGCCAA-AGAG----AGA-----GTGAG------------------------------------------------------------TCGTGTGTATGTG----------------------GTTGTATGACAGGGATGTTTGT-GAGAGTTG-----TGTGCCTTATA-----ATTTGGCA----GTGACAGCT--AAACTGATAAGATCTGA---TTGCTTCTTT
taeGut1        GAAGAA--GCAA--AATGATGAGAAA-CACTTGCATGAGCCGA-A--G----TGA-----GTGAG------------------------------------------------------------TTGTGTGTGTGTG--------------------T-GCAGTGGTGTGTGGTTGTTTGA-GAGAACTG-----TGTGTCTTATA-----ATTGGGCA----GTGATAGCT--AAACTGATAAGATCTGA---TTGCTTCTTT
anoCar2        AAAGAA--GCAA--AATGATGAGAAG-CCTTGCCTCGAACCCG-ACC--------ACCCTGTGTGCGAGTGAGC-GTGCGAGTGTGTG----------------------------GAG--ACGGATGAGTGTGAGCG--------GGCGCATGGGAGCTGTGATTG-GT-GGGCTTGGCA------------------------TT------CTGGGTG----ATGAGGGCC--AGACTGATAAGATCTGA---CTGCTTCTGT
xenTro3        CATAAAGGGCAA--AATGATGAGATC-ATCAGACTTGACTCCCCACAG----TGTGACTCGTG-GCACTTGTGT-GTGTTTGTG--------------------------------------TGCATGTTTGTGCATG--------------TGGGTGACTGGTTGA-GTGT------GAGTGAGCACCT------------TTCAC-----ATGGGTTC----CTGAGAGCT--AAACTGATAAGATCTGA---TTGCTTTCTG
danRer7        GCAGGA--ACAC--GATGATGAGAGC-GATGAGCTTTAGTCCAGAGAA----TGGTAC-TGTGTGAACGAT-----TGTGAGTGT------------------------------------TTGTTTGTGTGTGTGTG--------------TGTGTGTGTGTGTGA-GTGT-----GAAGT--------ACC-AATGA------TC------CTGGACA----CTGAAAGTA--AAACTGATAAGATCTGA---GTGTTCAAAT
tetNig2        TCGGCATGGGCC-GTGTGATGAGAGC-GATACTCTTTAGTCCAGTCAG----AGGTACCTTTGTGTTTGTG-------------TGT--------------------------------GCATACGTGTAGGTGTGTG------------------GGTAAGTATGT-TTAC-------AAT------C--AA-AATGTACCTTTTGAGTCACCTGGACG----CTGCAAGTA--AAACTGATAAGATCTGAGAATACCTGCTGA
fr3            TCAGCATGGGTA-ATGTGATGAGAGC-GATATTCTTTAGTCCAGTCAG----AGGTACTTTTGTGTGTGTCTTT-GTGTGTGTGT----------------------------------------ATGTGTGTTCATG--TTCA-----------------AAATG---TAC--CTTTTGAT---------------------TTAA------CTGGATG----CTGAAAGCA--AAACTGATAAGATCTGAGAATGCCTGCTGA
gasAcu1        GCATGG--GCGA--CGTGATGAGAGC-GATATGCTTTAGTCCA----------GTCAAAGGTACGTGTGTCTGG-GAGAGTGC--------------------------------------TTGACTGTATGAGTGTG--AAGA-----------------CTTTGT-GTGATTCTTGATGT----ACCTT-----TGAACAA---C------CTGGATG----ATGAAAGTA--AAACTGATAAGATCTGA---CCGTCCTTGC
oryLat2        TTAGTA-CGGGTGGCGTGATGAGAGC-GATACGCTTTAGTCCAGTAAA----AGGTACGAGTGCGTGTGTGAGT-GT--------------------------------------------CTGCTGGTGTGTGTGTG--G----------------------TTGC-TTGTTTCTCGATGT----ACCT----TTTGT---TCATT------CTGGACG----GTGAAAGTA--AAACTGATAAGAACTGAAAGCCCCAGCTAA
petMar1        ATAGAA--GCAG--GATGATGAAAAACTTTTTTCTTGAGCCCA-AAAATAAAATTCACACGTT-GTGTATTTGTTTTGTAATTGTGCGTA-------------------------ACACGTATTCACGTTTGTTCATG--TACACGTGT---T----GTTTATATGG-TTCTGTTTTAATGTTG-----CACA-CGTGTG------A------ACGGGCA----GTGGAAGGA--AAACTGATAAGATCTGA---CTGCTTCTAA
#=GC SS_cons   <<<<<<..<<<<...................................................................................................................................................................................................................................................>>>>>>>>>>
#=GC SS_anno   ...............CCCCCCC.....................................................................................................................................................................................................................GGGGGGGGGGGG.DDDD.............
//
```

# Conservation ZL4

```
# STOCKHOLM 1.0

hg19         GGTGATTCTTTTT----ATATGCCCTTCT-GATCCCAAGTATGG-CAGAAGG-AT-ATAAAGGTCACCAAGAATAATTTGGAGGTTGCCAAGGACTTATTTAA-AAGTGTTT-TCAAGTCCGACCGAGAACTC-TGACATTT
panTro3      GGTGATTCTTTTT----ATATGCCCTTCT-GATCCCAAGTATGG-CAGAAGG-AT-ATAAAGGTCACCAAGAATAATTTGGAGGTTGCCAAGGACTTATTTAA-AAGTGTTT-TCAAGTCCGACCGAGAACTC-TGACATTT
gorGor3      GGTGATTCTTTTT----ATATGCCCTTCT-GATCCCAAGTATGG-CAGAAGG-AT-ATAAAGGTCACCAAGAATAATTTGGAGGTTGCCAAGGACTTATTTAA-AAGTGTTT-TCAAGTCCGACCGAGAACTC-TGACATTT
ponAbe2      GGTGATTCTTTTT----ATATGCCCTTCT-GATCCCAAGTATGGCCAGAAGG-AT-ATAAAGGTCACCAAGAATAATTTGGAGGTTGCCAAGGACTTATTTAA-AAGTGTTT-TCAAGTCCGACCGAGAACTC-TGACATTT
rheMac2      GGTGATTCTTTTT----ATATGCCCTTCC-GATCCCAAGTATGGCCAGAAGG-AT-ATAAAGGTCACCAAGAATAATTTGGAGGTTGCCAAGGACTTACTTAA-AAGTGTTT-TCAAGTCCGACCGAGAACTC-TGACATTT
nomLeu1      GGTGATTCTTTTT----ATATGCCCTTCT-GATCCCAAGTATGGCCAGAAGG-AT-ATAAAGGTCACCAAGAATAATTTGGAGGTTGCCAAGGACTTATTTAA-AAGTGTTT-TCAAGTCCGACCGAGAACTC-TGACATTT
calJac3      GGTGATTCTTTTT----ATATGCCCTTCT-GGTCCCAAGTATGGCCGGAAGG-AT-ACAAAGATCACCAAGAATAATTTGGAGGTTGCCAAGGACTTATTTAA-AAGTGTTT-TCAAGTCCGACCGAGAACTC-TGACATTT
mm9          GGTGACTTTTTTA----TATG-TCCCTCT-GACCTCAGTTTTGATCAAAGGGTTA-T-CAAGGTCACCAAGAACAATTTAGAGATTGCCAAGGACTTGTGCAA-AAGTGTTT-TCAAGTCTGACCGAGAACTC-TGACATTT
rn4          GGTGACTTTTTTA----TATG-TCCCTCT-GACCTCAGTTTTGATCAAAGGGTTA-T-CAAGGTCACCAAGAACAATTTAGAGATTGCCAAGGACTTGTTCAA-AAGTGTTT-TCAAGTCTGACCGAGAACTC-TGACATTT
oryCun2      GGTGAT---CTTT----GTGTGCCTCTCT-AGTCCCAAGTATGGCCAGAGAG-AT-ATCAAGATCACCAAGAATAATTTGGAGGTTGCCAAGGACTTGTTTAA-AAGTGTTT-TCAAGTCTGACCGAGAACTC-TGACATTT
oviAri1      GGTGACT-TTTTT----ATATGCCCTCTA--TCCCAAGACTATGGCCAGAGGGTT-ATTAAGGTCACCAAGAATAATTTGGAGGTTGCCAAAGACTTGTTTTTGAAGTGTTT-TCAAGTCTGACCGAGAACTC-TGACATTT
susScr2      GGTGGCT-TTTTA----TATGCCCTCTGG--TCCAAAGATTA-TGGTCAGAGGGA-TATTAAGTCACCAAGAATAATTTGGAGGTTGCCAAGGACTTGTTTTA-AAGTATTT-TCAAGTCTGACTGAGAACTCTTGACATGT
equCab2      GGTGACTTTTTAT----ATGC-CCTTCTG-GTCCCAAGAGTTTGACCAGAGGAAT-GTCAAGGTCACCAAGAATAATTTGGAGGTTGCCAAGGACTTGT-TTA-TAGTGTTT-TCAAGTCTGACCGAGAACTC-TGACATTT
bosTau6      GGTGACT-TT-TT----ATATGCCCTCTA--TCCCGAGACTATGGCCAGAGGGAT-ATTAAGGTCACCAAGAATAATTTGGAGGTTGCCAAAGACTTGTTTTTGAAGTGTTT-TCAAGTCTGACCGAGAACTC-TGACATTT
ailMel1      GGTGACTCTTTAT----ATGC-ACTTCTG-GTCCCAAGATTATGGCCAGAGGGAT-ATCAGGGTCACCAAGAATAATTTGGAGATTGCCAAGGACTTGTTTTA-AAGTGCTT-TCAAGTCTGACCGAGAACTC-TGACATTT
canFam2      GGTGACTCT--AT----ATGC-CCTTCTG-GTCCCAAGATTATGGCCAGAGGGAT-ATCAGGGTCACCAAGAATAATTTGGAGGTTGCCAAGGACTTGTATTA-AAATGTTT-TCAAGTCTGACCGAGAACTC-TGACATTT
felCat4      GGTGACT-TT-TT----ATATGCCCTTCTGGTCCCAGGATTATGGCCAAAGGGAT-ATCAGTGTCACCAGGAATAATTTGGAGGTTGCCAAGGACTTATTTTA-AAGTGTTT-TCAAGTCTGACCGAGAACGC-TGACATTT
loxAfr3      GGTGATTATTTTT----ATATGTCCTCTG-GTCCTGAGACTGTGGCCACAGGGAT-ATCAAGGTCACCAAGAATAATTTGGAGGTTGCCAAGGACTTGTTTAA-AAGTGTTT-TCAAGTCTGACCGAGAACTC-TGACATTT
myoLuc2      GGTGACTTTTTAT----ATAC-CCTTCTG-GCCCCAAGATTATGGCCAGAGGGAC-AGCAAGGTCACCAAGAATAATTTGGAGGTTGCCAAGGACTTGTTTTA-AAGTGTTT-TCAAGTCTGACCGAGAACTC-TGACATTT
macEug2      GGTGATCTTTCTC----ACTC-CCTGCTGGCAC-TGAGATTATTGCCAAAAGGATTATTAGGGTCACCTAAATTAAGTTGGATGTTGCCAAGGACTTGTTAAAAAGGTTTTTATCAAGTCTGACCGAGAACTC-TGACATTT
monDom5      GGTGATCTTTCTC----ATTT-CCTGCTGGCAC-TGAGATTATTGCCAAAAGGA-TATCAGGGTCACCAAAATTAAGTTGGATATTGCCAAGGACTTGTTAAAAAGGTTTTTATCAAGTCTGACCGAGAACTC-TGACAGCT
ornAna1      GGTGATCTTCCTC----ATTC-CTTTCTGGCACTTGAAACCATTGCC-CAAAGGATCTCGAAGTCACCAAGAATAAGTTGGAGGTTGCCAAAGGCTTGTTTGAAAAGGTTTA-TCAAGCCCGACCGAGAACTC-TGACATTT
galGal3      GGTGAT-CTTTTT-------TTCCTTCTG-GCATTGAGAGAGTTGCCTGAAGGT--AACAAGCTCACCAAGATTAAAATGGAGGTTGCCAAGGACTTGTTTAA-AAGATGCTATCAAGTCTGACCGAGAACTC-CAACATCT
melGal1      GGTGAT-CTTTTT------TTTCCTTCTG-GCATTGAGAGAGTTGCCTGAAGGT--AACAAGTTCACCAAGATTAAAATGGAGGTTGCCAAGGACTTGTTTAA-AAGATGCTATCAAGTCTGACCGAGAACTC-TGACATCT
taeGut1      GGTGATATTTGATTTTTTTTTCCTTCTG-GCATTGAGTCAGTTGCTTGAAGGT--ATCAAGGTCACCAAGATTAATATGGAGATTGCCAAGGACTTGTTTAA-AAGTGGTATCAAGTCTGACCGAGAACTC-TGACATTT
anoCar2      GGTGAT-CTTTTC-------TTCCTTCTG-GCATTAAGCCAGTTGCCAGAAGAT--ATCAAGGTCACCAAGAACAATCTAGAGATTGCCAAGGACTTGTTAAG-AAGTTTTTTTCAGGTCCGACCGAGAGCTC-TCACATGT
xenTro3      GGTGGCCTTTCAC-------TTCGCTTCTTGCATTAAGTCATTGCCTGAAGAAT--GTTGTGGCCACCAAAATAAAAATGGAGATGCCAAGGACCTGAGCAA-GAGTTGTGTCTGTGTCCGACCGAGA-CTCCCAACAAGT
danRer7      GGTGGTCTCTCCT------TTTCCT-CTG-CTCTGAAGTCTTATAGCAGCAGGAT-ACACAGACCACCAAGATAAACACAGAGAATGCCAAGGACCTGGGGCC-CAGAAACACCCAGATCTGACCGAGAACTC-TGACATTA
fr3          GGTGGTACTCTAT------TTGCCTGTA--CACTGAGAT--GTGTGTAGCAGGT--TATATAACCACCAAGAGCA--GGAGAGCATGCCAAGGACCTGGGCAGGAGGTTCACCCAGTGTCCGACCGAGAGCTC-TAACAACT
gasAcu1      GGTGGTCCTTCCT------TTTCCTGTG--CACTGAGGT--GTGTGCAGCAGGT--TACATGGCCACCAAGAGTA--TCGGAGTATGCCAAGGACCTGGACGGGAGACCCA-CCAGCGTCTGACCGAGAACTC-CGACAAAG
oryLat2      GGTGGTTTCTCCT------TTGCCTCTG--CACTCAGGTTAGTGTGCAGCAGGT--TATATGGCCACCCAGAGTA--ACAGAGCATGCCAAGGACCCGGGCTGGAGGTGAAGCCGGTGTCTGACCGAGAGCTC-TGACAGTC
tetNig2      GGTGGTGCTCCAT------TTGCCTGTG--CACTGAGGT--GTGTGTACCAGGT--TATATACCCACCGAGATCA--CGAGAGCATGCCAAGGACCTGGGCAGGAGCCCTGCTCTGCGTCCGACCGAGAGCTC-TGACAACC
#=GC SS_cons (((((.................((((.(.(((..........)))).)))).........))))))..........((((.........((((......................)))).........)).)).....
#=GC SS_anno ..............................................................HHHHHH...........GGGGG............................GGGGGGGGG......ACA...
//
```

# Conservation ZL5

```
# STOCKHOLM 1.0

hg19           ATGTCCAATGATTAAATTTTTTCCACTGCTGTTCACATCATGATTTGTTGTGATTAATG--TATGTGGATGAGGACAT
panTro3        ATGTCCAATGATTAAACTTTTTCCACTGCTGTTCACATCATGATTTGTTGTGATTAATG--TATGTGGATGAGGACAT
gorGor3        ATGTCCAATGATTAAACTTTTTCCACTGCTGTTCACATCATGATTTGTTGTGATTAATG--TATGTGGATGAGGACAT
ponAbe2        ATGTCCAATGATTAAACTTTTTCCACTGCTGTTCACATCATGATTTGTTGTGATTAATG--TATGTGGATGAGGACAT
nomLeu1        ATGTCCAATGATTAAACTTTTTCCACTGCTGTTCACATCATGATTTGTTGTGATTAATG--TATGTGGATGAGGACAT
rheMac2        ATGTCCAATGATTAAACTTTTTCCACTGCTGTTCACATCATGATTTGTTGTGATTAATG--TATGTGGATGAGGACAT
calJac3        ATGTCCAATGATTAAACTTTTTCCACTGCTGTTCACATCATGATTTGTTGTGATTAATG--TATGTGGATGAGGACAT
hetGla1        ATGTCCAATGATTAAACTTT--CCACTGCTGTTCACATCATGACTTGTTGTGATTAATG--TATGTGGATGAGGACAT
cavPor3        ATGTCCAATGATTAAACTTTC--CACTGCTGTTCACATCATGACTTGCTGTGATTAATG--TATGTGGATGAGGACAT
oryCun2        ATGTCCAATGATTAAACTTTTTCCACTGCTGTTCACATCATGACTGATTGTGATTAATG--TATGTGGATGAGGACAT
susScr2        ATGTCCAATGATTAAACTGTTC-CACTGCTGTTCACATCATGATTTGTTGTGATTAATG--TATGTGGATGAGGACAT
oviAri1        ATGTCCAATGATTAAACTTTCC-CACTGCTGTTCACATCATGATTTGTTGTGATTAATG--TATGTGAATGAGGACAT
bosTau6        ATGTCCAATGATTAAACTTTTC-CATTGCTGTTCACATCATGATTTGTTGTGATTAATG--TATGTGAATGAGGACAT
equCab2        ATGTCCAATGATTAAACTTTTTCCACTGCTGTTCACATCATGATTTGTTGTGATTAATG--TATGTGGATGAGGACAT
felCat4        ATGTCCAATGATTAAACTTTTTCCACTGCTGTTCACATCATGATCTGTTGTGATTAATG--TATGTGGATGAGGACAT
canFam2        ATGTCCAATGATTAAACTTTTTCCACTGCTGTTCACATCATGATTTGTTGTGATTAATG--TATGTGGATGAGGACAT
ailMel1        ATGTCCAATGATTAAACTTTTTCCACTGCTGTTCACATCATGATTTGTTGTGATTAATG--TATGTGGATGAGGACAT
myoLuc2        ATGTCCAATGATTAAACTTTTCTCACTGCTGTTCACATCATGATTTATTGTGATTAATG--TATGTGGATGAGGACAT
loxAfr3        GTGTCCAATGATTAAACTCTTTCCACTGCTGTTCACATCATGATTTGCTGTGATTAAGG--TATGTGGATGAGGACAT
macEug2        ATATCCAGTGATTAAACCTTTT-CACTGCTGTTCACATCATGAACCGTTGTGATTATTAAATATCTGTATGAGGATAT
ornAna1        GTGTCCAATGATTAAAATATTTTCACTGCTGTTCACATCATGACTTGCTGTGATTATTC--TATGTGGATGAGGACAT
#=GC SS_cons ((((((..................................................................))))))
#=GC SS_anno .......CCCCCCC..........gggggggggggggg.dddd......ccccccc............DDDD......
//
```

# Conservation ZL6

```
# STOCKHOLM 1.0

hg19           CATATCCAGTGATTAAACCTTTTCACTGCTGTTCACATAATGAACCGTTGTGATTATTGAATACTTAAATGAGGATATG
panTro3        CATATCCAGTGATTAAACCTTTTCACTGCTGTTCACATAATGAACCGTTGTGATTATTGAATACTTAAATGAGGATATG
gorGor3        CATATCCAGTGATTAAACCTTTTCACTGCTGTTCACATAATGAACCGTTGTGATTATTGAATACTTAAATGAGGATATG
ponAbe2        CATATCCAGTGATTAAACCTTTTCACTGCTGTTCACATAATGAACCGTTGTGATTATTGAATACTTAAATGAGGATATG
nomLeu1        CATATCCAGTGATGAAACCTTTTCACTGCTGTTCACATAATGAACCGTTGTGATTATTGAATACTTAAATGAGGATATG
rheMac2        CATATCCAGTGATTAAACCTTTTCACTGCTGTTCACATAATGAACTGTTGTGATTATTGAATACTTAAATGAGGATATG
calJac3        CATATCCAGTGATTAAACCTTTTCACTGCTGTTCACATAATGAACTGCTATGATTATTAAATACTCAAATGAGGATATG
mm9            CATATCCGATGATTAAACTCT-TCACTGCTGTTCACATACTGAGCCGTTGTGATTATTGAGGAACTA-ATGAGGATATG
rn4            CATATCCGATGATTAAACCCT-TCACTGCTGTTCACATAATGAGCTGTTGTGATTACTGAGGAACTG-ATGAGGATGTG
hetGla1        CATATCCAGTGATTAAACCTTTTCACTGCTGTTCACATAATGAACCATTGTGATTATTGAGTACTTAAATGAGGATATG
cavPor3        CATATCCAGTGATTAAACCCTTTCACTGCTGTTCACATAATGAACCATTGTGATTACTGAATACTTAAATGAGGATATG
oryCun2        CATATCCGGTGATTAAACCTTTTCACTGCTGTTCACATAATGAACCGCTGTGATTATCAAAAACTTAAATGAGGATATG
susScr2        CATATCCAATGATTAAATTTTCTCACTGCTGTTCACATAATGAACTGTTGTGATTATTGAATATGTAAATGAGGATATG
oviAri1        CATATCCAATGATTAAACATTTTCACTGCTGTTCACATAATGAACTTATGTGATTACTGAATACATGAATGAGGATATG
bosTau6        CATATCCAATGATTAAACATTTTCACTGCTGTTCACATAATGAACTTATGTGATTACTGAATACATGAATGAGGATATG
equCab2        CATATCCAATGATTAAACCTTTTCACTGCTGTTCACATAATGAACTGTTGTGATTATTAAGTCTGTAAATGAGGATATG
felCat4        CATATCCAGTGATTAAACCTTTTCACTGCTGTTCACATAATGAACCGTTGTGATTATTGAATACGTTAATGAGGATATG
canFam2        CATATCCAGTGATTAAACCTTTTCACTGCTGTTCACATAATGAACCGTTGTGATTATTGAATATATAAATGAGGATATG
ailMel1        CATATCCAGTGATTAAACCTTTTCACTGCTGTTCACATAATGAACTGTTGTGATTATTGAATATATAAATGAGGATATG
myoLuc2        CATATCCAGTGATTAAACCTTTTCGCTGCTGTTCACATAATGAACCGCTGTGATTATTGAATATGTTAATGAGGATATG
loxAfr3        CATATCCAGTGATTAAACCTTTTCACTGCTGTTCACATAATGAACCGTTGTGATTACTGAATATTTAAATGAGGATATG
macEug2        CATATCCAGTGATTAAACCTTTTCACTGCTGTTCACATCATGAACCGTTGTGATTATTAAATATCTGTATGAGGATATG
#=GC SS_cons   (((((((.................................................................)))))))
#=GC SS_anno   ........CCCCCCC.........gggggggggggggg.dddd......ccccccc............DDDD.......
//
```

# Conservation ZL8

```
# STOCKHOLM 1.0

hg19         GTGGTAATGATGATCTGGTTGGACAAGAGTCTCTGAGCTTTTCTCTGAGGATCTTTGAACCCACCTGATCCAC
panTro3      GTGGTAATGATGATCTGGTTGGACAAGAGTCTCTGAGCTTTTCTCTGAGGATCTTTGAACCCACCTGATCCAC
gorGor3      GTGGTAATGATGATCTGGTTGGACAAGAGTCTCTGAGCTTTTCTCTGAGGATCTTTGAACCCACCTGATCCAC
nomLeu1      GTGGTAATGATGATCTGGTTGGACAAGAGTCTCTGAGCTTTTCTCTGAGGATCTTTGAACCCACCTGATCCAC
ponAbe2      GTGGTAATGATGATCTGCTTGGACAAGAGTCTCTGAGGTTTTCTCTGAGGATCTTTGAACCCACCTGATCCAC
equCab2      GTGGTAATGATGACCTGGTTGGACTAGAGTCTCTGAACATTCCTTTGAGGATCTTTGAATCCACCTGATCCAC
oryCun2      GTGGTCGTGATGACTTGGTTGGACTAGAGTCTCTGAGCTTT-CTCTGAGGATCTTTGAGACCACCTGATCCAC
loxAfr3      GTGGGAATGATGACCTGGTTGGACTAGAGTCTCTGAGCTTTTCTCTGAGGATCTTCGAAACCACCTGATCCAC
#=GC SS_cons ((((.................................................................))))
#=GC SS_anno ......CCCCCCC...................dddd........ccccccc.............DDDD.....
//
```
